# Detecting Endogenous Retrovirus-Driven Tissue-Specific Gene Transcription

**DOI:** 10.1093/gbe/evv049

**Published:** 2015-03-11

**Authors:** Mihaela Pavlicev, Kaori Hiratsuka, Kayleigh A. Swaggart, Caitlin Dunn, Louis Muglia

**Affiliations:** Center for Prevention of Preterm Birth, Perinatal Institute, Cincinnati Children's Hospital Medical Center and Department of Pediatrics, University of Cincinnati College of Medicine

**Keywords:** long terminal repeats, LTR, endogenous retrovirus, placenta, transcriptome

## Abstract

Transposable elements (TEs) comprise approximately half of the human genome, and several independent lines of investigation have demonstrated their role in rewiring gene expression during development, evolution, and oncogenesis. The identification of their regulatory effects has largely been idiosyncratic, by linking activity with isolated genes. Their distribution throughout the genome raises critical questions—do these elements contribute to broad tissue- and lineage-specific regulation? If so, in what manner, as enhancers, promoters, RNAs? Here, we devise a novel approach to systematically dissect the genome-wide consequences of TE insertion on gene expression, and test the hypothesis that classes of endogenous retrovirus long terminal repeats (LTRs) exert tissue-specific regulation of adjacent genes. Using correlation of expression patterns across 18 tissue types, we reveal the tissue-specific uncoupling of gene expression due to 62 different LTR classes. These patterns are specific to the retroviral insertion, as the same genes in species without the LTRs do not exhibit the same effect. Although the LTRs can be transcribed themselves, the most highly transcribed TEs do not have the largest effects on adjacent regulation of coding genes, suggesting they function predominantly as enhancers. Moreover, the tissue-specific patterns of gene expression that are detected by our method arise from a limited number of genes, rather than as a general consequence of LTR integration. These findings identify basic principles of co-opting LTRs for genome evolution, and support the utility of our method for the analysis of TE, or other specific gene sets, in relation to the rest of the genome.

## Introduction

The acquisition of cell- and tissue-specific patterns of gene expression is central to morphological and physiological differentiation during development and evolution. The gene regulatory basis of phenotypic differences is often addressed by comparing the levels of gene expression between tissues within, or in the case of evolutionary divergence, between, species. Such comparisons appear relatively straightforward when single genes are of primary interest. However, tissue divergence into characteristic phenotypes involves suits of genes, coregulated in their spatiotemporal expression, often through shared flanking regulatory mechanisms. Transposable elements (TEs) have been proposed to play such coordinating role in development and evolution; yet detecting their tissue-specific regulatory signatures has been difficult. In contrast, obtaining profiles of tissue- or taxon-specific gene expression is relatively undemanding given modern sequencing technologies. Here, we present a straightforward approach to screen for tissue-specific signatures of TEs using transcriptomic data.

TEs have entered the genome in past viral invasions and comprise 50% or more of the human genome (de Koning et al. 2011). Although their transposing activity is often suppressed, the importance of these genomic elements in introducing genetic variation, enhancing plastic environmental responses, and in particular in long-term diversification of plants and animals, is well recognized (Britten and Kohne 1968; Kidwell and Lisch 2001; Deininger et al. 2003; Leib-Mosch et al. 2005; Medstrand et al. 2005; Feschotte and Pritham 2007; Feschotte 2008; Belyayev et al. 2010; Hua-Van et al. 2011; Bonchev and Parisod 2013; Casacuberta and Gonzalez 2013). Numerous examples of co-option of TEs into a series of crucial functions have been documented, including recombination, splicing, exonification, and various modes of gene regulation (e.g., Ayarpadikannan et al. 2015). Among the most prominent examples are effects on the regulation of adjacent genes. These effects include single TE recruitment into cis-regulation in a single lineage, as well as striking examples of multiple independent co-options of different TEs across species, for the regulation of orthologous genes in homologous tissues (apoptosis inhibitory protein; Romanish et al. 2007; prolactin in placental mammals; Emera et al. 2012). In another notable example, the envelope gene of distinct endoviruses has been recruited for the same function in placenta in different lineages (syncytin; Blaise et al. 2003; Dupressoir et al. 2005; Heidmann et al. 2009; Cornelis et al. 2014). Such multiple independent co-options suggest that TEs are readily recruited into function during the evolution.

TEs often manifest lineage- and tissue specificity of effects. The potential for tissue-specific or developmental stage-specific effects is prominently revealed in cancers, where particular TEs are often found to be highly expressed (Tomita et al. 1990; Patzke et al. 2002; Wang-Johanning et al. 2007; Gimenez et al. 2010; Lamprecht et al. 2010; Kim et al. 2013; Lock et al. 2014). Yet context specificity has been demonstrated also in normal development (Landry et al. 2002), including effects as fundamental as early cell fate determination (Macfarlan et al. 2012; Fort et al. 2014). Several features constitute the potential of TEs for rewiring gene regulation in a context-specific manner. First, by coding for specific binding sites, their effect can be restricted to the tissue context expressing the relevant DNA binding molecules (Schon et al. 2009; Wang et al. 2012). Second, due to their replication within genome, they can coordinately regulate multiple genes. Third, they are also restricted to the phylogenetic context, which they invaded, adding potential for discrete lineage differences. In particular the retroviral long terminal repeat (LTR) regions are inherently enriched in transcription factor binding sites (Sundaram et al. 2014) in order to use the host’s machinery for their own replication. The recruitment of LTRs into the function of a promoter, enhancer (Xie et al. 2013), or both has been shown to affect tissue-specific expression profiles, both in terms of recruitment of single genes and modification of multiple genes’ expression (below). Moreover, recently TEs have been found to be enriched in, and contribute to function and evolution of long intergenic noncoding (LINC) RNA (Kelley and Rinn 2012; Kapusta et al. 2013; Kapusta and Feschotte 2014). It is not clear whether these different modes of function are related and potentially coordinated across repeats of the same element, or whether they arise independently from each other.

Although transcriptomes are readily mined for the transcripts initiating in, or including TE sequences (Conley et al. 2008; Faulkner et al. 2009), the tissue-specific effects of TEs are not always reflected in their transcription. It has been difficult therefore to systematically reveal the associations of particular element with particular tissues. Here, we develop a systematic approach to detect tissue-specific effects of classes of TEs on gene-expression. We demonstrate the approach focusing on LTRs of endogenous retroviruses (ERV). The binding site-rich LTRs are remnants of ERVs in the genome after these have been deactivated. We detected multiple associations between LTR elements and tissues, driven by the expression of genes colocalized with LTR repeats. Several of these associations had been previously implicated, either due to their effect on tissue-specific regulation of single genes, or in some cases having a systemic effect in cancers. We then focus on expression of placenta-specific LTRs and find that the increase in LTR transcription in placenta relative to other tissues is largely due to a small number of repeats rather than the genome wide effects.

## Materials and Methods

The approach used in this article was developed to identify tissue-specific signatures of gene subsets at the transcriptome level. We focused on gene subsets with a particular LTR within 10 kb upstream of the transcriptional start site, in the same orientation of the linked gene transcripts, and considered whether the expression of these LTR-associated genes is potentially affected by the presence of the particular LTR element. In the event of genome-wide effects of the element on the transcriptional regulation in a particular tissue, we would expect that the expression pattern of LTR-associated genes in that tissue differs from expression pattern of other genes in the genome (detail below).

### Transcriptome Data

The transcriptome data used in this study were mapped by Kim et al. (2012). Raw data stem from publicly available Illumina Human Body Map 2.0 (HBM2.0) RNASeq data (73–83 million 50 bp paired-end reads from 16 normal nonplacental human tissues). It was mapped to the reference human genome sequence (hg19). We supplemented this data with two RNASeq transcriptomes of human reproductive tissues: human villous placenta at term and differentiated endometrial stromal cells in the cell culture (GP Wagner lab, Yale). Two replicates were profiled by RNASeq (single reads, 50 bp) and their average was used in the study. Although the transcriptome of cultured cells may not be fully representative of the in vivo transcriptome, it is particularly homogeneous, avoiding contamination of the signal with that of the adjacent tissues. The human placental sample represents two human samples profiled separately and subsequently averaged. The placental transcriptomes were sequenced at the Cincinnati Children’s Hospital Medical Center sequencing core, using Illumina sequencer and retrieving 30 million of 50 bp paired-end reads. Reads were aligned to human genome hg19 and processed using Cufflinks (Trapnell et al. 2010). We chose fragments per kilobase sequence per million reads (FPKM) = 1 as the lower cutoff for determining the presence of a gene transcript in the sample. Prior to the analysis of coexpression, we removed 221 ubiquitously highly expressed “house keeping” genes, defined here as those expressed in all 18 examined tissues at the FPKM values >50. We also removed genes not expressed in any tissue beyond expression threshold, resulting in 15,447 genes that entered the analysis. Inclusion of altogether 18 tissues allowed us to perform 136 comparisons between tissues, and therefore identify potential tissue-specific signatures, when an LTR consistently changed the gene coregulation between the specific tissue and majority of the remaining tissues. We tested 62 common LTR elements present in the human genome (table 1). The human genome assembly hg19 was used throughout the study.

**Table 1:** The List of LTR Elements (nomenclature following the RepBase [Jurka et al. 2005]; minimal phylogenetic distribution according to USCS, March 2015), Included in the Screen

LTR Element	Total Numbers in Human Genome	Numbers of 10 kb Upstream of Genes	Vicinity of Genes (%)	Taxon
LTR78	4,819	105	2.18	Mammals
LTR79	4,054	109	2.69	Mammals
MLT1M	2,956	108	3.65	Mammals
LTR10A	313	18	5.75	*Eutherians*
LTR16A	6,966	220	3.16	*Eutherians*
LTR33	9,260	301	3.25	*Eutherians*
LTR67B	3,717	124	3.34	*Eutherians*
LTR16C	6,631	218	3.29	*Eutherians*
LTR78B	3,281	65	1.98	*Eutherians*
LTR9	2,011	106	5.27	*Eutherians*
MER21C	5,501	192	3.49	*Eutherians*
MER54B	434	22	5.07	*Eutherians*
MLT1A	9,070	231	2.55	*Eutherians*
MLT1A0	20,643	590	2.86	*Eutherians*
MLT1A1	6,766	198	2.93	*Eutherians*
MLT1B	18,004	553	3.07	*Eutherians*
MLT1C	19,824	644	3.25	*Eutherians*
MLT1D	20,741	656	3.16	*Eutherians*
MLT1E1A	3,362	82	2.44	*Eutherians*
MLT1E2	3,996	102	2.55	*Eutherians*
MLT1F	4,297	167	3.89	*Eutherians*
MLT1F1	3,279	115	3.51	*Eutherians*
MLT1F2	6,036	203	3.36	*eutherians*
MLT1G	2,854	100	3.5	*Eutherians*
MLT1G1	3,592	120	3.34	*Eutherians*
MLT1H	10,094	273	2.7	*Eutherians*
MLT1H1	3,640	90	2.47	*Eutherians*
MLT1H2	4,714	145	3.08	*Eutherians*
MLT1I	11,089	312	2.81	*Eutherians*
MLT1J	15,270	560	3.67	*Eutherians*
MLT1J1	4,925	126	2.56	*Eutherians*
MLT1J2	6,925	203	2.93	*Eutherians*
MLT1K	18,173	617	3.4	*Eutherians*
MLT1L	12,074	377	3.12	*Eutherians*
MLT1N2	5,884	224	3.81	*Eutherians*
MLT2B1	4,480	111	2.48	*Eutherians*
MLT2B2	2,209	80	3.62	*Eutherians*
MLT2B3	3,313	87	2.63	*Eutherians*
MLT2B4	4,587	94	2.05	*Eutherians*
MLT2D	4,525	112	2.48	*Eutherians*
MSTC	3,169	128	4.04	*Eutherians*
LTR7B	848	50	5.9	*Primates*
LTR8	3,543	170	4.8	*Primates*
LTR12C	2,740	206	7.52	*Primates*
LTR12D	489	27	5.52	*Primates*
MER21A	1,921	117	6.09	*Primates*
MER39	3,337	73	2.19	*Primates*
MER39B	1,179	93	7.89	*Primates*
MER41B	2,852	126	4.42	*Primates*
MLT2A1	3,780	69	1.83	*Primates*
MLT2A2	3,898	99	2.54	*Primates*
MSTA	19,782	490	2.48	Primates
MSTB	8,562	247	2.88	*Primates*
MSTD	7,665	251	3.27	*Primates*
MSTB1	5,073	158	3.11	Primates
LTR2	887	61	6.88	*Anthropoids*
LTR22B	233	13	5.58	*Anthropoids*
LTR2B	326	34	10.42	*Anthropoids*
MER11A	964	53	5.5	*Anthropoids*
THE1A	4,233	93	2.2	*Anthropoids*
THE1C	9,874	233	2.36	*Anthropoids*
THE1D	12,642	305	2.41	*Anthropoids*

Note.—The number of repeats for each element, and the number, and percentage of the repeats that are localized within 10 kb upstream of genes in human genome. LTR: Long Terminal Repeats, MER: MEdium Reiteration repeats

### Scaling of Gene Expression

In order to arrive at meaningful conclusions from comparing gene expression across samples, it is desirable that the variance in gene expression is uniform across all levels of expression, rather than being a function of the mean expression level. To this end, data are usually transformed, and the appropriate transformation depends on the type of measurement (for microarray data, see Durbin et al. 2002). RNASeq gene expression is measured as a ratio of gene transcript reads in the total number of reads. The variance on this scale changes with the mean in a nonlinear fashion. The transformation appropriate for the proportional data is arcsine square root transformation (Sachs 1979). This transformation has been shown to be well approximated by the square root (Wagner et al. 2013), which is the transformation used on all transcriptomes in this article.

### Measuring Similarity of Gene Expression Profiles

The aim of this approach is to determine whether a particular subset of genes is being regulated in a tissue-specific manner. If so, the coexpression of this subset of genes in different tissues will show a significantly lower value than when measured in other, less tissue-specific genes. We measured the coexpression of genes across tissues by the Pearson product-moment correlation of the gene expression levels, which will be justified in detail below. This measure assesses to what extent the genes with high expression in tissue A tend to also be highly (positive correlation) or lowly (negative correlation) expressed in tissue B. Lack of coexpression of a set of genes between tissue A and all other tissues implies tissue specificity of gene regulation.

**Fig. 1.—:**
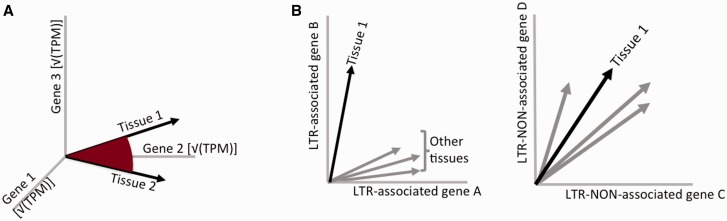
Schematic presentation of the transcriptome comparisons. (*A*) Similarity of transcriptomes relates to the angle in expression space spanned by the gene-axes, here shown as the 3-dimensional space of three genes. (*B*) Tissue-specific TE effect on gene expression is reflected as the subspace (here 2-dimensional space) of TE-associated genes, in which specific tissue shows lower similarity to other transcriptomes, when compared with the similarity between tissues in the space of other genes in the genome.

The gene expression profile of a cell type or tissue can be conceptualized as a vector in a high dimensional space spanned by the gene axes (fig. 1*A*). The tissue expression levels of each gene (i.e., score in each dimension, *g_i_*) define the tissue vector. The length of each vector can be calculated as $\sqrt{\sum {\left(g_{i}\right)}^{2}}$. As we use the square root of the expression values in this study $g_{i}=\sqrt{G_{i}}$ (where *G_i_* is the standardized measurement, like FPKM or transcripts per million [TPM] score), the length of each transcriptome vector reduces to $\sqrt{\sum {\left(\sqrt{G_{i}}\right)}^{2}}=\sqrt{\sum G_{i}}$. Note that the expression of single genes is measured as the proportion of the transcripts of particular gene in the total, and TPM measurement is explicitly conceived such that the sum of the expression scores of all genes in the transcriptome is constant across tissues (Wagner et al. 2012). It follows that the above vector length is constant and therefore only angles between these vectors are informative. Between-vector angle is proportional to the Pearson product-moment correlation of the two vectors representing the two transcriptomes. We therefore use the correlation between square root transformed scores as the measurement of similarity between transcriptomes (fig. 1*A*).

Within this space, capturing a subset of genes, which differ between the transcriptomes, entails identifying a subspace of genes, in which tissue vectors are particularly dissimilar. In this study, the specific subspace was chosen a priori as the set of genes potentially regulated by an LTR. We set out to test whether this subset of genes manifests lowered similarity of expression among tissues, relative to other genes (fig. 1*B*).

**Fig. 2.—:**
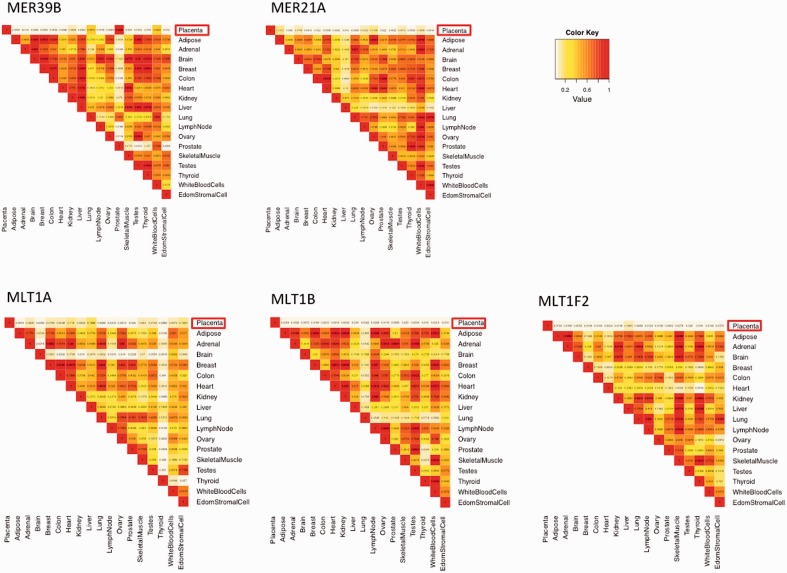
Heat maps for the five LTR elements that show the placenta-specific regulation of transcription. The genes colocalized with particular LTR elements are less coregulated (more divergent in expression) between pairs of tissues than genes selected at random. The color shows the odds ratio to observe the particular effect, for every pair of tissues. The presented LTRs show high specificity in placenta, as the LTR-associated genes show significantly lower coregulation with all other transcriptomes than random gene subsets.

To capture this potential effect of LTRs, we compared the correlation between tissues calculated from the LTR-associated genes (LTR+), and the correlation between tissues calculated from LTR-absent genes (LTR−). The result can be expressed as a ratio between the two similarity measures, LTR+/LTR−, for every pair-wise tissue comparison. This summary statistic allows us to assign the effect of an LTR in each tissue pair a single value, namely the correlation of the subset of genes relative to correlation across all other genes. We use absolute correlation values, because the magnitude, rather than the sign, is of interest. The resulting value ranges from 0 (when numerator equals 0) to a potentially undefined number (when denominator equals zero). The latter is not expected to be problematic, as while the similarity between tissues can be small, it is unlikely to be 0. The rationale for this summary statistics is that if there is no detectable effect of an LTR on gene expression pattern in either of the two tissues, the coregulation of LTR-associated genes will not differ significantly from the coregulation of remaining genes in the genome. Figure 2 and supplementary files S1–S4, Supplementary Material online, show the heat plots in which each square represents the odds ratio to observe the value for LTR+/LTR− as low or lower as the one observed. The odds ratio was calculated based on the null distribution of the statistics for each pair of tissues compared. Null distribution was generated by randomly resampling 5,000 times the set of genes corresponding to the number of LTR-associated genes and calculating the statistics (2,000 samples were used for 20- and 50-kb intervals). The observed value was compared with this null distribution to determine the significance of the observation. We have chosen to present the heat map with odds ratios rather than the values of the statistics itself, because the distribution of the statistics is specific to each square of the matrix, and hence the same value of the statistic may imply a significant observation in one, and a very common observation in another pair of tissue. The visual effect can in this case be misleading. The code for the described calculation is written in R (ver. 3.0.1) and is available from the authors.

### Determining the Genes Driving Tissue-Specific Signature

To determine whether a small number of genes are driving the expression pattern, we examined the effect of single genes on our measurement using jackknife, thereby removing single LTR-associated genes from the set of genes and recalculating the ratio of similarity between the subset and whole genome. The difference between the value based on a reduced set and the value based on a full gene subset is an estimate of the particular gene’s contribution. We recorded genes as contributing candidates if their removal increased the coregulation statistics by more than 5% of the value observed with full subset.

### Robustness

The interval of 10 kb upstream of the gene location was chosen arbitrarily in this work. Gene expression is known to be affected by the LTRs located further upstream of the genes. To test to what extent the detected signal is robust to changes in the length of genomic interval considered, we reanalyzed the data using intervals of 20 and 50 kb upstream of the coding genes and included TEs residing in these intervals. Longer interval inevitably results in a larger number of LTR-associated coding genes.

### LTR Expression in Placenta

In a closer examination of detected signals, we focused on the placental, lung fibroblast, and skeletal muscle myoblast transcriptomes. To examine whether the tissue-specific function of an LTR is also reflected in LTR transcription, we counted the number of reads mapping one or multiple times to the full genomic sequence of a particular LTR, and compared these between the tissue with which the LTR was associated in previous analyses, and tissues with no such association. To this end, we downloaded all genomic sequences for every LTR studied using UCSC table browser, and used Bowtie2 (Langmead et al. 2009) to map reads from the transcriptomes of placenta, skeletal myoblast, and lung fibroblast. Within these, we further identified all transcribed (>1 FPKM) repeats to which reads mapped uniquely. Even though noncoding DNA in the transcriptomes could stem from the contamination with genomic DNA, the expression pattern is highly similar between two independently processed biological replicates of placenta and it is therefore unlikely to be artifactual.

## Results

### Tissue-Specific Effects of LTRs on Gene Expression

To determine tissue-specific effects of LTRs, we asked whether a set of genes with transcription start sites within 10 kb downstream of an LTR (LTR-associated genes), manifest a distinct gene expression pattern in a tissue—a pattern that differs from the expression pattern of these genes in other tissues. As a metric, we used the degree of coexpression of LTR-associated genes between tissues, relative to coexpression of LTR-unassociated genes. We refer to this metric as the relative coexpression (see Materials and Methods for detail). The rationale of this approach is that the coexpression of genes represents similarity in their regulation between tissues, and therefore a group of genes with significantly lowered correlation is likely differentially regulated between tissues. We consider an LTR to have a tissue-specific regulatory effect when LTR-associated genes manifest significantly lower coexpression in comparison with multiple other tissues.

We tested 62 LTR elements (table 1) in 18 tissues. The heat maps in figure 2 show the effects of five TEs on relative coexpression. The score of the relative coexpression is specific to pairs of tissues (corresponding to single matrix elements) and is not comparable between different pairs of tissues. The presented values in the heat maps are therefore the significance levels (*P*). Significance values were generated in nonparametric tests separately for each pair of tissues. Each element of the matrix thus reports the odds of observing the value of the relative coexpression between tissue pairs as low or lower, when a gene subset is randomly selected from the genome. Figure 2 shows a subset of plots for LTRs found to be associated with placental expression. The results for the full set of tested LTRs can be found in supplementary file S1, Supplementary Material online. We report the significant LTR-tissue associations in table 2. We define “significant association” with a particular tissue when the LTR effect is seen in more than half of comparisons of that tissue with others.

**Table 2:** Detected Associations between the LTR and Tissues, as well as the Genes that Contribute Strongly to the Signature

LTR	Tissue	LTR-Associated Genes
LTR67B	ESC^a^	*HLA-DRA, MASP1, PSCA, RBP7, FABP7, SPON2, RASL11B, AP1S2, CRLS1, MAN2A1, FZD5, KIAA1217, CSRP2, DOK2, TYMP*
	Adipose^a^	*HLA-DRA, MASP1, PSCA, RBP7, FABP7, PREPL, AP1S2*
MLT1J2	Lung^b^	*NAPSA, SFTPB, SPTLC3, MB, ADAMTS5*
	Heart^b^	*MB, SFTPB, NPNT*
	Adrenal^b^	*CCL14, CXCL13, SPTLC3, MB, NAPSA, SFTPB, PIP, PLBD1, ADAMTS5*
	Thyroid^b^	*DAPK1, MB, NPNT, CXCL13, NAPSA, SFTPB, CCL14, PIP, ADAMTS5*
MLT1A	Brain^b^	*PSG1, MBP, LRRC39, CCK, F2, RARRES1, PDLIM3, IYD, FGR*
	Thyroid^b^	*PSG1, MBP, LRRC39, CCK, F2, RARRES1, PDLIM3, IYD, FGR, LIPG, CKS2, SLC26A7*
	Placenta^a^	*PSG1, MBP, LRRC39, CCK, F2, RARRES1, PDLIM3, IYD, FGR*
MLT1A0	Kidney^a^	*GPX3, PDZK1IP1, CASQ2, MYOM2, RBP4, SCGB3A1, FLNC, SYNPO2*
	Ovary^a^	*GPX3, MYOM2, RBP4, SCGB3A1, MGST1, RPL18A, MT1X*
MLT1B	Placenta^b^	*CRH, PSG2, PSG3, PSG4, PSG5, PSG7, ANG, PRR4, SLC22A1, SYNPO2*
MLT1F2	Placenta^b^	*CGA, AKR1C2, NPPA*
MLT1H2	Breast^a^	*ANKRD9, LEP, LOX, FKBP5, PLEKHB1, ITLN1, PRPS2, PPBP, WNT2*
MLT1C	ESC^b^	*G6PD, GDF15, S100P, SPARCL1, COL5A1, COL6A3, GPM6B, WFDC2, ARG1, HPD, ABLIM2, MKNK2, PPP1R1A, IYD, CPVL, LCP1*
MLT1E1A	Testes^b^	*CLPB, PRAME, ROPN1B, IDS, KCNIP4, PLA2G2A, COX5A, ZC3H14, VIPR1, ACPP*
	Colon^a^	*COX17, PLA2G2A, COX5A, ACPP, CLPB, ROPN1B, C1orf162*
	Lymph node^a^	*COX17, PLA2G2A, COX5A, ACPP, DECR1, ROPN1B*
	Prostate^a^	*COX17, PLA2G2A, COX5A, ACPP, CLPB, ROPN1B, C1orf162*
	Brain^b^	*EGFLAM, KCNIP4, PLA2G2A, IDS, VIPR1, ACPP, ROPN1B, ZDHHC18, CTHRC1, MYOCD, TMEM45A*
	ESC^a^	*COX17, PLA2G2A, COX5A, ACPP, CLPB, ROPN1B, C1orf162, MYOCD, TMEM45A, CTHRC1, IDS, KCNIP4, VIPR1, PRAME*
MLT1E2	Adipose^a^	*ADH1B, HADHB, ATP5EP2, COMMD6, MYLK2, GNLY, LYVE1*
MLT1J1	Adipose^b^	*ALDH2, RBP7, CES1, SMTN, AKAP6, NPNT*
MLT1J	Heart^b^	*CYP11A1, FABP3, MB, SERPINA3*
	Skel. muscle^a^	*CACNG1, CYP11A1, FABP3, HSPB6, MB, SOD2, SERPINA3, ADRA2C*
MLT1J2	Lung^b^	*NAPSA, SFTPB, SPTLC3, MB, ADAMTS5*
	Heart^b^	*NPNT, SFTPB, MB*
	Thyroid^b^	*NAPSA, SFTPB, CCL14, PIP, ADAMTS5*
	Lymph node^a^	*CCL14, CXCL13, SPTLC3, MB, SFTPB, PIP, ADAMTS5*
	Adrenal^a^	*CCL14, CXCL13, SPTLC3, MB, SFTPB, PIP, ADAMTS5, NAPSA, PLBD1*
MLT1M	Kidney^a^	*TMEM37, NEBL, SORBS2, PNRC1, ADAM33, LEPRE1*
LTR78	Adrenal^b^	*CXCL13, PPP1R1A, HLA-DRA, MRAP, RCAN2, PAH, RBP1, CLEC12A, G6PD, FGF7*
	Ovary^b^	*CXCL13, PPP1R1A, HLA-DRA, MRAP, RCAN2, PAH, RBP1, CLEC12A, G6PD, PLEK*
	Kidney^a^	*C1orf115, PPP1R1A, CXCL13, MRAP, RCAN2, PAH, FGF7, HLA-DRA, RBP1, MDH2, CLEC12A, PLEK, G6PD*
	Adipose^a^	*HLA-DRA, PPP1R1A, CXCL13, RCAN2, PAH, RBP1, LIPA, MDH2, CLEC12A, PLEK, G6PD*
MLT1N2	Skel. muscle^a^	*C2orf72, COX6A2, PFKFB3, NDRG4, PCOLCE2, DEFB1, HSP90B1, TNFSF10, PROK1, IGSF6, PLEK*
MLT2A1	Prostate^a^	*PIP, PLAGL1, SEMA3C, NKAIN2, ASB12, DIO2, ARL4C, TPD52L1*
LTR78B	Brain^b^	*DSCR3, GPM6A, PAFAH2, LTBP2, CCL18, LACTB, MSRA, TMEM38B, HVCN1*
LTR16A	Brain^a^	*DIRAS2, ENC1, NCAM1, SCG3, SCRN1, ANTXR2, CFI, FBP1, HABP2, KIAA1598, OAZ3, GIMAP2, G6PD*
LTR16C	WBC^a^	*CCK, EGFL6, RASA1, XIRP1, VWF, SYN2, NPPA, CPS1, C7, HCK, VCAN, IGFBP7*
MER39	ESC^a^	*ARHGAP8, SLC17A5, SRY, PTGER3, SORBS2, HGD, FHL5, HORMAD1, TNFSF13, FCGR2B, GPX3, SMOX, CADPS, GABRG2, TPD52L1, CLEC7A*
	WBC^a^	*ADHFE1, ARHGAP8, CLEC7A, GPX3, TNFSF13, AGTRAP, CADPS, GABRG2, NAGA, TPD52L1, PTGER3, SORBS2, HGD, FHL5, HORMAD1, SLC17A5, SRY*
**MER39B**	**Placenta**^b^	*RNF187, S100P, SCTR, HAS2, IDH2, CCR6, PAQR7, SCP2, PTK2B, KLRD1*
**MER21A**	**Placenta**^b^	***CYP19A1**, **ST6GAL1**, ANKS1B, FYCO1, HGD, AZIN1, LY6E, KIAA0368*
**LTR2**	**Prostate**^a^	*ACPP, FAM89A, FSCN1, ZFN32, EDNRB, SLC12A3, APOC1, ART5, CD5, OAS1, TYMP*
	**Liver**^a^	***APOC1**, FAM89A, FSCN1, EDNRB, ASNS, NECAP1, LIMS1, TYMP, ACPP, COIL, PDHB, SEPHS2*
	**Kidney**^a^	*FAM89A, FSCN1, SLC12A3, APOC1, IQSEC3, EDNRB, ACPP, ART5, COIL, CD5, OAS1*
	Ovary^a^	*ASNS, EDNRB, FAM89A, ZNF32, FSCN1, PDHB, SLC12A3, APOC1, ACPP, COIL, CD5, OAS1, TYMP*
**MER11A**	Breast^b^	*BAAT, ACER2, DYSF, LEP, AMACR, APOD, DCXR, PTGR1, IQCG, GIMAP4*
	Ovary^b^	*BAAT, ACER2, DYSF, LEP, AMACR, APOD, IQCG, GIMAP4, GSTM1, GSTM5, ACOT2*
	Lymph node^a^	*BAAT, LEP, AMACR, APOD, PTGR1, IQCG, GIMAP4, GSTM1, GSTM5, FSTL3*
	WBC^b^	*BAAT, ACER2, DYSF, LEP, AMACR, APOD, PTGR1, GIMAP4, FSTL3, GCNT1, GSTM1, GSTM5, CYP17A1*
	ESC^b^	*BAAT, DYSF, LEP, AMACR, APOD, PTGR1, FSTL3, GIMAP4, GSTM5, CYP17A1, IQCG, ACOT2*
	**Liver**^a^	***BAAT**, ACER2, DYSF, LEP, AMACR, APOD, DCXR, PTGR1, IQCG, GIMAP4, GSTM1, GSTM5, CYP17A1*
	Kidney^b^	*BAAT, ACER2, DYSF, LEP, AMACR, APOD, PTGR1, IQCG, GIMAP4*
THE1C	Liver^a^	*ALDH1A1, SPINK1, UGT1A1, CHN1, TM4SF5, ASB2, CD48, COL5A1, HLA-A, PAMR1*
**LTR12D**	Prostate^b^	***DHRS2**, RAB11B, BTNL9, ETFDH, AGMAT, LEPROTL1*
MSTA	WBC^b^	*CYP19A1, LYZ, SERPING1, AKR1C2, MYLK, PDE4DIP, C4BPA, CAP2, SELLN*
	Lung^a^	*CYP19A1, LYZ, AKR1C2, PDE4DIP, CAP2, FAM134B, SERPING1*

Note.—ESC, embryonal stromal cells; WBC, white blood cells. Boldface indicates the previously documented tissue-specific effects of the particular LTR/ERV on gene expression.

^a^Signifies weak effect; >4 of 17 tissue comparisons are significant.

^b^Signifies strong pattern; >7 of 17 tissue comparisons are significant.

### Single Gene Drivers of Tissue-Specific Expression Patterns

Even when LTRs show a tissue-specific signature, it is unlikely that all LTR-associated genes are involved in this signal. We determined the effect of single genes on the relative coexpression using jackknife. To this end, we measured the change in the score upon removing LTR-associated genes one at a time (Materials and Methods). This test was performed for all strong associations between LTR and tissue where significant effect on coexpression was shown in >50% of pairwise tissue comparisons (minimum 9 of 17). The main drivers of tissue-specific effects are listed in table 2. These genes were singled out in this study as genes the removal of which increased relative correlation score by ≥5% of the observed value. The choice of this threshold is arbitrary and governed in this study by the motivation to detect possible candidates for future experimental validation, rather than to exclude false positive cases.

There is no apparent pattern to the distances between the LTR and the genes, within the 10-kb interval. We measured the distance between the 3′-end of the LTR and 5′-end of the adjacent downstream gene, for the elements in which the association was found. The median distances for the different TEs range from 3 to 7 kb. Within the genes significantly associated with the particular element, the distances of single elements mostly manifest the full range, from 0.1 to 10 kb.

### Lineage Specificity of LTR Effect

A very common LTR may be associated with genes driving the expression pattern in a certain tissue by coincidence, especially if only a few genes are responsible. To test whether the effect of LTR-associated genes may be due to the genes themselves irrespective of the presence of the LTR, we repeated the analysis with the orthologous gene sets in mouse. Where possible, we specifically searched for the presence of the same signature in the expression of orthologous mouse genes, when the associated LTR is absent in mice. This test is complicated by the availability of matched transcriptomes. We found in two cases that the genes themselves maintained the same effect, meaning that the effect is likely not driven, even if potentially enhanced, by the presence of LTR in the human genome. This is the case for the effects of MER11-associated genes in ovary and breast, as well as the LTR2-associated genes in liver.

We observed moreover that even when LTRs are shared between mouse and human, the coexpression pattern is often not shared. One reason is certainly that between mouse and human, LTRs are rarely colocalized with the same set of genes (not shown). It has been noted previously that recruited LTRs often have a taxon-specific regulation even if they are present in multiple species (Cohen et al. 2009; Ward et al. 2013; but see Sundaram et al. 2014 for conserved effects also). In general therefore, if a TE is solely responsible for the tissue specificity of a gene set, this specificity should be lacking in the absence of TE. The opposite however is not expected; a shared functional TE does not necessarily imply shared tissue specificity of the same gene set in different species. This further underscores the flexibility of TE-mediated gene-regulatory evolution.

### Robustness of the Signal

We tested to what extent the detected signal depends on the length of genomic interval considered, by reanalyzing the data using 20- and 50-kb intervals upstream of the coding genes. Longer intervals resulted in a 2-fold (for 20 kb), respectively, 4.5-fold (for 50 kb) greater number of LTR-associated coding genes. The results are qualitatively congruent with the results from the 10-kb interval. However, the signal frequently weakens with increased length of the interval, implying that the genes contributing to the expression pattern are likely within a short interval of the LTR. In some cases, new associations can be revealed using longer intervals, such as the association of MER11A with placenta, or MLT1C with heart. These are likely due to effects of the additional organ-specific genes that have been included into the analysis when longer intervals are included. As LTR have been associated with long-distance effects, this is not surprising. Overall, the result supports the robustness of the approach. The heat maps corresponding to 20- and 50-kb intervals can be found in supplementary material for comparison.

### Placental Gene Expression Is Influenced by Specific LTRs

We examined in greater detail a subset of LTRs predicted to function in the placenta. A signal for placenta-specific effect on gene expression was detected for five LTRs: MLT1F2, MLT1A, MER39B, MER21A, and MLT1B. Notably, the effects of MER21A and MER39B in placenta have been reported previously (see Discussion).

### Sequence Divergence

**Fig. 3.—:**
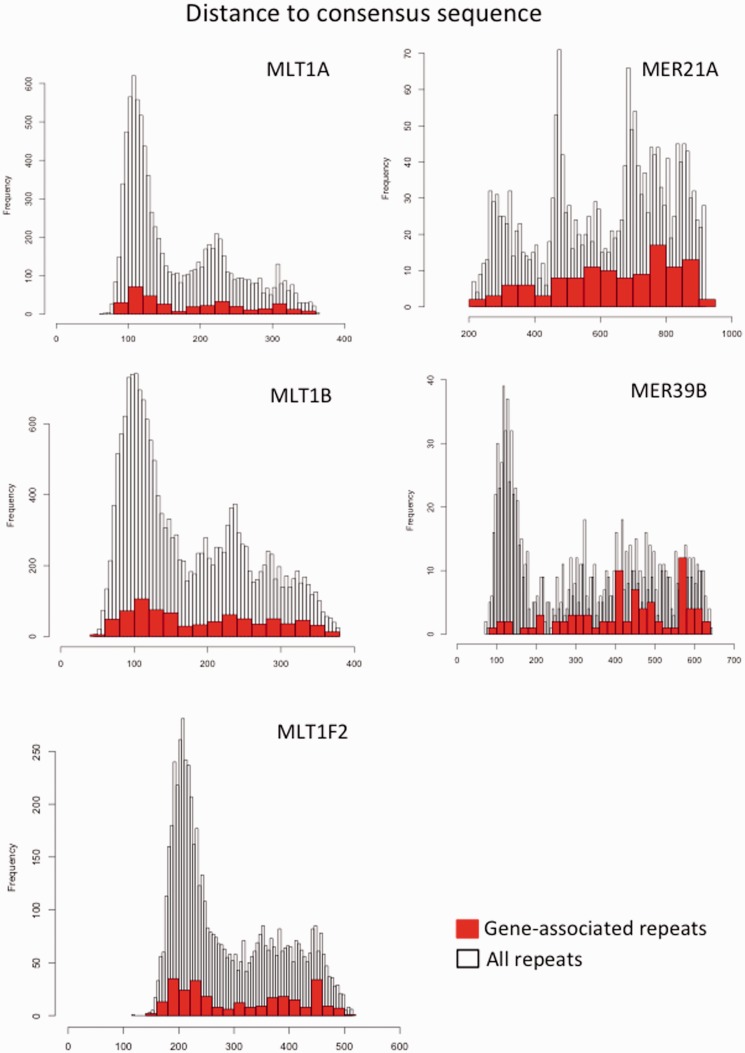
Distribution of sequence divergence from the consensus sequence for the five putative placental LTRs. The histogram in the background shows the distribution of the distances for all elements. The histogram in red color shows the distribution for the elements colocalized within 10 kb upstream of the coding genes. Note that consensus is an estimated sequence and may or may not resemble ancestral state (e.g., when there were unknown waves of recent viral activity).

To examine whether putatively functional LTRs—those within 10 kb upstream of the genes—share common sequences, we characterized the sequence conservation by comparing them to the consensus sequence (retrieved from Repbase [Jurka et al. 2005]). We used Levenshtein distance (Levenshtein 1966), which calculates the minimal number of single change steps between two strings and accounts for deletions and insertions, as these elements are often of different lengths. The result is proportional to the length of the sequence (*x* axis in the histograms). Figure 3 shows the distributions of Levenshtein distances for the gene-associated repeats of the five putative placental LTR (in red). The distribution is plotted against the background of all genomic repeats of the particular LTR. We see that the numerous MLT repeats have similar overall distribution. If we consider consensus sequence to be a proxy for ancestral sequence, then the high peak represents conserved elements, flanked by the diverged and possibly eroded repeats. Note that the gene-associated repeats in red largely follow this distribution. As opposed to the phylogenetically older eutherian MLT repeats, the two MER LTRs stem from more recent invasions, and are primate-specific. MER39B nevertheless shows similar overall distribution of sequence conservation, whereas MER21A is more diverse. In both, the gene-associated repeats tend to be found among highly diverged repeats.

### Expression of Placenta-Associated LTR

**Fig. 4.—:**
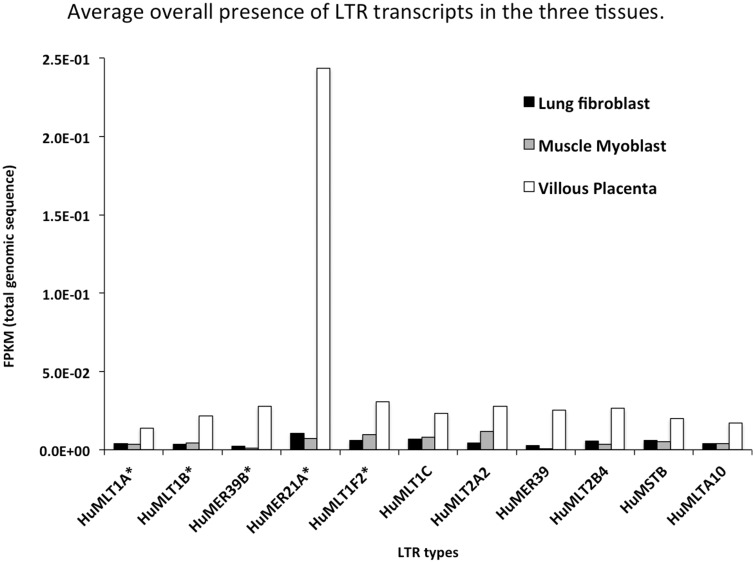
The overall levels of mapped (one or multiple times) paired reads to a set of LTR, using a placental transcriptome as well as that of two other tissues: skeletal myoblast and lung fibroblast. This mapping regards all members of particular LTR type, yielding very low normalized FPKM values. However, the consistently increased placental values for all elements are notable. The putative placenta-specific elements are marked with asterisk, and depending on the proportion of active elements involved, and the type of action, may or may not be distinguishable by increased genome-wide expression. “Hu” in the name refers to human data.

We also examined to what extent the LTR sequences themselves are expressed as an indicator of promoter (rather than, or in addition to, enhancer activity), and whether the LTR-containing transcript is specific to the tissue with which they are associated. We examined the levels of transcription of the placenta-associated LTRs (MLT1F2, MLT1A, MER39B, MER21A, and MLT1B), as well as several LTRs that were not associated with placenta. We compared these levels among placenta, lung fibroblast, and skeletal muscle myoblast transcriptomes (ENCODE data set). Figure 4 shows the results of the comparison of genome-wide transcription. The displayed FPKM values represent total reads aligned one or more times, standardized by the total length summed over all repeats of the particular element in the genome, and standardized by the total number of reads in transcriptome. The reported values of average transcription are typically very low, as expected due to normalization by the total genomic LTR sequences, which undoubtedly include many degraded and unexpressed elements. We nevertheless find a consistently higher expression of all LTR sequence types in placenta than in other tissues, regardless of whether the LTR has been detected as placenta-specific by virtue of proximity to placentally expressed genes (fig. 4). This result is in accordance with the generally held view that TEs are less suppressed in placenta compared with other tissues. Comparing between elements, we find high expression of MER21 in placenta, but otherwise no general tissue-specific signature in expression of putative placenta-specific elements. Thus, the LTRs more likely function as enhancers than as transcription promoters.

**Fig. 5.—:**
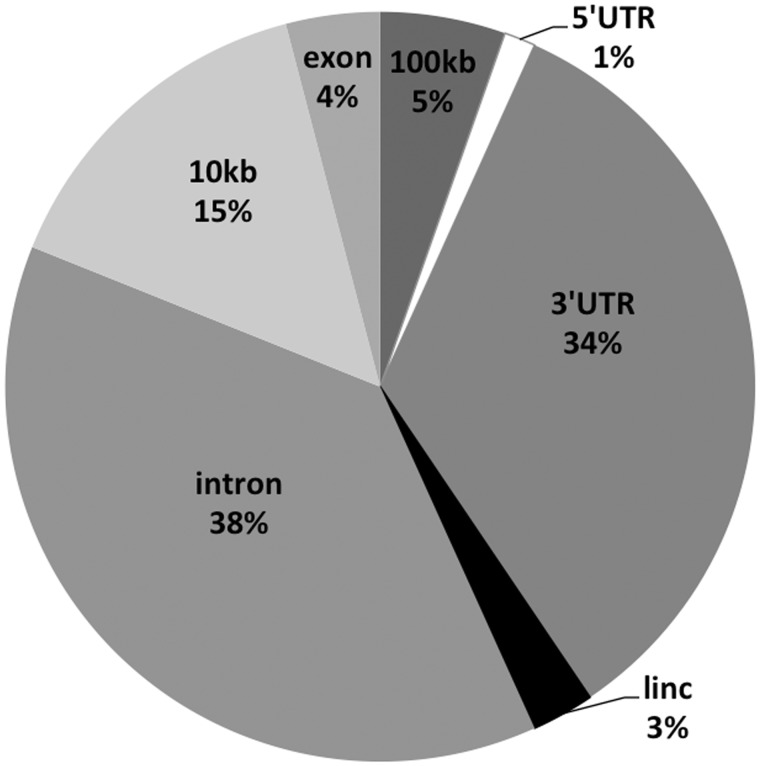
Proportion of identified expressed elements (pooled across the LTR types) in different genomic compartments. Note that most of these are not found immediately upstream of the genes, but rather in introns, LINC RNAs, and other intergenic regions.

Repeats pose a difficulty for transcriptome alignment, as many mapped reads cannot be uniquely assigned to a single genomic position. Thus it is not clear from the above result whether the reads stem from the overall baseline transcription, or from high transcription at only a small number of elements. We approached this question by focusing on the portion of reads that could be mapped unambiguously to single genomic loci. We found that of these loci, most manifest very low number of mapped reads, however few single repeats are moderately to highly transcribed (>10 FPKM). Although this pattern only considers unique loci, the few highly transcribed uniquely mapped loci account for a high proportion of the total reads that map to the genome: up to 85% of mapped reads in placenta, 51% of mapped reads in lung fibroblast, and 30% of mapped reads in skeletal myoblast, in the case of the elements examined here. It is also these highly expressed loci that account for differences in expression between tissues. The expressed LTR loci are located in various genomic compartments (fig. 5): most are found in introns of genes (38%) that are themselves either expressed or not expressed in the particular tissue, as well as in the 3′-UTR regions (34%). Only 15% of expressed elements are found in regions within 10 kb upstream of coding genes; 3% are parts of the known LINC RNAs (data set included in UCSC, December 2014), and 5% reside in intergenic regions without annotation, >10 kb from coding genes (fig. 5). The gene-associated repeats predicted to play a role in gene regulation in the first part of this study are not among this more highly expressed set.

**Fig. 6.—:**
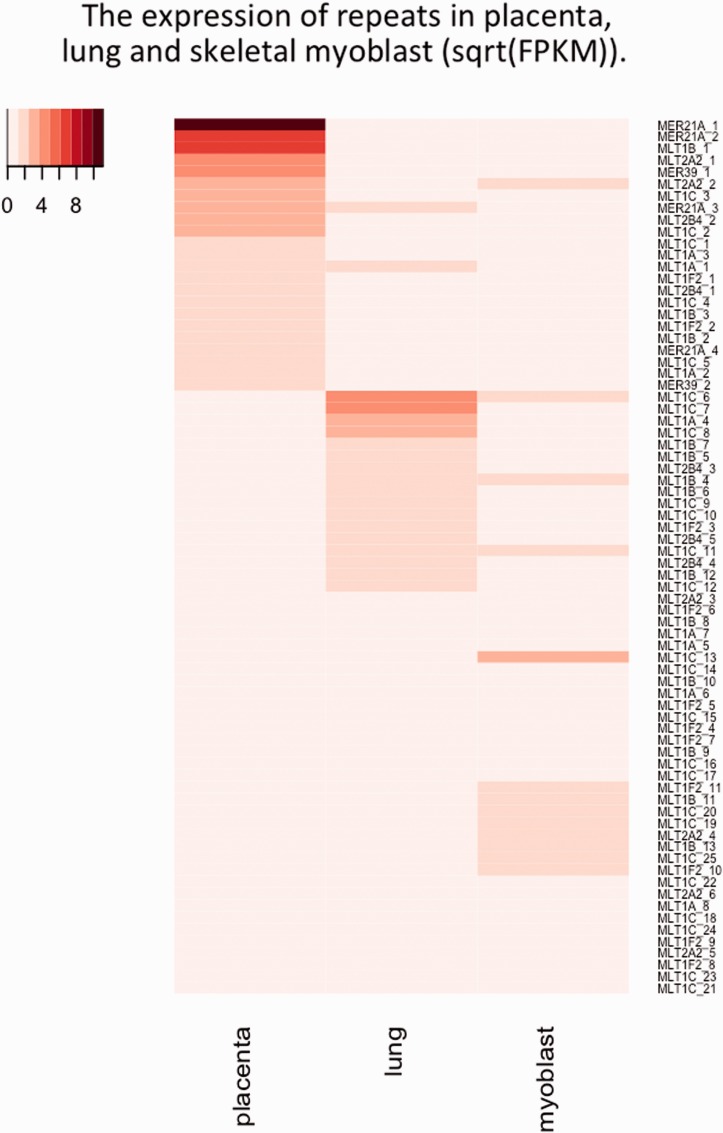
Comparison of transcription levels of individual elements selected for transcription >1 FPKM in either of the human placental tissue, lung fibroblast, or skeletal muscle myoblast. Only the elements from figure 4 were considered.

Figure 6 shows the expression levels of the single, particularly highly expressed individual repeats in placenta, myoblast, and lung fibroblast. All repeats of the LTR with FPKM >1 were included. Even though low, the expression appears tissue-specific, and the LTR class that was associated with the placenta due to effects on neighboring genes, is also predominantly expressed elsewhere in the genome. Overall, focusing on the portion of uniquely mapped reads, we find that much of the difference in number of mapped reads in placenta is due to very specific repeats rather than general expression, a finding arising also from the comparison of methylation patterns of different repeats across tissues (Reiss et al. 2007; Gimenez et al. 2009).

## Discussion

### Methodological Insights

The approach used in this article was developed to identify tissue-specific signatures of gene subsets at the transcriptome level. We used our computational tool on gene subsets with a particular LTR within 10 kb upstream of the transcriptional start site, in the same orientation of the linked gene transcripts. We considered that the expression of these LTR-associated genes is potentially affected by the presence of the particular LTR element. In the event of genome-wide effects of the element on the transcriptional regulation in a particular tissue, we would expect that the pattern of LTR-associated genes in that tissue differ from the coexpression pattern of other genes in the genome.

The method is a straightforward explorative tool, conceived to detect reliable signatures for subsequent experimental studies. Note that the interval of 10 kb was chosen arbitrarily in this study. We know that regulatory regions can be located at a greater distance from the gene. Two important factors influence the reliability of the results, one biological and another technical. First, transcriptomes represent snapshots in the development of cells, tissues, and organs. The effects of LTRs may occur at various times in development and may or may not leave a lasting signature on the expression of associated genes detectable at other developmental stages or physiological conditions. Not finding an effect therefore cannot exclude the role of an LTR in a particular tissue, as it can take place at different developmental times and under different conditions. The second factor affecting the results is technical. The method depends on the combinability of transcriptome data used. Transcriptional data have various sources of technical noise such as sample collection and preparation as well as the sequencing itself. FPKM measurement used here attempts to correct for the sequencing depth by normalizing to the total number of mapped reads. We relied in this study on previously reported FPKM values. However, Wagner et al (2013) showed that FPKM data still exhibit experiment specificity and proposed an alternative corrected measurement, TPM, to account for these differences. On TPM scale, the sum of expression measurements of single genes is constant across tissues, a property convenient for the use of correlations to assess similarity of transcriptomes, as is explained in the methods.

Two further aspects are important for signature interpretation. First, the correlation may be strongly driven by single genes with high expression. In such cases, the effect may or may not be associated with a functional LTR, as colocalization with a single gene may be coincidental. Such effects will be detected as single gene effects by the jackknife approach discussed above. Second, the degree of specificity revealed depends on the number and choice of tissues included in a study. In general, a greater number of tissues provides more fine-grained information about the uniqueness of a particular transcriptome and hence the specificity of the effects of particular LTRs.

### Concordance with Previously Reported Effects

This study suggested several cases of tissue-specific roles of LTRs. Using these signatures, we furthermore identified the genes with major contributions to this expression pattern. The relatively low numbers of genes colocalized with any one of the LTR elements preclude meaningful large-scale functional analyses such as gene ontology. To independently validate the results, we therefore examined previously documented functional associations between LTR-associated genes and the tissue for which they appear to be specifically regulated.

Indeed, we were able to replicate several of the LTR- gene-tissue associations that have been empirically determined previously (reviewed in Cohen et al 2009), supporting the utility of our approach. Specifically, this applies to the roles of MER21A and MER39B in placenta, as well as the roles of LTR12D and LTR2 in a wide array of tissues, and MER11A and LTR2 in liver in particular. Similarly, the role of MLT1J has been shown previously in regulation of *RCAN* in muscle (Serrano-Candelas et al. 2014), and is associated in our study with skeletal muscle and heart. Based on single fusion transcripts, the role of MLT1E1A has been detected previously in testes and muscle (Young et al. 2013). Furthermore, the specific genes *HSD17B* (MER21A), *BAAT* (MER11A), *DHRS2* (LTR12D), *APOC1* (LTR2), *ST6GALI*, and *CYP19A1* (MER21A), whose expression has been empirically found to be regulated by the associated LTRs, were also identified in our approach as the main contributors to the detected tissue-specific expression pattern. It is noteworthy that not all genes regulated by an LTR necessarily act in a tissue-specific manner. Because this approach focuses on tissue specificity only partial replication therefore may be expected. We found many novel associations that to our knowledge have no empirical evidence so far and will be of interest in future studies.

### Results from Healthy Tissue Overlap with Evidence for Disease and Cancer-Related Action

A particularly interesting set of replicated findings involve the associations previously considered cancer-specific. These include the effects of LTR2 in liver cancer (Medstrand et al. 2001) and kidney cancer (Takahashi et al. 2008; Cherkasova et al. 2011), LTR12D in general malignancy (Xu et al. 2013), and LTR16A in blood of the patients with multiple sclerosis (Beck et al. 2010). The role of ERV in cancer biology is well acknowledged (reviewed in Ruprecht et al. 2008; Katoh and Kurata 2013; Kassiotis 2014; Mourier et al. 2014; Xue and He 2014). Specific retroviral sequences are often found overrepresented in diseased tissue—whether the disease is triggered by the genetic or environmental perturbation. Such regulatory changes of LTR expression have been found to be tissue-specific and replicable, such as, for example, the expression of MLT1A in keratinocytes upon irradiation (Lee et al. 2012).

TEs have been proposed to increase evolvability by providing new genetic elements that are more likely than single mutations to have functional consequences. The potential for systemic tissue-specific effects stems from introduction of ready-made binding sites, as well as recently proposed associations with RNA genes (LINC, microRNA, Kelley and Rinn 2012; Harding et al. 2014). The expression of the elements themselves is frequently suppressed in the healthy tissue and can be enhanced by the environmental or genetic perturbation. Our results indicate however, that the tissue-specific effects are already detectable in healthy tissues, even if not as transcription of the elements themselves.

Given that the potential to coordinate expression of sets of genes is used in evolution of tissue-specific regulation, it is not surprising that many of the tissue-specific patterns of LTR-associated gene expression retrieved in this study based on normal transcriptomes, correspond to the patterns reported for carcinoma of the particular tissues. When activated in cancer, this module can substantially affect tissue behavior, yet this occurs not in a deregulated, but in a rather coordinated manner, and thus maintaining functionality for the cell, albeit not one that is in the long-term tolerable for the organism. The same principle that is thus thought to increase evolvability may also enable the efficiency of cancer.

### Expression of Tissue-Associated LTRs Themselves

In addition to tissue-specific effects on the transcription of LTR-associated genes, members of the same LTR groups show increased tissue-specific transcription. The potential drawback of our assessment is bias due to preferential recovery of the repeats with unique sequence, yet this bias appears relatively low given the high proportion of reads accounted for. Furthermore, because the same subset of repeats will be uniquely mappable across different tissues, this bias is consistent across human tissues, allowing valid comparison across tissues. We cannot exclude however that other, less distinct and thus less mappable elements are expressed in less tissue-specific ways across all these tissues, including the elements proximal to the expressed genes.

### Effects Are Not Limited to Placenta

Placenta is often regarded to be highly prone to activity of retroviral elements. Indeed, abundant evidence for the effect of TEs in placenta has been reported (Schulte et al. 1996; Sjottem et al. 1996; Bieche et al. 2003; Prudhomme et al. 2005; Huh et al. 2008; Gimenez et al. 2010; Chuong 2013; Chuong et al. 2013). We also found the general level of transcripts mapped to LTR regions to be higher in placenta than in other tissues for all LTRs tested (fig. 4). This supports the idea of a lower degree of LTR suppression in placenta. However, we also found that single repeats account for much of this difference in expression levels. On the contrary, all tissues in our data set appear similarly prone to the effect of LTRs on tissue-specific transcription pattern.

### Single Gene versus Systemic Effect

Some of the reported tissue-specific patterns seem to be dominated by the expression pattern of very small number of LTR-associated genes. Correlation as a measure is strongly affected by the outliers and therefore a strong change in expression of only a single or few genes can drive such effect. In this case, what appears to be a systemic effect of the whole subset is really an effect of one or very few genes in this subset. This can be easily detected in a subsequent jackknife analysis, as described above. These effects may or may not be associated with the common feature of a gene subset, such as the presence of the TE in the vicinity of their regulatory regions.

## Conclusions

In this manuscript, we develop a new approach to detect the tissue specificity of LTR effects, by detecting deviation in coexpression, utilizing information on gene coexpression across tissues. LTRs are often not the sole determinant of tissue specificity and their effects may therefore be hard to detect. The current approach accounts for this complication and measures the relative contribution to tissue-specific effects against the background of other genes. It can also detect the effects that are not associated with increased tissue-specific expression of particular LTRs themselves. Using this approach, we independently identified several previously known associations between LTRs and effects in particular tissues and the genes involved, as well as suggest further candidates. Focusing on the placental transcriptome, we confirmed previously established increased levels of LTR transcription and found that the placental increase in transcription is in large part due to few highly transcribed repeats that could be detected by unique mapping of reads. In addition, in placenta, myoblast, and lung fibroblast some tissue specificity of expression could be detected stemming from the repeats located largely in intronic or 3′-UTR regions of genes, and to much lesser extent in proximity of 5′-UTR. Collectively, these results indicate that tissue-specific actions of TEs exploit either multiple parallel mechanisms, some of which involve expression of TE and some do not, or a coordinated mechanism involving TEs localized proximally and distally from coding genes. To definitely show these associations are based on functional relationships, will require experimental work in the future.

Finally, the method developed here to detect tissue-specific regulation of LTR-colocalized genes sets is not limited to the analysis of LTR effects. Any subset of genes with a putative common feature (e.g., common function, sequence motif, or binding site) can be contrasted to the remaining genome in a similar way to detect tissue-specific or taxon-specific effect of an upstream regulation among tissues within an organism, or between homologous tissues in a phylogenetic context. Similarly, sets of candidate genes can be tested for changed coregulation across developmental stages, treatments, or pathological conditions.

## Data access

HumanBodyMap transcriptomes were retrieved from public data bases (see Materials and Methods). The reproductive tissue transcriptomes (placenta, endometrium) are available from GeneStation database at Vanderbilt University: http://genestation.org/Ohio_Collaborative.

## Supplementary Material

Supplementary files S1–S4 are available at *Genome Biology and Evolution* online (http://www.gbe.oxfordjournals.org/).

Supplementary Data
