# Supplementary material for: Detecting Endogenous Retrovirus-Driven Tissue-Specific Gene Transcription
Source: Genome Biol Evol. 2015 Mar 11;7(4):1082–97. doi: 10.1093/gbe/evv049 (PMC4419796; doi:10.1093/gbe/evv049)
Supplement: Supplementary Data [file supp_7_4_1082__index.html]

Detecting Endogenous Retrovirus-Driven Tissue-Specific Gene Transcription — Supplementary Data 

# Detecting Endogenous Retrovirus-Driven Tissue-Specific Gene Transcription

## Supplementary Data

files

**Files in this Data Supplement:**

- Supplementary Data - pdf file
- Supplementary Data - pdf file
- Supplementary Data - pdf file
- Supplementary Data - pdf file
