# Supplementary material for: Detecting Endogenous Retrovirus-Driven Tissue-Specific Gene Transcription
Source: Genome Biol Evol. 2015 Mar 11;7(4):1082–97. doi: 10.1093/gbe/evv049 (PMC4419796; doi:10.1093/gbe/evv049)
Supplement: Supplementary Data [file supp_evv049_S1_TePlots_10kb.pdf]

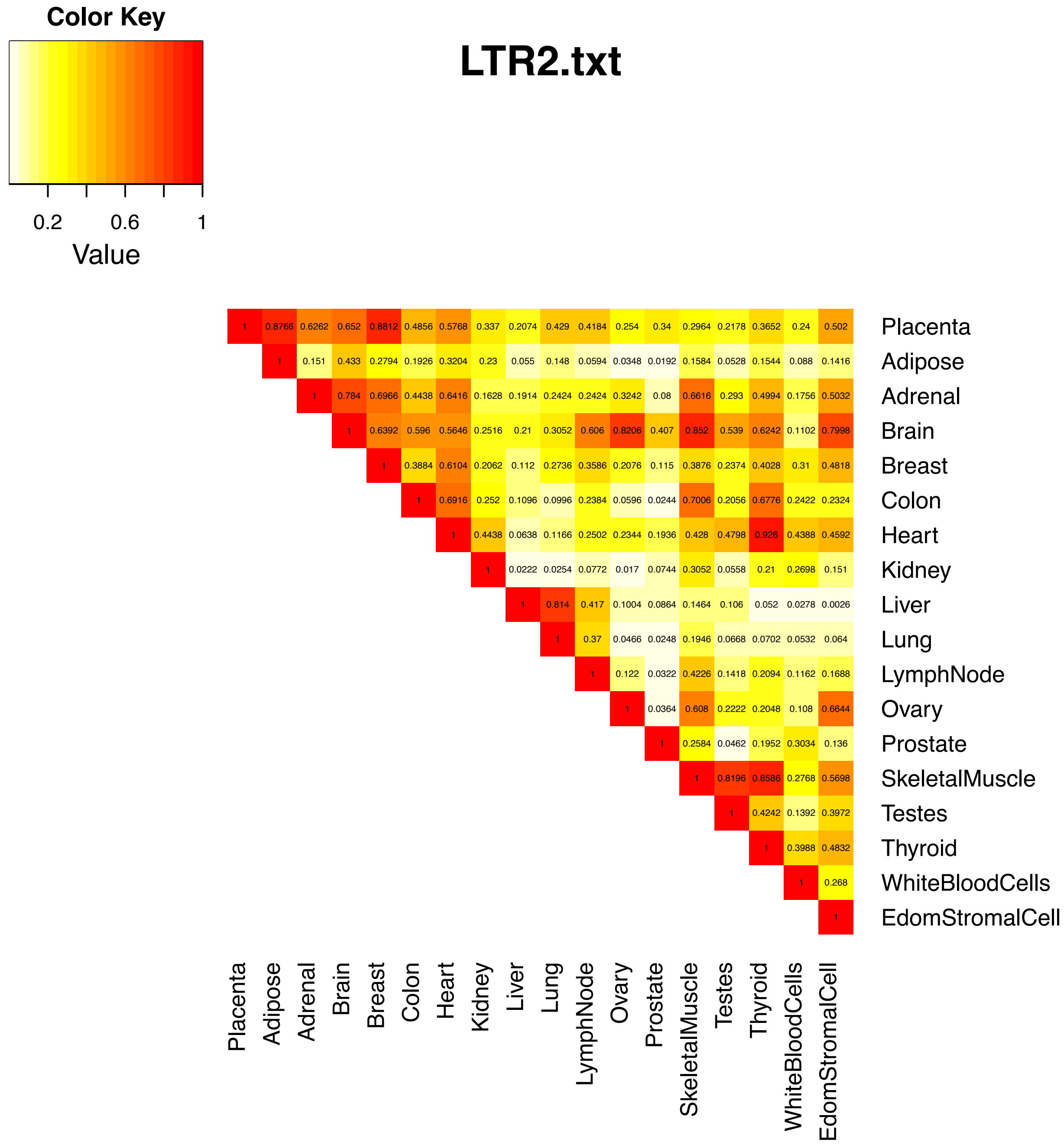
Color Key
0.2 0.6 1
Value
LTR2.txt
1 0.8766 0.6262 0.652 0.8812 0.4856 0.5768 0.337 0.2074 0.429 0.4184 0.254 0.34 0.2964 0.2178 0.3652 0.24 0.502 Placenta
1 0.151 0.433 0.2794 0.1926 0.3204 0.23 0.055 0.148 0.0594 0.0348 0.0192 0.1584 0.0528 0.1544 0.088 0.1416 Adipose
1 0.784 0.6966 0.4438 0.6416 0.1628 0.1914 0.2424 0.2424 0.3242 0.08 0.6616 0.293 0.4994 0.1756 0.5032 Adrenal
1 0.6392 0.596 0.5646 0.2516 0.21 0.3052 0.606 0.8206 0.407 0.852 0.539 0.6242 0.1102 0.7998 Brain
1 0.3884 0.6104 0.2062 0.112 0.2736 0.3586 0.2076 0.115 0.3876 0.2374 0.4028 0.31 0.4818 Breast
1 0.6916 0.252 0.1096 0.0996 0.2384 0.0596 0.0244 0.7006 0.2056 0.6776 0.2422 0.2324 Colon
1 0.4438 0.0638 0.1166 0.2502 0.2344 0.1936 0.428 0.4798 0.926 0.4388 0.4592 Heart
1 0.0222 0.0254 0.0772 0.017 0.0744 0.3052 0.0558 0.21 0.2698 0.151 Kidney
1 0.814 0.417 0.1004 0.0864 0.1464 0.106 0.052 0.0278 0.0026 Liver
1 0.37 0.0466 0.0248 0.1946 0.0668 0.0702 0.0532 0.064 Lung
1 0.122 0.0322 0.4226 0.1418 0.2094 0.1162 0.1688 LymphNode
1 0.0364 0.608 0.2222 0.2048 0.108 0.6644 Ovary
1 0.2584 0.0462 0.1952 0.3034 0.136 Prostate
1 0.8196 0.8586 0.2768 0.5698 SkeletalMuscle
1 0.4242 0.1392 0.3972 Testes
1 0.3988 0.4832 Thyroid
1 0.268 WhiteBloodCells
1 EdomStromalCell
Placenta Adipose Adrenal Brain Breast Colon Heart Kidney Liver Lung LymphNode Ovary Prostate SkeletalMuscle Testes Thyroid WhiteBloodCells EdomStromalCell

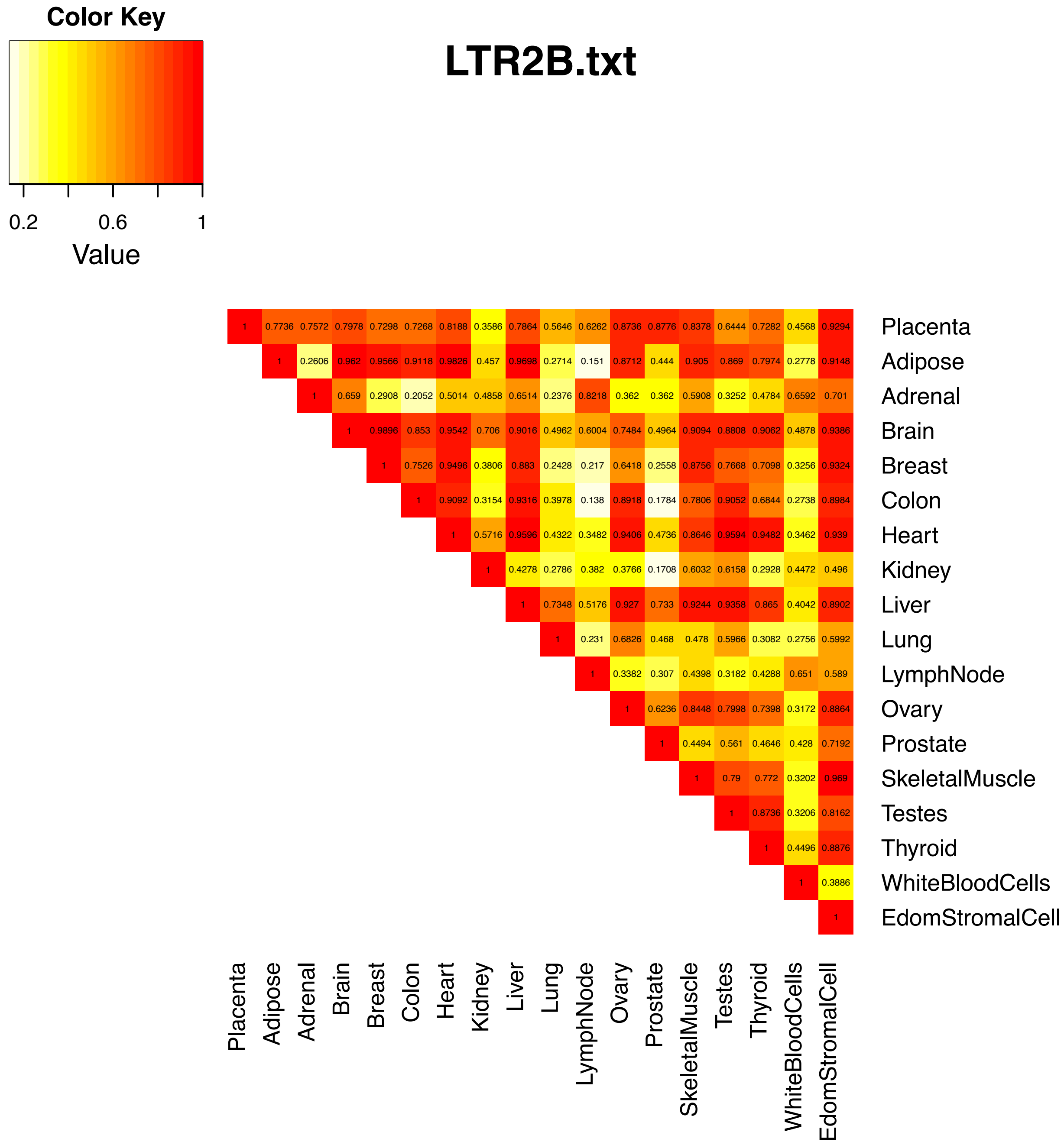
Color Key
0.2
0.6
1
Value
LTR2B.txt
1 0.7736 0.7572 0.7978 0.7298 0.7268 0.8188 0.3586 0.7864 0.5646 0.6262 0.8736 0.8776 0.8378 0.6444 0.7282 0.4568 0.9294 Placenta
1 0.2606 0.962 0.9566 0.9118 0.9826 0.457 0.9698 0.2714 0.151 0.8712 0.444 0.905 0.869 0.7974 0.2778 0.9148 Adipose
1 0.659 0.2908 0.2052 0.5014 0.4858 0.6514 0.2376 0.8218 0.362 0.362 0.5908 0.3252 0.4784 0.6592 0.701 Adrenal
1 0.9896 0.853 0.9542 0.706 0.9016 0.4962 0.6004 0.7484 0.4964 0.9094 0.8808 0.9062 0.4878 0.9386 Brain
1 0.7526 0.9496 0.3806 0.883 0.2428 0.217 0.6418 0.2558 0.8756 0.7668 0.7098 0.3256 0.9324 Breast
1 0.9092 0.3154 0.9316 0.3978 0.138 0.8918 0.1784 0.7806 0.9052 0.6844 0.2738 0.8984 Colon
1 0.5716 0.9596 0.4322 0.3482 0.9406 0.4736 0.8646 0.9594 0.9482 0.3462 0.939 Heart
1 0.4278 0.2786 0.382 0.3766 0.1708 0.6032 0.6158 0.2928 0.4472 0.496 Kidney
1 0.7348 0.5176 0.927 0.733 0.9244 0.9358 0.865 0.4042 0.8902 Liver
1 0.231 0.6826 0.468 0.478 0.5966 0.3082 0.2756 0.5992 Lung
1 0.3382 0.307 0.4398 0.3182 0.4288 0.651 0.589 LymphNode
1 0.6236 0.8448 0.7998 0.7398 0.3172 0.8864 Ovary
1 0.4494 0.561 0.4646 0.428 0.7192 Prostate
1 0.79 0.772 0.3202 0.969 SkeletalMuscle
1 0.8736 0.3206 0.8162 Testes
1 0.4496 0.8876 Thyroid
1 0.3886 WhiteBloodCells
1 EdomStromalCell
Placenta
Adipose
Adrenal
Brain
Breast
Colon
Heart
Kidney
Liver
Lung
LymphNode
Ovary
Prostate
SkeletalMuscle
Testes
Thyroid
WhiteBloodCells
EdomStromalCell

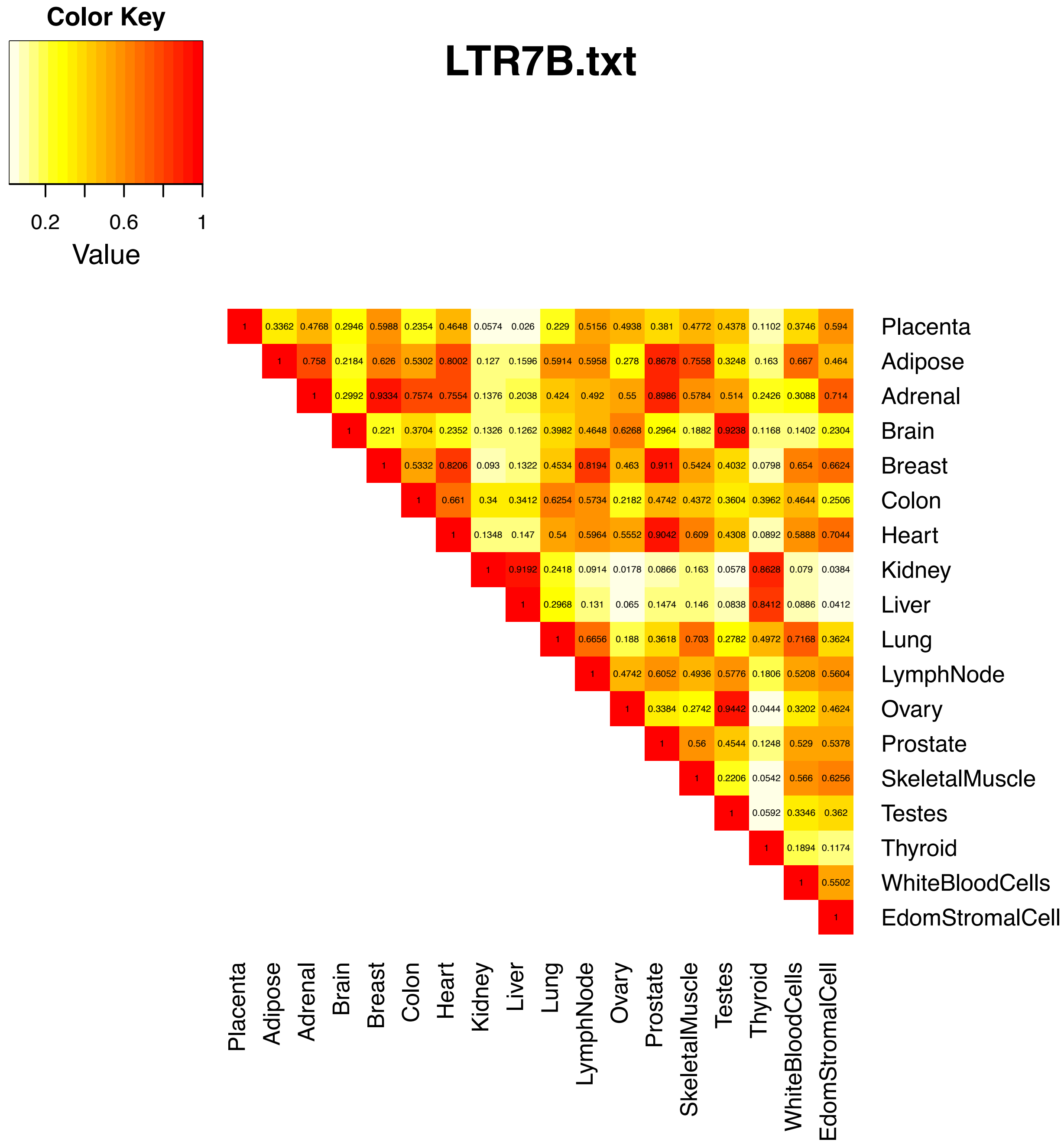
Color Key
0.2
0.6
1
Value
LTR7B.txt
Placenta 1 0.3362 0.4768 0.2946 0.5988 0.2354 0.4648 0.0574 0.026 0.229 0.5156 0.4938 0.381 0.4772 0.4378 0.1102 0.3746 0.594
Adipose 1 0.758 0.2184 0.626 0.5302 0.8002 0.127 0.1596 0.5914 0.5958 0.278 0.8678 0.7558 0.3248 0.163 0.667 0.464
Adrenal 1 0.2992 0.9334 0.7574 0.7554 0.1376 0.2038 0.424 0.492 0.55 0.8986 0.5784 0.514 0.2426 0.3088 0.714
Brain 1 0.221 0.3704 0.2352 0.1326 0.1262 0.3982 0.4648 0.6268 0.2964 0.1882 0.9238 0.1168 0.1402 0.2304
Breast 1 0.5332 0.8206 0.093 0.1322 0.4534 0.8194 0.463 0.911 0.5424 0.4032 0.0798 0.654 0.6624
Colon 1 0.661 0.34 0.3412 0.6254 0.5734 0.2182 0.4742 0.4372 0.3604 0.3962 0.4644 0.2506
Heart 1 0.1348 0.147 0.54 0.5964 0.5552 0.9042 0.609 0.4308 0.0892 0.5888 0.7044
Kidney 1 0.9192 0.2418 0.0914 0.0178 0.0866 0.163 0.0578 0.8628 0.079 0.0384
Liver 1 0.2968 0.131 0.065 0.1474 0.146 0.0838 0.8412 0.0886 0.0412
Lung 1 0.6656 0.188 0.3618 0.703 0.2782 0.4972 0.7168 0.3624
LymphNode 1 0.4742 0.6052 0.4936 0.5776 0.1806 0.5208 0.5604
Ovary 1 0.3384 0.2742 0.9442 0.0444 0.3202 0.4624
Prostate 1 0.56 0.4544 0.1248 0.529 0.5378
SkeletalMuscle 1 0.2206 0.0542 0.566 0.6256
Testes 1 0.0592 0.3346 0.362
Thyroid 1 0.1894 0.1174
WhiteBloodCells 1 0.5502
EdomStromalCell 1
Placenta Adipose Adrenal Brain Breast Colon Heart Kidney Liver Lung LymphNode Ovary Prostate SkeletalMuscle Testes Thyroid WhiteBloodCells EdomStromalCell

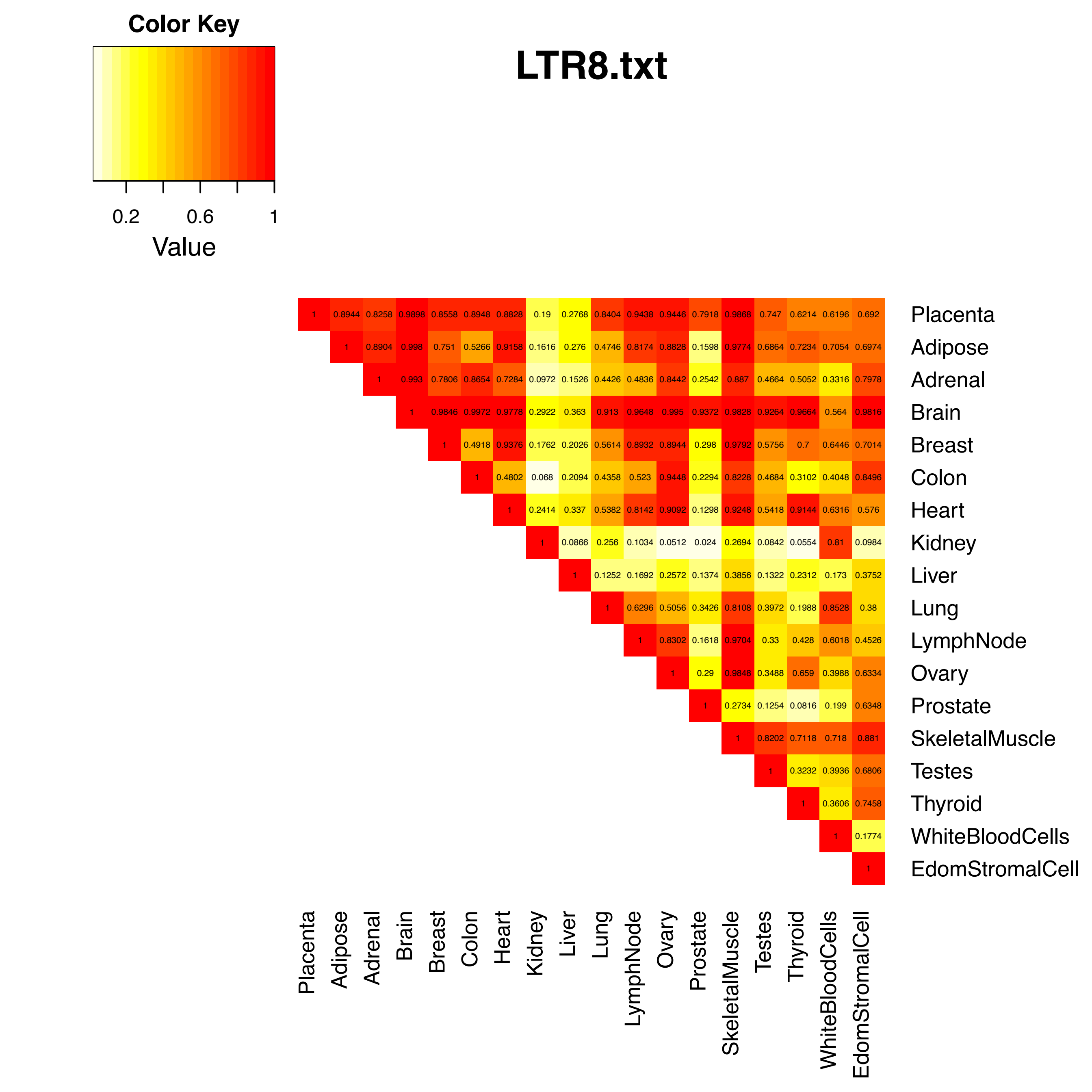
Color Key
0.2 0.6 1
Value
LTR8.txt
Placenta 1 0.8944 0.8258 0.9898 0.8558 0.8948 0.8828 0.19 0.2768 0.8404 0.9438 0.9446 0.7918 0.9868 0.747 0.6214 0.6196 0.692
Adipose 1 0.8904 0.998 0.751 0.5266 0.9158 0.1616 0.276 0.4746 0.8174 0.8828 0.1598 0.9774 0.6864 0.7234 0.7054 0.6974
Adrenal 1 0.993 0.7806 0.8654 0.7284 0.0972 0.1526 0.4426 0.4836 0.8442 0.2542 0.887 0.4664 0.5052 0.3316 0.7978
Brain 1 0.9846 0.9972 0.9778 0.2922 0.363 0.913 0.9648 0.995 0.9372 0.9828 0.9264 0.9664 0.564 0.9816
Breast 1 0.4918 0.9376 0.1762 0.2026 0.5614 0.8932 0.8944 0.298 0.9792 0.5756 0.7 0.6446 0.7014
Colon 1 0.4802 0.068 0.2094 0.4358 0.523 0.9448 0.2294 0.8228 0.4684 0.3102 0.4048 0.8496
Heart 1 0.2414 0.337 0.5382 0.8142 0.9092 0.1298 0.9248 0.5418 0.9144 0.6316 0.576
Kidney 1 0.0866 0.256 0.1034 0.0512 0.024 0.2694 0.0842 0.0554 0.81 0.0984
Liver 1 0.1252 0.1692 0.2572 0.1374 0.3856 0.1322 0.2312 0.173 0.3752
Lung 1 0.6296 0.5056 0.3426 0.8108 0.3972 0.1988 0.8528 0.38
LymphNode 1 0.8302 0.1618 0.9704 0.33 0.428 0.6018 0.4526
Ovary 1 0.29 0.9848 0.3488 0.659 0.3988 0.6334
Prostate 1 0.2734 0.1254 0.0816 0.199 0.6348
SkeletalMuscle 1 0.8202 0.7118 0.718 0.881
Testes 1 0.3232 0.3936 0.6806
Thyroid 1 0.3606 0.7458
WhiteBloodCells 1 0.1774
EdomStromalCell 1
Placenta Adipose Adrenal Brain Breast Colon Heart Kidney Liver Lung LymphNode Ovary Prostate SkeletalMuscle Testes Thyroid WhiteBloodCells EdomStromalCell

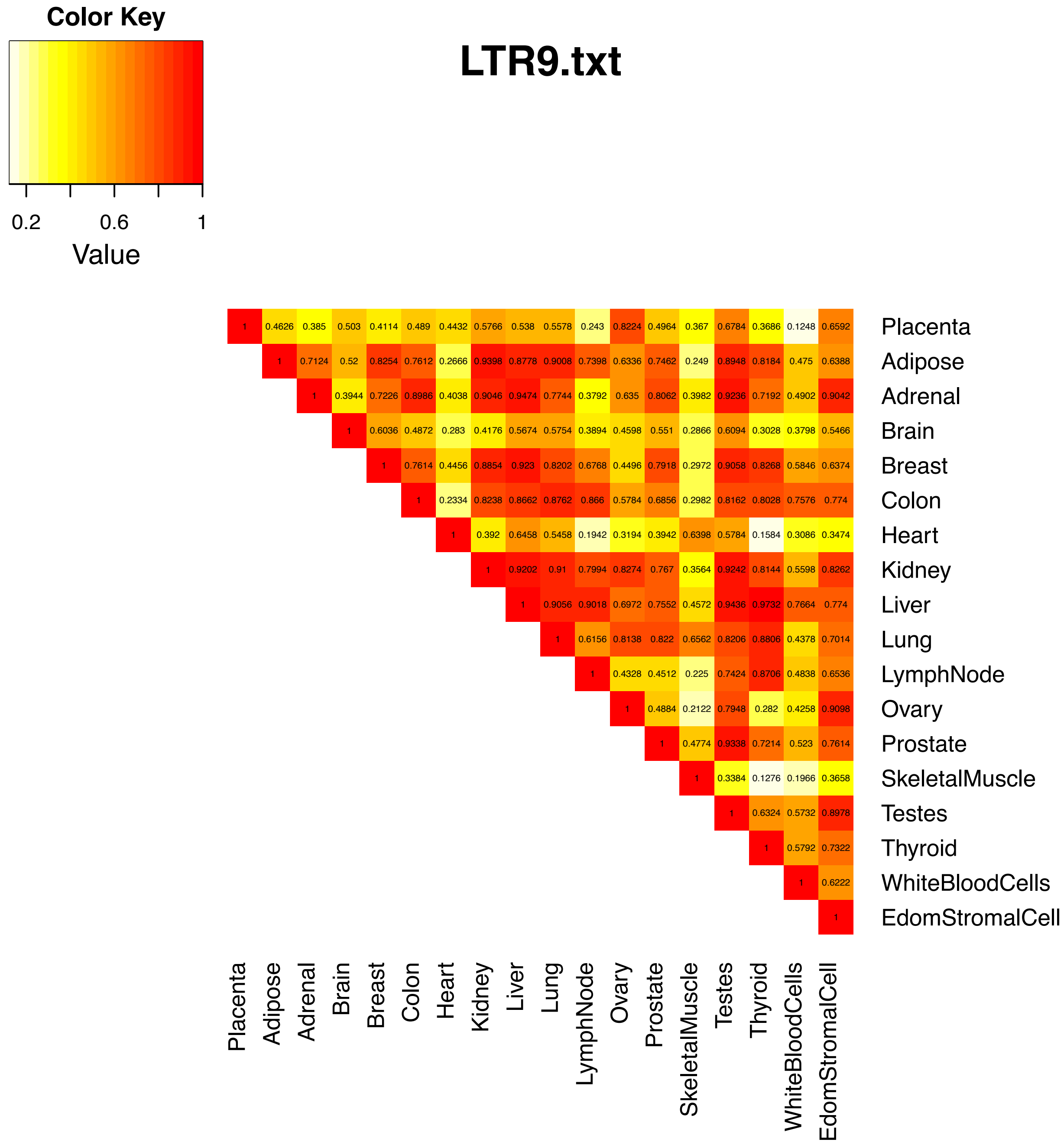
Color Key
0.2
0.6
1
Value
LTR9.txt
1 0.4626 0.385 0.503 0.4114 0.489 0.4432 0.5766 0.538 0.5578 0.243 0.8224 0.4964 0.367 0.6784 0.3686 0.1248 0.6592
1 0.7124 0.52 0.8254 0.7612 0.2666 0.9398 0.8778 0.9008 0.7398 0.6336 0.7462 0.249 0.8948 0.8184 0.475 0.6388
1 0.3944 0.7226 0.8986 0.4038 0.9046 0.9474 0.7744 0.3792 0.635 0.8062 0.3982 0.9236 0.7192 0.4902 0.9042
1 0.6036 0.4872 0.283 0.4176 0.5674 0.5754 0.3894 0.4598 0.551 0.2866 0.6094 0.3028 0.3798 0.5466
1 0.7614 0.4456 0.8854 0.923 0.8202 0.6768 0.4496 0.7918 0.2972 0.9058 0.8268 0.5846 0.6374
1 0.2334 0.8238 0.8662 0.8762 0.866 0.5784 0.6856 0.2982 0.8162 0.8028 0.7576 0.774
1 0.392 0.6458 0.5458 0.1942 0.3194 0.3942 0.6398 0.5784 0.1584 0.3086 0.3474
1 0.9202 0.91 0.7994 0.8274 0.767 0.3564 0.9242 0.8144 0.5598 0.8262
1 0.9056 0.9018 0.6972 0.7552 0.4572 0.9436 0.9732 0.7664 0.774
1 0.6156 0.8138 0.822 0.6562 0.8206 0.8806 0.4378 0.7014
1 0.4328 0.4512 0.225 0.7424 0.8706 0.4838 0.6536
1 0.4884 0.2122 0.7948 0.282 0.4258 0.9098
1 0.4774 0.9338 0.7214 0.523 0.7614
1 0.3384 0.1276 0.1966 0.3658
1 0.6324 0.5732 0.8978
1 0.5792 0.7322
1 0.6222
1
Placenta
Adipose
Adrenal
Brain
Breast
Colon
Heart
Kidney
Liver
Lung
LymphNode
Ovary
Prostate
SkeletalMuscle
Testes
Thyroid
WhiteBloodCells
EdomStromalCell

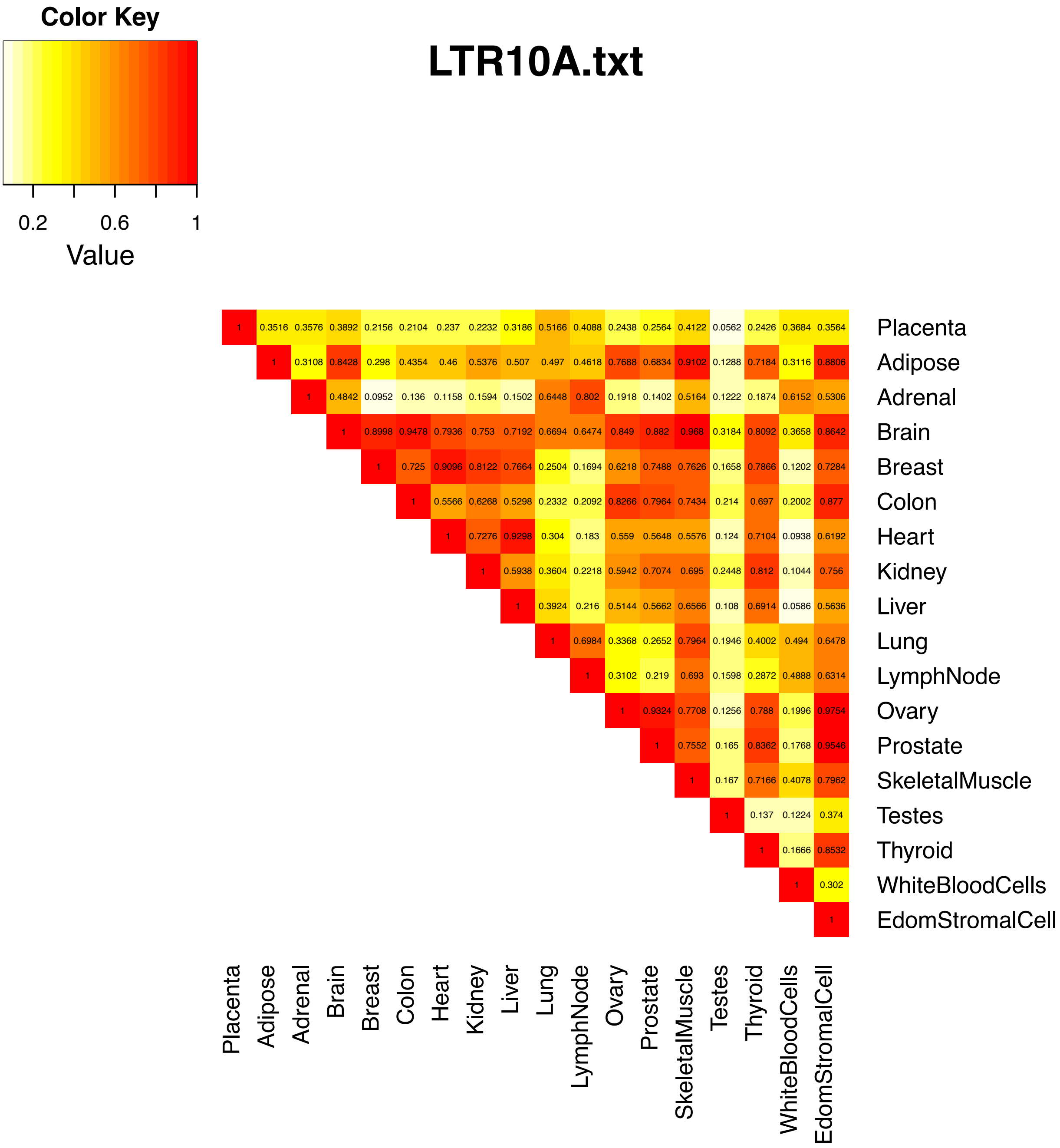
Color Key
0.2
0.6
1
Value
LTR10A.txt
Placenta 1 0.3516 0.3576 0.3892 0.2156 0.2104 0.237 0.2232 0.3186 0.5166 0.4088 0.2438 0.2564 0.4122 0.0562 0.2426 0.3684 0.3564
Adipose 1 0.3108 0.8428 0.298 0.4354 0.46 0.5376 0.507 0.497 0.4618 0.7688 0.6834 0.9102 0.1288 0.7184 0.3116 0.8806
Adrenal 1 0.4842 0.0952 0.136 0.1158 0.1594 0.1502 0.6448 0.802 0.1918 0.1402 0.5164 0.1222 0.1874 0.6152 0.5306
Brain 1 0.8998 0.9478 0.7936 0.753 0.7192 0.6694 0.6474 0.849 0.882 0.968 0.3184 0.8092 0.3658 0.8642
Breast 1 0.725 0.9096 0.8122 0.7664 0.2504 0.1694 0.6218 0.7488 0.7626 0.1658 0.7866 0.1202 0.7284
Colon 1 0.5566 0.6268 0.5298 0.2332 0.2092 0.8266 0.7964 0.7434 0.214 0.697 0.2002 0.877
Heart 1 0.7276 0.9298 0.304 0.183 0.559 0.5648 0.5576 0.124 0.7104 0.0938 0.6192
Kidney 1 0.5938 0.3604 0.2218 0.5942 0.7074 0.695 0.2448 0.812 0.1044 0.756
Liver 1 0.3924 0.216 0.5144 0.5662 0.6566 0.108 0.6914 0.0586 0.5636
Lung 1 0.6984 0.3368 0.2652 0.7964 0.1946 0.4002 0.494 0.6478
LymphNode 1 0.3102 0.219 0.693 0.1598 0.2872 0.4888 0.6314
Ovary 1 0.9324 0.7708 0.1256 0.788 0.1996 0.9754
Prostate 1 0.7552 0.165 0.8362 0.1768 0.9546
SkeletalMuscle 1 0.167 0.7166 0.4078 0.7962
Testes 1 0.137 0.1224 0.374
Thyroid 1 0.1666 0.8532
WhiteBloodCells 1 0.302
EdomStromalCell 1
Placenta
Adipose
Adrenal
Brain
Breast
Colon
Heart
Kidney
Liver
Lung
LymphNode
Ovary
Prostate
SkeletalMuscle
Testes
Thyroid
WhiteBloodCells
EdomStromalCell

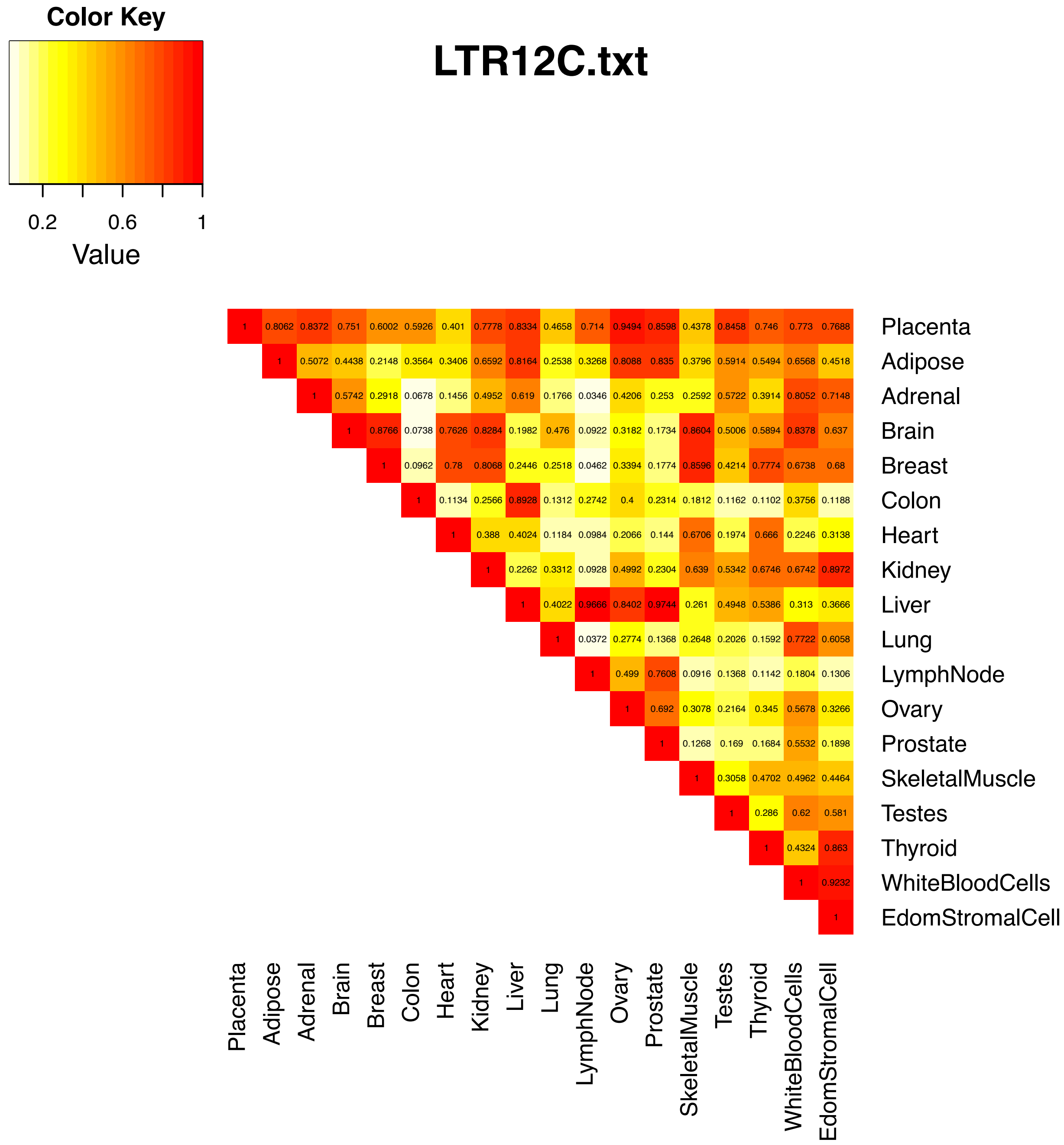
Color Key
0.2
0.6
1
Value
LTR12C.txt
Placenta 1 0.8062 0.8372 0.751 0.6002 0.5926 0.401 0.7778 0.8334 0.4658 0.714 0.9494 0.8598 0.4378 0.8458 0.746 0.773 0.7688
Adipose 1 0.5072 0.4438 0.2148 0.3564 0.3406 0.6592 0.8164 0.2538 0.3268 0.8088 0.835 0.3796 0.5914 0.5494 0.6568 0.4518
Adrenal 1 0.5742 0.2918 0.0678 0.1456 0.4952 0.619 0.1766 0.0346 0.4206 0.253 0.2592 0.5722 0.3914 0.8052 0.7148
Brain 1 0.8766 0.0738 0.7626 0.8284 0.1982 0.476 0.0922 0.3182 0.1734 0.8604 0.5006 0.5894 0.8378 0.637
Breast 1 0.0962 0.78 0.8068 0.2446 0.2518 0.0462 0.3394 0.1774 0.8596 0.4214 0.7774 0.6738 0.68
Colon 1 0.1134 0.2566 0.8928 0.1312 0.2742 0.4 0.2314 0.1812 0.1162 0.1102 0.3756 0.1188
Heart 1 0.388 0.4024 0.1184 0.0984 0.2066 0.144 0.6706 0.1974 0.666 0.2246 0.3138
Kidney 1 0.2262 0.3312 0.0928 0.4992 0.2304 0.639 0.5342 0.6746 0.6742 0.8972
Liver 1 0.4022 0.9666 0.8402 0.9744 0.261 0.4948 0.5386 0.313 0.3666
Lung 1 0.0372 0.2774 0.1368 0.2648 0.2026 0.1592 0.7722 0.6058
LymphNode 1 0.499 0.7608 0.0916 0.1368 0.1142 0.1804 0.1306
Ovary 1 0.692 0.3078 0.2164 0.345 0.5678 0.3266
Prostate 1 0.1268 0.169 0.1684 0.5532 0.1898
SkeletalMuscle 1 0.3058 0.4702 0.4962 0.4464
Testes 1 0.286 0.62 0.581
Thyroid 1 0.4324 0.863
WhiteBloodCells 1 0.9232
EdomStromalCell 1
Placenta
Adipose
Adrenal
Brain
Breast
Colon
Heart
Kidney
Liver
Lung
LymphNode
Ovary
Prostate
SkeletalMuscle
Testes
Thyroid
WhiteBloodCells
EdomStromalCell

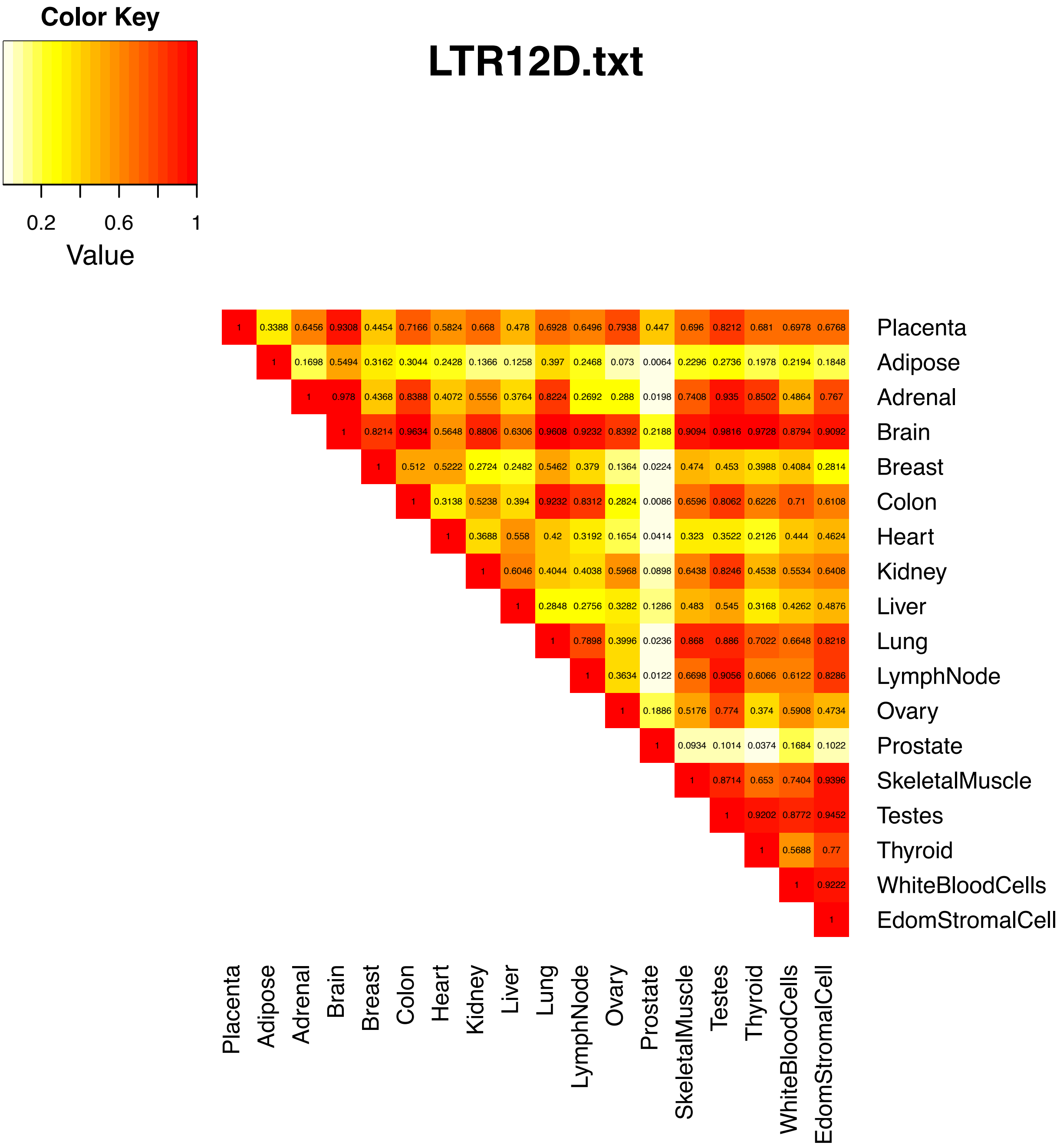
Color Key
0.2
0.6
1
Value
LTR12D.txt
Placenta: 1 0.3388 0.6456 0.9308 0.4454 0.7166 0.5824 0.668 0.478 0.6928 0.6496 0.7938 0.447 0.696 0.8212 0.681 0.6978 0.6768
Adipose: 1 0.1698 0.5494 0.3162 0.3044 0.2428 0.1366 0.1258 0.397 0.2468 0.073 0.0064 0.2296 0.2736 0.1978 0.2194 0.1848
Adrenal: 1 0.978 0.4368 0.8388 0.4072 0.5556 0.3764 0.8224 0.2692 0.288 0.0198 0.7408 0.935 0.8502 0.4864 0.767
Brain: 1 0.8214 0.9634 0.5648 0.8806 0.6306 0.9608 0.9232 0.8392 0.2188 0.9094 0.9816 0.9728 0.8794 0.9092
Breast: 1 0.512 0.5222 0.2724 0.2482 0.5462 0.379 0.1364 0.0224 0.474 0.453 0.3988 0.4084 0.2814
Colon: 1 0.3138 0.5238 0.394 0.9232 0.8312 0.2824 0.0086 0.6596 0.8062 0.6226 0.71 0.6108
Heart: 1 0.3688 0.558 0.42 0.3192 0.1654 0.0414 0.323 0.3522 0.2126 0.444 0.4624
Kidney: 1 0.6046 0.4044 0.4038 0.5968 0.0898 0.6438 0.8246 0.4538 0.5534 0.6408
Liver: 1 0.2848 0.2756 0.3282 0.1286 0.483 0.545 0.3168 0.4262 0.4876
Lung: 1 0.7898 0.3996 0.0236 0.868 0.886 0.7022 0.6648 0.8218
LymphNode: 1 0.3634 0.0122 0.6698 0.9056 0.6066 0.6122 0.8286
Ovary: 1 0.1886 0.5176 0.774 0.374 0.5908 0.4734
Prostate: 1 0.0934 0.1014 0.0374 0.1684 0.1022
SkeletalMuscle: 1 0.8714 0.653 0.7404 0.9396
Testes: 1 0.9202 0.8772 0.9452
Thyroid: 1 0.5688 0.77
WhiteBloodCells: 1 0.9222
EdomStromalCell: 1
Placenta Adipose Adrenal Brain Breast Colon Heart Kidney Liver Lung LymphNode Ovary Prostate SkeletalMuscle Testes Thyroid WhiteBloodCells EdomStromalCell

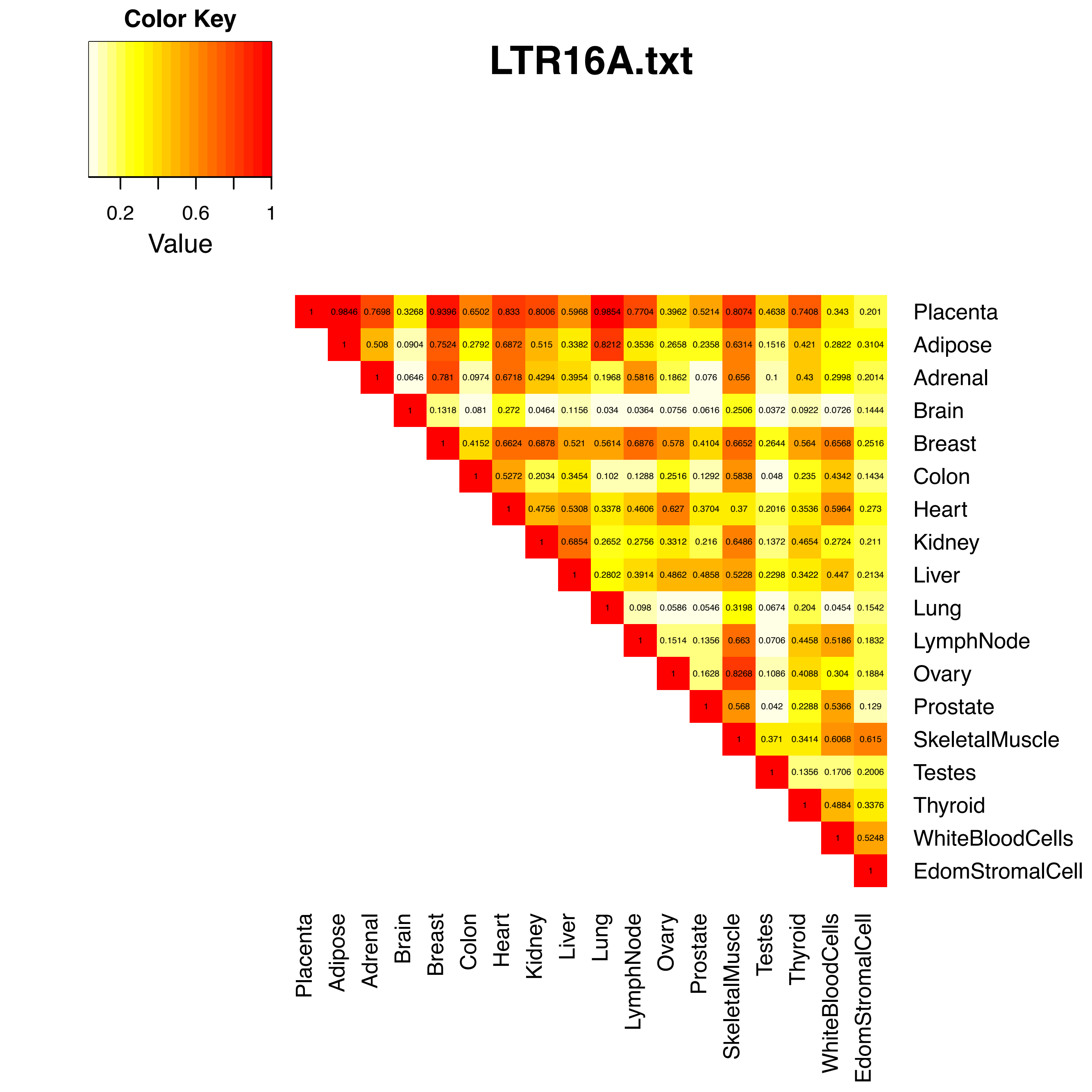
Color Key
0.2
0.6
1
Value
LTR16A.txt
Placenta: 1 0.9846 0.7698 0.3268 0.9396 0.6502 0.833 0.8006 0.5968 0.9854 0.7704 0.3962 0.5214 0.8074 0.4638 0.7408 0.343 0.201
Adipose: 1 0.508 0.0904 0.7524 0.2792 0.6872 0.515 0.3382 0.8212 0.3536 0.2658 0.2358 0.6314 0.1516 0.421 0.2822 0.3104
Adrenal: 1 0.0646 0.781 0.0974 0.6718 0.4294 0.3954 0.1968 0.5816 0.1862 0.076 0.656 0.1 0.43 0.2998 0.2014
Brain: 1 0.1318 0.081 0.272 0.0464 0.1156 0.034 0.0364 0.0756 0.0616 0.2506 0.0372 0.0922 0.0726 0.1444
Breast: 1 0.4152 0.6624 0.6878 0.521 0.5614 0.6876 0.578 0.4104 0.6652 0.2644 0.564 0.6568 0.2516
Colon: 1 0.5272 0.2034 0.3454 0.102 0.1288 0.2516 0.1292 0.5838 0.048 0.235 0.4342 0.1434
Heart: 1 0.4756 0.5308 0.3378 0.4606 0.627 0.3704 0.37 0.2016 0.3536 0.5964 0.273
Kidney: 1 0.6854 0.2652 0.2756 0.3312 0.216 0.6486 0.1372 0.4654 0.2724 0.211
Liver: 1 0.2802 0.3914 0.4862 0.4858 0.5228 0.2298 0.3422 0.447 0.2134
Lung: 1 0.098 0.0586 0.0546 0.3198 0.0674 0.204 0.0454 0.1542
LymphNode: 1 0.1514 0.1356 0.663 0.0706 0.4458 0.5186 0.1832
Ovary: 1 0.1628 0.8268 0.1086 0.4088 0.304 0.1884
Prostate: 1 0.568 0.042 0.2288 0.5366 0.129
SkeletalMuscle: 1 0.371 0.3414 0.6068 0.615
Testes: 1 0.1356 0.1706 0.2006
Thyroid: 1 0.4884 0.3376
WhiteBloodCells: 1 0.5248
EdomStromalCell: 1
Placenta Adipose Adrenal Brain Breast Colon Heart Kidney Liver Lung LymphNode Ovary Prostate SkeletalMuscle Testes Thyroid WhiteBloodCells EdomStromalCell

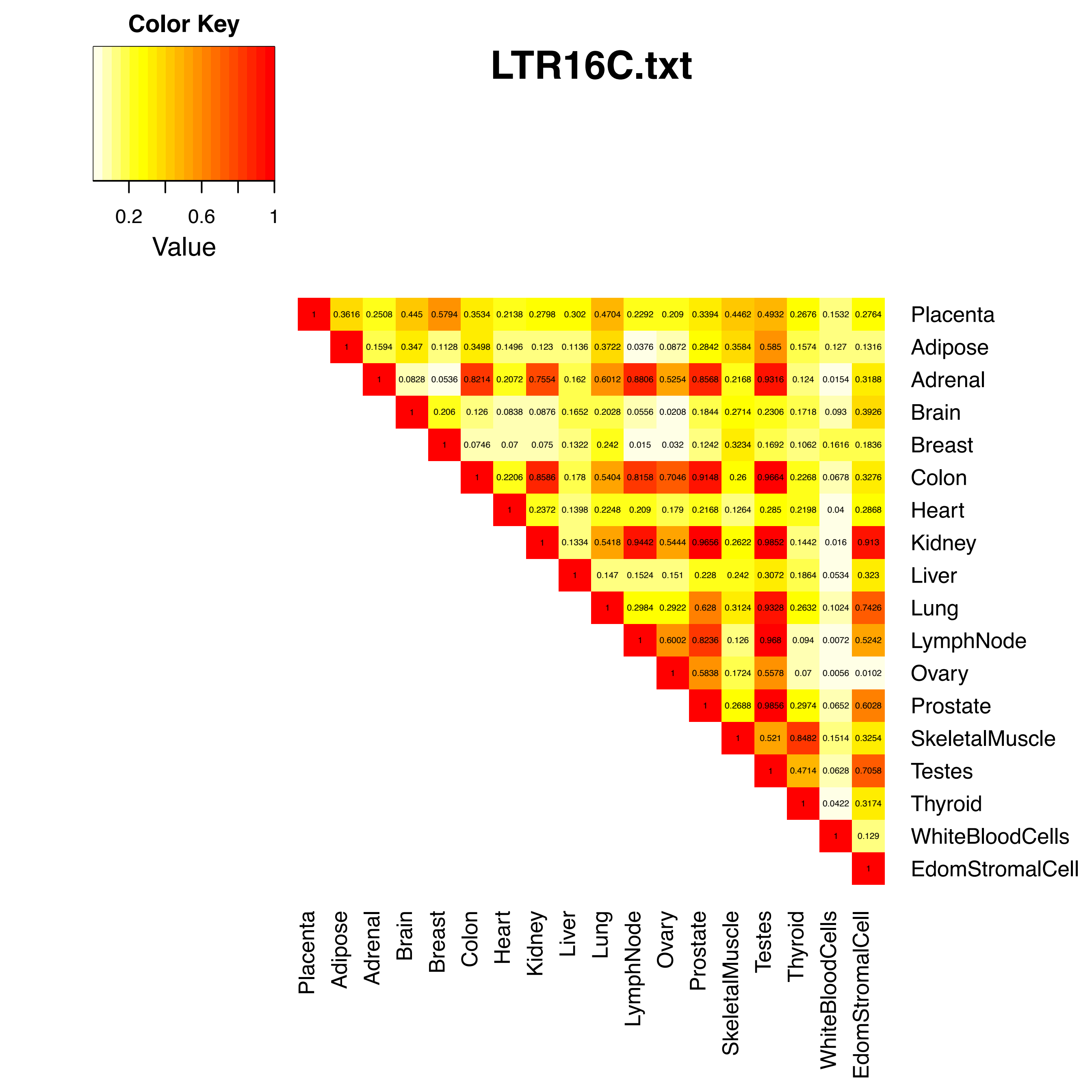
Color Key
0.2 0.6 1
Value
LTR16C.txt
Placenta: 1 0.3616 0.2508 0.445 0.5794 0.3534 0.2138 0.2798 0.302 0.4704 0.2292 0.209 0.3394 0.4462 0.4932 0.2676 0.1532 0.2764
Adipose: 1 0.1594 0.347 0.1128 0.3498 0.1496 0.123 0.1136 0.3722 0.0376 0.0872 0.2842 0.3584 0.585 0.1574 0.127 0.1316
Adrenal: 1 0.0828 0.0536 0.8214 0.2072 0.7554 0.162 0.6012 0.8806 0.5254 0.8568 0.2168 0.9316 0.124 0.0154 0.3188
Brain: 1 0.206 0.126 0.0838 0.0876 0.1652 0.2028 0.0556 0.0208 0.1844 0.2714 0.2306 0.1718 0.093 0.3926
Breast: 1 0.0746 0.07 0.075 0.1322 0.242 0.015 0.032 0.1242 0.3234 0.1692 0.1062 0.1616 0.1836
Colon: 1 0.2206 0.8586 0.178 0.5404 0.8158 0.7046 0.9148 0.26 0.9664 0.2268 0.0678 0.3276
Heart: 1 0.2372 0.1398 0.2248 0.209 0.179 0.2168 0.1264 0.285 0.2198 0.04 0.2868
Kidney: 1 0.1334 0.5418 0.9442 0.5444 0.9656 0.2622 0.9852 0.1442 0.016 0.913
Liver: 1 0.147 0.1524 0.151 0.228 0.242 0.3072 0.1864 0.0534 0.323
Lung: 1 0.2984 0.2922 0.628 0.3124 0.9328 0.2632 0.1024 0.7426
LymphNode: 1 0.6002 0.8236 0.126 0.968 0.094 0.0072 0.5242
Ovary: 1 0.5838 0.1724 0.5578 0.07 0.0056 0.0102
Prostate: 1 0.2688 0.9856 0.2974 0.0652 0.6028
SkeletalMuscle: 1 0.521 0.8482 0.1514 0.3254
Testes: 1 0.4714 0.0628 0.7058
Thyroid: 1 0.0422 0.3174
WhiteBloodCells: 1 0.129
EdomStromalCell: 1
Placenta Adipose Adrenal Brain Breast Colon Heart Kidney Liver Lung LymphNode Ovary Prostate SkeletalMuscle Testes Thyroid WhiteBloodCells EdomStromalCell

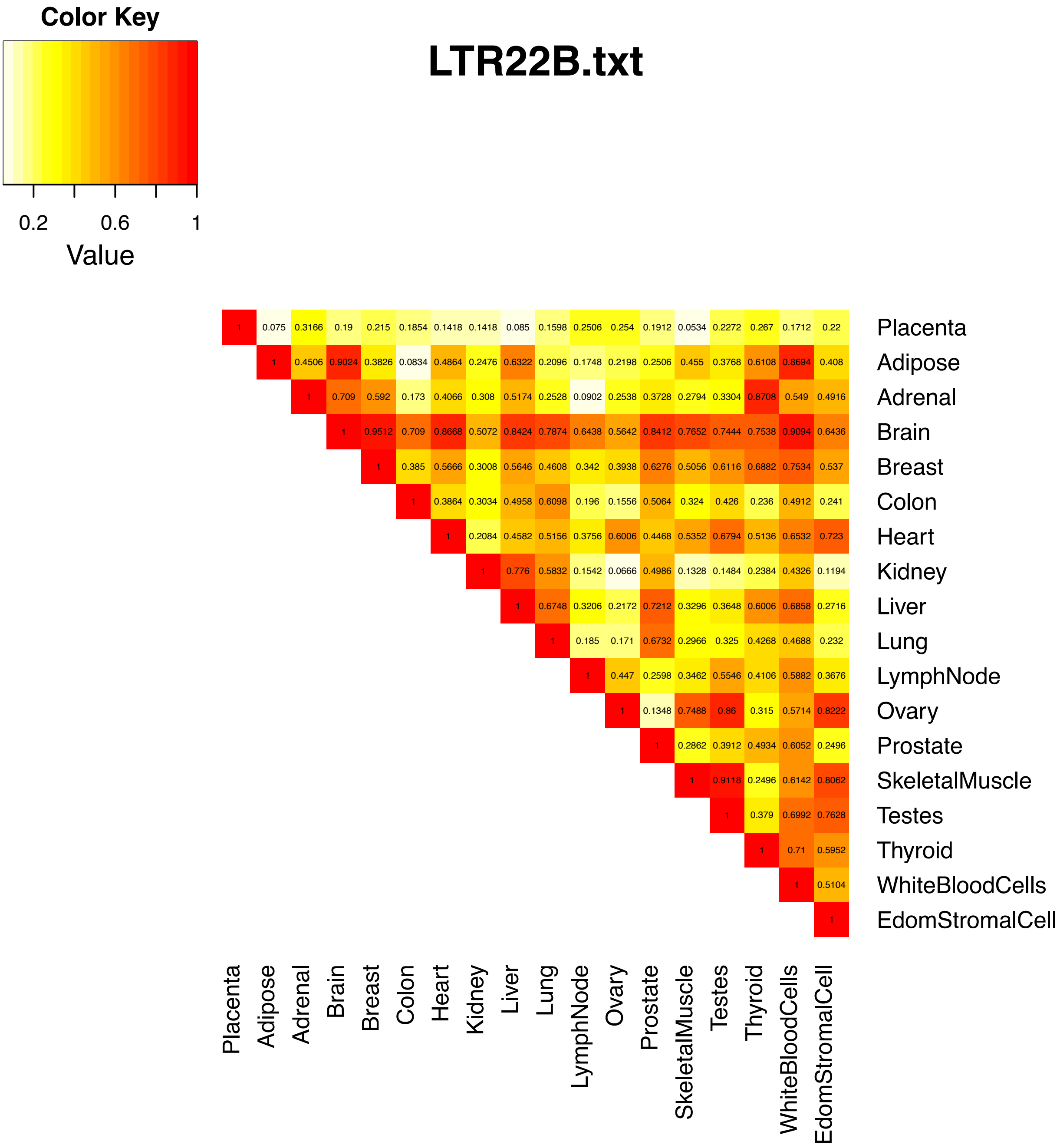
LTR22B.txt
Color Key
0.2 0.6 1
Value
Placenta 1 0.075 0.3166 0.19 0.215 0.1854 0.1418 0.1418 0.085 0.1598 0.2506 0.254 0.1912 0.0534 0.2272 0.267 0.1712 0.22
Adipose 1 0.4506 0.9024 0.3826 0.0834 0.4864 0.2476 0.6322 0.2096 0.1748 0.2198 0.2506 0.455 0.3768 0.6108 0.8694 0.408
Adrenal 1 0.709 0.592 0.173 0.4066 0.308 0.5174 0.2528 0.0902 0.2538 0.3728 0.2794 0.3304 0.8708 0.549 0.4916
Brain 1 0.9512 0.709 0.8668 0.5072 0.8424 0.7874 0.6438 0.5642 0.8412 0.7652 0.7444 0.7538 0.9094 0.6436
Breast 1 0.385 0.5666 0.3008 0.5646 0.4608 0.342 0.3938 0.6276 0.5056 0.6116 0.6882 0.7534 0.537
Colon 1 0.3864 0.3034 0.4958 0.6098 0.196 0.1556 0.5064 0.324 0.426 0.236 0.4912 0.241
Heart 1 0.2084 0.4582 0.5156 0.3756 0.6006 0.4468 0.5352 0.6794 0.5136 0.6532 0.723
Kidney 1 0.776 0.5832 0.1542 0.0666 0.4986 0.1328 0.1484 0.2384 0.4326 0.1194
Liver 1 0.6748 0.3206 0.2172 0.7212 0.3296 0.3648 0.6006 0.6858 0.2716
Lung 1 0.185 0.171 0.6732 0.2966 0.325 0.4268 0.4688 0.232
LymphNode 1 0.447 0.2598 0.3462 0.5546 0.4106 0.5882 0.3676
Ovary 1 0.1348 0.7488 0.86 0.315 0.5714 0.8222
Prostate 1 0.2862 0.3912 0.4934 0.6052 0.2496
SkeletalMuscle 1 0.9118 0.2496 0.6142 0.8062
Testes 1 0.379 0.6992 0.7628
Thyroid 1 0.71 0.5952
WhiteBloodCells 1 0.5104
EdomStromalCell 1
Placenta Adipose Adrenal Brain Breast Colon Heart Kidney Liver Lung LymphNode Ovary Prostate SkeletalMuscle Testes Thyroid WhiteBloodCells EdomStromalCell

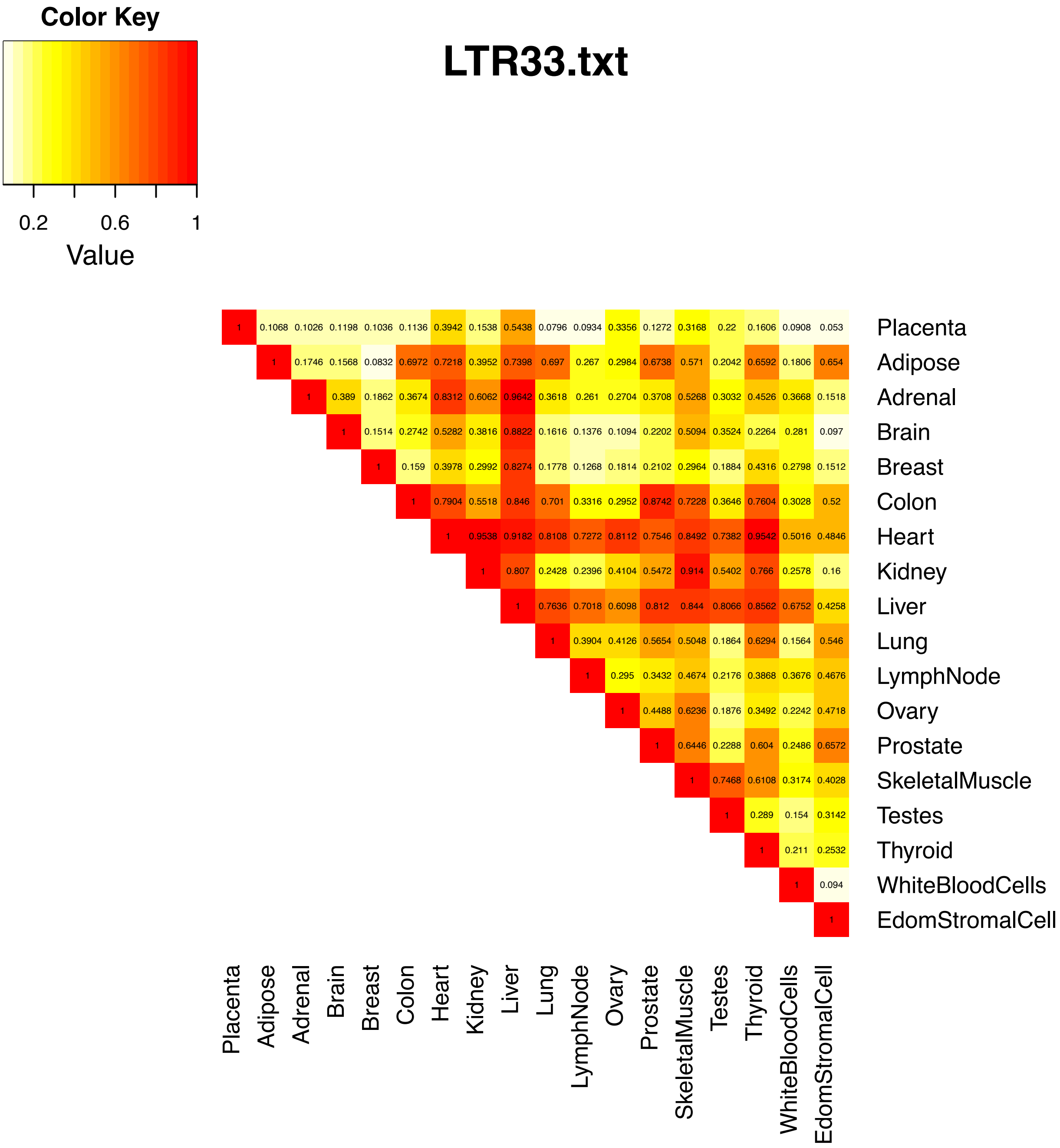
Color Key
0.2
0.6
1
Value
LTR33.txt
Placenta 1 0.1068 0.1026 0.1198 0.1036 0.1136 0.3942 0.1538 0.5438 0.0796 0.0934 0.3356 0.1272 0.3168 0.22 0.1606 0.0908 0.053
Adipose 1 0.1746 0.1568 0.0832 0.6972 0.7218 0.3952 0.7398 0.697 0.267 0.2984 0.6738 0.571 0.2042 0.6592 0.1806 0.654
Adrenal 1 0.389 0.1862 0.3674 0.8312 0.6062 0.9642 0.3618 0.261 0.2704 0.3708 0.5268 0.3032 0.4526 0.3668 0.1518
Brain 1 0.1514 0.2742 0.5282 0.3816 0.8822 0.1616 0.1376 0.1094 0.2202 0.5094 0.3524 0.2264 0.281 0.097
Breast 1 0.159 0.3978 0.2992 0.8274 0.1778 0.1268 0.1814 0.2102 0.2964 0.1884 0.4316 0.2798 0.1512
Colon 1 0.7904 0.5518 0.846 0.701 0.3316 0.2952 0.8742 0.7228 0.3646 0.7604 0.3028 0.52
Heart 1 0.9538 0.9182 0.8108 0.7272 0.8112 0.7546 0.8492 0.7382 0.9542 0.5016 0.4846
Kidney 1 0.807 0.2428 0.2396 0.4104 0.5472 0.914 0.5402 0.766 0.2578 0.16
Liver 1 0.7636 0.7018 0.6098 0.812 0.844 0.8066 0.8562 0.6752 0.4258
Lung 1 0.3904 0.4126 0.5654 0.5048 0.1864 0.6294 0.1564 0.546
LymphNode 1 0.295 0.3432 0.4674 0.2176 0.3868 0.3676 0.4676
Ovary 1 0.4488 0.6236 0.1876 0.3492 0.2242 0.4718
Prostate 1 0.6446 0.2288 0.604 0.2486 0.6572
SkeletalMuscle 1 0.7468 0.6108 0.3174 0.4028
Testes 1 0.289 0.154 0.3142
Thyroid 1 0.211 0.2532
WhiteBloodCells 1 0.094
EdomStromalCell 1
Placenta
Adipose
Adrenal
Brain
Breast
Colon
Heart
Kidney
Liver
Lung
LymphNode
Ovary
Prostate
SkeletalMuscle
Testes
Thyroid
WhiteBloodCells
EdomStromalCell

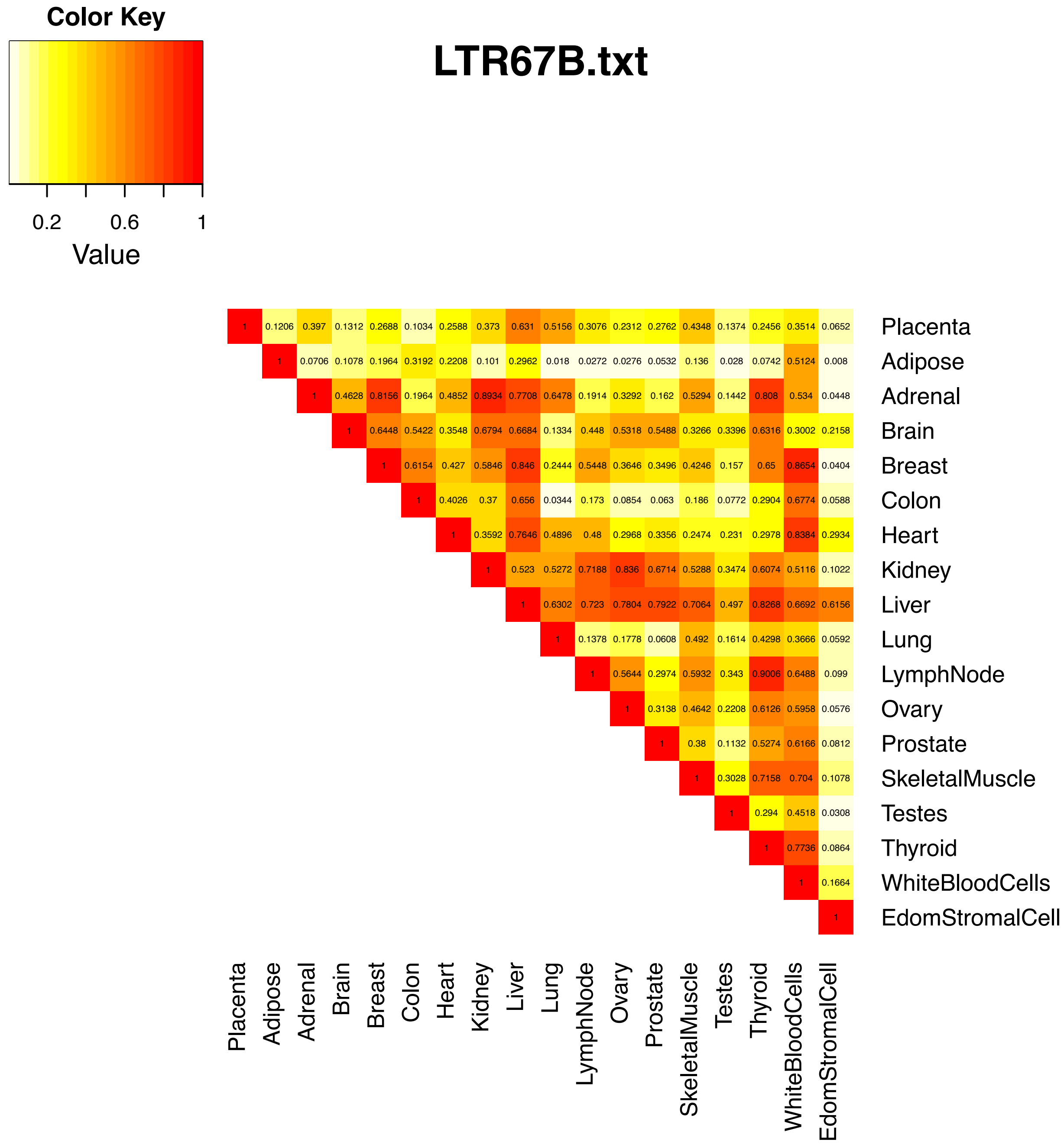
Color Key
0.2 0.6 1
Value
LTR67B.txt
Placenta 1 0.1206 0.397 0.1312 0.2688 0.1034 0.2588 0.373 0.631 0.5156 0.3076 0.2312 0.2762 0.4348 0.1374 0.2456 0.3514 0.0652
Adipose 1 0.0706 0.1078 0.1964 0.3192 0.2208 0.101 0.2962 0.018 0.0272 0.0276 0.0532 0.136 0.028 0.0742 0.5124 0.008
Adrenal 1 0.4628 0.8156 0.1964 0.4852 0.8934 0.7708 0.6478 0.1914 0.3292 0.162 0.5294 0.1442 0.808 0.534 0.0448
Brain 1 0.6448 0.5422 0.3548 0.6794 0.6684 0.1334 0.448 0.5318 0.5488 0.3266 0.3396 0.6316 0.3002 0.2158
Breast 1 0.6154 0.427 0.5846 0.846 0.2444 0.5448 0.3646 0.3496 0.4246 0.157 0.65 0.8654 0.0404
Colon 1 0.4026 0.37 0.656 0.0344 0.173 0.0854 0.063 0.186 0.0772 0.2904 0.6774 0.0588
Heart 1 0.3592 0.7646 0.4896 0.48 0.2968 0.3356 0.2474 0.231 0.2978 0.8384 0.2934
Kidney 1 0.523 0.5272 0.7188 0.836 0.6714 0.5288 0.3474 0.6074 0.5116 0.1022
Liver 1 0.6302 0.723 0.7804 0.7922 0.7064 0.497 0.8268 0.6692 0.6156
Lung 1 0.1378 0.1778 0.0608 0.492 0.1614 0.4298 0.3666 0.0592
LymphNode 1 0.5644 0.2974 0.5932 0.343 0.9006 0.6488 0.099
Ovary 1 0.3138 0.4642 0.2208 0.6126 0.5958 0.0576
Prostate 1 0.38 0.1132 0.5274 0.6166 0.0812
SkeletalMuscle 1 0.3028 0.7158 0.704 0.1078
Testes 1 0.294 0.4518 0.0308
Thyroid 1 0.7736 0.0864
WhiteBloodCells 1 0.1664
EdomStromalCell 1
Placenta Adipose Adrenal Brain Breast Colon Heart Kidney Liver Lung LymphNode Ovary Prostate SkeletalMuscle Testes Thyroid WhiteBloodCells EdomStromalCell

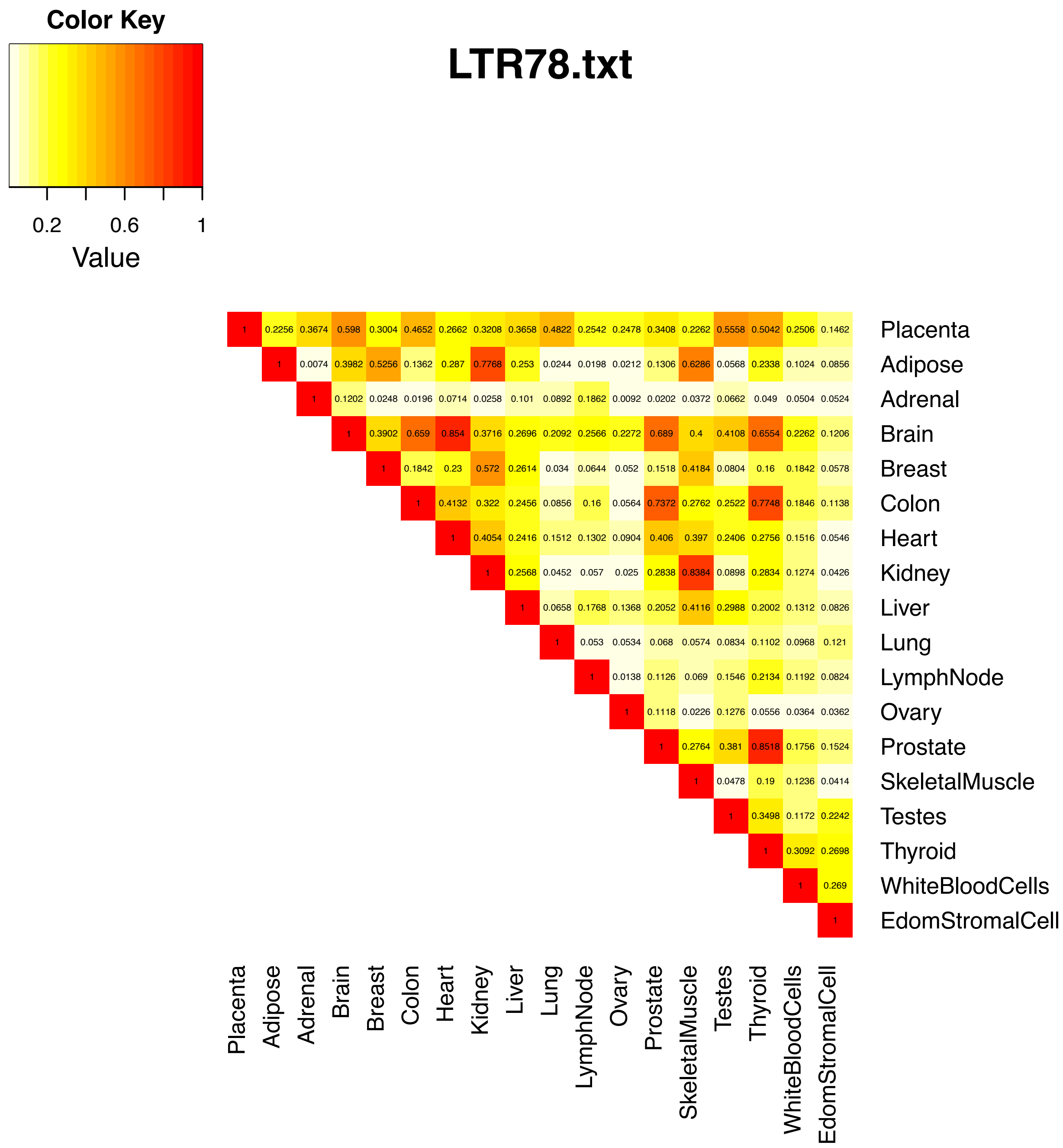
Color Key
0.2
0.6
1
Value
LTR78.txt
Placenta 1 0.2256 0.3674 0.598 0.3004 0.4652 0.2662 0.3208 0.3658 0.4822 0.2542 0.2478 0.3408 0.2262 0.5558 0.5042 0.2506 0.1462
Adipose 1 0.0074 0.3982 0.5256 0.1362 0.287 0.7768 0.253 0.0244 0.0198 0.0212 0.1306 0.6286 0.0568 0.2338 0.1024 0.0856
Adrenal 1 0.1202 0.0248 0.0196 0.0714 0.0258 0.101 0.0892 0.1862 0.0092 0.0202 0.0372 0.0662 0.049 0.0504 0.0524
Brain 1 0.3902 0.659 0.854 0.3716 0.2696 0.2092 0.2566 0.2272 0.689 0.4 0.4108 0.6554 0.2262 0.1206
Breast 1 0.1842 0.23 0.572 0.2614 0.034 0.0644 0.052 0.1518 0.4184 0.0804 0.16 0.1842 0.0578
Colon 1 0.4132 0.322 0.2456 0.0856 0.16 0.0564 0.7372 0.2762 0.2522 0.7748 0.1846 0.1138
Heart 1 0.4054 0.2416 0.1512 0.1302 0.0904 0.406 0.397 0.2406 0.2756 0.1516 0.0546
Kidney 1 0.2568 0.0452 0.057 0.025 0.2838 0.8384 0.0898 0.2834 0.1274 0.0426
Liver 1 0.0658 0.1768 0.1368 0.2052 0.4116 0.2988 0.2002 0.1312 0.0826
Lung 1 0.053 0.0534 0.068 0.0574 0.0834 0.1102 0.0968 0.121
LymphNode 1 0.0138 0.1126 0.069 0.1546 0.2134 0.1192 0.0824
Ovary 1 0.1118 0.0226 0.1276 0.0556 0.0364 0.0362
Prostate 1 0.2764 0.381 0.8518 0.1756 0.1524
SkeletalMuscle 1 0.0478 0.19 0.1236 0.0414
Testes 1 0.3498 0.1172 0.2242
Thyroid 1 0.3092 0.2698
WhiteBloodCells 1 0.269
EdomStromalCell 1
Placenta
Adipose
Adrenal
Brain
Breast
Colon
Heart
Kidney
Liver
Lung
LymphNode
Ovary
Prostate
SkeletalMuscle
Testes
Thyroid
WhiteBloodCells
EdomStromalCell

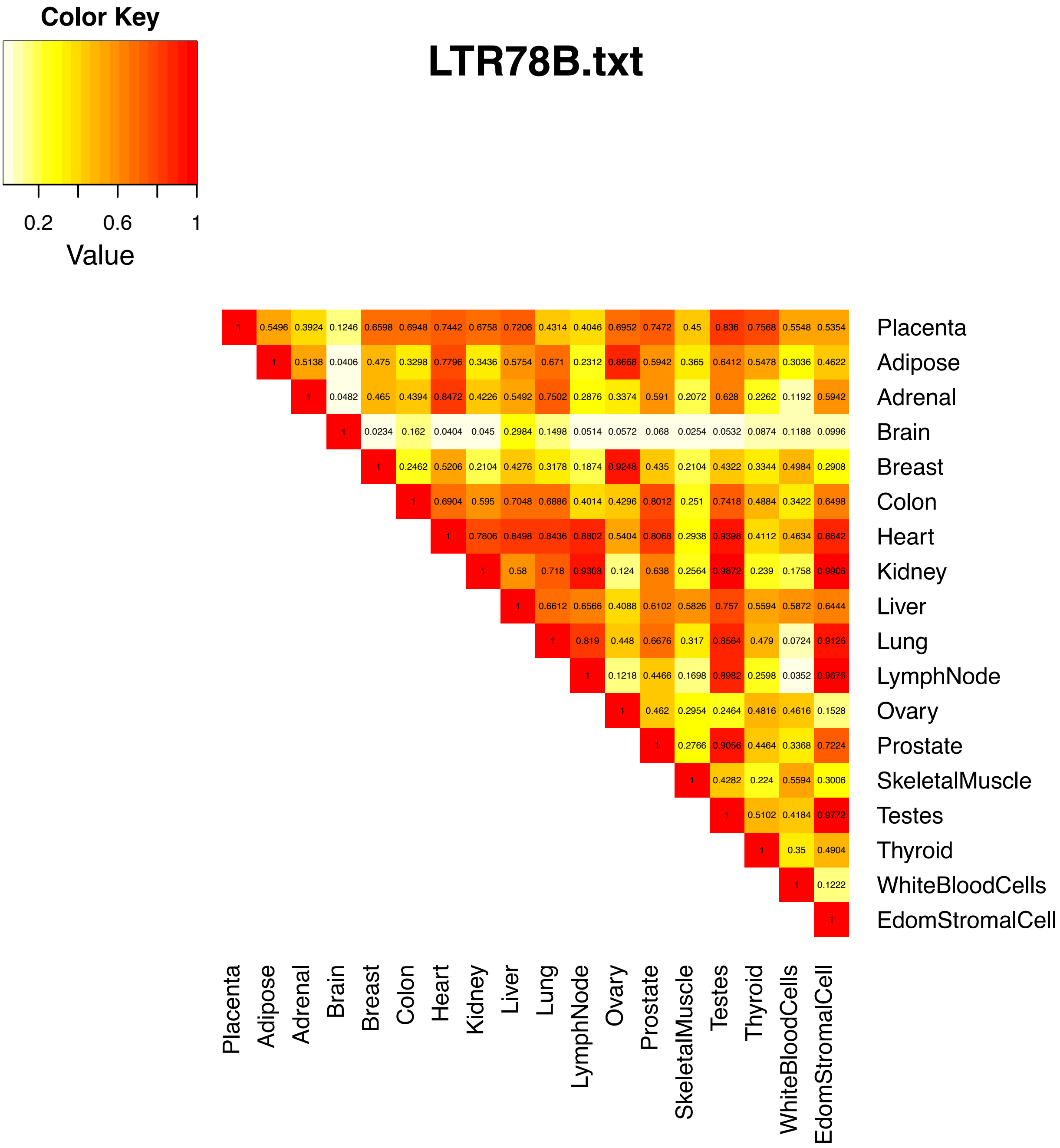

LTR78B.txt
Color Key
0.2
0.6
1
Value
Placenta 1 0.5496 0.3924 0.1246 0.6598 0.6948 0.7442 0.6758 0.7206 0.4314 0.4046 0.6952 0.7472 0.45 0.836 0.7568 0.5548 0.5354
Adipose 1 0.5138 0.0406 0.475 0.3298 0.7796 0.3436 0.5754 0.671 0.2312 0.8668 0.5942 0.365 0.6412 0.5478 0.3036 0.4622
Adrenal 1 0.0482 0.465 0.4394 0.8472 0.4226 0.5492 0.7502 0.2876 0.3374 0.591 0.2072 0.628 0.2262 0.1192 0.5942
Brain 1 0.0234 0.162 0.0404 0.045 0.2984 0.1498 0.0514 0.0572 0.068 0.0254 0.0532 0.0874 0.1188 0.0996
Breast 1 0.2462 0.5206 0.2104 0.4276 0.3178 0.1874 0.9248 0.435 0.2104 0.4322 0.3344 0.4984 0.2908
Colon 1 0.6904 0.595 0.7048 0.6886 0.4014 0.4296 0.8012 0.251 0.7418 0.4884 0.3422 0.6498
Heart 1 0.7806 0.8498 0.8436 0.8802 0.5404 0.8068 0.2938 0.9398 0.4112 0.4634 0.8642
Kidney 1 0.58 0.718 0.9308 0.124 0.638 0.2564 0.9872 0.239 0.1758 0.9906
Liver 1 0.6612 0.6566 0.4088 0.6102 0.5826 0.757 0.5594 0.5872 0.6444
Lung 1 0.819 0.448 0.6676 0.317 0.8564 0.479 0.0724 0.9126
LymphNode 1 0.1218 0.4466 0.1698 0.8982 0.2598 0.0352 0.9676
Ovary 1 0.462 0.2954 0.2464 0.4816 0.4616 0.1528
Prostate 1 0.2766 0.9056 0.4464 0.3368 0.7224
SkeletalMuscle 1 0.4282 0.224 0.5594 0.3006
Testes 1 0.5102 0.4184 0.9772
Thyroid 1 0.35 0.4904
WhiteBloodCells 1 0.1222
EdomStromalCell 1
Placenta
Adipose
Adrenal
Brain
Breast
Colon
Heart
Kidney
Liver
Lung
LymphNode
Ovary
Prostate
SkeletalMuscle
Testes
Thyroid
WhiteBloodCells
EdomStromalCell

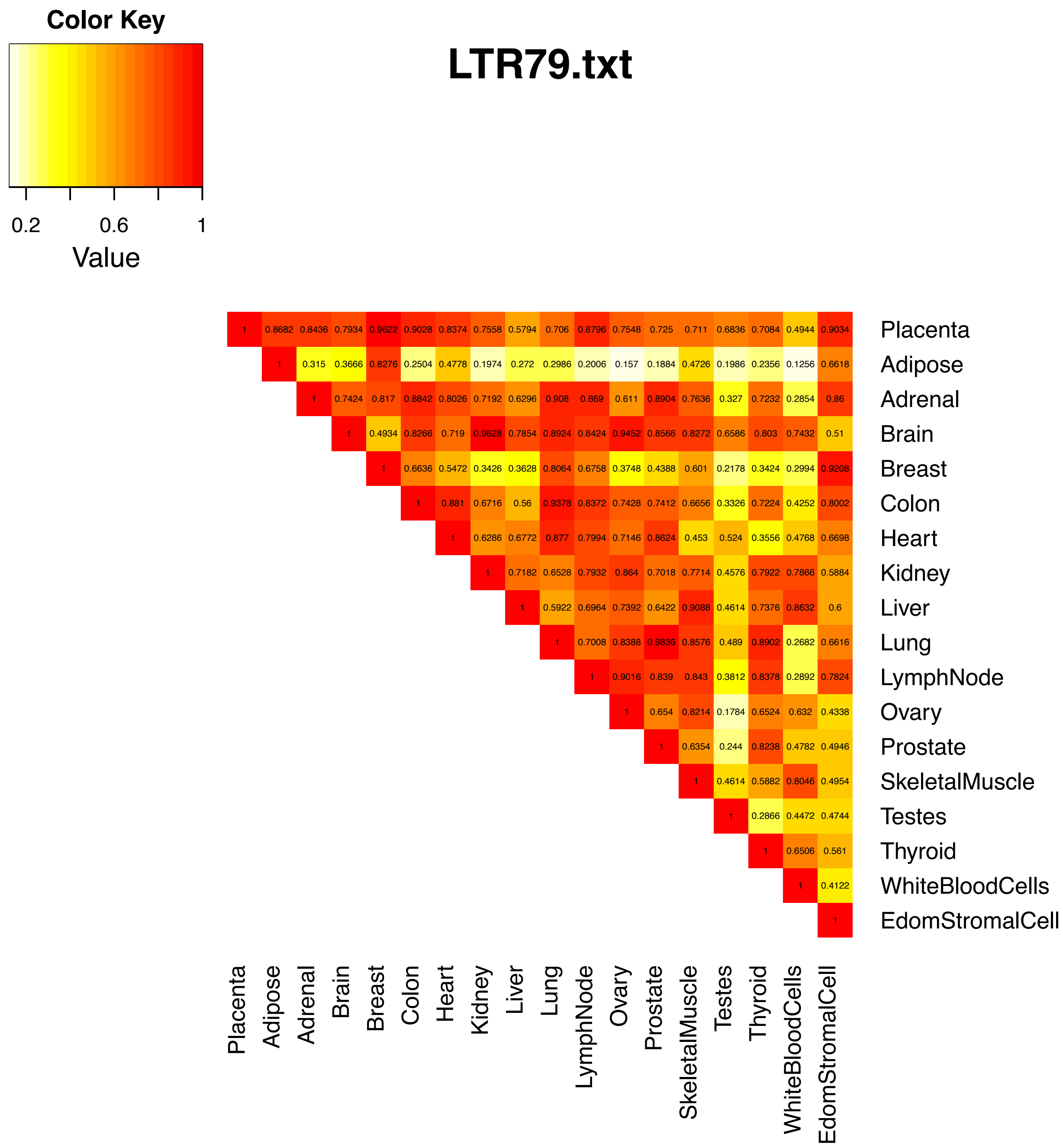
Color Key
0.2 0.6 1
Value
LTR79.txt
Placenta 1 0.8682 0.8436 0.7934 0.9622 0.9028 0.8374 0.7558 0.5794 0.706 0.8796 0.7548 0.725 0.711 0.6836 0.7084 0.4944 0.9034
Adipose 1 0.315 0.3666 0.8276 0.2504 0.4778 0.1974 0.272 0.2986 0.2006 0.157 0.1884 0.4726 0.1986 0.2356 0.1256 0.6618
Adrenal 1 0.7424 0.817 0.8842 0.8026 0.7192 0.6296 0.908 0.869 0.611 0.8904 0.7636 0.327 0.7232 0.2854 0.86
Brain 1 0.4934 0.8266 0.719 0.9628 0.7854 0.8924 0.8424 0.9452 0.8566 0.8272 0.6586 0.803 0.7432 0.51
Breast 1 0.6636 0.5472 0.3426 0.3628 0.8064 0.6758 0.3748 0.4388 0.601 0.2178 0.3424 0.2994 0.9208
Colon 1 0.881 0.6716 0.56 0.9378 0.8372 0.7428 0.7412 0.6656 0.3326 0.7224 0.4252 0.8002
Heart 1 0.6286 0.6772 0.877 0.7994 0.7146 0.8624 0.453 0.524 0.3556 0.4768 0.6698
Kidney 1 0.7182 0.6528 0.7932 0.864 0.7018 0.7714 0.4576 0.7922 0.7866 0.5884
Liver 1 0.5922 0.6964 0.7392 0.6422 0.9088 0.4614 0.7376 0.8632 0.6
Lung 1 0.7008 0.8388 0.9836 0.8576 0.489 0.8902 0.2682 0.6616
LymphNode 1 0.9016 0.839 0.843 0.3812 0.8378 0.2892 0.7824
Ovary 1 0.654 0.8214 0.1784 0.6524 0.632 0.4338
Prostate 1 0.6354 0.244 0.8238 0.4782 0.4946
SkeletalMuscle 1 0.4614 0.5882 0.8046 0.4954
Testes 1 0.2866 0.4472 0.4744
Thyroid 1 0.6506 0.561
WhiteBloodCells 1 0.4122
EdomStromalCell 1
Placenta Adipose Adrenal Brain Breast Colon Heart Kidney Liver Lung LymphNode Ovary Prostate SkeletalMuscle Testes Thyroid WhiteBloodCells EdomStromalCell

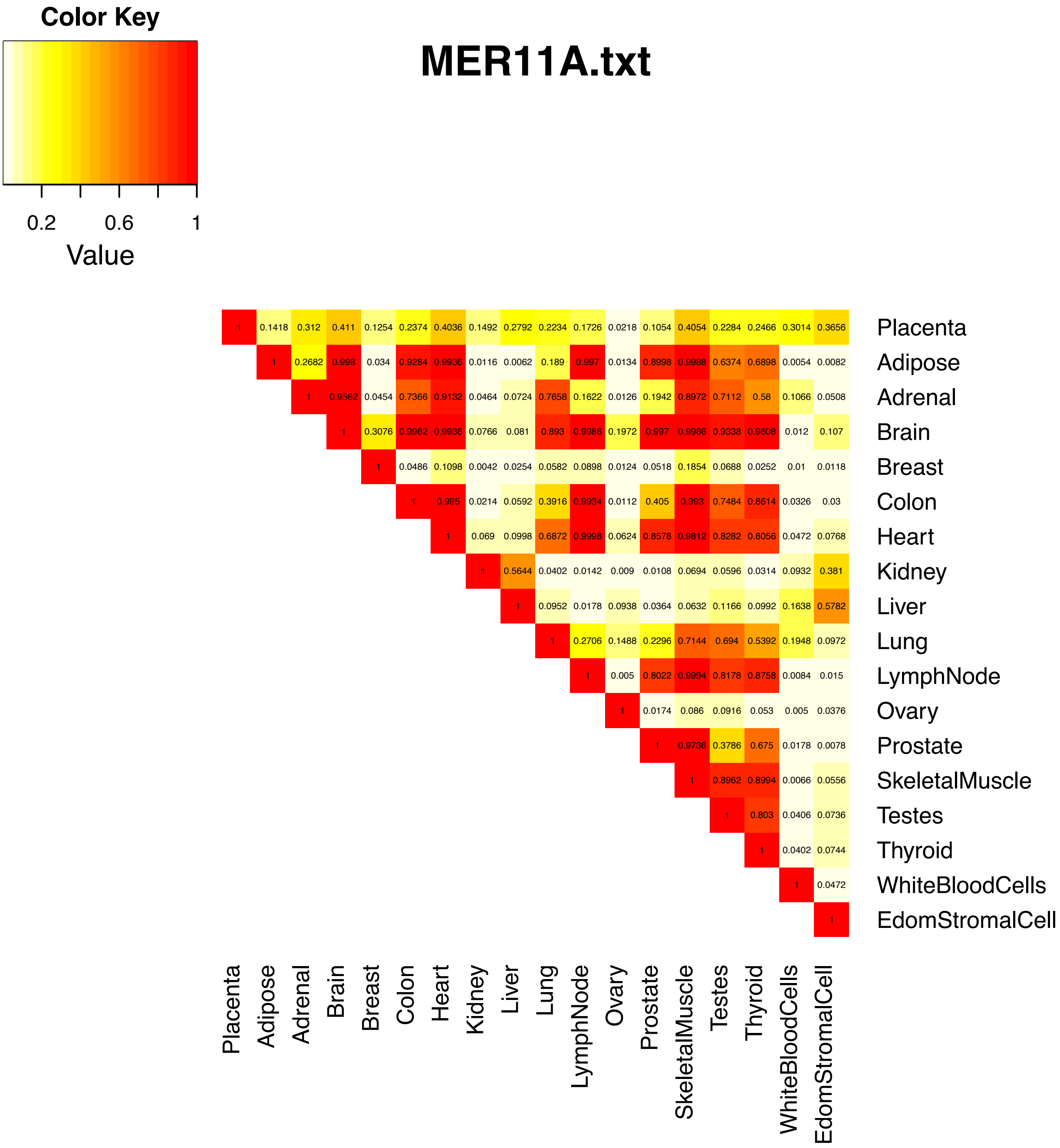
Color Key
0.2
0.6
1
Value
MER11A.txt
Placenta 1 0.1418 0.312 0.411 0.1254 0.2374 0.4036 0.1492 0.2792 0.2234 0.1726 0.0218 0.1054 0.4054 0.2284 0.2466 0.3014 0.3656
Adipose 1 0.2682 0.998 0.034 0.9284 0.9936 0.0116 0.0062 0.189 0.997 0.0134 0.8998 0.9988 0.6374 0.6898 0.0054 0.0082
Adrenal 1 0.9562 0.0454 0.7366 0.9132 0.0464 0.0724 0.7658 0.1622 0.0126 0.1942 0.8972 0.7112 0.58 0.1066 0.0508
Brain 1 0.3076 0.9962 0.9936 0.0766 0.081 0.893 0.9986 0.1972 0.997 0.9986 0.9338 0.9608 0.012 0.107
Breast 1 0.0486 0.1098 0.0042 0.0254 0.0582 0.0898 0.0124 0.0518 0.1854 0.0688 0.0252 0.01 0.0118
Colon 1 0.995 0.0214 0.0592 0.3916 0.9934 0.0112 0.405 0.993 0.7484 0.8614 0.0326 0.03
Heart 1 0.069 0.0998 0.6872 0.9998 0.0624 0.8578 0.9812 0.8282 0.8056 0.0472 0.0768
Kidney 1 0.5644 0.0402 0.0142 0.009 0.0108 0.0694 0.0596 0.0314 0.0932 0.381
Liver 1 0.0952 0.0178 0.0938 0.0364 0.0632 0.1166 0.0992 0.1638 0.5782
Lung 1 0.2706 0.1488 0.2296 0.7144 0.694 0.5392 0.1948 0.0972
LymphNode 1 0.005 0.8022 0.9994 0.8178 0.8758 0.0084 0.015
Ovary 1 0.0174 0.086 0.0916 0.053 0.005 0.0376
Prostate 1 0.9736 0.3786 0.675 0.0178 0.0078
SkeletalMuscle 1 0.8962 0.8994 0.0066 0.0556
Testes 1 0.803 0.0406 0.0736
Thyroid 1 0.0402 0.0744
WhiteBloodCells 1 0.0472
EdomStromalCell 1
Placenta Adipose Adrenal Brain Breast Colon Heart Kidney Liver Lung LymphNode Ovary Prostate SkeletalMuscle Testes Thyroid WhiteBloodCells EdomStromalCell

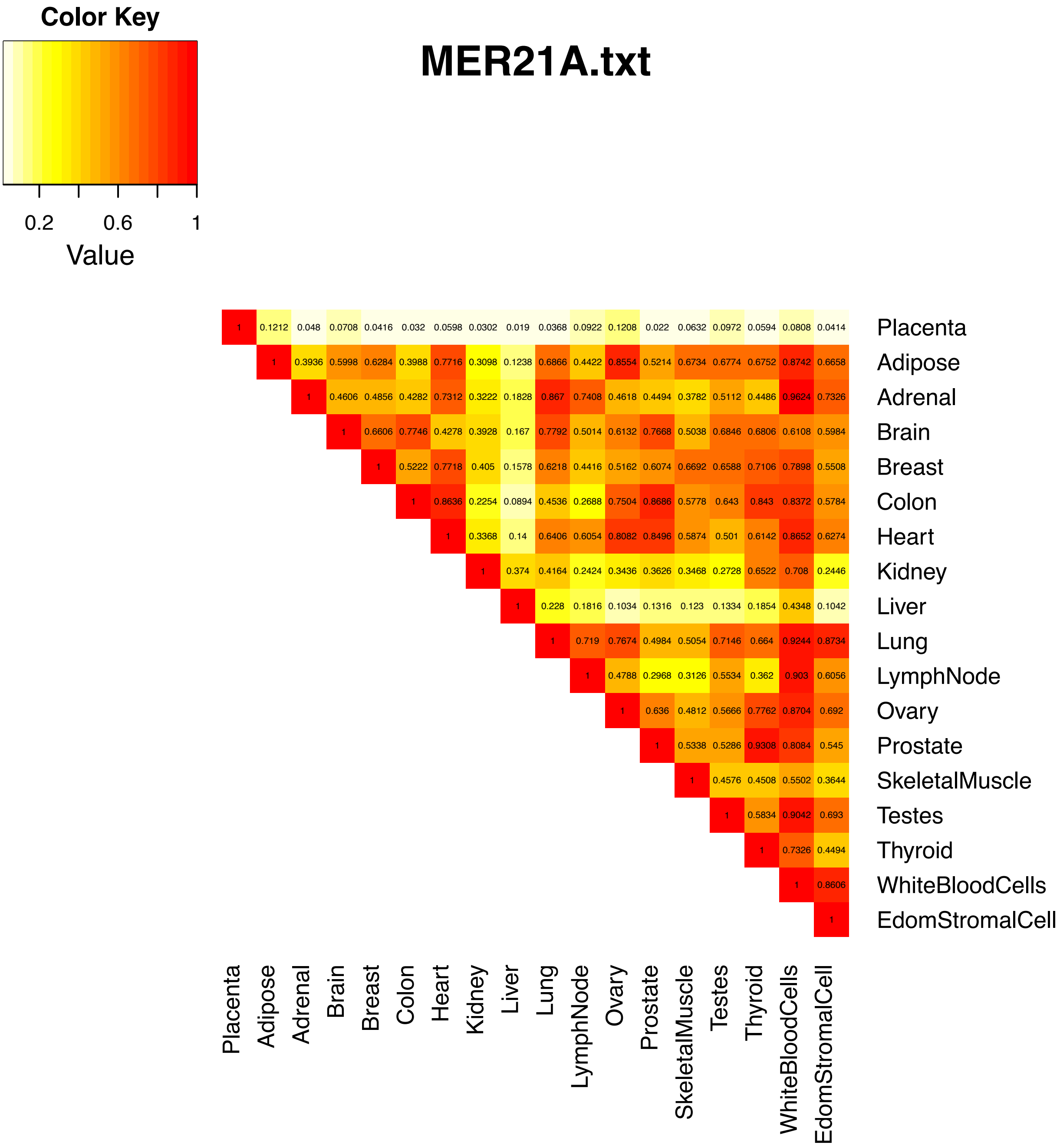
MER21A.txt
Color Key
0.2
0.6
1
Value
Placenta
Adipose
Adrenal
Brain
Breast
Colon
Heart
Kidney
Liver
Lung
LymphNode
Ovary
Prostate
SkeletalMuscle
Testes
Thyroid
WhiteBloodCells
EdomStromalCell

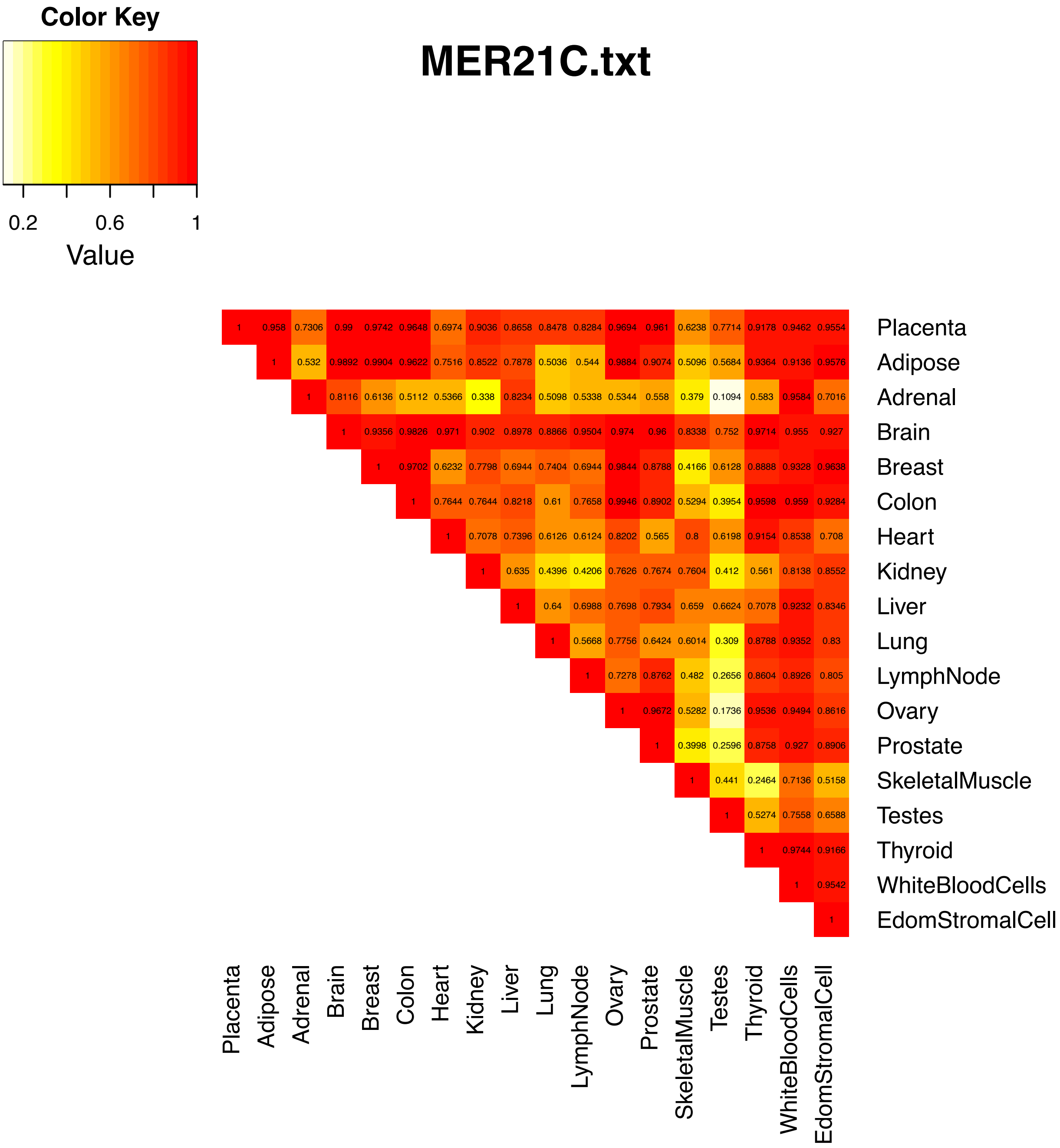

Color Key
0.2
0.6
1
Value
MER21C.txt
Placenta: 1 0.958 0.7306 0.99 0.9742 0.9648 0.6974 0.9036 0.8658 0.8478 0.8284 0.9694 0.961 0.6238 0.7714 0.9178 0.9462 0.9554
Adipose: 1 0.532 0.9892 0.9904 0.9622 0.7516 0.8522 0.7878 0.5036 0.544 0.9884 0.9074 0.5096 0.5684 0.9364 0.9136 0.9576
Adrenal: 1 0.8116 0.6136 0.5112 0.5366 0.338 0.8234 0.5098 0.5338 0.5344 0.558 0.379 0.1094 0.583 0.9584 0.7016
Brain: 1 0.9356 0.9826 0.971 0.902 0.8978 0.8866 0.9504 0.974 0.96 0.8338 0.752 0.9714 0.955 0.927
Breast: 1 0.9702 0.6232 0.7798 0.6944 0.7404 0.6944 0.9844 0.8788 0.4166 0.6128 0.8888 0.9328 0.9638
Colon: 1 0.7644 0.7644 0.8218 0.61 0.7658 0.9946 0.8902 0.5294 0.3954 0.9598 0.959 0.9284
Heart: 1 0.7078 0.7396 0.6126 0.6124 0.8202 0.565 0.8 0.6198 0.9154 0.8538 0.708
Kidney: 1 0.635 0.4396 0.4206 0.7626 0.7674 0.7604 0.412 0.561 0.8138 0.8552
Liver: 1 0.64 0.6988 0.7698 0.7934 0.659 0.6624 0.7078 0.9232 0.8346
Lung: 1 0.5668 0.7756 0.6424 0.6014 0.309 0.8788 0.9352 0.83
LymphNode: 1 0.7278 0.8762 0.482 0.2656 0.8604 0.8926 0.805
Ovary: 1 0.9672 0.5282 0.1736 0.9536 0.9494 0.8616
Prostate: 1 0.3998 0.2596 0.8758 0.927 0.8906
SkeletalMuscle: 1 0.441 0.2464 0.7136 0.5158
Testes: 1 0.5274 0.7558 0.6588
Thyroid: 1 0.9744 0.9166
WhiteBloodCells: 1 0.9542
EdomStromalCell: 1
Placenta Adipose Adrenal Brain Breast Colon Heart Kidney Liver Lung LymphNode Ovary Prostate SkeletalMuscle Testes Thyroid WhiteBloodCells EdomStromalCell

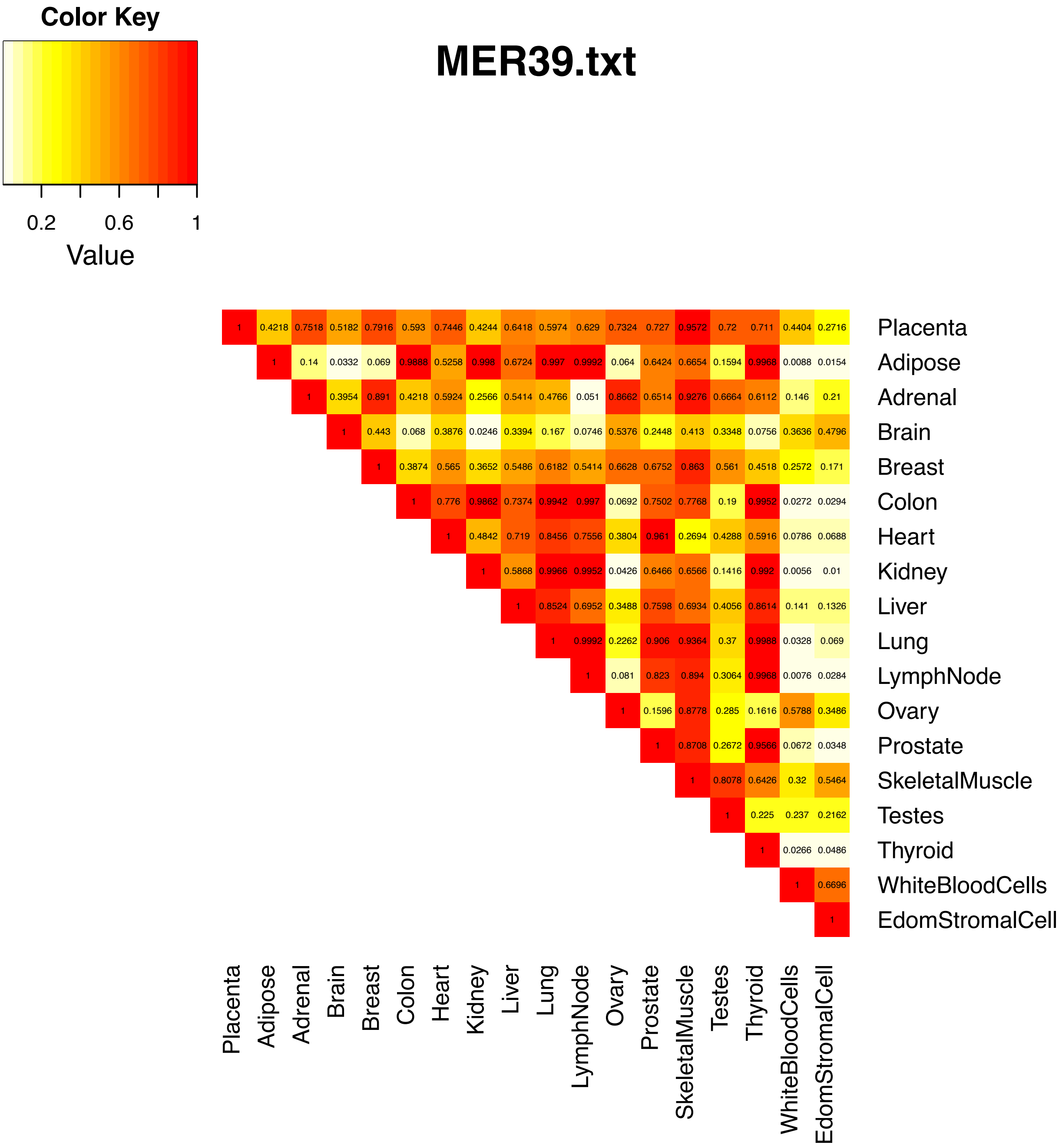
Color Key
0.2 0.6 1
Value
MER39.txt
Placenta 1 0.4218 0.7518 0.5182 0.7916 0.593 0.7446 0.4244 0.6418 0.5974 0.629 0.7324 0.727 0.9572 0.72 0.711 0.4404 0.2716
Adipose 1 0.14 0.0332 0.069 0.9888 0.5258 0.998 0.6724 0.997 0.9992 0.064 0.6424 0.6654 0.1594 0.9968 0.0088 0.0154
Adrenal 1 0.3954 0.891 0.4218 0.5924 0.2566 0.5414 0.4766 0.051 0.8662 0.6514 0.9276 0.6664 0.6112 0.146 0.21
Brain 1 0.443 0.068 0.3876 0.0246 0.3394 0.167 0.0746 0.5376 0.2448 0.413 0.3348 0.0756 0.3636 0.4796
Breast 1 0.3874 0.565 0.3652 0.5486 0.6182 0.5414 0.6628 0.6752 0.863 0.561 0.4518 0.2572 0.171
Colon 1 0.776 0.9862 0.7374 0.9942 0.997 0.0692 0.7502 0.7768 0.19 0.9952 0.0272 0.0294
Heart 1 0.4842 0.719 0.8456 0.7556 0.3804 0.961 0.2694 0.4288 0.5916 0.0786 0.0688
Kidney 1 0.5868 0.9966 0.9952 0.0426 0.6466 0.6566 0.1416 0.992 0.0056 0.01
Liver 1 0.8524 0.6952 0.3488 0.7598 0.6934 0.4056 0.8614 0.141 0.1326
Lung 1 0.9992 0.2262 0.906 0.9364 0.37 0.9988 0.0328 0.069
LymphNode 1 0.081 0.823 0.894 0.3064 0.9968 0.0076 0.0284
Ovary 1 0.1596 0.8778 0.285 0.1616 0.5788 0.3486
Prostate 1 0.8708 0.2672 0.9566 0.0672 0.0348
SkeletalMuscle 1 0.8078 0.6426 0.32 0.5464
Testes 1 0.225 0.237 0.2162
Thyroid 1 0.0266 0.0486
WhiteBloodCells 1 0.6696
EdomStromalCell 1
Placenta Adipose Adrenal Brain Breast Colon Heart Kidney Liver Lung LymphNode Ovary Prostate SkeletalMuscle Testes Thyroid WhiteBloodCells EdomStromalCell

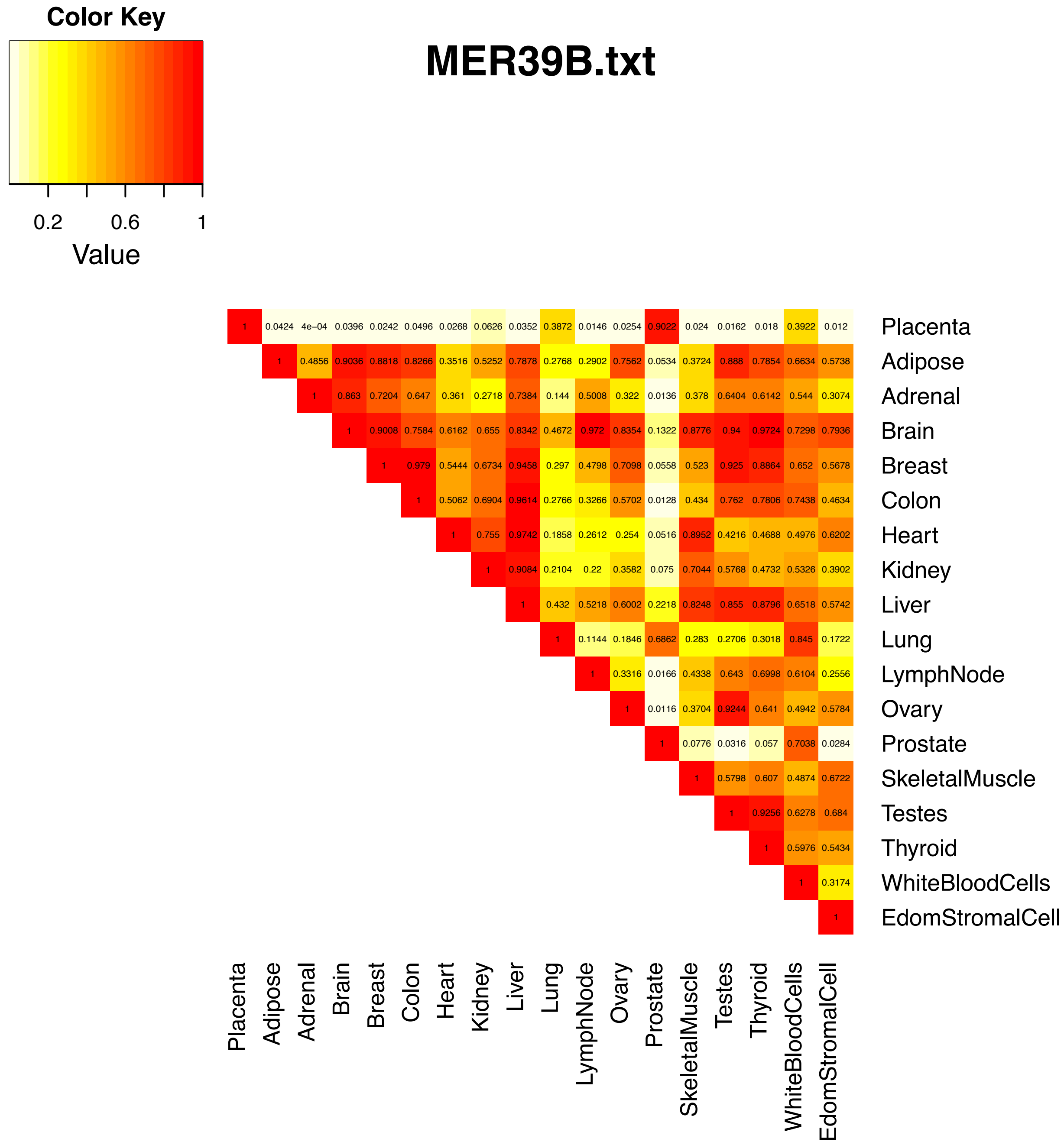
Color Key
0.2
0.6
1
Value
MER39B.txt
Placenta
Adipose
Adrenal
Brain
Breast
Colon
Heart
Kidney
Liver
Lung
LymphNode
Ovary
Prostate
SkeletalMuscle
Testes
Thyroid
WhiteBloodCells
EdomStromalCell
1 0.0424 4e−04 0.0396 0.0242 0.0496 0.0268 0.0626 0.0352 0.3872 0.0146 0.0254 0.9022 0.024 0.0162 0.018 0.3922 0.012
1 0.4856 0.9036 0.8818 0.8266 0.3516 0.5252 0.7878 0.2768 0.2902 0.7562 0.0534 0.3724 0.888 0.7854 0.6634 0.5738
1 0.863 0.7204 0.647 0.361 0.2718 0.7384 0.144 0.5008 0.322 0.0136 0.378 0.6404 0.6142 0.544 0.3074
1 0.9008 0.7584 0.6162 0.655 0.8342 0.4672 0.972 0.8354 0.1322 0.8776 0.94 0.9724 0.7298 0.7936
1 0.979 0.5444 0.6734 0.9458 0.297 0.4798 0.7098 0.0558 0.523 0.925 0.8864 0.652 0.5678
1 0.5062 0.6904 0.9614 0.2766 0.3266 0.5702 0.0128 0.434 0.762 0.7806 0.7438 0.4634
1 0.755 0.9742 0.1858 0.2612 0.254 0.0516 0.8952 0.4216 0.4688 0.4976 0.6202
1 0.9084 0.2104 0.22 0.3582 0.075 0.7044 0.5768 0.4732 0.5326 0.3902
1 0.432 0.5218 0.6002 0.2218 0.8248 0.855 0.8796 0.6518 0.5742
1 0.1144 0.1846 0.6862 0.283 0.2706 0.3018 0.845 0.1722
1 0.3316 0.0166 0.4338 0.643 0.6998 0.6104 0.2556
1 0.0116 0.3704 0.9244 0.641 0.4942 0.5784
1 0.0776 0.0316 0.057 0.7038 0.0284
1 0.5798 0.607 0.4874 0.6722
1 0.9256 0.6278 0.684
1 0.5976 0.5434
1 0.3174
1

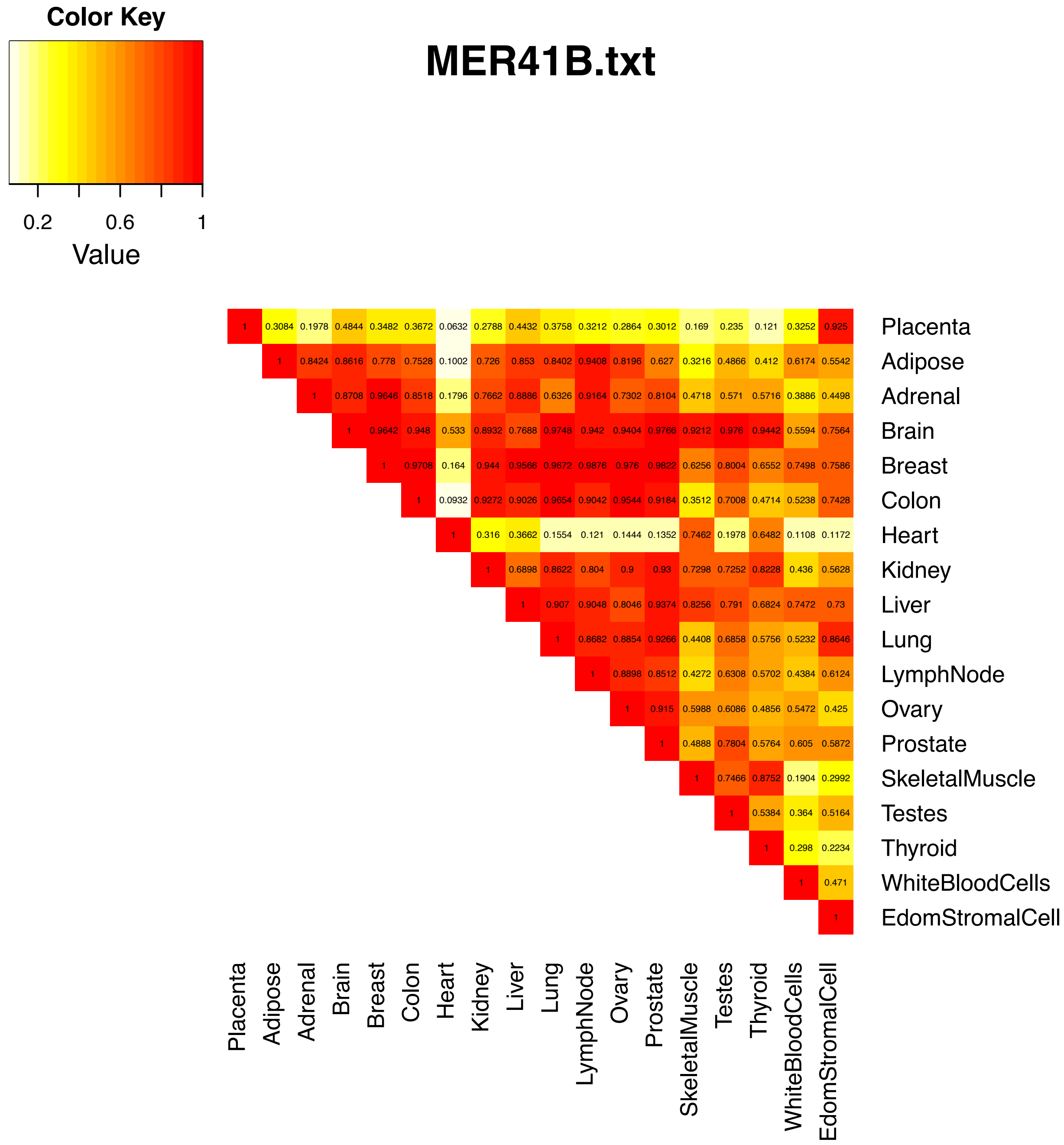
Color Key
0.2
0.6
1
Value
MER41B.txt
Placenta 1 0.3084 0.1978 0.4844 0.3482 0.3672 0.0632 0.2788 0.4432 0.3758 0.3212 0.2864 0.3012 0.169 0.235 0.121 0.3252 0.925
Adipose 1 0.8424 0.8616 0.778 0.7528 0.1002 0.726 0.853 0.8402 0.9408 0.8196 0.627 0.3216 0.4866 0.412 0.6174 0.5542
Adrenal 1 0.8708 0.9646 0.8518 0.1796 0.7662 0.8886 0.6326 0.9164 0.7302 0.8104 0.4718 0.571 0.5716 0.3886 0.4498
Brain 1 0.9642 0.948 0.533 0.8932 0.7688 0.9748 0.942 0.9404 0.9766 0.9212 0.976 0.9442 0.5594 0.7564
Breast 1 0.9708 0.164 0.944 0.9566 0.9672 0.9876 0.976 0.9822 0.6256 0.8004 0.6552 0.7498 0.7586
Colon 1 0.0932 0.9272 0.9026 0.9654 0.9042 0.9544 0.9184 0.3512 0.7008 0.4714 0.5238 0.7428
Heart 1 0.316 0.3662 0.1554 0.121 0.1444 0.1352 0.7462 0.1978 0.6482 0.1108 0.1172
Kidney 1 0.6898 0.8622 0.804 0.9 0.93 0.7298 0.7252 0.8228 0.436 0.5628
Liver 1 0.907 0.9048 0.8046 0.9374 0.8256 0.791 0.6824 0.7472 0.73
Lung 1 0.8682 0.8854 0.9266 0.4408 0.6858 0.5756 0.5232 0.8646
LymphNode 1 0.8898 0.8512 0.4272 0.6308 0.5702 0.4384 0.6124
Ovary 1 0.915 0.5988 0.6086 0.4856 0.5472 0.425
Prostate 1 0.4888 0.7804 0.5764 0.605 0.5872
SkeletalMuscle 1 0.7466 0.8752 0.1904 0.2992
Testes 1 0.5384 0.364 0.5164
Thyroid 1 0.298 0.2234
WhiteBloodCells 1 0.471
EdomStromalCell 1
Placenta
Adipose
Adrenal
Brain
Breast
Colon
Heart
Kidney
Liver
Lung
LymphNode
Ovary
Prostate
SkeletalMuscle
Testes
Thyroid
WhiteBloodCells
EdomStromalCell

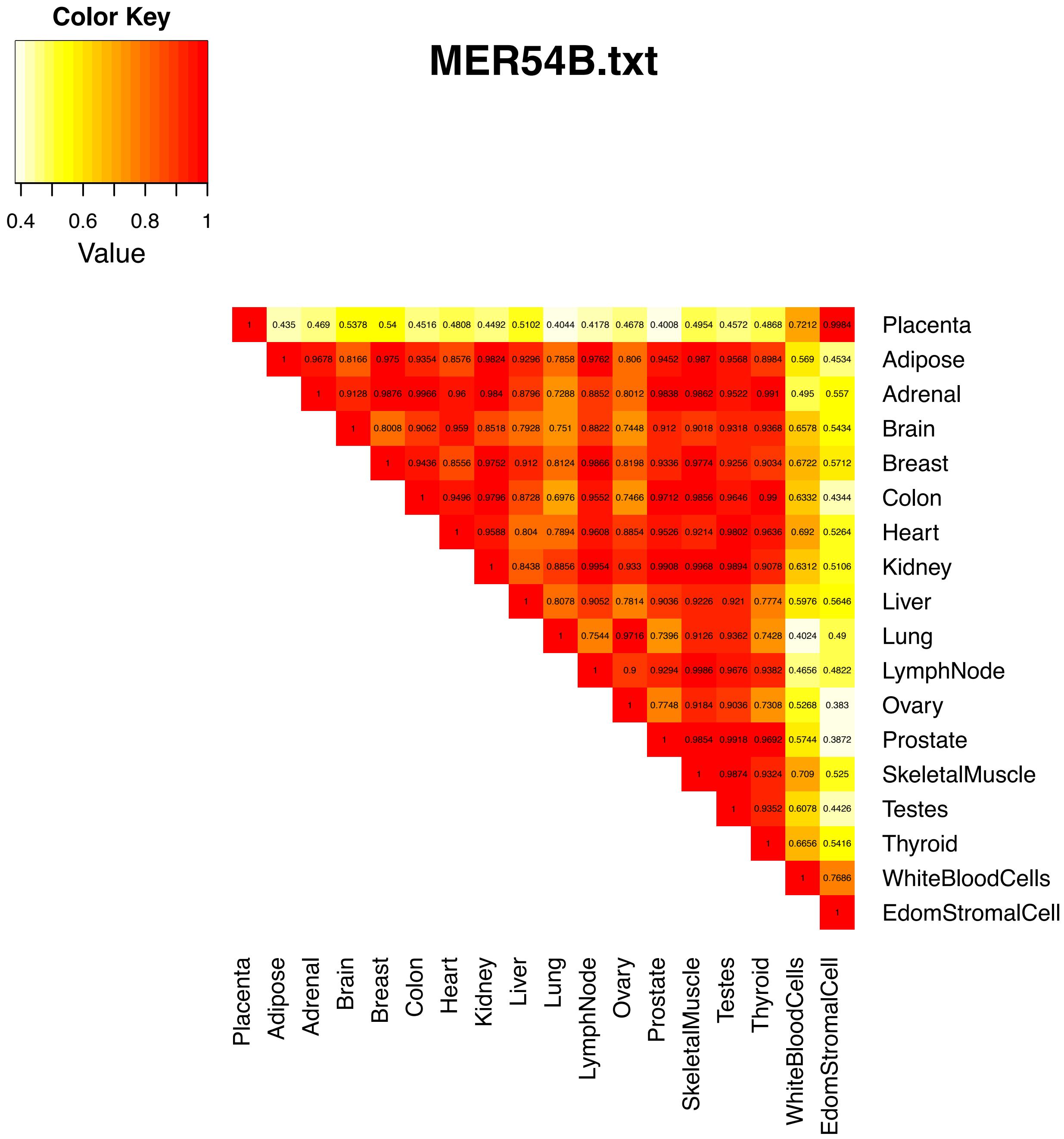
Color Key
0.4
0.6
0.8
1
Value
MER54B.txt
Placenta: 1 0.435 0.469 0.5378 0.54 0.4516 0.4808 0.4492 0.5102 0.4044 0.4178 0.4678 0.4008 0.4954 0.4572 0.4868 0.7212 0.9984
Adipose: 1 0.9678 0.8166 0.975 0.9354 0.8576 0.9824 0.9296 0.7858 0.9762 0.806 0.9452 0.987 0.9568 0.8984 0.569 0.4534
Adrenal: 1 0.9128 0.9876 0.9966 0.96 0.984 0.8796 0.7288 0.8852 0.8012 0.9838 0.9862 0.9522 0.991 0.495 0.557
Brain: 1 0.8008 0.9062 0.959 0.8518 0.7928 0.751 0.8822 0.7448 0.912 0.9018 0.9318 0.9368 0.6578 0.5434
Breast: 1 0.9436 0.8556 0.9752 0.912 0.8124 0.9866 0.8198 0.9336 0.9774 0.9256 0.9034 0.6722 0.5712
Colon: 1 0.9496 0.9796 0.8728 0.6976 0.9552 0.7466 0.9712 0.9856 0.9646 0.99 0.6332 0.4344
Heart: 1 0.9588 0.804 0.7894 0.9608 0.8854 0.9526 0.9214 0.9802 0.9636 0.692 0.5264
Kidney: 1 0.8438 0.8856 0.9954 0.933 0.9908 0.9968 0.9894 0.9078 0.6312 0.5106
Liver: 1 0.8078 0.9052 0.7814 0.9036 0.9226 0.921 0.7774 0.5976 0.5646
Lung: 1 0.7544 0.9716 0.7396 0.9126 0.9362 0.7428 0.4024 0.49
LymphNode: 1 0.9 0.9294 0.9986 0.9676 0.9382 0.4656 0.4822
Ovary: 1 0.7748 0.9184 0.9036 0.7308 0.5268 0.383
Prostate: 1 0.9854 0.9918 0.9692 0.5744 0.3872
SkeletalMuscle: 1 0.9874 0.9324 0.709 0.525
Testes: 1 0.9352 0.6078 0.4426
Thyroid: 1 0.6656 0.5416
WhiteBloodCells: 1 0.7686
EdomStromalCell: 1
Placenta Adipose Adrenal Brain Breast Colon Heart Kidney Liver Lung LymphNode Ovary Prostate SkeletalMuscle Testes Thyroid WhiteBloodCells EdomStromalCell

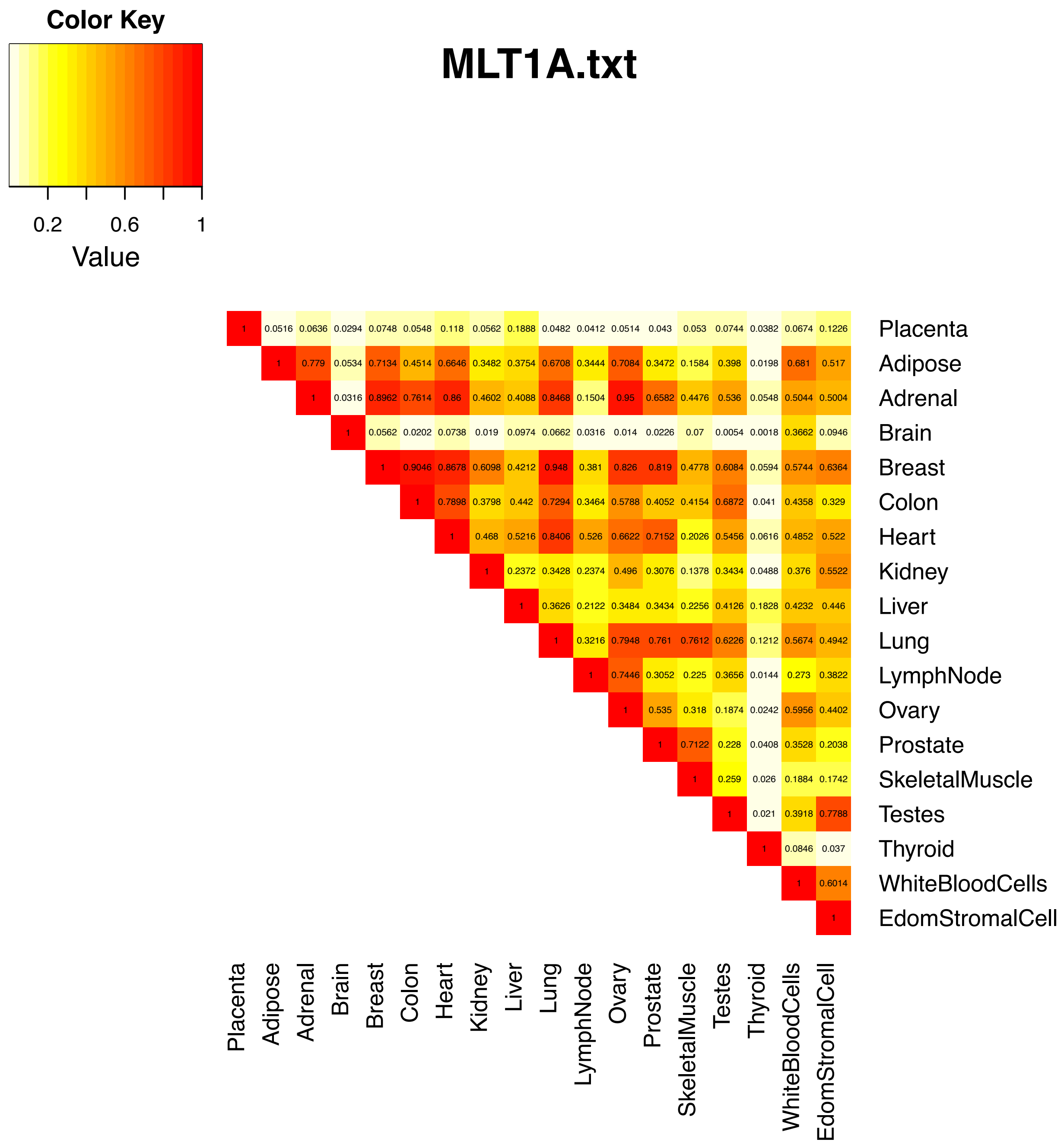
Color Key
0.2
0.6
1
Value
MLT1A.txt
Placenta 1 0.0516 0.0636 0.0294 0.0748 0.0548 0.118 0.0562 0.1888 0.0482 0.0412 0.0514 0.043 0.053 0.0744 0.0382 0.0674 0.1226
Adipose 1 0.779 0.0534 0.7134 0.4514 0.6646 0.3482 0.3754 0.6708 0.3444 0.7084 0.3472 0.1584 0.398 0.0198 0.681 0.517
Adrenal 1 0.0316 0.8962 0.7614 0.86 0.4602 0.4088 0.8468 0.1504 0.95 0.6582 0.4476 0.536 0.0548 0.5044 0.5004
Brain 1 0.0562 0.0202 0.0738 0.019 0.0974 0.0662 0.0316 0.014 0.0226 0.07 0.0054 0.0018 0.3662 0.0946
Breast 1 0.9046 0.8678 0.6098 0.4212 0.948 0.381 0.826 0.819 0.4778 0.6084 0.0594 0.5744 0.6364
Colon 1 0.7898 0.3798 0.442 0.7294 0.3464 0.5788 0.4052 0.4154 0.6872 0.041 0.4358 0.329
Heart 1 0.468 0.5216 0.8406 0.526 0.6622 0.7152 0.2026 0.5456 0.0616 0.4852 0.522
Kidney 1 0.2372 0.3428 0.2374 0.496 0.3076 0.1378 0.3434 0.0488 0.376 0.5522
Liver 1 0.3626 0.2122 0.3484 0.3434 0.2256 0.4126 0.1828 0.4232 0.446
Lung 1 0.3216 0.7948 0.761 0.7612 0.6226 0.1212 0.5674 0.4942
LymphNode 1 0.7446 0.3052 0.225 0.3656 0.0144 0.273 0.3822
Ovary 1 0.535 0.318 0.1874 0.0242 0.5956 0.4402
Prostate 1 0.7122 0.228 0.0408 0.3528 0.2038
SkeletalMuscle 1 0.259 0.026 0.1884 0.1742
Testes 1 0.021 0.3918 0.7788
Thyroid 1 0.0846 0.037
WhiteBloodCells 1 0.6014
EdomStromalCell 1
Placenta
Adipose
Adrenal
Brain
Breast
Colon
Heart
Kidney
Liver
Lung
LymphNode
Ovary
Prostate
SkeletalMuscle
Testes
Thyroid
WhiteBloodCells
EdomStromalCell

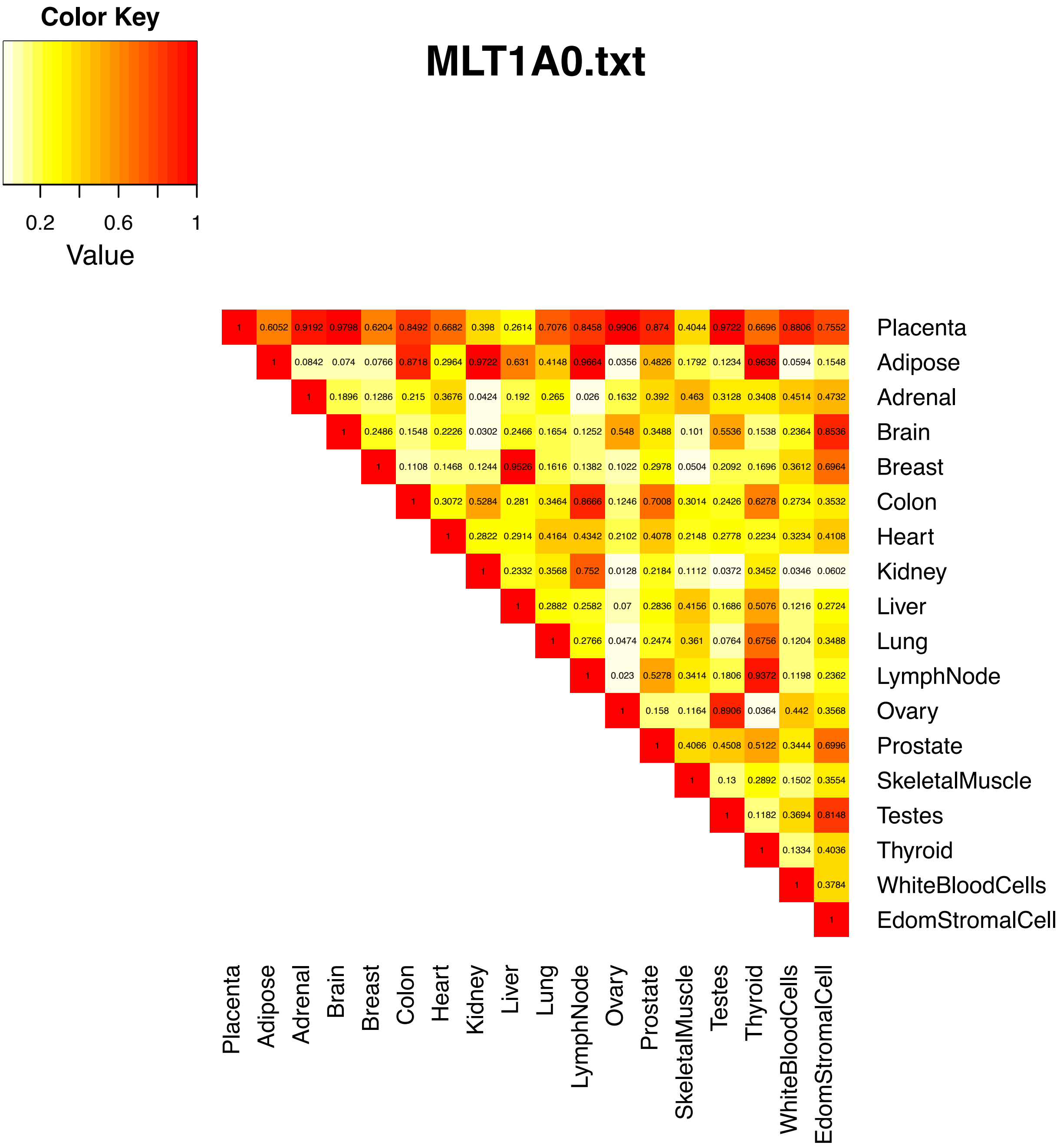
Color Key
0.2
0.6
1
Value
MLT1A0.txt
Placenta
Adipose
Adrenal
Brain
Breast
Colon
Heart
Kidney
Liver
Lung
LymphNode
Ovary
Prostate
SkeletalMuscle
Testes
Thyroid
WhiteBloodCells
EdomStromalCell
1 0.6052 0.9192 0.9798 0.6204 0.8492 0.6682 0.398 0.2614 0.7076 0.8458 0.9906 0.874 0.4044 0.9722 0.6696 0.8806 0.7552
1 0.0842 0.074 0.0766 0.8718 0.2964 0.9722 0.631 0.4148 0.9664 0.0356 0.4826 0.1792 0.1234 0.9636 0.0594 0.1548
1 0.1896 0.1286 0.215 0.3676 0.0424 0.192 0.265 0.026 0.1632 0.392 0.463 0.3128 0.3408 0.4514 0.4732
1 0.2486 0.1548 0.2226 0.0302 0.2466 0.1654 0.1252 0.548 0.3488 0.101 0.5536 0.1538 0.2364 0.8536
1 0.1108 0.1468 0.1244 0.9526 0.1616 0.1382 0.1022 0.2978 0.0504 0.2092 0.1696 0.3612 0.6964
1 0.3072 0.5284 0.281 0.3464 0.8666 0.1246 0.7008 0.3014 0.2426 0.6278 0.2734 0.3532
1 0.2822 0.2914 0.4164 0.4342 0.2102 0.4078 0.2148 0.2778 0.2234 0.3234 0.4108
1 0.2332 0.3568 0.752 0.0128 0.2184 0.1112 0.0372 0.3452 0.0346 0.0602
1 0.2882 0.2582 0.07 0.2836 0.4156 0.1686 0.5076 0.1216 0.2724
1 0.2766 0.0474 0.2474 0.361 0.0764 0.6756 0.1204 0.3488
1 0.023 0.5278 0.3414 0.1806 0.9372 0.1198 0.2362
1 0.158 0.1164 0.8906 0.0364 0.442 0.3568
1 0.4066 0.4508 0.5122 0.3444 0.6996
1 0.13 0.2892 0.1502 0.3554
1 0.1182 0.3694 0.8148
1 0.1334 0.4036
1 0.3784
1

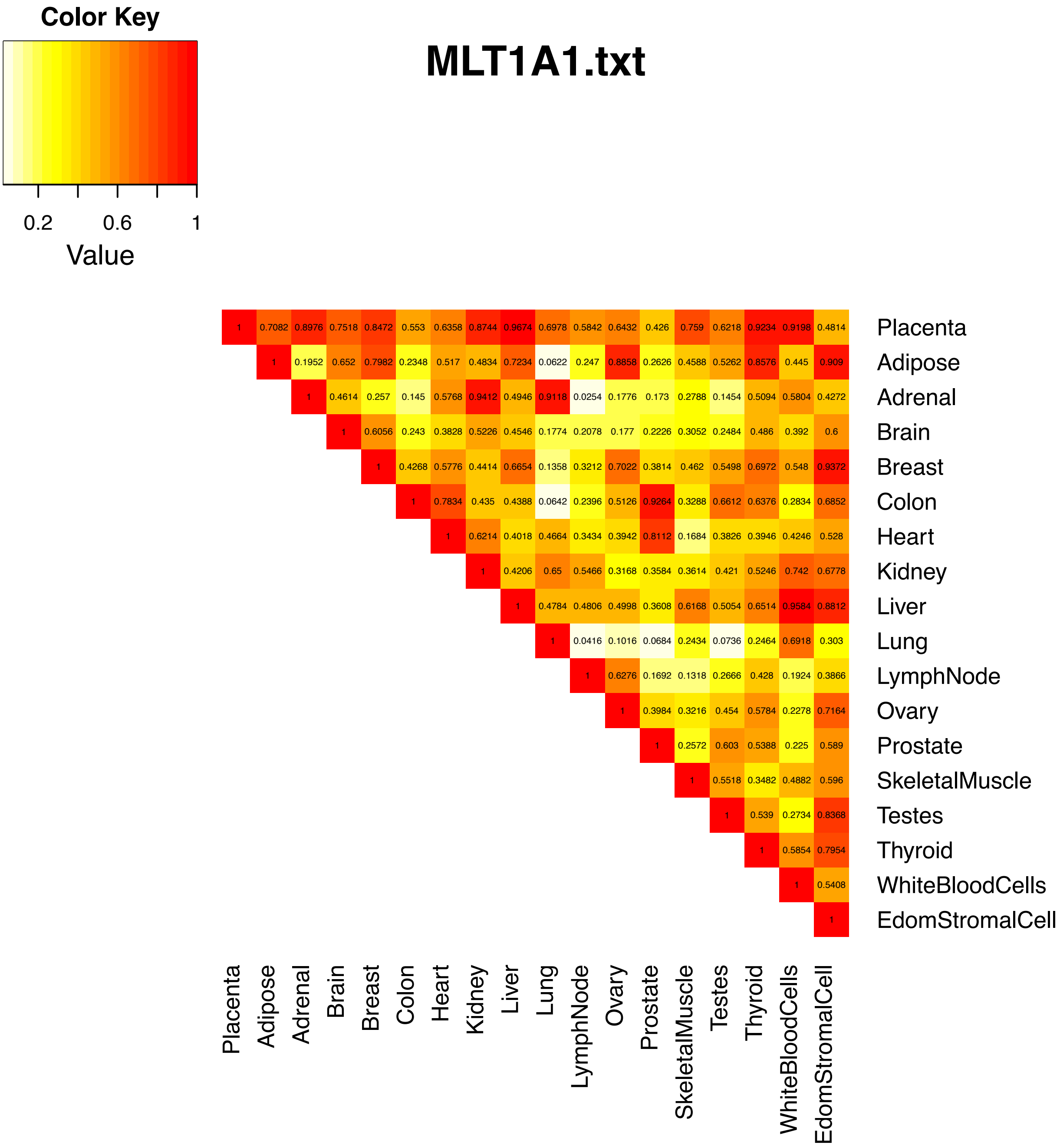

	Placenta	Adipose	Adrenal	Brain	Breast	Colon	Heart	Kidney	Liver	Lung	LymphNode	Ovary	Prostate	SkeletalMuscle	Testes	Thyroid	WhiteBloodCells	EdomStromalCell
Placenta	1	0.7082	0.8976	0.7518	0.8472	0.553	0.6358	0.8744	0.9674	0.6978	0.5842	0.6432	0.426	0.759	0.6218	0.9234	0.9198	0.4814
Adipose		1	0.1952	0.652	0.7982	0.2348	0.517	0.4834	0.7234	0.0622	0.247	0.8858	0.2626	0.4588	0.5262	0.8576	0.445	0.909
Adrenal			1	0.4614	0.257	0.145	0.5768	0.9412	0.4946	0.9118	0.0254	0.1776	0.173	0.2788	0.1454	0.5094	0.5804	0.4272
Brain				1	0.6056	0.243	0.3828	0.5226	0.4546	0.1774	0.2078	0.177	0.2226	0.3052	0.2484	0.486	0.392	0.6
Breast					1	0.4268	0.5776	0.4414	0.6654	0.1358	0.3212	0.7022	0.3814	0.462	0.5498	0.6972	0.548	0.9372
Colon						1	0.7834	0.435	0.4388	0.0642	0.2396	0.5126	0.9264	0.3288	0.6612	0.6376	0.2834	0.6852
Heart							1	0.6214	0.4018	0.4664	0.3434	0.3942	0.8112	0.1684	0.3826	0.3946	0.4246	0.528
Kidney								1	0.4206	0.65	0.5466	0.3168	0.3584	0.3614	0.421	0.5246	0.742	0.6778
Liver									1	0.4784	0.4806	0.4998	0.3608	0.6168	0.5054	0.6514	0.9584	0.8812
Lung										1	0.0416	0.1016	0.0684	0.2434	0.0736	0.2464	0.6918	0.303
LymphNode											1	0.6276	0.1692	0.1318	0.2666	0.428	0.1924	0.3866
Ovary												1	0.3984	0.3216	0.454	0.5784	0.2278	0.7164
Prostate													1	0.2572	0.603	0.5388	0.225	0.589
SkeletalMuscle														1	0.5518	0.3482	0.4882	0.596
Testes															1	0.539	0.2734	0.8368
Thyroid																1	0.5854	0.7954
WhiteBloodCells																	1	0.5408
EdomStromalCell																		1

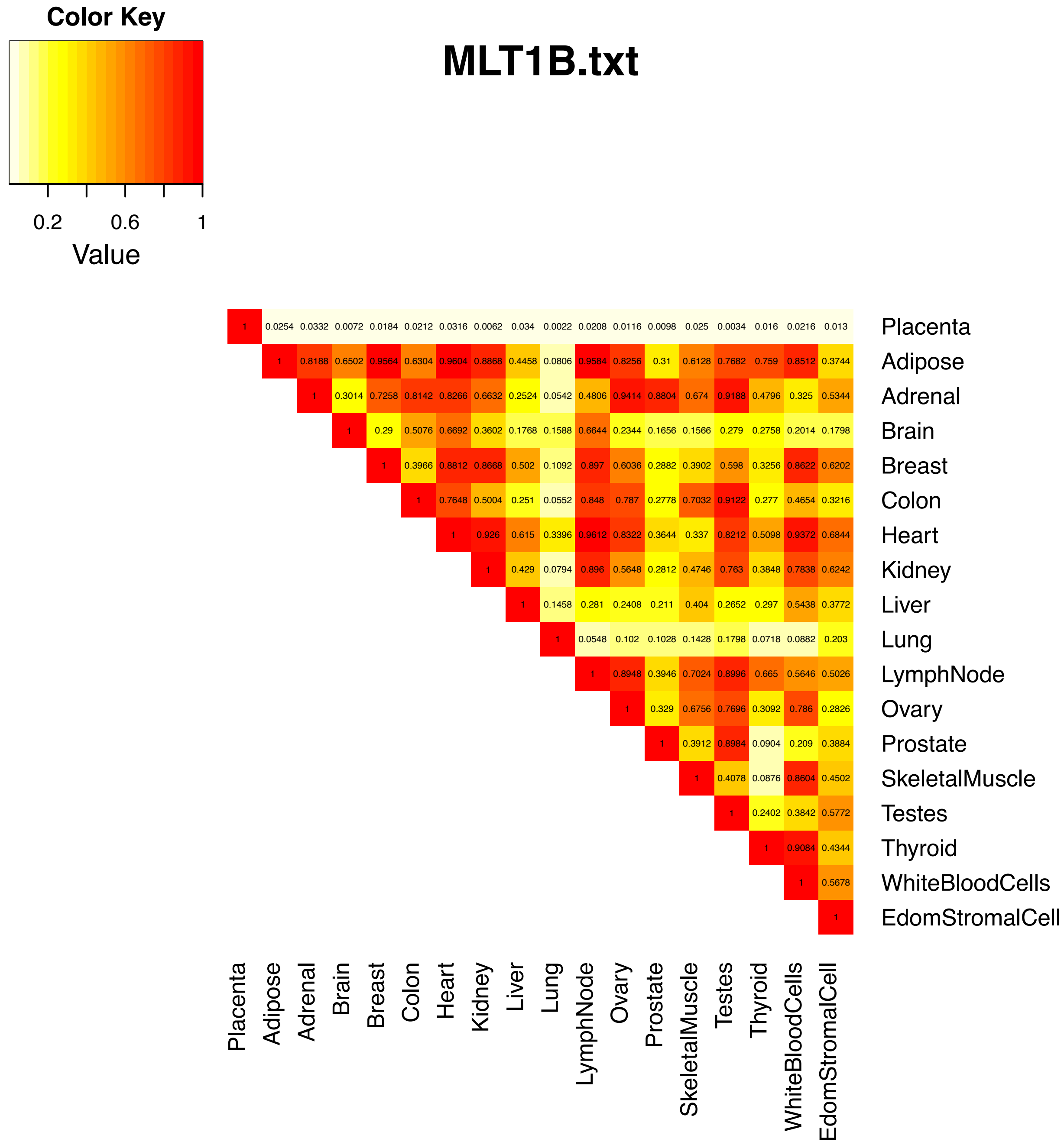
MLT1B.txt
Color Key
0.2
0.6
1
Value
Placenta 1 0.0254 0.0332 0.0072 0.0184 0.0212 0.0316 0.0062 0.034 0.0022 0.0208 0.0116 0.0098 0.025 0.0034 0.016 0.0216 0.013
Adipose 1 0.8188 0.6502 0.9564 0.6304 0.9604 0.8868 0.4458 0.0806 0.9584 0.8256 0.31 0.6128 0.7682 0.759 0.8512 0.3744
Adrenal 1 0.3014 0.7258 0.8142 0.8266 0.6632 0.2524 0.0542 0.4806 0.9414 0.8804 0.674 0.9188 0.4796 0.325 0.5344
Brain 1 0.29 0.5076 0.6692 0.3602 0.1768 0.1588 0.6644 0.2344 0.1656 0.1566 0.279 0.2758 0.2014 0.1798
Breast 1 0.3966 0.8812 0.8668 0.502 0.1092 0.897 0.6036 0.2882 0.3902 0.598 0.3256 0.8622 0.6202
Colon 1 0.7648 0.5004 0.251 0.0552 0.848 0.787 0.2778 0.7032 0.9122 0.277 0.4654 0.3216
Heart 1 0.926 0.615 0.3396 0.9612 0.8322 0.3644 0.337 0.8212 0.5098 0.9372 0.6844
Kidney 1 0.429 0.0794 0.896 0.5648 0.2812 0.4746 0.763 0.3848 0.7838 0.6242
Liver 1 0.1458 0.281 0.2408 0.211 0.404 0.2652 0.297 0.5438 0.3772
Lung 1 0.0548 0.102 0.1028 0.1428 0.1798 0.0718 0.0882 0.203
LymphNode 1 0.8948 0.3946 0.7024 0.8996 0.665 0.5646 0.5026
Ovary 1 0.329 0.6756 0.7696 0.3092 0.786 0.2826
Prostate 1 0.3912 0.8984 0.0904 0.209 0.3884
SkeletalMuscle 1 0.4078 0.0876 0.8604 0.4502
Testes 1 0.2402 0.3842 0.5772
Thyroid 1 0.9084 0.4344
WhiteBloodCells 1 0.5678
EdomStromalCell 1
Placenta
Adipose
Adrenal
Brain
Breast
Colon
Heart
Kidney
Liver
Lung
LymphNode
Ovary
Prostate
SkeletalMuscle
Testes
Thyroid
WhiteBloodCells
EdomStromalCell

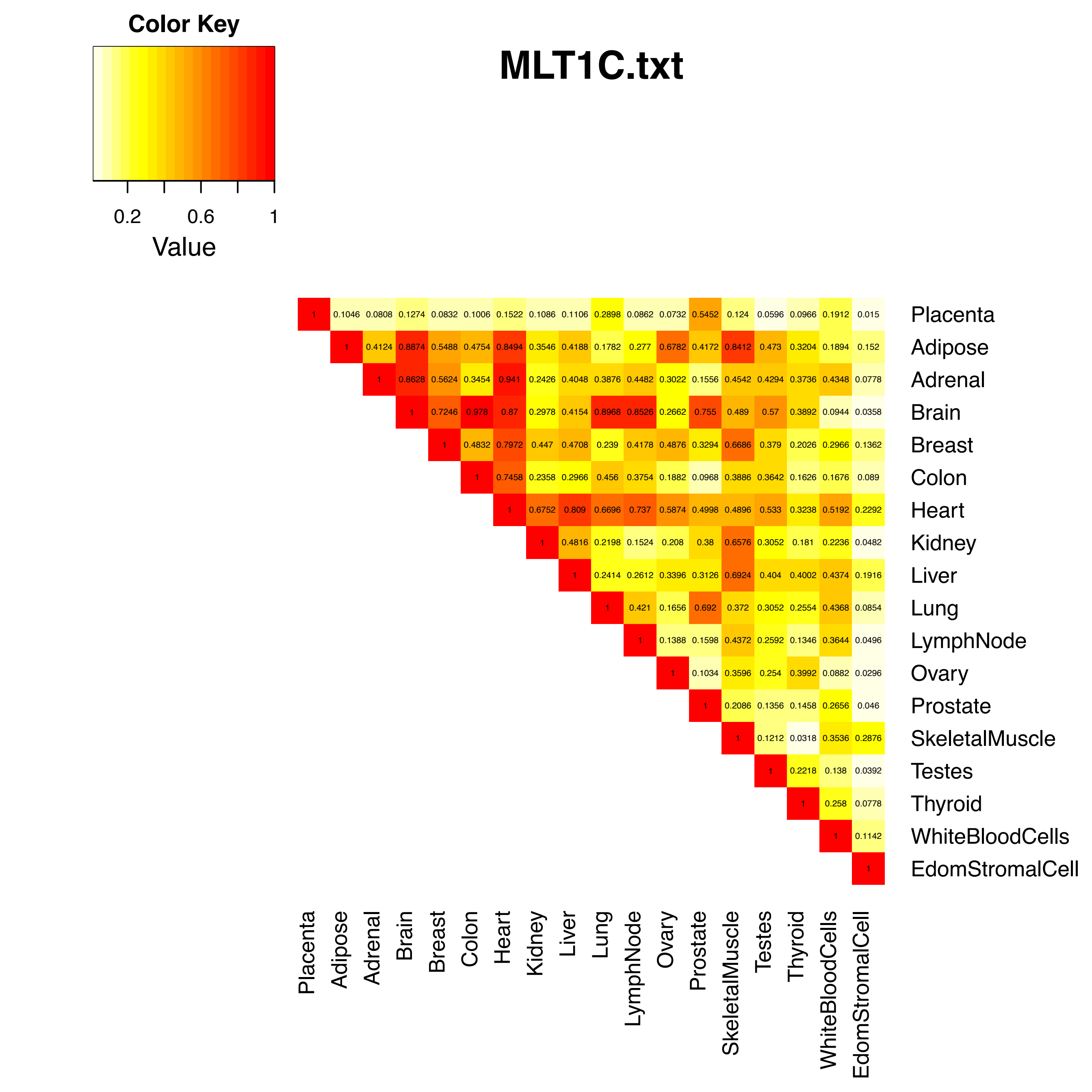

Color Key
0.2
0.6
1
Value
MLT1C.txt
Placenta
Adipose
Adrenal
Brain
Breast
Colon
Heart
Kidney
Liver
Lung
LymphNode
Ovary
Prostate
SkeletalMuscle
Testes
Thyroid
WhiteBloodCells
EdomStromalCell
1 0.1046 0.0808 0.1274 0.0832 0.1006 0.1522 0.1086 0.1106 0.2898 0.0862 0.0732 0.5452 0.124 0.0596 0.0966 0.1912 0.015
1 0.4124 0.8874 0.5488 0.4754 0.8494 0.3546 0.4188 0.1782 0.277 0.6782 0.4172 0.8412 0.473 0.3204 0.1894 0.152
1 0.8628 0.5624 0.3454 0.941 0.2426 0.4048 0.3876 0.4482 0.3022 0.1556 0.4542 0.4294 0.3736 0.4348 0.0778
1 0.7246 0.978 0.87 0.2978 0.4154 0.8968 0.8526 0.2662 0.755 0.489 0.57 0.3892 0.0944 0.0358
1 0.4832 0.7972 0.447 0.4708 0.239 0.4178 0.4876 0.3294 0.6686 0.379 0.2026 0.2966 0.1362
1 0.7458 0.2358 0.2966 0.456 0.3754 0.1882 0.0968 0.3886 0.3642 0.1626 0.1676 0.089
1 0.6752 0.809 0.6696 0.737 0.5874 0.4998 0.4896 0.533 0.3238 0.5192 0.2292
1 0.4816 0.2198 0.1524 0.208 0.38 0.6576 0.3052 0.181 0.2236 0.0482
1 0.2414 0.2612 0.3396 0.3126 0.6924 0.404 0.4002 0.4374 0.1916
1 0.421 0.1656 0.692 0.372 0.3052 0.2554 0.4368 0.0854
1 0.1388 0.1598 0.4372 0.2592 0.1346 0.3644 0.0496
1 0.1034 0.3596 0.254 0.3992 0.0882 0.0296
1 0.2086 0.1356 0.1458 0.2656 0.046
1 0.1212 0.0318 0.3536 0.2876
1 0.2218 0.138 0.0392
1 0.258 0.0778
1 0.1142
1

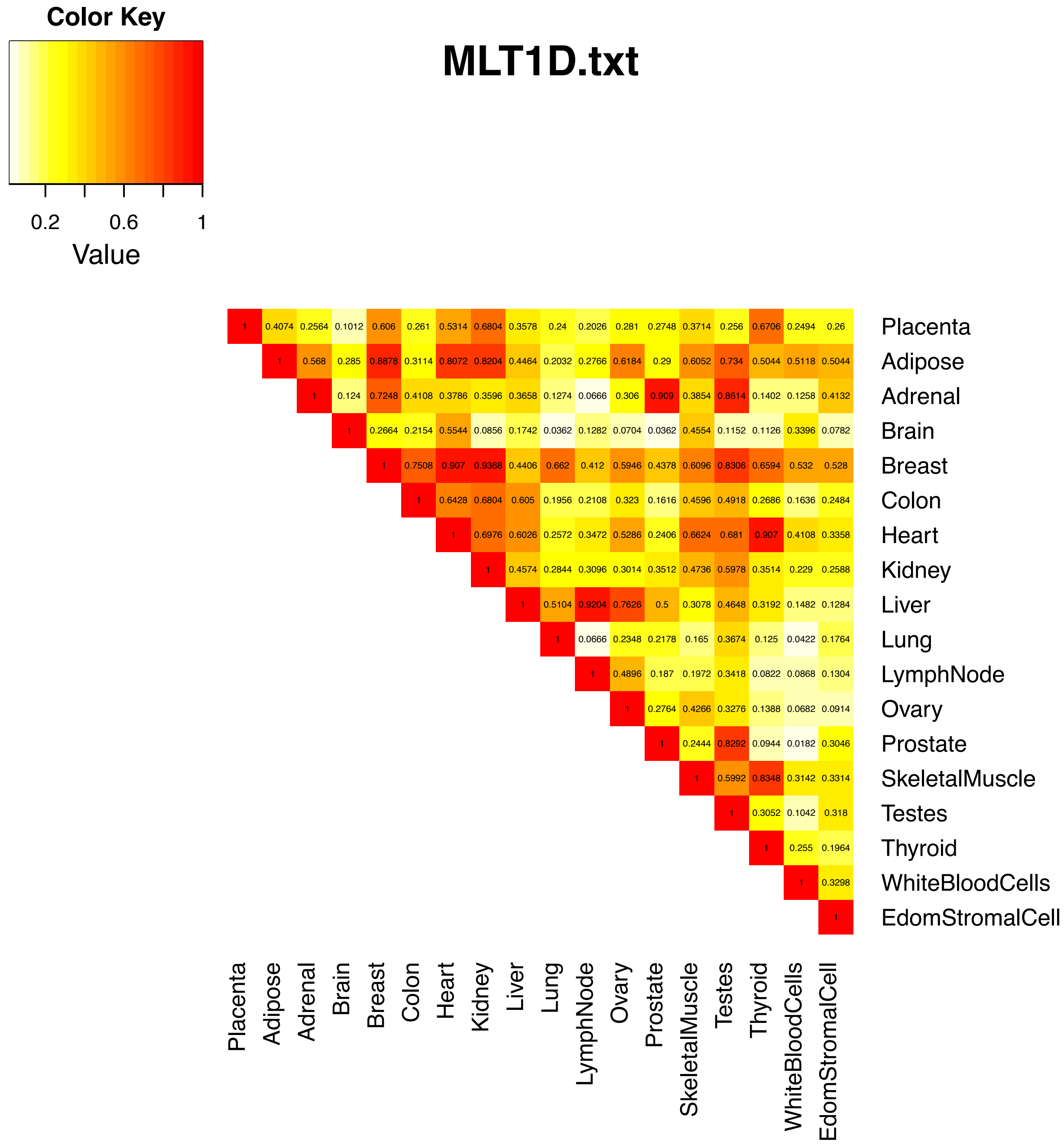
Color Key
0.2
0.6
1
Value
MLT1D.txt
Placenta
Adipose
Adrenal
Brain
Breast
Colon
Heart
Kidney
Liver
Lung
LymphNode
Ovary
Prostate
SkeletalMuscle
Testes
Thyroid
WhiteBloodCells
EdomStromalCell
1 0.4074 0.2564 0.1012 0.606 0.261 0.5314 0.6804 0.3578 0.24 0.2026 0.281 0.2748 0.3714 0.256 0.6706 0.2494 0.26
1 0.568 0.285 0.8878 0.3114 0.8072 0.8204 0.4464 0.2032 0.2766 0.6184 0.29 0.6052 0.734 0.5044 0.5118 0.5044
1 0.124 0.7248 0.4108 0.3786 0.3596 0.3658 0.1274 0.0666 0.306 0.909 0.3854 0.8614 0.1402 0.1258 0.4132
1 0.2664 0.2154 0.5544 0.0856 0.1742 0.0362 0.1282 0.0704 0.0362 0.4554 0.1152 0.1126 0.3396 0.0782
1 0.7508 0.907 0.9368 0.4406 0.662 0.412 0.5946 0.4378 0.6096 0.8306 0.6594 0.532 0.528
1 0.6428 0.6804 0.605 0.1956 0.2108 0.323 0.1616 0.4596 0.4918 0.2686 0.1636 0.2484
1 0.6976 0.6026 0.2572 0.3472 0.5286 0.2406 0.6624 0.681 0.907 0.4108 0.3358
1 0.4574 0.2844 0.3096 0.3014 0.3512 0.4736 0.5978 0.3514 0.229 0.2588
1 0.5104 0.9204 0.7626 0.5 0.3078 0.4648 0.3192 0.1482 0.1284
1 0.0666 0.2348 0.2178 0.165 0.3674 0.125 0.0422 0.1764
1 0.4896 0.187 0.1972 0.3418 0.0822 0.0868 0.1304
1 0.2764 0.4266 0.3276 0.1388 0.0682 0.0914
1 0.2444 0.8292 0.0944 0.0182 0.3046
1 0.5992 0.8348 0.3142 0.3314
1 0.3052 0.1042 0.318
1 0.255 0.1964
1 0.3298
1
Placenta
Adipose
Adrenal
Brain
Breast
Colon
Heart
Kidney
Liver
Lung
LymphNode
Ovary
Prostate
SkeletalMuscle
Testes
Thyroid
WhiteBloodCells
EdomStromalCell

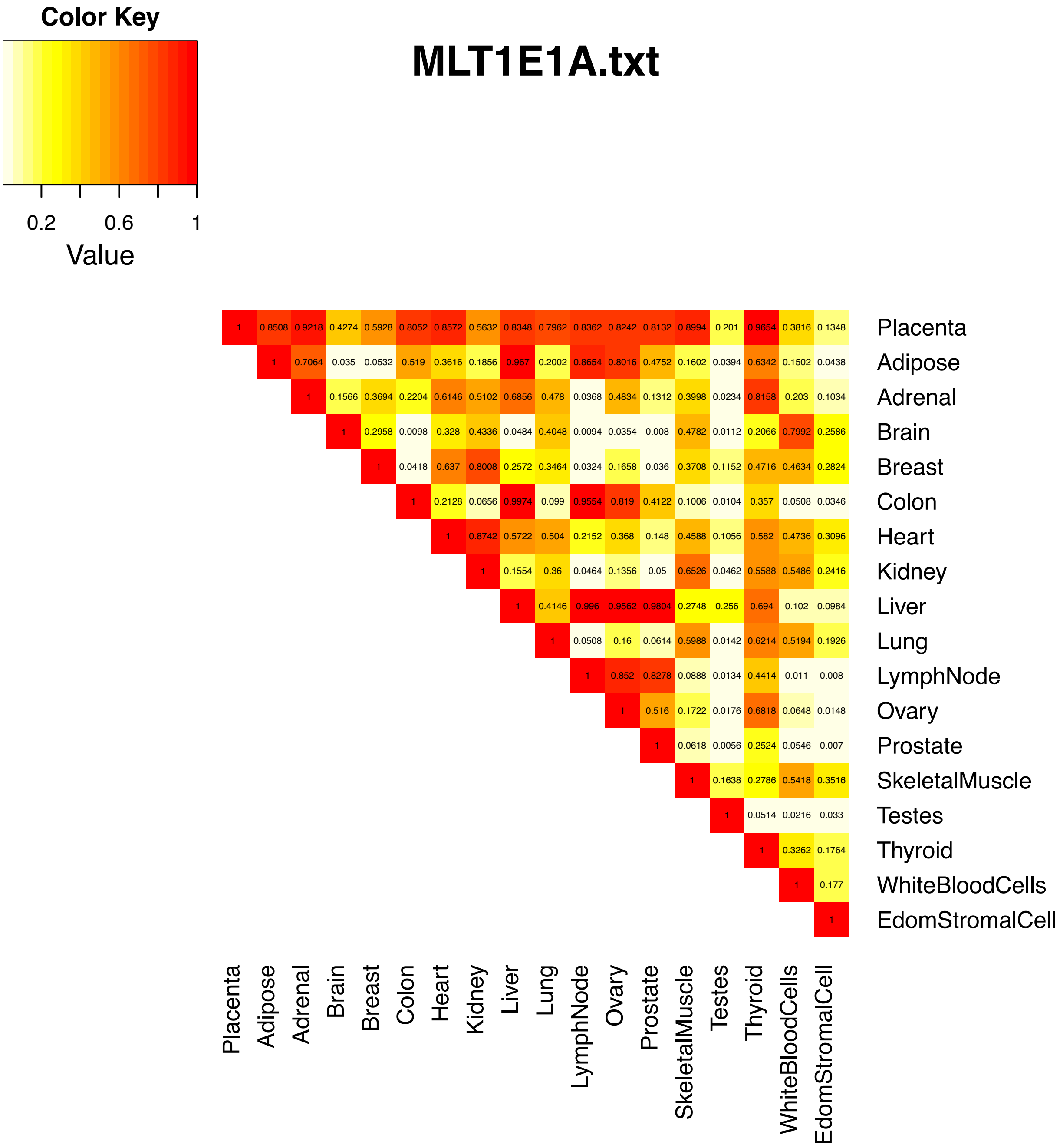

Color Key
0.2
0.6
1
Value
MLT1E1A.txt
Placenta 1 0.8508 0.9218 0.4274 0.5928 0.8052 0.8572 0.5632 0.8348 0.7962 0.8362 0.8242 0.8132 0.8994 0.201 0.9654 0.3816 0.1348
Adipose 1 0.7064 0.035 0.0532 0.519 0.3616 0.1856 0.967 0.2002 0.8654 0.8016 0.4752 0.1602 0.0394 0.6342 0.1502 0.0438
Adrenal 1 0.1566 0.3694 0.2204 0.6146 0.5102 0.6856 0.478 0.0368 0.4834 0.1312 0.3998 0.0234 0.8158 0.203 0.1034
Brain 1 0.2958 0.0098 0.328 0.4336 0.0484 0.4048 0.0094 0.0354 0.008 0.4782 0.0112 0.2066 0.7992 0.2586
Breast 1 0.0418 0.637 0.8008 0.2572 0.3464 0.0324 0.1658 0.036 0.3708 0.1152 0.4716 0.4634 0.2824
Colon 1 0.2128 0.0656 0.9974 0.099 0.9554 0.819 0.4122 0.1006 0.0104 0.357 0.0508 0.0346
Heart 1 0.8742 0.5722 0.504 0.2152 0.368 0.148 0.4588 0.1056 0.582 0.4736 0.3096
Kidney 1 0.1554 0.36 0.0464 0.1356 0.05 0.6526 0.0462 0.5588 0.5486 0.2416
Liver 1 0.4146 0.996 0.9562 0.9804 0.2748 0.256 0.694 0.102 0.0984
Lung 1 0.0508 0.16 0.0614 0.5988 0.0142 0.6214 0.5194 0.1926
LymphNode 1 0.852 0.8278 0.0888 0.0134 0.4414 0.011 0.008
Ovary 1 0.516 0.1722 0.0176 0.6818 0.0648 0.0148
Prostate 1 0.0618 0.0056 0.2524 0.0546 0.007
SkeletalMuscle 1 0.1638 0.2786 0.5418 0.3516
Testes 1 0.0514 0.0216 0.033
Thyroid 1 0.3262 0.1764
WhiteBloodCells 1 0.177
EdomStromalCell 1
Placenta
Adipose
Adrenal
Brain
Breast
Colon
Heart
Kidney
Liver
Lung
LymphNode
Ovary
Prostate
SkeletalMuscle
Testes
Thyroid
WhiteBloodCells
EdomStromalCell

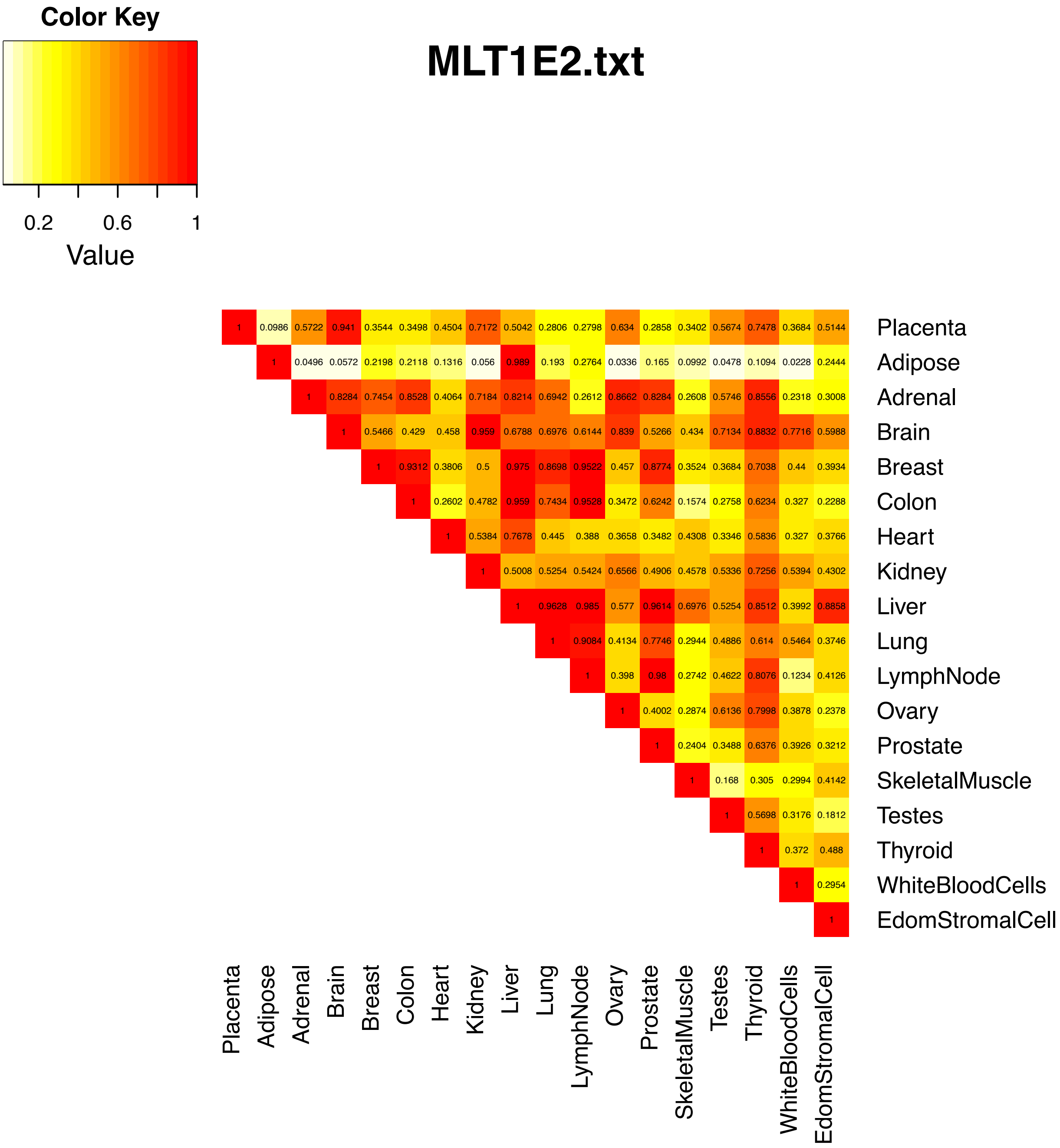
Color Key
0.2
0.6
1
Value
MLT1E2.txt
Placenta: 1 0.0986 0.5722 0.941 0.3544 0.3498 0.4504 0.7172 0.5042 0.2806 0.2798 0.634 0.2858 0.3402 0.5674 0.7478 0.3684 0.5144
Adipose: 1 0.0496 0.0572 0.2198 0.2118 0.1316 0.056 0.989 0.193 0.2764 0.0336 0.165 0.0992 0.0478 0.1094 0.0228 0.2444
Adrenal: 1 0.8284 0.7454 0.8528 0.4064 0.7184 0.8214 0.6942 0.2612 0.8662 0.8284 0.2608 0.5746 0.8556 0.2318 0.3008
Brain: 1 0.5466 0.429 0.458 0.959 0.6788 0.6976 0.6144 0.839 0.5266 0.434 0.7134 0.8832 0.7716 0.5988
Breast: 1 0.9312 0.3806 0.5 0.975 0.8698 0.9522 0.457 0.8774 0.3524 0.3684 0.7038 0.44 0.3934
Colon: 1 0.2602 0.4782 0.959 0.7434 0.9528 0.3472 0.6242 0.1574 0.2758 0.6234 0.327 0.2288
Heart: 1 0.5384 0.7678 0.445 0.388 0.3658 0.3482 0.4308 0.3346 0.5836 0.327 0.3766
Kidney: 1 0.5008 0.5254 0.5424 0.6566 0.4906 0.4578 0.5336 0.7256 0.5394 0.4302
Liver: 1 0.9628 0.985 0.577 0.9614 0.6976 0.5254 0.8512 0.3992 0.8858
Lung: 1 0.9084 0.4134 0.7746 0.2944 0.4886 0.614 0.5464 0.3746
LymphNode: 1 0.398 0.98 0.2742 0.4622 0.8076 0.1234 0.4126
Ovary: 1 0.4002 0.2874 0.6136 0.7998 0.3878 0.2378
Prostate: 1 0.2404 0.3488 0.6376 0.3926 0.3212
SkeletalMuscle: 1 0.168 0.305 0.2994 0.4142
Testes: 1 0.5698 0.3176 0.1812
Thyroid: 1 0.372 0.488
WhiteBloodCells: 1 0.2954
EdomStromalCell: 1
Placenta
Adipose
Adrenal
Brain
Breast
Colon
Heart
Kidney
Liver
Lung
LymphNode
Ovary
Prostate
SkeletalMuscle
Testes
Thyroid
WhiteBloodCells
EdomStromalCell

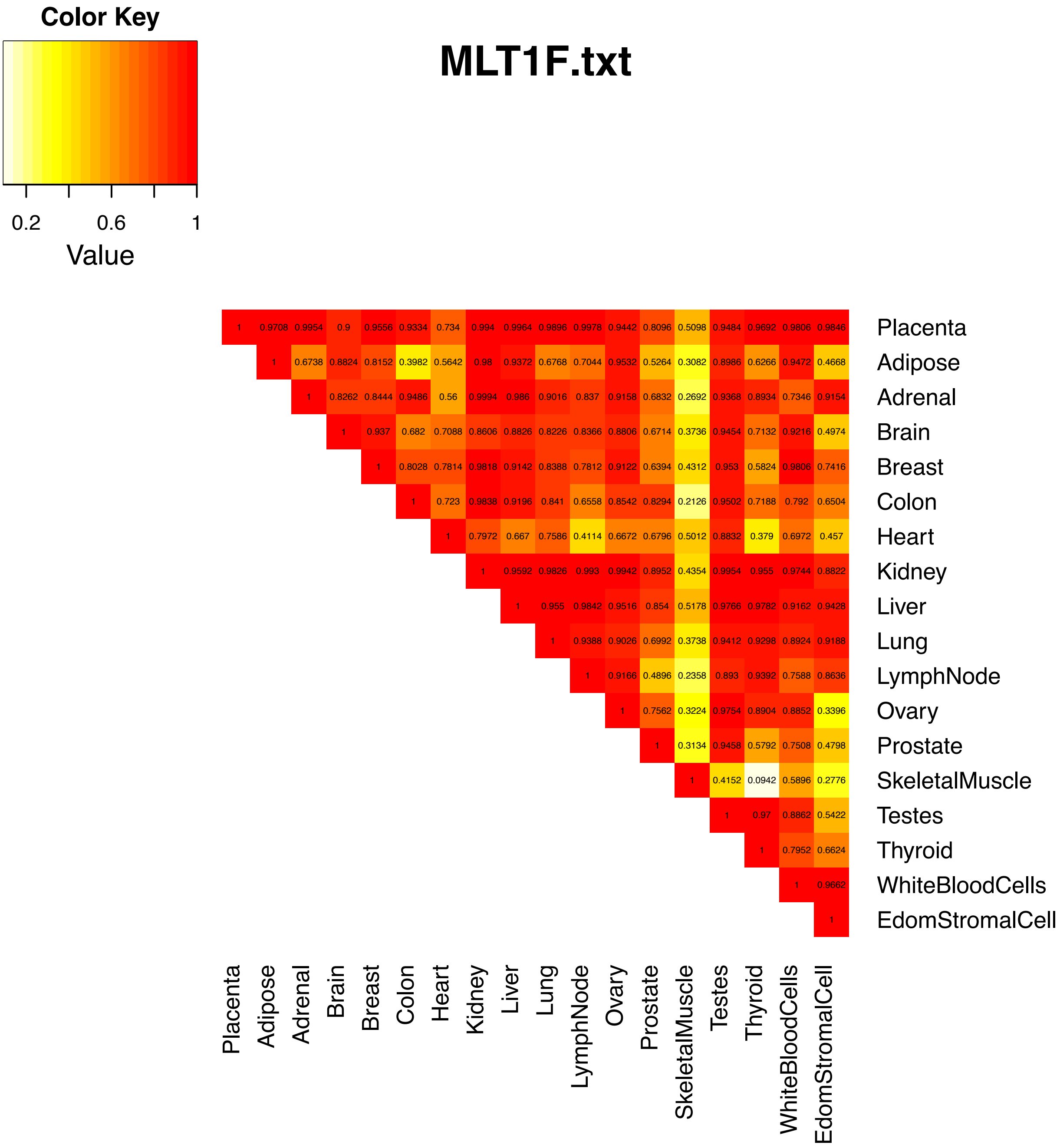
Color Key
0.2
0.6
1
Value
MLT1F.txt
1 0.9708 0.9954 0.9 0.9556 0.9334 0.734 0.994 0.9964 0.9896 0.9978 0.9442 0.8096 0.5098 0.9484 0.9692 0.9806 0.9846 Placenta
1 0.6738 0.8824 0.8152 0.3982 0.5642 0.98 0.9372 0.6768 0.7044 0.9532 0.5264 0.3082 0.8986 0.6266 0.9472 0.4668 Adipose
1 0.8262 0.8444 0.9486 0.56 0.9994 0.986 0.9016 0.837 0.9158 0.6832 0.2692 0.9368 0.8934 0.7346 0.9154 Adrenal
1 0.937 0.682 0.7088 0.8606 0.8826 0.8226 0.8366 0.8806 0.6714 0.3736 0.9454 0.7132 0.9216 0.4974 Brain
1 0.8028 0.7814 0.9818 0.9142 0.8388 0.7812 0.9122 0.6394 0.4312 0.953 0.5824 0.9806 0.7416 Breast
1 0.723 0.9838 0.9196 0.841 0.6558 0.8542 0.8294 0.2126 0.9502 0.7188 0.792 0.6504 Colon
1 0.7972 0.667 0.7586 0.4114 0.6672 0.6796 0.5012 0.8832 0.379 0.6972 0.457 Heart
1 0.9592 0.9826 0.993 0.9942 0.8952 0.4354 0.9954 0.955 0.9744 0.8822 Kidney
1 0.955 0.9842 0.9516 0.854 0.5178 0.9766 0.9782 0.9162 0.9428 Liver
1 0.9388 0.9026 0.6992 0.3738 0.9412 0.9298 0.8924 0.9188 Lung
1 0.9166 0.4896 0.2358 0.893 0.9392 0.7588 0.8636 LymphNode
1 0.7562 0.3224 0.9754 0.8904 0.8852 0.3396 Ovary
1 0.3134 0.9458 0.5792 0.7508 0.4798 Prostate
1 0.4152 0.0942 0.5896 0.2776 SkeletalMuscle
1 0.97 0.8862 0.5422 Testes
1 0.7952 0.6624 Thyroid
1 0.9662 WhiteBloodCells
1 EdomStromalCell
Placenta
Adipose
Adrenal
Brain
Breast
Colon
Heart
Kidney
Liver
Lung
LymphNode
Ovary
Prostate
SkeletalMuscle
Testes
Thyroid
WhiteBloodCells
EdomStromalCell

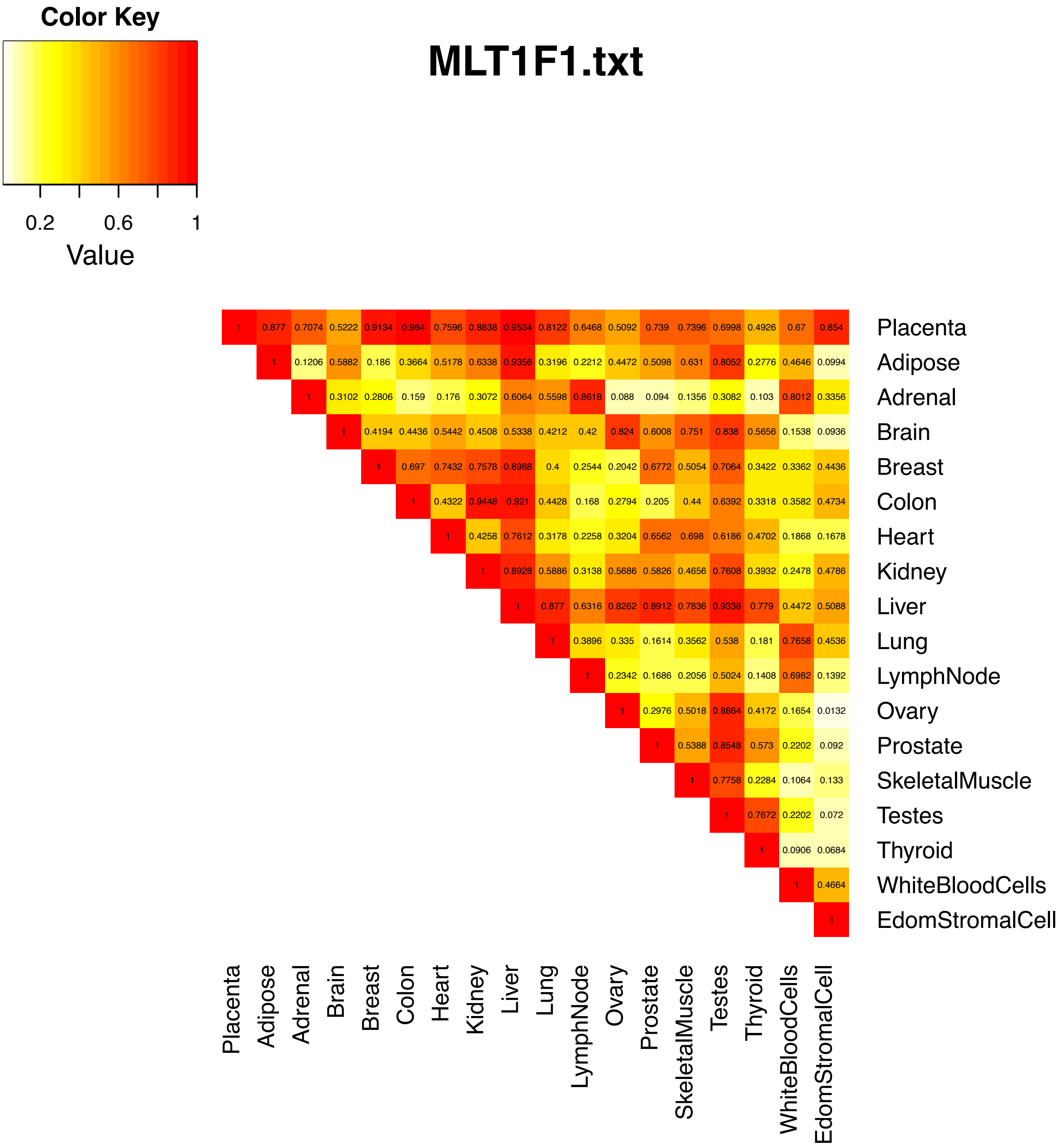
Color Key
0.2
0.6
1
Value
MLT1F1.txt
Placenta 1 0.877 0.7074 0.5222 0.9134 0.964 0.7596 0.8838 0.9534 0.8122 0.6468 0.5092 0.739 0.7396 0.6998 0.4926 0.67 0.854
Adipose 1 0.1206 0.5882 0.186 0.3664 0.5178 0.6338 0.9356 0.3196 0.2212 0.4472 0.5098 0.631 0.8052 0.2776 0.4646 0.0994
Adrenal 1 0.3102 0.2806 0.159 0.176 0.3072 0.6064 0.5598 0.8618 0.088 0.094 0.1356 0.3082 0.103 0.8012 0.3356
Brain 1 0.4194 0.4436 0.5442 0.4508 0.5338 0.4212 0.42 0.824 0.6008 0.751 0.838 0.5656 0.1538 0.0936
Breast 1 0.697 0.7432 0.7578 0.8988 0.4 0.2544 0.2042 0.6772 0.5054 0.7064 0.3422 0.3362 0.4436
Colon 1 0.4322 0.9448 0.921 0.4428 0.168 0.2794 0.205 0.44 0.6392 0.3318 0.3582 0.4734
Heart 1 0.4258 0.7612 0.3178 0.2258 0.3204 0.6562 0.698 0.6186 0.4702 0.1868 0.1678
Kidney 1 0.8928 0.5886 0.3138 0.5686 0.5826 0.4656 0.7608 0.3932 0.2478 0.4786
Liver 1 0.877 0.6316 0.8262 0.8912 0.7836 0.9338 0.779 0.4472 0.5088
Lung 1 0.3896 0.335 0.1614 0.3562 0.538 0.181 0.7658 0.4536
LymphNode 1 0.2342 0.1686 0.2056 0.5024 0.1408 0.6982 0.1392
Ovary 1 0.2976 0.5018 0.8864 0.4172 0.1654 0.0132
Prostate 1 0.5388 0.8548 0.573 0.2202 0.092
SkeletalMuscle 1 0.7758 0.2284 0.1064 0.133
Testes 1 0.7672 0.2202 0.072
Thyroid 1 0.0906 0.0684
WhiteBloodCells 1 0.4664
EdomStromalCell 1
Placenta Adipose Adrenal Brain Breast Colon Heart Kidney Liver Lung LymphNode Ovary Prostate SkeletalMuscle Testes Thyroid WhiteBloodCells EdomStromalCell

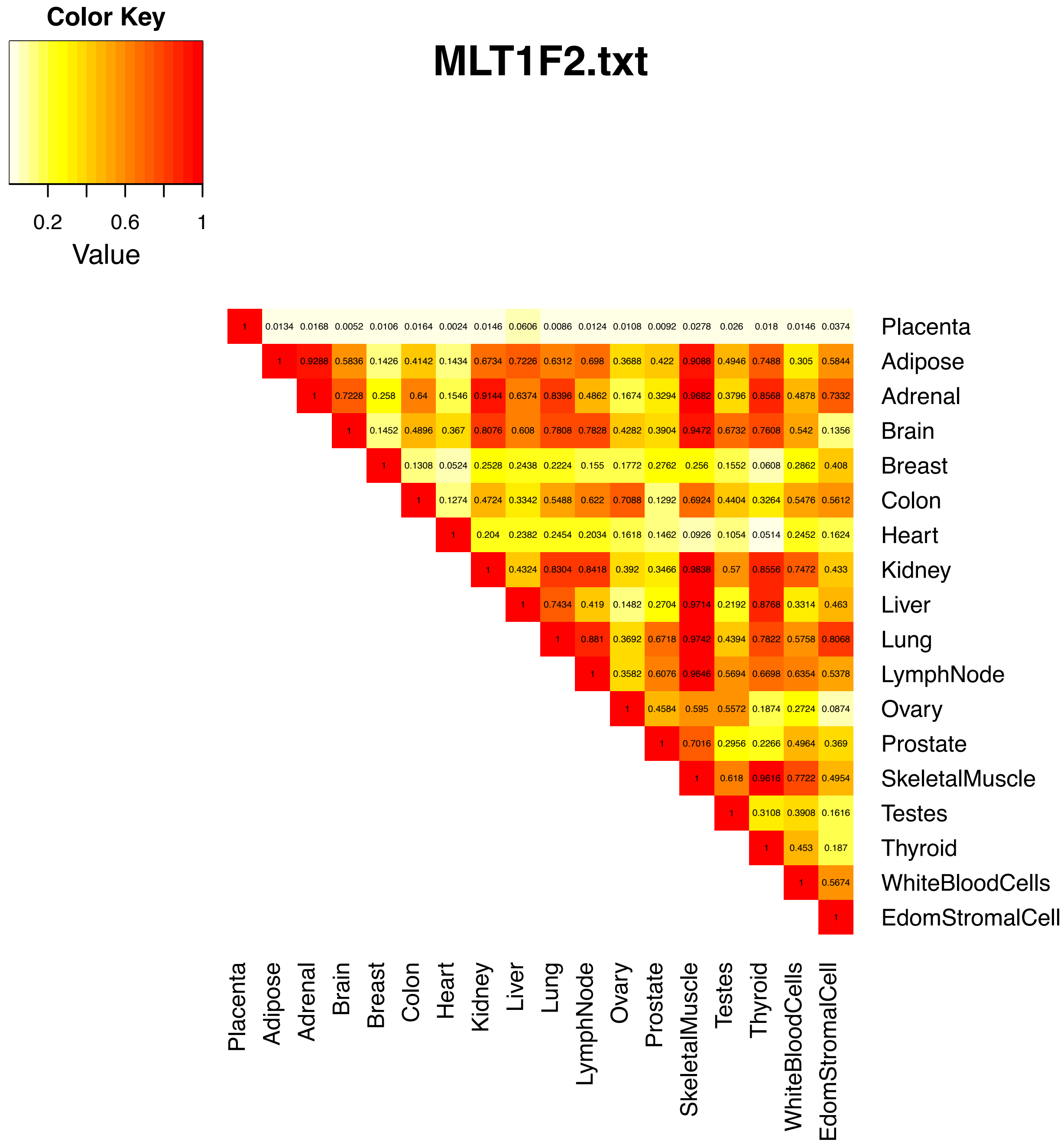
Color Key
0.2
0.6
1
Value
MLT1F2.txt
Placenta 1 0.0134 0.0168 0.0052 0.0106 0.0164 0.0024 0.0146 0.0606 0.0086 0.0124 0.0108 0.0092 0.0278 0.026 0.018 0.0146 0.0374
Adipose 1 0.9288 0.5836 0.1426 0.4142 0.1434 0.6734 0.7226 0.6312 0.698 0.3688 0.422 0.9088 0.4946 0.7488 0.305 0.5844
Adrenal 1 0.7228 0.258 0.64 0.1546 0.9144 0.6374 0.8396 0.4862 0.1674 0.3294 0.9682 0.3796 0.8568 0.4878 0.7332
Brain 1 0.1452 0.4896 0.367 0.8076 0.608 0.7808 0.7828 0.4282 0.3904 0.9472 0.6732 0.7608 0.542 0.1356
Breast 1 0.1308 0.0524 0.2528 0.2438 0.2224 0.155 0.1772 0.2762 0.256 0.1552 0.0608 0.2862 0.408
Colon 1 0.1274 0.4724 0.3342 0.5488 0.622 0.7088 0.1292 0.6924 0.4404 0.3264 0.5476 0.5612
Heart 1 0.204 0.2382 0.2454 0.2034 0.1618 0.1462 0.0926 0.1054 0.0514 0.2452 0.1624
Kidney 1 0.4324 0.8304 0.8418 0.392 0.3466 0.9838 0.57 0.8556 0.7472 0.433
Liver 1 0.7434 0.419 0.1482 0.2704 0.9714 0.2192 0.8768 0.3314 0.463
Lung 1 0.881 0.3692 0.6718 0.9742 0.4394 0.7822 0.5758 0.8068
LymphNode 1 0.3582 0.6076 0.9646 0.5694 0.6698 0.6354 0.5378
Ovary 1 0.4584 0.595 0.5572 0.1874 0.2724 0.0874
Prostate 1 0.7016 0.2956 0.2266 0.4964 0.369
SkeletalMuscle 1 0.618 0.9616 0.7722 0.4954
Testes 1 0.3108 0.3908 0.1616
Thyroid 1 0.453 0.187
WhiteBloodCells 1 0.5674
EdomStromalCell 1
Placenta
Adipose
Adrenal
Brain
Breast
Colon
Heart
Kidney
Liver
Lung
LymphNode
Ovary
Prostate
SkeletalMuscle
Testes
Thyroid
WhiteBloodCells
EdomStromalCell

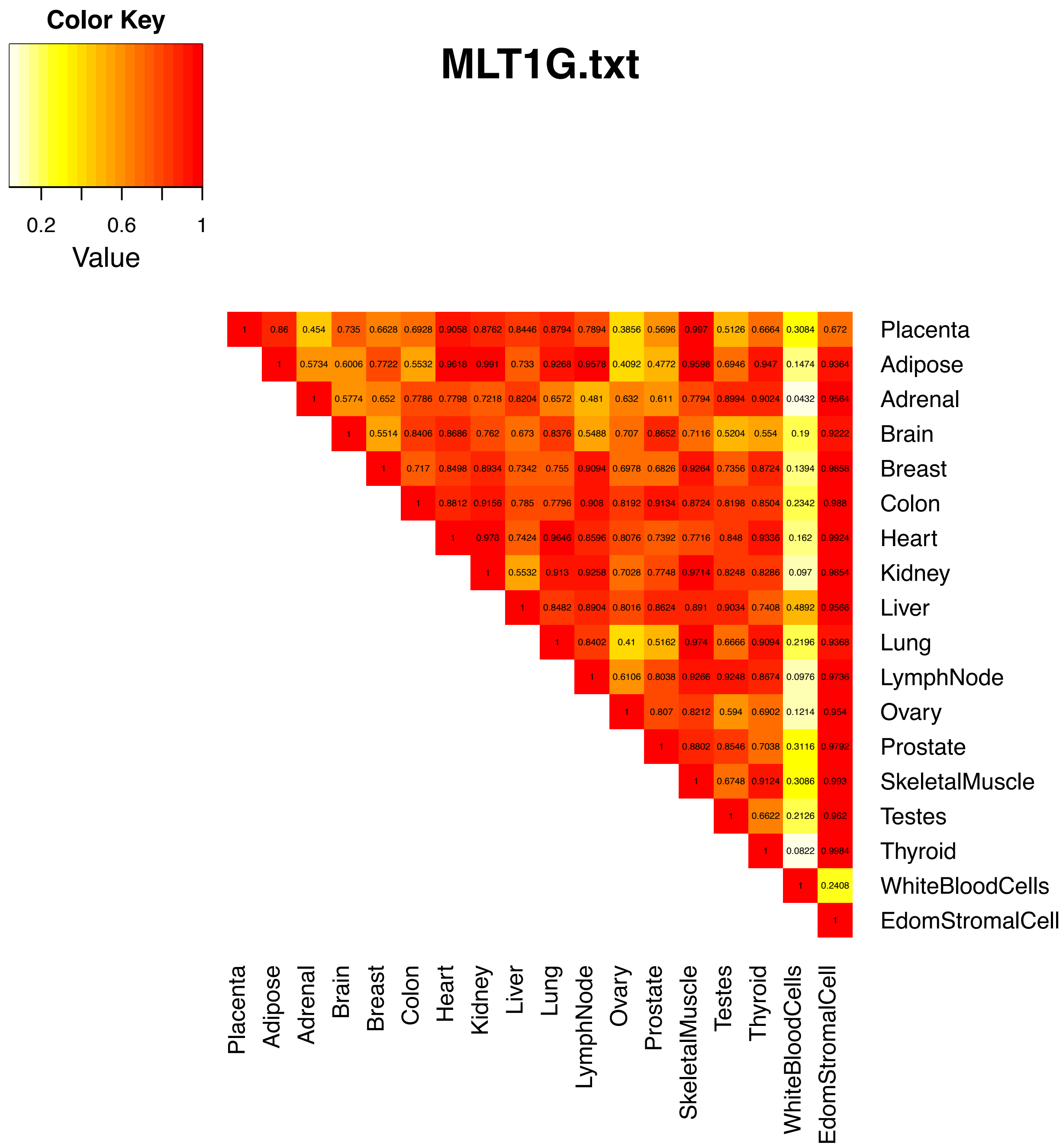
Color Key
0.2
0.6
1
Value
MLT1G.txt
Placenta 1 0.86 0.454 0.735 0.6628 0.6928 0.9058 0.8762 0.8446 0.8794 0.7894 0.3856 0.5696 0.997 0.5126 0.6664 0.3084 0.672
Adipose 1 0.5734 0.6006 0.7722 0.5532 0.9618 0.991 0.733 0.9268 0.9578 0.4092 0.4772 0.9598 0.6946 0.947 0.1474 0.9364
Adrenal 1 0.5774 0.652 0.7786 0.7798 0.7218 0.8204 0.6572 0.481 0.632 0.611 0.7794 0.8994 0.9024 0.0432 0.9564
Brain 1 0.5514 0.8406 0.8686 0.762 0.673 0.8376 0.5488 0.707 0.8652 0.7116 0.5204 0.554 0.19 0.9222
Breast 1 0.717 0.8498 0.8934 0.7342 0.755 0.9094 0.6978 0.6826 0.9264 0.7356 0.8724 0.1394 0.9858
Colon 1 0.8812 0.9156 0.785 0.7796 0.908 0.8192 0.9134 0.8724 0.8198 0.8504 0.2342 0.988
Heart 1 0.978 0.7424 0.9646 0.8596 0.8076 0.7392 0.7716 0.848 0.9336 0.162 0.9924
Kidney 1 0.5532 0.913 0.9258 0.7028 0.7748 0.9714 0.8248 0.8286 0.097 0.9854
Liver 1 0.8482 0.8904 0.8016 0.8624 0.891 0.9034 0.7408 0.4892 0.9566
Lung 1 0.8402 0.41 0.5162 0.974 0.6666 0.9094 0.2196 0.9368
LymphNode 1 0.6106 0.8038 0.9266 0.9248 0.8674 0.0976 0.9736
Ovary 1 0.807 0.8212 0.594 0.6902 0.1214 0.954
Prostate 1 0.8802 0.8546 0.7038 0.3116 0.9792
SkeletalMuscle 1 0.6748 0.9124 0.3086 0.993
Testes 1 0.6622 0.2126 0.962
Thyroid 1 0.0822 0.9984
WhiteBloodCells 1 0.2408
EdomStromalCell 1
Placenta
Adipose
Adrenal
Brain
Breast
Colon
Heart
Kidney
Liver
Lung
LymphNode
Ovary
Prostate
SkeletalMuscle
Testes
Thyroid
WhiteBloodCells
EdomStromalCell

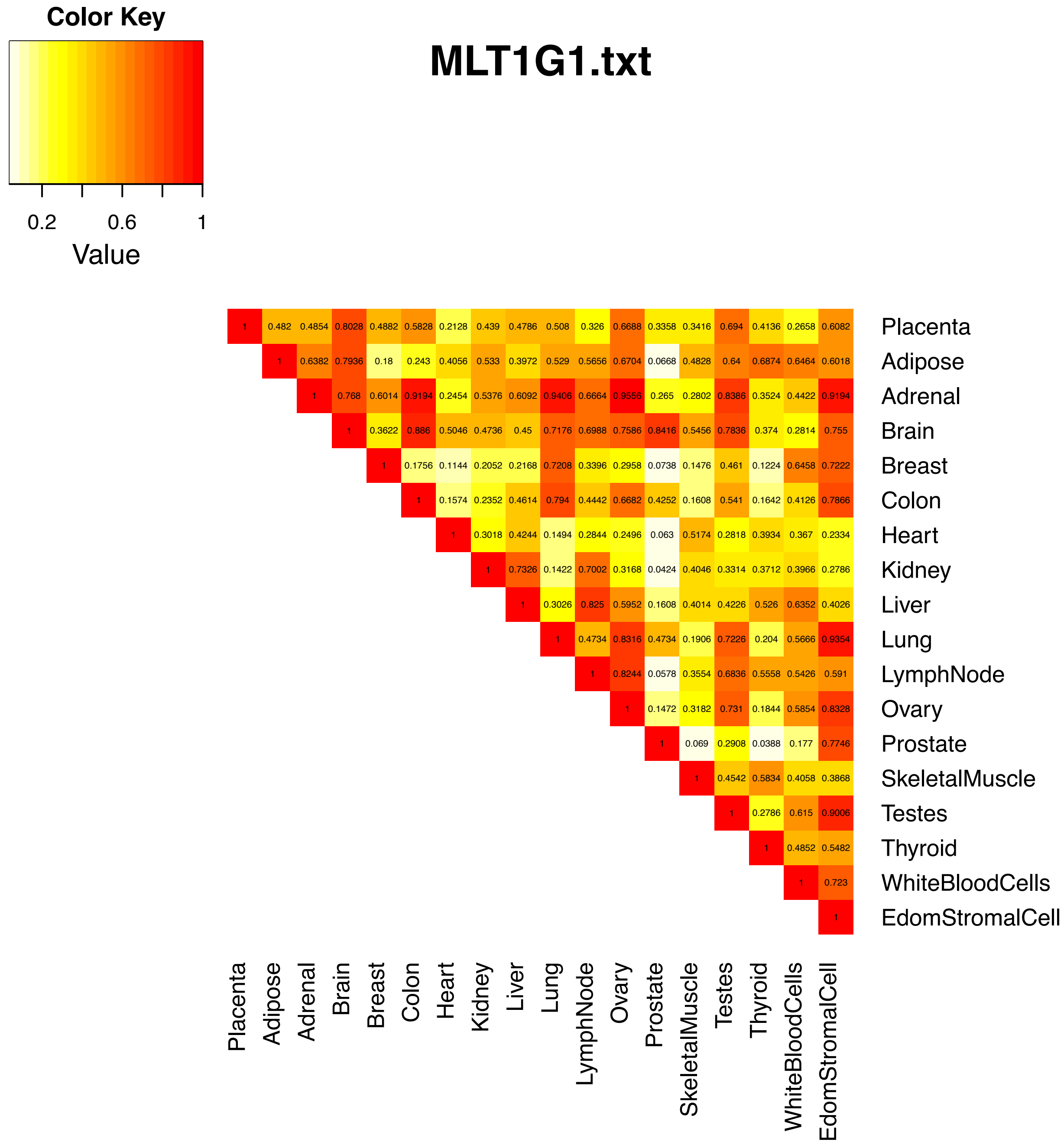
Color Key
0.2
0.6
1
Value
MLT1G1.txt
1 0.482 0.4854 0.8028 0.4882 0.5828 0.2128 0.439 0.4786 0.508 0.326 0.6688 0.3358 0.3416 0.694 0.4136 0.2658 0.6082 Placenta
1 0.6382 0.7936 0.18 0.243 0.4056 0.533 0.3972 0.529 0.5656 0.6704 0.0668 0.4828 0.64 0.6874 0.6464 0.6018 Adipose
1 0.768 0.6014 0.9194 0.2454 0.5376 0.6092 0.9406 0.6664 0.9556 0.265 0.2802 0.8386 0.3524 0.4422 0.9194 Adrenal
1 0.3622 0.886 0.5046 0.4736 0.45 0.7176 0.6988 0.7586 0.8416 0.5456 0.7836 0.374 0.2814 0.755 Brain
1 0.1756 0.1144 0.2052 0.2168 0.7208 0.3396 0.2958 0.0738 0.1476 0.461 0.1224 0.6458 0.7222 Breast
1 0.1574 0.2352 0.4614 0.794 0.4442 0.6682 0.4252 0.1608 0.541 0.1642 0.4126 0.7866 Colon
1 0.3018 0.4244 0.1494 0.2844 0.2496 0.063 0.5174 0.2818 0.3934 0.367 0.2334 Heart
1 0.7326 0.1422 0.7002 0.3168 0.0424 0.4046 0.3314 0.3712 0.3966 0.2786 Kidney
1 0.3026 0.825 0.5952 0.1608 0.4014 0.4226 0.526 0.6352 0.4026 Liver
1 0.4734 0.8316 0.4734 0.1906 0.7226 0.204 0.5666 0.9354 Lung
1 0.8244 0.0578 0.3554 0.6836 0.5558 0.5426 0.591 LymphNode
1 0.1472 0.3182 0.731 0.1844 0.5854 0.8328 Ovary
1 0.069 0.2908 0.0388 0.177 0.7746 Prostate
1 0.4542 0.5834 0.4058 0.3868 SkeletalMuscle
1 0.2786 0.615 0.9006 Testes
1 0.4852 0.5482 Thyroid
1 0.723 WhiteBloodCells
1 EdomStromalCell
Placenta Adipose Adrenal Brain Breast Colon Heart Kidney Liver Lung LymphNode Ovary Prostate SkeletalMuscle Testes Thyroid WhiteBloodCells EdomStromalCell

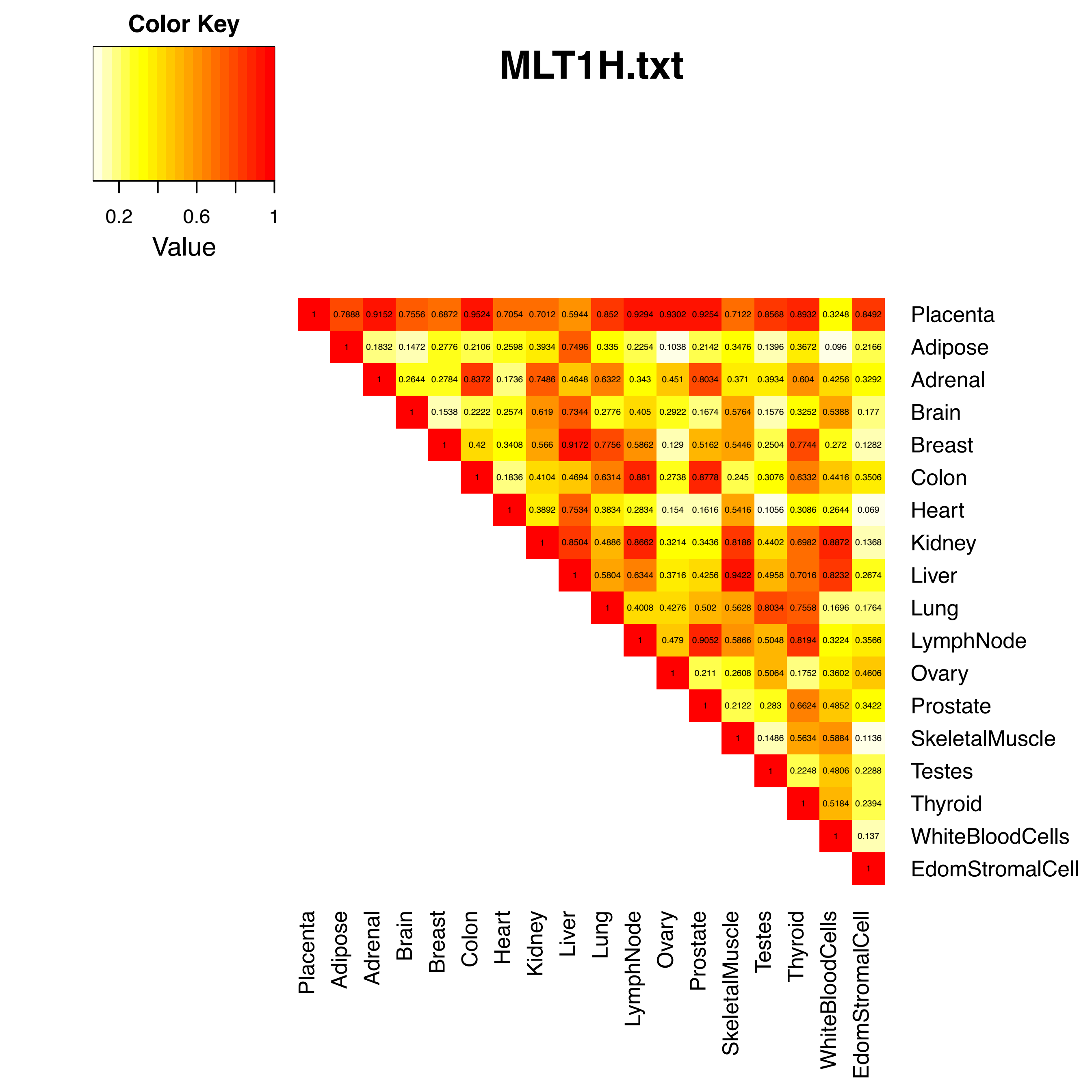
Color Key
0.2
0.6
1
Value
MLT1H.txt
1 0.7888 0.9152 0.7556 0.6872 0.9524 0.7054 0.7012 0.5944 0.852 0.9294 0.9302 0.9254 0.7122 0.8568 0.8932 0.3248 0.8492 Placenta
1 0.1832 0.1472 0.2776 0.2106 0.2598 0.3934 0.7496 0.335 0.2254 0.1038 0.2142 0.3476 0.1396 0.3672 0.096 0.2166 Adipose
1 0.2644 0.2784 0.8372 0.1736 0.7486 0.4648 0.6322 0.343 0.451 0.8034 0.371 0.3934 0.604 0.4256 0.3292 Adrenal
1 0.1538 0.2222 0.2574 0.619 0.7344 0.2776 0.405 0.2922 0.1674 0.5764 0.1576 0.3252 0.5388 0.177 Brain
1 0.42 0.3408 0.566 0.9172 0.7756 0.5862 0.129 0.5162 0.5446 0.2504 0.7744 0.272 0.1282 Breast
1 0.1836 0.4104 0.4694 0.6314 0.881 0.2738 0.8778 0.245 0.3076 0.6332 0.4416 0.3506 Colon
1 0.3892 0.7534 0.3834 0.2834 0.154 0.1616 0.5416 0.1056 0.3086 0.2644 0.069 Heart
1 0.8504 0.4886 0.8662 0.3214 0.3436 0.8186 0.4402 0.6982 0.8872 0.1368 Kidney
1 0.5804 0.6344 0.3716 0.4256 0.9422 0.4958 0.7016 0.8232 0.2674 Liver
1 0.4008 0.4276 0.502 0.5628 0.8034 0.7558 0.1696 0.1764 Lung
1 0.479 0.9052 0.5866 0.5048 0.8194 0.3224 0.3566 LymphNode
1 0.211 0.2608 0.5064 0.1752 0.3602 0.4606 Ovary
1 0.2122 0.283 0.6624 0.4852 0.3422 Prostate
1 0.1486 0.5634 0.5884 0.1136 SkeletalMuscle
1 0.2248 0.4806 0.2288 Testes
1 0.5184 0.2394 Thyroid
1 0.137 WhiteBloodCells
1 EdomStromalCell
Placenta
Adipose
Adrenal
Brain
Breast
Colon
Heart
Kidney
Liver
Lung
LymphNode
Ovary
Prostate
SkeletalMuscle
Testes
Thyroid
WhiteBloodCells
EdomStromalCell

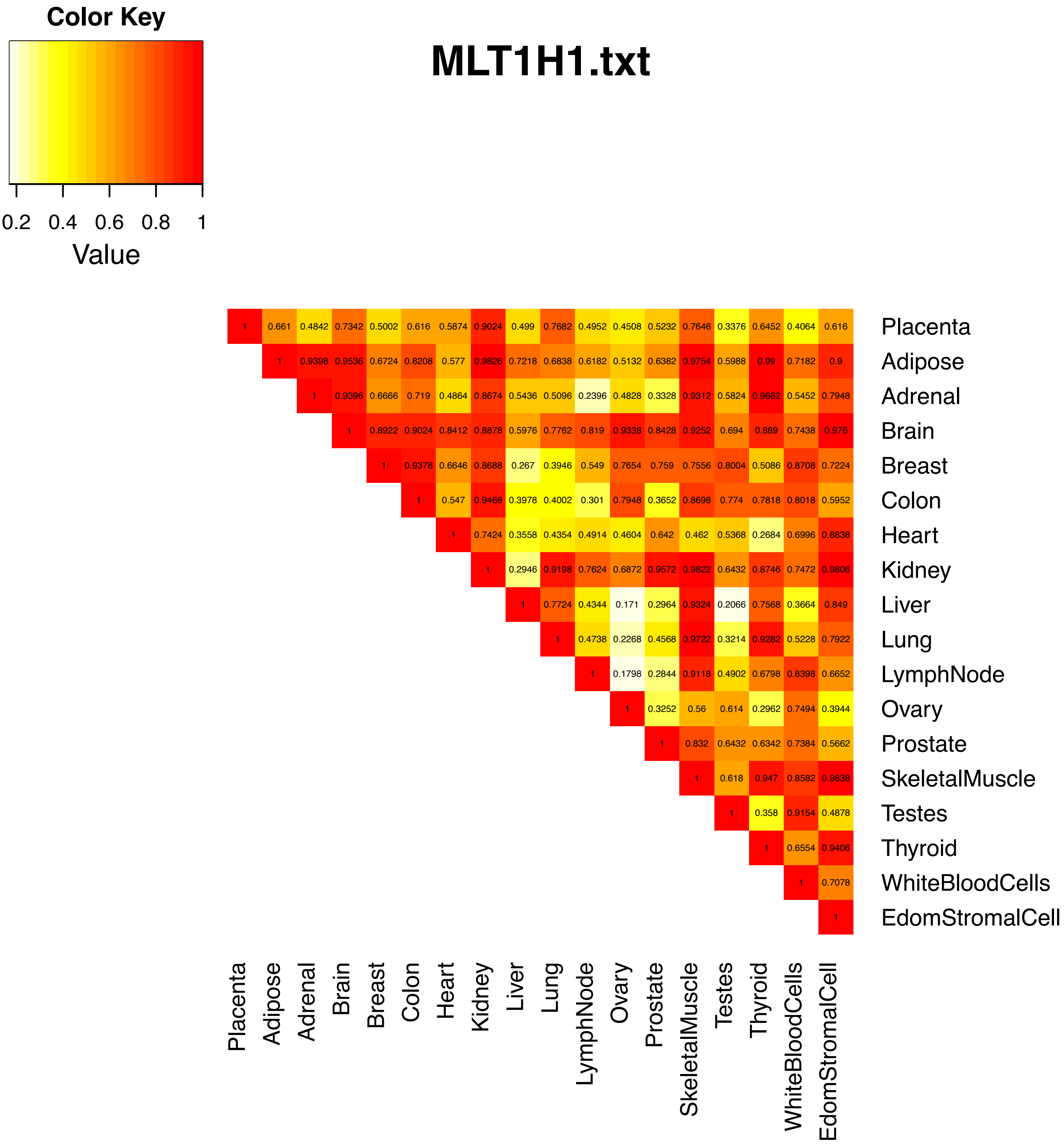
Color Key
0.2 0.4 0.6 0.8 1
Value
MLT1H1.txt
Placenta: 1 0.661 0.4842 0.7342 0.5002 0.616 0.5874 0.9024 0.499 0.7682 0.4952 0.4508 0.5232 0.7646 0.3376 0.6452 0.4064 0.616
Adipose: 1 0.9398 0.9536 0.6724 0.8208 0.577 0.9826 0.7218 0.6838 0.6182 0.5132 0.6382 0.9754 0.5988 0.99 0.7182 0.9
Adrenal: 1 0.9396 0.6666 0.719 0.4864 0.8674 0.5436 0.5096 0.2396 0.4828 0.3328 0.9312 0.5824 0.9682 0.5452 0.7948
Brain: 1 0.8922 0.9024 0.8412 0.8878 0.5976 0.7762 0.819 0.9338 0.8428 0.9252 0.694 0.889 0.7438 0.976
Breast: 1 0.9378 0.6646 0.8688 0.267 0.3946 0.549 0.7654 0.759 0.7556 0.8004 0.5086 0.8708 0.7224
Colon: 1 0.547 0.9468 0.3978 0.4002 0.301 0.7948 0.3652 0.8698 0.774 0.7818 0.8018 0.5952
Heart: 1 0.7424 0.3558 0.4354 0.4914 0.4604 0.642 0.462 0.5368 0.2684 0.6996 0.8838
Kidney: 1 0.2946 0.9198 0.7624 0.6872 0.9572 0.9822 0.6432 0.8746 0.7472 0.9806
Liver: 1 0.7724 0.4344 0.171 0.2964 0.9324 0.2066 0.7568 0.3664 0.849
Lung: 1 0.4738 0.2268 0.4568 0.9722 0.3214 0.9282 0.5228 0.7922
LymphNode: 1 0.1798 0.2844 0.9118 0.4902 0.6798 0.8398 0.6652
Ovary: 1 0.3252 0.56 0.614 0.2962 0.7494 0.3944
Prostate: 1 0.832 0.6432 0.6342 0.7384 0.5662
SkeletalMuscle: 1 0.618 0.947 0.8582 0.9838
Testes: 1 0.358 0.9154 0.4878
Thyroid: 1 0.6554 0.9406
WhiteBloodCells: 1 0.7078
EdomStromalCell: 1
Placenta Adipose Adrenal Brain Breast Colon Heart Kidney Liver Lung LymphNode Ovary Prostate SkeletalMuscle Testes Thyroid WhiteBloodCells EdomStromalCell

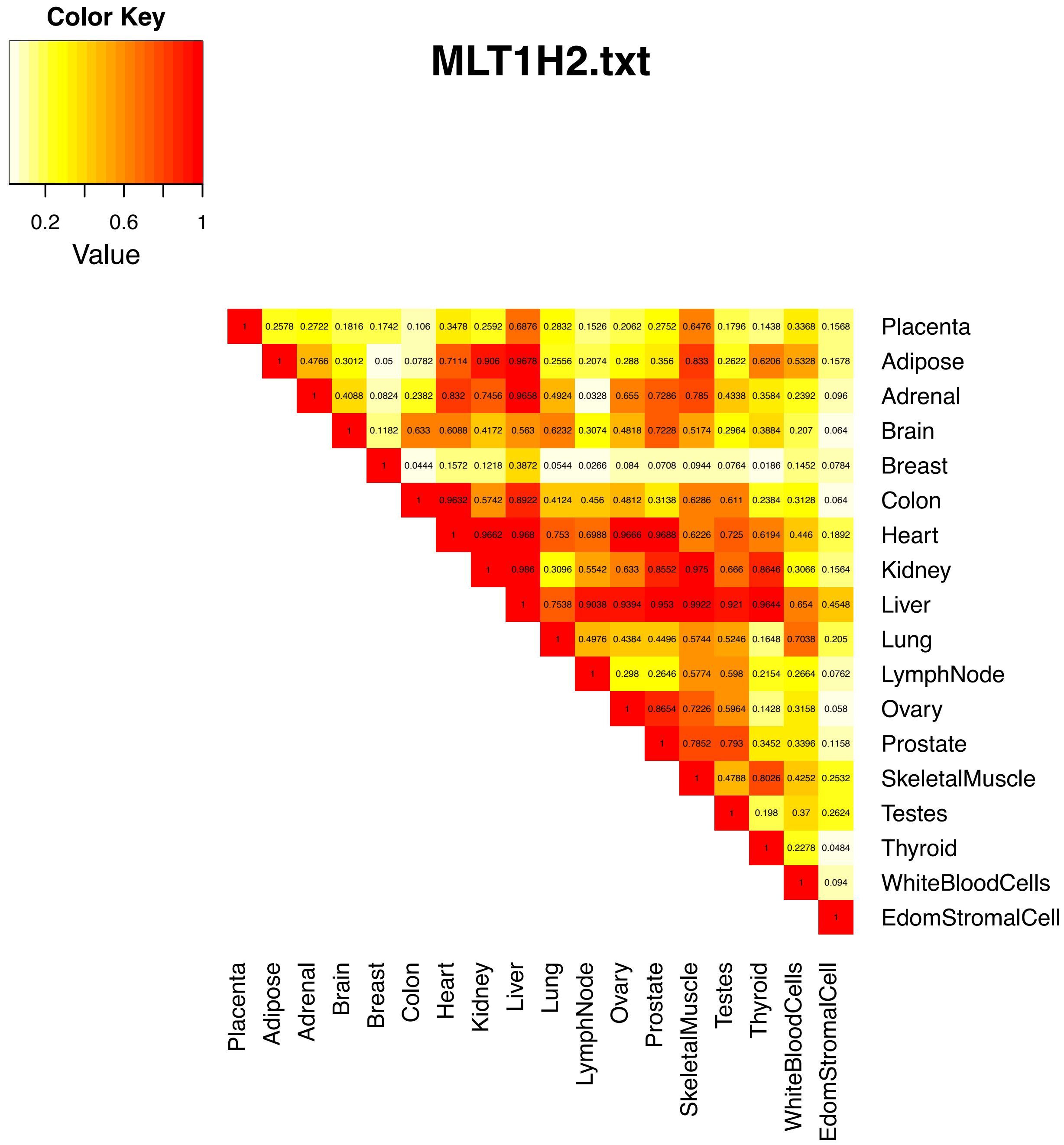
Color Key
0.2 0.6 1
Value
MLT1H2.txt
1 0.2578 0.2722 0.1816 0.1742 0.106 0.3478 0.2592 0.6876 0.2832 0.1526 0.2062 0.2752 0.6476 0.1796 0.1438 0.3368 0.1568 Placenta
1 0.4766 0.3012 0.05 0.0782 0.7114 0.906 0.9678 0.2556 0.2074 0.288 0.356 0.833 0.2622 0.6206 0.5328 0.1578 Adipose
1 0.4088 0.0824 0.2382 0.832 0.7456 0.9658 0.4924 0.0328 0.655 0.7286 0.785 0.4338 0.3584 0.2392 0.096 Adrenal
1 0.1182 0.633 0.6088 0.4172 0.563 0.6232 0.3074 0.4818 0.7228 0.5174 0.2964 0.3884 0.207 0.064 Brain
1 0.0444 0.1572 0.1218 0.3872 0.0544 0.0266 0.084 0.0708 0.0944 0.0764 0.0186 0.1452 0.0784 Breast
1 0.9632 0.5742 0.8922 0.4124 0.456 0.4812 0.3138 0.6286 0.611 0.2384 0.3128 0.064 Colon
1 0.9662 0.968 0.753 0.6988 0.9666 0.9688 0.6226 0.725 0.6194 0.446 0.1892 Heart
1 0.986 0.3096 0.5542 0.633 0.8552 0.975 0.666 0.8646 0.3066 0.1564 Kidney
1 0.7538 0.9038 0.9394 0.953 0.9922 0.921 0.9644 0.654 0.4548 Liver
1 0.4976 0.4384 0.4496 0.5744 0.5246 0.1648 0.7038 0.205 Lung
1 0.298 0.2646 0.5774 0.598 0.2154 0.2664 0.0762 LymphNode
1 0.8654 0.7226 0.5964 0.1428 0.3158 0.058 Ovary
1 0.7852 0.793 0.3452 0.3396 0.1158 Prostate
1 0.4788 0.8026 0.4252 0.2532 SkeletalMuscle
1 0.198 0.37 0.2624 Testes
1 0.2278 0.0484 Thyroid
1 0.094 WhiteBloodCells
1 EdomStromalCell
Placenta
Adipose
Adrenal
Brain
Breast
Colon
Heart
Kidney
Liver
Lung
LymphNode
Ovary
Prostate
SkeletalMuscle
Testes
Thyroid
WhiteBloodCells
EdomStromalCell

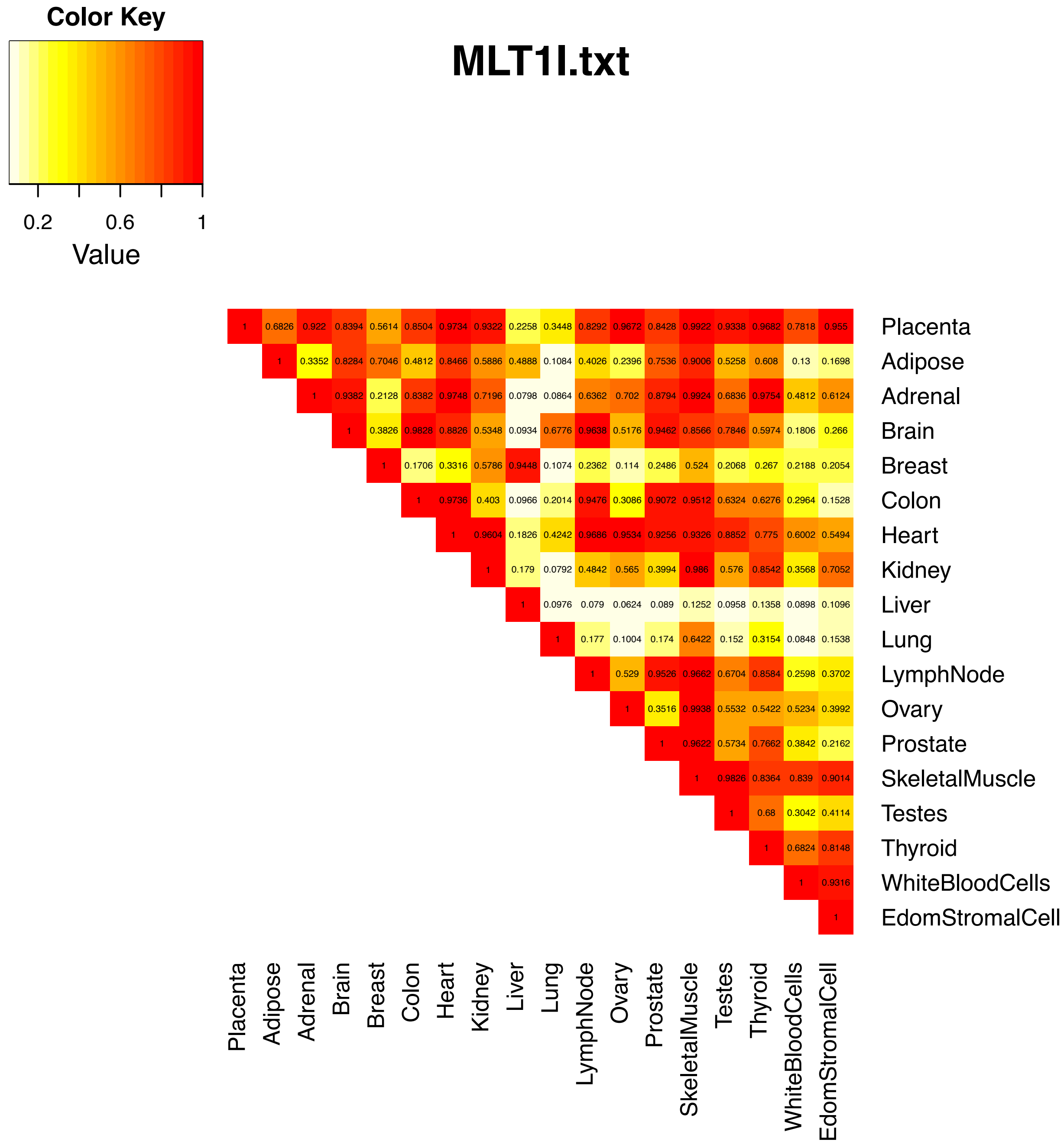
Color Key
MLT1I.txt
0.2
0.6
1
Value
Placenta
Adipose
Adrenal
Brain
Breast
Colon
Heart
Kidney
Liver
Lung
LymphNode
Ovary
Prostate
SkeletalMuscle
Testes
Thyroid
WhiteBloodCells
EdomStromalCell
1 0.6826 0.922 0.8394 0.5614 0.8504 0.9734 0.9322 0.2258 0.3448 0.8292 0.9672 0.8428 0.9922 0.9338 0.9682 0.7818 0.955
1 0.3352 0.8284 0.7046 0.4812 0.8466 0.5886 0.4888 0.1084 0.4026 0.2396 0.7536 0.9006 0.5258 0.608 0.13 0.1698
1 0.9382 0.2128 0.8382 0.9748 0.7196 0.0798 0.0864 0.6362 0.702 0.8794 0.9924 0.6836 0.9754 0.4812 0.6124
1 0.3826 0.9828 0.8826 0.5348 0.0934 0.6776 0.9638 0.5176 0.9462 0.8566 0.7846 0.5974 0.1806 0.266
1 0.1706 0.3316 0.5786 0.9448 0.1074 0.2362 0.114 0.2486 0.524 0.2068 0.267 0.2188 0.2054
1 0.9736 0.403 0.0966 0.2014 0.9476 0.3086 0.9072 0.9512 0.6324 0.6276 0.2964 0.1528
1 0.9604 0.1826 0.4242 0.9686 0.9534 0.9256 0.9326 0.8852 0.775 0.6002 0.5494
1 0.179 0.0792 0.4842 0.565 0.3994 0.986 0.576 0.8542 0.3568 0.7052
1 0.0976 0.079 0.0624 0.089 0.1252 0.0958 0.1358 0.0898 0.1096
1 0.177 0.1004 0.174 0.6422 0.152 0.3154 0.0848 0.1538
1 0.529 0.9526 0.9662 0.6704 0.8584 0.2598 0.3702
1 0.3516 0.9938 0.5532 0.5422 0.5234 0.3992
1 0.9622 0.5734 0.7662 0.3842 0.2162
1 0.9826 0.8364 0.839 0.9014
1 0.68 0.3042 0.4114
1 0.6824 0.8148
1 0.9316
1

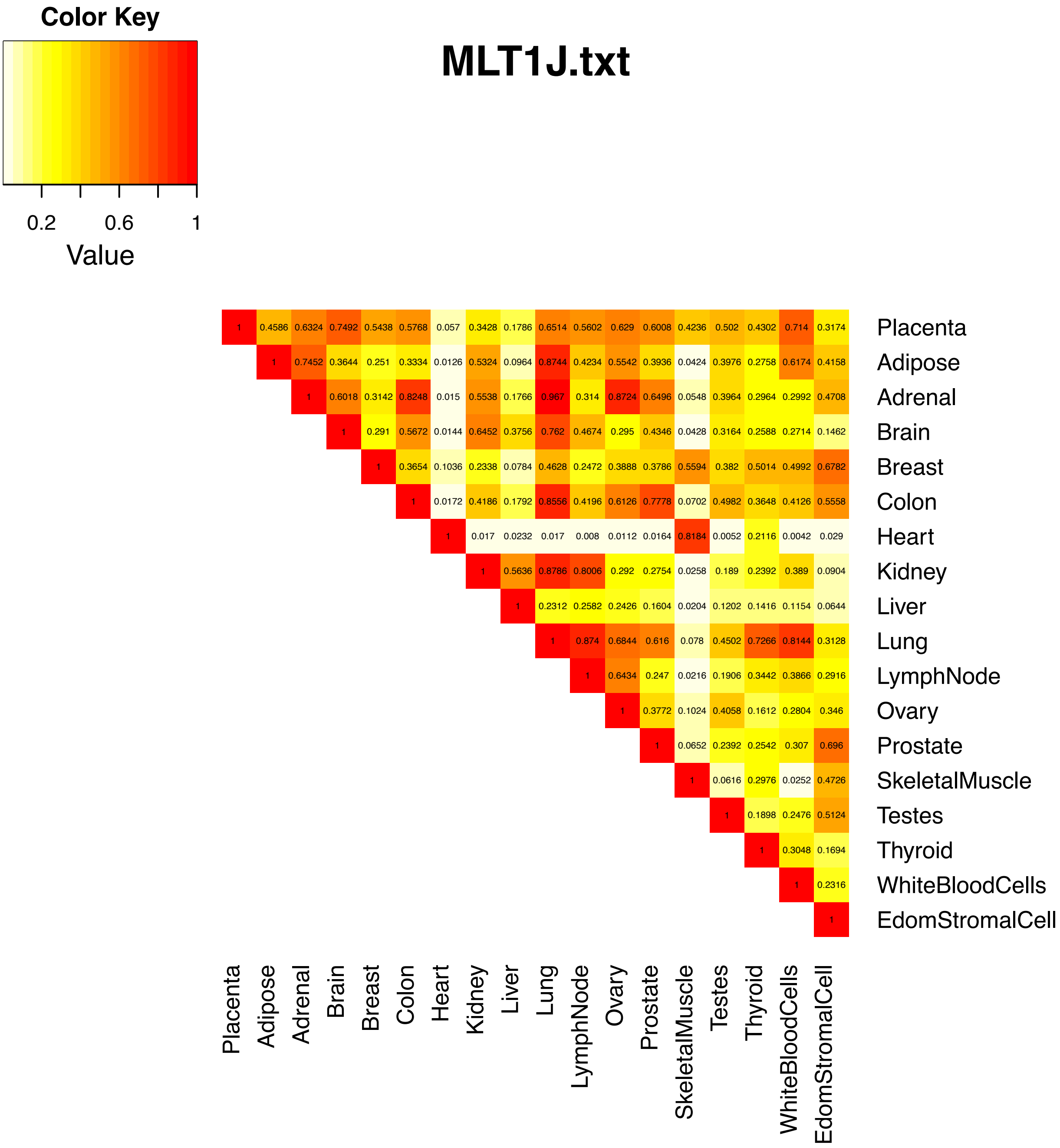
Color Key
0.2
0.6
1
Value
MLT1J.txt
Placenta: 1 0.4586 0.6324 0.7492 0.5438 0.5768 0.057 0.3428 0.1786 0.6514 0.5602 0.629 0.6008 0.4236 0.502 0.4302 0.714 0.3174
Adipose: 1 0.7452 0.3644 0.251 0.3334 0.0126 0.5324 0.0964 0.8744 0.4234 0.5542 0.3936 0.0424 0.3976 0.2758 0.6174 0.4158
Adrenal: 1 0.6018 0.3142 0.8248 0.015 0.5538 0.1766 0.967 0.314 0.8724 0.6496 0.0548 0.3964 0.2964 0.2992 0.4708
Brain: 1 0.291 0.5672 0.0144 0.6452 0.3756 0.762 0.4674 0.295 0.4346 0.0428 0.3164 0.2588 0.2714 0.1462
Breast: 1 0.3654 0.1036 0.2338 0.0784 0.4628 0.2472 0.3888 0.3786 0.5594 0.382 0.5014 0.4992 0.6782
Colon: 1 0.0172 0.4186 0.1792 0.8556 0.4196 0.6126 0.7778 0.0702 0.4982 0.3648 0.4126 0.5558
Heart: 1 0.017 0.0232 0.017 0.008 0.0112 0.0164 0.8184 0.0052 0.2116 0.0042 0.029
Kidney: 1 0.5636 0.8786 0.8006 0.292 0.2754 0.0258 0.189 0.2392 0.389 0.0904
Liver: 1 0.2312 0.2582 0.2426 0.1604 0.0204 0.1202 0.1416 0.1154 0.0644
Lung: 1 0.874 0.6844 0.616 0.078 0.4502 0.7266 0.8144 0.3128
LymphNode: 1 0.6434 0.247 0.0216 0.1906 0.3442 0.3866 0.2916
Ovary: 1 0.3772 0.1024 0.4058 0.1612 0.2804 0.346
Prostate: 1 0.0652 0.2392 0.2542 0.307 0.696
SkeletalMuscle: 1 0.0616 0.2976 0.0252 0.4726
Testes: 1 0.1898 0.2476 0.5124
Thyroid: 1 0.3048 0.1694
WhiteBloodCells: 1 0.2316
EdomStromalCell: 1
Placenta Adipose Adrenal Brain Breast Colon Heart Kidney Liver Lung LymphNode Ovary Prostate SkeletalMuscle Testes Thyroid WhiteBloodCells EdomStromalCell

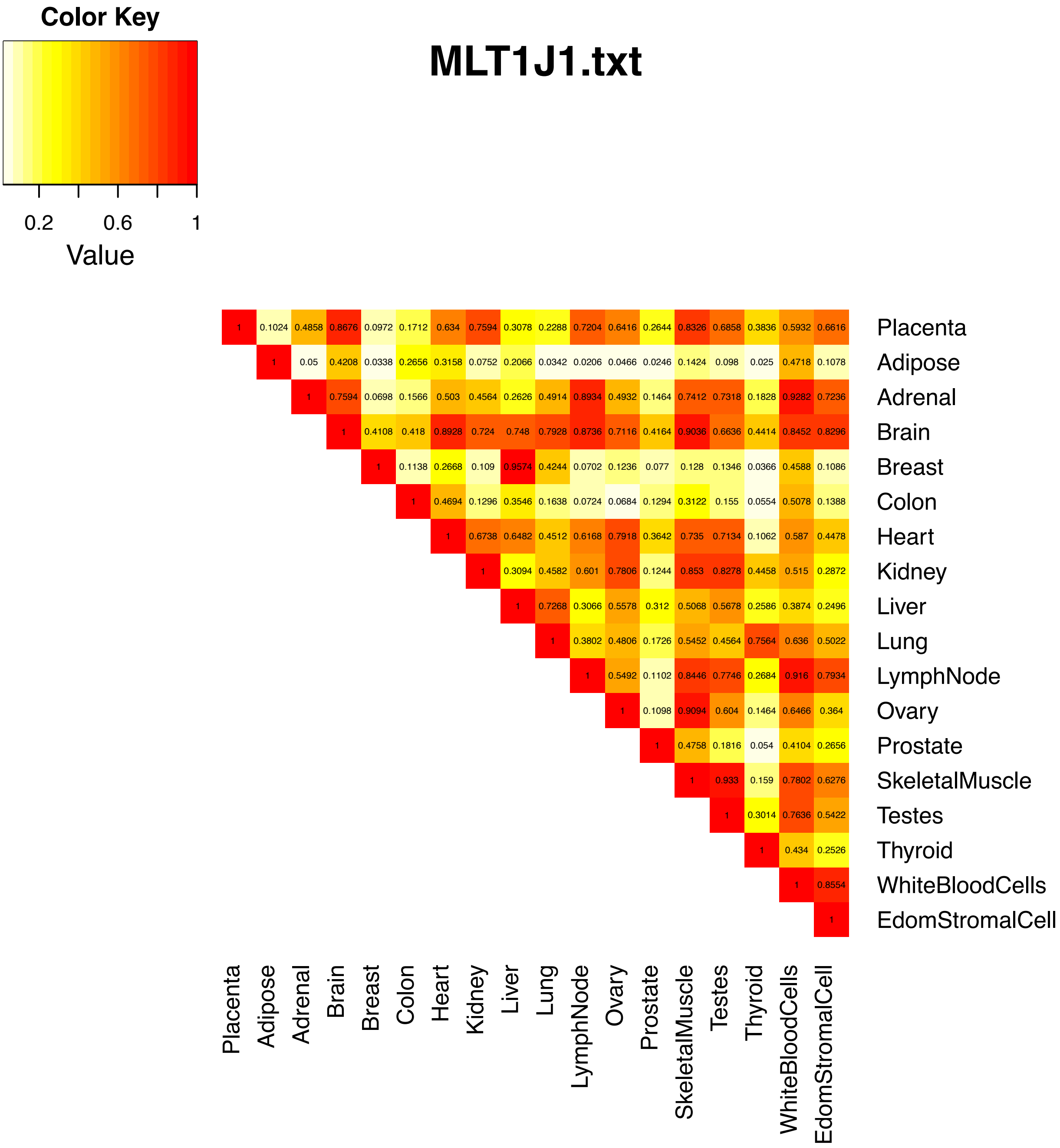
Color Key
0.2 0.6 1
Value
MLT1J1.txt
Placenta: 1 0.1024 0.4858 0.8676 0.0972 0.1712 0.634 0.7594 0.3078 0.2288 0.7204 0.6416 0.2644 0.8326 0.6858 0.3836 0.5932 0.6616
Adipose: 1 0.05 0.4208 0.0338 0.2656 0.3158 0.0752 0.2066 0.0342 0.0206 0.0466 0.0246 0.1424 0.098 0.025 0.4718 0.1078
Adrenal: 1 0.7594 0.0698 0.1566 0.503 0.4564 0.2626 0.4914 0.8934 0.4932 0.1464 0.7412 0.7318 0.1828 0.9282 0.7236
Brain: 1 0.4108 0.418 0.8928 0.724 0.748 0.7928 0.8736 0.7116 0.4164 0.9036 0.6636 0.4414 0.8452 0.8296
Breast: 1 0.1138 0.2668 0.109 0.9574 0.4244 0.0702 0.1236 0.077 0.128 0.1346 0.0366 0.4588 0.1086
Colon: 1 0.4694 0.1296 0.3546 0.1638 0.0724 0.0684 0.1294 0.3122 0.155 0.0554 0.5078 0.1388
Heart: 1 0.6738 0.6482 0.4512 0.6168 0.7918 0.3642 0.735 0.7134 0.1062 0.587 0.4478
Kidney: 1 0.3094 0.4582 0.601 0.7806 0.1244 0.853 0.8278 0.4458 0.515 0.2872
Liver: 1 0.7268 0.3066 0.5578 0.312 0.5068 0.5678 0.2586 0.3874 0.2496
Lung: 1 0.3802 0.4806 0.1726 0.5452 0.4564 0.7564 0.636 0.5022
LymphNode: 1 0.5492 0.1102 0.8446 0.7746 0.2684 0.916 0.7934
Ovary: 1 0.1098 0.9094 0.604 0.1464 0.6466 0.364
Prostate: 1 0.4758 0.1816 0.054 0.4104 0.2656
SkeletalMuscle: 1 0.933 0.159 0.7802 0.6276
Testes: 1 0.3014 0.7636 0.5422
Thyroid: 1 0.434 0.2526
WhiteBloodCells: 1 0.8554
EdomStromalCell: 1
Placenta Adipose Adrenal Brain Breast Colon Heart Kidney Liver Lung LymphNode Ovary Prostate SkeletalMuscle Testes Thyroid WhiteBloodCells EdomStromalCell

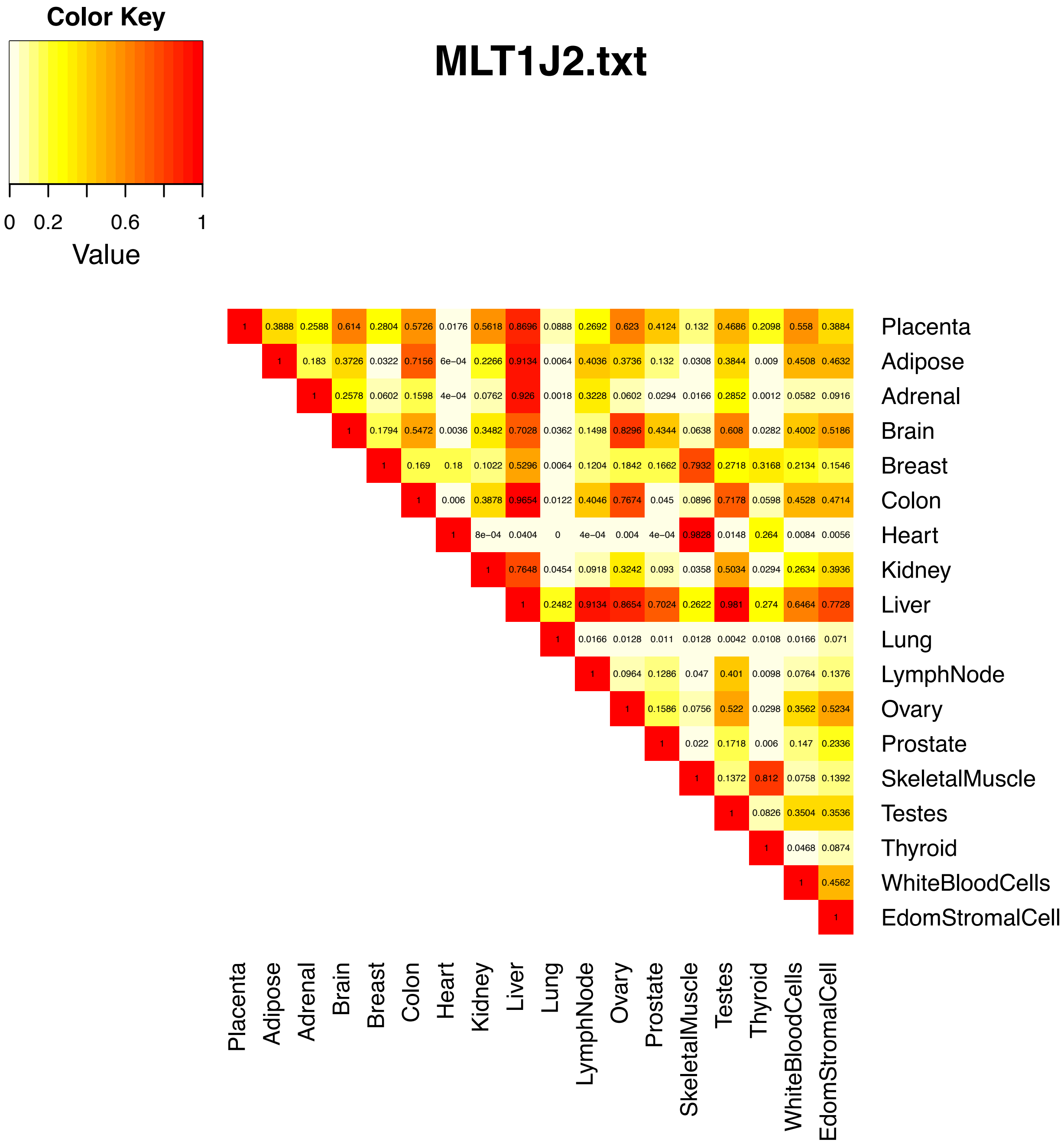
Color Key
0 0.2 0.6 1
Value
MLT1J2.txt
Placenta 1 0.3888 0.2588 0.614 0.2804 0.5726 0.0176 0.5618 0.8696 0.0888 0.2692 0.623 0.4124 0.132 0.4686 0.2098 0.558 0.3884
Adipose 1 0.183 0.3726 0.0322 0.7156 6e−04 0.2266 0.9134 0.0064 0.4036 0.3736 0.132 0.0308 0.3844 0.009 0.4508 0.4632
Adrenal 1 0.2578 0.0602 0.1598 4e−04 0.0762 0.926 0.0018 0.3228 0.0602 0.0294 0.0166 0.2852 0.0012 0.0582 0.0916
Brain 1 0.1794 0.5472 0.0036 0.3482 0.7028 0.0362 0.1498 0.8296 0.4344 0.0638 0.608 0.0282 0.4002 0.5186
Breast 1 0.169 0.18 0.1022 0.5296 0.0064 0.1204 0.1842 0.1662 0.7932 0.2718 0.3168 0.2134 0.1546
Colon 1 0.006 0.3878 0.9654 0.0122 0.4046 0.7674 0.045 0.0896 0.7178 0.0598 0.4528 0.4714
Heart 1 8e−04 0.0404 0 4e−04 0.004 4e−04 0.9828 0.0148 0.264 0.0084 0.0056
Kidney 1 0.7648 0.0454 0.0918 0.3242 0.093 0.0358 0.5034 0.0294 0.2634 0.3936
Liver 1 0.2482 0.9134 0.8654 0.7024 0.2622 0.981 0.274 0.6464 0.7728
Lung 1 0.0166 0.0128 0.011 0.0128 0.0042 0.0108 0.0166 0.071
LymphNode 1 0.0964 0.1286 0.047 0.401 0.0098 0.0764 0.1376
Ovary 1 0.1586 0.0756 0.522 0.0298 0.3562 0.5234
Prostate 1 0.022 0.1718 0.006 0.147 0.2336
SkeletalMuscle 1 0.1372 0.812 0.0758 0.1392
Testes 1 0.0826 0.3504 0.3536
Thyroid 1 0.0468 0.0874
WhiteBloodCells 1 0.4562
EdomStromalCell 1
Placenta Adipose Adrenal Brain Breast Colon Heart Kidney Liver Lung LymphNode Ovary Prostate SkeletalMuscle Testes Thyroid WhiteBloodCells EdomStromalCell

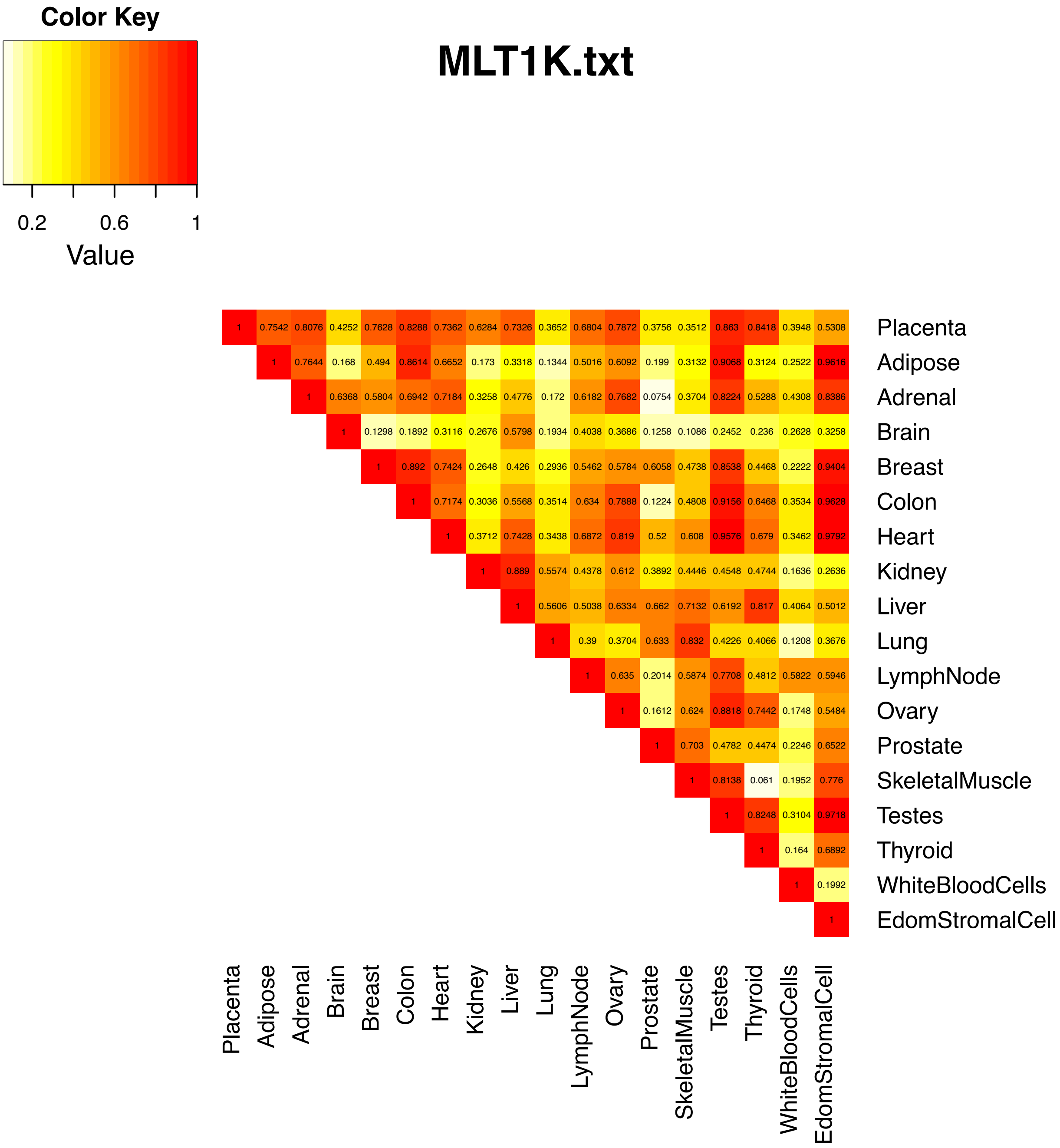
Color Key
0.2
0.6
1
Value
MLT1K.txt
Placenta 1 0.7542 0.8076 0.4252 0.7628 0.8288 0.7362 0.6284 0.7326 0.3652 0.6804 0.7872 0.3756 0.3512 0.863 0.8418 0.3948 0.5308
Adipose 1 0.7644 0.168 0.494 0.8614 0.6652 0.173 0.3318 0.1344 0.5016 0.6092 0.199 0.3132 0.9068 0.3124 0.2522 0.9616
Adrenal 1 0.6368 0.5804 0.6942 0.7184 0.3258 0.4776 0.172 0.6182 0.7682 0.0754 0.3704 0.8224 0.5288 0.4308 0.8386
Brain 1 0.1298 0.1892 0.3116 0.2676 0.5798 0.1934 0.4038 0.3686 0.1258 0.1086 0.2452 0.236 0.2628 0.3258
Breast 1 0.892 0.7424 0.2648 0.426 0.2936 0.5462 0.5784 0.6058 0.4738 0.8538 0.4468 0.2222 0.9404
Colon 1 0.7174 0.3036 0.5568 0.3514 0.634 0.7888 0.1224 0.4808 0.9156 0.6468 0.3534 0.9628
Heart 1 0.3712 0.7428 0.3438 0.6872 0.819 0.52 0.608 0.9576 0.679 0.3462 0.9792
Kidney 1 0.889 0.5574 0.4378 0.612 0.3892 0.4446 0.4548 0.4744 0.1636 0.2636
Liver 1 0.5606 0.5038 0.6334 0.662 0.7132 0.6192 0.817 0.4064 0.5012
Lung 1 0.39 0.3704 0.633 0.832 0.4226 0.4066 0.1208 0.3676
LymphNode 1 0.635 0.2014 0.5874 0.7708 0.4812 0.5822 0.5946
Ovary 1 0.1612 0.624 0.8818 0.7442 0.1748 0.5484
Prostate 1 0.703 0.4782 0.4474 0.2246 0.6522
SkeletalMuscle 1 0.8138 0.061 0.1952 0.776
Testes 1 0.8248 0.3104 0.9718
Thyroid 1 0.164 0.6892
WhiteBloodCells 1 0.1992
EdomStromalCell 1
Placenta
Adipose
Adrenal
Brain
Breast
Colon
Heart
Kidney
Liver
Lung
LymphNode
Ovary
Prostate
SkeletalMuscle
Testes
Thyroid
WhiteBloodCells
EdomStromalCell

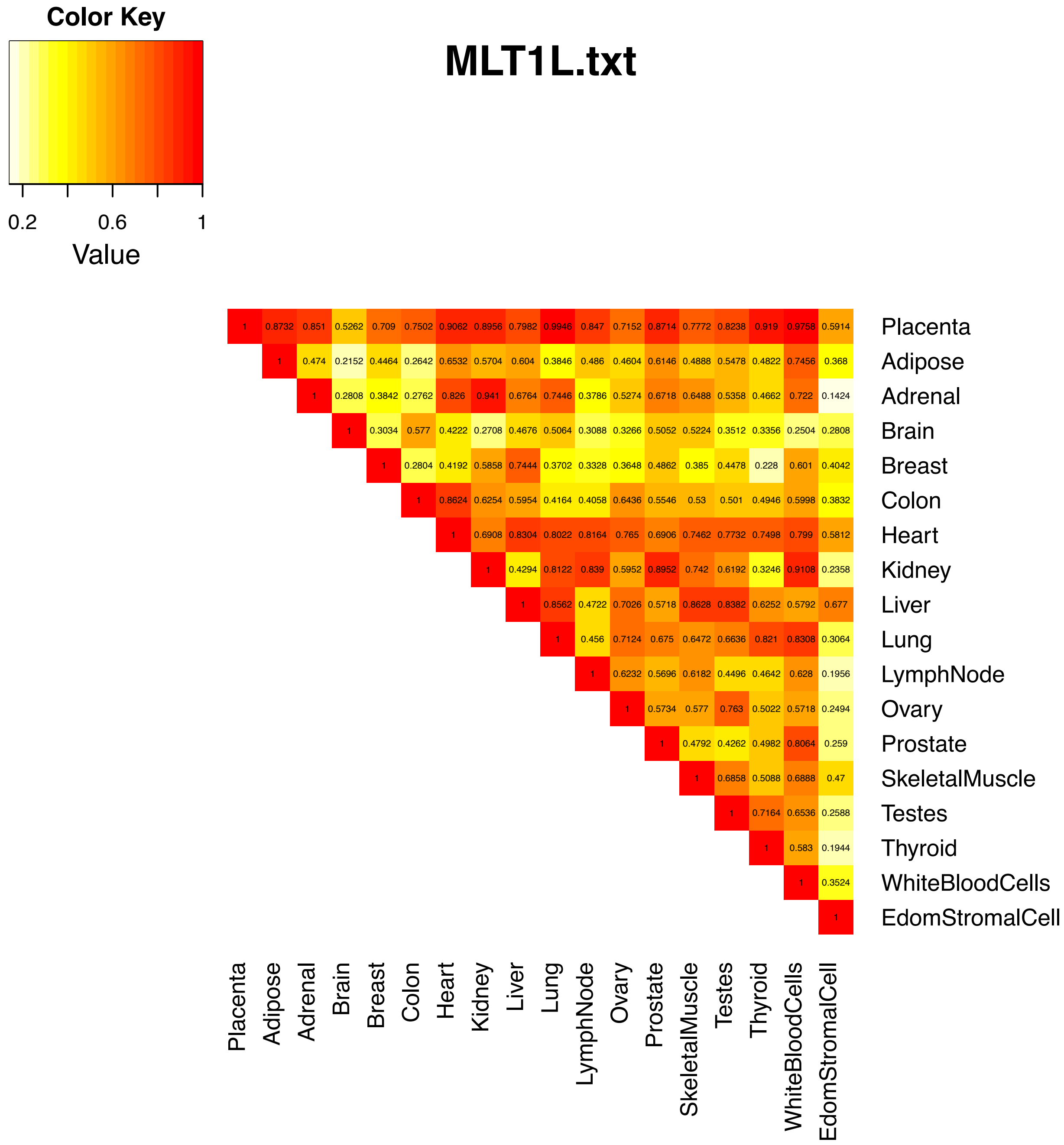
Color Key
0.2
0.6
1
Value
MLT1L.txt
Placenta: 1 0.8732 0.851 0.5262 0.709 0.7502 0.9062 0.8956 0.7982 0.9946 0.847 0.7152 0.8714 0.7772 0.8238 0.919 0.9758 0.5914
Adipose: 1 0.474 0.2152 0.4464 0.2642 0.6532 0.5704 0.604 0.3846 0.486 0.4604 0.6146 0.4888 0.5478 0.4822 0.7456 0.368
Adrenal: 1 0.2808 0.3842 0.2762 0.826 0.941 0.6764 0.7446 0.3786 0.5274 0.6718 0.6488 0.5358 0.4662 0.722 0.1424
Brain: 1 0.3034 0.577 0.4222 0.2708 0.4676 0.5064 0.3088 0.3266 0.5052 0.5224 0.3512 0.3356 0.2504 0.2808
Breast: 1 0.2804 0.4192 0.5858 0.7444 0.3702 0.3328 0.3648 0.4862 0.385 0.4478 0.228 0.601 0.4042
Colon: 1 0.8624 0.6254 0.5954 0.4164 0.4058 0.6436 0.5546 0.53 0.501 0.4946 0.5998 0.3832
Heart: 1 0.6908 0.8304 0.8022 0.8164 0.765 0.6906 0.7462 0.7732 0.7498 0.799 0.5812
Kidney: 1 0.4294 0.8122 0.839 0.5952 0.8952 0.742 0.6192 0.3246 0.9108 0.2358
Liver: 1 0.8562 0.4722 0.7026 0.5718 0.8628 0.8382 0.6252 0.5792 0.677
Lung: 1 0.456 0.7124 0.675 0.6472 0.6636 0.821 0.8308 0.3064
LymphNode: 1 0.6232 0.5696 0.6182 0.4496 0.4642 0.628 0.1956
Ovary: 1 0.5734 0.577 0.763 0.5022 0.5718 0.2494
Prostate: 1 0.4792 0.4262 0.4982 0.8064 0.259
SkeletalMuscle: 1 0.6858 0.5088 0.6888 0.47
Testes: 1 0.7164 0.6536 0.2588
Thyroid: 1 0.583 0.1944
WhiteBloodCells: 1 0.3524
EdomStromalCell: 1
Placenta
Adipose
Adrenal
Brain
Breast
Colon
Heart
Kidney
Liver
Lung
LymphNode
Ovary
Prostate
SkeletalMuscle
Testes
Thyroid
WhiteBloodCells
EdomStromalCell

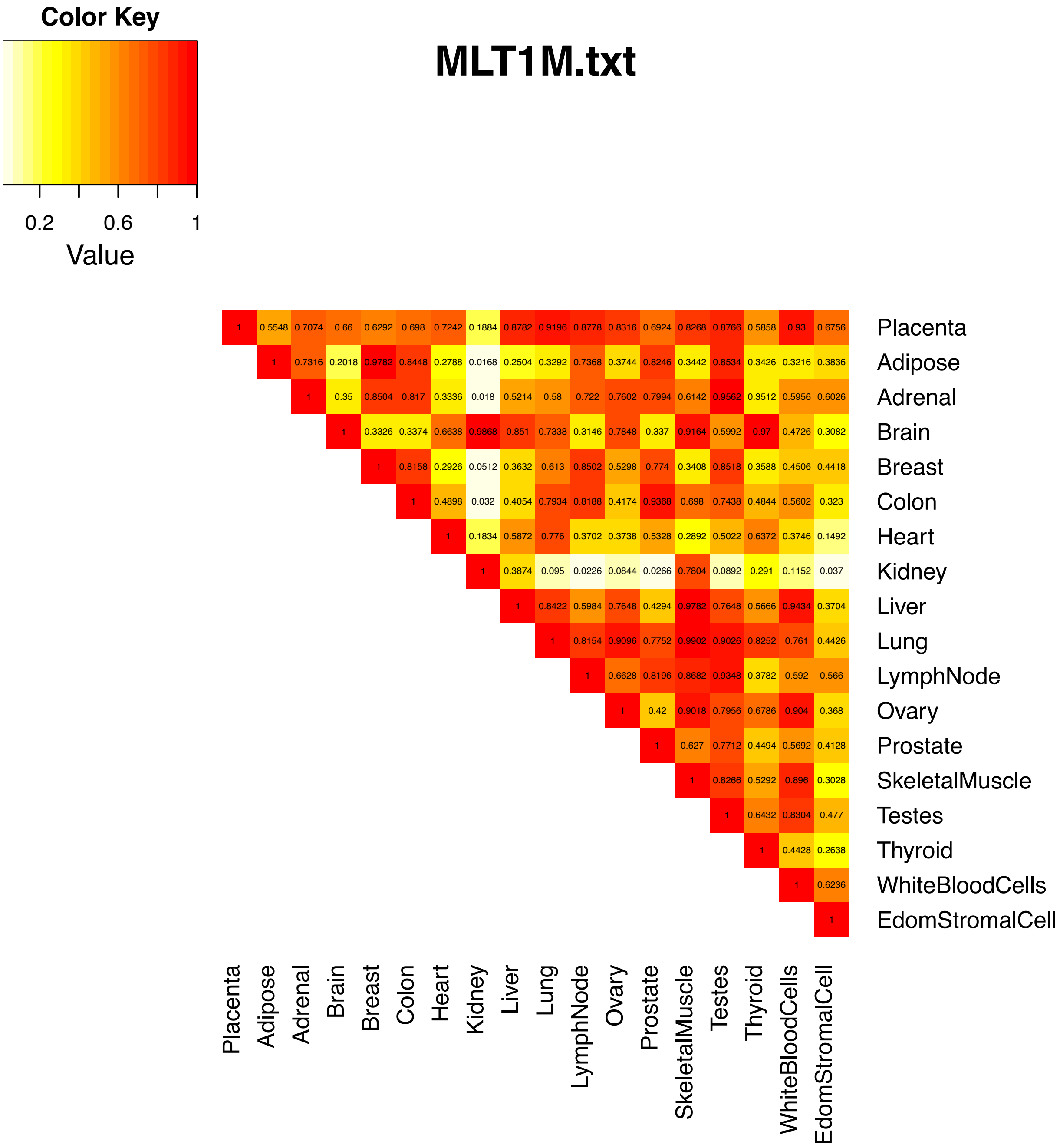
Color Key
0.2 0.6 1
Value
MLT1M.txt
Placenta: 1 0.5548 0.7074 0.66 0.6292 0.698 0.7242 0.1884 0.8782 0.9196 0.8778 0.8316 0.6924 0.8268 0.8766 0.5858 0.93 0.6756
Adipose: 1 0.7316 0.2018 0.9782 0.8448 0.2788 0.0168 0.2504 0.3292 0.7368 0.3744 0.8246 0.3442 0.8534 0.3426 0.3216 0.3836
Adrenal: 1 0.35 0.8504 0.817 0.3336 0.018 0.5214 0.58 0.722 0.7602 0.7994 0.6142 0.9562 0.3512 0.5956 0.6026
Brain: 1 0.3326 0.3374 0.6638 0.9868 0.851 0.7338 0.3146 0.7848 0.337 0.9164 0.5992 0.97 0.4726 0.3082
Breast: 1 0.8158 0.2926 0.0512 0.3632 0.613 0.8502 0.5298 0.774 0.3408 0.8518 0.3588 0.4506 0.4418
Colon: 1 0.4898 0.032 0.4054 0.7934 0.8188 0.4174 0.9368 0.698 0.7438 0.4844 0.5602 0.323
Heart: 1 0.1834 0.5872 0.776 0.3702 0.3738 0.5328 0.2892 0.5022 0.6372 0.3746 0.1492
Kidney: 1 0.3874 0.095 0.0226 0.0844 0.0266 0.7804 0.0892 0.291 0.1152 0.037
Liver: 1 0.8422 0.5984 0.7648 0.4294 0.9782 0.7648 0.5666 0.9434 0.3704
Lung: 1 0.8154 0.9096 0.7752 0.9902 0.9026 0.8252 0.761 0.4426
LymphNode: 1 0.6628 0.8196 0.8682 0.9348 0.3782 0.592 0.566
Ovary: 1 0.42 0.9018 0.7956 0.6786 0.904 0.368
Prostate: 1 0.627 0.7712 0.4494 0.5692 0.4128
SkeletalMuscle: 1 0.8266 0.5292 0.896 0.3028
Testes: 1 0.6432 0.8304 0.477
Thyroid: 1 0.4428 0.2638
WhiteBloodCells: 1 0.6236
EdomStromalCell: 1
Placenta Adipose Adrenal Brain Breast Colon Heart Kidney Liver Lung LymphNode Ovary Prostate SkeletalMuscle Testes Thyroid WhiteBloodCells EdomStromalCell

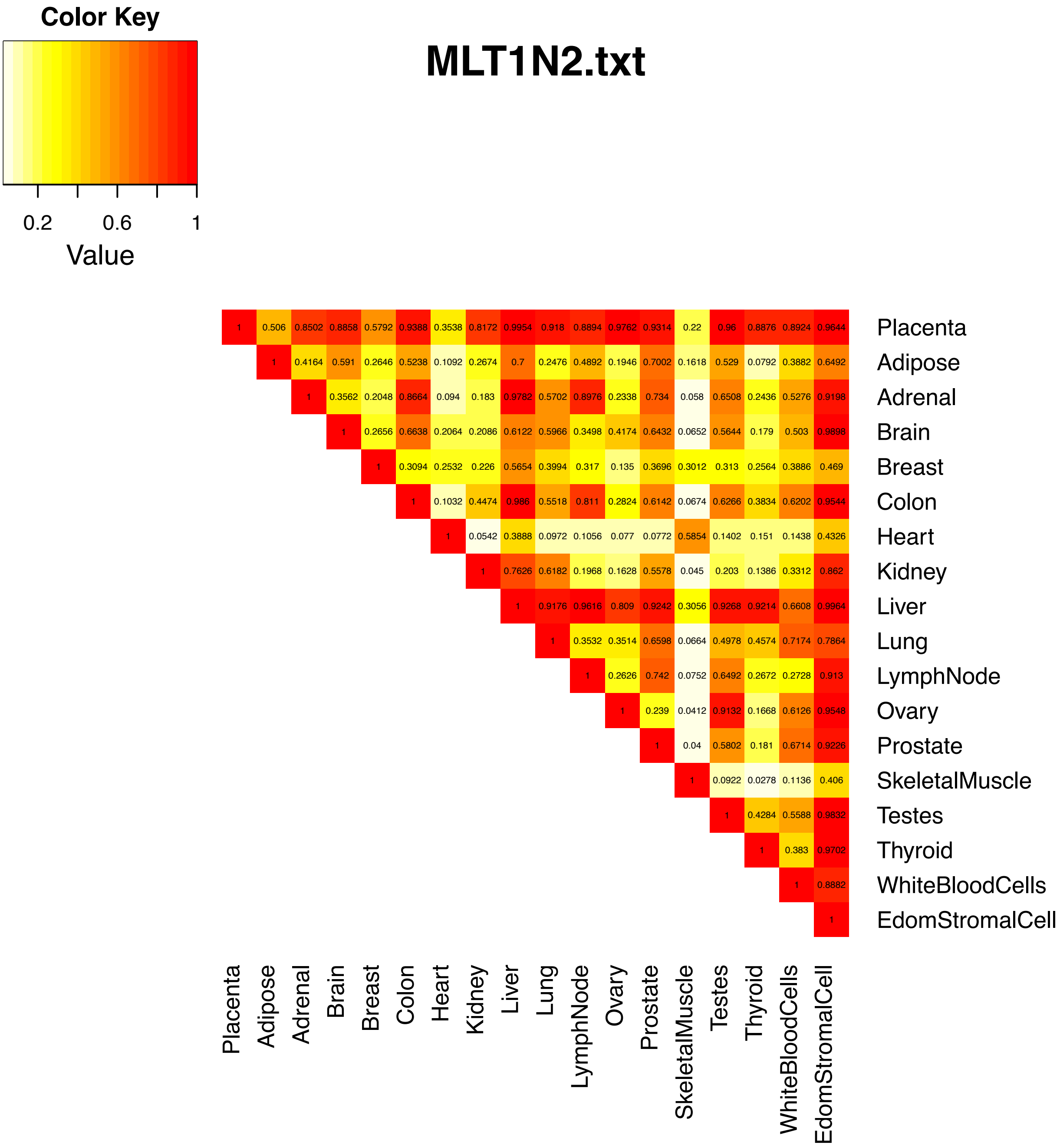
Color Key
0.2 0.6 1
Value
MLT1N2.txt
Placenta: 1 0.506 0.8502 0.8858 0.5792 0.9388 0.3538 0.8172 0.9954 0.918 0.8894 0.9762 0.9314 0.22 0.96 0.8876 0.8924 0.9644
Adipose: 1 0.4164 0.591 0.2646 0.5238 0.1092 0.2674 0.7 0.2476 0.4892 0.1946 0.7002 0.1618 0.529 0.0792 0.3882 0.6492
Adrenal: 1 0.3562 0.2048 0.8664 0.094 0.183 0.9782 0.5702 0.8976 0.2338 0.734 0.058 0.6508 0.2436 0.5276 0.9198
Brain: 1 0.2656 0.6638 0.2064 0.2086 0.6122 0.5966 0.3498 0.4174 0.6432 0.0652 0.5644 0.179 0.503 0.9898
Breast: 1 0.3094 0.2532 0.226 0.5654 0.3994 0.317 0.135 0.3696 0.3012 0.313 0.2564 0.3886 0.469
Colon: 1 0.1032 0.4474 0.986 0.5518 0.811 0.2824 0.6142 0.0674 0.6266 0.3834 0.6202 0.9544
Heart: 1 0.0542 0.3888 0.0972 0.1056 0.077 0.0772 0.5854 0.1402 0.151 0.1438 0.4326
Kidney: 1 0.7626 0.6182 0.1968 0.1628 0.5578 0.045 0.203 0.1386 0.3312 0.862
Liver: 1 0.9176 0.9616 0.809 0.9242 0.3056 0.9268 0.9214 0.6608 0.9964
Lung: 1 0.3532 0.3514 0.6598 0.0664 0.4978 0.4574 0.7174 0.7864
LymphNode: 1 0.2626 0.742 0.0752 0.6492 0.2672 0.2728 0.913
Ovary: 1 0.239 0.0412 0.9132 0.1668 0.6126 0.9548
Prostate: 1 0.04 0.5802 0.181 0.6714 0.9226
SkeletalMuscle: 1 0.0922 0.0278 0.1136 0.406
Testes: 1 0.4284 0.5588 0.9832
Thyroid: 1 0.383 0.9702
WhiteBloodCells: 1 0.8882
EdomStromalCell: 1
Placenta Adipose Adrenal Brain Breast Colon Heart Kidney Liver Lung LymphNode Ovary Prostate SkeletalMuscle Testes Thyroid WhiteBloodCells EdomStromalCell

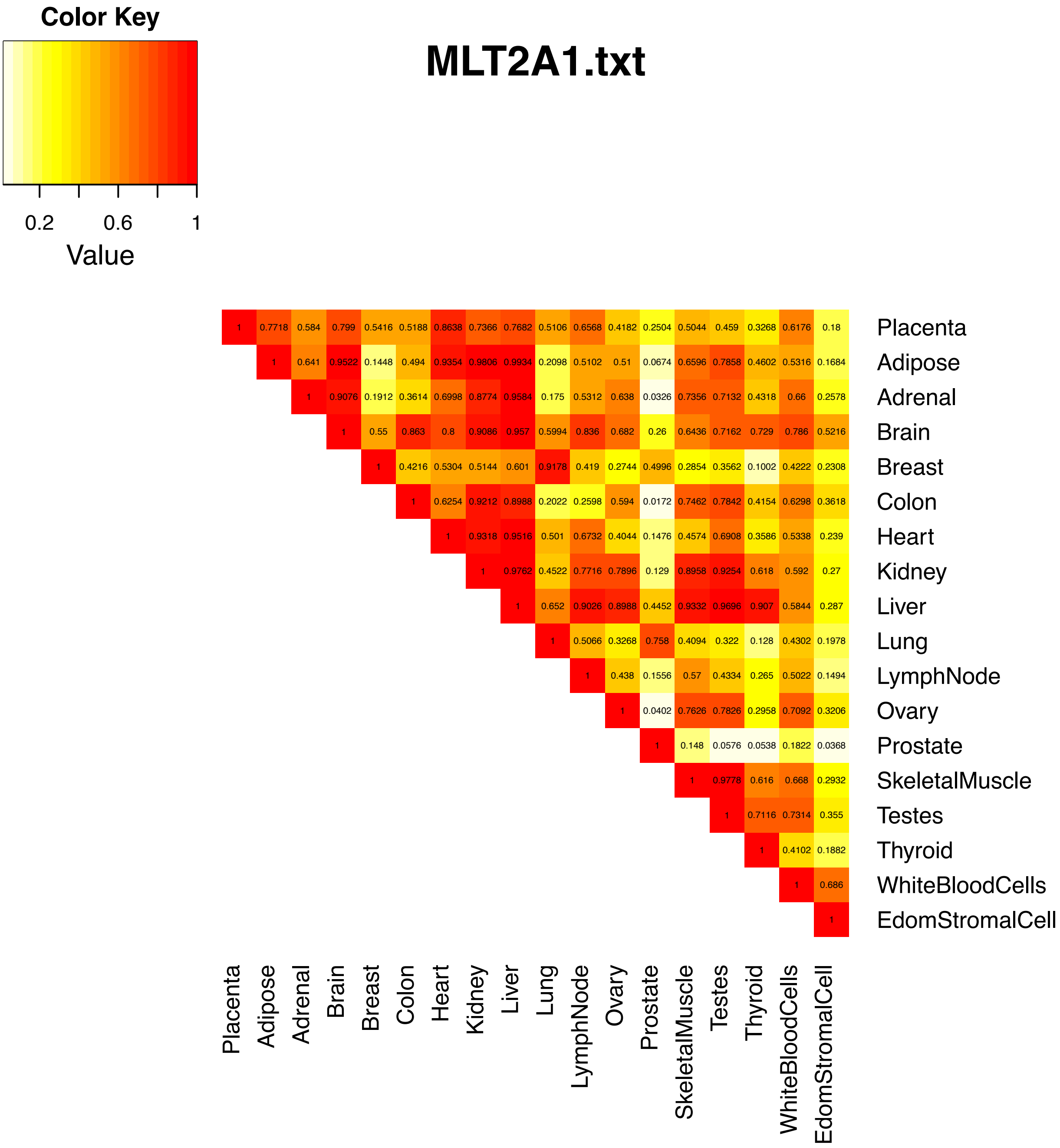
Color Key
0.2
0.6
1
Value
MLT2A1.txt
Placenta 1 0.7718 0.584 0.799 0.5416 0.5188 0.8638 0.7366 0.7682 0.5106 0.6568 0.4182 0.2504 0.5044 0.459 0.3268 0.6176 0.18
Adipose 1 0.641 0.9522 0.1448 0.494 0.9354 0.9806 0.9934 0.2098 0.5102 0.51 0.0674 0.6596 0.7858 0.4602 0.5316 0.1684
Adrenal 1 0.9076 0.1912 0.3614 0.6998 0.8774 0.9584 0.175 0.5312 0.638 0.0326 0.7356 0.7132 0.4318 0.66 0.2578
Brain 1 0.55 0.863 0.8 0.9086 0.957 0.5994 0.836 0.682 0.26 0.6436 0.7162 0.729 0.786 0.5216
Breast 1 0.4216 0.5304 0.5144 0.601 0.9178 0.419 0.2744 0.4996 0.2854 0.3562 0.1002 0.4222 0.2308
Colon 1 0.6254 0.9212 0.8988 0.2022 0.2598 0.594 0.0172 0.7462 0.7842 0.4154 0.6298 0.3618
Heart 1 0.9318 0.9516 0.501 0.6732 0.4044 0.1476 0.4574 0.6908 0.3586 0.5338 0.239
Kidney 1 0.9762 0.4522 0.7716 0.7896 0.129 0.8958 0.9254 0.618 0.592 0.27
Liver 1 0.652 0.9026 0.8988 0.4452 0.9332 0.9696 0.907 0.5844 0.287
Lung 1 0.5066 0.3268 0.758 0.4094 0.322 0.128 0.4302 0.1978
LymphNode 1 0.438 0.1556 0.57 0.4334 0.265 0.5022 0.1494
Ovary 1 0.0402 0.7626 0.7826 0.2958 0.7092 0.3206
Prostate 1 0.148 0.0576 0.0538 0.1822 0.0368
SkeletalMuscle 1 0.9778 0.616 0.668 0.2932
Testes 1 0.7116 0.7314 0.355
Thyroid 1 0.4102 0.1882
WhiteBloodCells 1 0.686
EdomStromalCell 1
Placenta
Adipose
Adrenal
Brain
Breast
Colon
Heart
Kidney
Liver
Lung
LymphNode
Ovary
Prostate
SkeletalMuscle
Testes
Thyroid
WhiteBloodCells
EdomStromalCell

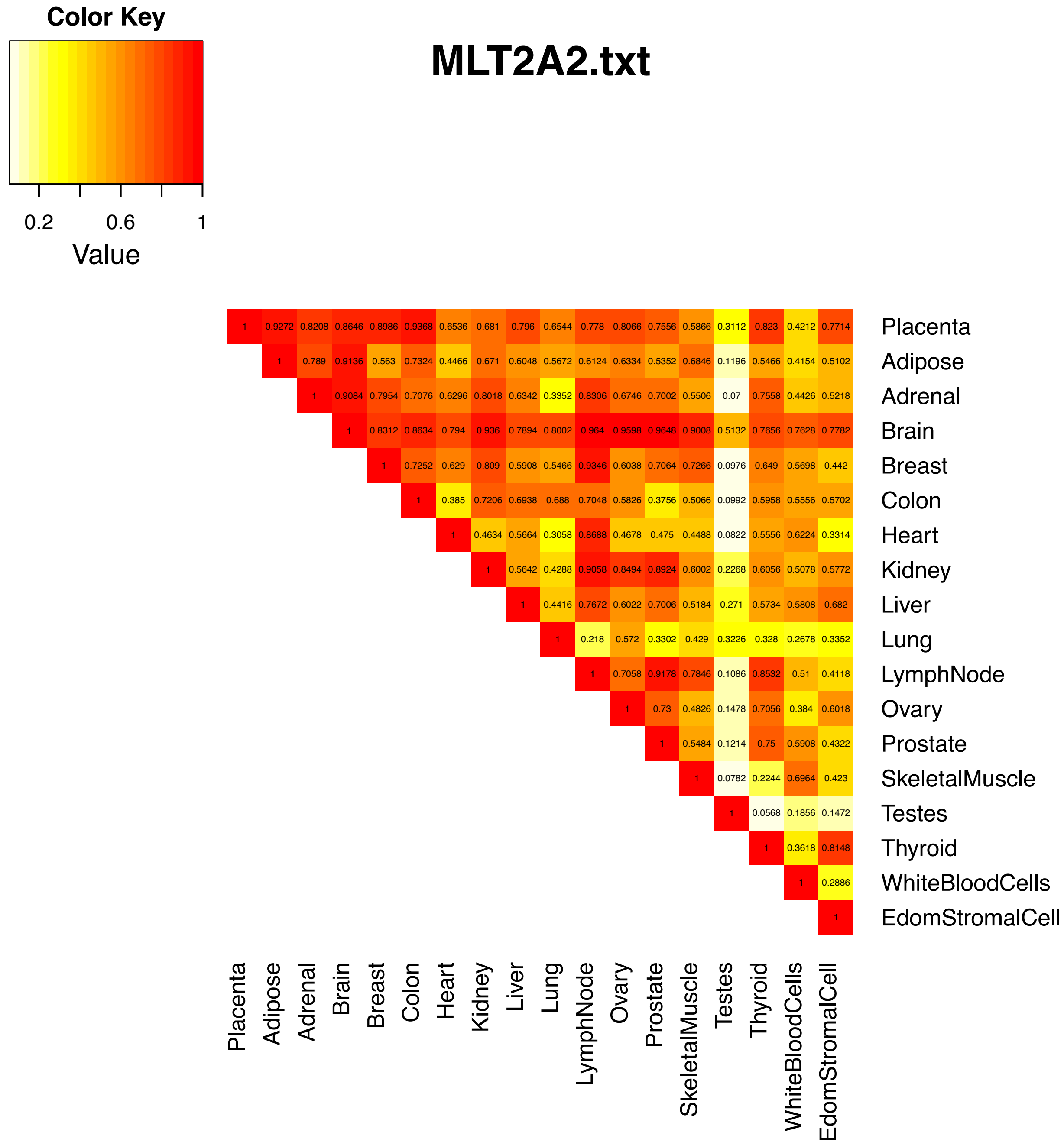
Color Key
0.2
0.6
1
Value
MLT2A2.txt
Placenta: 1 0.9272 0.8208 0.8646 0.8986 0.9368 0.6536 0.681 0.796 0.6544 0.778 0.8066 0.7556 0.5866 0.3112 0.823 0.4212 0.7714
Adipose: 1 0.789 0.9136 0.563 0.7324 0.4466 0.671 0.6048 0.5672 0.6124 0.6334 0.5352 0.6846 0.1196 0.5466 0.4154 0.5102
Adrenal: 1 0.9084 0.7954 0.7076 0.6296 0.8018 0.6342 0.3352 0.8306 0.6746 0.7002 0.5506 0.07 0.7558 0.4426 0.5218
Brain: 1 0.8312 0.8634 0.794 0.936 0.7894 0.8002 0.964 0.9598 0.9648 0.9008 0.5132 0.7656 0.7628 0.7782
Breast: 1 0.7252 0.629 0.809 0.5908 0.5466 0.9346 0.6038 0.7064 0.7266 0.0976 0.649 0.5698 0.442
Colon: 1 0.385 0.7206 0.6938 0.688 0.7048 0.5826 0.3756 0.5066 0.0992 0.5958 0.5556 0.5702
Heart: 1 0.4634 0.5664 0.3058 0.8688 0.4678 0.475 0.4488 0.0822 0.5556 0.6224 0.3314
Kidney: 1 0.5642 0.4288 0.9058 0.8494 0.8924 0.6002 0.2268 0.6056 0.5078 0.5772
Liver: 1 0.4416 0.7672 0.6022 0.7006 0.5184 0.271 0.5734 0.5808 0.682
Lung: 1 0.218 0.572 0.3302 0.429 0.3226 0.328 0.2678 0.3352
LymphNode: 1 0.7058 0.9178 0.7846 0.1086 0.8532 0.51 0.4118
Ovary: 1 0.73 0.4826 0.1478 0.7056 0.384 0.6018
Prostate: 1 0.5484 0.1214 0.75 0.5908 0.4322
SkeletalMuscle: 1 0.0782 0.2244 0.6964 0.423
Testes: 1 0.0568 0.1856 0.1472
Thyroid: 1 0.3618 0.8148
WhiteBloodCells: 1 0.2886
EdomStromalCell: 1
Placenta Adipose Adrenal Brain Breast Colon Heart Kidney Liver Lung LymphNode Ovary Prostate SkeletalMuscle Testes Thyroid WhiteBloodCells EdomStromalCell

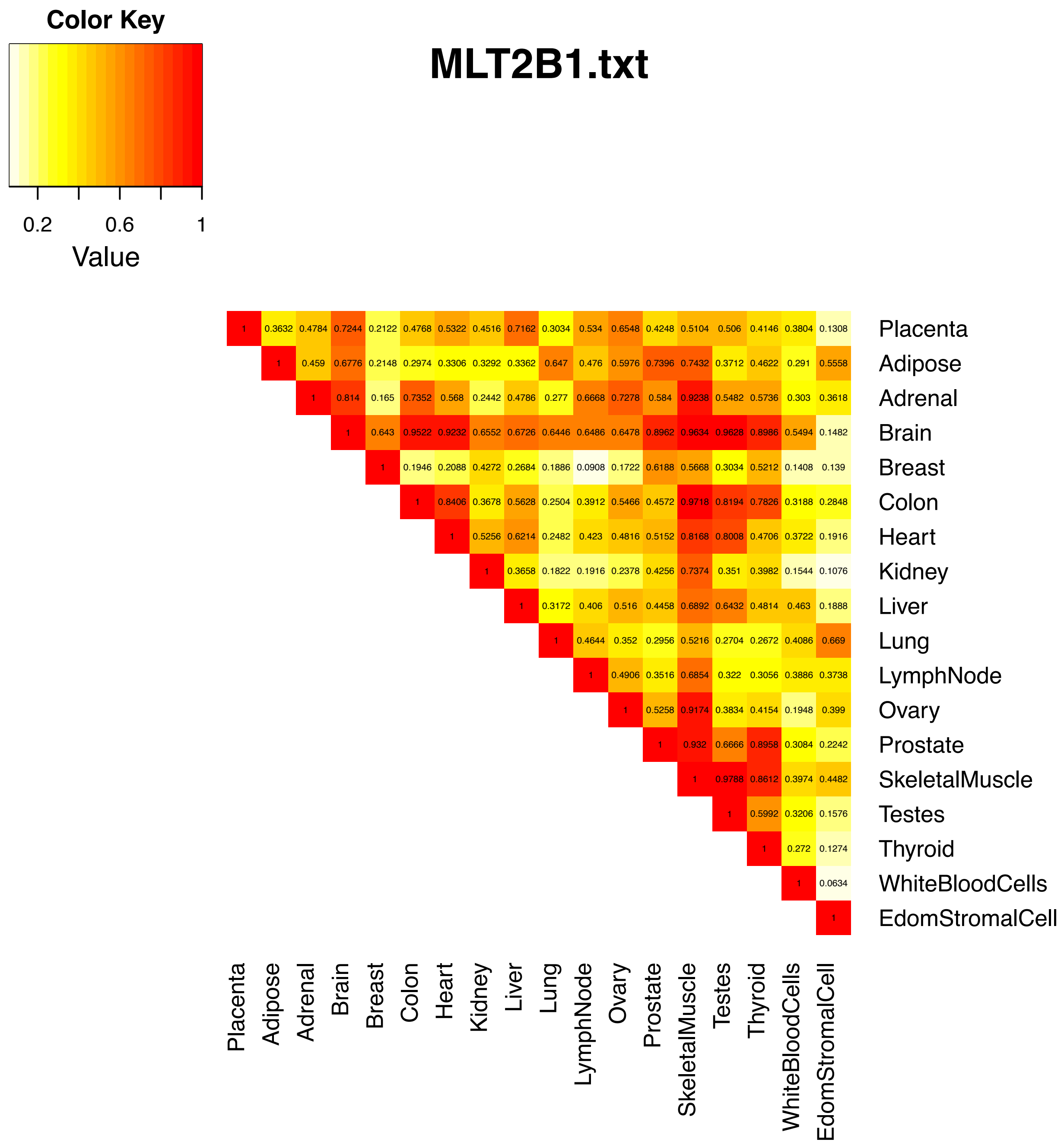
Color Key
0.2
0.6
1
Value
MLT2B1.txt
1 0.3632 0.4784 0.7244 0.2122 0.4768 0.5322 0.4516 0.7162 0.3034 0.534 0.6548 0.4248 0.5104 0.506 0.4146 0.3804 0.1308 Placenta
1 0.459 0.6776 0.2148 0.2974 0.3306 0.3292 0.3362 0.647 0.476 0.5976 0.7396 0.7432 0.3712 0.4622 0.291 0.5558 Adipose
1 0.814 0.165 0.7352 0.568 0.2442 0.4786 0.277 0.6668 0.7278 0.584 0.9238 0.5482 0.5736 0.303 0.3618 Adrenal
1 0.643 0.9522 0.9232 0.6552 0.6726 0.6446 0.6486 0.6478 0.8962 0.9634 0.9628 0.8986 0.5494 0.1482 Brain
1 0.1946 0.2088 0.4272 0.2684 0.1886 0.0908 0.1722 0.6188 0.5668 0.3034 0.5212 0.1408 0.139 Breast
1 0.8406 0.3678 0.5628 0.2504 0.3912 0.5466 0.4572 0.9718 0.8194 0.7826 0.3188 0.2848 Colon
1 0.5256 0.6214 0.2482 0.423 0.4816 0.5152 0.8168 0.8008 0.4706 0.3722 0.1916 Heart
1 0.3658 0.1822 0.1916 0.2378 0.4256 0.7374 0.351 0.3982 0.1544 0.1076 Kidney
1 0.3172 0.406 0.516 0.4458 0.6892 0.6432 0.4814 0.463 0.1888 Liver
1 0.4644 0.352 0.2956 0.5216 0.2704 0.2672 0.4086 0.669 Lung
1 0.4906 0.3516 0.6854 0.322 0.3056 0.3886 0.3738 LymphNode
1 0.5258 0.9174 0.3834 0.4154 0.1948 0.399 Ovary
1 0.932 0.6666 0.8958 0.3084 0.2242 Prostate
1 0.9788 0.8612 0.3974 0.4482 SkeletalMuscle
1 0.5992 0.3206 0.1576 Testes
1 0.272 0.1274 Thyroid
1 0.0634 WhiteBloodCells
1 EdomStromalCell
Placenta
Adipose
Adrenal
Brain
Breast
Colon
Heart
Kidney
Liver
Lung
LymphNode
Ovary
Prostate
SkeletalMuscle
Testes
Thyroid
WhiteBloodCells
EdomStromalCell

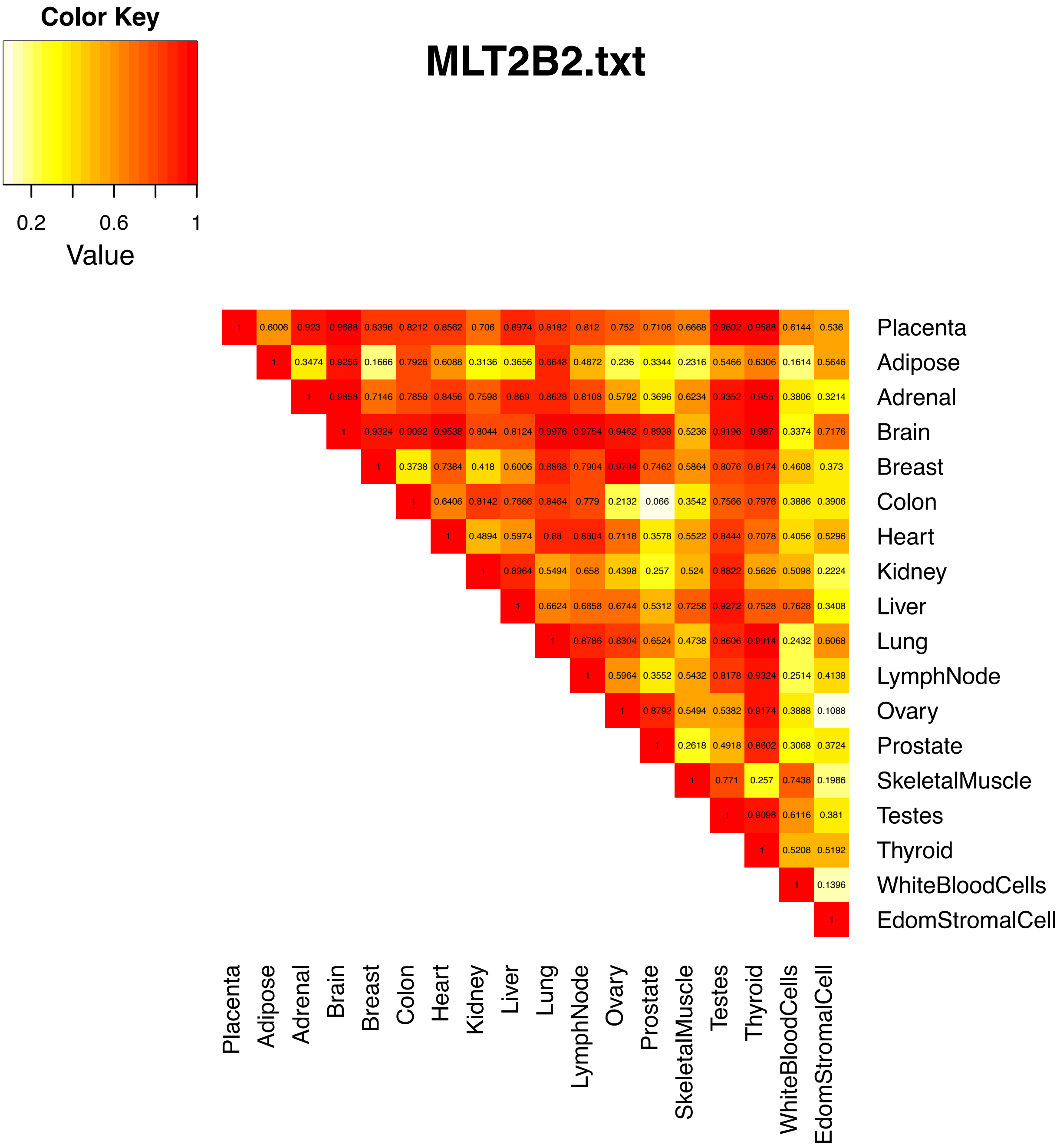
Color Key
0.2 0.6 1
Value
MLT2B2.txt
Placenta 1 0.6006 0.923 0.9688 0.8396 0.8212 0.8562 0.706 0.8974 0.8182 0.812 0.752 0.7106 0.6668 0.9602 0.9588 0.6144 0.536
Adipose 1 0.3474 0.9256 0.1666 0.7926 0.6088 0.3136 0.3656 0.8648 0.4872 0.236 0.3344 0.2316 0.5466 0.6306 0.1614 0.5646
Adrenal 1 0.9858 0.7146 0.7858 0.8456 0.7598 0.869 0.8628 0.8108 0.5792 0.3696 0.6234 0.9352 0.955 0.3806 0.3214
Brain 1 0.9324 0.9092 0.9538 0.8044 0.8124 0.9976 0.9754 0.9462 0.8938 0.5236 0.9196 0.987 0.3374 0.7176
Breast 1 0.3738 0.7384 0.418 0.6006 0.8868 0.7904 0.9704 0.7462 0.5864 0.8076 0.8174 0.4608 0.373
Colon 1 0.6406 0.8142 0.7666 0.8464 0.779 0.2132 0.066 0.3542 0.7566 0.7976 0.3886 0.3906
Heart 1 0.4894 0.5974 0.88 0.8804 0.7118 0.3578 0.5522 0.8444 0.7078 0.4056 0.5296
Kidney 1 0.8964 0.5494 0.658 0.4398 0.257 0.524 0.8822 0.5626 0.5098 0.2224
Liver 1 0.6624 0.6858 0.6744 0.5312 0.7258 0.9272 0.7528 0.7628 0.3408
Lung 1 0.8786 0.8304 0.6524 0.4738 0.8606 0.9914 0.2432 0.6068
LymphNode 1 0.5964 0.3552 0.5432 0.8178 0.9324 0.2514 0.4138
Ovary 1 0.8792 0.5494 0.5382 0.9174 0.3888 0.1088
Prostate 1 0.2618 0.4918 0.8602 0.3068 0.3724
SkeletalMuscle 1 0.771 0.257 0.7438 0.1986
Testes 1 0.9098 0.6116 0.381
Thyroid 1 0.5208 0.5192
WhiteBloodCells 1 0.1396
EdomStromalCell 1
Placenta Adipose Adrenal Brain Breast Colon Heart Kidney Liver Lung LymphNode Ovary Prostate SkeletalMuscle Testes Thyroid WhiteBloodCells EdomStromalCell

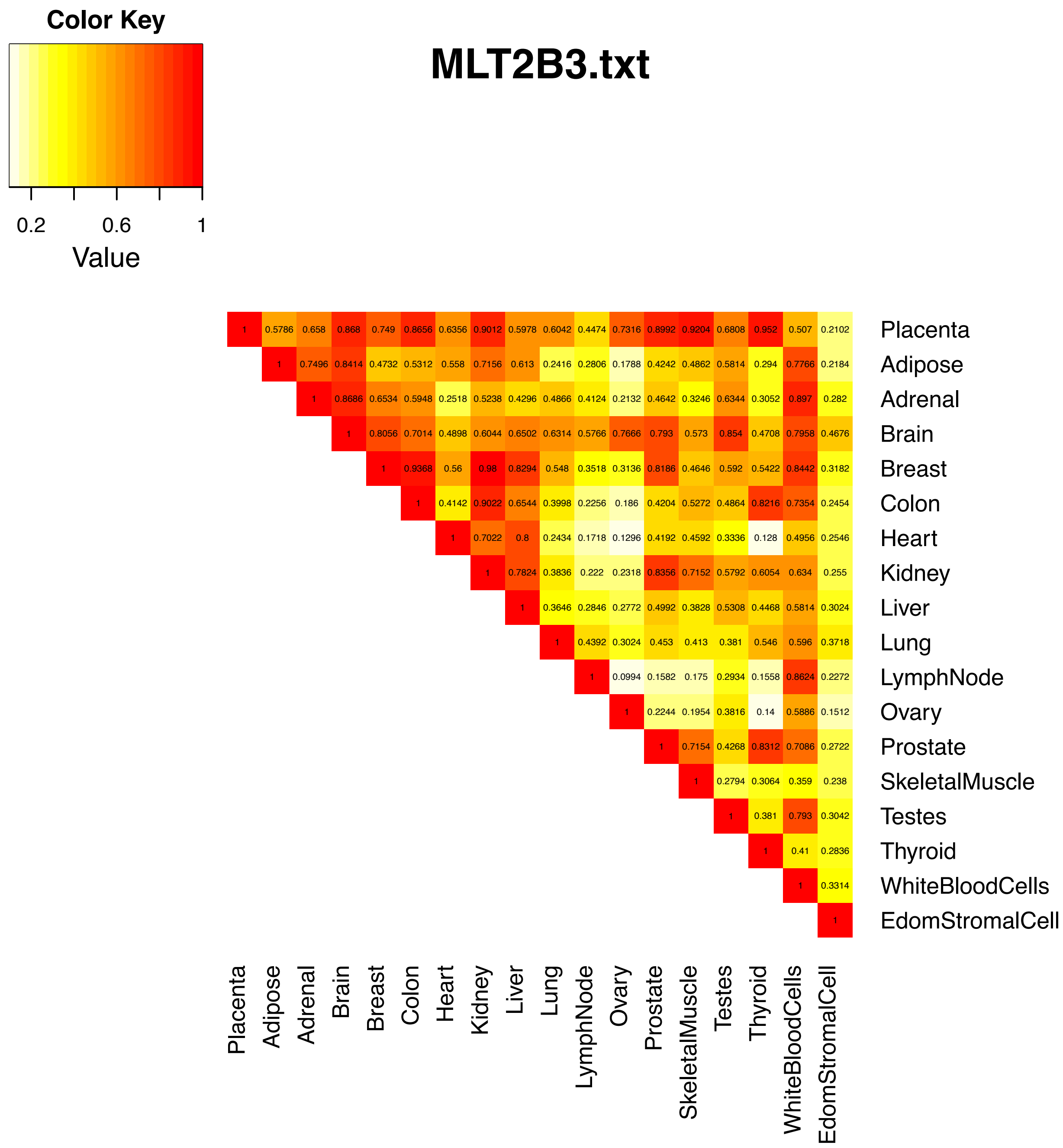
MLT2B3.txt
Color Key
0.2 0.6 1
Value
Placenta 1 0.5786 0.658 0.868 0.749 0.8656 0.6356 0.9012 0.5978 0.6042 0.4474 0.7316 0.8992 0.9204 0.6808 0.952 0.507 0.2102
Adipose 1 0.7496 0.8414 0.4732 0.5312 0.558 0.7156 0.613 0.2416 0.2806 0.1788 0.4242 0.4862 0.5814 0.294 0.7766 0.2184
Adrenal 1 0.8686 0.6534 0.5948 0.2518 0.5238 0.4296 0.4866 0.4124 0.2132 0.4642 0.3246 0.6344 0.3052 0.897 0.282
Brain 1 0.8056 0.7014 0.4898 0.6044 0.6502 0.6314 0.5766 0.7666 0.793 0.573 0.854 0.4708 0.7958 0.4676
Breast 1 0.9368 0.56 0.98 0.8294 0.548 0.3518 0.3136 0.8186 0.4646 0.592 0.5422 0.8442 0.3182
Colon 1 0.4142 0.9022 0.6544 0.3998 0.2256 0.186 0.4204 0.5272 0.4864 0.8216 0.7354 0.2454
Heart 1 0.7022 0.8 0.2434 0.1718 0.1296 0.4192 0.4592 0.3336 0.128 0.4956 0.2546
Kidney 1 0.7824 0.3836 0.222 0.2318 0.8356 0.7152 0.5792 0.6054 0.634 0.255
Liver 1 0.3646 0.2846 0.2772 0.4992 0.3828 0.5308 0.4468 0.5814 0.3024
Lung 1 0.4392 0.3024 0.453 0.413 0.381 0.546 0.596 0.3718
LymphNode 1 0.0994 0.1582 0.175 0.2934 0.1558 0.8624 0.2272
Ovary 1 0.2244 0.1954 0.3816 0.14 0.5886 0.1512
Prostate 1 0.7154 0.4268 0.8312 0.7086 0.2722
SkeletalMuscle 1 0.2794 0.3064 0.359 0.238
Testes 1 0.381 0.793 0.3042
Thyroid 1 0.41 0.2836
WhiteBloodCells 1 0.3314
EdomStromalCell 1
Placenta Adipose Adrenal Brain Breast Colon Heart Kidney Liver Lung LymphNode Ovary Prostate SkeletalMuscle Testes Thyroid WhiteBloodCells EdomStromalCell

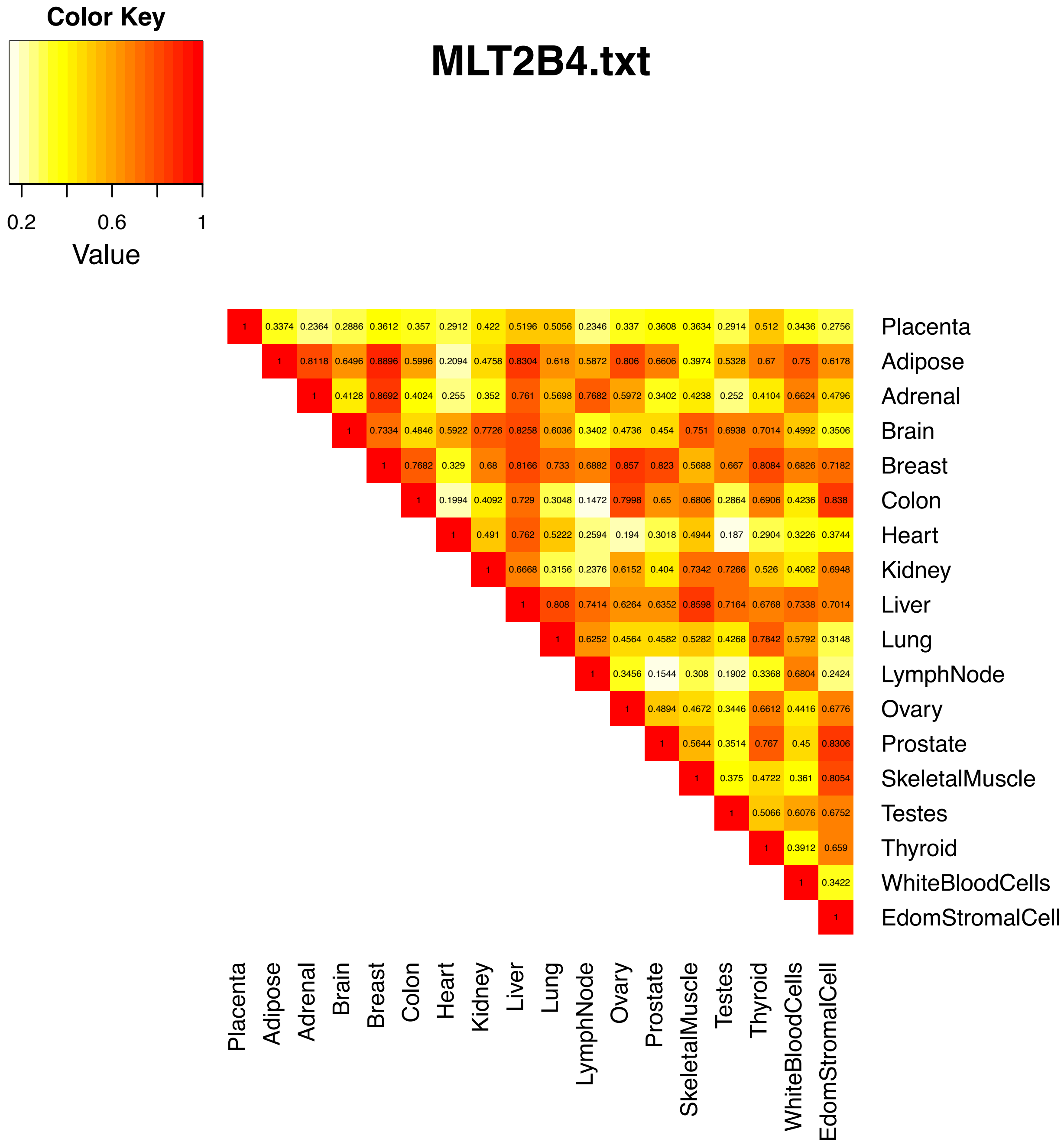
Color Key
0.2 0.6 1
Value
MLT2B4.txt
Placenta: 1 0.3374 0.2364 0.2886 0.3612 0.357 0.2912 0.422 0.5196 0.5056 0.2346 0.337 0.3608 0.3634 0.2914 0.512 0.3436 0.2756
Adipose: 1 0.8118 0.6496 0.8896 0.5996 0.2094 0.4758 0.8304 0.618 0.5872 0.806 0.6606 0.3974 0.5328 0.67 0.75 0.6178
Adrenal: 1 0.4128 0.8692 0.4024 0.255 0.352 0.761 0.5698 0.7682 0.5972 0.3402 0.4238 0.252 0.4104 0.6624 0.4796
Brain: 1 0.7334 0.4846 0.5922 0.7726 0.8258 0.6036 0.3402 0.4736 0.454 0.751 0.6938 0.7014 0.4992 0.3506
Breast: 1 0.7682 0.329 0.68 0.8166 0.733 0.6882 0.857 0.823 0.5688 0.667 0.8084 0.6826 0.7182
Colon: 1 0.1994 0.4092 0.729 0.3048 0.1472 0.7998 0.65 0.6806 0.2864 0.6906 0.4236 0.838
Heart: 1 0.491 0.762 0.5222 0.2594 0.194 0.3018 0.4944 0.187 0.2904 0.3226 0.3744
Kidney: 1 0.6668 0.3156 0.2376 0.6152 0.404 0.7342 0.7266 0.526 0.4062 0.6948
Liver: 1 0.808 0.7414 0.6264 0.6352 0.8598 0.7164 0.6768 0.7338 0.7014
Lung: 1 0.6252 0.4564 0.4582 0.5282 0.4268 0.7842 0.5792 0.3148
LymphNode: 1 0.3456 0.1544 0.308 0.1902 0.3368 0.6804 0.2424
Ovary: 1 0.4894 0.4672 0.3446 0.6612 0.4416 0.6776
Prostate: 1 0.5644 0.3514 0.767 0.45 0.8306
SkeletalMuscle: 1 0.375 0.4722 0.361 0.8054
Testes: 1 0.5066 0.6076 0.6752
Thyroid: 1 0.3912 0.659
WhiteBloodCells: 1 0.3422
EdomStromalCell: 1
Placenta Adipose Adrenal Brain Breast Colon Heart Kidney Liver Lung LymphNode Ovary Prostate SkeletalMuscle Testes Thyroid WhiteBloodCells EdomStromalCell

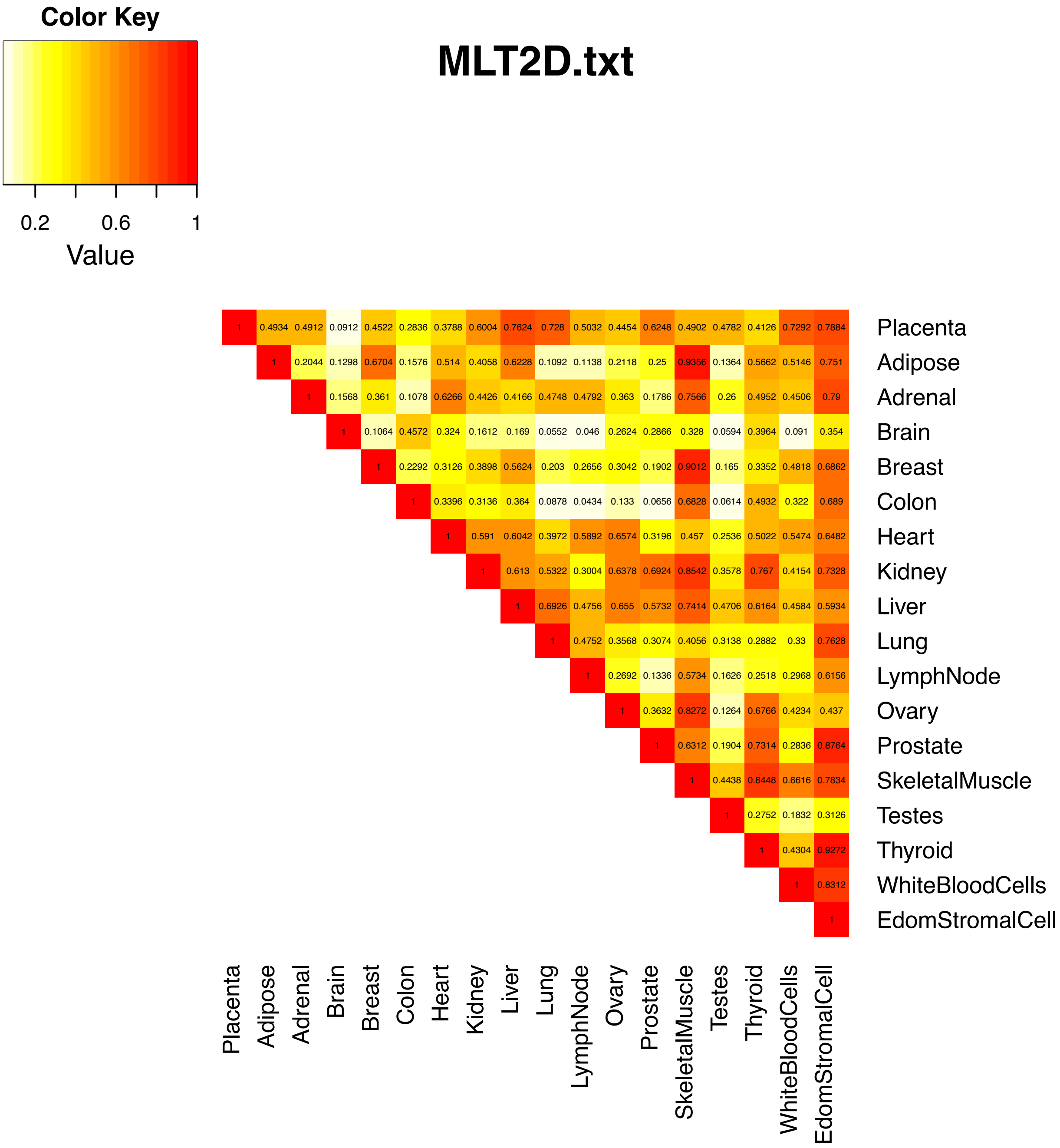

Color Key
0.2
0.6
1
Value
MLT2D.txt
Placenta: 1 0.4934 0.4912 0.0912 0.4522 0.2836 0.3788 0.6004 0.7624 0.728 0.5032 0.4454 0.6248 0.4902 0.4782 0.4126 0.7292 0.7884
Adipose: 1 0.2044 0.1298 0.6704 0.1576 0.514 0.4058 0.6228 0.1092 0.1138 0.2118 0.25 0.9356 0.1364 0.5662 0.5146 0.751
Adrenal: 1 0.1568 0.361 0.1078 0.6266 0.4426 0.4166 0.4748 0.4792 0.363 0.1786 0.7566 0.26 0.4952 0.4506 0.79
Brain: 1 0.1064 0.4572 0.324 0.1612 0.169 0.0552 0.046 0.2624 0.2866 0.328 0.0594 0.3964 0.091 0.354
Breast: 1 0.2292 0.3126 0.3898 0.5624 0.203 0.2656 0.3042 0.1902 0.9012 0.165 0.3352 0.4818 0.6862
Colon: 1 0.3396 0.3136 0.364 0.0878 0.0434 0.133 0.0656 0.6828 0.0614 0.4932 0.322 0.689
Heart: 1 0.591 0.6042 0.3972 0.5892 0.6574 0.3196 0.457 0.2536 0.5022 0.5474 0.6482
Kidney: 1 0.613 0.5322 0.3004 0.6378 0.6924 0.8542 0.3578 0.767 0.4154 0.7328
Liver: 1 0.6926 0.4756 0.655 0.5732 0.7414 0.4706 0.6164 0.4584 0.5934
Lung: 1 0.4752 0.3568 0.3074 0.4056 0.3138 0.2882 0.33 0.7628
LymphNode: 1 0.2692 0.1336 0.5734 0.1626 0.2518 0.2968 0.6156
Ovary: 1 0.3632 0.8272 0.1264 0.6766 0.4234 0.437
Prostate: 1 0.6312 0.1904 0.7314 0.2836 0.8764
SkeletalMuscle: 1 0.4438 0.8448 0.6616 0.7834
Testes: 1 0.2752 0.1832 0.3126
Thyroid: 1 0.4304 0.9272
WhiteBloodCells: 1 0.8312
EdomStromalCell: 1
Placenta Adipose Adrenal Brain Breast Colon Heart Kidney Liver Lung LymphNode Ovary Prostate SkeletalMuscle Testes Thyroid WhiteBloodCells EdomStromalCell

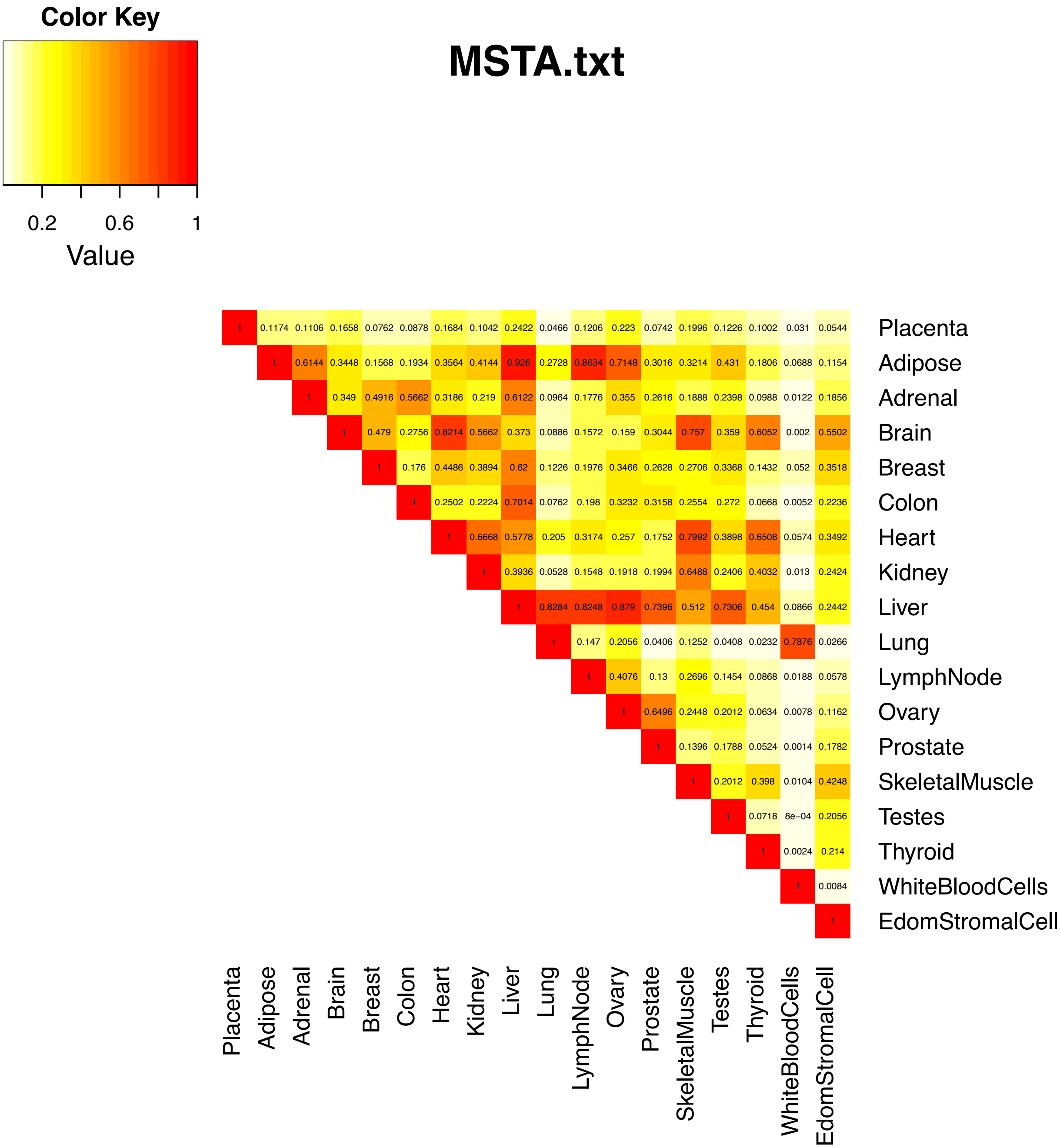
Color Key
0.2 0.6 1
Value
MSTA.txt
1 0.1174 0.1106 0.1658 0.0762 0.0878 0.1684 0.1042 0.2422 0.0466 0.1206 0.223 0.0742 0.1996 0.1226 0.1002 0.031 0.0544 Placenta
1 0.6144 0.3448 0.1568 0.1934 0.3564 0.4144 0.926 0.2728 0.8634 0.7148 0.3016 0.3214 0.431 0.1806 0.0688 0.1154 Adipose
1 0.349 0.4916 0.5662 0.3186 0.219 0.6122 0.0964 0.1776 0.355 0.2616 0.1888 0.2398 0.0988 0.0122 0.1856 Adrenal
1 0.479 0.2756 0.8214 0.5662 0.373 0.0886 0.1572 0.159 0.3044 0.757 0.359 0.6052 0.002 0.5502 Brain
1 0.176 0.4486 0.3894 0.62 0.1226 0.1976 0.3466 0.2628 0.2706 0.3368 0.1432 0.052 0.3518 Breast
1 0.2502 0.2224 0.7014 0.0762 0.198 0.3232 0.3158 0.2554 0.272 0.0668 0.0052 0.2236 Colon
1 0.6668 0.5778 0.205 0.3174 0.257 0.1752 0.7992 0.3898 0.6508 0.0574 0.3492 Heart
1 0.3936 0.0528 0.1548 0.1918 0.1994 0.6488 0.2406 0.4032 0.013 0.2424 Kidney
1 0.8284 0.8248 0.879 0.7396 0.512 0.7306 0.454 0.0866 0.2442 Liver
1 0.147 0.2056 0.0406 0.1252 0.0408 0.0232 0.7876 0.0266 Lung
1 0.4076 0.13 0.2696 0.1454 0.0868 0.0188 0.0578 LymphNode
1 0.6496 0.2448 0.2012 0.0634 0.0078 0.1162 Ovary
1 0.1396 0.1788 0.0524 0.0014 0.1782 Prostate
1 0.2012 0.398 0.0104 0.4248 SkeletalMuscle
1 0.0718 8e-04 0.2056 Testes
1 0.0024 0.214 Thyroid
1 0.0084 WhiteBloodCells
1 EdomStromalCell
Placenta Adipose Adrenal Brain Breast Colon Heart Kidney Liver Lung LymphNode Ovary Prostate SkeletalMuscle Testes Thyroid WhiteBloodCells EdomStromalCell

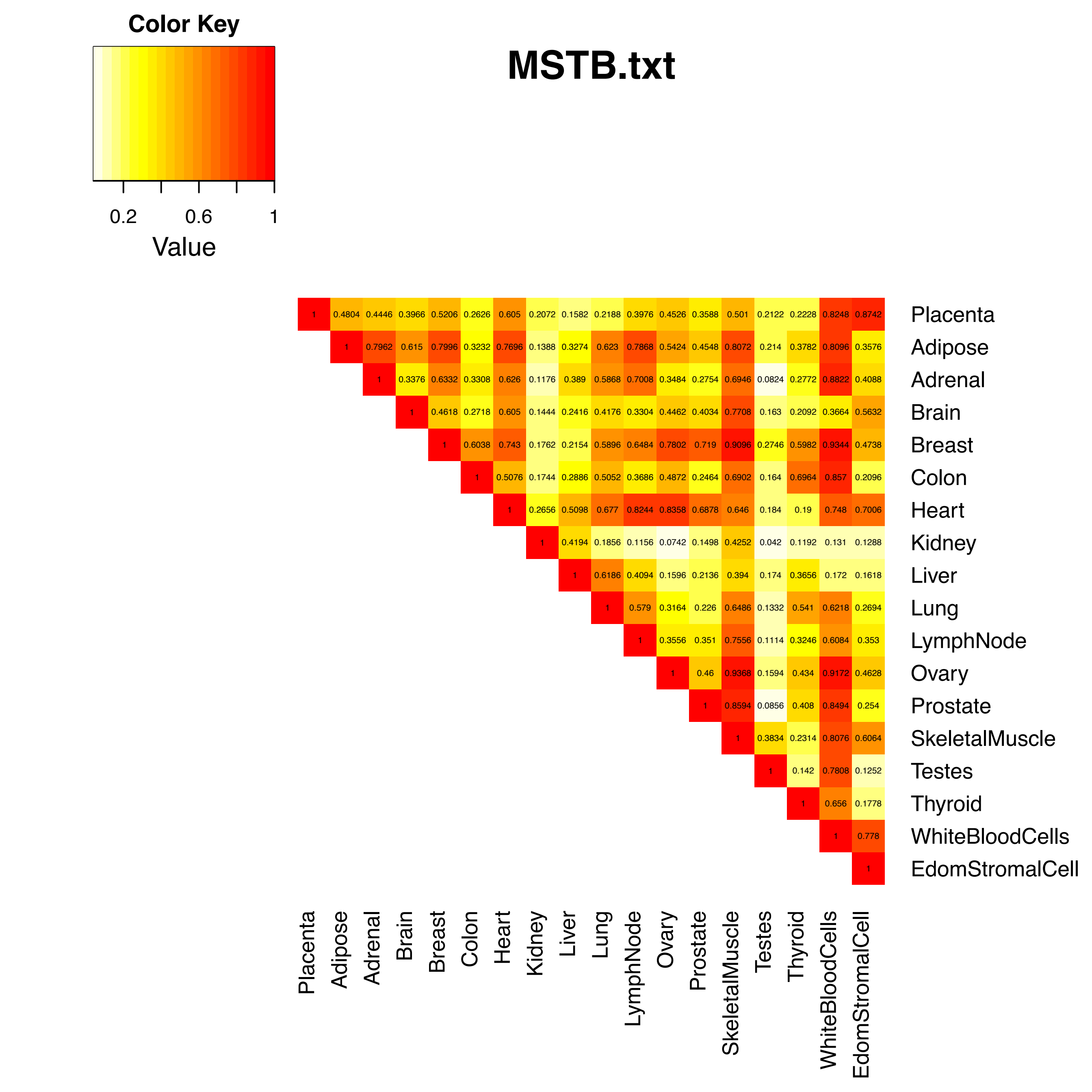
Color Key
0.2 0.6 1
Value
MSTB.txt
Placenta 1 0.4804 0.4446 0.3966 0.5206 0.2626 0.605 0.2072 0.1582 0.2188 0.3976 0.4526 0.3588 0.501 0.2122 0.2228 0.8248 0.8742
Adipose 1 0.7962 0.615 0.7996 0.3232 0.7696 0.1388 0.3274 0.623 0.7868 0.5424 0.4548 0.8072 0.214 0.3782 0.8096 0.3576
Adrenal 1 0.3376 0.6332 0.3308 0.626 0.1176 0.389 0.5868 0.7008 0.3484 0.2754 0.6946 0.0824 0.2772 0.8822 0.4088
Brain 1 0.4618 0.2718 0.605 0.1444 0.2416 0.4176 0.3304 0.4462 0.4034 0.7708 0.163 0.2092 0.3664 0.5632
Breast 1 0.6038 0.743 0.1762 0.2154 0.5896 0.6484 0.7802 0.719 0.9096 0.2746 0.5982 0.9344 0.4738
Colon 1 0.5076 0.1744 0.2886 0.5052 0.3686 0.4872 0.2464 0.6902 0.164 0.6964 0.857 0.2096
Heart 1 0.2656 0.5098 0.677 0.8244 0.8358 0.6878 0.646 0.184 0.19 0.748 0.7006
Kidney 1 0.4194 0.1856 0.1156 0.0742 0.1498 0.4252 0.042 0.1192 0.131 0.1288
Liver 1 0.6186 0.4094 0.1596 0.2136 0.394 0.174 0.3656 0.172 0.1618
Lung 1 0.579 0.3164 0.226 0.6486 0.1332 0.541 0.6218 0.2694
LymphNode 1 0.3556 0.351 0.7556 0.1114 0.3246 0.6084 0.353
Ovary 1 0.46 0.9368 0.1594 0.434 0.9172 0.4628
Prostate 1 0.8594 0.0856 0.408 0.8494 0.254
SkeletalMuscle 1 0.3834 0.2314 0.8076 0.6064
Testes 1 0.142 0.7808 0.1252
Thyroid 1 0.656 0.1778
WhiteBloodCells 1 0.778
EdomStromalCell 1
Placenta Adipose Adrenal Brain Breast Colon Heart Kidney Liver Lung LymphNode Ovary Prostate SkeletalMuscle Testes Thyroid WhiteBloodCells EdomStromalCell

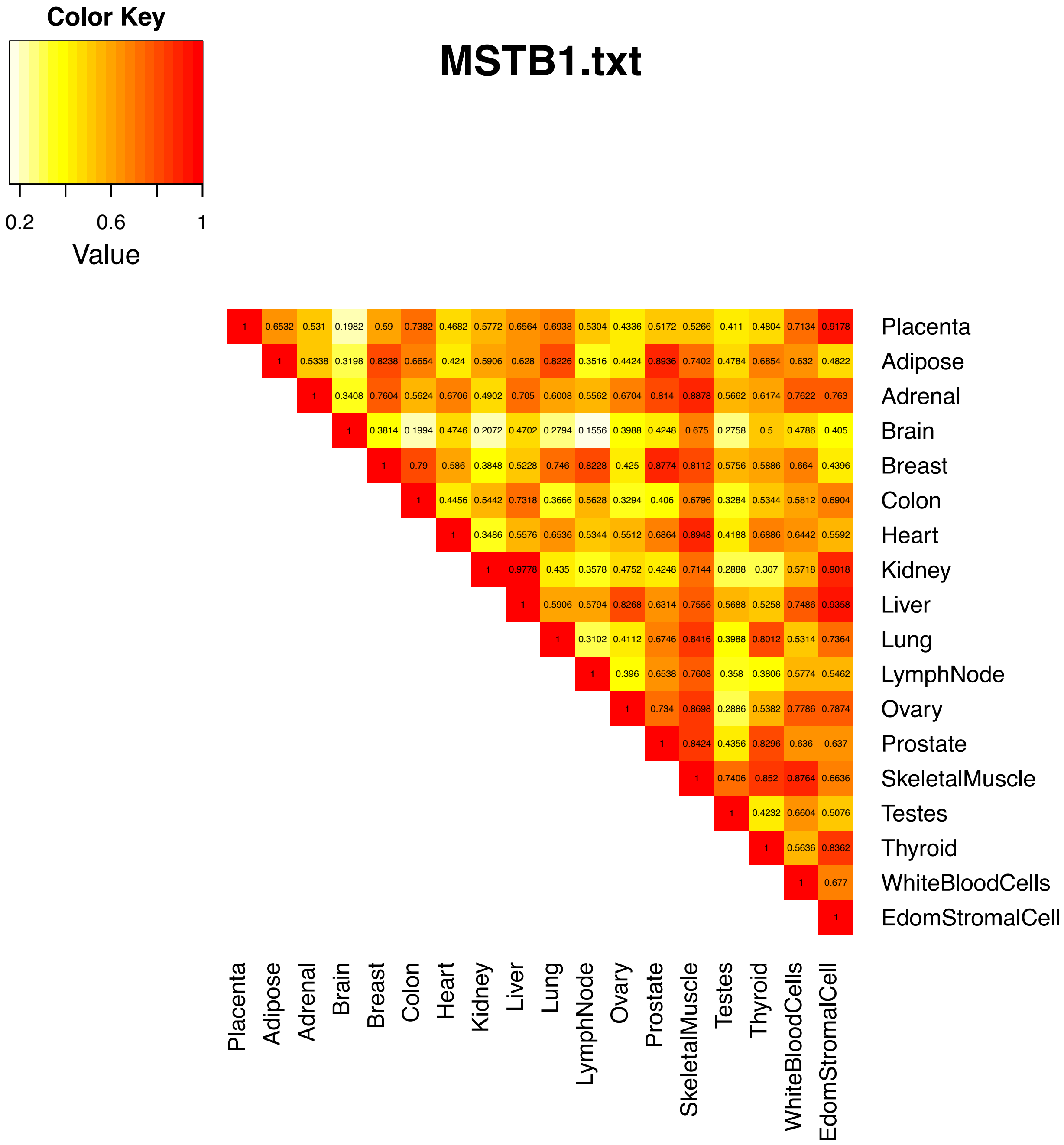
Color Key
0.2
0.6
1
Value
MSTB1.txt
Placenta 1 0.6532 0.531 0.1982 0.59 0.7382 0.4682 0.5772 0.6564 0.6938 0.5304 0.4336 0.5172 0.5266 0.411 0.4804 0.7134 0.9178
Adipose 1 0.5338 0.3198 0.8238 0.6654 0.424 0.5906 0.628 0.8226 0.3516 0.4424 0.8936 0.7402 0.4784 0.6854 0.632 0.4822
Adrenal 1 0.3408 0.7604 0.5624 0.6706 0.4902 0.705 0.6008 0.5562 0.6704 0.814 0.8878 0.5662 0.6174 0.7622 0.763
Brain 1 0.3814 0.1994 0.4746 0.2072 0.4702 0.2794 0.1556 0.3988 0.4248 0.675 0.2758 0.5 0.4786 0.405
Breast 1 0.79 0.586 0.3848 0.5228 0.746 0.8228 0.425 0.8774 0.8112 0.5756 0.5886 0.664 0.4396
Colon 1 0.4456 0.5442 0.7318 0.3666 0.5628 0.3294 0.406 0.6796 0.3284 0.5344 0.5812 0.6904
Heart 1 0.3486 0.5576 0.6536 0.5344 0.5512 0.6864 0.8948 0.4188 0.6886 0.6442 0.5592
Kidney 1 0.9778 0.435 0.3578 0.4752 0.4248 0.7144 0.2888 0.307 0.5718 0.9018
Liver 1 0.5906 0.5794 0.8268 0.6314 0.7556 0.5688 0.5258 0.7486 0.9358
Lung 1 0.3102 0.4112 0.6746 0.8416 0.3988 0.8012 0.5314 0.7364
LymphNode 1 0.396 0.6538 0.7608 0.358 0.3806 0.5774 0.5462
Ovary 1 0.734 0.8698 0.2886 0.5382 0.7786 0.7874
Prostate 1 0.8424 0.4356 0.8296 0.636 0.637
SkeletalMuscle 1 0.7406 0.852 0.8764 0.6636
Testes 1 0.4232 0.6604 0.5076
Thyroid 1 0.5636 0.8362
WhiteBloodCells 1 0.677
EdomStromalCell 1
Placenta Adipose Adrenal Brain Breast Colon Heart Kidney Liver Lung LymphNode Ovary Prostate SkeletalMuscle Testes Thyroid WhiteBloodCells EdomStromalCell

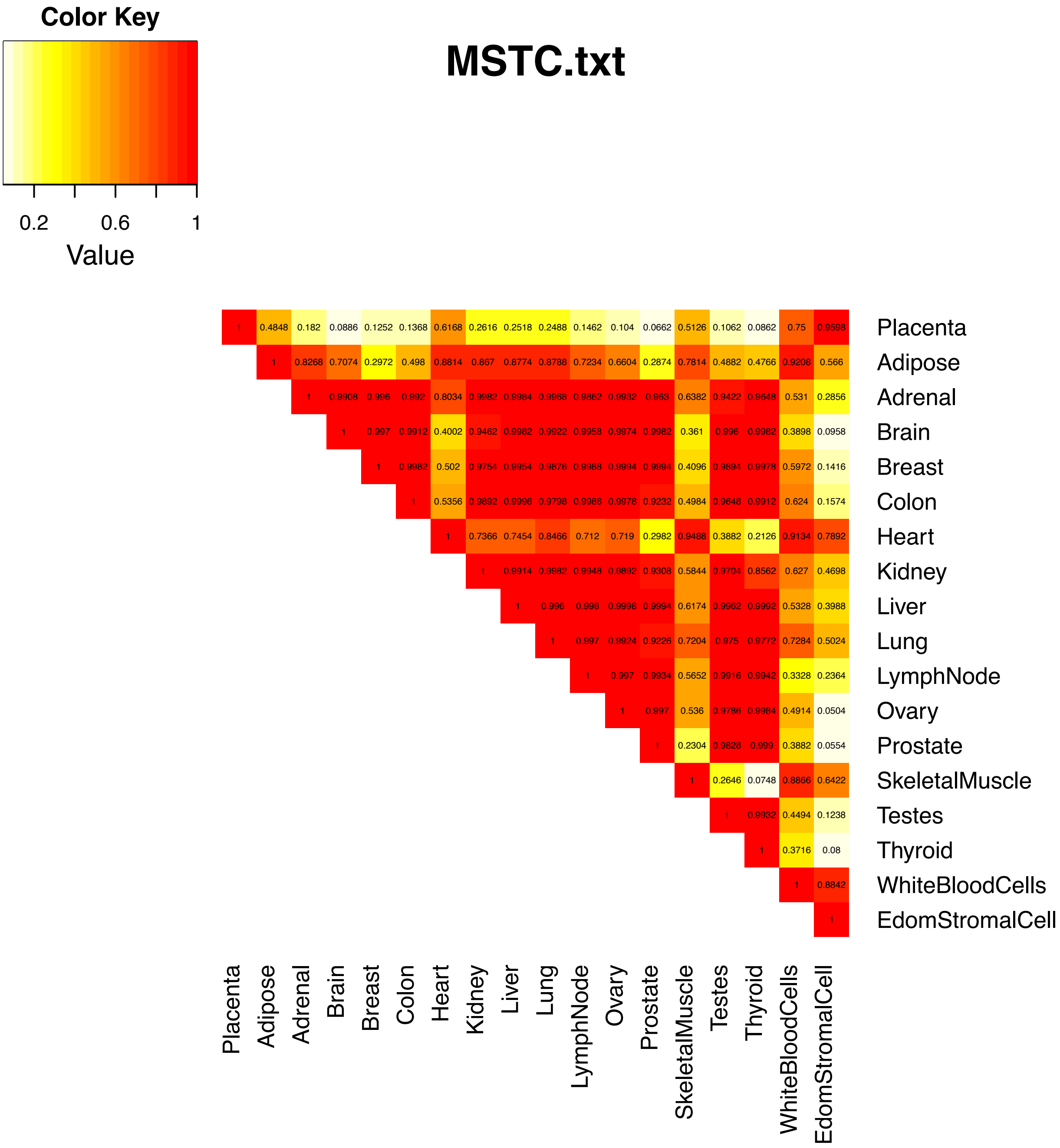
MSTC.txt
Color Key
0.2 0.6 1
Value
Placenta 1 0.4848 0.182 0.0886 0.1252 0.1368 0.6168 0.2616 0.2518 0.2488 0.1462 0.104 0.0662 0.5126 0.1062 0.0862 0.75 0.9598
Adipose 1 0.8268 0.7074 0.2972 0.498 0.8814 0.867 0.8774 0.8788 0.7234 0.6604 0.2874 0.7814 0.4882 0.4766 0.9208 0.566
Adrenal 1 0.9908 0.996 0.992 0.8034 0.9982 0.9984 0.9968 0.9862 0.9932 0.963 0.6382 0.9422 0.9648 0.531 0.2856
Brain 1 0.997 0.9912 0.4002 0.9462 0.9982 0.9922 0.9958 0.9974 0.9982 0.361 0.996 0.9982 0.3898 0.0958
Breast 1 0.9982 0.502 0.9754 0.9954 0.9876 0.9988 0.9994 0.9994 0.4096 0.9894 0.9978 0.5972 0.1416
Colon 1 0.5356 0.9892 0.9996 0.9798 0.9986 0.9978 0.9232 0.4984 0.9648 0.9912 0.624 0.1574
Heart 1 0.7366 0.7454 0.8466 0.712 0.719 0.2982 0.9488 0.3882 0.2126 0.9134 0.7892
Kidney 1 0.9914 0.9982 0.9948 0.9892 0.9308 0.5844 0.9704 0.8562 0.627 0.4698
Liver 1 0.996 0.998 0.9998 0.9994 0.6174 0.9962 0.9992 0.5328 0.3988
Lung 1 0.997 0.9924 0.9226 0.7204 0.975 0.9772 0.7284 0.5024
LymphNode 1 0.997 0.9934 0.5652 0.9916 0.9942 0.3328 0.2364
Ovary 1 0.997 0.536 0.9786 0.9984 0.4914 0.0504
Prostate 1 0.2304 0.9828 0.999 0.3882 0.0554
SkeletalMuscle 1 0.2646 0.0748 0.8866 0.6422
Testes 1 0.9932 0.4494 0.1238
Thyroid 1 0.3716 0.08
WhiteBloodCells 1 0.8842
EdomStromalCell 1
Placenta Adipose Adrenal Brain Breast Colon Heart Kidney Liver Lung LymphNode Ovary Prostate SkeletalMuscle Testes Thyroid WhiteBloodCells EdomStromalCell

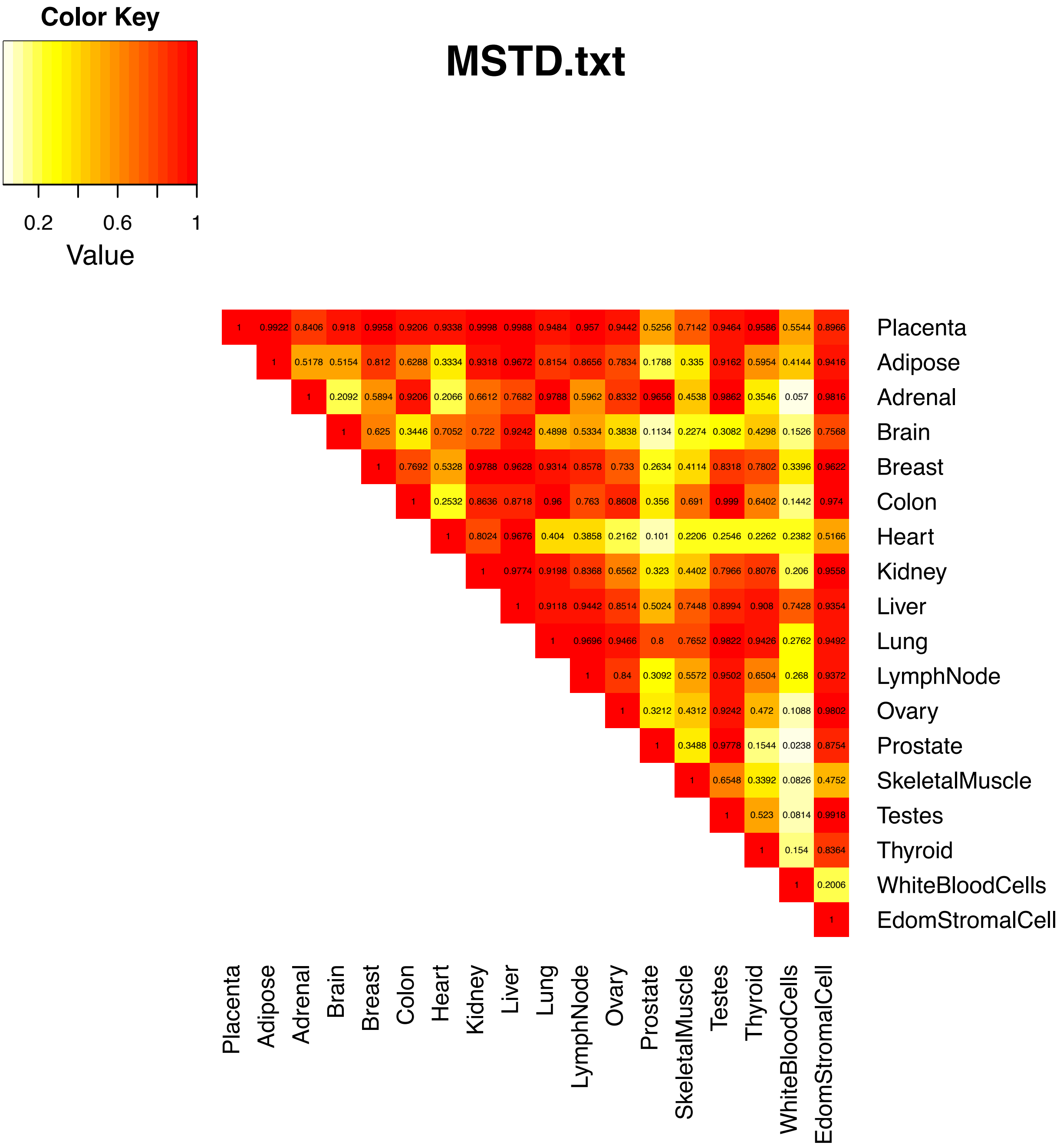
Color Key
0.2
0.6
1
Value
MSTD.txt
Placenta: 1 0.9922 0.8406 0.918 0.9958 0.9206 0.9338 0.9998 0.9988 0.9484 0.957 0.9442 0.5256 0.7142 0.9464 0.9586 0.5544 0.8966
Adipose: 1 0.5178 0.5154 0.812 0.6288 0.3334 0.9318 0.9672 0.8154 0.8656 0.7834 0.1788 0.335 0.9162 0.5954 0.4144 0.9416
Adrenal: 1 0.2092 0.5894 0.9206 0.2066 0.6612 0.7682 0.9788 0.5962 0.8332 0.9656 0.4538 0.9862 0.3546 0.057 0.9816
Brain: 1 0.625 0.3446 0.7052 0.722 0.9242 0.4898 0.5334 0.3838 0.1134 0.2274 0.3082 0.4298 0.1526 0.7568
Breast: 1 0.7692 0.5328 0.9788 0.9628 0.9314 0.8578 0.733 0.2634 0.4114 0.8318 0.7802 0.3396 0.9622
Colon: 1 0.2532 0.8636 0.8718 0.96 0.763 0.8608 0.356 0.691 0.999 0.6402 0.1442 0.974
Heart: 1 0.8024 0.9676 0.404 0.3858 0.2162 0.101 0.2206 0.2546 0.2262 0.2382 0.5166
Kidney: 1 0.9774 0.9198 0.8368 0.6562 0.323 0.4402 0.7966 0.8076 0.206 0.9558
Liver: 1 0.9118 0.9442 0.8514 0.5024 0.7448 0.8994 0.908 0.7428 0.9354
Lung: 1 0.9696 0.9466 0.8 0.7652 0.9822 0.9426 0.2762 0.9492
LymphNode: 1 0.84 0.3092 0.5572 0.9502 0.6504 0.268 0.9372
Ovary: 1 0.3212 0.4312 0.9242 0.472 0.1088 0.9802
Prostate: 1 0.3488 0.9778 0.1544 0.0238 0.8754
SkeletalMuscle: 1 0.6548 0.3392 0.0826 0.4752
Testes: 1 0.523 0.0814 0.9918
Thyroid: 1 0.154 0.8364
WhiteBloodCells: 1 0.2006
EdomStromalCell: 1
Placenta Adipose Adrenal Brain Breast Colon Heart Kidney Liver Lung LymphNode Ovary Prostate SkeletalMuscle Testes Thyroid WhiteBloodCells EdomStromalCell

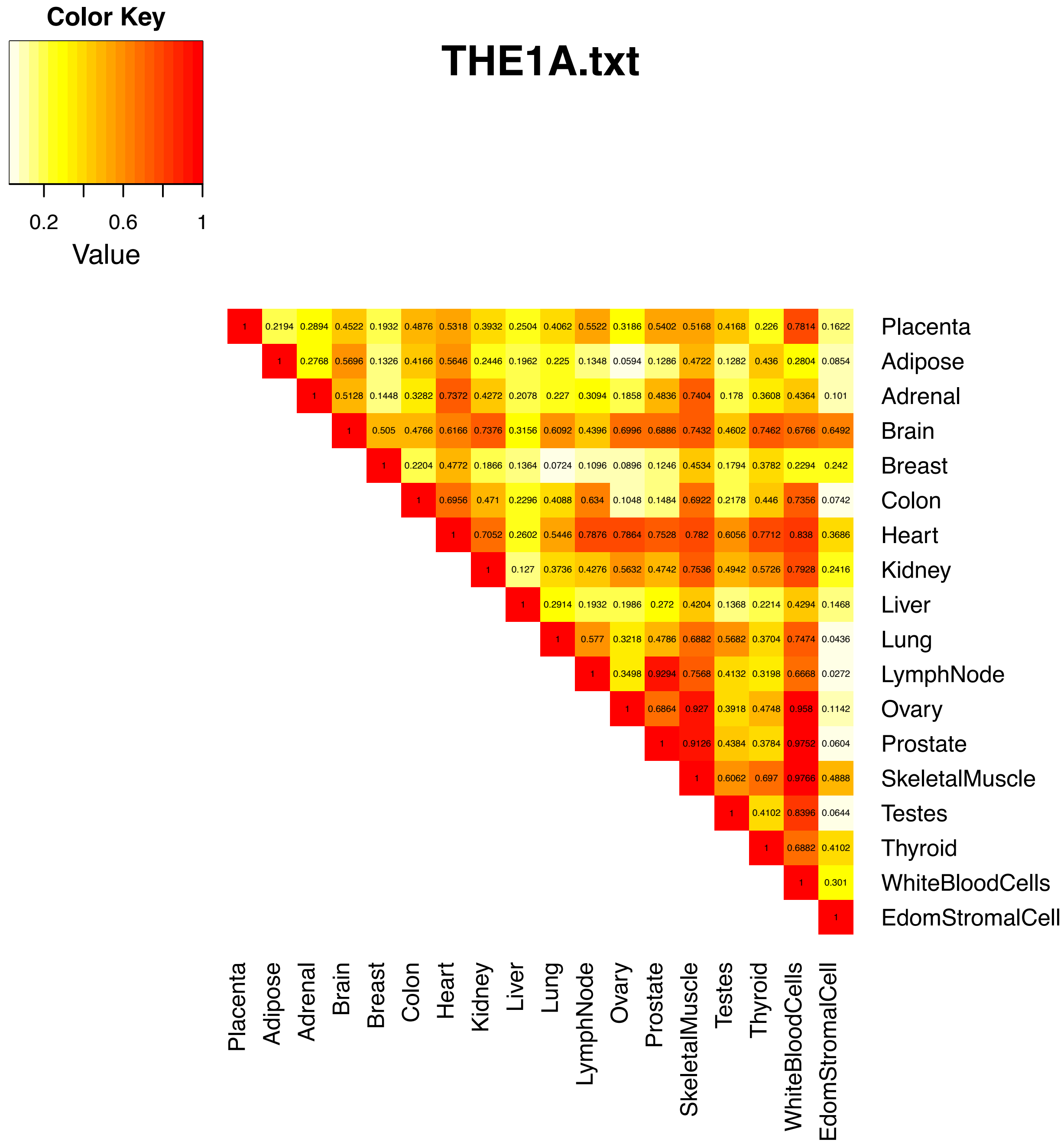
THE1A.txt
Color Key
0.2 0.6 1
Value
Placenta 1 0.2194 0.2894 0.4522 0.1932 0.4876 0.5318 0.3932 0.2504 0.4062 0.5522 0.3186 0.5402 0.5168 0.4168 0.226 0.7814 0.1622
Adipose 1 0.2768 0.5696 0.1326 0.4166 0.5646 0.2446 0.1962 0.225 0.1348 0.0594 0.1286 0.4722 0.1282 0.436 0.2804 0.0854
Adrenal 1 0.5128 0.1448 0.3282 0.7372 0.4272 0.2078 0.227 0.3094 0.1858 0.4836 0.7404 0.178 0.3608 0.4364 0.101
Brain 1 0.505 0.4766 0.6166 0.7376 0.3156 0.6092 0.4396 0.6996 0.6886 0.7432 0.4602 0.7462 0.6766 0.6492
Breast 1 0.2204 0.4772 0.1866 0.1364 0.0724 0.1096 0.0896 0.1246 0.4534 0.1794 0.3782 0.2294 0.242
Colon 1 0.6956 0.471 0.2296 0.4088 0.634 0.1048 0.1484 0.6922 0.2178 0.446 0.7356 0.0742
Heart 1 0.7052 0.2602 0.5446 0.7876 0.7864 0.7528 0.782 0.6056 0.7712 0.838 0.3686
Kidney 1 0.127 0.3736 0.4276 0.5632 0.4742 0.7536 0.4942 0.5726 0.7928 0.2416
Liver 1 0.2914 0.1932 0.1986 0.272 0.4204 0.1368 0.2214 0.4294 0.1468
Lung 1 0.577 0.3218 0.4786 0.6882 0.5682 0.3704 0.7474 0.0436
LymphNode 1 0.3498 0.9294 0.7568 0.4132 0.3198 0.6668 0.0272
Ovary 1 0.6864 0.927 0.3918 0.4748 0.958 0.1142
Prostate 1 0.9126 0.4384 0.3784 0.9752 0.0604
SkeletalMuscle 1 0.6062 0.697 0.9766 0.4888
Testes 1 0.4102 0.8396 0.0644
Thyroid 1 0.6882 0.4102
WhiteBloodCells 1 0.301
EdomStromalCell 1
Placenta Adipose Adrenal Brain Breast Colon Heart Kidney Liver Lung LymphNode Ovary Prostate SkeletalMuscle Testes Thyroid WhiteBloodCells EdomStromalCell

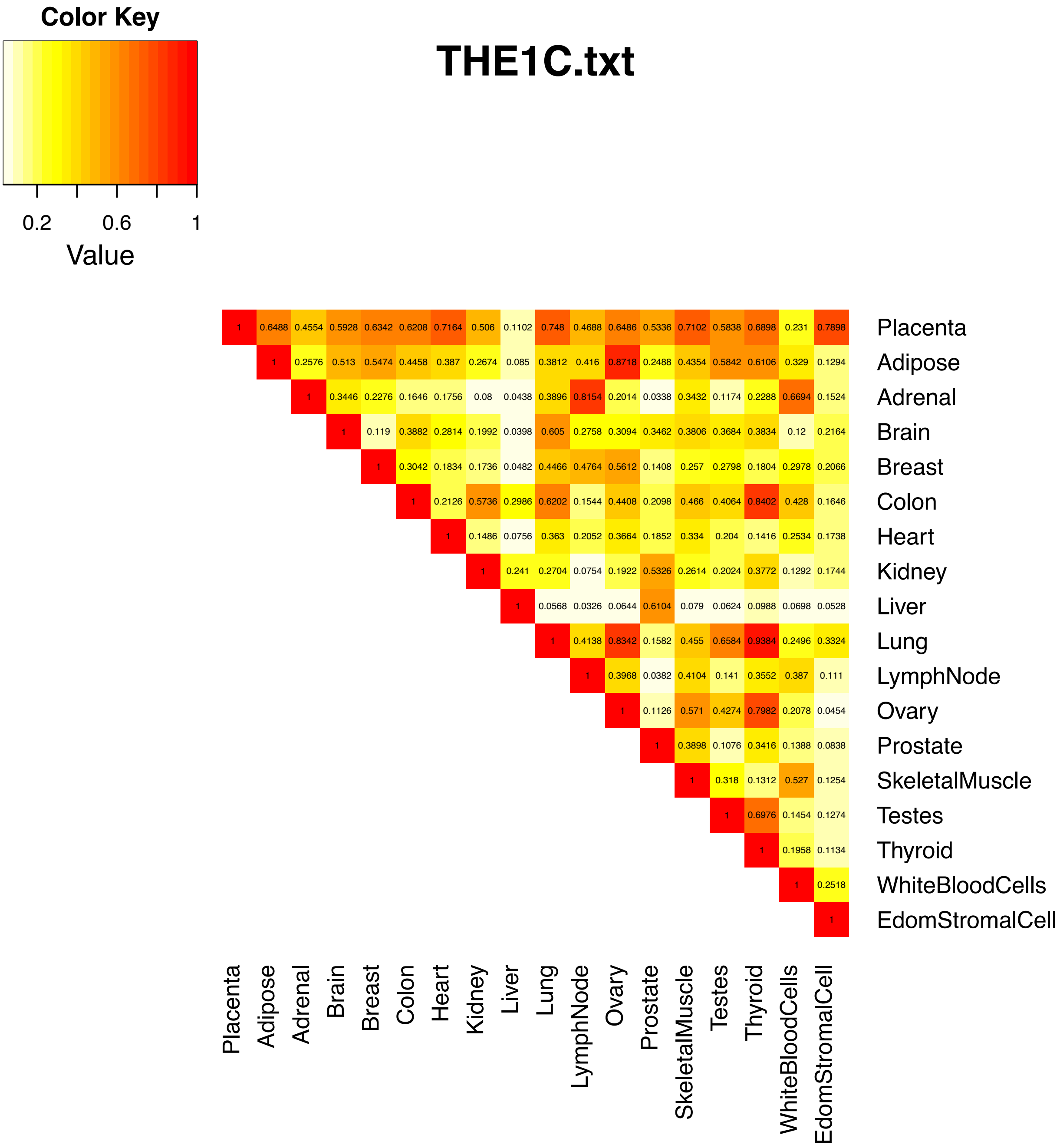
Color Key
0.2
0.6
1
Value
THE1C.txt
Placenta 1 0.6488 0.4554 0.5928 0.6342 0.6208 0.7164 0.506 0.1102 0.748 0.4688 0.6486 0.5336 0.7102 0.5838 0.6898 0.231 0.7898
Adipose 1 0.2576 0.513 0.5474 0.4458 0.387 0.2674 0.085 0.3812 0.416 0.8718 0.2488 0.4354 0.5842 0.6106 0.329 0.1294
Adrenal 1 0.3446 0.2276 0.1646 0.1756 0.08 0.0438 0.3896 0.8154 0.2014 0.0338 0.3432 0.1174 0.2288 0.6694 0.1524
Brain 1 0.119 0.3882 0.2814 0.1992 0.0398 0.605 0.2758 0.3094 0.3462 0.3806 0.3684 0.3834 0.12 0.2164
Breast 1 0.3042 0.1834 0.1736 0.0482 0.4466 0.4764 0.5612 0.1408 0.257 0.2798 0.1804 0.2978 0.2066
Colon 1 0.2126 0.5736 0.2986 0.6202 0.1544 0.4408 0.2098 0.466 0.4064 0.8402 0.428 0.1646
Heart 1 0.1486 0.0756 0.363 0.2052 0.3664 0.1852 0.334 0.204 0.1416 0.2534 0.1738
Kidney 1 0.241 0.2704 0.0754 0.1922 0.5326 0.2614 0.2024 0.3772 0.1292 0.1744
Liver 1 0.0568 0.0326 0.0644 0.6104 0.079 0.0624 0.0988 0.0698 0.0528
Lung 1 0.4138 0.8342 0.1582 0.455 0.6584 0.9384 0.2496 0.3324
LymphNode 1 0.3968 0.0382 0.4104 0.141 0.3552 0.387 0.111
Ovary 1 0.1126 0.571 0.4274 0.7982 0.2078 0.0454
Prostate 1 0.3898 0.1076 0.3416 0.1388 0.0838
SkeletalMuscle 1 0.318 0.1312 0.527 0.1254
Testes 1 0.6976 0.1454 0.1274
Thyroid 1 0.1958 0.1134
WhiteBloodCells 1 0.2518
EdomStromalCell 1
Placenta Adipose Adrenal Brain Breast Colon Heart Kidney Liver Lung LymphNode Ovary Prostate SkeletalMuscle Testes Thyroid WhiteBloodCells EdomStromalCell

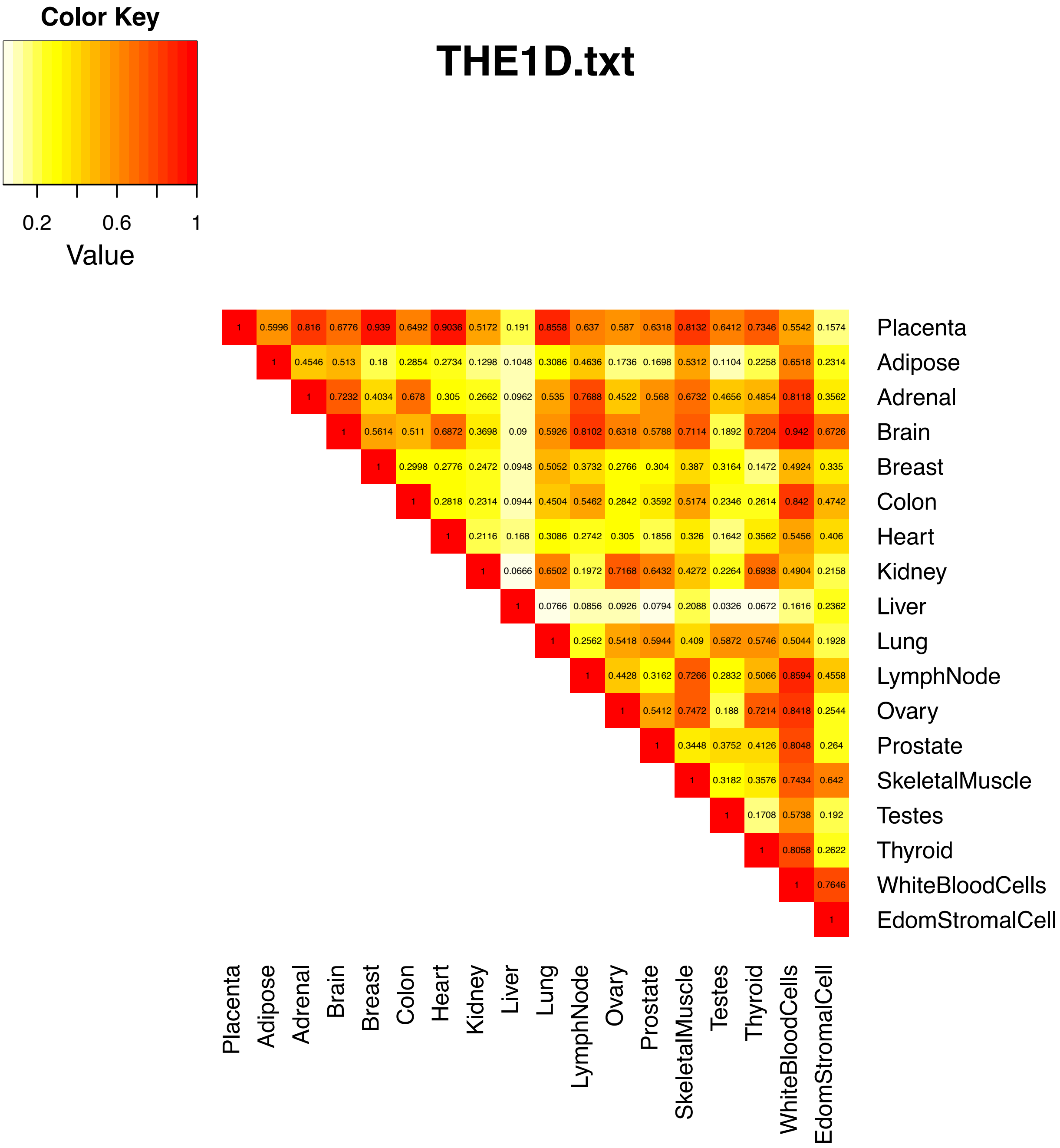
Color Key
0.2
0.6
1
Value
THE1D.txt
Placenta 1 0.5996 0.816 0.6776 0.939 0.6492 0.9036 0.5172 0.191 0.8558 0.637 0.587 0.6318 0.8132 0.6412 0.7346 0.5542 0.1574
Adipose 1 0.4546 0.513 0.18 0.2854 0.2734 0.1298 0.1048 0.3086 0.4636 0.1736 0.1698 0.5312 0.1104 0.2258 0.6518 0.2314
Adrenal 1 0.7232 0.4034 0.678 0.305 0.2662 0.0962 0.535 0.7688 0.4522 0.568 0.6732 0.4656 0.4854 0.8118 0.3562
Brain 1 0.5614 0.511 0.6872 0.3698 0.09 0.5926 0.8102 0.6318 0.5788 0.7114 0.1892 0.7204 0.942 0.6726
Breast 1 0.2998 0.2776 0.2472 0.0948 0.5052 0.3732 0.2766 0.304 0.387 0.3164 0.1472 0.4924 0.335
Colon 1 0.2818 0.2314 0.0944 0.4504 0.5462 0.2842 0.3592 0.5174 0.2346 0.2614 0.842 0.4742
Heart 1 0.2116 0.168 0.3086 0.2742 0.305 0.1856 0.326 0.1642 0.3562 0.5456 0.406
Kidney 1 0.0666 0.6502 0.1972 0.7168 0.6432 0.4272 0.2264 0.6938 0.4904 0.2158
Liver 1 0.0766 0.0856 0.0926 0.0794 0.2088 0.0326 0.0672 0.1616 0.2362
Lung 1 0.2562 0.5418 0.5944 0.409 0.5872 0.5746 0.5044 0.1928
LymphNode 1 0.4428 0.3162 0.7266 0.2832 0.5066 0.8594 0.4558
Ovary 1 0.5412 0.7472 0.188 0.7214 0.8418 0.2544
Prostate 1 0.3448 0.3752 0.4126 0.8048 0.264
SkeletalMuscle 1 0.3182 0.3576 0.7434 0.642
Testes 1 0.1708 0.5738 0.192
Thyroid 1 0.8058 0.2622
WhiteBloodCells 1 0.7646
EdomStromalCell 1
Placenta
Adipose
Adrenal
Brain
Breast
Colon
Heart
Kidney
Liver
Lung
LymphNode
Ovary
Prostate
SkeletalMuscle
Testes
Thyroid
WhiteBloodCells
EdomStromalCell
