# Supplementary material for: Detecting Endogenous Retrovirus-Driven Tissue-Specific Gene Transcription
Source: Genome Biol Evol. 2015 Mar 11;7(4):1082–97. doi: 10.1093/gbe/evv049 (PMC4419796; doi:10.1093/gbe/evv049)
Supplement: Supplementary Data [file supp_evv049_S2_TEPlots_Mouse.pdf]

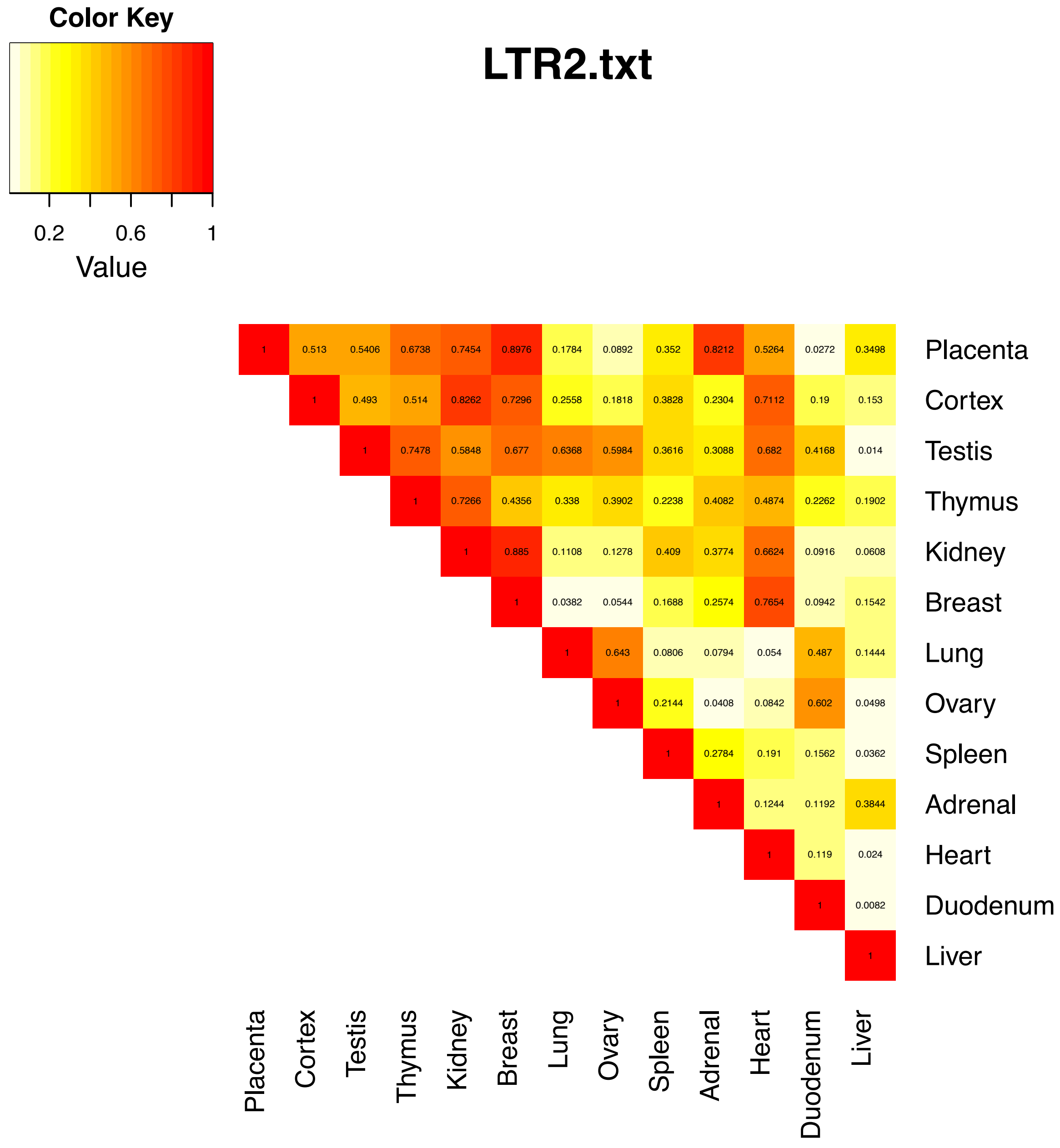
Color Key
0.2 0.6 1
Value
LTR2.txt
Placenta: 1 0.513 0.5406 0.6738 0.7454 0.8976 0.1784 0.0892 0.352 0.8212 0.5264 0.0272 0.3498
Cortex: 1 0.493 0.514 0.8262 0.7296 0.2558 0.1818 0.3828 0.2304 0.7112 0.19 0.153
Testis: 1 0.7478 0.5848 0.677 0.6368 0.5984 0.3616 0.3088 0.682 0.4168 0.014
Thymus: 1 0.7266 0.4356 0.338 0.3902 0.2238 0.4082 0.4874 0.2262 0.1902
Kidney: 1 0.885 0.1108 0.1278 0.409 0.3774 0.6624 0.0916 0.0608
Breast: 1 0.0382 0.0544 0.1688 0.2574 0.7654 0.0942 0.1542
Lung: 1 0.643 0.0806 0.0794 0.054 0.487 0.1444
Ovary: 1 0.2144 0.0408 0.0842 0.602 0.0498
Spleen: 1 0.2784 0.191 0.1562 0.0362
Adrenal: 1 0.1244 0.1192 0.3844
Heart: 1 0.119 0.024
Duodenum: 1 0.0082
Liver: 1
Placenta Cortex Testis Thymus Kidney Breast Lung Ovary Spleen Adrenal Heart Duodenum Liver

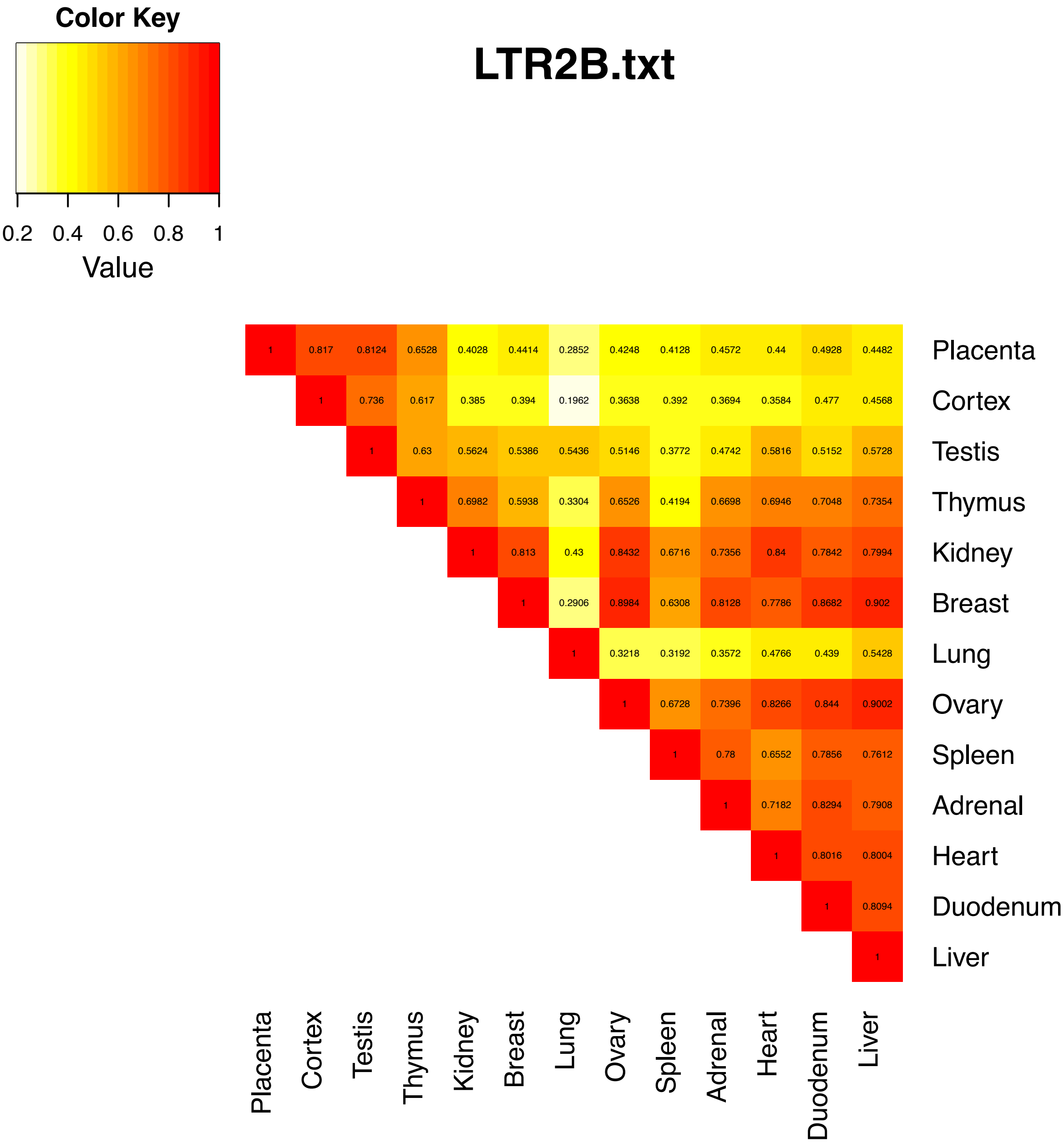
Color Key
0.2 0.4 0.6 0.8 1
Value
LTR2B.txt
Placenta: 1 0.817 0.8124 0.6528 0.4028 0.4414 0.2852 0.4248 0.4128 0.4572 0.44 0.4928 0.4482
Cortex: 1 0.736 0.617 0.385 0.394 0.1962 0.3638 0.392 0.3694 0.3584 0.477 0.4568
Testis: 1 0.63 0.5624 0.5386 0.5436 0.5146 0.3772 0.4742 0.5816 0.5152 0.5728
Thymus: 1 0.6982 0.5938 0.3304 0.6526 0.4194 0.6698 0.6946 0.7048 0.7354
Kidney: 1 0.813 0.43 0.8432 0.6716 0.7356 0.84 0.7842 0.7994
Breast: 1 0.2906 0.8984 0.6308 0.8128 0.7786 0.8682 0.902
Lung: 1 0.3218 0.3192 0.3572 0.4766 0.439 0.5428
Ovary: 1 0.6728 0.7396 0.8266 0.844 0.9002
Spleen: 1 0.78 0.6552 0.7856 0.7612
Adrenal: 1 0.7182 0.8294 0.7908
Heart: 1 0.8016 0.8004
Duodenum: 1 0.8094
Liver: 1
Placenta Cortex Testis Thymus Kidney Breast Lung Ovary Spleen Adrenal Heart Duodenum Liver

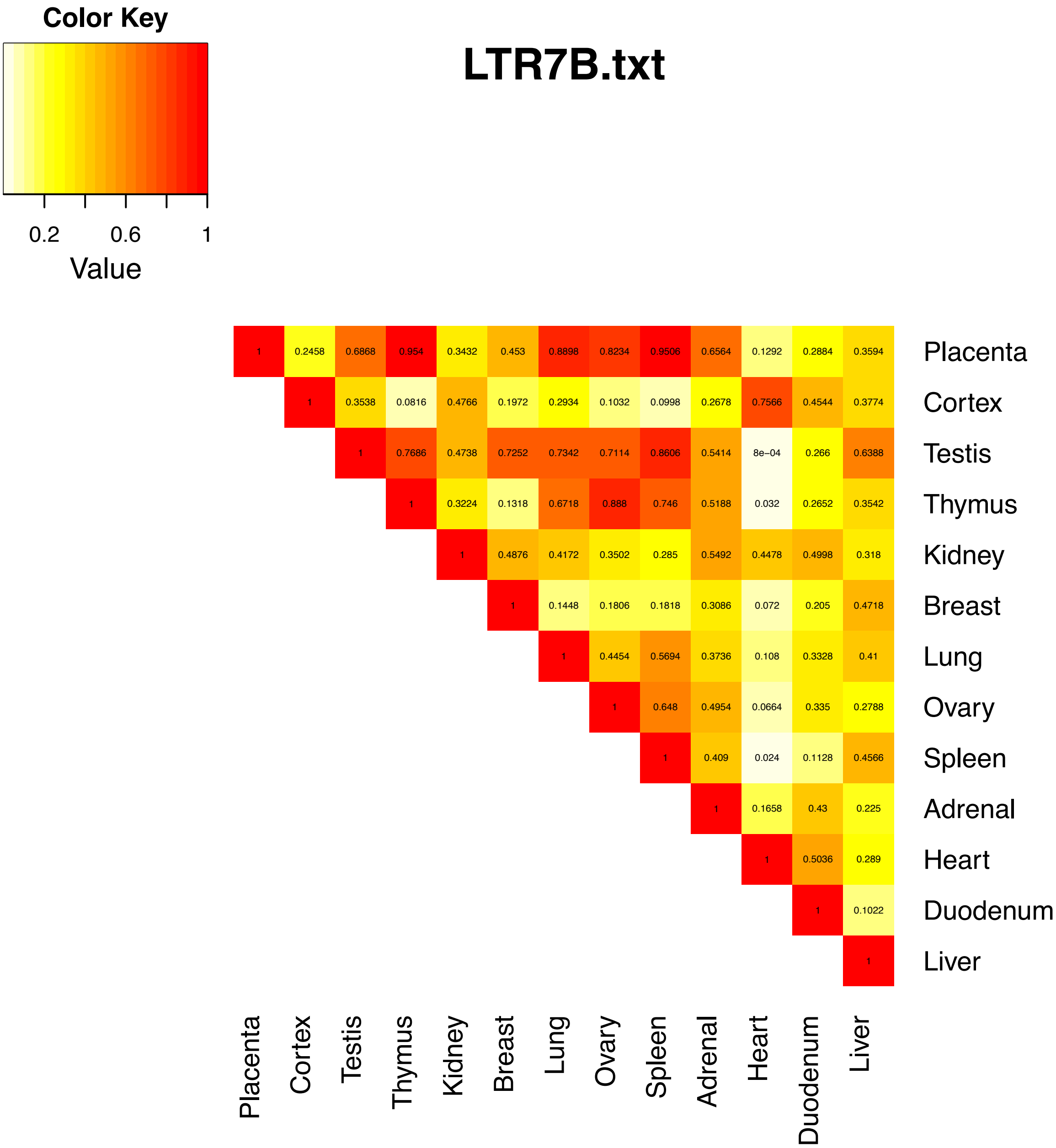
Color Key
0.2
0.6
1
Value
LTR7B.txt
1 0.2458 0.6868 0.954 0.3432 0.453 0.8898 0.8234 0.9506 0.6564 0.1292 0.2884 0.3594 Placenta
1 0.3538 0.0816 0.4766 0.1972 0.2934 0.1032 0.0998 0.2678 0.7566 0.4544 0.3774 Cortex
1 0.7686 0.4738 0.7252 0.7342 0.7114 0.8606 0.5414 8e−04 0.266 0.6388 Testis
1 0.3224 0.1318 0.6718 0.888 0.746 0.5188 0.032 0.2652 0.3542 Thymus
1 0.4876 0.4172 0.3502 0.285 0.5492 0.4478 0.4998 0.318 Kidney
1 0.1448 0.1806 0.1818 0.3086 0.072 0.205 0.4718 Breast
1 0.4454 0.5694 0.3736 0.108 0.3328 0.41 Lung
1 0.648 0.4954 0.0664 0.335 0.2788 Ovary
1 0.409 0.024 0.1128 0.4566 Spleen
1 0.1658 0.43 0.225 Adrenal
1 0.5036 0.289 Heart
1 0.1022 Duodenum
1 Liver
Placenta
Cortex
Testis
Thymus
Kidney
Breast
Lung
Ovary
Spleen
Adrenal
Heart
Duodenum
Liver

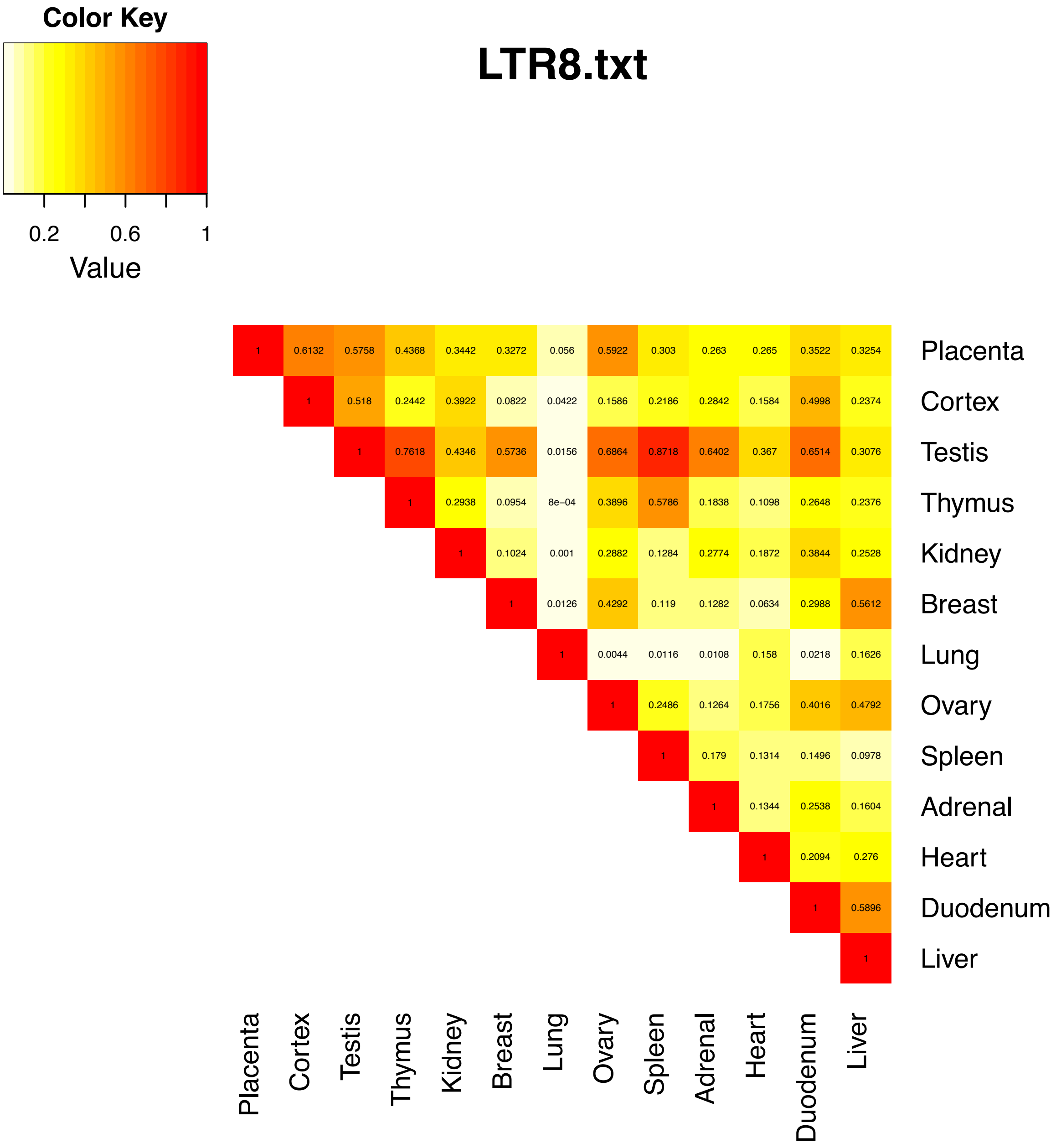
Color Key
0.2
0.6
1
Value
LTR8.txt
Placenta: 1 0.6132 0.5758 0.4368 0.3442 0.3272 0.056 0.5922 0.303 0.263 0.265 0.3522 0.3254
Cortex: 1 0.518 0.2442 0.3922 0.0822 0.0422 0.1586 0.2186 0.2842 0.1584 0.4998 0.2374
Testis: 1 0.7618 0.4346 0.5736 0.0156 0.6864 0.8718 0.6402 0.367 0.6514 0.3076
Thymus: 1 0.2938 0.0954 8e−04 0.3896 0.5786 0.1838 0.1098 0.2648 0.2376
Kidney: 1 0.1024 0.001 0.2882 0.1284 0.2774 0.1872 0.3844 0.2528
Breast: 1 0.0126 0.4292 0.119 0.1282 0.0634 0.2988 0.5612
Lung: 1 0.0044 0.0116 0.0108 0.158 0.0218 0.1626
Ovary: 1 0.2486 0.1264 0.1756 0.4016 0.4792
Spleen: 1 0.179 0.1314 0.1496 0.0978
Adrenal: 1 0.1344 0.2538 0.1604
Heart: 1 0.2094 0.276
Duodenum: 1 0.5896
Liver: 1
Placenta
Cortex
Testis
Thymus
Kidney
Breast
Lung
Ovary
Spleen
Adrenal
Heart
Duodenum
Liver

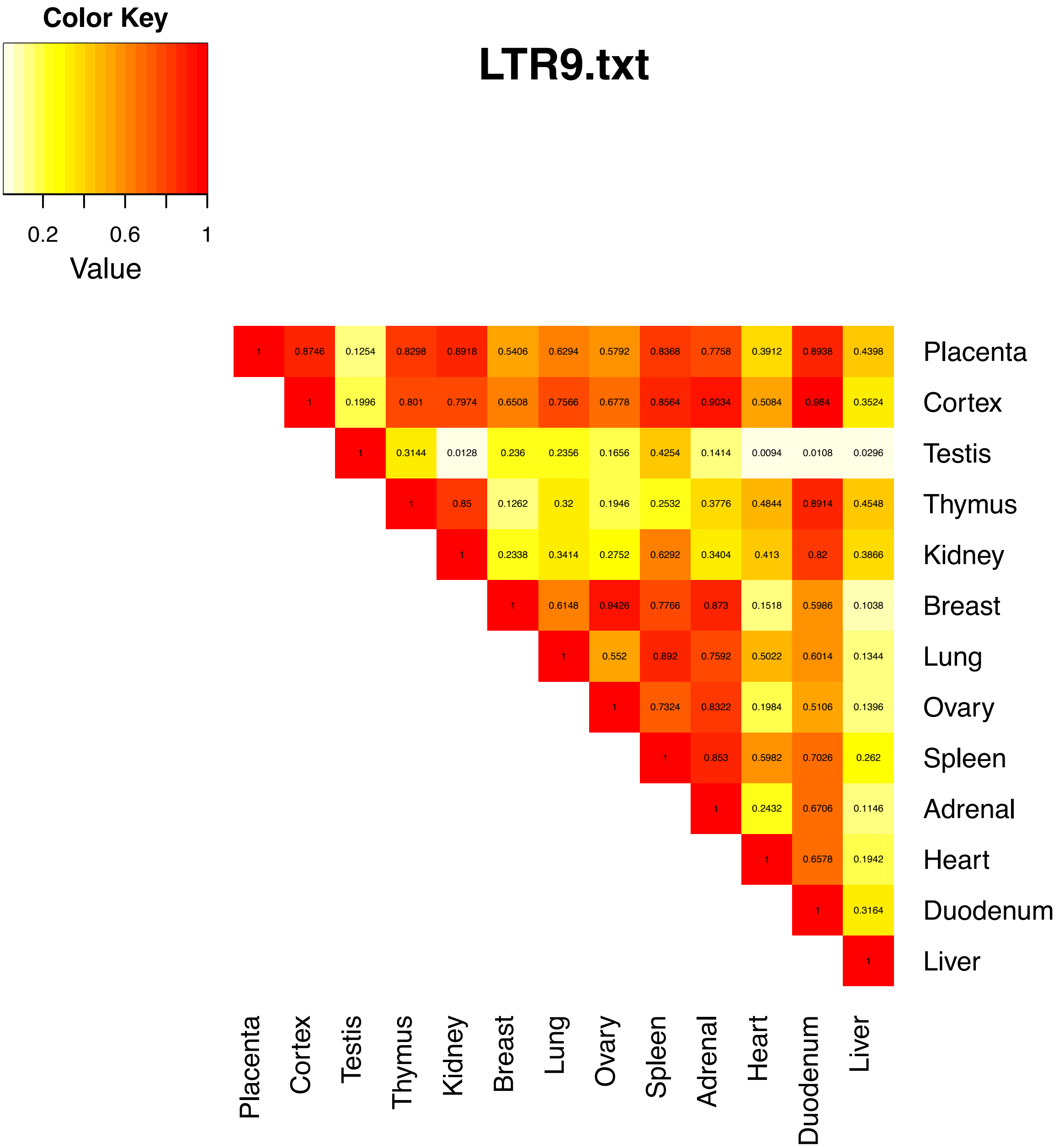
Color Key
0.2 0.6 1
Value
LTR9.txt
Placenta 1 0.8746 0.1254 0.8298 0.8918 0.5406 0.6294 0.5792 0.8368 0.7758 0.3912 0.8938 0.4398
Cortex 1 0.1996 0.801 0.7974 0.6508 0.7566 0.6778 0.8564 0.9034 0.5084 0.984 0.3524
Testis 1 0.3144 0.0128 0.236 0.2356 0.1656 0.4254 0.1414 0.0094 0.0108 0.0296
Thymus 1 0.85 0.1262 0.32 0.1946 0.2532 0.3776 0.4844 0.8914 0.4548
Kidney 1 0.2338 0.3414 0.2752 0.6292 0.3404 0.413 0.82 0.3866
Breast 1 0.6148 0.9426 0.7766 0.873 0.1518 0.5986 0.1038
Lung 1 0.552 0.892 0.7592 0.5022 0.6014 0.1344
Ovary 1 0.7324 0.8322 0.1984 0.5106 0.1396
Spleen 1 0.853 0.5982 0.7026 0.262
Adrenal 1 0.2432 0.6706 0.1146
Heart 1 0.6578 0.1942
Duodenum 1 0.3164
Liver 1
Placenta Cortex Testis Thymus Kidney Breast Lung Ovary Spleen Adrenal Heart Duodenum Liver

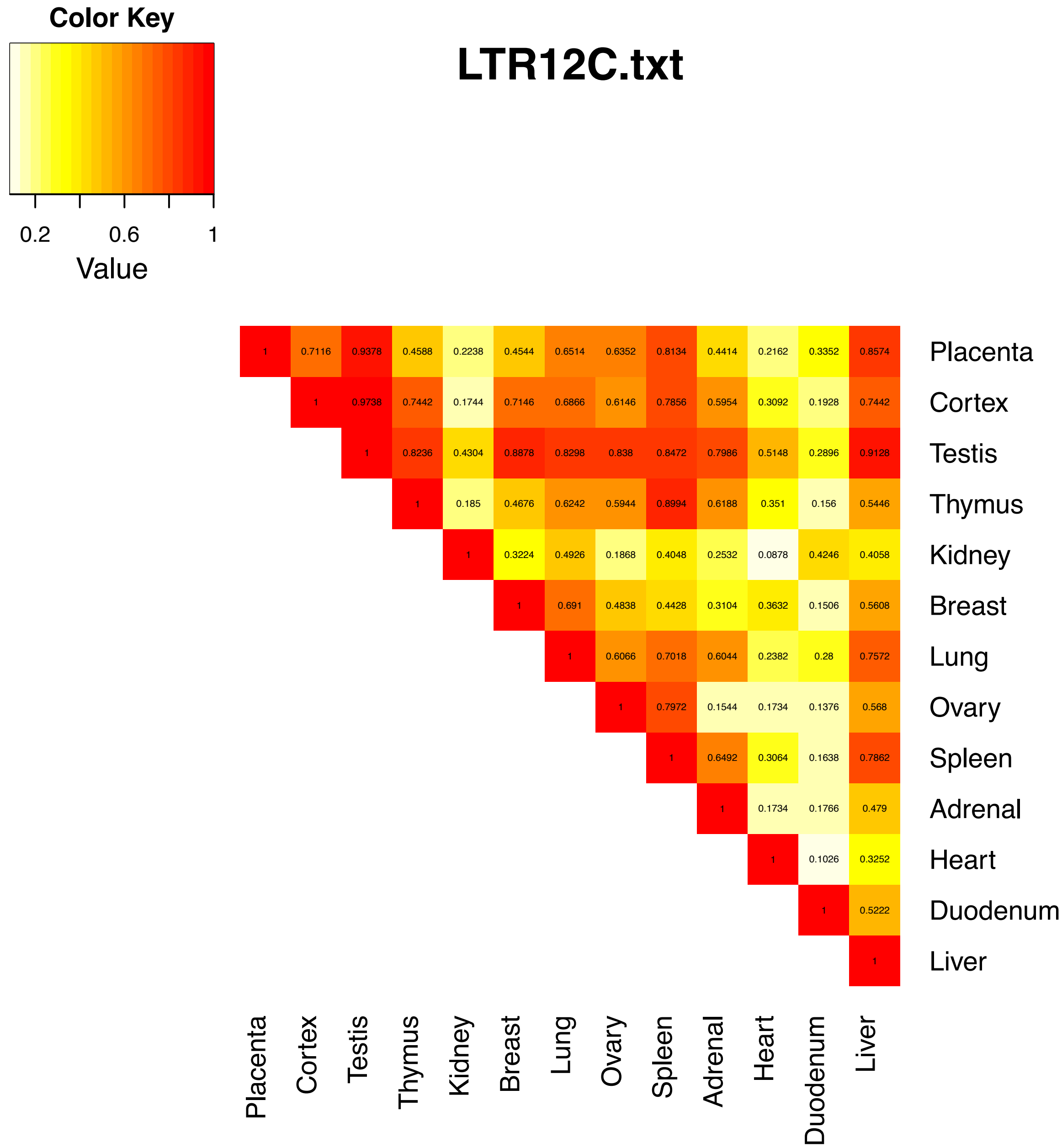
Color Key
0.2
0.6
1
Value
LTR12C.txt
Placenta
Cortex
Testis
Thymus
Kidney
Breast
Lung
Ovary
Spleen
Adrenal
Heart
Duodenum
Liver
1 0.7116 0.9378 0.4588 0.2238 0.4544 0.6514 0.6352 0.8134 0.4414 0.2162 0.3352 0.8574
1 0.9738 0.7442 0.1744 0.7146 0.6866 0.6146 0.7856 0.5954 0.3092 0.1928 0.7442
1 0.8236 0.4304 0.8878 0.8298 0.838 0.8472 0.7986 0.5148 0.2896 0.9128
1 0.185 0.4676 0.6242 0.5944 0.8994 0.6188 0.351 0.156 0.5446
1 0.3224 0.4926 0.1868 0.4048 0.2532 0.0878 0.4246 0.4058
1 0.691 0.4838 0.4428 0.3104 0.3632 0.1506 0.5608
1 0.6066 0.7018 0.6044 0.2382 0.28 0.7572
1 0.7972 0.1544 0.1734 0.1376 0.568
1 0.6492 0.3064 0.1638 0.7862
1 0.1734 0.1766 0.479
1 0.1026 0.3252
1 0.5222
1

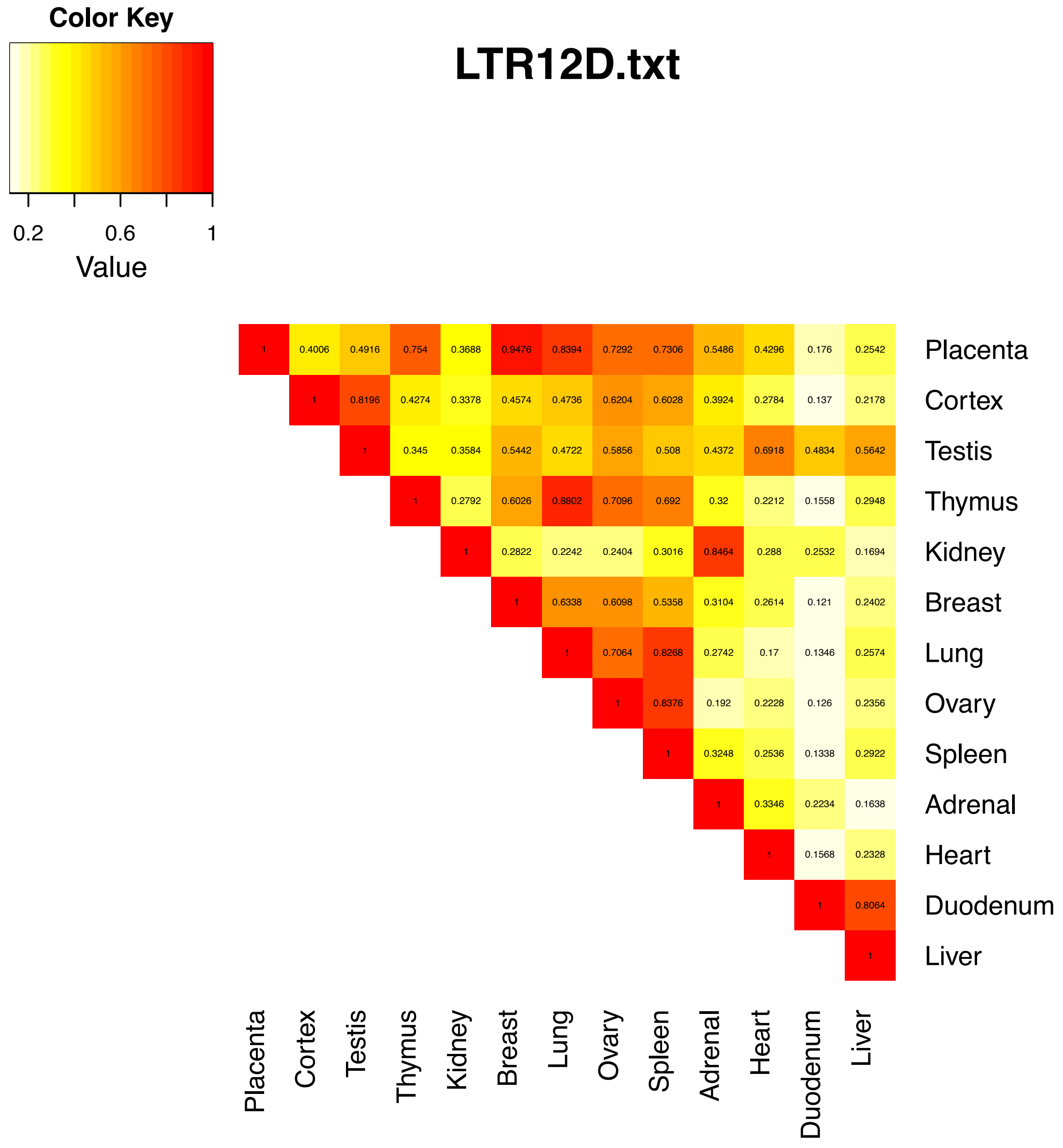
Color Key
0.2
0.6
1
Value
LTR12D.txt
Placenta 1 0.4006 0.4916 0.754 0.3688 0.9476 0.8394 0.7292 0.7306 0.5486 0.4296 0.176 0.2542
Cortex 1 0.8196 0.4274 0.3378 0.4574 0.4736 0.6204 0.6028 0.3924 0.2784 0.137 0.2178
Testis 1 0.345 0.3584 0.5442 0.4722 0.5856 0.508 0.4372 0.6918 0.4834 0.5642
Thymus 1 0.2792 0.6026 0.8802 0.7096 0.692 0.32 0.2212 0.1558 0.2948
Kidney 1 0.2822 0.2242 0.2404 0.3016 0.8464 0.288 0.2532 0.1694
Breast 1 0.6338 0.6098 0.5358 0.3104 0.2614 0.121 0.2402
Lung 1 0.7064 0.8268 0.2742 0.17 0.1346 0.2574
Ovary 1 0.8376 0.192 0.2228 0.126 0.2356
Spleen 1 0.3248 0.2536 0.1338 0.2922
Adrenal 1 0.3346 0.2234 0.1638
Heart 1 0.1568 0.2328
Duodenum 1 0.8064
Liver 1
Placenta
Cortex
Testis
Thymus
Kidney
Breast
Lung
Ovary
Spleen
Adrenal
Heart
Duodenum
Liver

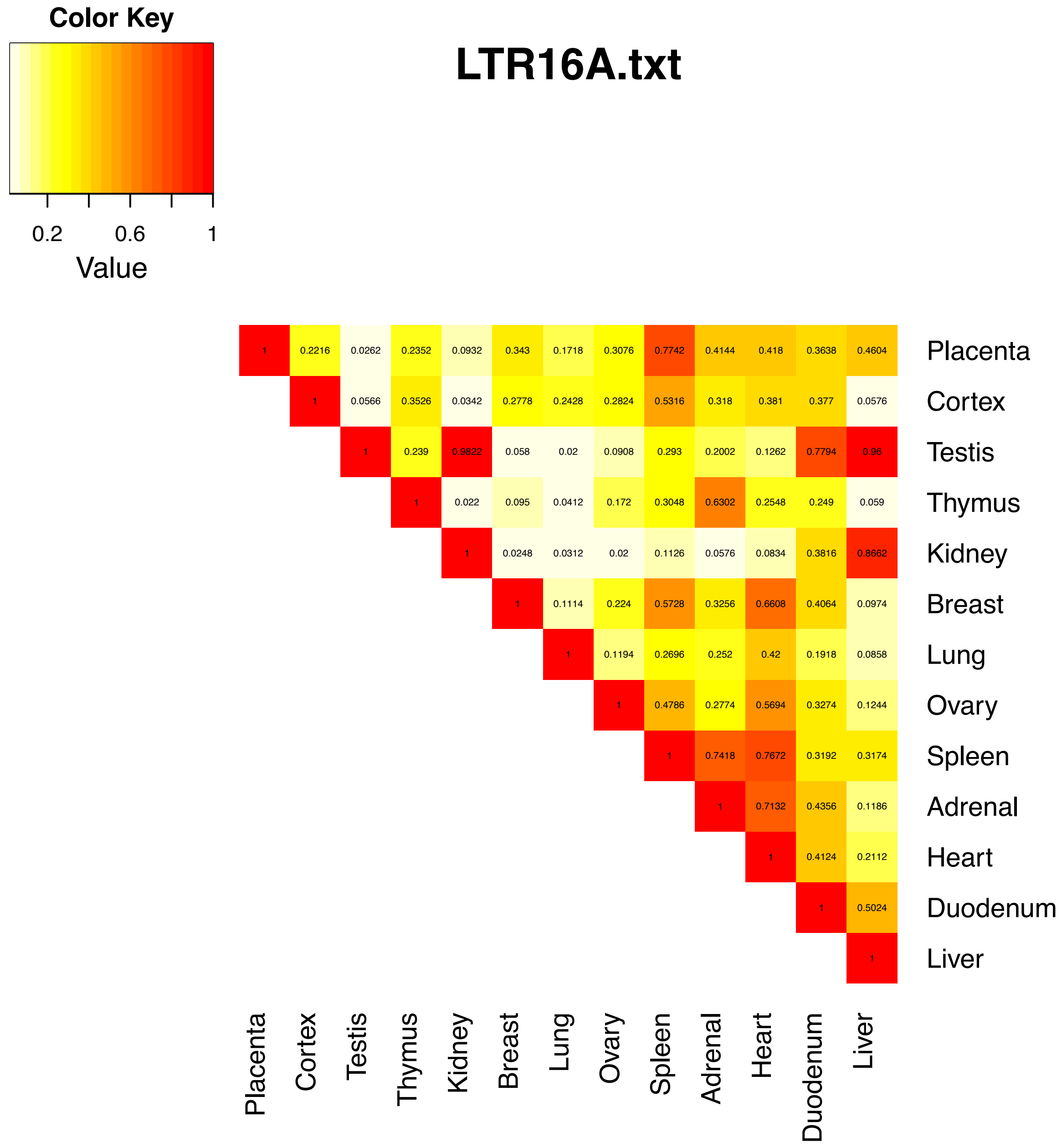
Color Key
0.2
0.6
1
Value
LTR16A.txt
Placenta
Cortex
Testis
Thymus
Kidney
Breast
Lung
Ovary
Spleen
Adrenal
Heart
Duodenum
Liver
1 0.2216 0.0262 0.2352 0.0932 0.343 0.1718 0.3076 0.7742 0.4144 0.418 0.3638 0.4604
1 0.0566 0.3526 0.0342 0.2778 0.2428 0.2824 0.5316 0.318 0.381 0.377 0.0576
1 0.239 0.9822 0.058 0.02 0.0908 0.293 0.2002 0.1262 0.7794 0.96
1 0.022 0.095 0.0412 0.172 0.3048 0.6302 0.2548 0.249 0.059
1 0.0248 0.0312 0.02 0.1126 0.0576 0.0834 0.3816 0.8662
1 0.1114 0.224 0.5728 0.3256 0.6608 0.4064 0.0974
1 0.1194 0.2696 0.252 0.42 0.1918 0.0858
1 0.4786 0.2774 0.5694 0.3274 0.1244
1 0.7418 0.7672 0.3192 0.3174
1 0.7132 0.4356 0.1186
1 0.4124 0.2112
1 0.5024
1

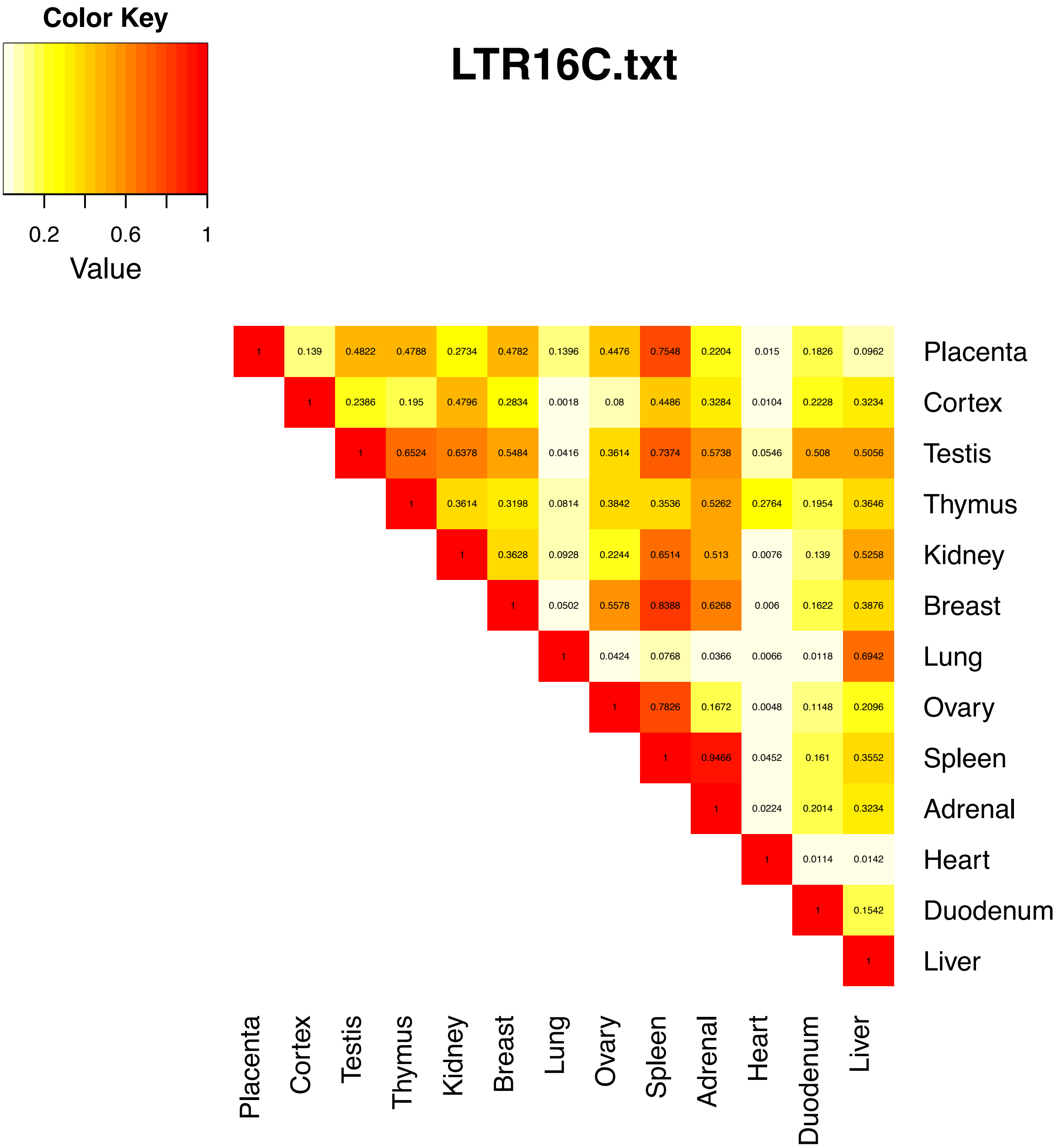

Color Key
0.2
0.6
1
Value
LTR16C.txt
Placenta
Cortex
Testis
Thymus
Kidney
Breast
Lung
Ovary
Spleen
Adrenal
Heart
Duodenum
Liver
1 0.139 0.4822 0.4788 0.2734 0.4782 0.1396 0.4476 0.7548 0.2204 0.015 0.1826 0.0962
1 0.2386 0.195 0.4796 0.2834 0.0018 0.08 0.4486 0.3284 0.0104 0.2228 0.3234
1 0.6524 0.6378 0.5484 0.0416 0.3614 0.7374 0.5738 0.0546 0.508 0.5056
1 0.3614 0.3198 0.0814 0.3842 0.3536 0.5262 0.2764 0.1954 0.3646
1 0.3628 0.0928 0.2244 0.6514 0.513 0.0076 0.139 0.5258
1 0.0502 0.5578 0.8388 0.6268 0.006 0.1622 0.3876
1 0.0424 0.0768 0.0366 0.0066 0.0118 0.6942
1 0.7826 0.1672 0.0048 0.1148 0.2096
1 0.9466 0.0452 0.161 0.3552
1 0.0224 0.2014 0.3234
1 0.0114 0.0142
1 0.1542
1

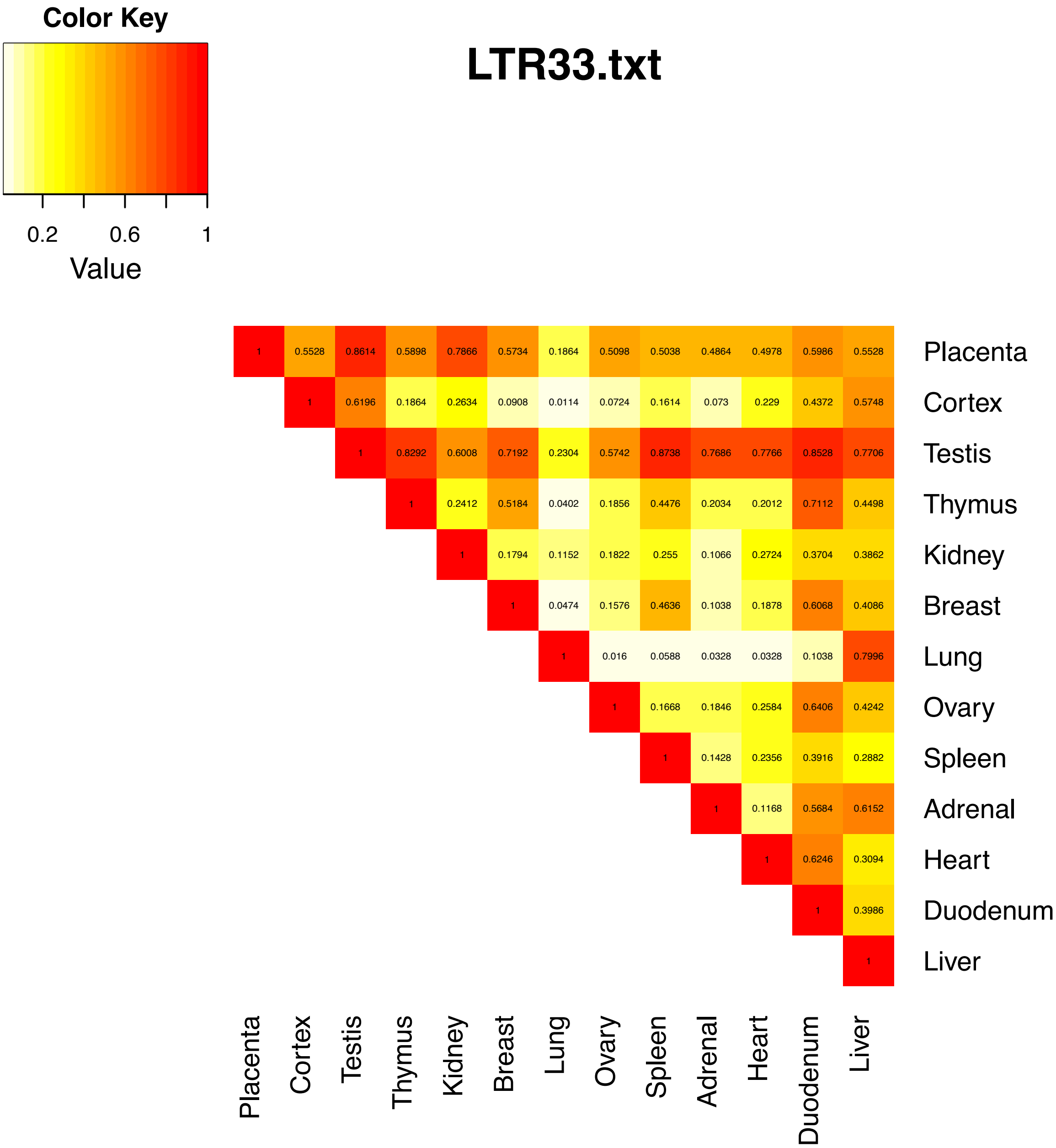
Color Key
0.2
0.6
1
Value
LTR33.txt
Placenta: 1 0.5528 0.8614 0.5898 0.7866 0.5734 0.1864 0.5098 0.5038 0.4864 0.4978 0.5986 0.5528
Cortex: 1 0.6196 0.1864 0.2634 0.0908 0.0114 0.0724 0.1614 0.073 0.229 0.4372 0.5748
Testis: 1 0.8292 0.6008 0.7192 0.2304 0.5742 0.8738 0.7686 0.7766 0.8528 0.7706
Thymus: 1 0.2412 0.5184 0.0402 0.1856 0.4476 0.2034 0.2012 0.7112 0.4498
Kidney: 1 0.1794 0.1152 0.1822 0.255 0.1066 0.2724 0.3704 0.3862
Breast: 1 0.0474 0.1576 0.4636 0.1038 0.1878 0.6068 0.4086
Lung: 1 0.016 0.0588 0.0328 0.0328 0.1038 0.7996
Ovary: 1 0.1668 0.1846 0.2584 0.6406 0.4242
Spleen: 1 0.1428 0.2356 0.3916 0.2882
Adrenal: 1 0.1168 0.5684 0.6152
Heart: 1 0.6246 0.3094
Duodenum: 1 0.3986
Liver: 1
Placenta
Cortex
Testis
Thymus
Kidney
Breast
Lung
Ovary
Spleen
Adrenal
Heart
Duodenum
Liver

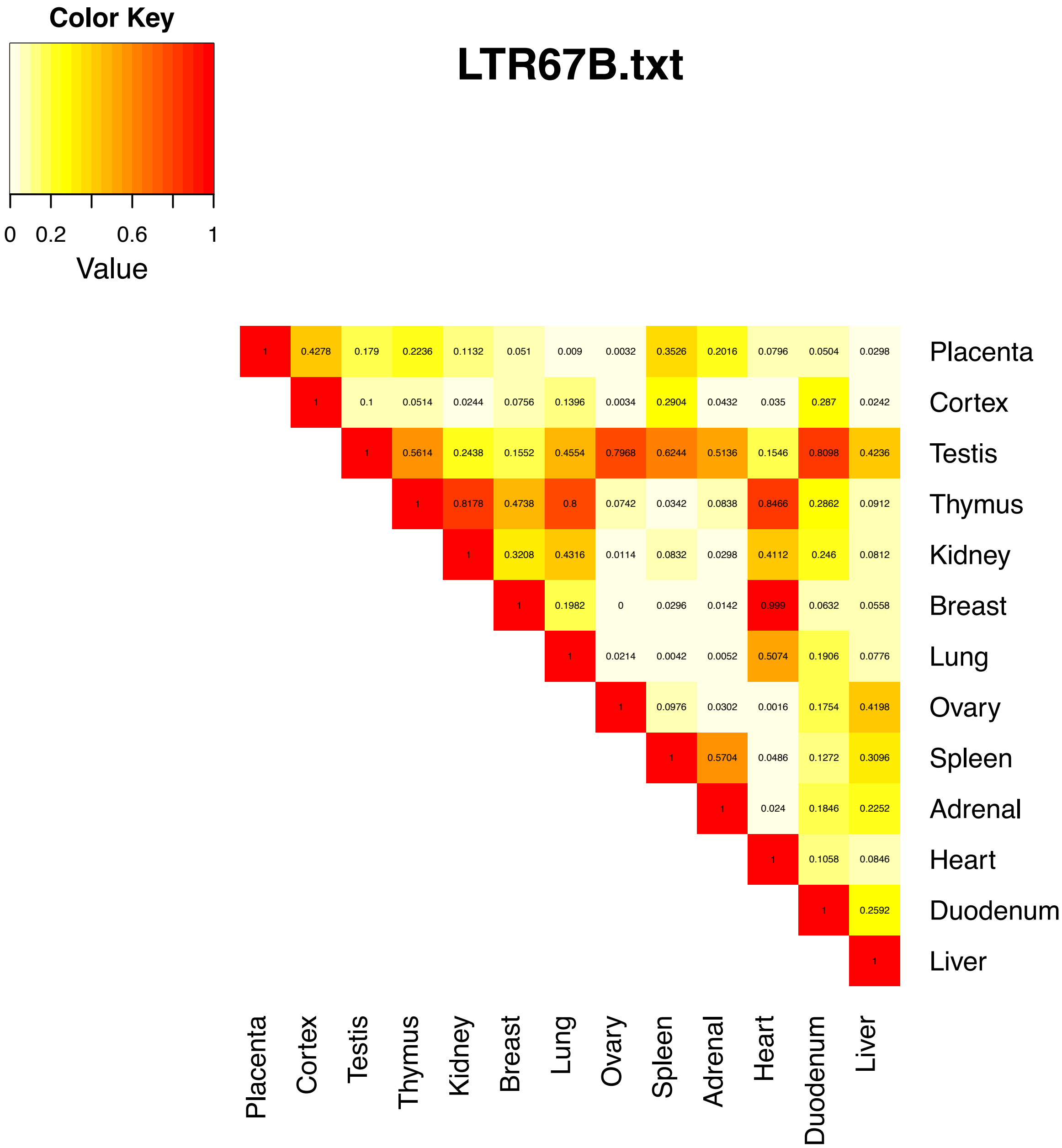
Color Key
0 0.2 0.6 1
Value
LTR67B.txt
Placenta Cortex Testis Thymus Kidney Breast Lung Ovary Spleen Adrenal Heart Duodenum Liver
1 0.4278 0.179 0.2236 0.1132 0.051 0.009 0.0032 0.3526 0.2016 0.0796 0.0504 0.0298
1 0.1 0.0514 0.0244 0.0756 0.1396 0.0034 0.2904 0.0432 0.035 0.287 0.0242
1 0.5614 0.2438 0.1552 0.4554 0.7968 0.6244 0.5136 0.1546 0.8098 0.4236
1 0.8178 0.4738 0.8 0.0742 0.0342 0.0838 0.8466 0.2862 0.0912
1 0.3208 0.4316 0.0114 0.0832 0.0298 0.4112 0.246 0.0812
1 0.1982 0 0.0296 0.0142 0.999 0.0632 0.0558
1 0.0214 0.0042 0.0052 0.5074 0.1906 0.0776
1 0.0976 0.0302 0.0016 0.1754 0.4198
1 0.5704 0.0486 0.1272 0.3096
1 0.024 0.1846 0.2252
1 0.1058 0.0846
1 0.2592
1

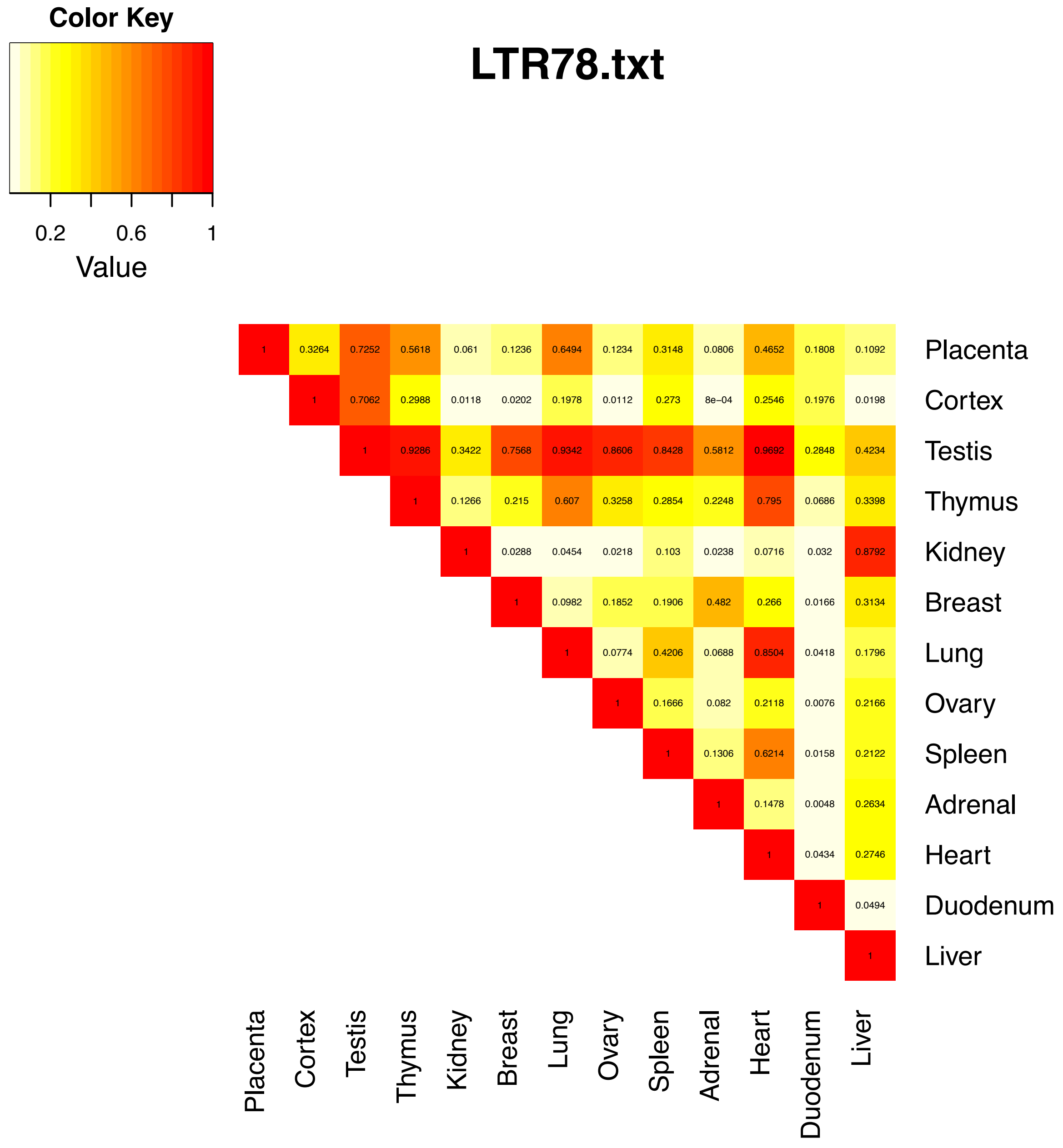
LTR78.txt
Color Key
0.2
0.6
1
Value
1 0.3264 0.7252 0.5618 0.061 0.1236 0.6494 0.1234 0.3148 0.0806 0.4652 0.1808 0.1092 Placenta
1 0.7062 0.2988 0.0118 0.0202 0.1978 0.0112 0.273 8e−04 0.2546 0.1976 0.0198 Cortex
1 0.9286 0.3422 0.7568 0.9342 0.8606 0.8428 0.5812 0.9692 0.2848 0.4234 Testis
1 0.1266 0.215 0.607 0.3258 0.2854 0.2248 0.795 0.0686 0.3398 Thymus
1 0.0288 0.0454 0.0218 0.103 0.0238 0.0716 0.032 0.8792 Kidney
1 0.0982 0.1852 0.1906 0.482 0.266 0.0166 0.3134 Breast
1 0.0774 0.4206 0.0688 0.8504 0.0418 0.1796 Lung
1 0.1666 0.082 0.2118 0.0076 0.2166 Ovary
1 0.1306 0.6214 0.0158 0.2122 Spleen
1 0.1478 0.0048 0.2634 Adrenal
1 0.0434 0.2746 Heart
1 0.0494 Duodenum
1 Liver
Placenta
Cortex
Testis
Thymus
Kidney
Breast
Lung
Ovary
Spleen
Adrenal
Heart
Duodenum
Liver

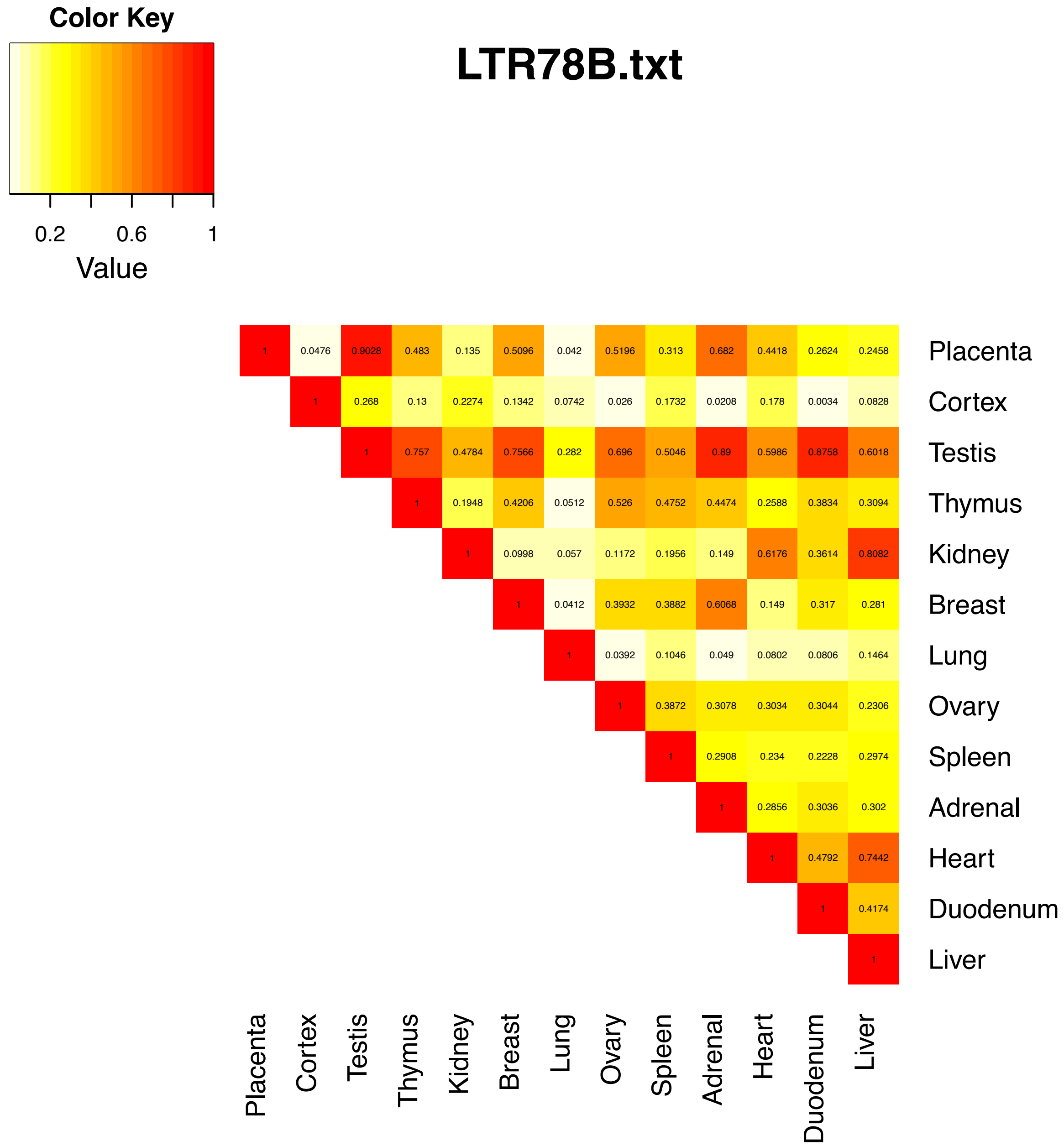
Color Key
0.2
0.6
1
Value
LTR78B.txt
Placenta
Cortex
Testis
Thymus
Kidney
Breast
Lung
Ovary
Spleen
Adrenal
Heart
Duodenum
Liver
1 0.0476 0.9028 0.483 0.135 0.5096 0.042 0.5196 0.313 0.682 0.4418 0.2624 0.2458
1 0.268 0.13 0.2274 0.1342 0.0742 0.026 0.1732 0.0208 0.178 0.0034 0.0828
1 0.757 0.4784 0.7566 0.282 0.696 0.5046 0.89 0.5986 0.8758 0.6018
1 0.1948 0.4206 0.0512 0.526 0.4752 0.4474 0.2588 0.3834 0.3094
1 0.0998 0.057 0.1172 0.1956 0.149 0.6176 0.3614 0.8082
1 0.0412 0.3932 0.3882 0.6068 0.149 0.317 0.281
1 0.0392 0.1046 0.049 0.0802 0.0806 0.1464
1 0.3872 0.3078 0.3034 0.3044 0.2306
1 0.2908 0.234 0.2228 0.2974
1 0.2856 0.3036 0.302
1 0.4792 0.7442
1 0.4174
1

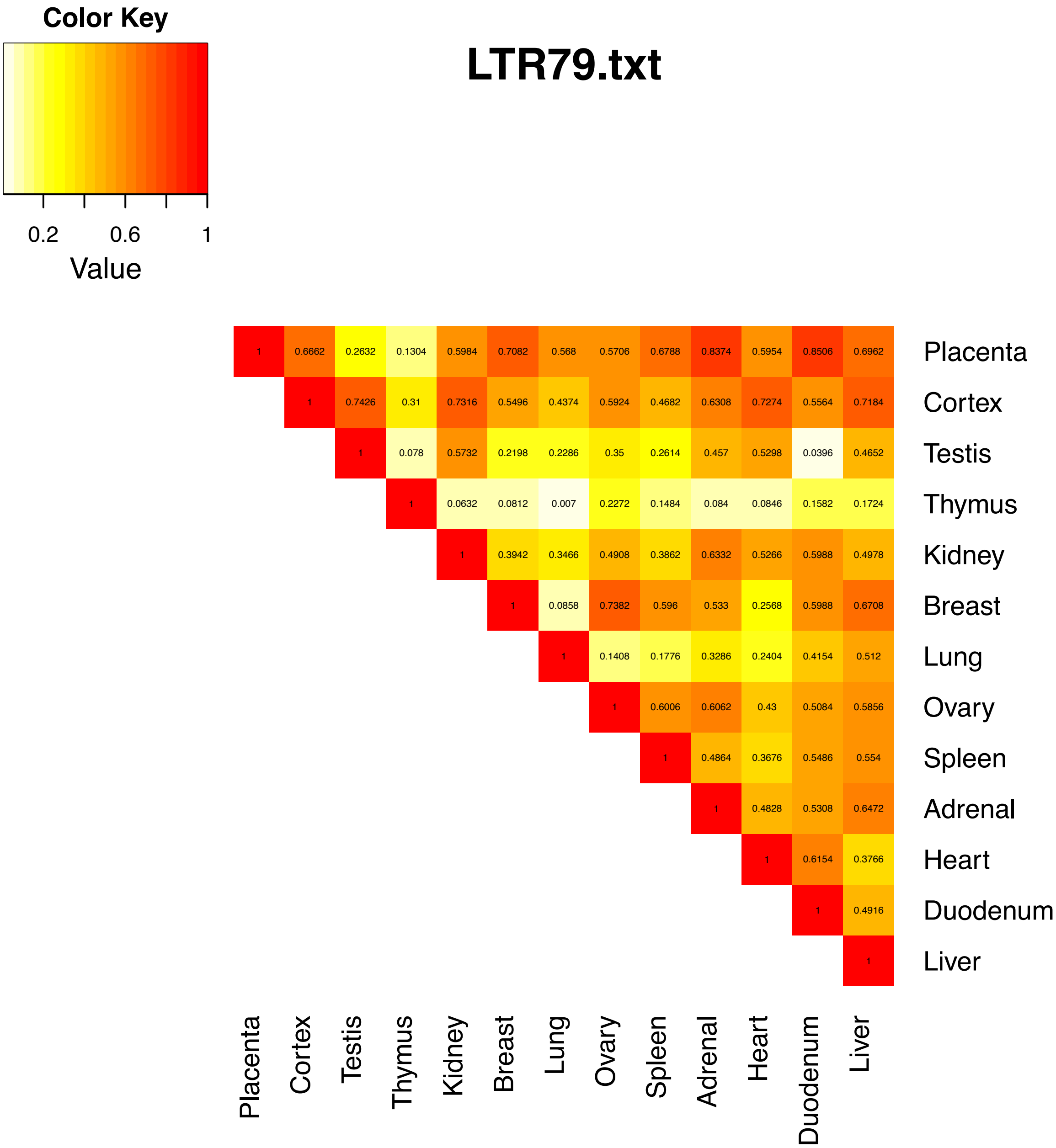
Color Key
0.2
0.6
1
Value
LTR79.txt
Placenta: 1 0.6662 0.2632 0.1304 0.5984 0.7082 0.568 0.5706 0.6788 0.8374 0.5954 0.8506 0.6962
Cortex: 1 0.7426 0.31 0.7316 0.5496 0.4374 0.5924 0.4682 0.6308 0.7274 0.5564 0.7184
Testis: 1 0.078 0.5732 0.2198 0.2286 0.35 0.2614 0.457 0.5298 0.0396 0.4652
Thymus: 1 0.0632 0.0812 0.007 0.2272 0.1484 0.084 0.0846 0.1582 0.1724
Kidney: 1 0.3942 0.3466 0.4908 0.3862 0.6332 0.5266 0.5988 0.4978
Breast: 1 0.0858 0.7382 0.596 0.533 0.2568 0.5988 0.6708
Lung: 1 0.1408 0.1776 0.3286 0.2404 0.4154 0.512
Ovary: 1 0.6006 0.6062 0.43 0.5084 0.5856
Spleen: 1 0.4864 0.3676 0.5486 0.554
Adrenal: 1 0.4828 0.5308 0.6472
Heart: 1 0.6154 0.3766
Duodenum: 1 0.4916
Liver: 1
Placenta
Cortex
Testis
Thymus
Kidney
Breast
Lung
Ovary
Spleen
Adrenal
Heart
Duodenum
Liver

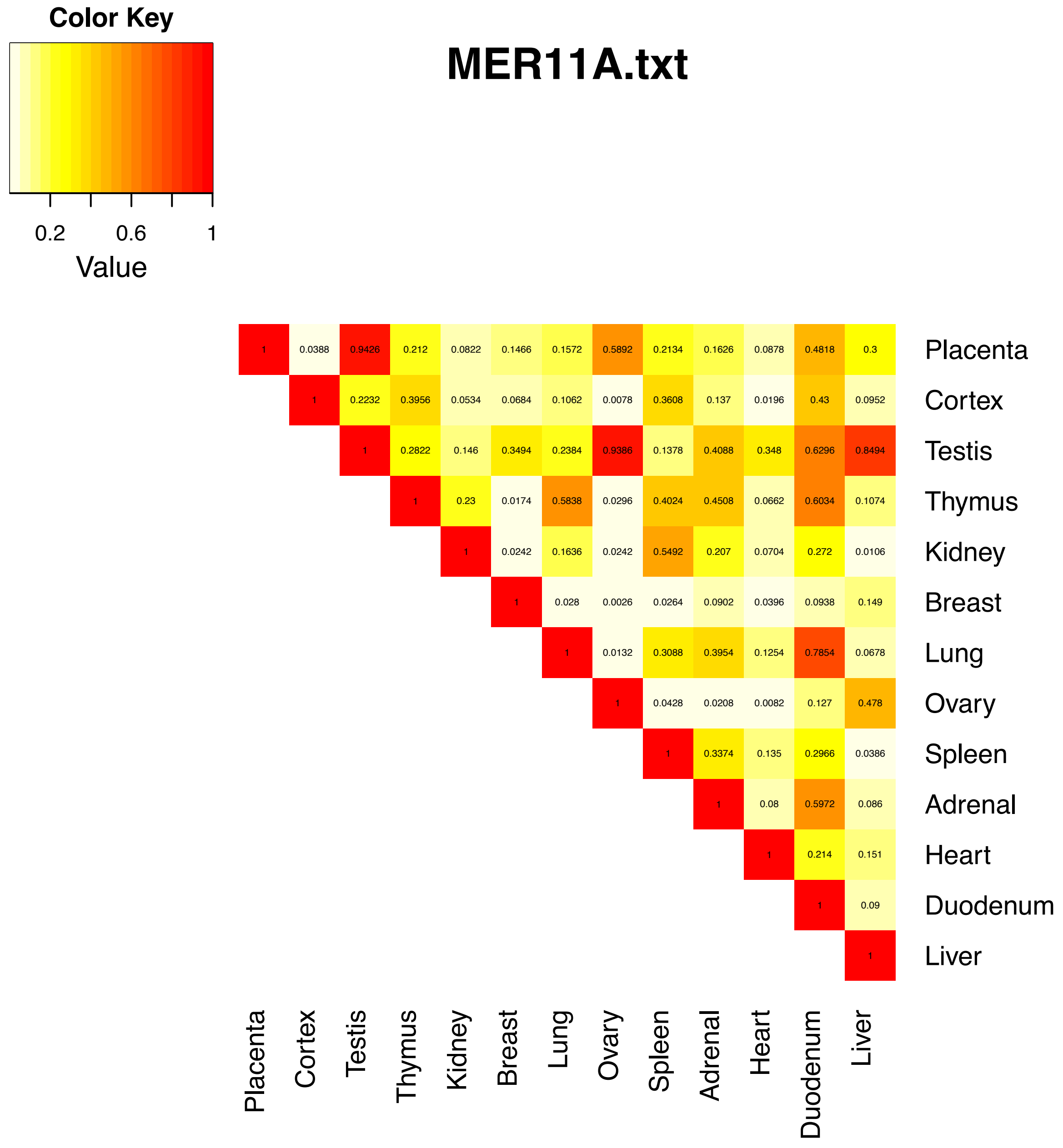
Color Key
0.2
0.6
1
Value
MER11A.txt
Placenta
Cortex
Testis
Thymus
Kidney
Breast
Lung
Ovary
Spleen
Adrenal
Heart
Duodenum
Liver
1 0.0388 0.9426 0.212 0.0822 0.1466 0.1572 0.5892 0.2134 0.1626 0.0878 0.4818 0.3
1 0.2232 0.3956 0.0534 0.0684 0.1062 0.0078 0.3608 0.137 0.0196 0.43 0.0952
1 0.2822 0.146 0.3494 0.2384 0.9386 0.1378 0.4088 0.348 0.6296 0.8494
1 0.23 0.0174 0.5838 0.0296 0.4024 0.4508 0.0662 0.6034 0.1074
1 0.0242 0.1636 0.0242 0.5492 0.207 0.0704 0.272 0.0106
1 0.028 0.0026 0.0264 0.0902 0.0396 0.0938 0.149
1 0.0132 0.3088 0.3954 0.1254 0.7854 0.0678
1 0.0428 0.0208 0.0082 0.127 0.478
1 0.3374 0.135 0.2966 0.0386
1 0.08 0.5972 0.086
1 0.214 0.151
1 0.09
1

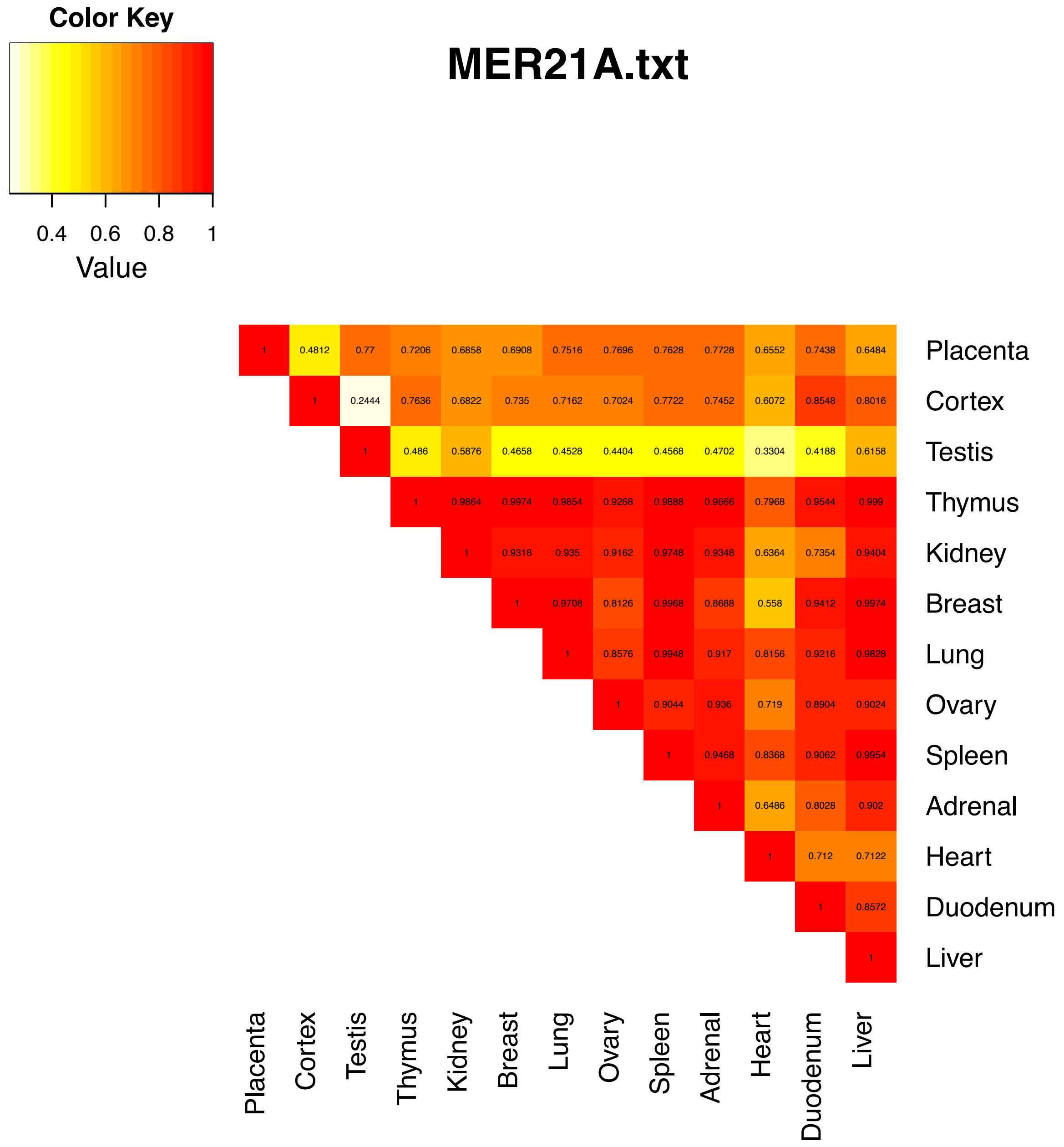
Color Key
0.4 0.6 0.8 1
Value
MER21A.txt
Placenta: 1 0.4812 0.77 0.7206 0.6858 0.6908 0.7516 0.7696 0.7628 0.7728 0.6552 0.7438 0.6484
Cortex: 1 0.2444 0.7636 0.6822 0.735 0.7162 0.7024 0.7722 0.7452 0.6072 0.8548 0.8016
Testis: 1 0.486 0.5876 0.4658 0.4528 0.4404 0.4568 0.4702 0.3304 0.4188 0.6158
Thymus: 1 0.9864 0.9974 0.9854 0.9268 0.9888 0.9666 0.7968 0.9544 0.999
Kidney: 1 0.9318 0.935 0.9162 0.9748 0.9348 0.6364 0.7354 0.9404
Breast: 1 0.9708 0.8126 0.9968 0.8688 0.558 0.9412 0.9974
Lung: 1 0.8576 0.9948 0.917 0.8156 0.9216 0.9828
Ovary: 1 0.9044 0.936 0.719 0.8904 0.9024
Spleen: 1 0.9468 0.8368 0.9062 0.9954
Adrenal: 1 0.6486 0.8028 0.902
Heart: 1 0.712 0.7122
Duodenum: 1 0.8572
Liver: 1
Placenta Cortex Testis Thymus Kidney Breast Lung Ovary Spleen Adrenal Heart Duodenum Liver

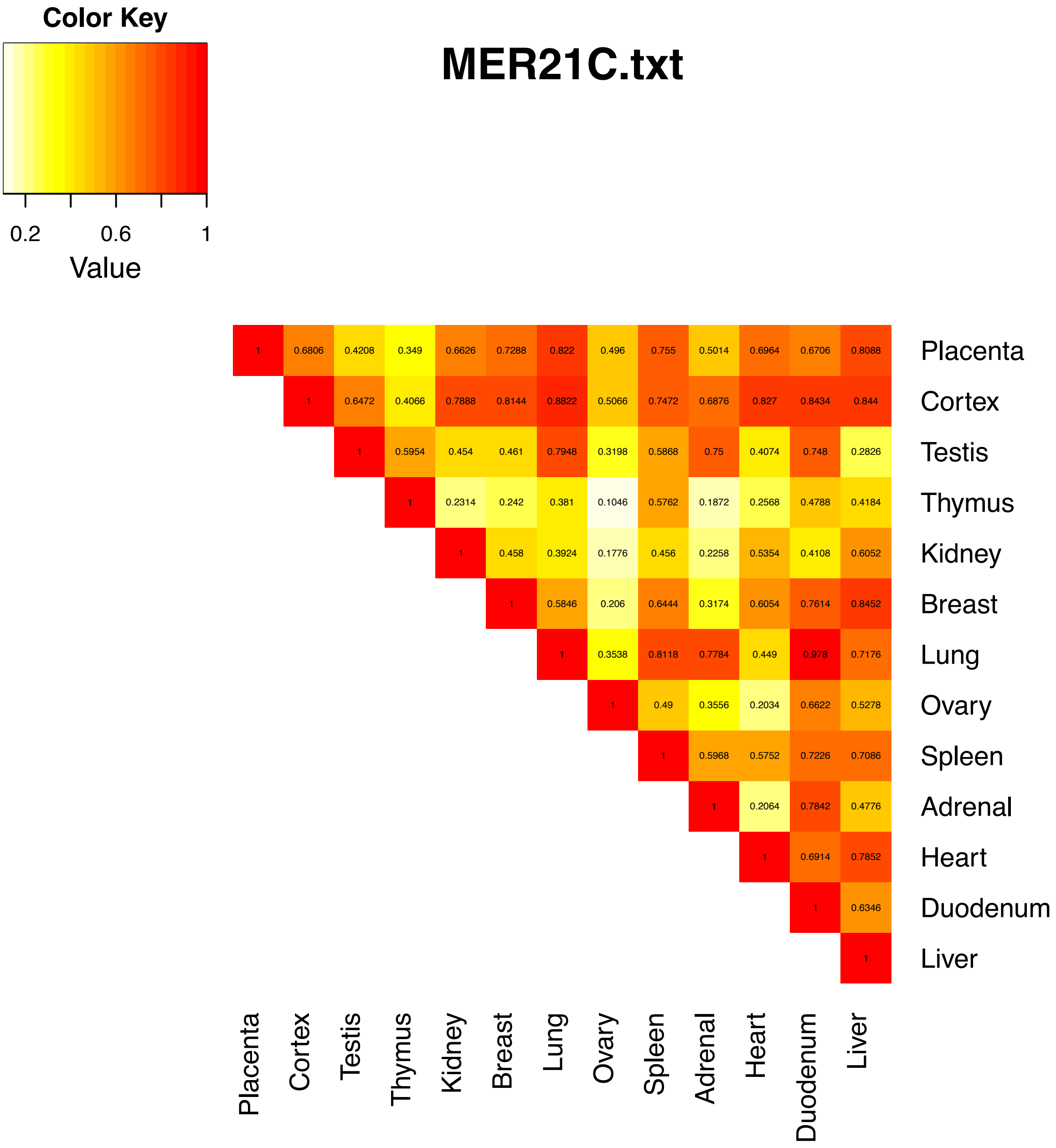
Color Key
0.2
0.6
1
Value
MER21C.txt
Placenta 1 0.6806 0.4208 0.349 0.6626 0.7288 0.822 0.496 0.755 0.5014 0.6964 0.6706 0.8088
Cortex 1 0.6472 0.4066 0.7888 0.8144 0.8822 0.5066 0.7472 0.6876 0.827 0.8434 0.844
Testis 1 0.5954 0.454 0.461 0.7948 0.3198 0.5868 0.75 0.4074 0.748 0.2826
Thymus 1 0.2314 0.242 0.381 0.1046 0.5762 0.1872 0.2568 0.4788 0.4184
Kidney 1 0.458 0.3924 0.1776 0.456 0.2258 0.5354 0.4108 0.6052
Breast 1 0.5846 0.206 0.6444 0.3174 0.6054 0.7614 0.8452
Lung 1 0.3538 0.8118 0.7784 0.449 0.978 0.7176
Ovary 1 0.49 0.3556 0.2034 0.6622 0.5278
Spleen 1 0.5968 0.5752 0.7226 0.7086
Adrenal 1 0.2064 0.7842 0.4776
Heart 1 0.6914 0.7852
Duodenum 1 0.6346
Liver 1
Placenta
Cortex
Testis
Thymus
Kidney
Breast
Lung
Ovary
Spleen
Adrenal
Heart
Duodenum
Liver

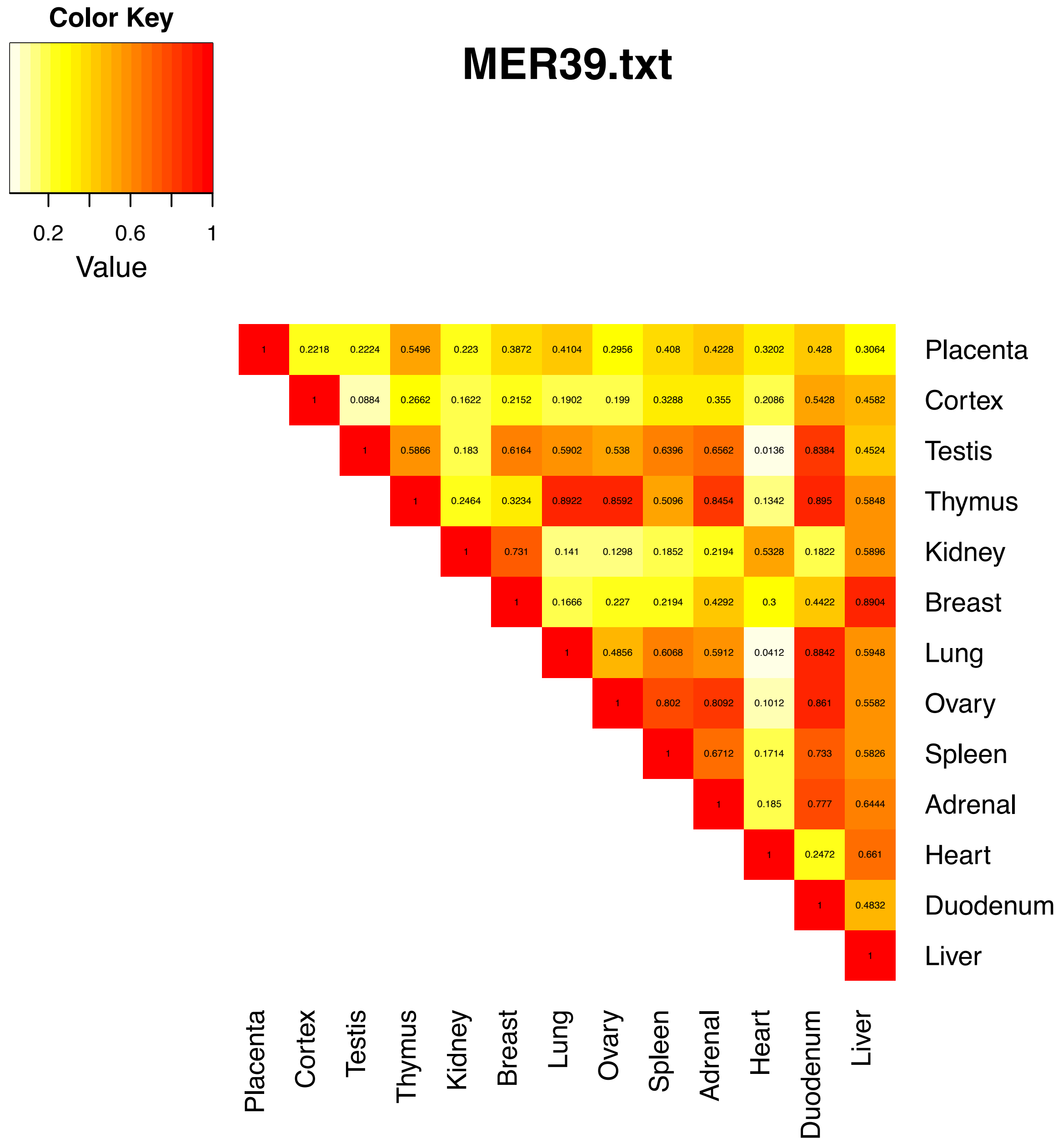
Color Key
0.2
0.6
1
Value
MER39.txt
Placenta
Cortex
Testis
Thymus
Kidney
Breast
Lung
Ovary
Spleen
Adrenal
Heart
Duodenum
Liver
1 0.2218 0.2224 0.5496 0.223 0.3872 0.4104 0.2956 0.408 0.4228 0.3202 0.428 0.3064
1 0.0884 0.2662 0.1622 0.2152 0.1902 0.199 0.3288 0.355 0.2086 0.5428 0.4582
1 0.5866 0.183 0.6164 0.5902 0.538 0.6396 0.6562 0.0136 0.8384 0.4524
1 0.2464 0.3234 0.8922 0.8592 0.5096 0.8454 0.1342 0.895 0.5848
1 0.731 0.141 0.1298 0.1852 0.2194 0.5328 0.1822 0.5896
1 0.1666 0.227 0.2194 0.4292 0.3 0.4422 0.8904
1 0.4856 0.6068 0.5912 0.0412 0.8842 0.5948
1 0.802 0.8092 0.1012 0.861 0.5582
1 0.6712 0.1714 0.733 0.5826
1 0.185 0.777 0.6444
1 0.2472 0.661
1 0.4832
1

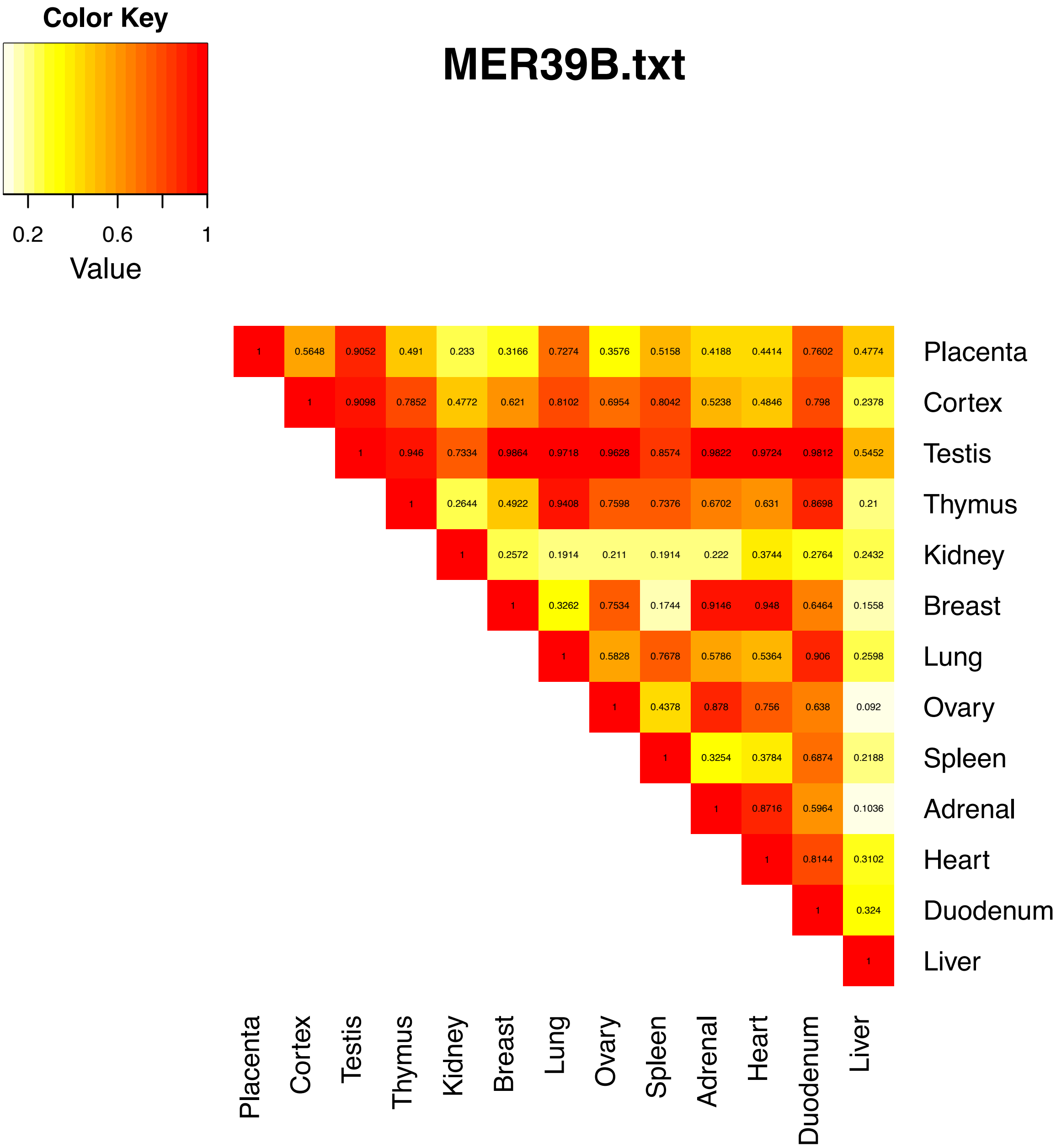
Color Key
0.2
0.6
1
Value
MER39B.txt
Placenta 1 0.5648 0.9052 0.491 0.233 0.3166 0.7274 0.3576 0.5158 0.4188 0.4414 0.7602 0.4774
Cortex 1 0.9098 0.7852 0.4772 0.621 0.8102 0.6954 0.8042 0.5238 0.4846 0.798 0.2378
Testis 1 0.946 0.7334 0.9864 0.9718 0.9628 0.8574 0.9822 0.9724 0.9812 0.5452
Thymus 1 0.2644 0.4922 0.9408 0.7598 0.7376 0.6702 0.631 0.8698 0.21
Kidney 1 0.2572 0.1914 0.211 0.1914 0.222 0.3744 0.2764 0.2432
Breast 1 0.3262 0.7534 0.1744 0.9146 0.948 0.6464 0.1558
Lung 1 0.5828 0.7678 0.5786 0.5364 0.906 0.2598
Ovary 1 0.4378 0.878 0.756 0.638 0.092
Spleen 1 0.3254 0.3784 0.6874 0.2188
Adrenal 1 0.8716 0.5964 0.1036
Heart 1 0.8144 0.3102
Duodenum 1 0.324
Liver 1
Placenta
Cortex
Testis
Thymus
Kidney
Breast
Lung
Ovary
Spleen
Adrenal
Heart
Duodenum
Liver

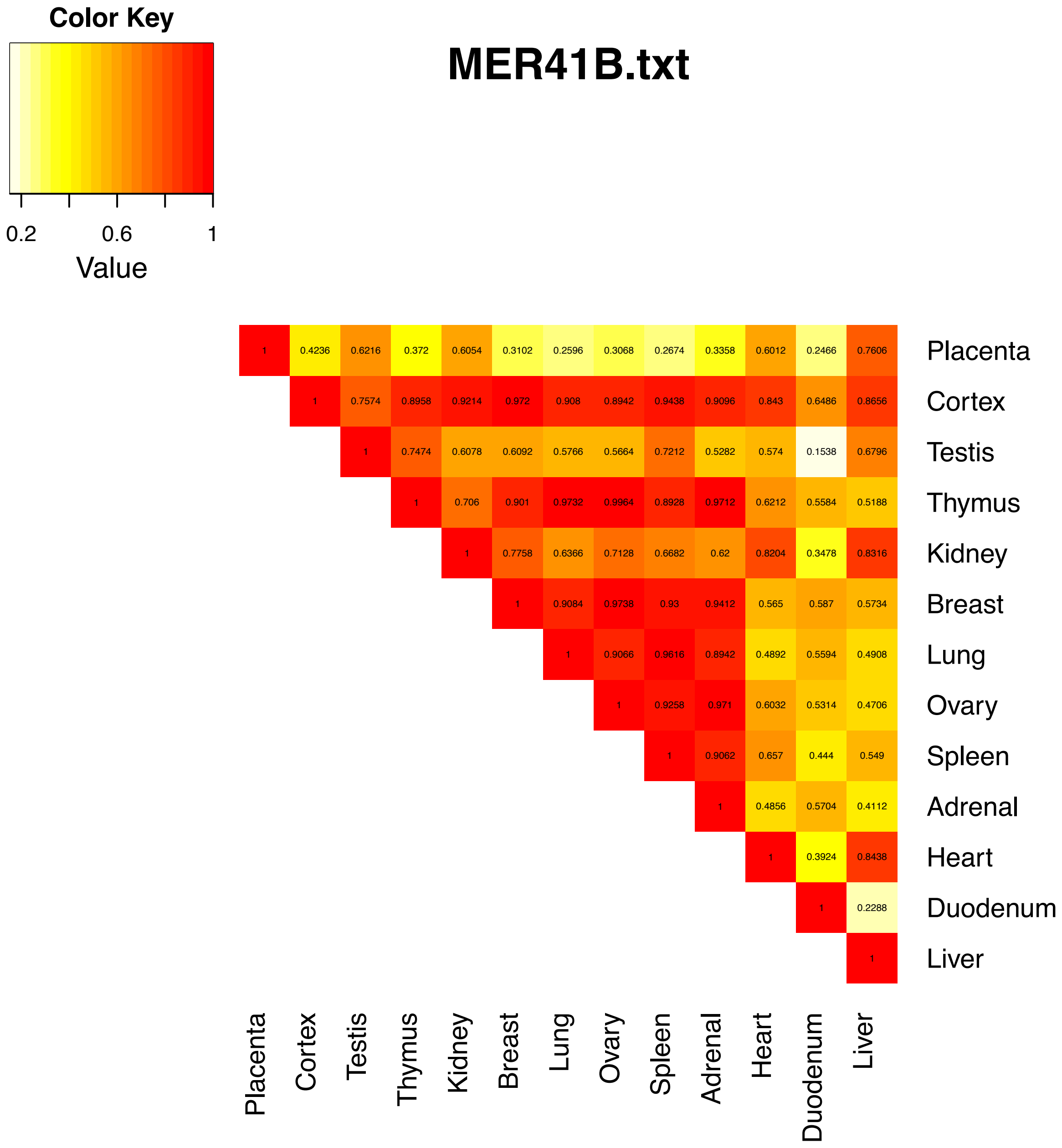
Color Key
0.2
0.6
1
Value
MER41B.txt
1 0.4236 0.6216 0.372 0.6054 0.3102 0.2596 0.3068 0.2674 0.3358 0.6012 0.2466 0.7606 Placenta
1 0.7574 0.8958 0.9214 0.972 0.908 0.8942 0.9438 0.9096 0.843 0.6486 0.8656 Cortex
1 0.7474 0.6078 0.6092 0.5766 0.5664 0.7212 0.5282 0.574 0.1538 0.6796 Testis
1 0.706 0.901 0.9732 0.9964 0.8928 0.9712 0.6212 0.5584 0.5188 Thymus
1 0.7758 0.6366 0.7128 0.6682 0.62 0.8204 0.3478 0.8316 Kidney
1 0.9084 0.9738 0.93 0.9412 0.565 0.587 0.5734 Breast
1 0.9066 0.9616 0.8942 0.4892 0.5594 0.4908 Lung
1 0.9258 0.971 0.6032 0.5314 0.4706 Ovary
1 0.9062 0.657 0.444 0.549 Spleen
1 0.4856 0.5704 0.4112 Adrenal
1 0.3924 0.8438 Heart
1 0.2288 Duodenum
1 Liver
Placenta
Cortex
Testis
Thymus
Kidney
Breast
Lung
Ovary
Spleen
Adrenal
Heart
Duodenum
Liver

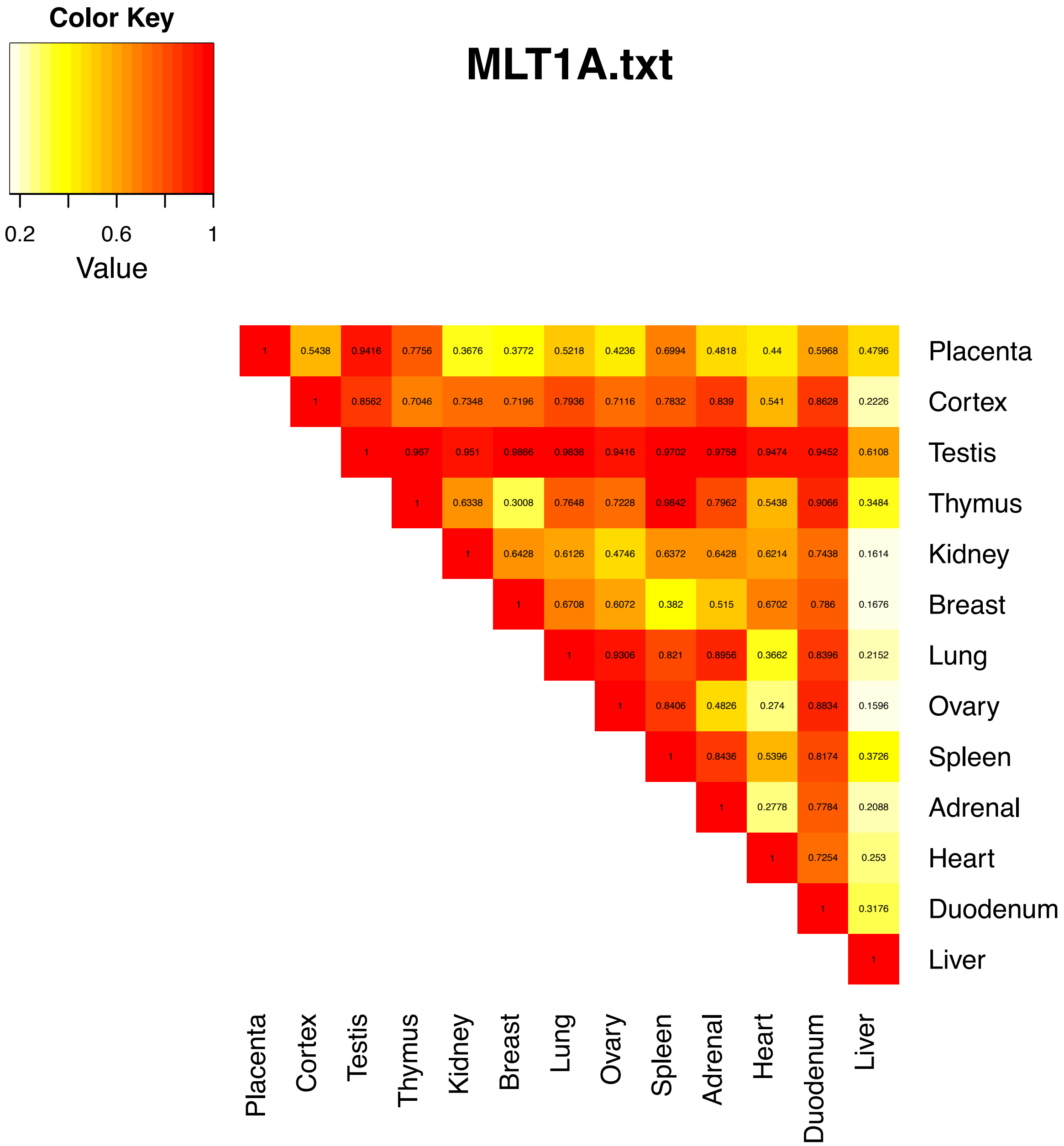
Color Key
0.2
0.6
1
Value
MLT1A.txt
Placenta: 1, 0.5438, 0.9416, 0.7756, 0.3676, 0.3772, 0.5218, 0.4236, 0.6994, 0.4818, 0.44, 0.5968, 0.4796
Cortex: 1, 0.8562, 0.7046, 0.7348, 0.7196, 0.7936, 0.7116, 0.7832, 0.839, 0.541, 0.8628, 0.2226
Testis: 1, 0.967, 0.951, 0.9866, 0.9836, 0.9416, 0.9702, 0.9758, 0.9474, 0.9452, 0.6108
Thymus: 1, 0.6338, 0.3008, 0.7648, 0.7228, 0.9842, 0.7962, 0.5438, 0.9066, 0.3484
Kidney: 1, 0.6428, 0.6126, 0.4746, 0.6372, 0.6428, 0.6214, 0.7438, 0.1614
Breast: 1, 0.6708, 0.6072, 0.382, 0.515, 0.6702, 0.786, 0.1676
Lung: 1, 0.9306, 0.821, 0.8956, 0.3662, 0.8396, 0.2152
Ovary: 1, 0.8406, 0.4826, 0.274, 0.8834, 0.1596
Spleen: 1, 0.8436, 0.5396, 0.8174, 0.3726
Adrenal: 1, 0.2778, 0.7784, 0.2088
Heart: 1, 0.7254, 0.253
Duodenum: 1, 0.3176
Liver: 1
Placenta
Cortex
Testis
Thymus
Kidney
Breast
Lung
Ovary
Spleen
Adrenal
Heart
Duodenum
Liver

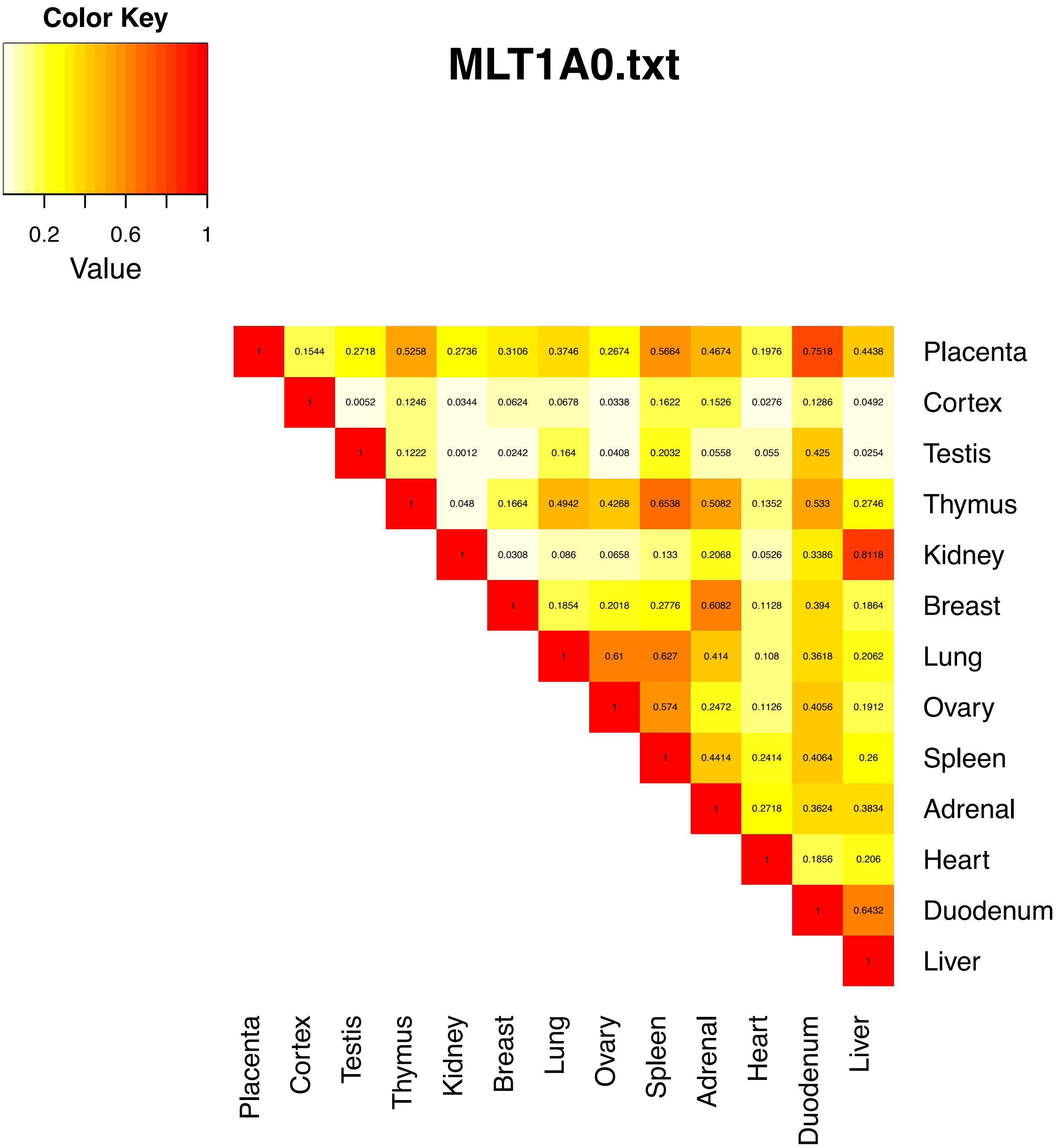
Color Key
0.2 0.6 1
Value
MLT1A0.txt
Placenta: 1 0.1544 0.2718 0.5258 0.2736 0.3106 0.3746 0.2674 0.5664 0.4674 0.1976 0.7518 0.4438
Cortex: 1 0.0052 0.1246 0.0344 0.0624 0.0678 0.0338 0.1622 0.1526 0.0276 0.1286 0.0492
Testis: 1 0.1222 0.0012 0.0242 0.164 0.0408 0.2032 0.0558 0.055 0.425 0.0254
Thymus: 1 0.048 0.1664 0.4942 0.4268 0.6538 0.5082 0.1352 0.533 0.2746
Kidney: 1 0.0308 0.086 0.0658 0.133 0.2068 0.0526 0.3386 0.8118
Breast: 1 0.1854 0.2018 0.2776 0.6082 0.1128 0.394 0.1864
Lung: 1 0.61 0.627 0.414 0.108 0.3618 0.2062
Ovary: 1 0.574 0.2472 0.1126 0.4056 0.1912
Spleen: 1 0.4414 0.2414 0.4064 0.26
Adrenal: 1 0.2718 0.3624 0.3834
Heart: 1 0.1856 0.206
Duodenum: 1 0.6432
Liver: 1
Placenta
Cortex
Testis
Thymus
Kidney
Breast
Lung
Ovary
Spleen
Adrenal
Heart
Duodenum
Liver

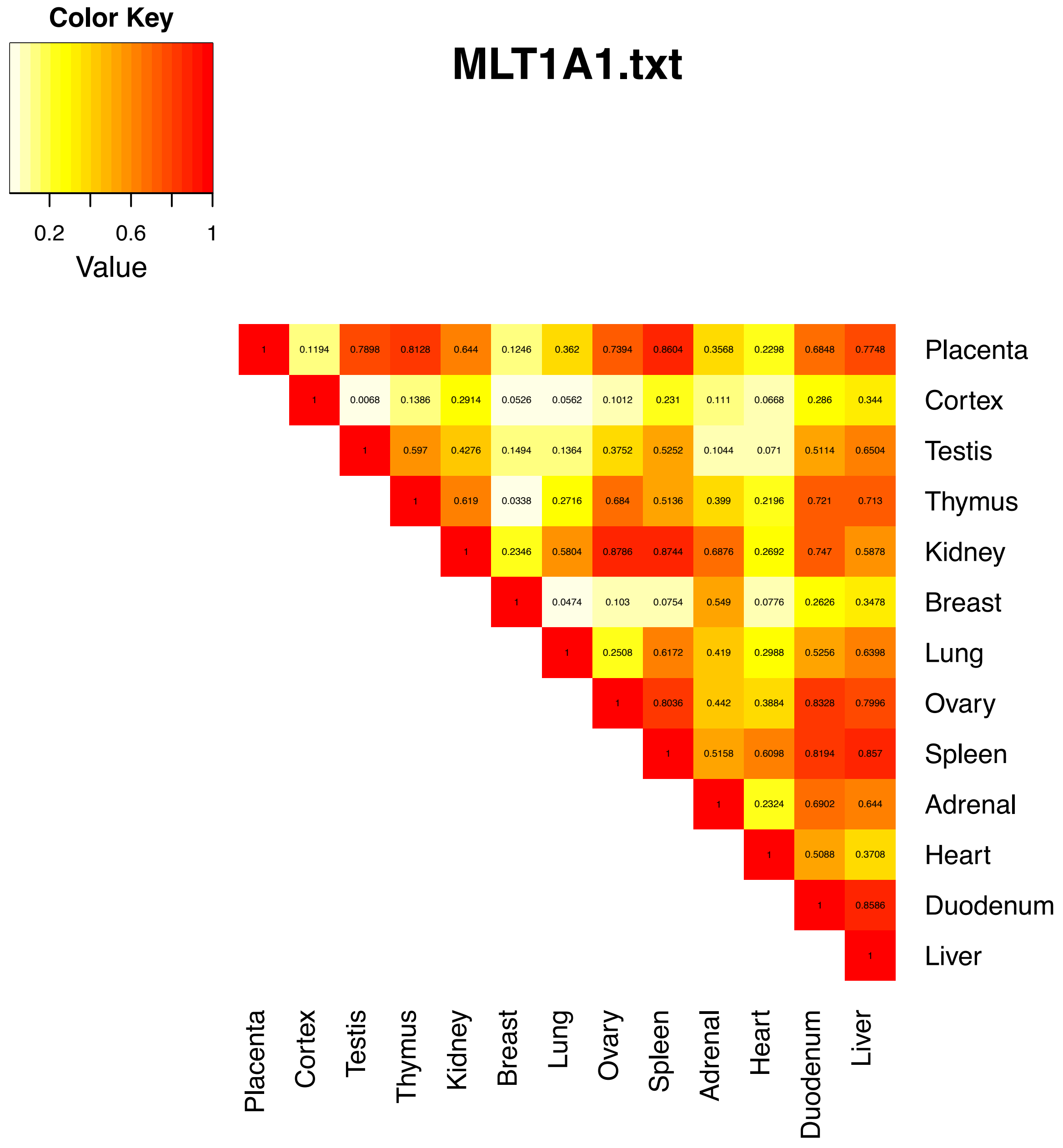
Color Key
MLT1A1.txt
0.2
0.6
1
Value
1 0.1194 0.7898 0.8128 0.644 0.1246 0.362 0.7394 0.8604 0.3568 0.2298 0.6848 0.7748 Placenta
1 0.0068 0.1386 0.2914 0.0526 0.0562 0.1012 0.231 0.111 0.0668 0.286 0.344 Cortex
1 0.597 0.4276 0.1494 0.1364 0.3752 0.5252 0.1044 0.071 0.5114 0.6504 Testis
1 0.619 0.0338 0.2716 0.684 0.5136 0.399 0.2196 0.721 0.713 Thymus
1 0.2346 0.5804 0.8786 0.8744 0.6876 0.2692 0.747 0.5878 Kidney
1 0.0474 0.103 0.0754 0.549 0.0776 0.2626 0.3478 Breast
1 0.2508 0.6172 0.419 0.2988 0.5256 0.6398 Lung
1 0.8036 0.442 0.3884 0.8328 0.7996 Ovary
1 0.5158 0.6098 0.8194 0.857 Spleen
1 0.2324 0.6902 0.644 Adrenal
1 0.5088 0.3708 Heart
1 0.8586 Duodenum
1 Liver
Placenta
Cortex
Testis
Thymus
Kidney
Breast
Lung
Ovary
Spleen
Adrenal
Heart
Duodenum
Liver

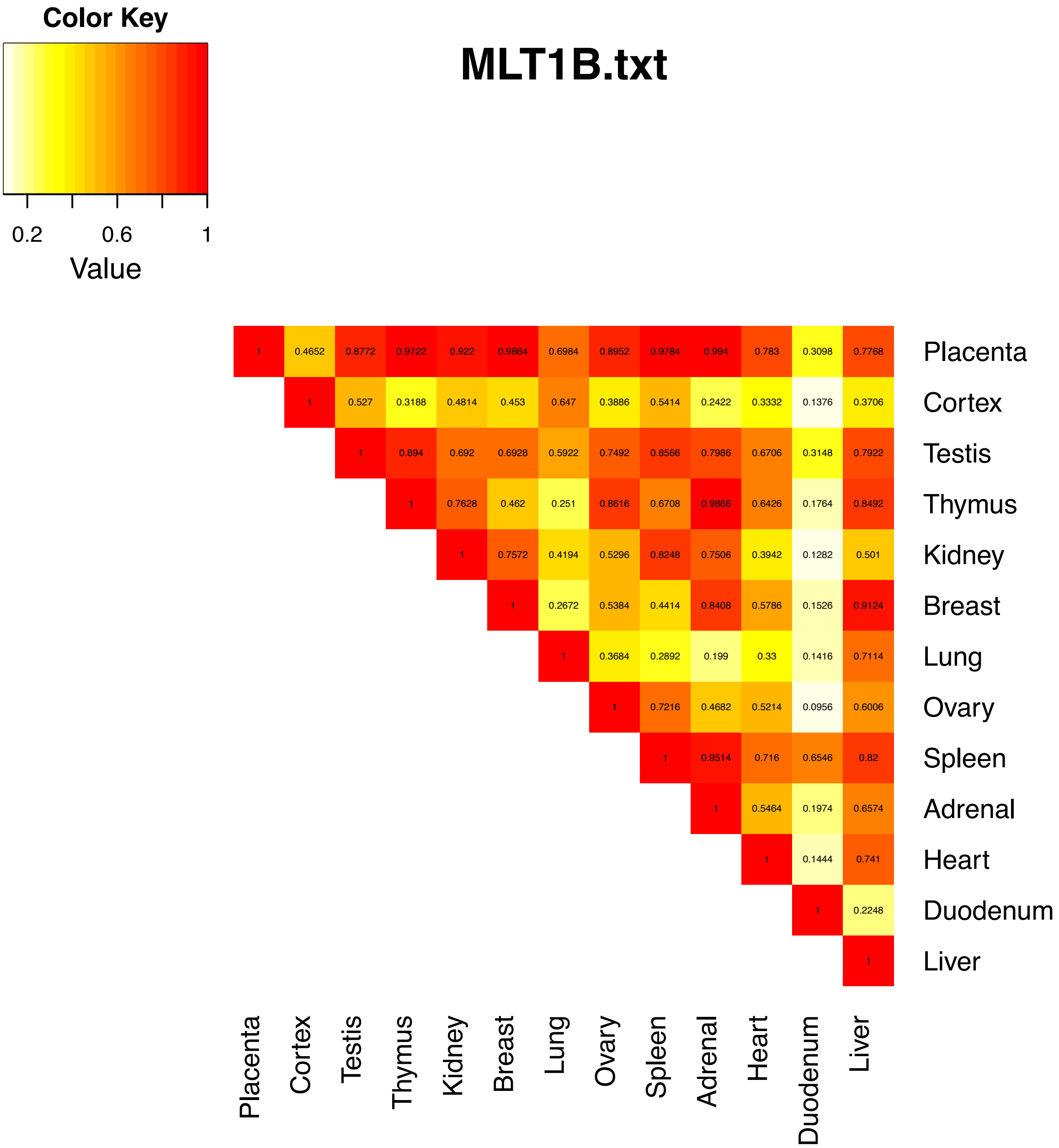
Color Key
0.2
0.6
1
Value
MLT1B.txt
Placenta
Cortex
Testis
Thymus
Kidney
Breast
Lung
Ovary
Spleen
Adrenal
Heart
Duodenum
Liver
1 0.4652 0.8772 0.9722 0.922 0.9864 0.6984 0.8952 0.9784 0.994 0.783 0.3098 0.7768
1 0.527 0.3188 0.4814 0.453 0.647 0.3886 0.5414 0.2422 0.3332 0.1376 0.3706
1 0.894 0.692 0.6928 0.5922 0.7492 0.8566 0.7986 0.6706 0.3148 0.7922
1 0.7628 0.462 0.251 0.8616 0.6708 0.9866 0.6426 0.1764 0.8492
1 0.7572 0.4194 0.5296 0.8248 0.7506 0.3942 0.1282 0.501
1 0.2672 0.5384 0.4414 0.8408 0.5786 0.1526 0.9124
1 0.3684 0.2892 0.199 0.33 0.1416 0.7114
1 0.7216 0.4682 0.5214 0.0956 0.6006
1 0.9514 0.716 0.6546 0.82
1 0.5464 0.1974 0.6574
1 0.1444 0.741
1 0.2248
1

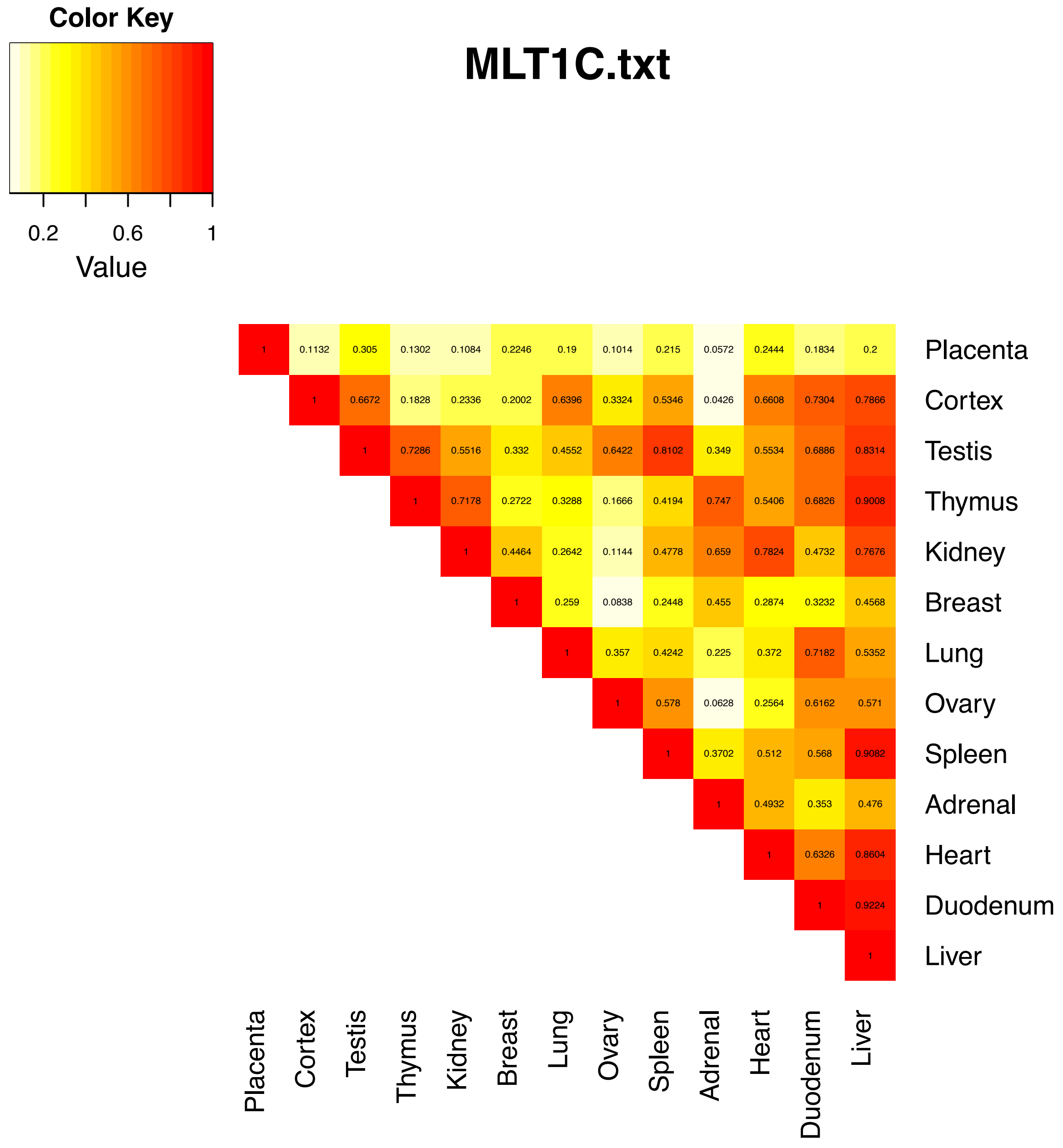
Color Key
0.2 0.6 1
Value
MLT1C.txt
1 0.1132 0.305 0.1302 0.1084 0.2246 0.19 0.1014 0.215 0.0572 0.2444 0.1834 0.2 Placenta
1 0.6672 0.1828 0.2336 0.2002 0.6396 0.3324 0.5346 0.0426 0.6608 0.7304 0.7866 Cortex
1 0.7286 0.5516 0.332 0.4552 0.6422 0.8102 0.349 0.5534 0.6886 0.8314 Testis
1 0.7178 0.2722 0.3288 0.1666 0.4194 0.747 0.5406 0.6826 0.9008 Thymus
1 0.4464 0.2642 0.1144 0.4778 0.659 0.7824 0.4732 0.7676 Kidney
1 0.259 0.0838 0.2448 0.455 0.2874 0.3232 0.4568 Breast
1 0.357 0.4242 0.225 0.372 0.7182 0.5352 Lung
1 0.578 0.0628 0.2564 0.6162 0.571 Ovary
1 0.3702 0.512 0.568 0.9082 Spleen
1 0.4932 0.353 0.476 Adrenal
1 0.6326 0.8604 Heart
1 0.9224 Duodenum
1 Liver
Placenta Cortex Testis Thymus Kidney Breast Lung Ovary Spleen Adrenal Heart Duodenum Liver

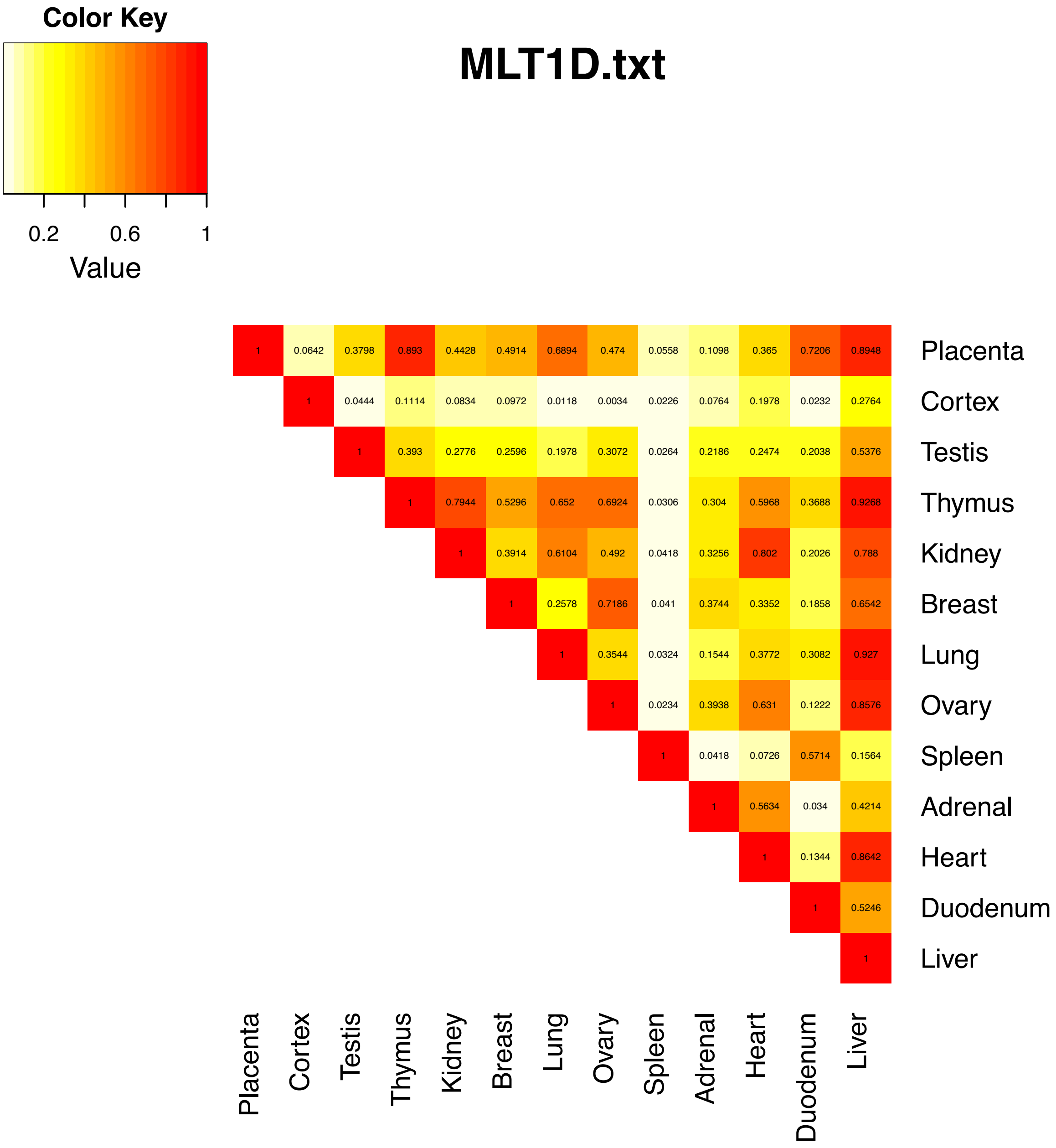
Color Key
0.2 0.6 1
Value
MLT1D.txt
1 0.0642 0.3798 0.893 0.4428 0.4914 0.6894 0.474 0.0558 0.1098 0.365 0.7206 0.8948
1 0.0444 0.1114 0.0834 0.0972 0.0118 0.0034 0.0226 0.0764 0.1978 0.0232 0.2764
1 0.393 0.2776 0.2596 0.1978 0.3072 0.0264 0.2186 0.2474 0.2038 0.5376
1 0.7944 0.5296 0.652 0.6924 0.0306 0.304 0.5968 0.3688 0.9268
1 0.3914 0.6104 0.492 0.0418 0.3256 0.802 0.2026 0.788
1 0.2578 0.7186 0.041 0.3744 0.3352 0.1858 0.6542
1 0.3544 0.0324 0.1544 0.3772 0.3082 0.927
1 0.0234 0.3938 0.631 0.1222 0.8576
1 0.0418 0.0726 0.5714 0.1564
1 0.5634 0.034 0.4214
1 0.1344 0.8642
1 0.5246
1
Placenta
Cortex
Testis
Thymus
Kidney
Breast
Lung
Ovary
Spleen
Adrenal
Heart
Duodenum
Liver

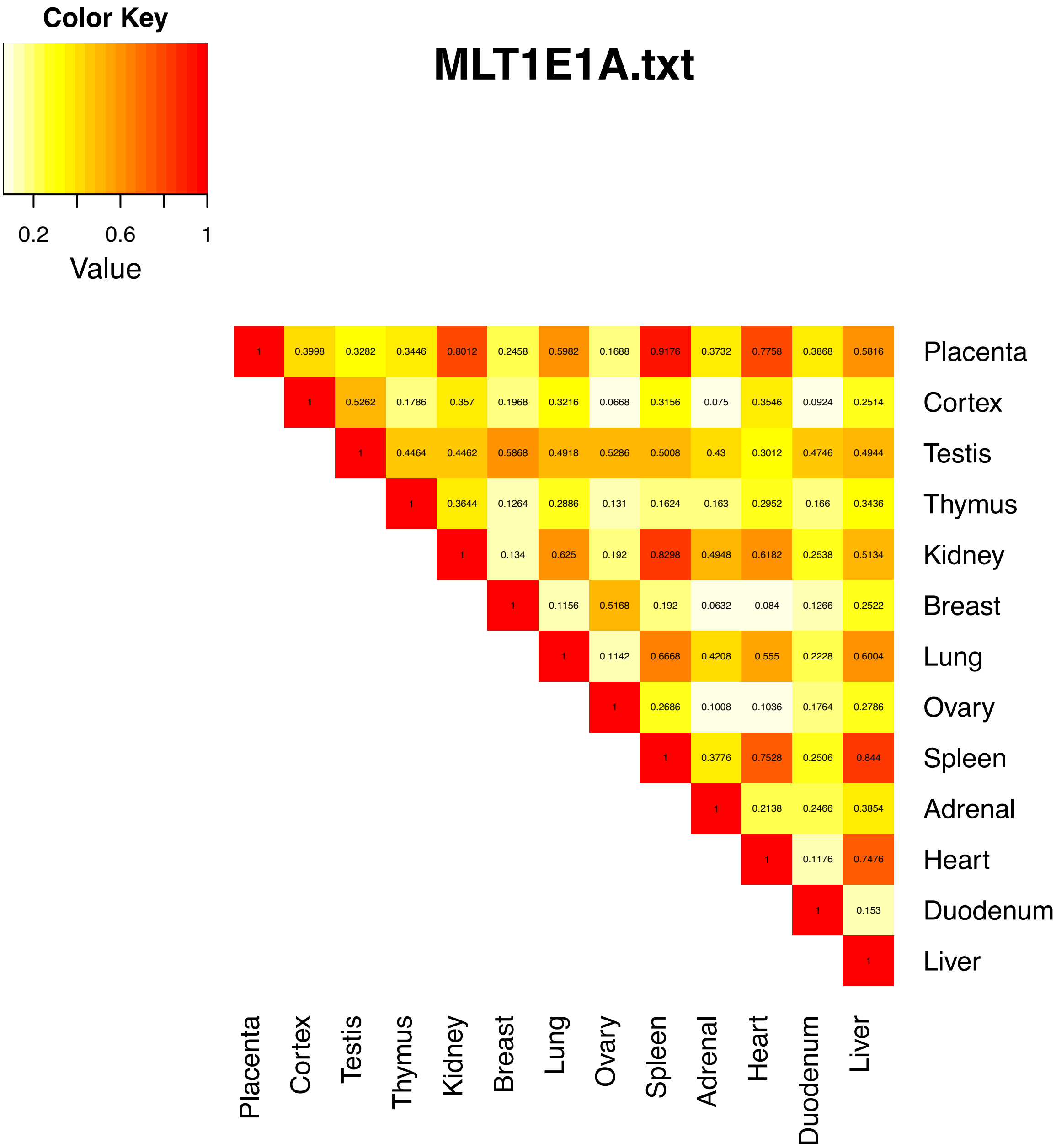
Color Key
0.2
0.6
1
Value
MLT1E1A.txt
Placenta
Cortex
Testis
Thymus
Kidney
Breast
Lung
Ovary
Spleen
Adrenal
Heart
Duodenum
Liver
1 0.3998 0.3282 0.3446 0.8012 0.2458 0.5982 0.1688 0.9176 0.3732 0.7758 0.3868 0.5816
1 0.5262 0.1786 0.357 0.1968 0.3216 0.0668 0.3156 0.075 0.3546 0.0924 0.2514
1 0.4464 0.4462 0.5868 0.4918 0.5286 0.5008 0.43 0.3012 0.4746 0.4944
1 0.3644 0.1264 0.2886 0.131 0.1624 0.163 0.2952 0.166 0.3436
1 0.134 0.625 0.192 0.8298 0.4948 0.6182 0.2538 0.5134
1 0.1156 0.5168 0.192 0.0632 0.084 0.1266 0.2522
1 0.1142 0.6668 0.4208 0.555 0.2228 0.6004
1 0.2686 0.1008 0.1036 0.1764 0.2786
1 0.3776 0.7528 0.2506 0.844
1 0.2138 0.2466 0.3854
1 0.1176 0.7476
1 0.153
1

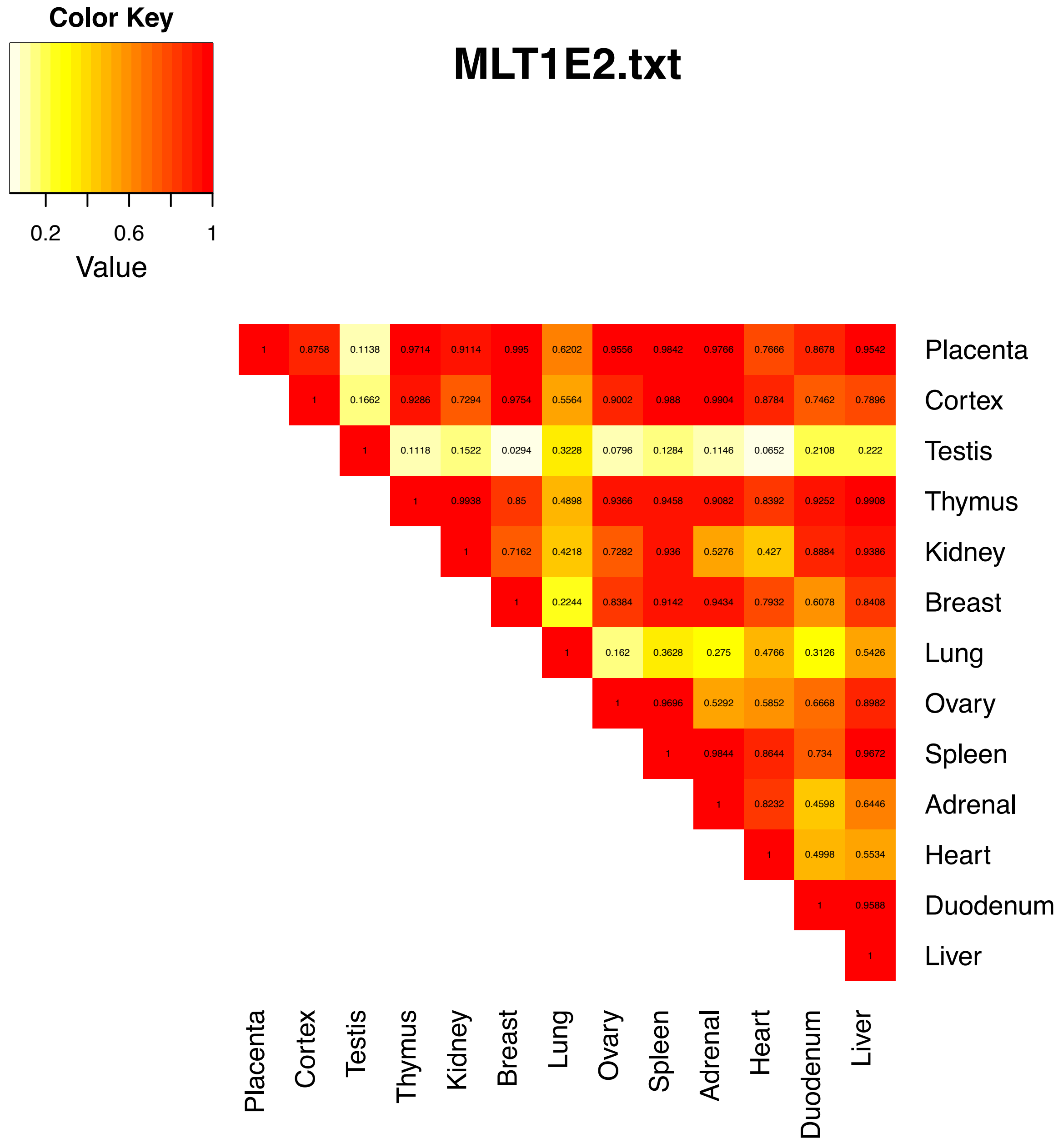
Color Key
0.2
0.6
1
Value
MLT1E2.txt
Placenta
Cortex
Testis
Thymus
Kidney
Breast
Lung
Ovary
Spleen
Adrenal
Heart
Duodenum
Liver
1 0.8758 0.1138 0.9714 0.9114 0.995 0.6202 0.9556 0.9842 0.9766 0.7666 0.8678 0.9542
1 0.1662 0.9286 0.7294 0.9754 0.5564 0.9002 0.988 0.9904 0.8784 0.7462 0.7896
1 0.1118 0.1522 0.0294 0.3228 0.0796 0.1284 0.1146 0.0652 0.2108 0.222
1 0.9938 0.85 0.4898 0.9366 0.9458 0.9082 0.8392 0.9252 0.9908
1 0.7162 0.4218 0.7282 0.936 0.5276 0.427 0.8884 0.9386
1 0.2244 0.8384 0.9142 0.9434 0.7932 0.6078 0.8408
1 0.162 0.3628 0.275 0.4766 0.3126 0.5426
1 0.9696 0.5292 0.5852 0.6668 0.8982
1 0.9844 0.8644 0.734 0.9672
1 0.8232 0.4598 0.6446
1 0.4998 0.5534
1 0.9588
1

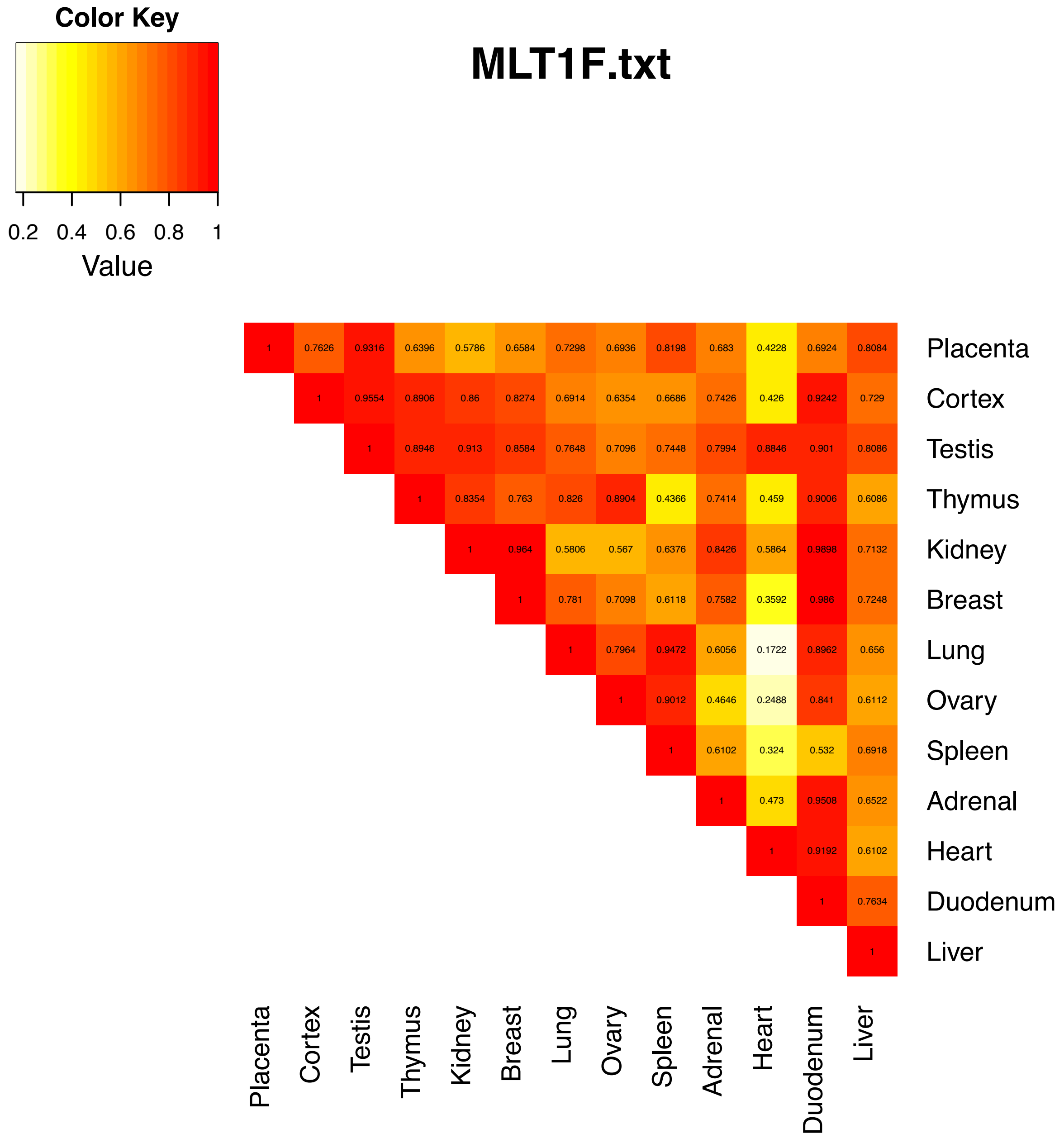
Color Key
0.2 0.4 0.6 0.8 1
Value
MLT1F.txt
1 0.7626 0.9316 0.6396 0.5786 0.6584 0.7298 0.6936 0.8198 0.683 0.4228 0.6924 0.8084 Placenta
1 0.9554 0.8906 0.86 0.8274 0.6914 0.6354 0.6686 0.7426 0.426 0.9242 0.729 Cortex
1 0.8946 0.913 0.8584 0.7648 0.7096 0.7448 0.7994 0.8846 0.901 0.8086 Testis
1 0.8354 0.763 0.826 0.8904 0.4366 0.7414 0.459 0.9006 0.6086 Thymus
1 0.964 0.5806 0.567 0.6376 0.8426 0.5864 0.9898 0.7132 Kidney
1 0.781 0.7098 0.6118 0.7582 0.3592 0.986 0.7248 Breast
1 0.7964 0.9472 0.6056 0.1722 0.8962 0.656 Lung
1 0.9012 0.4646 0.2488 0.841 0.6112 Ovary
1 0.6102 0.324 0.532 0.6918 Spleen
1 0.473 0.9508 0.6522 Adrenal
1 0.9192 0.6102 Heart
1 0.7634 Duodenum
1 Liver
Placenta Cortex Testis Thymus Kidney Breast Lung Ovary Spleen Adrenal Heart Duodenum Liver

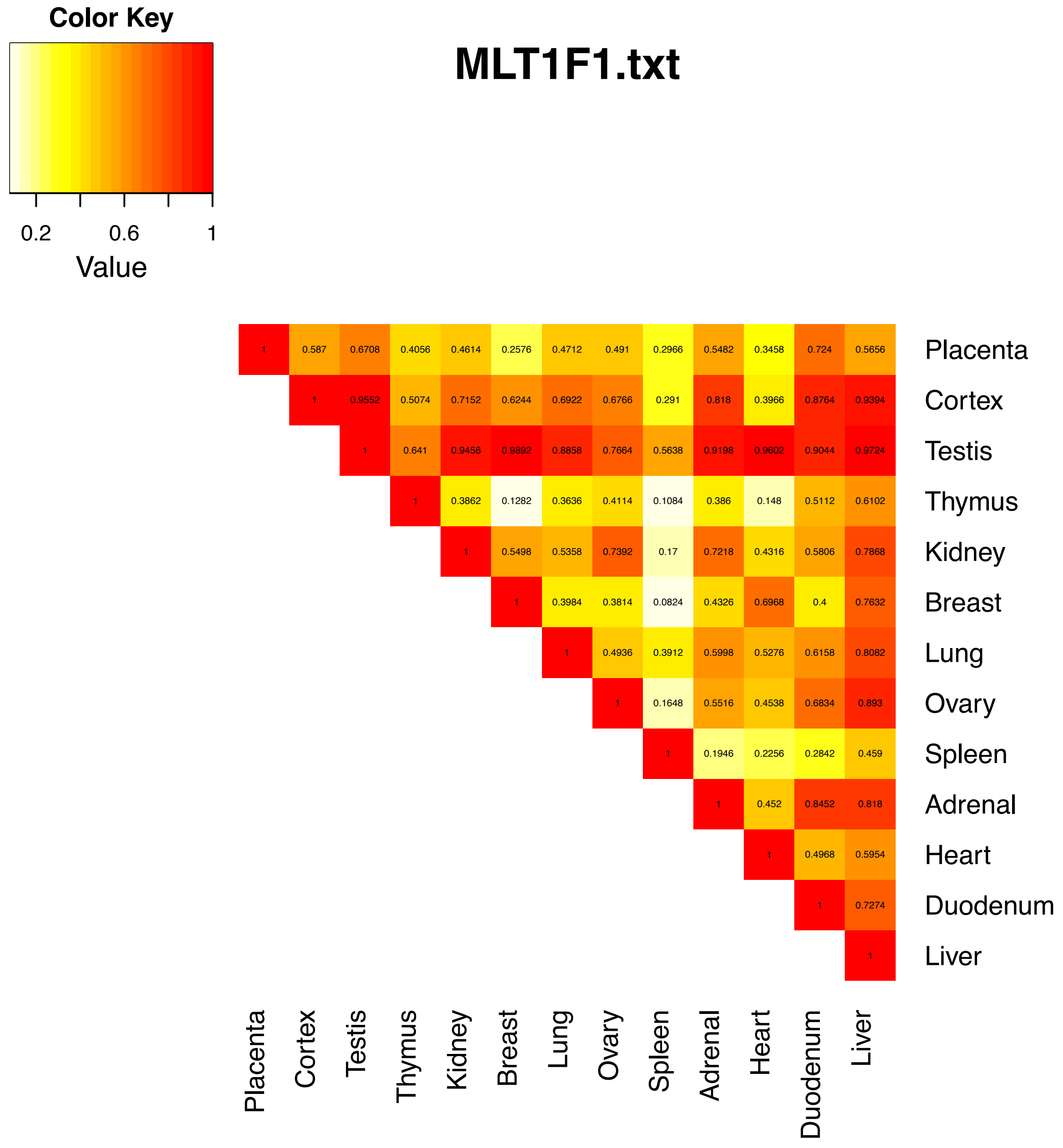
Color Key
0.2
0.6
1
Value
MLT1F1.txt
Placenta: 1 0.587 0.6708 0.4056 0.4614 0.2576 0.4712 0.491 0.2966 0.5482 0.3458 0.724 0.5656
Cortex: 1 0.9552 0.5074 0.7152 0.6244 0.6922 0.6766 0.291 0.818 0.3966 0.8764 0.9394
Testis: 1 0.641 0.9456 0.9892 0.8858 0.7664 0.5638 0.9198 0.9602 0.9044 0.9724
Thymus: 1 0.3862 0.1282 0.3636 0.4114 0.1084 0.386 0.148 0.5112 0.6102
Kidney: 1 0.5498 0.5358 0.7392 0.17 0.7218 0.4316 0.5806 0.7868
Breast: 1 0.3984 0.3814 0.0824 0.4326 0.6968 0.4 0.7632
Lung: 1 0.4936 0.3912 0.5998 0.5276 0.6158 0.8082
Ovary: 1 0.1648 0.5516 0.4538 0.6834 0.893
Spleen: 1 0.1946 0.2256 0.2842 0.459
Adrenal: 1 0.452 0.8452 0.818
Heart: 1 0.4968 0.5954
Duodenum: 1 0.7274
Liver: 1
Placenta
Cortex
Testis
Thymus
Kidney
Breast
Lung
Ovary
Spleen
Adrenal
Heart
Duodenum
Liver

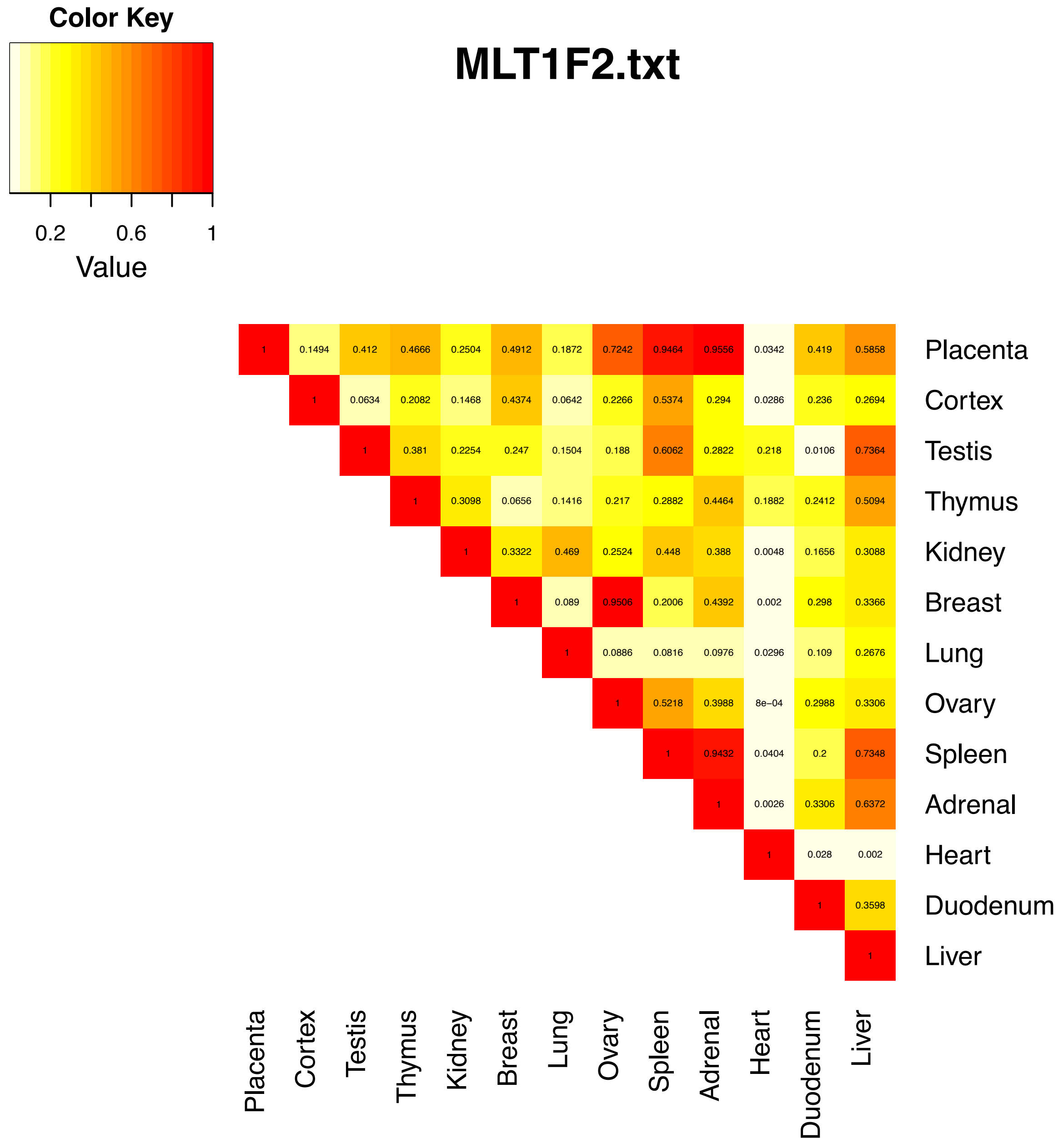
MLT1F2.txt
Color Key
0.2 0.6 1
Value
Placenta: 1 0.1494 0.412 0.4666 0.2504 0.4912 0.1872 0.7242 0.9464 0.9556 0.0342 0.419 0.5858
Cortex: 1 0.0634 0.2082 0.1468 0.4374 0.0642 0.2266 0.5374 0.294 0.0286 0.236 0.2694
Testis: 1 0.381 0.2254 0.247 0.1504 0.188 0.6062 0.2822 0.218 0.0106 0.7364
Thymus: 1 0.3098 0.0656 0.1416 0.217 0.2882 0.4464 0.1882 0.2412 0.5094
Kidney: 1 0.3322 0.469 0.2524 0.448 0.388 0.0048 0.1656 0.3088
Breast: 1 0.089 0.9506 0.2006 0.4392 0.002 0.298 0.3366
Lung: 1 0.0886 0.0816 0.0976 0.0296 0.109 0.2676
Ovary: 1 0.5218 0.3988 8e−04 0.2988 0.3306
Spleen: 1 0.9432 0.0404 0.2 0.7348
Adrenal: 1 0.0026 0.3306 0.6372
Heart: 1 0.028 0.002
Duodenum: 1 0.3598
Liver: 1
Placenta Cortex Testis Thymus Kidney Breast Lung Ovary Spleen Adrenal Heart Duodenum Liver

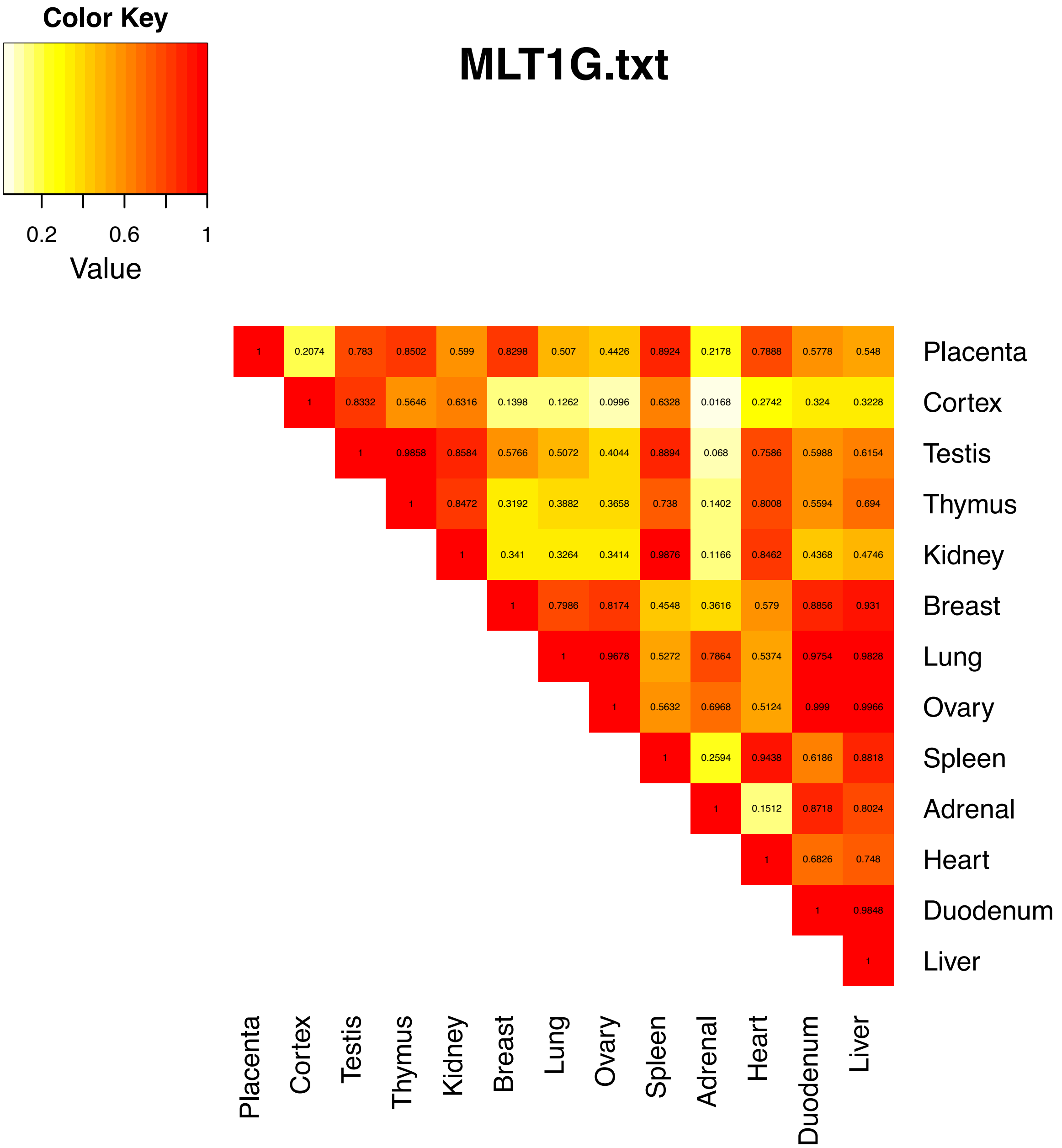
Color Key
0.2
0.6
1
Value
MLT1G.txt
Placenta
Cortex
Testis
Thymus
Kidney
Breast
Lung
Ovary
Spleen
Adrenal
Heart
Duodenum
Liver
1 0.2074 0.783 0.8502 0.599 0.8298 0.507 0.4426 0.8924 0.2178 0.7888 0.5778 0.548
1 0.8332 0.5646 0.6316 0.1398 0.1262 0.0996 0.6328 0.0168 0.2742 0.324 0.3228
1 0.9858 0.8584 0.5766 0.5072 0.4044 0.8894 0.068 0.7586 0.5988 0.6154
1 0.8472 0.3192 0.3882 0.3658 0.738 0.1402 0.8008 0.5594 0.694
1 0.341 0.3264 0.3414 0.9876 0.1166 0.8462 0.4368 0.4746
1 0.7986 0.8174 0.4548 0.3616 0.579 0.8856 0.931
1 0.9678 0.5272 0.7864 0.5374 0.9754 0.9828
1 0.5632 0.6968 0.5124 0.999 0.9966
1 0.2594 0.9438 0.6186 0.8818
1 0.1512 0.8718 0.8024
1 0.6826 0.748
1 0.9848
1

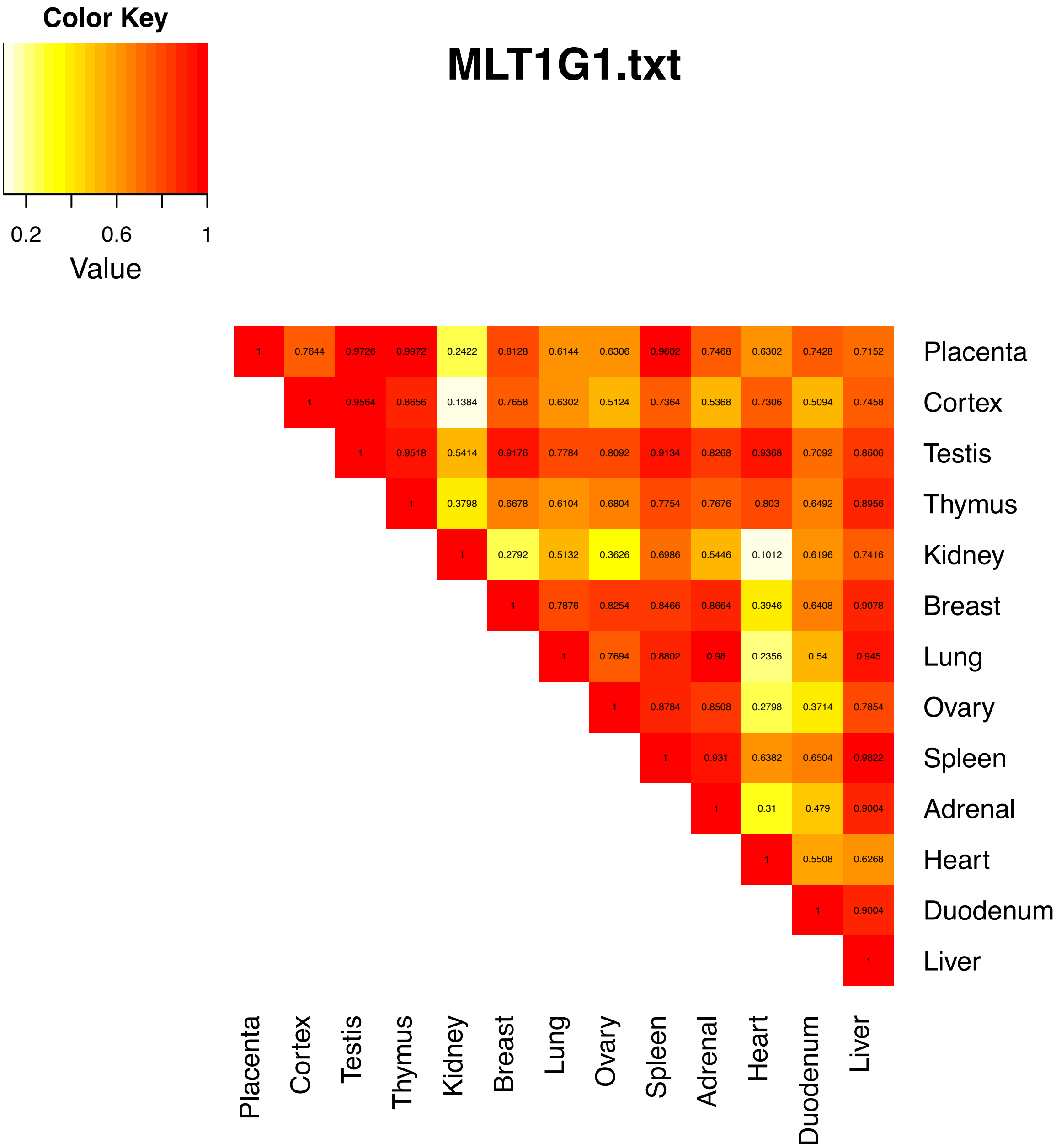
Color Key
0.2
0.6
1
Value
MLT1G1.txt
Placenta
Cortex
Testis
Thymus
Kidney
Breast
Lung
Ovary
Spleen
Adrenal
Heart
Duodenum
Liver
1 0.7644 0.9726 0.9972 0.2422 0.8128 0.6144 0.6306 0.9602 0.7468 0.6302 0.7428 0.7152
1 0.9564 0.8656 0.1384 0.7658 0.6302 0.5124 0.7364 0.5368 0.7306 0.5094 0.7458
1 0.9518 0.5414 0.9176 0.7784 0.8092 0.9134 0.8268 0.9368 0.7092 0.8606
1 0.3798 0.6678 0.6104 0.6804 0.7754 0.7676 0.803 0.6492 0.8956
1 0.2792 0.5132 0.3626 0.6986 0.5446 0.1012 0.6196 0.7416
1 0.7876 0.8254 0.8466 0.8664 0.3946 0.6408 0.9078
1 0.7694 0.8802 0.98 0.2356 0.54 0.945
1 0.8784 0.8508 0.2798 0.3714 0.7854
1 0.931 0.6382 0.6504 0.9822
1 0.31 0.479 0.9004
1 0.5508 0.6268
1 0.9004
1

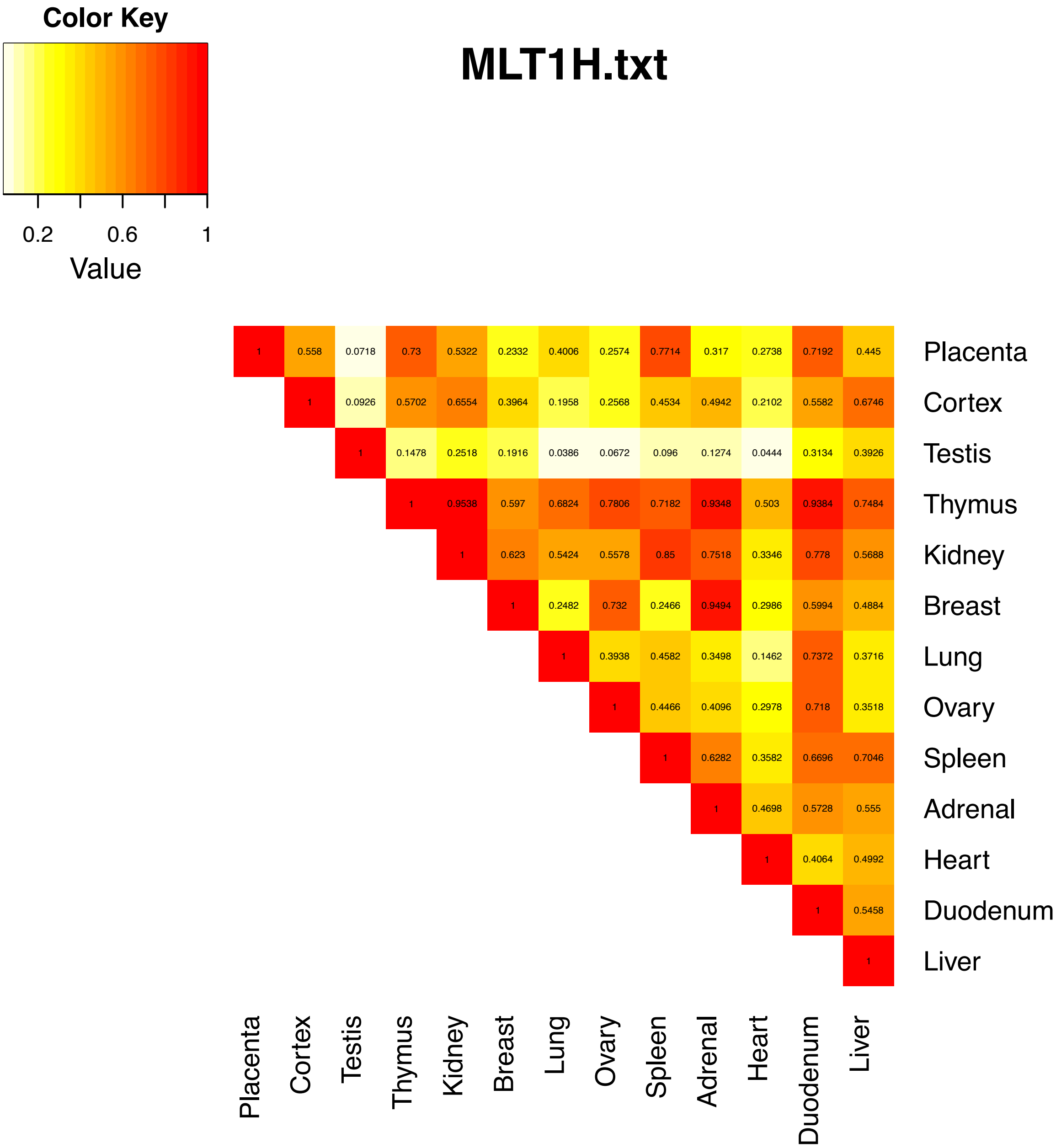
Color Key
Value
0.2
0.6
1
MLT1H.txt
Placenta
Cortex
Testis
Thymus
Kidney
Breast
Lung
Ovary
Spleen
Adrenal
Heart
Duodenum
Liver
1 0.558 0.0718 0.73 0.5322 0.2332 0.4006 0.2574 0.7714 0.317 0.2738 0.7192 0.445
1 0.0926 0.5702 0.6554 0.3964 0.1958 0.2568 0.4534 0.4942 0.2102 0.5582 0.6746
1 0.1478 0.2518 0.1916 0.0386 0.0672 0.096 0.1274 0.0444 0.3134 0.3926
1 0.9538 0.597 0.6824 0.7806 0.7182 0.9348 0.503 0.9384 0.7484
1 0.623 0.5424 0.5578 0.85 0.7518 0.3346 0.778 0.5688
1 0.2482 0.732 0.2466 0.9494 0.2986 0.5994 0.4884
1 0.3938 0.4582 0.3498 0.1462 0.7372 0.3716
1 0.4466 0.4096 0.2978 0.718 0.3518
1 0.6282 0.3582 0.6696 0.7046
1 0.4698 0.5728 0.555
1 0.4064 0.4992
1 0.5458
1

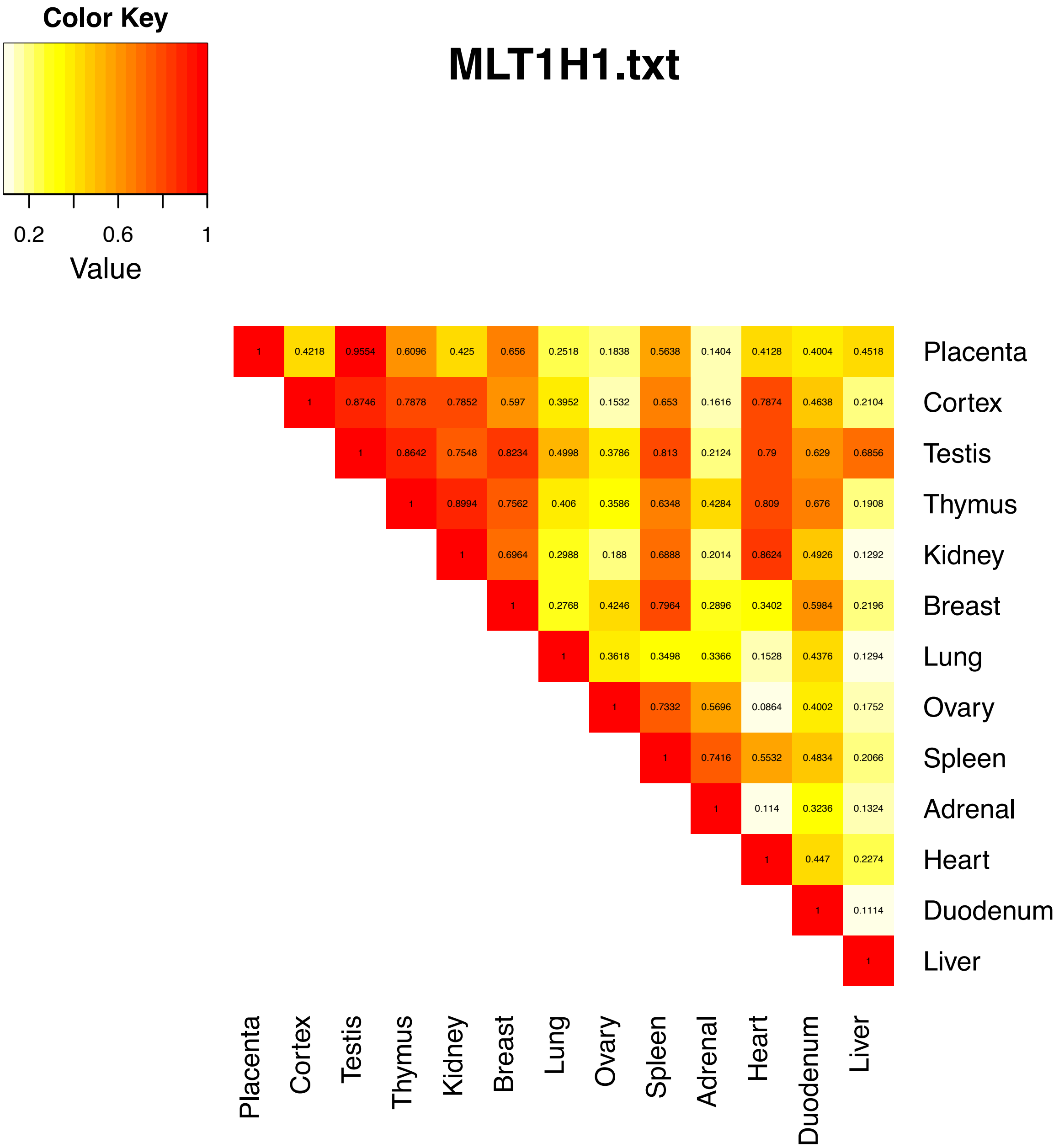
Color Key
0.2
0.6
1
Value
MLT1H1.txt
Placenta: 1 0.4218 0.9554 0.6096 0.425 0.656 0.2518 0.1838 0.5638 0.1404 0.4128 0.4004 0.4518
Cortex: 1 0.8746 0.7878 0.7852 0.597 0.3952 0.1532 0.653 0.1616 0.7874 0.4638 0.2104
Testis: 1 0.8642 0.7548 0.8234 0.4998 0.3786 0.813 0.2124 0.79 0.629 0.6856
Thymus: 1 0.8994 0.7562 0.406 0.3586 0.6348 0.4284 0.809 0.676 0.1908
Kidney: 1 0.6964 0.2988 0.188 0.6888 0.2014 0.8624 0.4926 0.1292
Breast: 1 0.2768 0.4246 0.7964 0.2896 0.3402 0.5984 0.2196
Lung: 1 0.3618 0.3498 0.3366 0.1528 0.4376 0.1294
Ovary: 1 0.7332 0.5696 0.0864 0.4002 0.1752
Spleen: 1 0.7416 0.5532 0.4834 0.2066
Adrenal: 1 0.114 0.3236 0.1324
Heart: 1 0.447 0.2274
Duodenum: 1 0.1114
Liver: 1
Placenta
Cortex
Testis
Thymus
Kidney
Breast
Lung
Ovary
Spleen
Adrenal
Heart
Duodenum
Liver

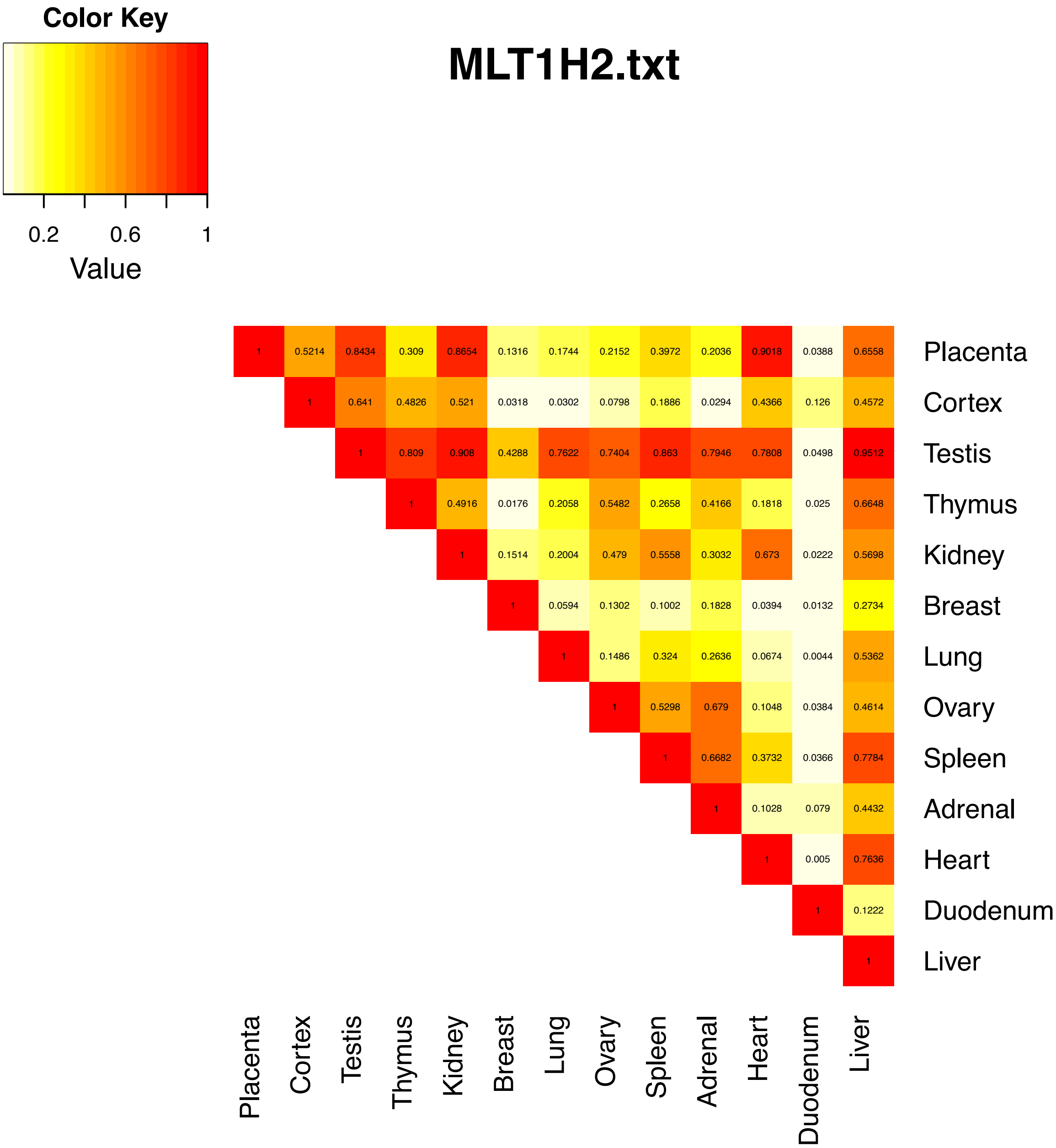
Color Key
0.2
0.6
1
Value
MLT1H2.txt
Placenta: 1, 0.5214, 0.8434, 0.309, 0.8654, 0.1316, 0.1744, 0.2152, 0.3972, 0.2036, 0.9018, 0.0388, 0.6558
Cortex: 1, 0.641, 0.4826, 0.521, 0.0318, 0.0302, 0.0798, 0.1886, 0.0294, 0.4366, 0.126, 0.4572
Testis: 1, 0.809, 0.908, 0.4288, 0.7622, 0.7404, 0.863, 0.7946, 0.7808, 0.0498, 0.9512
Thymus: 1, 0.4916, 0.0176, 0.2058, 0.5482, 0.2658, 0.4166, 0.1818, 0.025, 0.6648
Kidney: 1, 0.1514, 0.2004, 0.479, 0.5558, 0.3032, 0.673, 0.0222, 0.5698
Breast: 1, 0.0594, 0.1302, 0.1002, 0.1828, 0.0394, 0.0132, 0.2734
Lung: 1, 0.1486, 0.324, 0.2636, 0.0674, 0.0044, 0.5362
Ovary: 1, 0.5298, 0.679, 0.1048, 0.0384, 0.4614
Spleen: 1, 0.6682, 0.3732, 0.0366, 0.7784
Adrenal: 1, 0.1028, 0.079, 0.4432
Heart: 1, 0.005, 0.7636
Duodenum: 1, 0.1222
Liver: 1
Placenta
Cortex
Testis
Thymus
Kidney
Breast
Lung
Ovary
Spleen
Adrenal
Heart
Duodenum
Liver

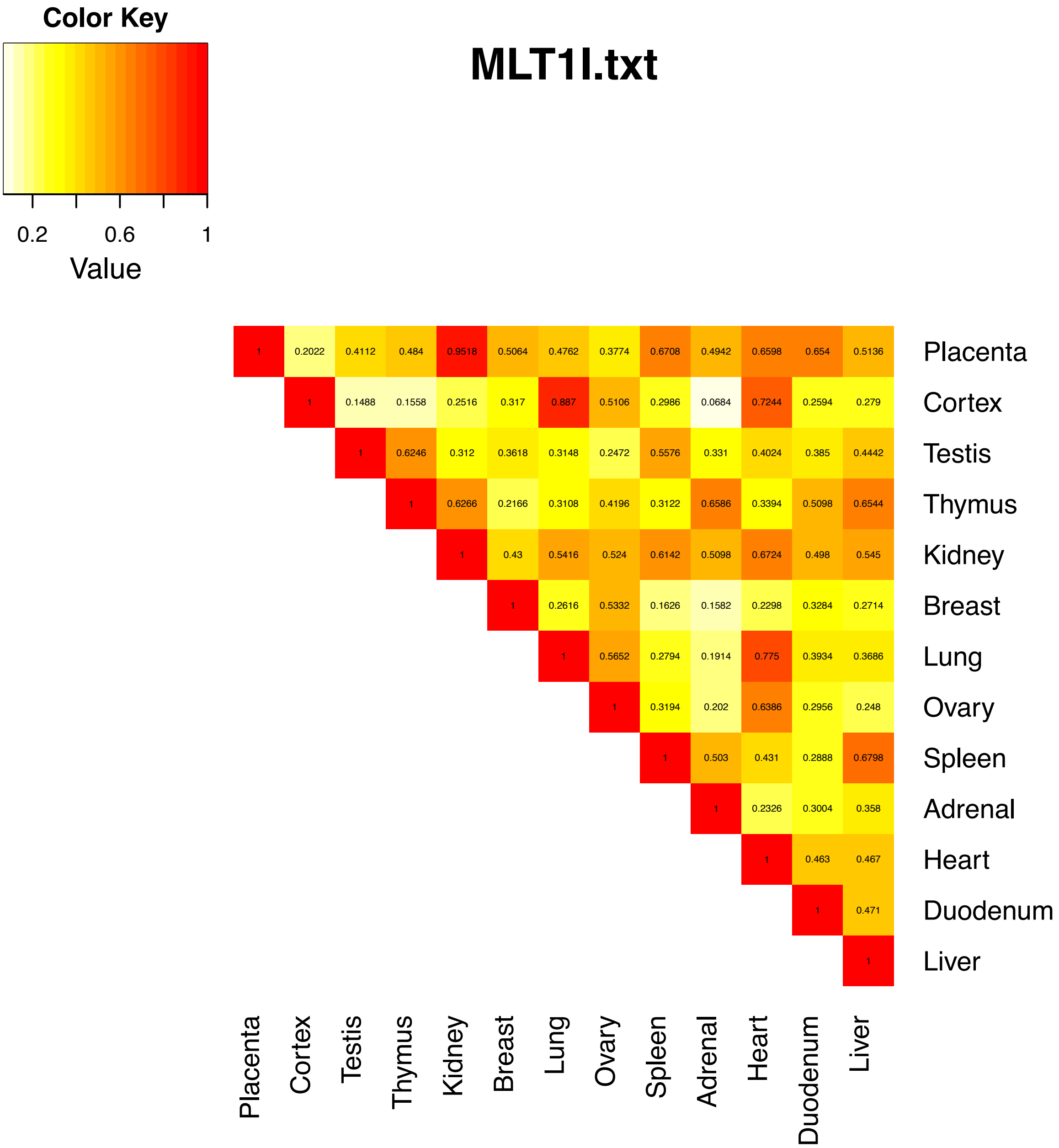
Color Key
0.2
0.6
1
Value
MLT1I.txt
Placenta: 1, 0.2022, 0.4112, 0.484, 0.9518, 0.5064, 0.4762, 0.3774, 0.6708, 0.4942, 0.6598, 0.654, 0.5136
Cortex: 1, 0.1488, 0.1558, 0.2516, 0.317, 0.887, 0.5106, 0.2986, 0.0684, 0.7244, 0.2594, 0.279
Testis: 1, 0.6246, 0.312, 0.3618, 0.3148, 0.2472, 0.5576, 0.331, 0.4024, 0.385, 0.4442
Thymus: 1, 0.6266, 0.2166, 0.3108, 0.4196, 0.3122, 0.6586, 0.3394, 0.5098, 0.6544
Kidney: 1, 0.43, 0.5416, 0.524, 0.6142, 0.5098, 0.6724, 0.498, 0.545
Breast: 1, 0.2616, 0.5332, 0.1626, 0.1582, 0.2298, 0.3284, 0.2714
Lung: 1, 0.5652, 0.2794, 0.1914, 0.775, 0.3934, 0.3686
Ovary: 1, 0.3194, 0.202, 0.6386, 0.2956, 0.248
Spleen: 1, 0.503, 0.431, 0.2888, 0.6798
Adrenal: 1, 0.2326, 0.3004, 0.358
Heart: 1, 0.463, 0.467
Duodenum: 1, 0.471
Liver: 1
Placenta
Cortex
Testis
Thymus
Kidney
Breast
Lung
Ovary
Spleen
Adrenal
Heart
Duodenum
Liver

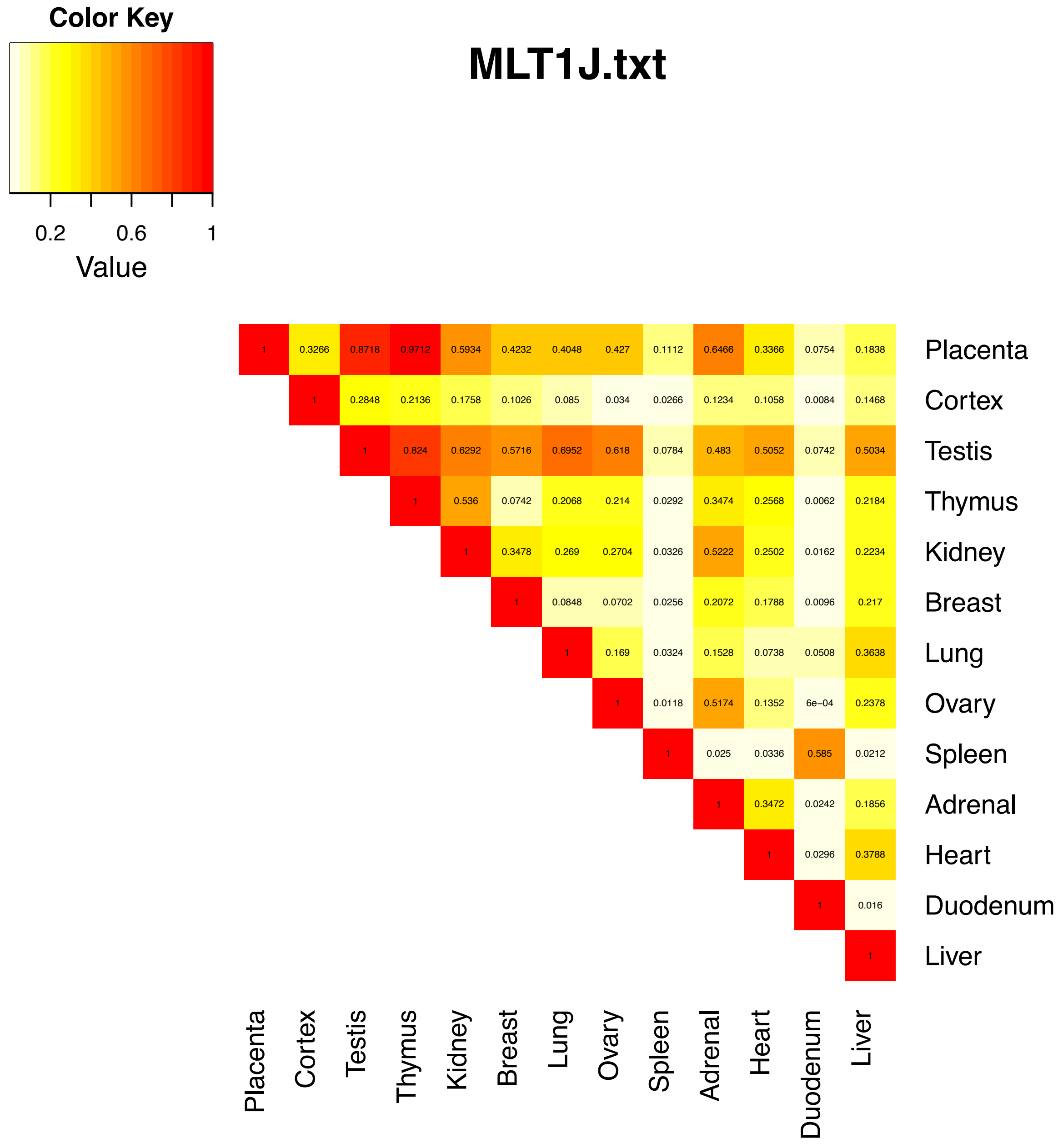
Color Key
0.2
0.6
1
Value
MLT1J.txt
Placenta: 1, 0.3266, 0.8718, 0.9712, 0.5934, 0.4232, 0.4048, 0.427, 0.1112, 0.6466, 0.3366, 0.0754, 0.1838
Cortex: 1, 0.2848, 0.2136, 0.1758, 0.1026, 0.085, 0.034, 0.0266, 0.1234, 0.1058, 0.0084, 0.1468
Testis: 1, 0.824, 0.6292, 0.5716, 0.6952, 0.618, 0.0784, 0.483, 0.5052, 0.0742, 0.5034
Thymus: 1, 0.536, 0.0742, 0.2068, 0.214, 0.0292, 0.3474, 0.2568, 0.0062, 0.2184
Kidney: 1, 0.3478, 0.269, 0.2704, 0.0326, 0.5222, 0.2502, 0.0162, 0.2234
Breast: 1, 0.0848, 0.0702, 0.0256, 0.2072, 0.1788, 0.0096, 0.217
Lung: 1, 0.169, 0.0324, 0.1528, 0.0738, 0.0508, 0.3638
Ovary: 1, 0.0118, 0.5174, 0.1352, 6e−04, 0.2378
Spleen: 1, 0.025, 0.0336, 0.585, 0.0212
Adrenal: 1, 0.3472, 0.0242, 0.1856
Heart: 1, 0.0296, 0.3788
Duodenum: 1, 0.016
Liver: 1
Placenta
Cortex
Testis
Thymus
Kidney
Breast
Lung
Ovary
Spleen
Adrenal
Heart
Duodenum
Liver

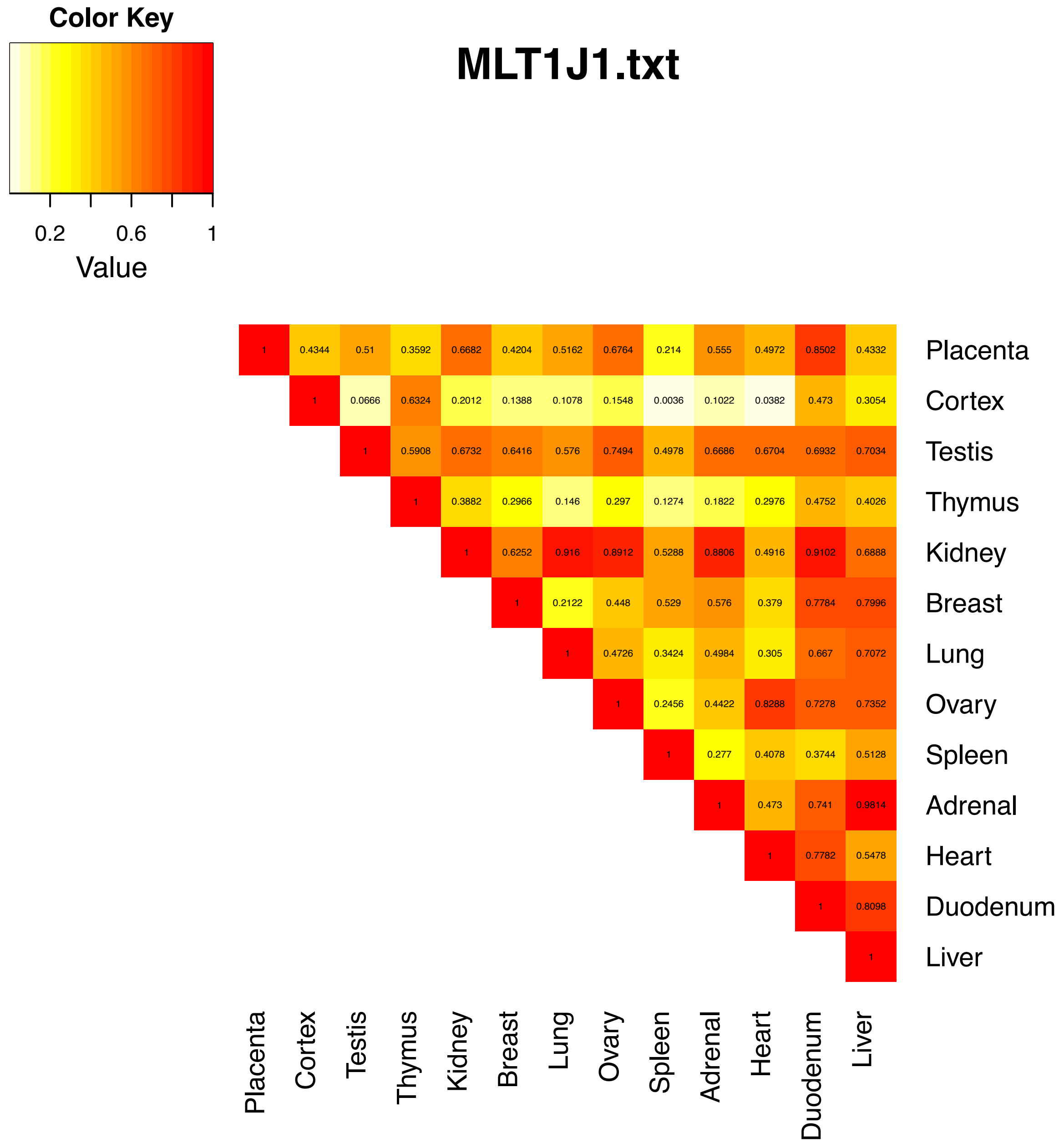
Color Key
0.2
0.6
1
Value
MLT1J1.txt
1 0.4344 0.51 0.3592 0.6682 0.4204 0.5162 0.6764 0.214 0.555 0.4972 0.8502 0.4332 Placenta
1 0.0666 0.6324 0.2012 0.1388 0.1078 0.1548 0.0036 0.1022 0.0382 0.473 0.3054 Cortex
1 0.5908 0.6732 0.6416 0.576 0.7494 0.4978 0.6686 0.6704 0.6932 0.7034 Testis
1 0.3882 0.2966 0.146 0.297 0.1274 0.1822 0.2976 0.4752 0.4026 Thymus
1 0.6252 0.916 0.8912 0.5288 0.8806 0.4916 0.9102 0.6888 Kidney
1 0.2122 0.448 0.529 0.576 0.379 0.7784 0.7996 Breast
1 0.4726 0.3424 0.4984 0.305 0.667 0.7072 Lung
1 0.2456 0.4422 0.8288 0.7278 0.7352 Ovary
1 0.277 0.4078 0.3744 0.5128 Spleen
1 0.473 0.741 0.9814 Adrenal
1 0.7782 0.5478 Heart
1 0.8098 Duodenum
1 Liver
Placenta
Cortex
Testis
Thymus
Kidney
Breast
Lung
Ovary
Spleen
Adrenal
Heart
Duodenum
Liver

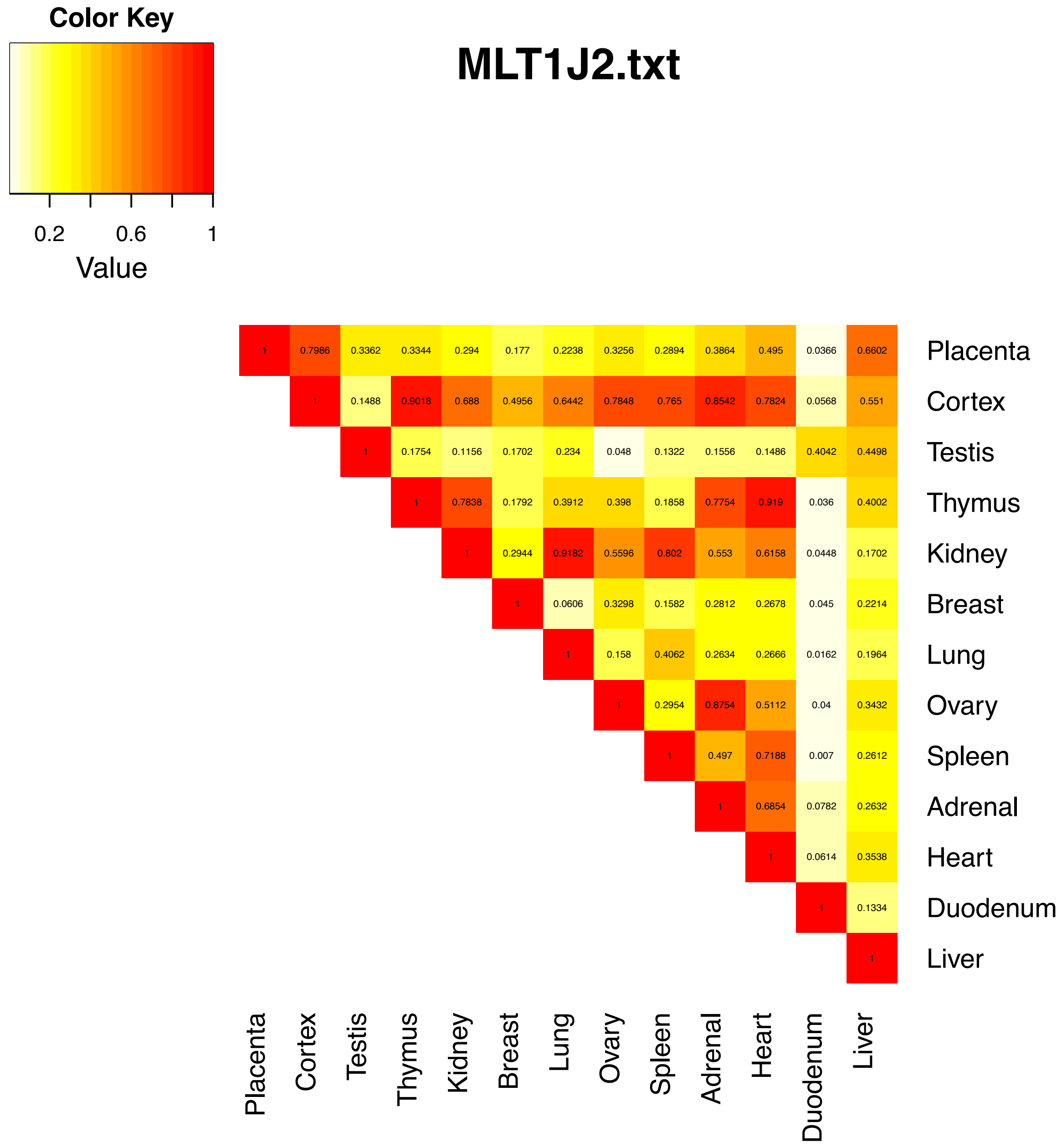

Color Key
0.2
0.6
1
Value
MLT1J2.txt
Placenta
Cortex
Testis
Thymus
Kidney
Breast
Lung
Ovary
Spleen
Adrenal
Heart
Duodenum
Liver
1 0.7986 0.3362 0.3344 0.294 0.177 0.2238 0.3256 0.2894 0.3864 0.495 0.0366 0.6602
1 0.1488 0.9018 0.688 0.4956 0.6442 0.7848 0.765 0.8542 0.7824 0.0568 0.551
1 0.1754 0.1156 0.1702 0.234 0.048 0.1322 0.1556 0.1486 0.4042 0.4498
1 0.7838 0.1792 0.3912 0.398 0.1858 0.7754 0.919 0.036 0.4002
1 0.2944 0.9182 0.5596 0.802 0.553 0.6158 0.0448 0.1702
1 0.0606 0.3298 0.1582 0.2812 0.2678 0.045 0.2214
1 0.158 0.4062 0.2634 0.2666 0.0162 0.1964
1 0.2954 0.8754 0.5112 0.04 0.3432
1 0.497 0.7188 0.007 0.2612
1 0.6854 0.0782 0.2632
1 0.0614 0.3538
1 0.1334
1

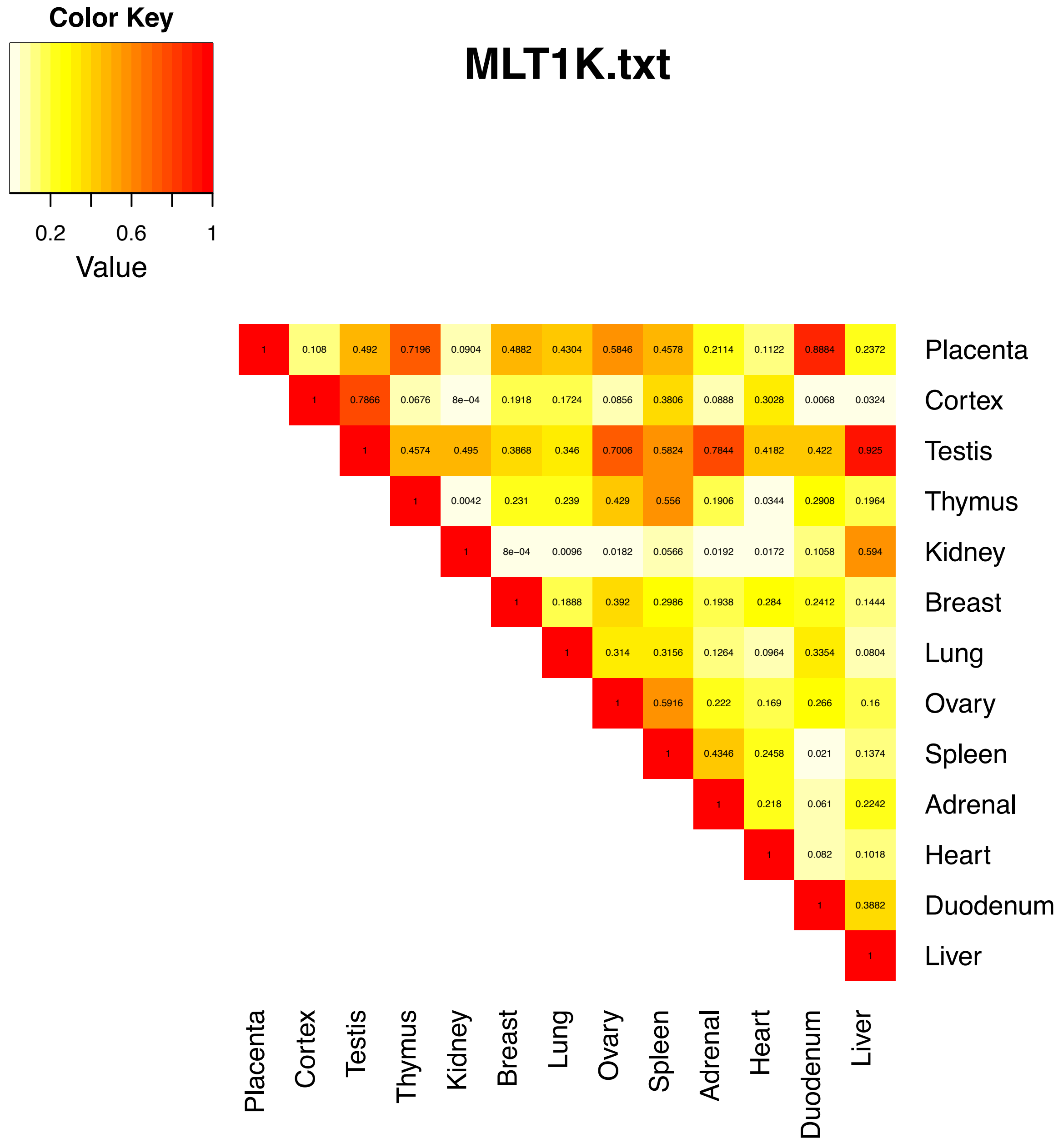
Color Key
0.2
0.6
1
Value
MLT1K.txt
1 0.108 0.492 0.7196 0.0904 0.4882 0.4304 0.5846 0.4578 0.2114 0.1122 0.8884 0.2372
1 0.7866 0.0676 8e−04 0.1918 0.1724 0.0856 0.3806 0.0888 0.3028 0.0068 0.0324
1 0.4574 0.495 0.3868 0.346 0.7006 0.5824 0.7844 0.4182 0.422 0.925
1 0.0042 0.231 0.239 0.429 0.556 0.1906 0.0344 0.2908 0.1964
1 8e−04 0.0096 0.0182 0.0566 0.0192 0.0172 0.1058 0.594
1 0.1888 0.392 0.2986 0.1938 0.284 0.2412 0.1444
1 0.314 0.3156 0.1264 0.0964 0.3354 0.0804
1 0.5916 0.222 0.169 0.266 0.16
1 0.4346 0.2458 0.021 0.1374
1 0.218 0.061 0.2242
1 0.082 0.1018
1 0.3882
1
Placenta
Cortex
Testis
Thymus
Kidney
Breast
Lung
Ovary
Spleen
Adrenal
Heart
Duodenum
Liver
Placenta
Cortex
Testis
Thymus
Kidney
Breast
Lung
Ovary
Spleen
Adrenal
Heart
Duodenum
Liver

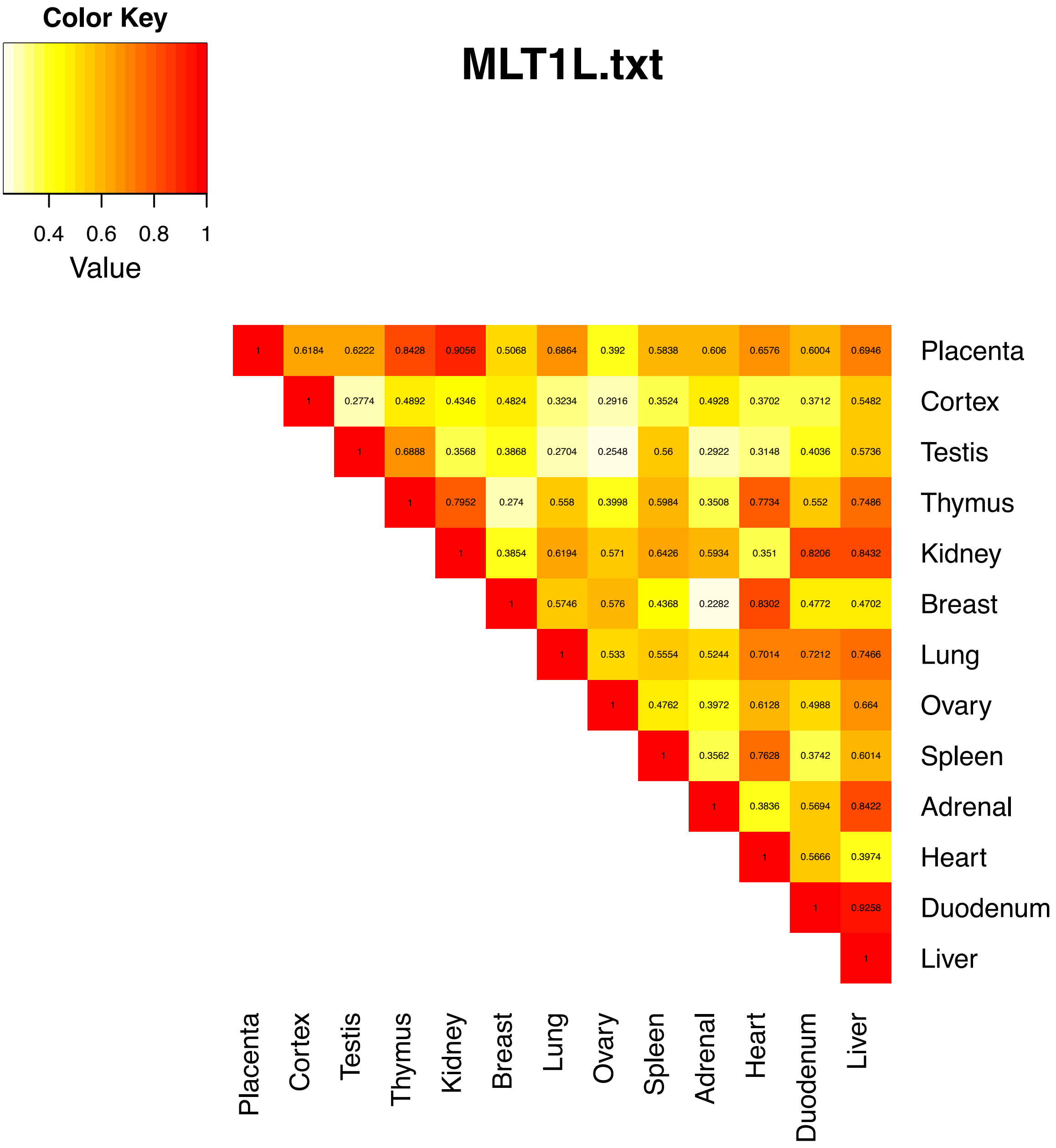
Color Key
0.4 0.6 0.8 1
Value
MLT1L.txt
Placenta: 1 0.6184 0.6222 0.8428 0.9056 0.5068 0.6864 0.392 0.5838 0.606 0.6576 0.6004 0.6946
Cortex: 1 0.2774 0.4892 0.4346 0.4824 0.3234 0.2916 0.3524 0.4928 0.3702 0.3712 0.5482
Testis: 1 0.6888 0.3568 0.3868 0.2704 0.2548 0.56 0.2922 0.3148 0.4036 0.5736
Thymus: 1 0.7952 0.274 0.558 0.3998 0.5984 0.3508 0.7734 0.552 0.7486
Kidney: 1 0.3854 0.6194 0.571 0.6426 0.5934 0.351 0.8206 0.8432
Breast: 1 0.5746 0.576 0.4368 0.2282 0.8302 0.4772 0.4702
Lung: 1 0.533 0.5554 0.5244 0.7014 0.7212 0.7466
Ovary: 1 0.4762 0.3972 0.6128 0.4988 0.664
Spleen: 1 0.3562 0.7628 0.3742 0.6014
Adrenal: 1 0.3836 0.5694 0.8422
Heart: 1 0.5666 0.3974
Duodenum: 1 0.9258
Liver: 1
Placenta Cortex Testis Thymus Kidney Breast Lung Ovary Spleen Adrenal Heart Duodenum Liver

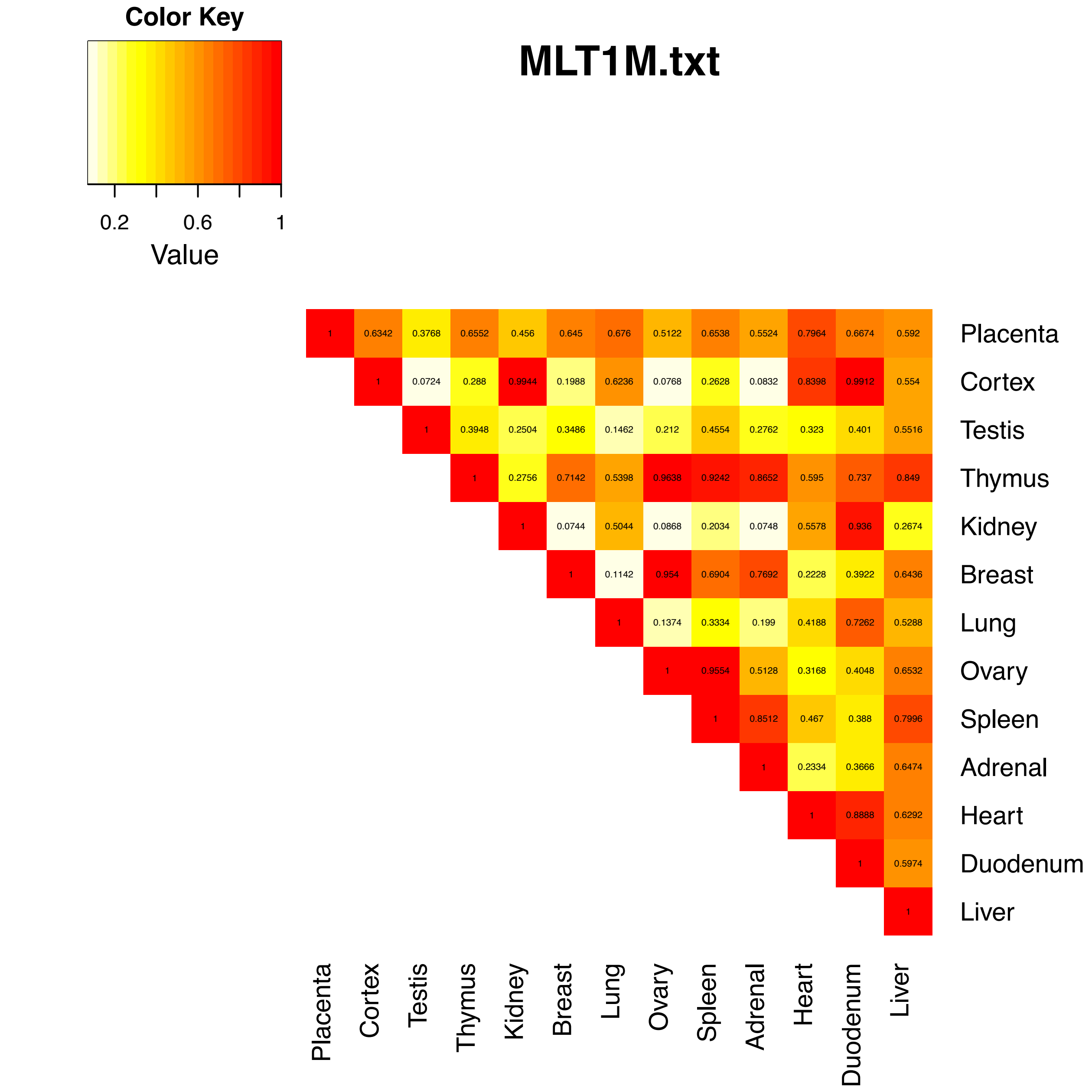
Color Key
0.2
0.6
1
Value
MLT1M.txt
Placenta 1 0.6342 0.3768 0.6552 0.456 0.645 0.676 0.5122 0.6538 0.5524 0.7964 0.6674 0.592
Cortex 1 0.0724 0.288 0.9944 0.1988 0.6236 0.0768 0.2628 0.0832 0.8398 0.9912 0.554
Testis 1 0.3948 0.2504 0.3486 0.1462 0.212 0.4554 0.2762 0.323 0.401 0.5516
Thymus 1 0.2756 0.7142 0.5398 0.9638 0.9242 0.8652 0.595 0.737 0.849
Kidney 1 0.0744 0.5044 0.0868 0.2034 0.0748 0.5578 0.936 0.2674
Breast 1 0.1142 0.954 0.6904 0.7692 0.2228 0.3922 0.6436
Lung 1 0.1374 0.3334 0.199 0.4188 0.7262 0.5288
Ovary 1 0.9554 0.5128 0.3168 0.4048 0.6532
Spleen 1 0.8512 0.467 0.388 0.7996
Adrenal 1 0.2334 0.3666 0.6474
Heart 1 0.8888 0.6292
Duodenum 1 0.5974
Liver 1
Placenta
Cortex
Testis
Thymus
Kidney
Breast
Lung
Ovary
Spleen
Adrenal
Heart
Duodenum
Liver

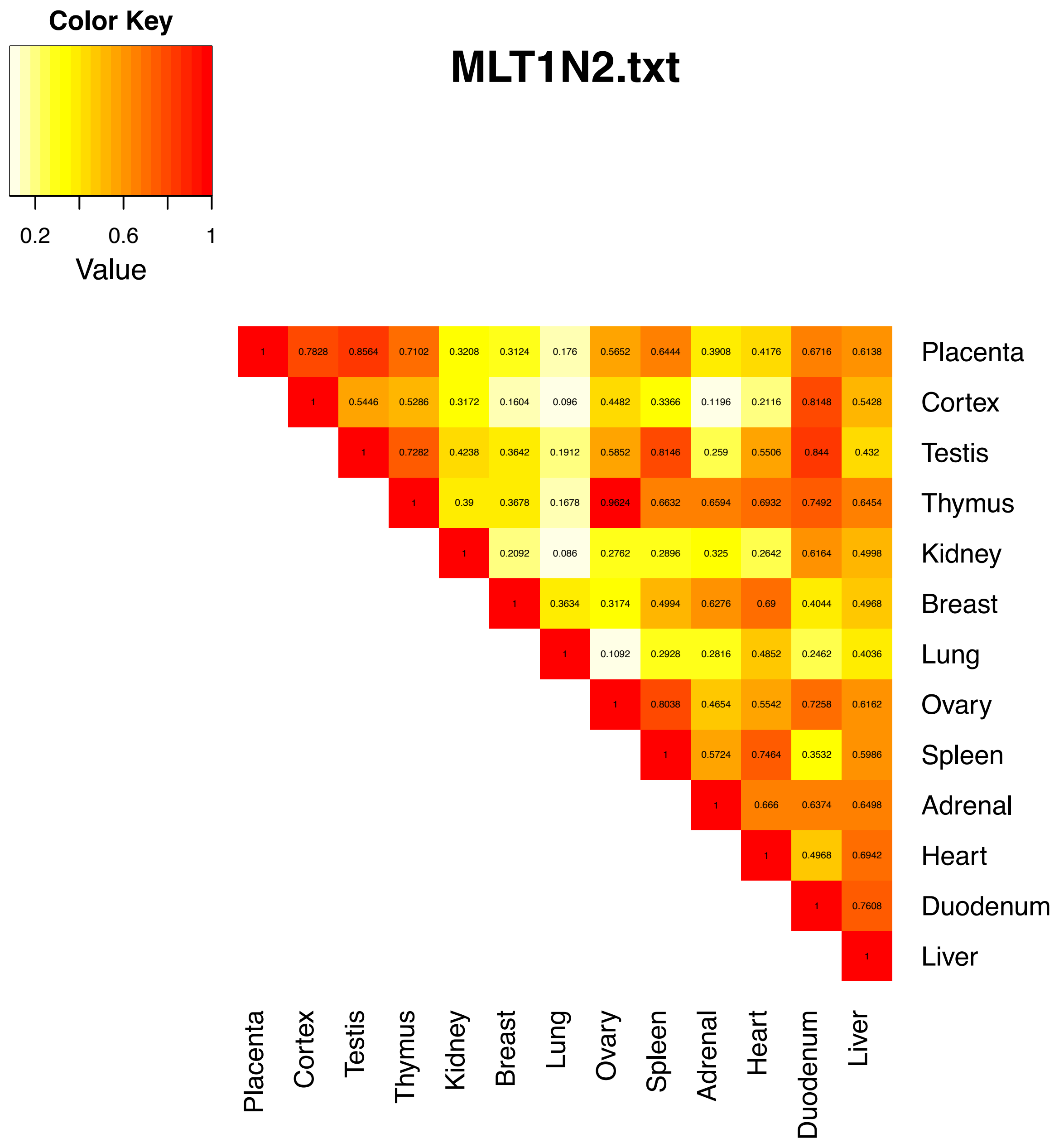

Color Key
0.2
0.6
1
Value
MLT1N2.txt
Placenta
Cortex
Testis
Thymus
Kidney
Breast
Lung
Ovary
Spleen
Adrenal
Heart
Duodenum
Liver
1 0.7828 0.8564 0.7102 0.3208 0.3124 0.176 0.5652 0.6444 0.3908 0.4176 0.6716 0.6138
1 0.5446 0.5286 0.3172 0.1604 0.096 0.4482 0.3366 0.1196 0.2116 0.8148 0.5428
1 0.7282 0.4238 0.3642 0.1912 0.5852 0.8146 0.259 0.5506 0.844 0.432
1 0.39 0.3678 0.1678 0.9624 0.6632 0.6594 0.6932 0.7492 0.6454
1 0.2092 0.086 0.2762 0.2896 0.325 0.2642 0.6164 0.4998
1 0.3634 0.3174 0.4994 0.6276 0.69 0.4044 0.4968
1 0.1092 0.2928 0.2816 0.4852 0.2462 0.4036
1 0.8038 0.4654 0.5542 0.7258 0.6162
1 0.5724 0.7464 0.3532 0.5986
1 0.666 0.6374 0.6498
1 0.4968 0.6942
1 0.7608
1

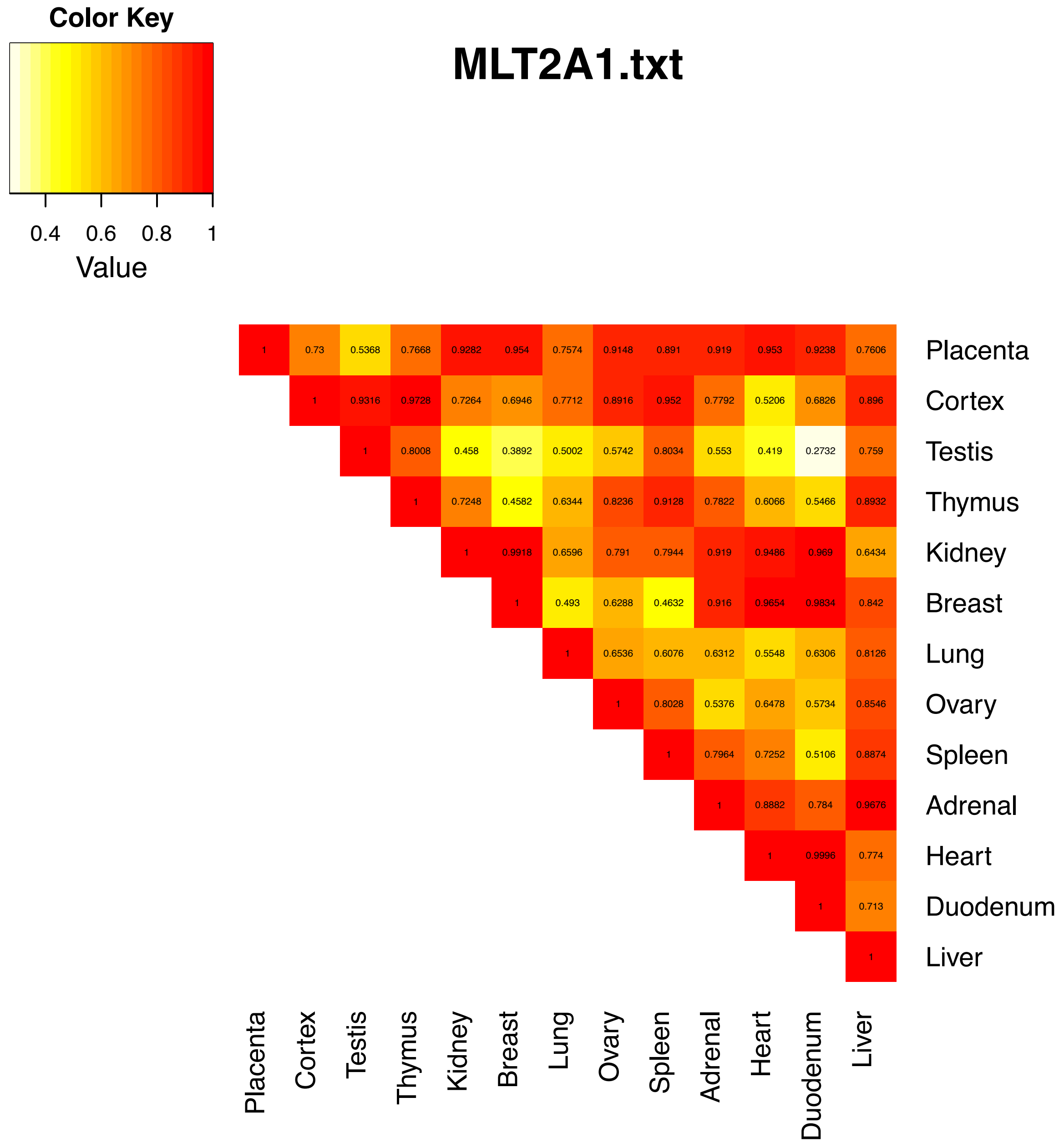
Color Key
0.4 0.6 0.8 1
Value
MLT2A1.txt
Placenta: 1 0.73 0.5368 0.7668 0.9282 0.954 0.7574 0.9148 0.891 0.919 0.953 0.9238 0.7606
Cortex: 1 0.9316 0.9728 0.7264 0.6946 0.7712 0.8916 0.952 0.7792 0.5206 0.6826 0.896
Testis: 1 0.8008 0.458 0.3892 0.5002 0.5742 0.8034 0.553 0.419 0.2732 0.759
Thymus: 1 0.7248 0.4582 0.6344 0.8236 0.9128 0.7822 0.6066 0.5466 0.8932
Kidney: 1 0.9918 0.6596 0.791 0.7944 0.919 0.9486 0.969 0.6434
Breast: 1 0.493 0.6288 0.4632 0.916 0.9654 0.9834 0.842
Lung: 1 0.6536 0.6076 0.6312 0.5548 0.6306 0.8126
Ovary: 1 0.8028 0.5376 0.6478 0.5734 0.8546
Spleen: 1 0.7964 0.7252 0.5106 0.8874
Adrenal: 1 0.8882 0.784 0.9676
Heart: 1 0.9996 0.774
Duodenum: 1 0.713
Liver: 1
Placenta Cortex Testis Thymus Kidney Breast Lung Ovary Spleen Adrenal Heart Duodenum Liver

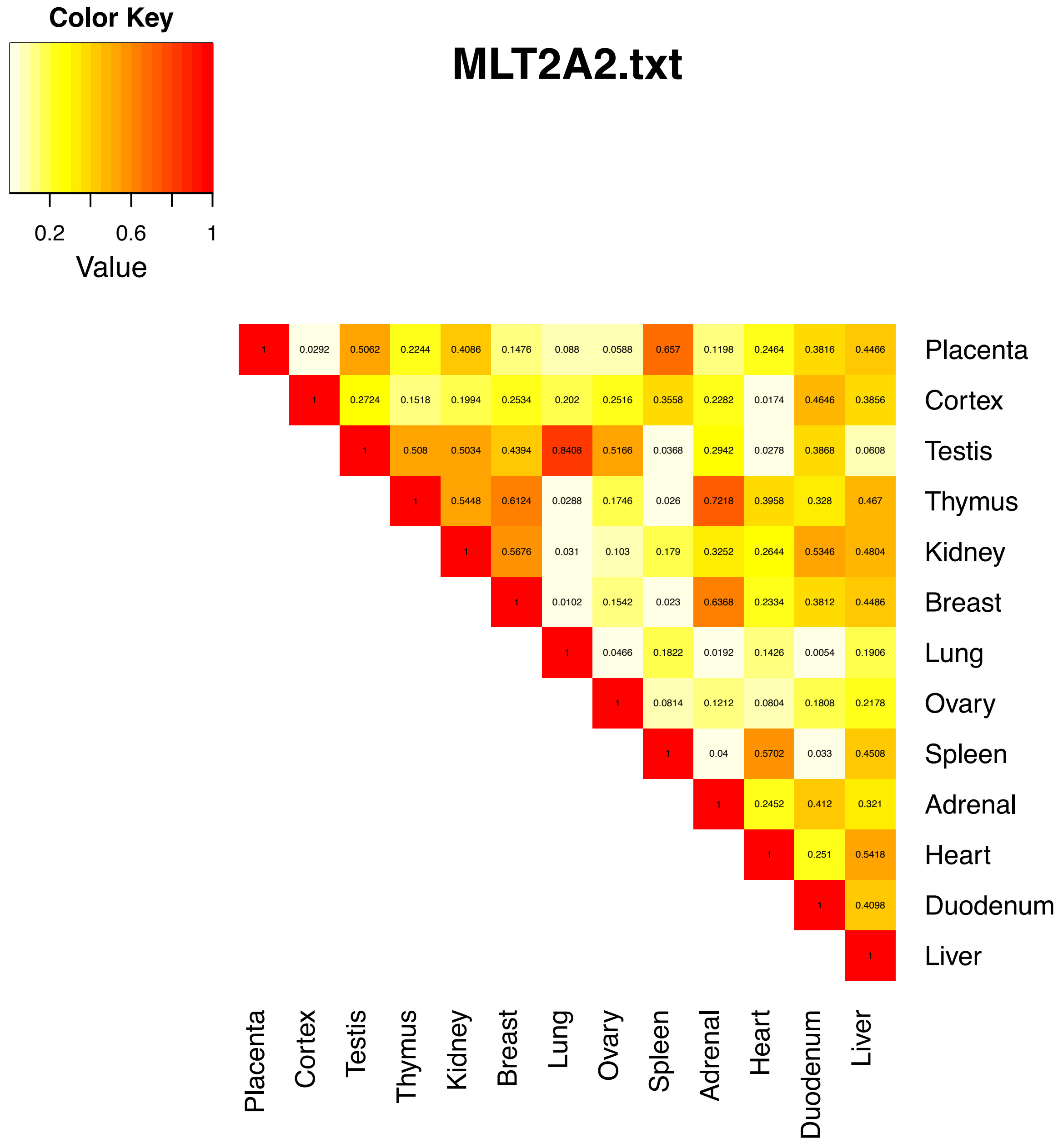
MLT2A2.txt
Color Key
0.2 0.6 1
Value
Placenta: 1 0.0292 0.5062 0.2244 0.4086 0.1476 0.088 0.0588 0.657 0.1198 0.2464 0.3816 0.4466
Cortex: 1 0.2724 0.1518 0.1994 0.2534 0.202 0.2516 0.3558 0.2282 0.0174 0.4646 0.3856
Testis: 1 0.508 0.5034 0.4394 0.8408 0.5166 0.0368 0.2942 0.0278 0.3868 0.0608
Thymus: 1 0.5448 0.6124 0.0288 0.1746 0.026 0.7218 0.3958 0.328 0.467
Kidney: 1 0.5676 0.031 0.103 0.179 0.3252 0.2644 0.5346 0.4804
Breast: 1 0.0102 0.1542 0.023 0.6368 0.2334 0.3812 0.4486
Lung: 1 0.0466 0.1822 0.0192 0.1426 0.0054 0.1906
Ovary: 1 0.0814 0.1212 0.0804 0.1808 0.2178
Spleen: 1 0.04 0.5702 0.033 0.4508
Adrenal: 1 0.2452 0.412 0.321
Heart: 1 0.251 0.5418
Duodenum: 1 0.4098
Liver: 1
Placenta Cortex Testis Thymus Kidney Breast Lung Ovary Spleen Adrenal Heart Duodenum Liver

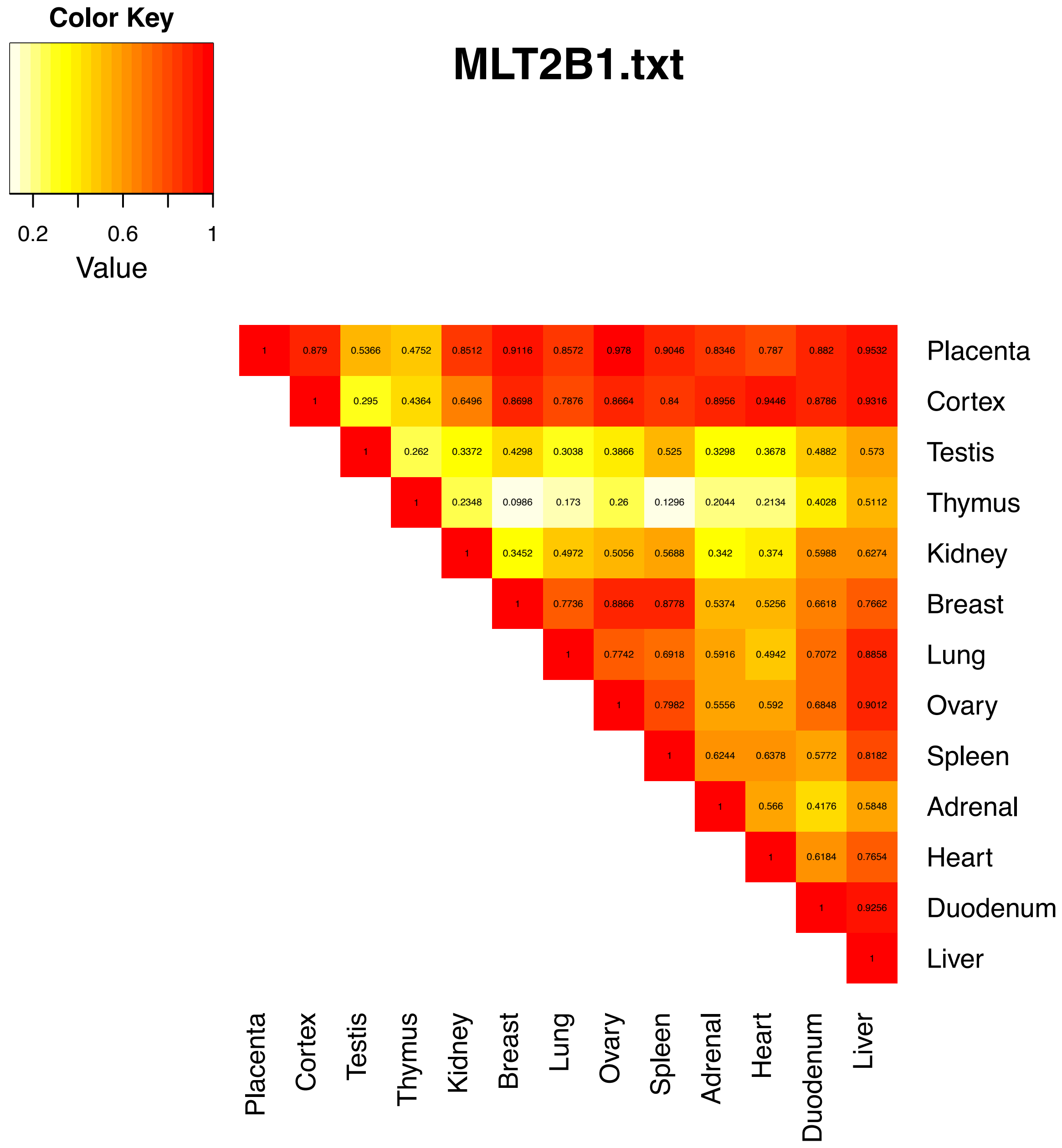
Color Key
0.2
0.6
1
Value
MLT2B1.txt
Placenta
Cortex
Testis
Thymus
Kidney
Breast
Lung
Ovary
Spleen
Adrenal
Heart
Duodenum
Liver
1 0.879 0.5366 0.4752 0.8512 0.9116 0.8572 0.978 0.9046 0.8346 0.787 0.882 0.9532
1 0.295 0.4364 0.6496 0.8698 0.7876 0.8664 0.84 0.8956 0.9446 0.8786 0.9316
1 0.262 0.3372 0.4298 0.3038 0.3866 0.525 0.3298 0.3678 0.4882 0.573
1 0.2348 0.0986 0.173 0.26 0.1296 0.2044 0.2134 0.4028 0.5112
1 0.3452 0.4972 0.5056 0.5688 0.342 0.374 0.5988 0.6274
1 0.7736 0.8866 0.8778 0.5374 0.5256 0.6618 0.7662
1 0.7742 0.6918 0.5916 0.4942 0.7072 0.8858
1 0.7982 0.5556 0.592 0.6848 0.9012
1 0.6244 0.6378 0.5772 0.8182
1 0.566 0.4176 0.5848
1 0.6184 0.7654
1 0.9256
1

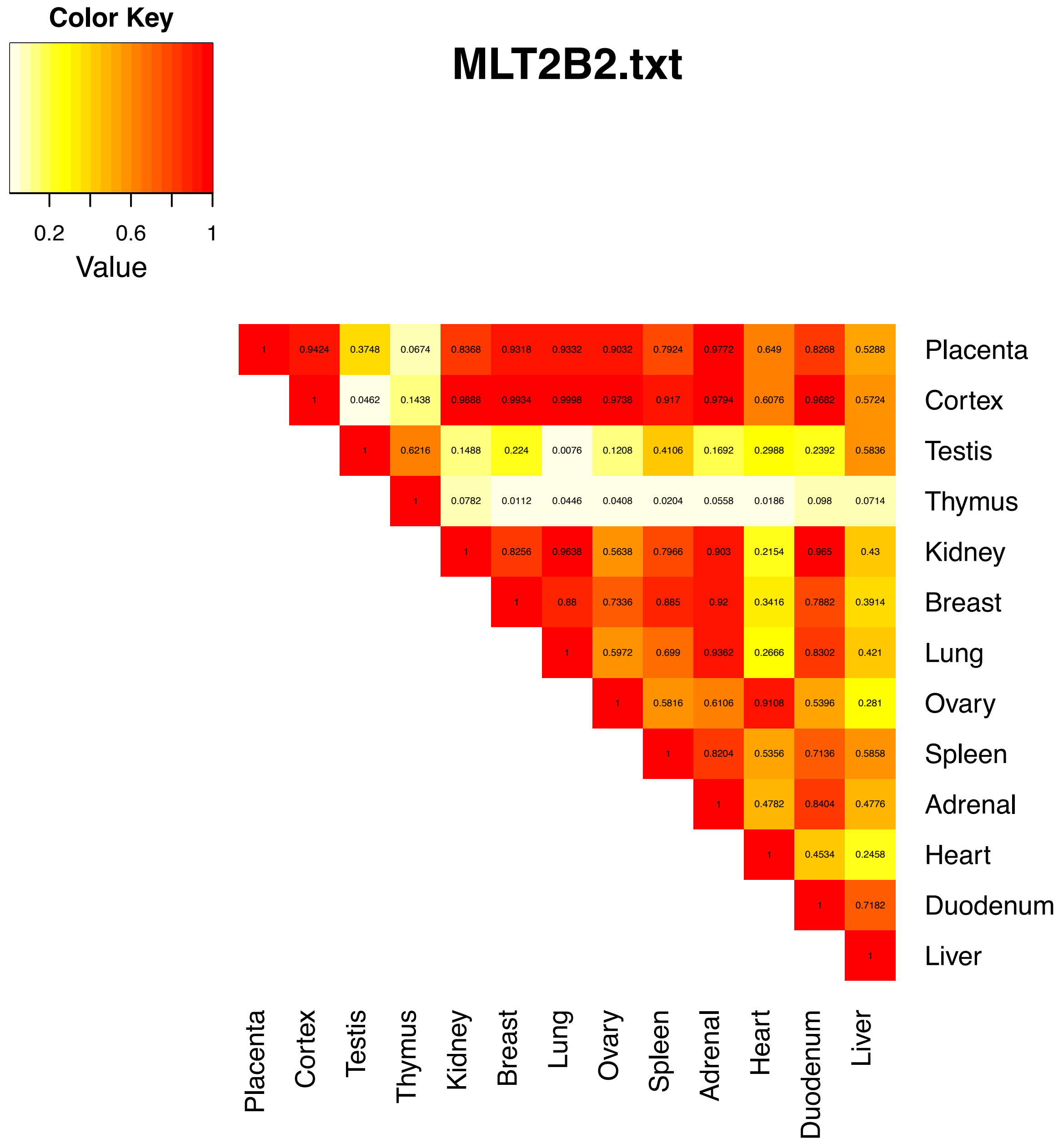
Color Key
0.2
0.6
1
Value
MLT2B2.txt
1 0.9424 0.3748 0.0674 0.8368 0.9318 0.9332 0.9032 0.7924 0.9772 0.649 0.8268 0.5288 Placenta
1 0.0462 0.1438 0.9888 0.9934 0.9998 0.9738 0.917 0.9794 0.6076 0.9682 0.5724 Cortex
1 0.6216 0.1488 0.224 0.0076 0.1208 0.4106 0.1692 0.2988 0.2392 0.5836 Testis
1 0.0782 0.0112 0.0446 0.0408 0.0204 0.0558 0.0186 0.098 0.0714 Thymus
1 0.8256 0.9638 0.5638 0.7966 0.903 0.2154 0.965 0.43 Kidney
1 0.88 0.7336 0.885 0.92 0.3416 0.7882 0.3914 Breast
1 0.5972 0.699 0.9362 0.2666 0.8302 0.421 Lung
1 0.5816 0.6106 0.9108 0.5396 0.281 Ovary
1 0.8204 0.5356 0.7136 0.5858 Spleen
1 0.4782 0.8404 0.4776 Adrenal
1 0.4534 0.2458 Heart
1 0.7182 Duodenum
1 Liver
Placenta
Cortex
Testis
Thymus
Kidney
Breast
Lung
Ovary
Spleen
Adrenal
Heart
Duodenum
Liver

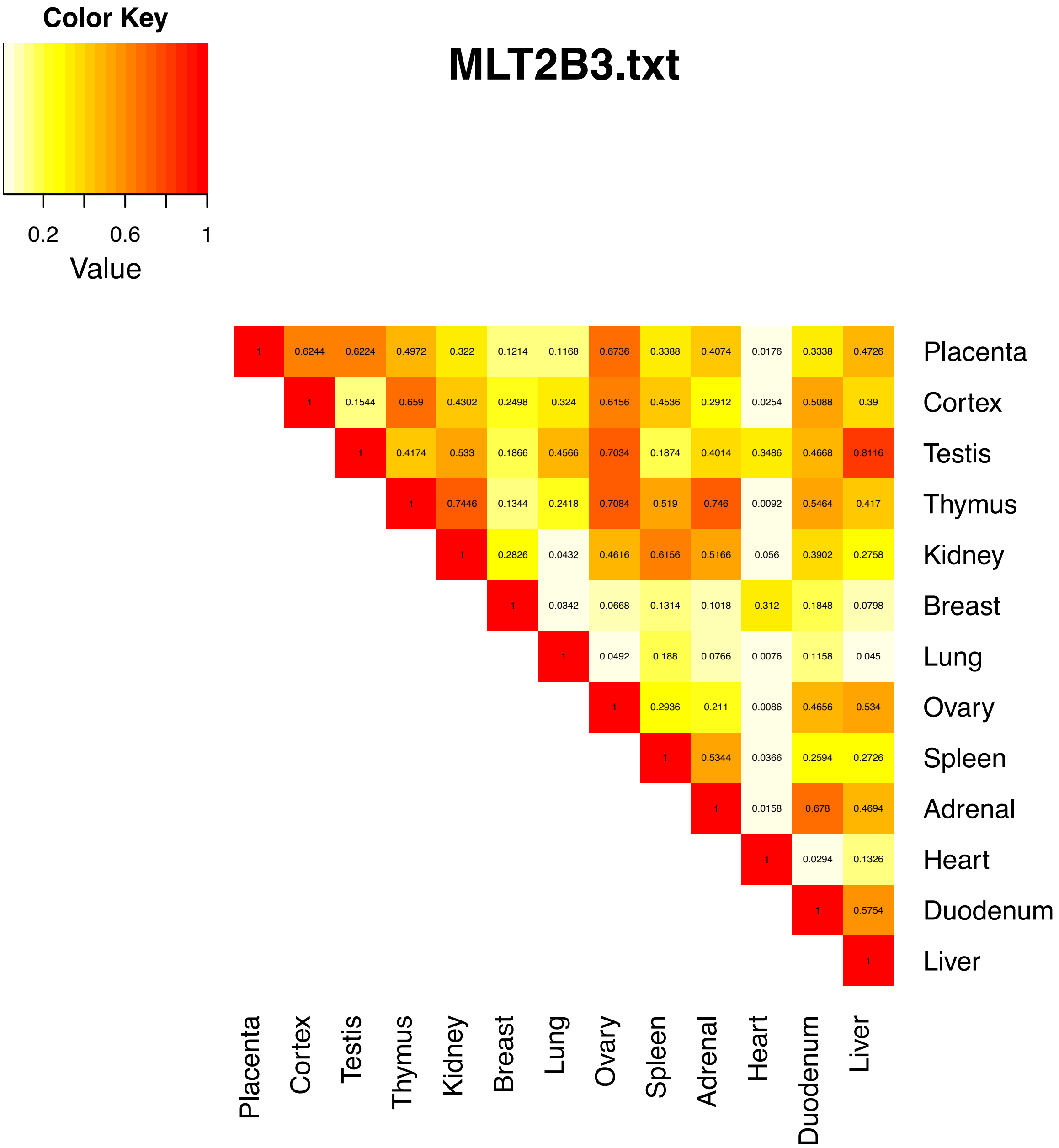
Color Key
0.2 0.6 1
Value
MLT2B3.txt
Placenta: 1 0.6244 0.6224 0.4972 0.322 0.1214 0.1168 0.6736 0.3388 0.4074 0.0176 0.3338 0.4726
Cortex: 1 0.1544 0.659 0.4302 0.2498 0.324 0.6156 0.4536 0.2912 0.0254 0.5088 0.39
Testis: 1 0.4174 0.533 0.1866 0.4566 0.7034 0.1874 0.4014 0.3486 0.4668 0.8116
Thymus: 1 0.7446 0.1344 0.2418 0.7084 0.519 0.746 0.0092 0.5464 0.417
Kidney: 1 0.2826 0.0432 0.4616 0.6156 0.5166 0.056 0.3902 0.2758
Breast: 1 0.0342 0.0668 0.1314 0.1018 0.312 0.1848 0.0798
Lung: 1 0.0492 0.188 0.0766 0.0076 0.1158 0.045
Ovary: 1 0.2936 0.211 0.0086 0.4656 0.534
Spleen: 1 0.5344 0.0366 0.2594 0.2726
Adrenal: 1 0.0158 0.678 0.4694
Heart: 1 0.0294 0.1326
Duodenum: 1 0.5754
Liver: 1
Placenta Cortex Testis Thymus Kidney Breast Lung Ovary Spleen Adrenal Heart Duodenum Liver

MLT2B4.txt

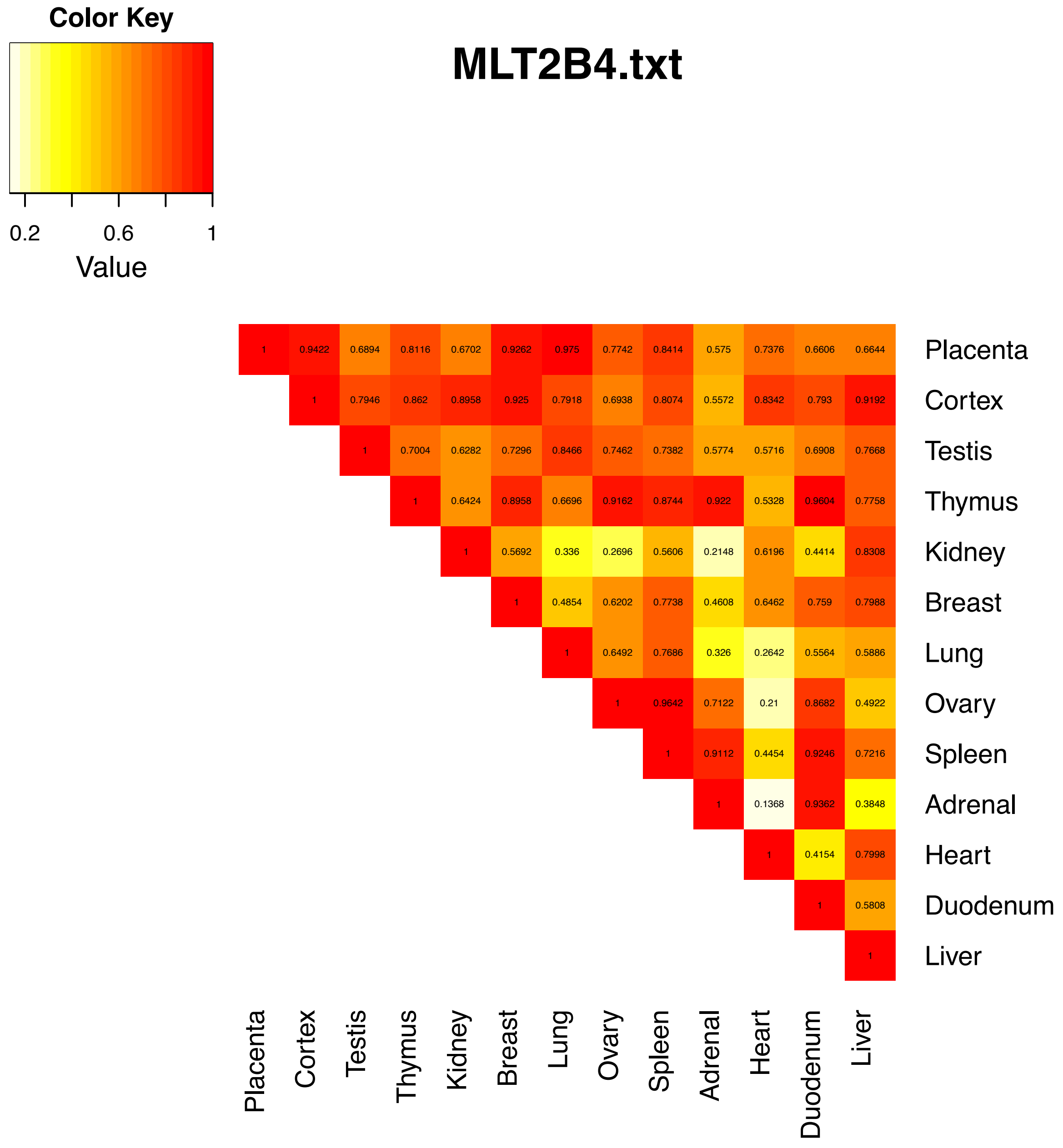

Color Key
0.2
0.6
1
Value
1 0.9422 0.6894 0.8116 0.6702 0.9262 0.975 0.7742 0.8414 0.575 0.7376 0.6606 0.6644 Placenta
1 0.7946 0.862 0.8958 0.925 0.7918 0.6938 0.8074 0.5572 0.8342 0.793 0.9192 Cortex
1 0.7004 0.6282 0.7296 0.8466 0.7462 0.7382 0.5774 0.5716 0.6908 0.7668 Testis
1 0.6424 0.8958 0.6696 0.9162 0.8744 0.922 0.5328 0.9604 0.7758 Thymus
1 0.5692 0.336 0.2696 0.5606 0.2148 0.6196 0.4414 0.8308 Kidney
1 0.4854 0.6202 0.7738 0.4608 0.6462 0.759 0.7988 Breast
1 0.6492 0.7686 0.326 0.2642 0.5564 0.5886 Lung
1 0.9642 0.7122 0.21 0.8682 0.4922 Ovary
1 0.9112 0.4454 0.9246 0.7216 Spleen
1 0.1368 0.9362 0.3848 Adrenal
1 0.4154 0.7998 Heart
1 0.5808 Duodenum
1 Liver
Placenta
Cortex
Testis
Thymus
Kidney
Breast
Lung
Ovary
Spleen
Adrenal
Heart
Duodenum
Liver

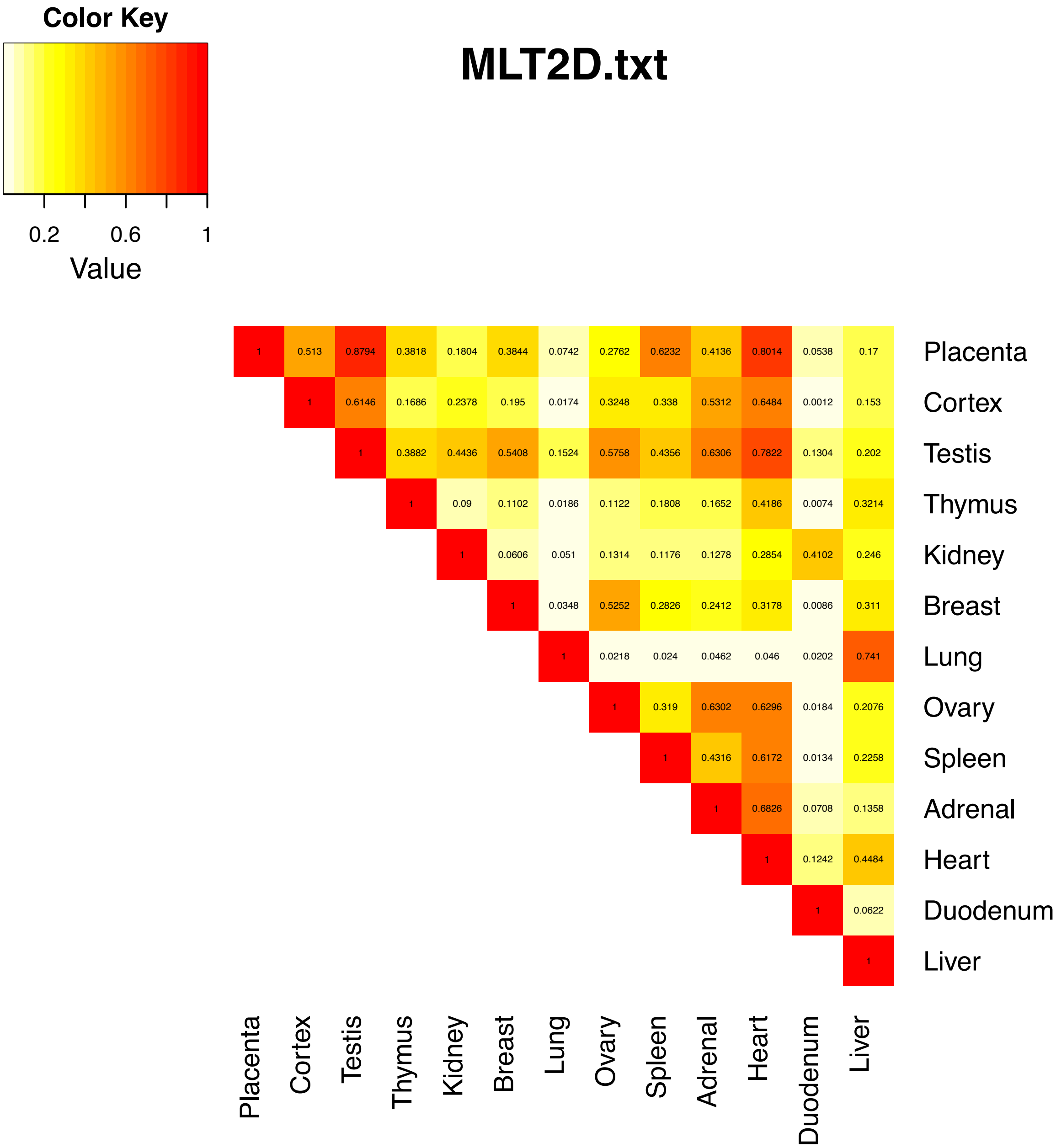

Color Key
0.2
0.6
1
Value
MLT2D.txt
Placenta
Cortex
Testis
Thymus
Kidney
Breast
Lung
Ovary
Spleen
Adrenal
Heart
Duodenum
Liver
1 0.513 0.8794 0.3818 0.1804 0.3844 0.0742 0.2762 0.6232 0.4136 0.8014 0.0538 0.17
1 0.6146 0.1686 0.2378 0.195 0.0174 0.3248 0.338 0.5312 0.6484 0.0012 0.153
1 0.3882 0.4436 0.5408 0.1524 0.5758 0.4356 0.6306 0.7822 0.1304 0.202
1 0.09 0.1102 0.0186 0.1122 0.1808 0.1652 0.4186 0.0074 0.3214
1 0.0606 0.051 0.1314 0.1176 0.1278 0.2854 0.4102 0.246
1 0.0348 0.5252 0.2826 0.2412 0.3178 0.0086 0.311
1 0.0218 0.024 0.0462 0.046 0.0202 0.741
1 0.319 0.6302 0.6296 0.0184 0.2076
1 0.4316 0.6172 0.0134 0.2258
1 0.6826 0.0708 0.1358
1 0.1242 0.4484
1 0.0622
1

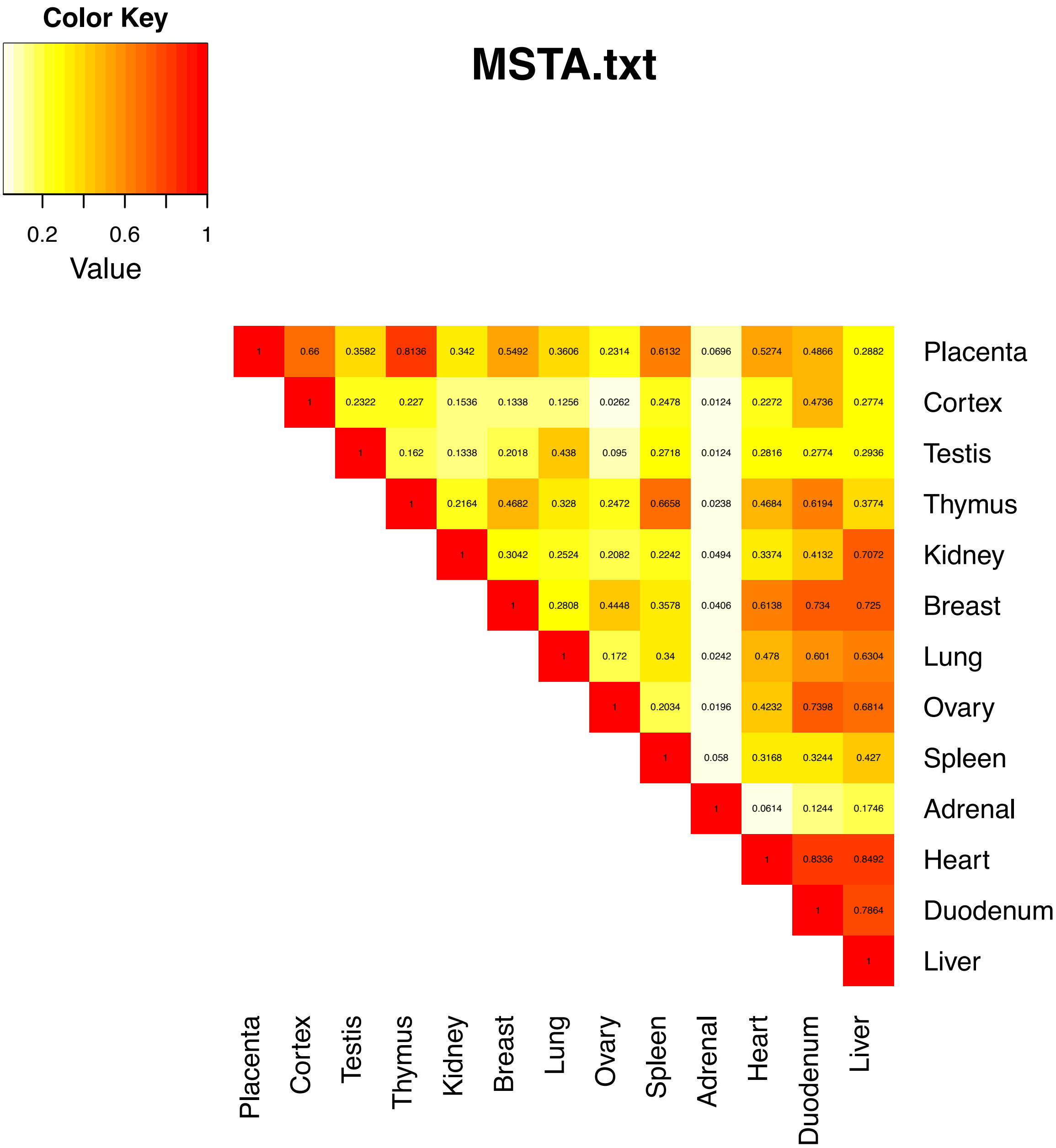
Color Key
0.2
0.6
1
Value
MSTA.txt
Placenta
Cortex
Testis
Thymus
Kidney
Breast
Lung
Ovary
Spleen
Adrenal
Heart
Duodenum
Liver
1 0.66 0.3582 0.8136 0.342 0.5492 0.3606 0.2314 0.6132 0.0696 0.5274 0.4866 0.2882
1 0.2322 0.227 0.1536 0.1338 0.1256 0.0262 0.2478 0.0124 0.2272 0.4736 0.2774
1 0.162 0.1338 0.2018 0.438 0.095 0.2718 0.0124 0.2816 0.2774 0.2936
1 0.2164 0.4682 0.328 0.2472 0.6658 0.0238 0.4684 0.6194 0.3774
1 0.3042 0.2524 0.2082 0.2242 0.0494 0.3374 0.4132 0.7072
1 0.2808 0.4448 0.3578 0.0406 0.6138 0.734 0.725
1 0.172 0.34 0.0242 0.478 0.601 0.6304
1 0.2034 0.0196 0.4232 0.7398 0.6814
1 0.058 0.3168 0.3244 0.427
1 0.0614 0.1244 0.1746
1 0.8336 0.8492
1 0.7864
1

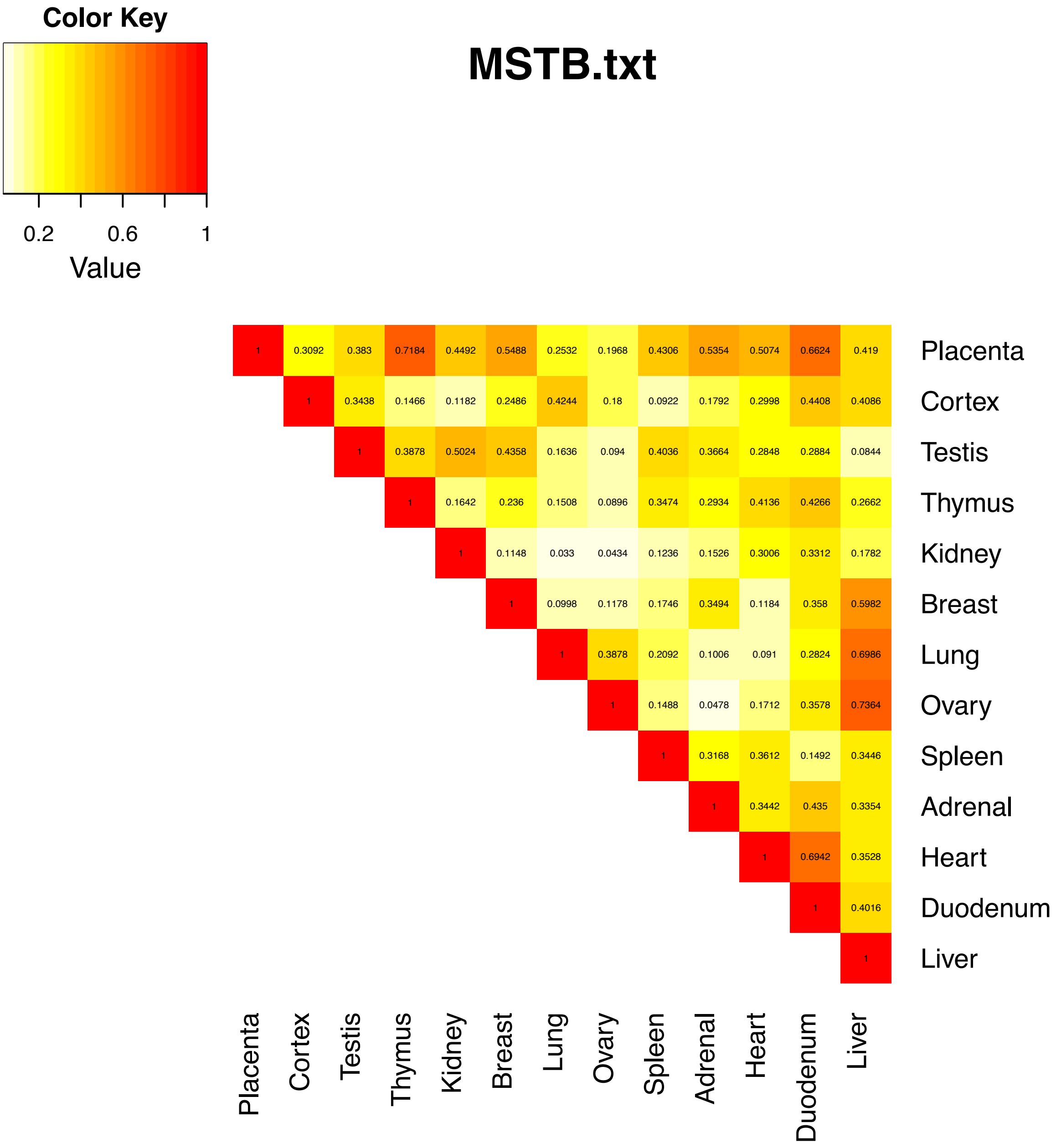
Color Key
0.2
0.6
1
Value
MSTB.txt
Placenta 1 0.3092 0.383 0.7184 0.4492 0.5488 0.2532 0.1968 0.4306 0.5354 0.5074 0.6624 0.419
Cortex 1 0.3438 0.1466 0.1182 0.2486 0.4244 0.18 0.0922 0.1792 0.2998 0.4408 0.4086
Testis 1 0.3878 0.5024 0.4358 0.1636 0.094 0.4036 0.3664 0.2848 0.2884 0.0844
Thymus 1 0.1642 0.236 0.1508 0.0896 0.3474 0.2934 0.4136 0.4266 0.2662
Kidney 1 0.1148 0.033 0.0434 0.1236 0.1526 0.3006 0.3312 0.1782
Breast 1 0.0998 0.1178 0.1746 0.3494 0.1184 0.358 0.5982
Lung 1 0.3878 0.2092 0.1006 0.091 0.2824 0.6986
Ovary 1 0.1488 0.0478 0.1712 0.3578 0.7364
Spleen 1 0.3168 0.3612 0.1492 0.3446
Adrenal 1 0.3442 0.435 0.3354
Heart 1 0.6942 0.3528
Duodenum 1 0.4016
Liver 1
Placenta
Cortex
Testis
Thymus
Kidney
Breast
Lung
Ovary
Spleen
Adrenal
Heart
Duodenum
Liver

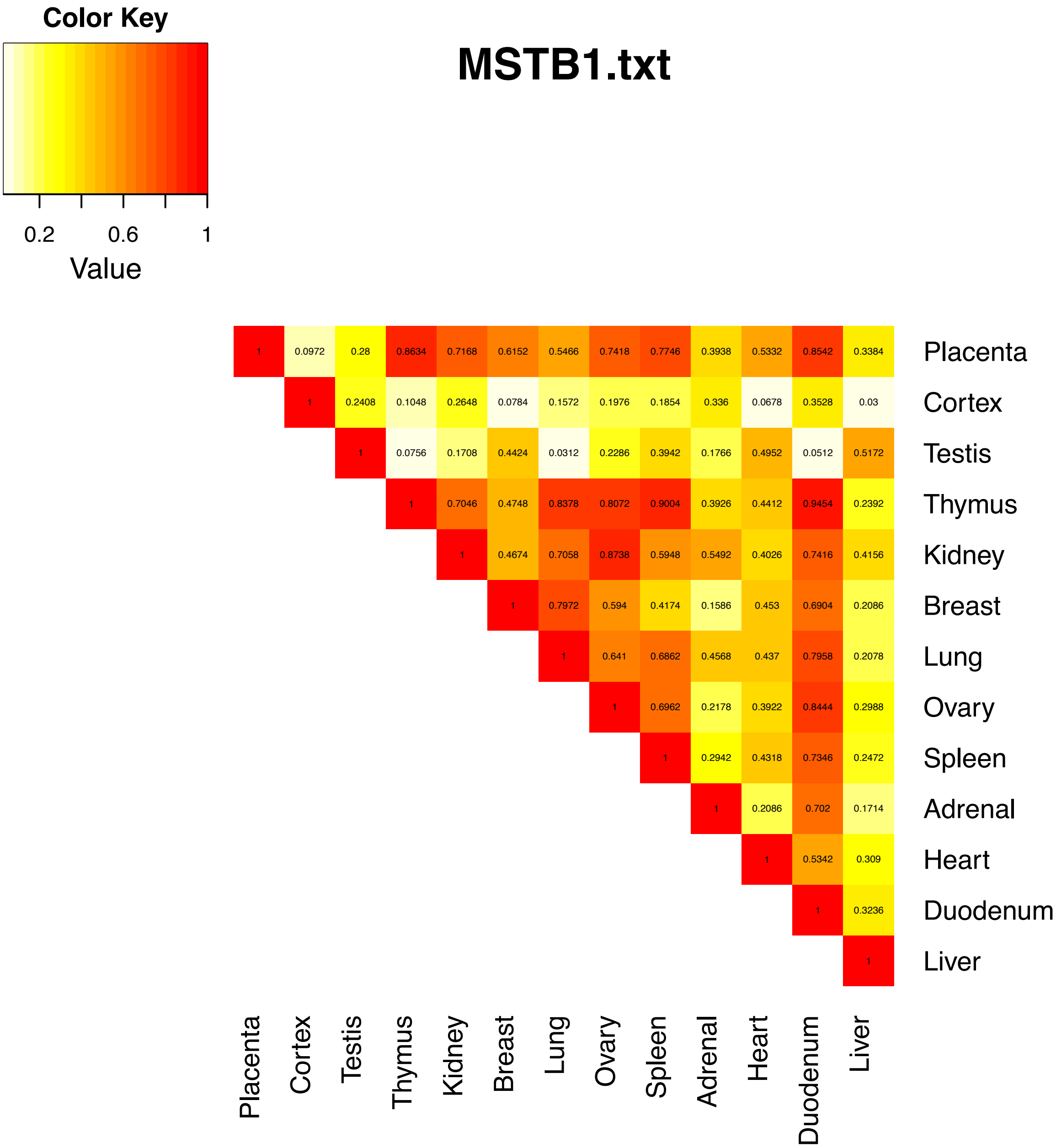
Color Key
0.2 0.6 1
Value
MSTB1.txt
Placenta 1 0.0972 0.28 0.8634 0.7168 0.6152 0.5466 0.7418 0.7746 0.3938 0.5332 0.8542 0.3384
Cortex 1 0.2408 0.1048 0.2648 0.0784 0.1572 0.1976 0.1854 0.336 0.0678 0.3528 0.03
Testis 1 0.0756 0.1708 0.4424 0.0312 0.2286 0.3942 0.1766 0.4952 0.0512 0.5172
Thymus 1 0.7046 0.4748 0.8378 0.8072 0.9004 0.3926 0.4412 0.9454 0.2392
Kidney 1 0.4674 0.7058 0.8738 0.5948 0.5492 0.4026 0.7416 0.4156
Breast 1 0.7972 0.594 0.4174 0.1586 0.453 0.6904 0.2086
Lung 1 0.641 0.6862 0.4568 0.437 0.7958 0.2078
Ovary 1 0.6962 0.2178 0.3922 0.8444 0.2988
Spleen 1 0.2942 0.4318 0.7346 0.2472
Adrenal 1 0.2086 0.702 0.1714
Heart 1 0.5342 0.309
Duodenum 1 0.3236
Liver 1
Placenta Cortex Testis Thymus Kidney Breast Lung Ovary Spleen Adrenal Heart Duodenum Liver

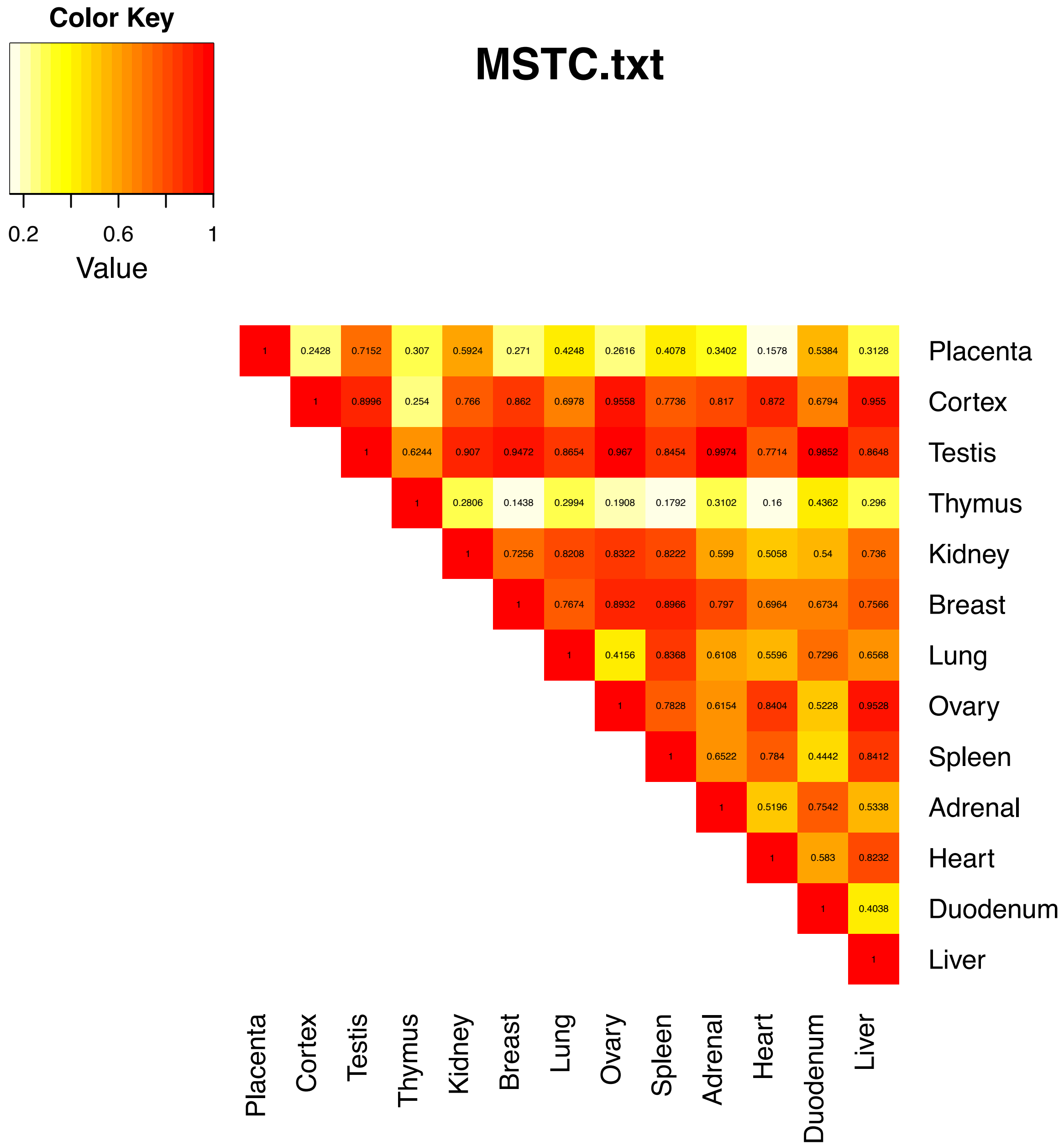

Color Key
0.2
0.6
1
Value
MSTC.txt
1 0.2428 0.7152 0.307 0.5924 0.271 0.4248 0.2616 0.4078 0.3402 0.1578 0.5384 0.3128
Placenta
1 0.8996 0.254 0.766 0.862 0.6978 0.9558 0.7736 0.817 0.872 0.6794 0.955
Cortex
1 0.6244 0.907 0.9472 0.8654 0.967 0.8454 0.9974 0.7714 0.9852 0.8648
Testis
1 0.2806 0.1438 0.2994 0.1908 0.1792 0.3102 0.16 0.4362 0.296
Thymus
1 0.7256 0.8208 0.8322 0.8222 0.599 0.5058 0.54 0.736
Kidney
1 0.7674 0.8932 0.8966 0.797 0.6964 0.6734 0.7566
Breast
1 0.4156 0.8368 0.6108 0.5596 0.7296 0.6568
Lung
1 0.7828 0.6154 0.8404 0.5228 0.9528
Ovary
1 0.6522 0.784 0.4442 0.8412
Spleen
1 0.5196 0.7542 0.5338
Adrenal
1 0.583 0.8232
Heart
1 0.4038
Duodenum
1
Liver
Placenta
Cortex
Testis
Thymus
Kidney
Breast
Lung
Ovary
Spleen
Adrenal
Heart
Duodenum
Liver

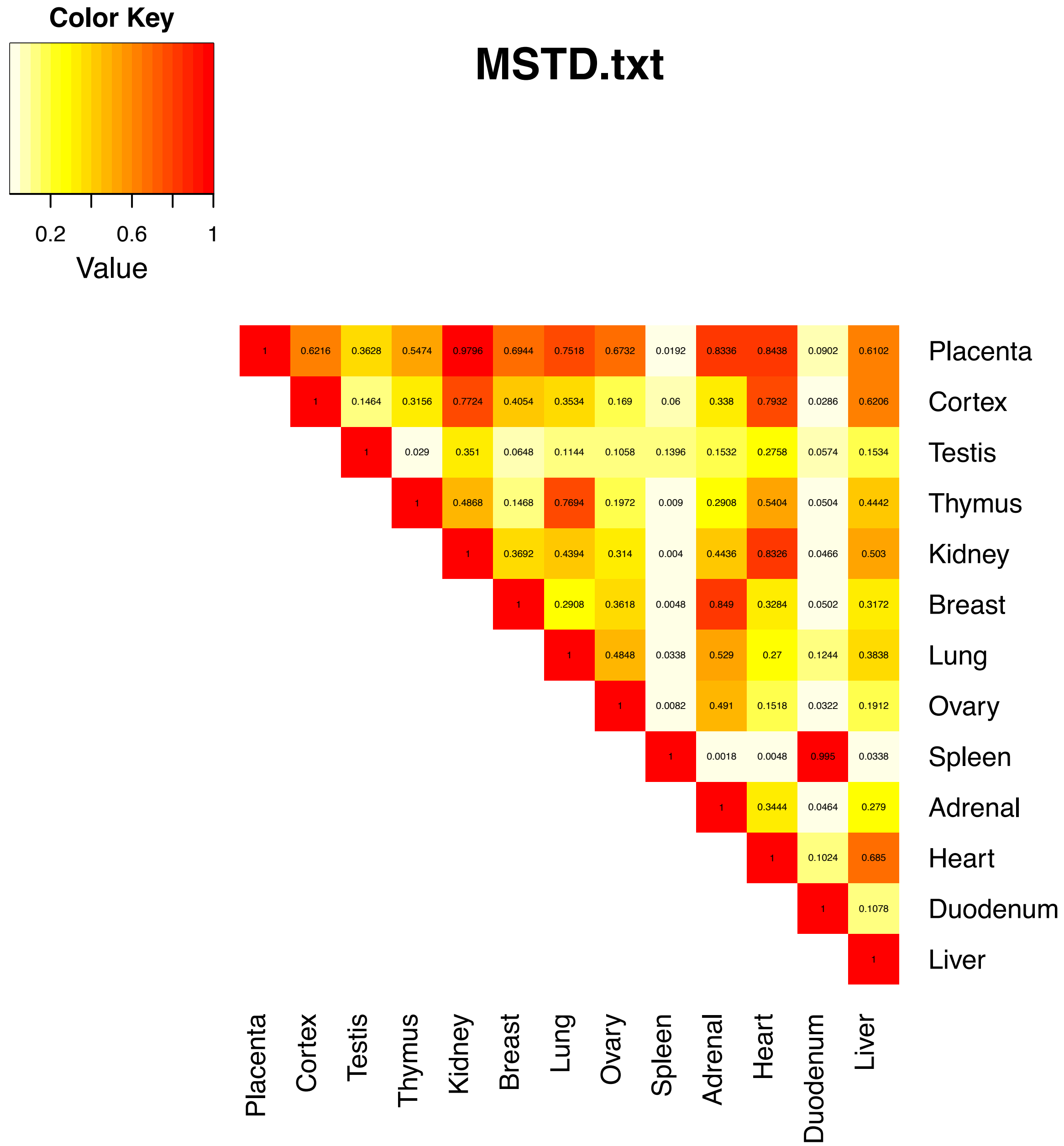
Color Key
0.2
0.6
1
Value
MSTD.txt
Placenta 1 0.6216 0.3628 0.5474 0.9796 0.6944 0.7518 0.6732 0.0192 0.8336 0.8438 0.0902 0.6102
Cortex 1 0.1464 0.3156 0.7724 0.4054 0.3534 0.169 0.06 0.338 0.7932 0.0286 0.6206
Testis 1 0.029 0.351 0.0648 0.1144 0.1058 0.1396 0.1532 0.2758 0.0574 0.1534
Thymus 1 0.4868 0.1468 0.7694 0.1972 0.009 0.2908 0.5404 0.0504 0.4442
Kidney 1 0.3692 0.4394 0.314 0.004 0.4436 0.8326 0.0466 0.503
Breast 1 0.2908 0.3618 0.0048 0.849 0.3284 0.0502 0.3172
Lung 1 0.4848 0.0338 0.529 0.27 0.1244 0.3838
Ovary 1 0.0082 0.491 0.1518 0.0322 0.1912
Spleen 1 0.0018 0.0048 0.995 0.0338
Adrenal 1 0.3444 0.0464 0.279
Heart 1 0.1024 0.685
Duodenum 1 0.1078
Liver 1
Placenta
Cortex
Testis
Thymus
Kidney
Breast
Lung
Ovary
Spleen
Adrenal
Heart
Duodenum
Liver

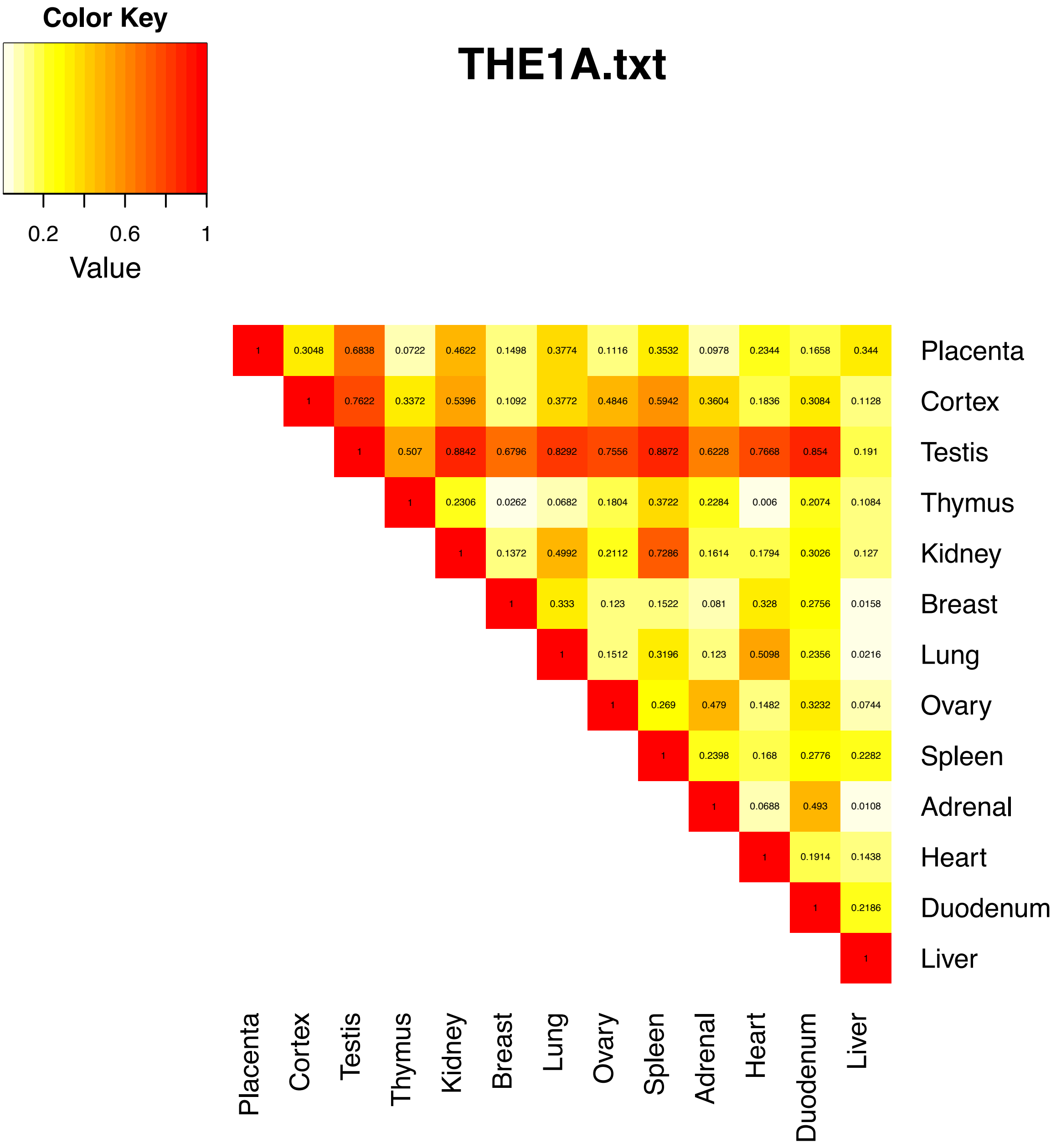
Color Key
0.2
0.6
1
Value
THE1A.txt
Placenta
Cortex
Testis
Thymus
Kidney
Breast
Lung
Ovary
Spleen
Adrenal
Heart
Duodenum
Liver
1 0.3048 0.6838 0.0722 0.4622 0.1498 0.3774 0.1116 0.3532 0.0978 0.2344 0.1658 0.344
1 0.7622 0.3372 0.5396 0.1092 0.3772 0.4846 0.5942 0.3604 0.1836 0.3084 0.1128
1 0.507 0.8842 0.6796 0.8292 0.7556 0.8872 0.6228 0.7668 0.854 0.191
1 0.2306 0.0262 0.0682 0.1804 0.3722 0.2284 0.006 0.2074 0.1084
1 0.1372 0.4992 0.2112 0.7286 0.1614 0.1794 0.3026 0.127
1 0.333 0.123 0.1522 0.081 0.328 0.2756 0.0158
1 0.1512 0.3196 0.123 0.5098 0.2356 0.0216
1 0.269 0.479 0.1482 0.3232 0.0744
1 0.2398 0.168 0.2776 0.2282
1 0.0688 0.493 0.0108
1 0.1914 0.1438
1 0.2186
1

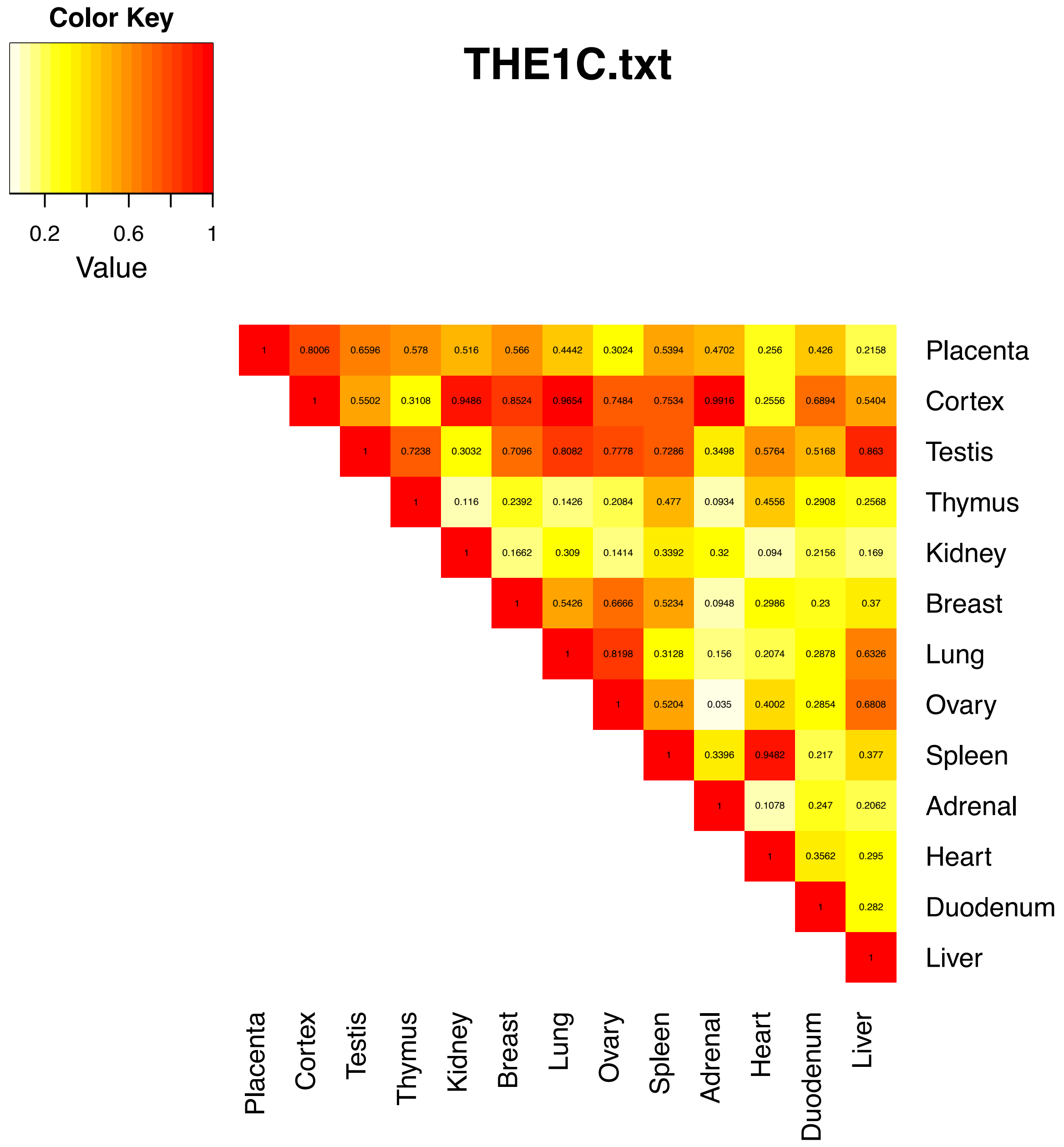
THE1C.txt
Color Key
0.2
0.6
1
Value
Placenta
Cortex
Testis
Thymus
Kidney
Breast
Lung
Ovary
Spleen
Adrenal
Heart
Duodenum
Liver
1 0.8006 0.6596 0.578 0.516 0.566 0.4442 0.3024 0.5394 0.4702 0.256 0.426 0.2158
1 0.5502 0.3108 0.9486 0.8524 0.9654 0.7484 0.7534 0.9916 0.2556 0.6894 0.5404
1 0.7238 0.3032 0.7096 0.8082 0.7778 0.7286 0.3498 0.5764 0.5168 0.863
1 0.116 0.2392 0.1426 0.2084 0.477 0.0934 0.4556 0.2908 0.2568
1 0.1662 0.309 0.1414 0.3392 0.32 0.094 0.2156 0.169
1 0.5426 0.6666 0.5234 0.0948 0.2986 0.23 0.37
1 0.8198 0.3128 0.156 0.2074 0.2878 0.6326
1 0.5204 0.035 0.4002 0.2854 0.6808
1 0.3396 0.9482 0.217 0.377
1 0.1078 0.247 0.2062
1 0.3562 0.295
1 0.282
1

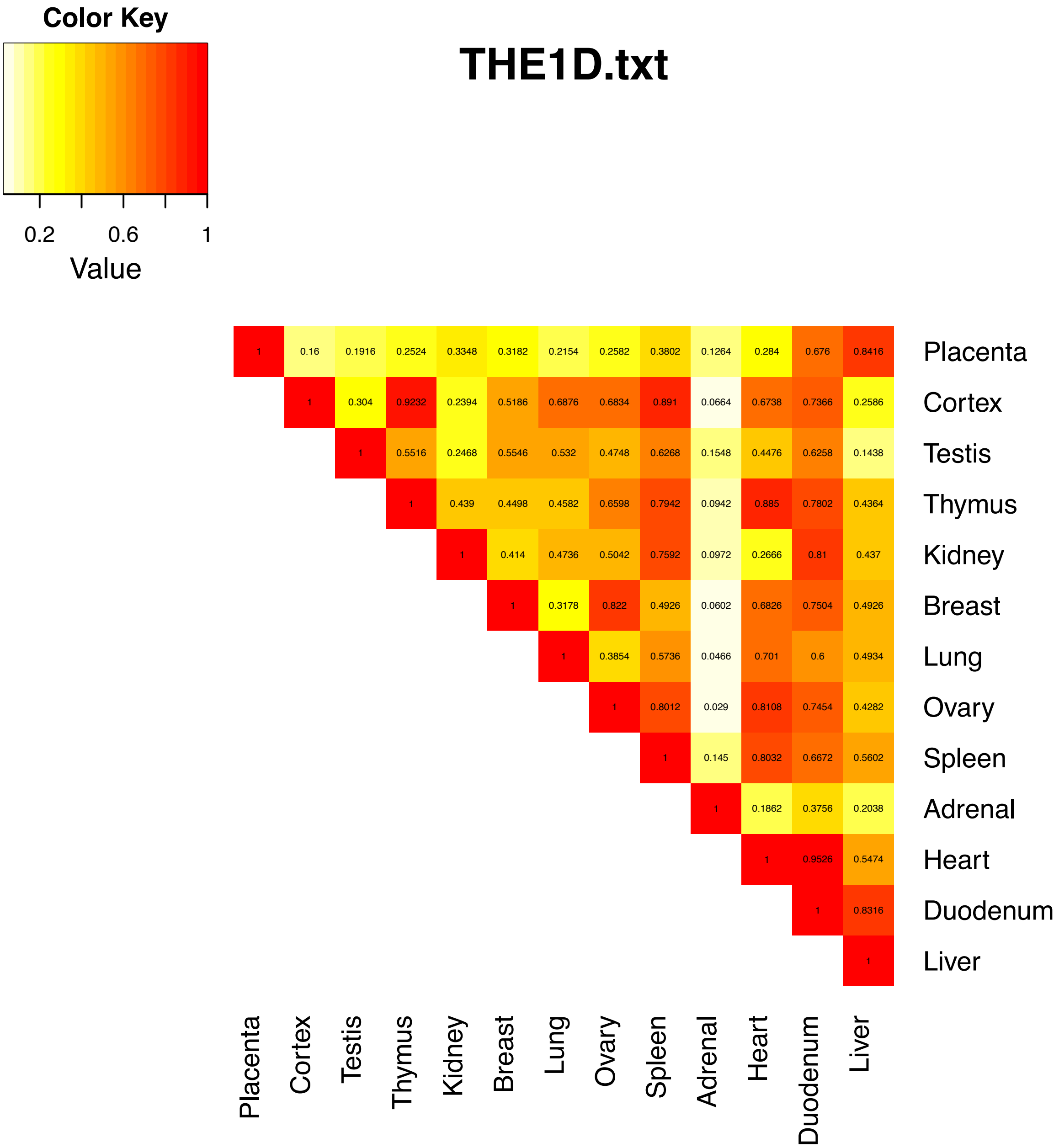
Color Key
0.2
0.6
1
Value
THE1D.txt
Placenta: 1 0.16 0.1916 0.2524 0.3348 0.3182 0.2154 0.2582 0.3802 0.1264 0.284 0.676 0.8416
Cortex: 1 0.304 0.9232 0.2394 0.5186 0.6876 0.6834 0.891 0.0664 0.6738 0.7366 0.2586
Testis: 1 0.5516 0.2468 0.5546 0.532 0.4748 0.6268 0.1548 0.4476 0.6258 0.1438
Thymus: 1 0.439 0.4498 0.4582 0.6598 0.7942 0.0942 0.885 0.7802 0.4364
Kidney: 1 0.414 0.4736 0.5042 0.7592 0.0972 0.2666 0.81 0.437
Breast: 1 0.3178 0.822 0.4926 0.0602 0.6826 0.7504 0.4926
Lung: 1 0.3854 0.5736 0.0466 0.701 0.6 0.4934
Ovary: 1 0.8012 0.029 0.8108 0.7454 0.4282
Spleen: 1 0.145 0.8032 0.6672 0.5602
Adrenal: 1 0.1862 0.3756 0.2038
Heart: 1 0.9526 0.5474
Duodenum: 1 0.8316
Liver: 1
Placenta
Cortex
Testis
Thymus
Kidney
Breast
Lung
Ovary
Spleen
Adrenal
Heart
Duodenum
Liver
