# Supplementary material for: Detecting Endogenous Retrovirus-Driven Tissue-Specific Gene Transcription
Source: Genome Biol Evol. 2015 Mar 11;7(4):1082–97. doi: 10.1093/gbe/evv049 (PMC4419796; doi:10.1093/gbe/evv049)
Supplement: Supplementary Data [file supp_evv049_S3_TEPlots_20kb.pdf]

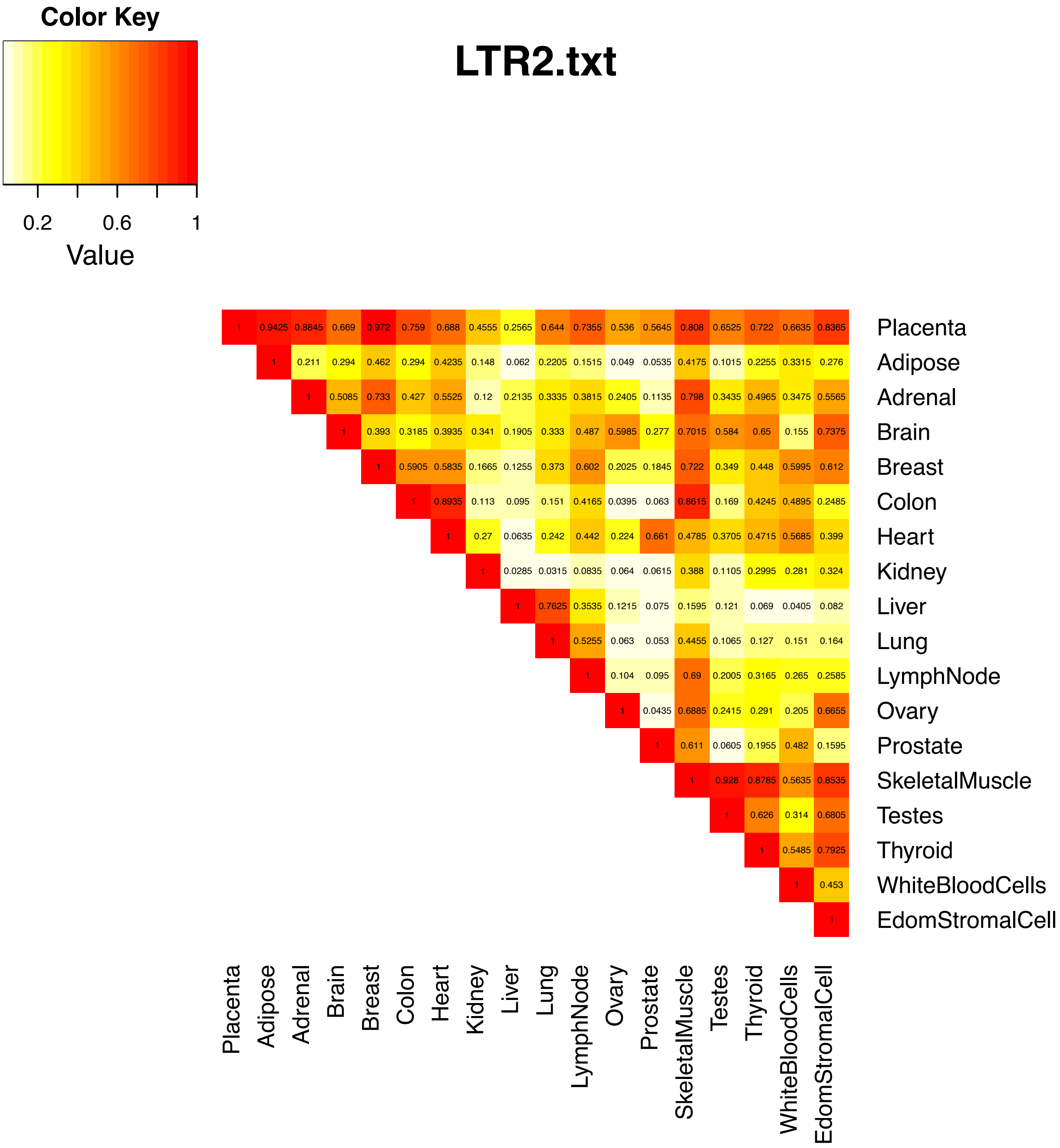
Color Key
0.2
0.6
1
Value
LTR2.txt
Placenta 1 0.9425 0.8845 0.669 0.972 0.759 0.688 0.4555 0.2565 0.644 0.7355 0.536 0.5645 0.808 0.6525 0.722 0.6635 0.8365
Adipose 1 0.211 0.294 0.462 0.294 0.4235 0.148 0.062 0.2205 0.1515 0.049 0.0535 0.4175 0.1015 0.2255 0.3315 0.276
Adrenal 1 0.5085 0.733 0.427 0.5525 0.12 0.2135 0.3335 0.3815 0.2405 0.1135 0.798 0.3435 0.4965 0.3475 0.5565
Brain 1 0.393 0.3185 0.3935 0.341 0.1905 0.333 0.487 0.5985 0.277 0.7015 0.584 0.65 0.155 0.7375
Breast 1 0.5905 0.5835 0.1665 0.1255 0.373 0.602 0.2025 0.1845 0.722 0.349 0.448 0.5995 0.612
Colon 1 0.8935 0.113 0.095 0.151 0.4165 0.0395 0.063 0.8615 0.169 0.4245 0.4895 0.2485
Heart 1 0.27 0.0635 0.242 0.442 0.224 0.661 0.4785 0.3705 0.4715 0.5685 0.399
Kidney 1 0.0285 0.0315 0.0835 0.064 0.0615 0.388 0.1105 0.2995 0.281 0.324
Liver 1 0.7625 0.3535 0.1215 0.075 0.1595 0.121 0.069 0.0405 0.082
Lung 1 0.5255 0.063 0.053 0.4455 0.1065 0.127 0.151 0.164
LymphNode 1 0.104 0.095 0.69 0.2005 0.3165 0.265 0.2585
Ovary 1 0.0435 0.6885 0.2415 0.291 0.205 0.6655
Prostate 1 0.611 0.0605 0.1955 0.482 0.1595
SkeletalMuscle 1 0.928 0.8785 0.5635 0.8535
Testes 1 0.626 0.314 0.6805
Thyroid 1 0.5485 0.7925
WhiteBloodCells 1 0.453
EdomStromalCell 1
Placenta
Adipose
Adrenal
Brain
Breast
Colon
Heart
Kidney
Liver
Lung
LymphNode
Ovary
Prostate
SkeletalMuscle
Testes
Thyroid
WhiteBloodCells
EdomStromalCell

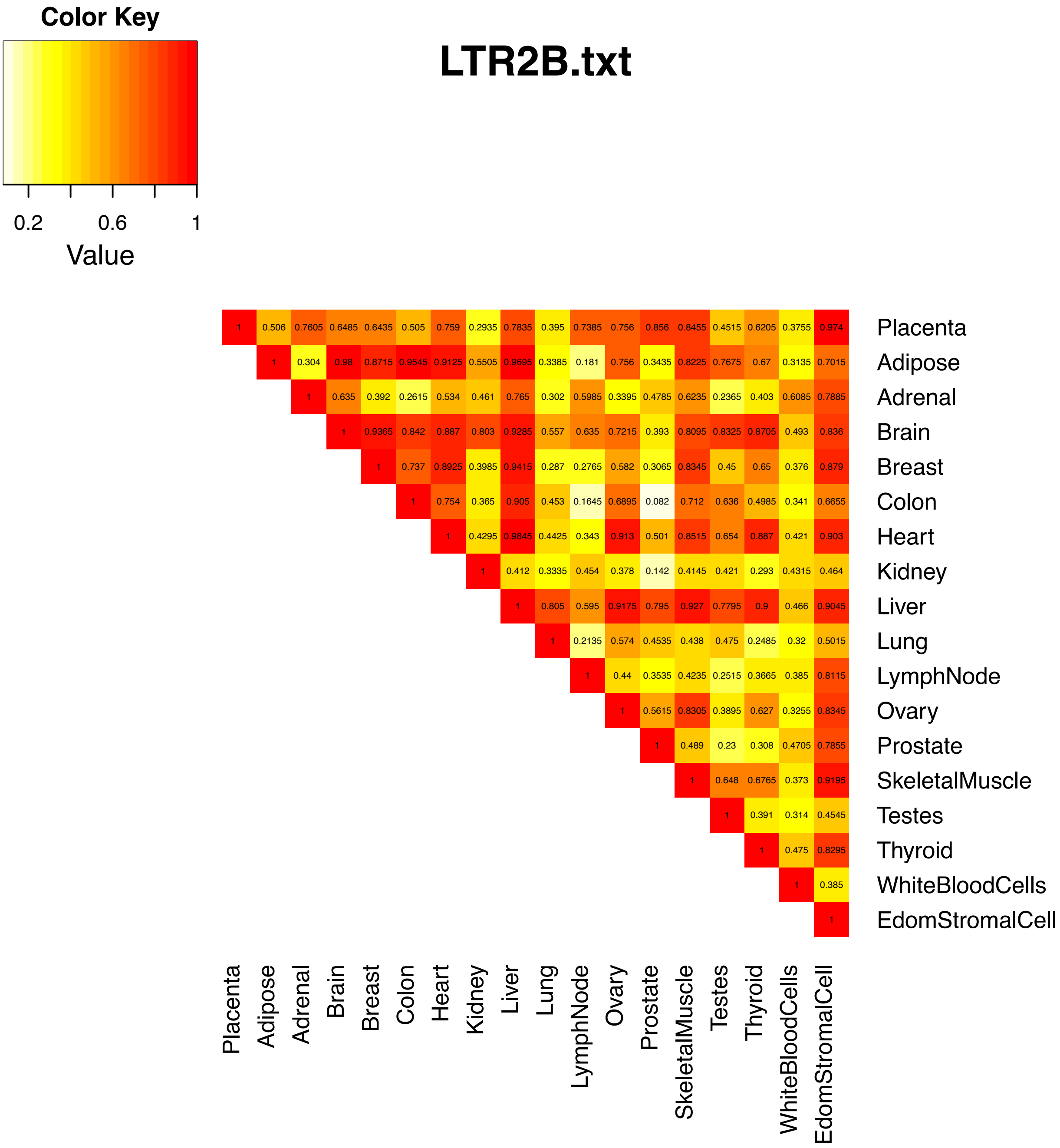
Color Key
0.2
0.6
1
Value
LTR2B.txt
Placenta: 1 0.506 0.7605 0.6485 0.6435 0.505 0.759 0.2935 0.7835 0.395 0.7385 0.756 0.856 0.8455 0.4515 0.6205 0.3755 0.974
Adipose: 1 0.304 0.98 0.8715 0.9545 0.9125 0.5505 0.9695 0.3385 0.181 0.756 0.3435 0.8225 0.7675 0.67 0.3135 0.7015
Adrenal: 1 0.635 0.392 0.2615 0.534 0.461 0.765 0.302 0.5985 0.3395 0.4785 0.6235 0.2365 0.403 0.6085 0.7885
Brain: 1 0.9365 0.842 0.887 0.803 0.9285 0.557 0.635 0.7215 0.393 0.8095 0.8325 0.8705 0.493 0.836
Breast: 1 0.737 0.8925 0.3985 0.9415 0.287 0.2765 0.582 0.3065 0.8345 0.45 0.65 0.376 0.879
Colon: 1 0.754 0.365 0.905 0.453 0.1645 0.6895 0.082 0.712 0.636 0.4985 0.341 0.6655
Heart: 1 0.4295 0.9845 0.4425 0.343 0.913 0.501 0.8515 0.654 0.887 0.421 0.903
Kidney: 1 0.412 0.3335 0.454 0.378 0.142 0.4145 0.421 0.293 0.4315 0.464
Liver: 1 0.805 0.595 0.9175 0.795 0.927 0.7795 0.9 0.466 0.9045
Lung: 1 0.2135 0.574 0.4535 0.438 0.475 0.2485 0.32 0.5015
LymphNode: 1 0.44 0.3535 0.4235 0.2515 0.3665 0.385 0.8115
Ovary: 1 0.5615 0.8305 0.3895 0.627 0.3255 0.8345
Prostate: 1 0.489 0.23 0.308 0.4705 0.7855
SkeletalMuscle: 1 0.648 0.6765 0.373 0.9195
Testes: 1 0.391 0.314 0.4545
Thyroid: 1 0.475 0.8295
WhiteBloodCells: 1 0.385
EdomStromalCell: 1
Placenta
Adipose
Adrenal
Brain
Breast
Colon
Heart
Kidney
Liver
Lung
LymphNode
Ovary
Prostate
SkeletalMuscle
Testes
Thyroid
WhiteBloodCells
EdomStromalCell

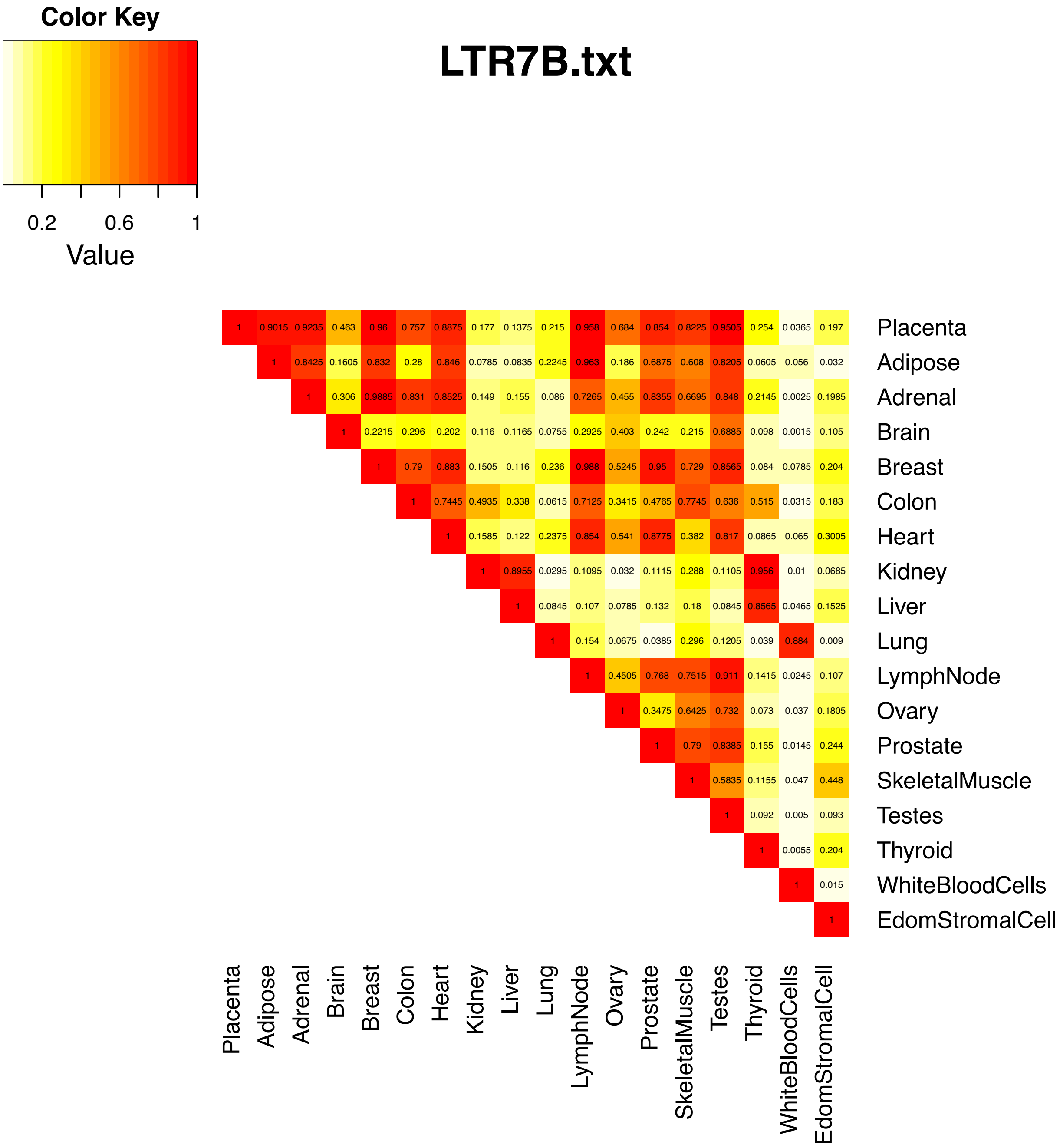

Color Key
0.2
0.6
1
Value
LTR7B.txt
Placenta
Adipose
Adrenal
Brain
Breast
Colon
Heart
Kidney
Liver
Lung
LymphNode
Ovary
Prostate
SkeletalMuscle
Testes
Thyroid
WhiteBloodCells
EdomStromalCell
1 0.9015 0.9235 0.463 0.96 0.757 0.8875 0.177 0.1375 0.215 0.958 0.684 0.854 0.8225 0.9505 0.254 0.0365 0.197
1 0.8425 0.1605 0.832 0.28 0.846 0.0785 0.0835 0.2245 0.963 0.186 0.6875 0.608 0.8205 0.0605 0.056 0.032
1 0.306 0.9885 0.831 0.8525 0.149 0.155 0.086 0.7265 0.455 0.8355 0.6695 0.848 0.2145 0.0025 0.1985
1 0.2215 0.296 0.202 0.116 0.1165 0.0755 0.2925 0.403 0.242 0.215 0.6885 0.098 0.0015 0.105
1 0.79 0.883 0.1505 0.116 0.236 0.988 0.5245 0.95 0.729 0.8565 0.084 0.0785 0.204
1 0.7445 0.4935 0.338 0.0615 0.7125 0.3415 0.4765 0.7745 0.636 0.515 0.0315 0.183
1 0.1585 0.122 0.2375 0.854 0.541 0.8775 0.382 0.817 0.0865 0.065 0.3005
1 0.8955 0.0295 0.1095 0.032 0.1115 0.288 0.1105 0.956 0.01 0.0685
1 0.0845 0.107 0.0785 0.132 0.18 0.0845 0.8565 0.0465 0.1525
1 0.154 0.0675 0.0385 0.296 0.1205 0.039 0.884 0.009
1 0.4505 0.768 0.7515 0.911 0.1415 0.0245 0.107
1 0.3475 0.6425 0.732 0.073 0.037 0.1805
1 0.79 0.8385 0.155 0.0145 0.244
1 0.5835 0.1155 0.047 0.448
1 0.092 0.005 0.093
1 0.0055 0.204
1 0.015
1

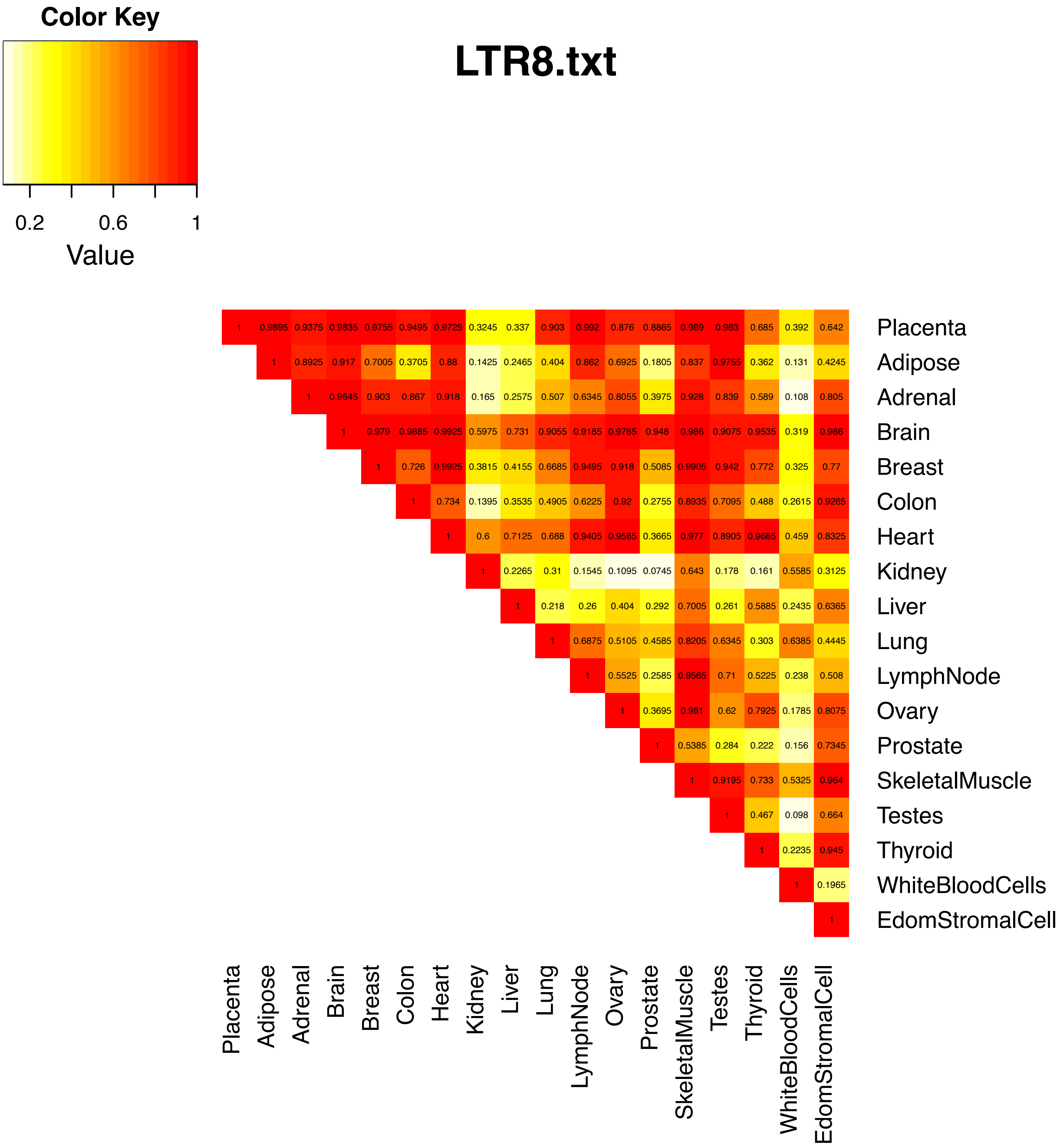
Color Key
0.2
0.6
1
Value
LTR8.txt
Placenta
Adipose
Adrenal
Brain
Breast
Colon
Heart
Kidney
Liver
Lung
LymphNode
Ovary
Prostate
SkeletalMuscle
Testes
Thyroid
WhiteBloodCells
EdomStromalCell
1 0.9895 0.9375 0.9835 0.9755 0.9495 0.9725 0.3245 0.337 0.903 0.992 0.876 0.8865 0.989 0.963 0.685 0.392 0.642
1 0.8925 0.917 0.7005 0.3705 0.88 0.1425 0.2465 0.404 0.862 0.6925 0.1805 0.837 0.9755 0.362 0.131 0.4245
1 0.9645 0.903 0.867 0.918 0.165 0.2575 0.507 0.6345 0.8055 0.3975 0.928 0.839 0.589 0.108 0.805
1 0.979 0.9885 0.9925 0.5975 0.731 0.9055 0.9185 0.9785 0.948 0.986 0.9075 0.9535 0.319 0.986
1 0.726 0.9925 0.3815 0.4155 0.6685 0.9495 0.918 0.5085 0.9905 0.942 0.772 0.325 0.77
1 0.734 0.1395 0.3535 0.4905 0.6225 0.92 0.2755 0.8935 0.7095 0.488 0.2615 0.9265
1 0.6 0.7125 0.688 0.9405 0.9585 0.3665 0.977 0.8905 0.9685 0.459 0.8325
1 0.2265 0.31 0.1545 0.1095 0.0745 0.643 0.178 0.161 0.5585 0.3125
1 0.218 0.26 0.404 0.292 0.7005 0.261 0.5885 0.2435 0.6365
1 0.6875 0.5105 0.4585 0.8205 0.6345 0.303 0.6385 0.4445
1 0.5525 0.2585 0.9565 0.71 0.5225 0.238 0.508
1 0.3695 0.981 0.62 0.7925 0.1785 0.8075
1 0.5385 0.284 0.222 0.156 0.7345
1 0.9195 0.733 0.5325 0.964
1 0.467 0.098 0.664
1 0.2235 0.945
1 0.1965
1

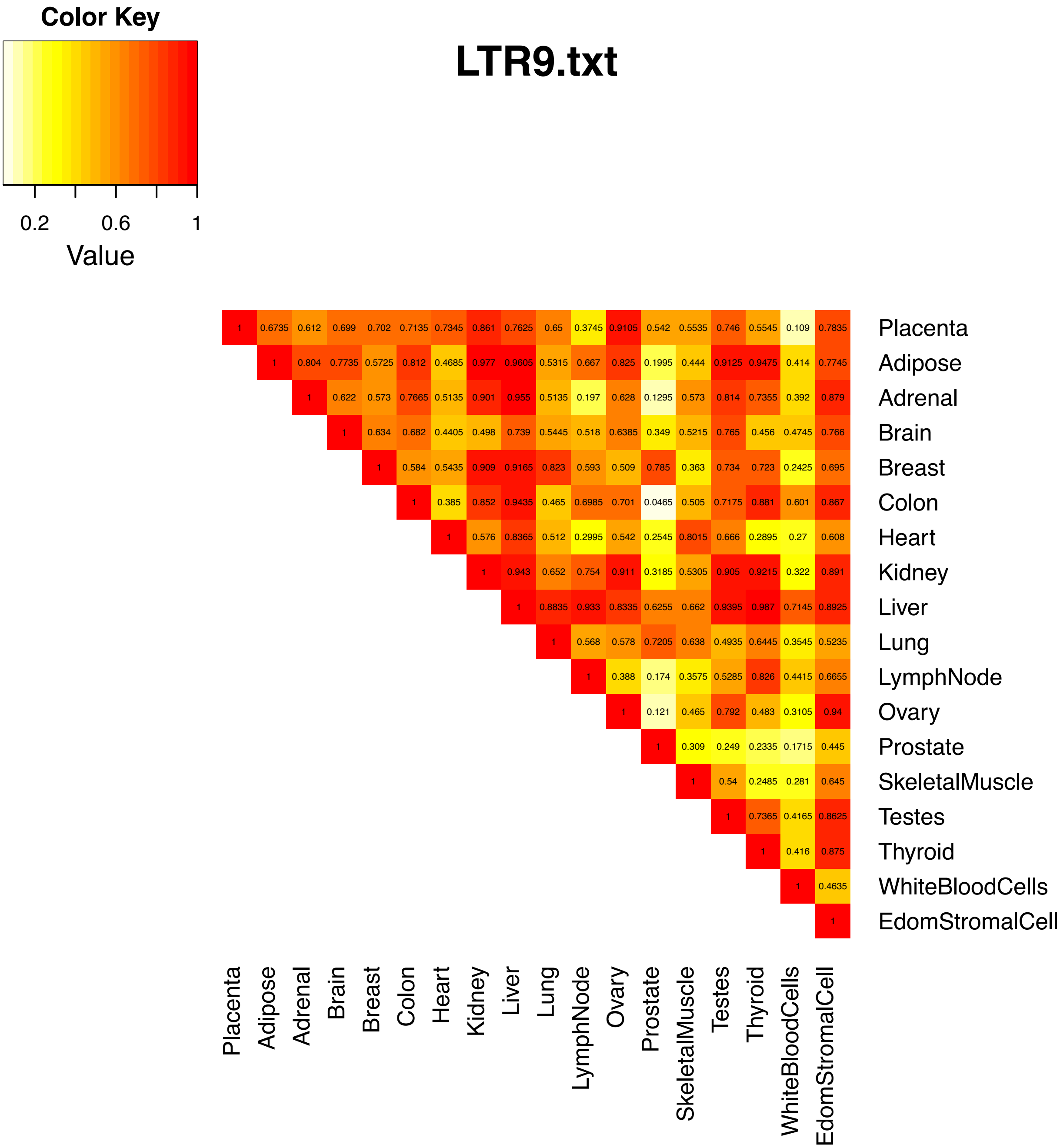
Color Key
0.2
0.6
1
Value
LTR9.txt
Placenta 1 0.6735 0.612 0.699 0.702 0.7135 0.7345 0.861 0.7625 0.65 0.3745 0.9105 0.542 0.5535 0.746 0.5545 0.109 0.7835
Adipose 1 0.804 0.7735 0.5725 0.812 0.4685 0.977 0.9605 0.5315 0.667 0.825 0.1995 0.444 0.9125 0.9475 0.414 0.7745
Adrenal 1 0.622 0.573 0.7665 0.5135 0.901 0.955 0.5135 0.197 0.628 0.1295 0.573 0.814 0.7355 0.392 0.879
Brain 1 0.634 0.682 0.4405 0.498 0.739 0.5445 0.518 0.6385 0.349 0.5215 0.765 0.456 0.4745 0.766
Breast 1 0.584 0.5435 0.909 0.9165 0.823 0.593 0.509 0.785 0.363 0.734 0.723 0.2425 0.695
Colon 1 0.385 0.852 0.9435 0.465 0.6985 0.701 0.0465 0.505 0.7175 0.881 0.601 0.867
Heart 1 0.576 0.8365 0.512 0.2995 0.542 0.2545 0.8015 0.666 0.2895 0.27 0.608
Kidney 1 0.943 0.652 0.754 0.911 0.3185 0.5305 0.905 0.9215 0.322 0.891
Liver 1 0.8835 0.933 0.8335 0.6255 0.662 0.9395 0.987 0.7145 0.8925
Lung 1 0.568 0.578 0.7205 0.638 0.4935 0.6445 0.3545 0.5235
LymphNode 1 0.388 0.174 0.3575 0.5285 0.826 0.4415 0.6655
Ovary 1 0.121 0.465 0.792 0.483 0.3105 0.94
Prostate 1 0.309 0.249 0.2335 0.1715 0.445
SkeletalMuscle 1 0.54 0.2485 0.281 0.645
Testes 1 0.7365 0.4165 0.8625
Thyroid 1 0.416 0.875
WhiteBloodCells 1 0.4635
EdomStromalCell 1
Placenta
Adipose
Adrenal
Brain
Breast
Colon
Heart
Kidney
Liver
Lung
LymphNode
Ovary
Prostate
SkeletalMuscle
Testes
Thyroid
WhiteBloodCells
EdomStromalCell

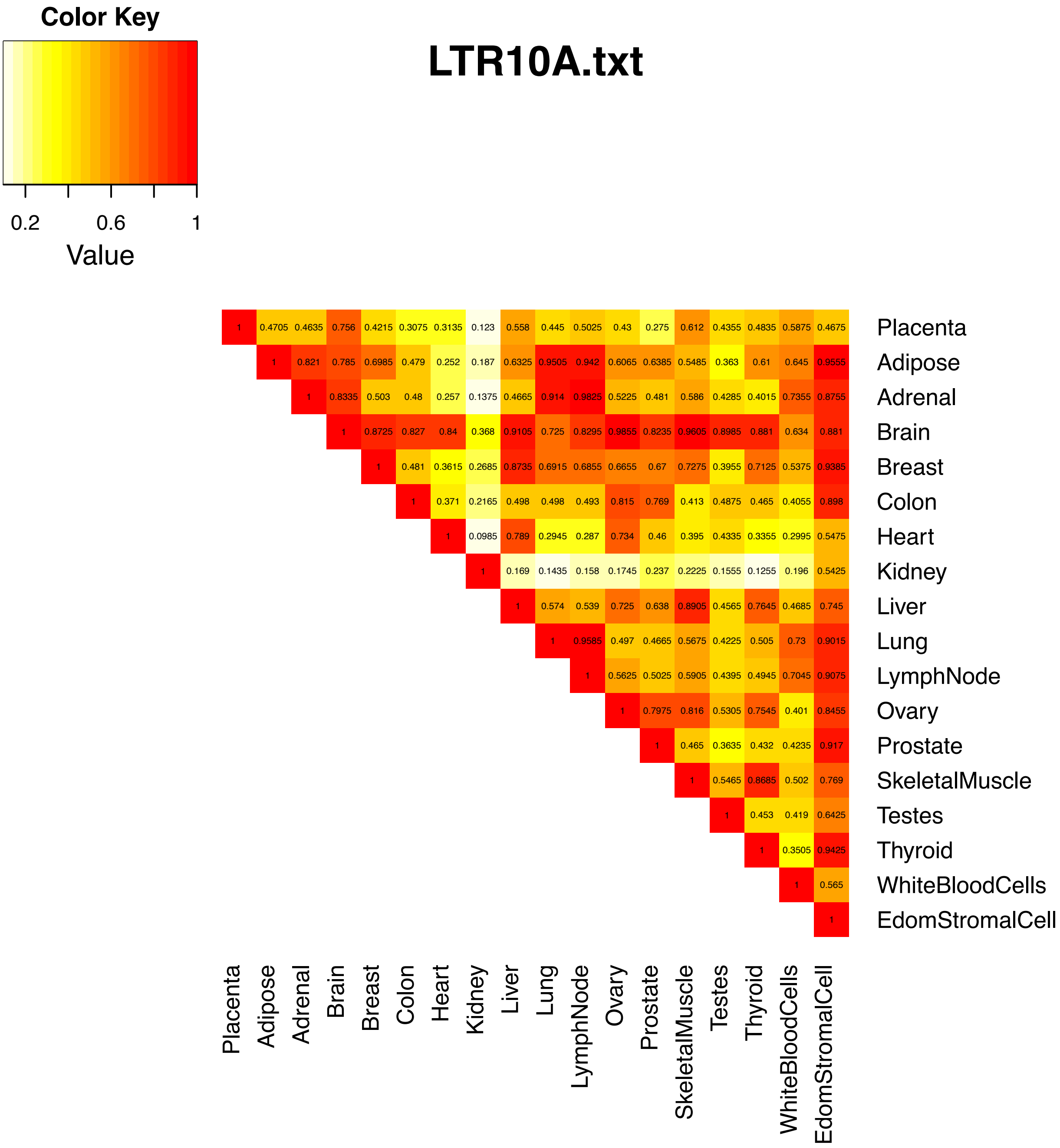
Color Key
0.2
0.6
1
Value
LTR10A.txt
1 0.4705 0.4635 0.756 0.4215 0.3075 0.3135 0.123 0.558 0.445 0.5025 0.43 0.275 0.612 0.4355 0.4835 0.5875 0.4675 Placenta
1 0.821 0.785 0.6985 0.479 0.252 0.187 0.6325 0.9505 0.942 0.6065 0.6385 0.5485 0.363 0.61 0.645 0.9555 Adipose
1 0.8335 0.503 0.48 0.257 0.1375 0.4665 0.914 0.9825 0.5225 0.481 0.586 0.4285 0.4015 0.7355 0.8755 Adrenal
1 0.8725 0.827 0.84 0.368 0.9105 0.725 0.8295 0.9855 0.8235 0.9605 0.8985 0.881 0.634 0.881 Brain
1 0.481 0.3615 0.2685 0.8735 0.6915 0.6855 0.6655 0.67 0.7275 0.3955 0.7125 0.5375 0.9385 Breast
1 0.371 0.2165 0.498 0.498 0.493 0.815 0.769 0.413 0.4875 0.465 0.4055 0.898 Colon
1 0.0985 0.789 0.2945 0.287 0.734 0.46 0.395 0.4335 0.3355 0.2995 0.5475 Heart
1 0.169 0.1435 0.158 0.1745 0.237 0.2225 0.1555 0.1255 0.196 0.5425 Kidney
1 0.574 0.539 0.725 0.638 0.8905 0.4565 0.7645 0.4685 0.745 Liver
1 0.9585 0.497 0.4665 0.5675 0.4225 0.505 0.73 0.9015 Lung
1 0.5625 0.5025 0.5905 0.4395 0.4945 0.7045 0.9075 LymphNode
1 0.7975 0.816 0.5305 0.7545 0.401 0.8455 Ovary
1 0.465 0.3635 0.432 0.4235 0.917 Prostate
1 0.5465 0.8685 0.502 0.769 SkeletalMuscle
1 0.453 0.419 0.6425 Testes
1 0.3505 0.9425 Thyroid
1 0.565 WhiteBloodCells
1 EdomStromalCell
Placenta
Adipose
Adrenal
Brain
Breast
Colon
Heart
Kidney
Liver
Lung
LymphNode
Ovary
Prostate
SkeletalMuscle
Testes
Thyroid
WhiteBloodCells
EdomStromalCell

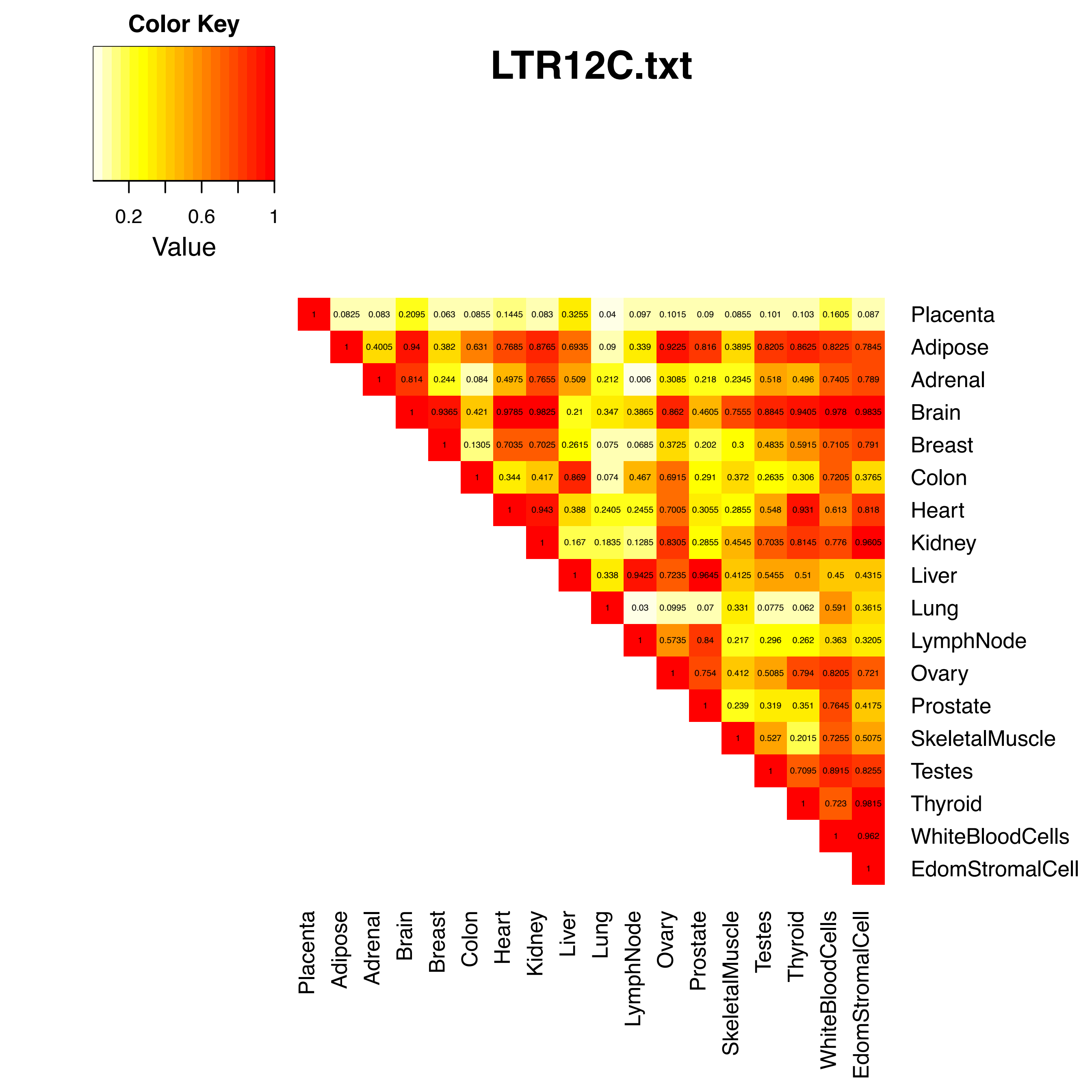

Color Key
0.2
0.6
1
Value
LTR12C.txt
1 0.0825 0.083 0.2095 0.063 0.0855 0.1445 0.083 0.3255 0.04 0.097 0.1015 0.09 0.0855 0.101 0.103 0.1605 0.087 Placenta
1 0.4005 0.94 0.382 0.631 0.7685 0.8765 0.6935 0.09 0.339 0.9225 0.816 0.3895 0.8205 0.8625 0.8225 0.7845 Adipose
1 0.814 0.244 0.084 0.4975 0.7655 0.509 0.212 0.006 0.3085 0.218 0.2345 0.518 0.496 0.7405 0.789 Adrenal
1 0.9365 0.421 0.9785 0.9825 0.21 0.347 0.3865 0.862 0.4605 0.7555 0.8845 0.9405 0.978 0.9835 Brain
1 0.1305 0.7035 0.7025 0.2615 0.075 0.0685 0.3725 0.202 0.3 0.4835 0.5915 0.7105 0.791 Breast
1 0.344 0.417 0.869 0.074 0.467 0.6915 0.291 0.372 0.2635 0.306 0.7205 0.3765 Colon
1 0.943 0.388 0.2405 0.2455 0.7005 0.3055 0.2855 0.548 0.931 0.613 0.818 Heart
1 0.167 0.1835 0.1285 0.8305 0.2855 0.4545 0.7035 0.8145 0.776 0.9605 Kidney
1 0.338 0.9425 0.7235 0.9645 0.4125 0.5455 0.51 0.45 0.4315 Liver
1 0.03 0.0995 0.07 0.331 0.0775 0.062 0.591 0.3615 Lung
1 0.5735 0.84 0.217 0.296 0.262 0.363 0.3205 LymphNode
1 0.754 0.412 0.5085 0.794 0.8205 0.721 Ovary
1 0.239 0.319 0.351 0.7645 0.4175 Prostate
1 0.527 0.2015 0.7255 0.5075 SkeletalMuscle
1 0.7095 0.8915 0.8255 Testes
1 0.723 0.9815 Thyroid
1 0.962 WhiteBloodCells
1 EdomStromalCell
Placenta
Adipose
Adrenal
Brain
Breast
Colon
Heart
Kidney
Liver
Lung
LymphNode
Ovary
Prostate
SkeletalMuscle
Testes
Thyroid
WhiteBloodCells
EdomStromalCell

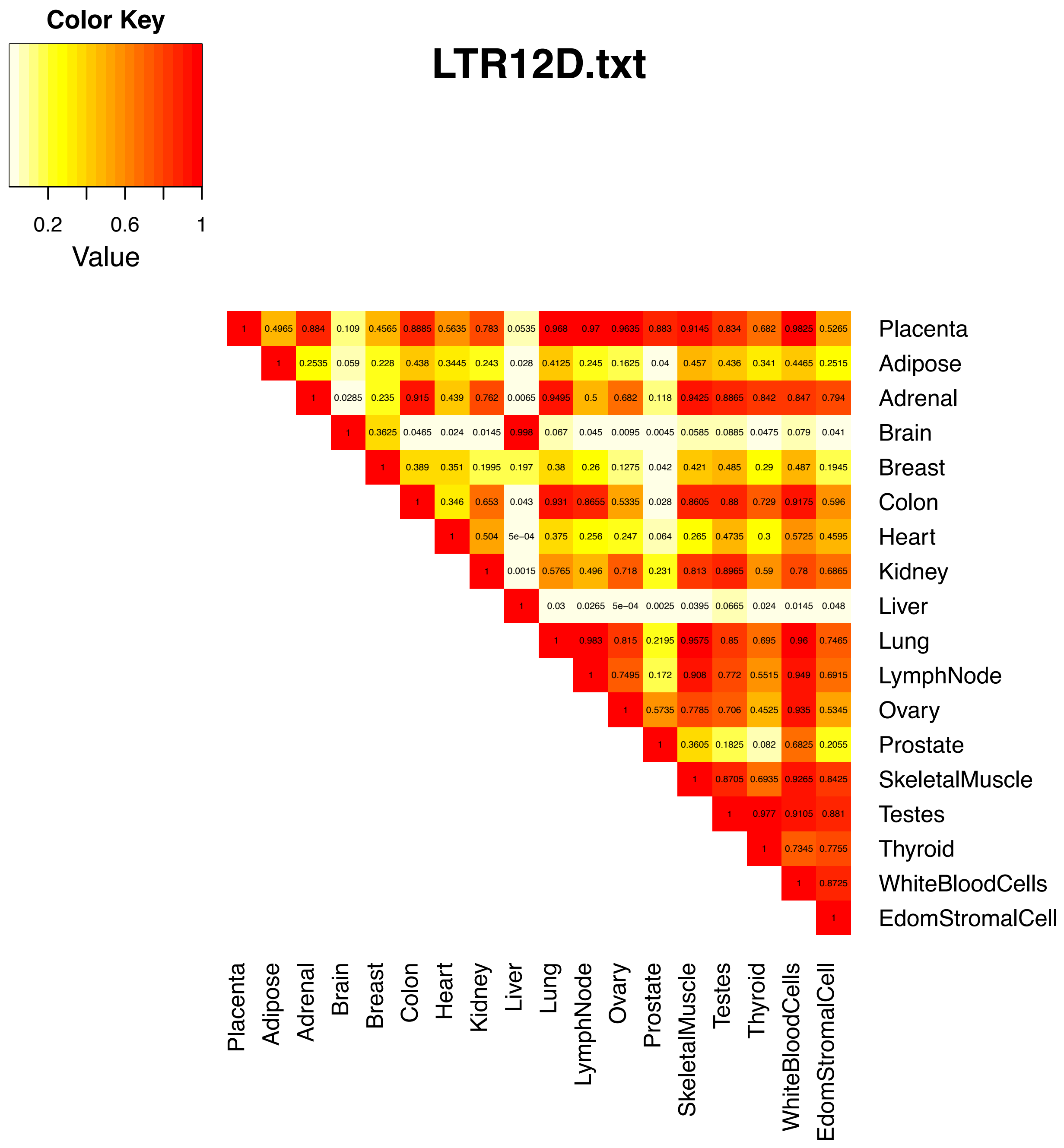
Color Key
0.2
0.6
1
Value
LTR12D.txt
Placenta 1 0.4965 0.884 0.109 0.4565 0.8885 0.5635 0.783 0.0535 0.968 0.97 0.9635 0.883 0.9145 0.834 0.682 0.9825 0.5265
Adipose 1 0.2535 0.059 0.228 0.438 0.3445 0.243 0.028 0.4125 0.245 0.1625 0.04 0.457 0.436 0.341 0.4465 0.2515
Adrenal 1 0.0285 0.235 0.915 0.439 0.762 0.0065 0.9495 0.5 0.682 0.118 0.9425 0.8865 0.842 0.847 0.794
Brain 1 0.3625 0.0465 0.024 0.0145 0.998 0.067 0.045 0.0095 0.0045 0.0585 0.0885 0.0475 0.079 0.041
Breast 1 0.389 0.351 0.1995 0.197 0.38 0.26 0.1275 0.042 0.421 0.485 0.29 0.487 0.1945
Colon 1 0.346 0.653 0.043 0.931 0.8655 0.5335 0.028 0.8605 0.88 0.729 0.9175 0.596
Heart 1 0.504 5e−04 0.375 0.256 0.247 0.064 0.265 0.4735 0.3 0.5725 0.4595
Kidney 1 0.0015 0.5765 0.496 0.718 0.231 0.813 0.8965 0.59 0.78 0.6865
Liver 1 0.03 0.0265 5e−04 0.0025 0.0395 0.0665 0.024 0.0145 0.048
Lung 1 0.983 0.815 0.2195 0.9575 0.85 0.695 0.96 0.7465
LymphNode 1 0.7495 0.172 0.908 0.772 0.5515 0.949 0.6915
Ovary 1 0.5735 0.7785 0.706 0.4525 0.935 0.5345
Prostate 1 0.3605 0.1825 0.082 0.6825 0.2055
SkeletalMuscle 1 0.8705 0.6935 0.9265 0.8425
Testes 1 0.977 0.9105 0.881
Thyroid 1 0.7345 0.7755
WhiteBloodCells 1 0.8725
EdomStromalCell 1
Placenta
Adipose
Adrenal
Brain
Breast
Colon
Heart
Kidney
Liver
Lung
LymphNode
Ovary
Prostate
SkeletalMuscle
Testes
Thyroid
WhiteBloodCells
EdomStromalCell

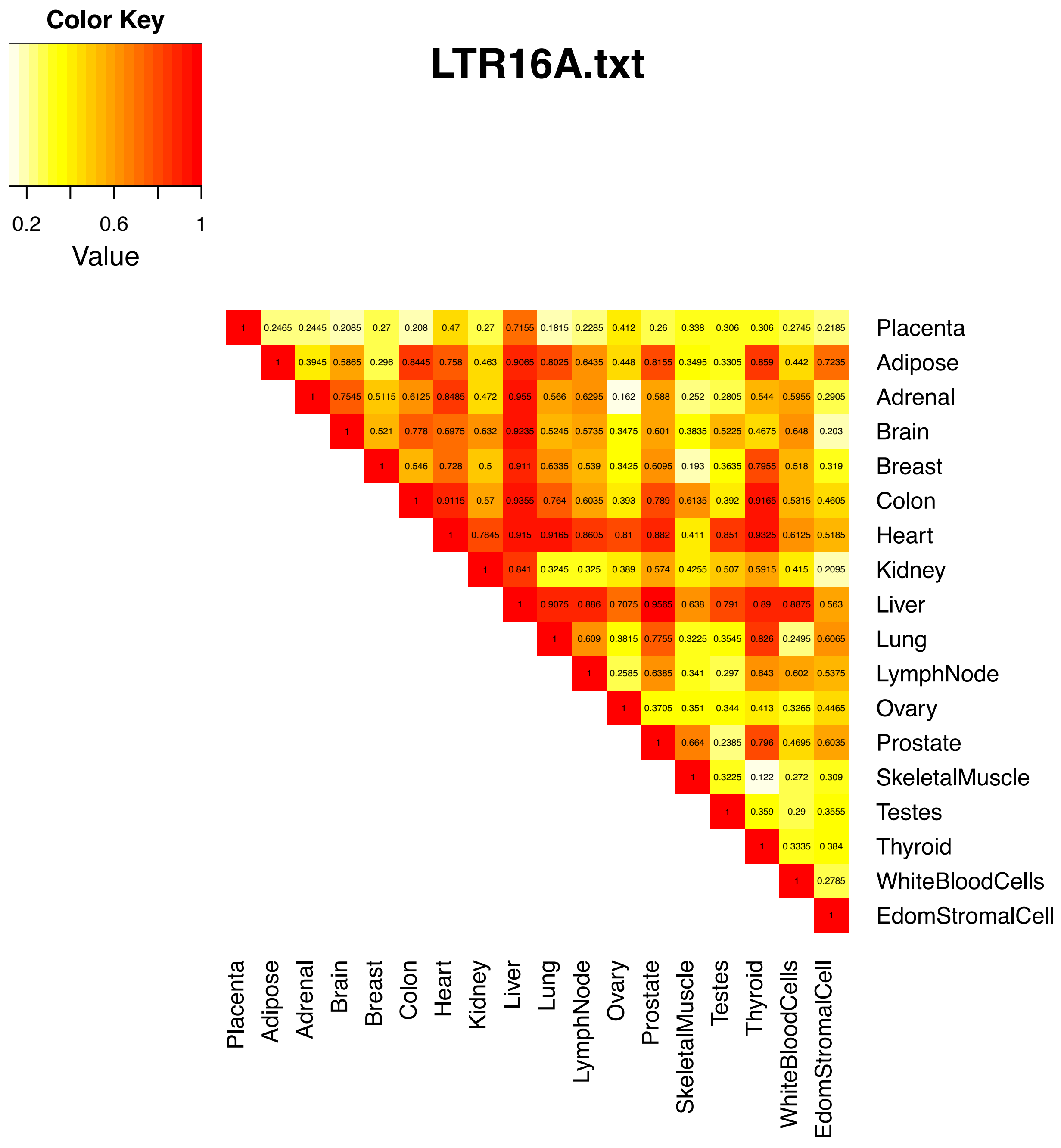
LTR16A.txt
Color Key
0.2 0.6 1
Value
Placenta 1 0.2465 0.2445 0.2085 0.27 0.208 0.47 0.27 0.7155 0.1815 0.2285 0.412 0.26 0.338 0.306 0.306 0.2745 0.2185
Adipose 1 0.3945 0.5865 0.296 0.8445 0.758 0.463 0.9065 0.8025 0.6435 0.448 0.8155 0.3495 0.3305 0.859 0.442 0.7235
Adrenal 1 0.7545 0.5115 0.6125 0.8485 0.472 0.955 0.566 0.6295 0.162 0.588 0.252 0.2805 0.544 0.5955 0.2905
Brain 1 0.521 0.778 0.6975 0.632 0.9235 0.5245 0.5735 0.3475 0.601 0.3835 0.5225 0.4675 0.648 0.203
Breast 1 0.546 0.728 0.5 0.911 0.6335 0.539 0.3425 0.6095 0.193 0.3635 0.7955 0.518 0.319
Colon 1 0.9115 0.57 0.9355 0.764 0.6035 0.393 0.789 0.6135 0.392 0.9165 0.5315 0.4605
Heart 1 0.7845 0.915 0.9165 0.8605 0.81 0.882 0.411 0.851 0.9325 0.6125 0.5185
Kidney 1 0.841 0.3245 0.325 0.389 0.574 0.4255 0.507 0.5915 0.415 0.2095
Liver 1 0.9075 0.886 0.7075 0.9565 0.638 0.791 0.89 0.8875 0.563
Lung 1 0.609 0.3815 0.7755 0.3225 0.3545 0.826 0.2495 0.6065
LymphNode 1 0.2585 0.6385 0.341 0.297 0.643 0.602 0.5375
Ovary 1 0.3705 0.351 0.344 0.413 0.3265 0.4465
Prostate 1 0.664 0.2385 0.796 0.4695 0.6035
SkeletalMuscle 1 0.3225 0.122 0.272 0.309
Testes 1 0.359 0.29 0.3555
Thyroid 1 0.3335 0.384
WhiteBloodCells 1 0.2785
EdomStromalCell 1
Placenta
Adipose
Adrenal
Brain
Breast
Colon
Heart
Kidney
Liver
Lung
LymphNode
Ovary
Prostate
SkeletalMuscle
Testes
Thyroid
WhiteBloodCells
EdomStromalCell

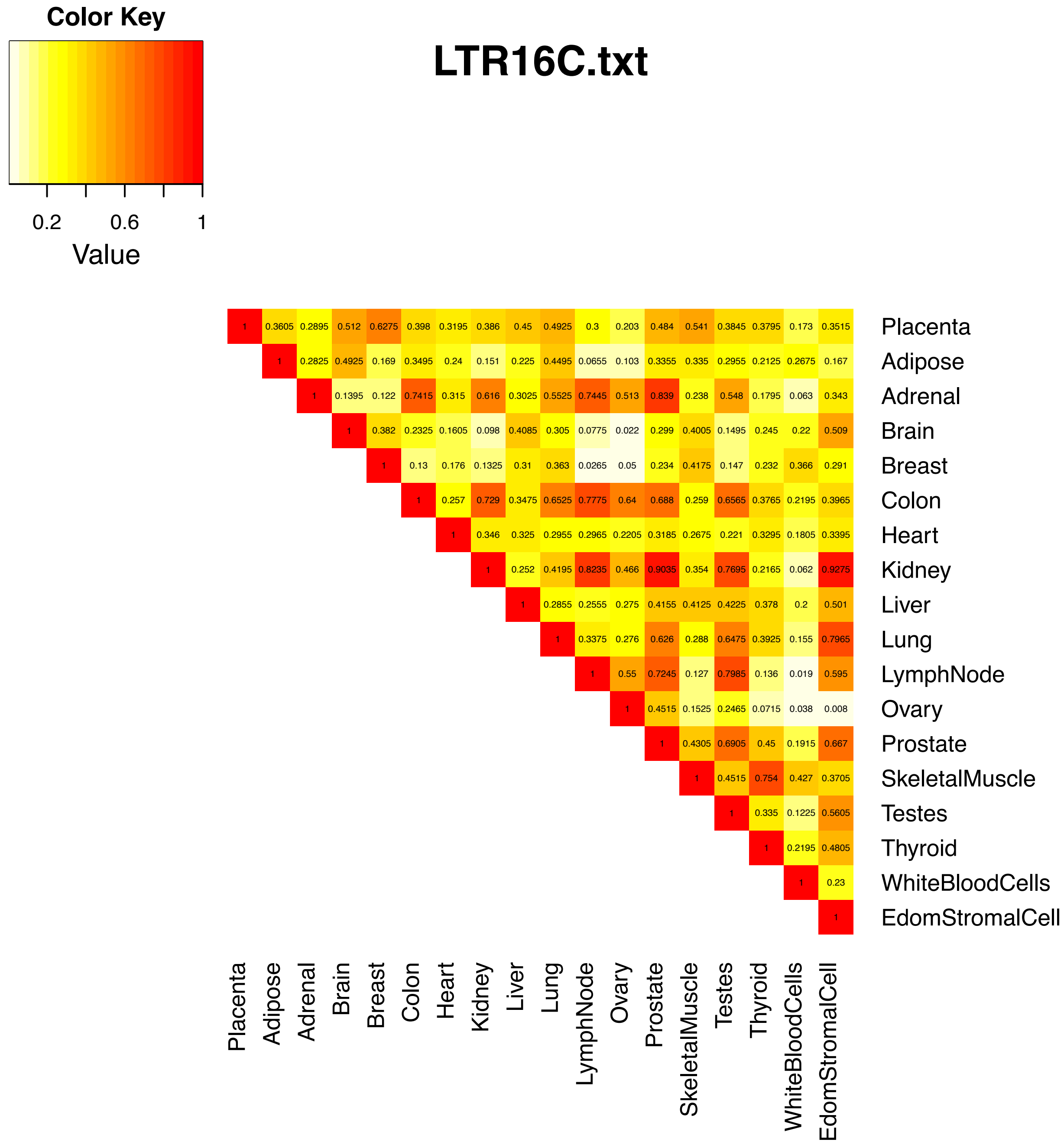
Color Key
0.2
0.6
1
Value
LTR16C.txt
1 0.3605 0.2895 0.512 0.6275 0.398 0.3195 0.386 0.45 0.4925 0.3 0.203 0.484 0.541 0.3845 0.3795 0.173 0.3515 Placenta
1 0.2825 0.4925 0.169 0.3495 0.24 0.151 0.225 0.4495 0.0655 0.103 0.3355 0.335 0.2955 0.2125 0.2675 0.167 Adipose
1 0.1395 0.122 0.7415 0.315 0.616 0.3025 0.5525 0.7445 0.513 0.839 0.238 0.548 0.1795 0.063 0.343 Adrenal
1 0.382 0.2325 0.1605 0.098 0.4085 0.305 0.0775 0.022 0.299 0.4005 0.1495 0.245 0.22 0.509 Brain
1 0.13 0.176 0.1325 0.31 0.363 0.0265 0.05 0.234 0.4175 0.147 0.232 0.366 0.291 Breast
1 0.257 0.729 0.3475 0.6525 0.7775 0.64 0.688 0.259 0.6565 0.3765 0.2195 0.3965 Colon
1 0.346 0.325 0.2955 0.2965 0.2205 0.3185 0.2675 0.221 0.3295 0.1805 0.3395 Heart
1 0.252 0.4195 0.8235 0.466 0.9035 0.354 0.7695 0.2165 0.062 0.9275 Kidney
1 0.2855 0.2555 0.275 0.4155 0.4125 0.4225 0.378 0.2 0.501 Liver
1 0.3375 0.276 0.626 0.288 0.6475 0.3925 0.155 0.7965 Lung
1 0.55 0.7245 0.127 0.7985 0.136 0.019 0.595 LymphNode
1 0.4515 0.1525 0.2465 0.0715 0.038 0.008 Ovary
1 0.4305 0.6905 0.45 0.1915 0.667 Prostate
1 0.4515 0.754 0.427 0.3705 SkeletalMuscle
1 0.335 0.1225 0.5605 Testes
1 0.2195 0.4805 Thyroid
1 0.23 WhiteBloodCells
1 EdomStromalCell
Placenta
Adipose
Adrenal
Brain
Breast
Colon
Heart
Kidney
Liver
Lung
LymphNode
Ovary
Prostate
SkeletalMuscle
Testes
Thyroid
WhiteBloodCells
EdomStromalCell

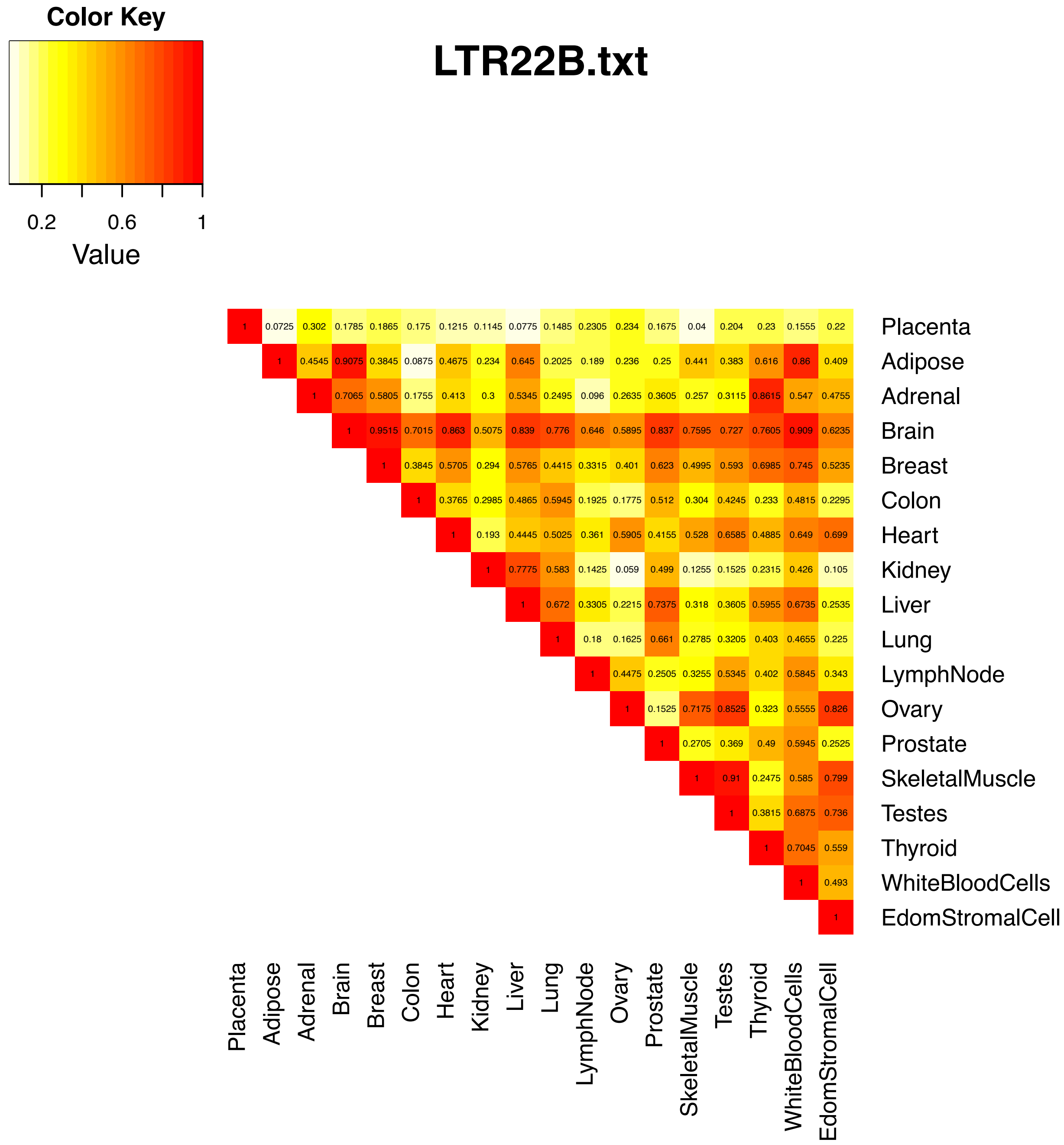
Color Key
0.2
0.6
1
Value
LTR22B.txt
1 0.0725 0.302 0.1785 0.1865 0.175 0.1215 0.1145 0.0775 0.1485 0.2305 0.234 0.1675 0.04 0.204 0.23 0.1555 0.22 Placenta
1 0.4545 0.9075 0.3845 0.0875 0.4675 0.234 0.645 0.2025 0.189 0.236 0.25 0.441 0.383 0.616 0.86 0.409 Adipose
1 0.7065 0.5805 0.1755 0.413 0.3 0.5345 0.2495 0.096 0.2635 0.3605 0.257 0.3115 0.8615 0.547 0.4755 Adrenal
1 0.9515 0.7015 0.863 0.5075 0.839 0.776 0.646 0.5895 0.837 0.7595 0.727 0.7605 0.909 0.6235 Brain
1 0.3845 0.5705 0.294 0.5765 0.4415 0.3315 0.401 0.623 0.4995 0.593 0.6985 0.745 0.5235 Breast
1 0.3765 0.2985 0.4865 0.5945 0.1925 0.1775 0.512 0.304 0.4245 0.233 0.4815 0.2295 Colon
1 0.193 0.4445 0.5025 0.361 0.5905 0.4155 0.528 0.6585 0.4885 0.649 0.699 Heart
1 0.7775 0.583 0.1425 0.059 0.499 0.1255 0.1525 0.2315 0.426 0.105 Kidney
1 0.672 0.3305 0.2215 0.7375 0.318 0.3605 0.5955 0.6735 0.2535 Liver
1 0.18 0.1625 0.661 0.2785 0.3205 0.403 0.4655 0.225 Lung
1 0.4475 0.2505 0.3255 0.5345 0.402 0.5845 0.343 LymphNode
1 0.1525 0.7175 0.8525 0.323 0.5555 0.826 Ovary
1 0.2705 0.369 0.49 0.5945 0.2525 Prostate
1 0.91 0.2475 0.585 0.799 SkeletalMuscle
1 0.3815 0.6875 0.736 Testes
1 0.7045 0.559 Thyroid
1 0.493 WhiteBloodCells
1 EdomStromalCell
Placenta
Adipose
Adrenal
Brain
Breast
Colon
Heart
Kidney
Liver
Lung
LymphNode
Ovary
Prostate
SkeletalMuscle
Testes
Thyroid
WhiteBloodCells
EdomStromalCell

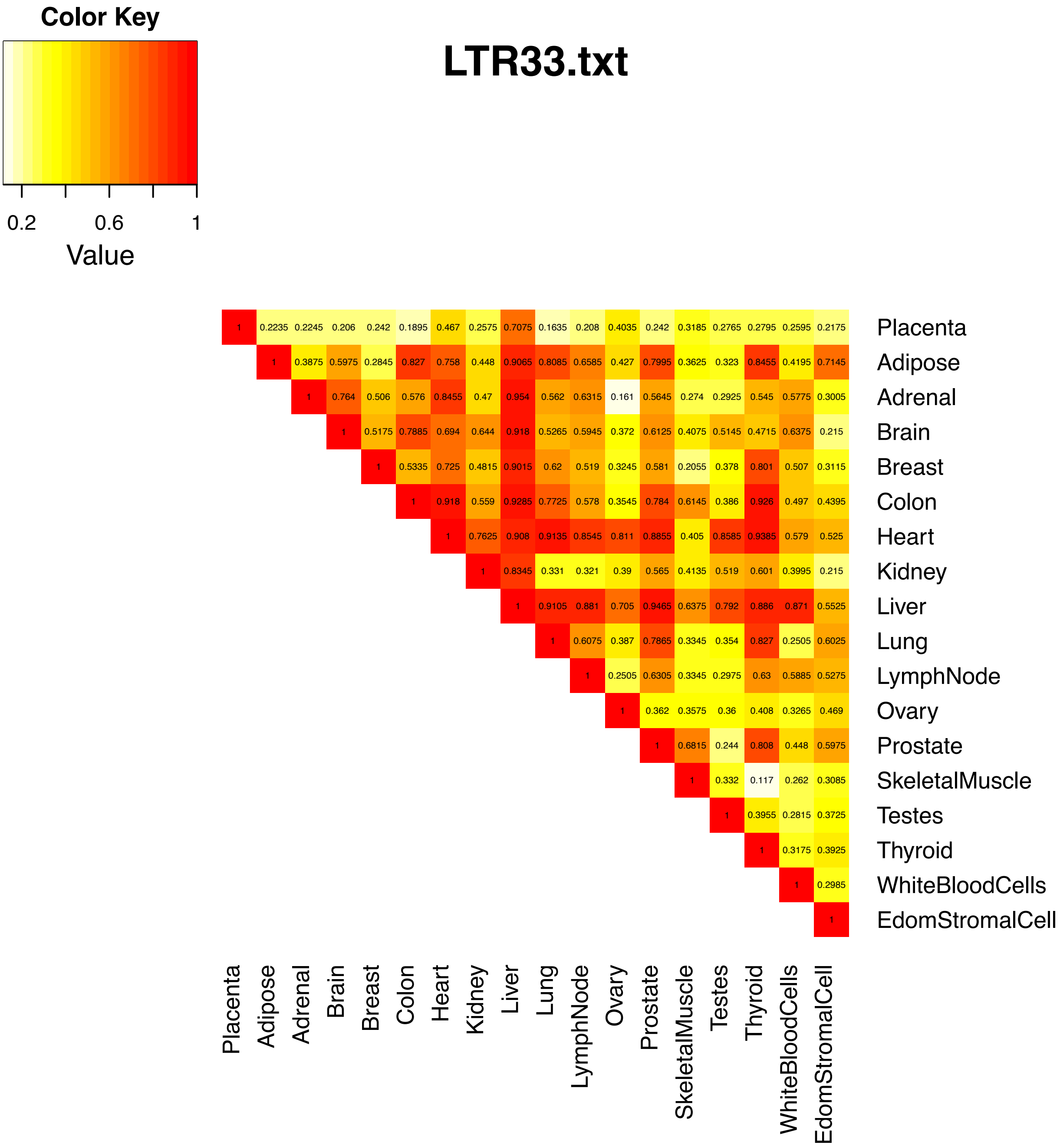

Color Key
0.2
0.6
1
Value
LTR33.txt
1 0.2235 0.2245 0.206 0.242 0.1895 0.467 0.2575 0.7075 0.1635 0.208 0.4035 0.242 0.3185 0.2765 0.2795 0.2595 0.2175
Placenta
1 0.3875 0.5975 0.2845 0.827 0.758 0.448 0.9065 0.8085 0.6585 0.427 0.7995 0.3625 0.323 0.8455 0.4195 0.7145
Adipose
1 0.764 0.506 0.576 0.8455 0.47 0.954 0.562 0.6315 0.161 0.5645 0.274 0.2925 0.545 0.5775 0.3005
Adrenal
1 0.5175 0.7885 0.694 0.644 0.918 0.5265 0.5945 0.372 0.6125 0.4075 0.5145 0.4715 0.6375 0.215
Brain
1 0.5335 0.725 0.4815 0.9015 0.62 0.519 0.3245 0.581 0.2055 0.378 0.801 0.507 0.3115
Breast
1 0.918 0.559 0.9285 0.7725 0.578 0.3545 0.784 0.6145 0.386 0.926 0.497 0.4395
Colon
1 0.7625 0.908 0.9135 0.8545 0.811 0.8855 0.405 0.8585 0.9385 0.579 0.525
Heart
1 0.8345 0.331 0.321 0.39 0.565 0.4135 0.519 0.601 0.3995 0.215
Kidney
1 0.9105 0.881 0.705 0.9465 0.6375 0.792 0.886 0.871 0.5525
Liver
1 0.6075 0.387 0.7865 0.3345 0.354 0.827 0.2505 0.6025
Lung
1 0.2505 0.6305 0.3345 0.2975 0.63 0.5885 0.5275
LymphNode
1 0.362 0.3575 0.36 0.408 0.3265 0.469
Ovary
1 0.6815 0.244 0.808 0.448 0.5975
Prostate
1 0.332 0.117 0.262 0.3085
SkeletalMuscle
1 0.3955 0.2815 0.3725
Testes
1 0.3175 0.3925
Thyroid
1 0.2985
WhiteBloodCells
1
EdomStromalCell
Placenta
Adipose
Adrenal
Brain
Breast
Colon
Heart
Kidney
Liver
Lung
LymphNode
Ovary
Prostate
SkeletalMuscle
Testes
Thyroid
WhiteBloodCells
EdomStromalCell

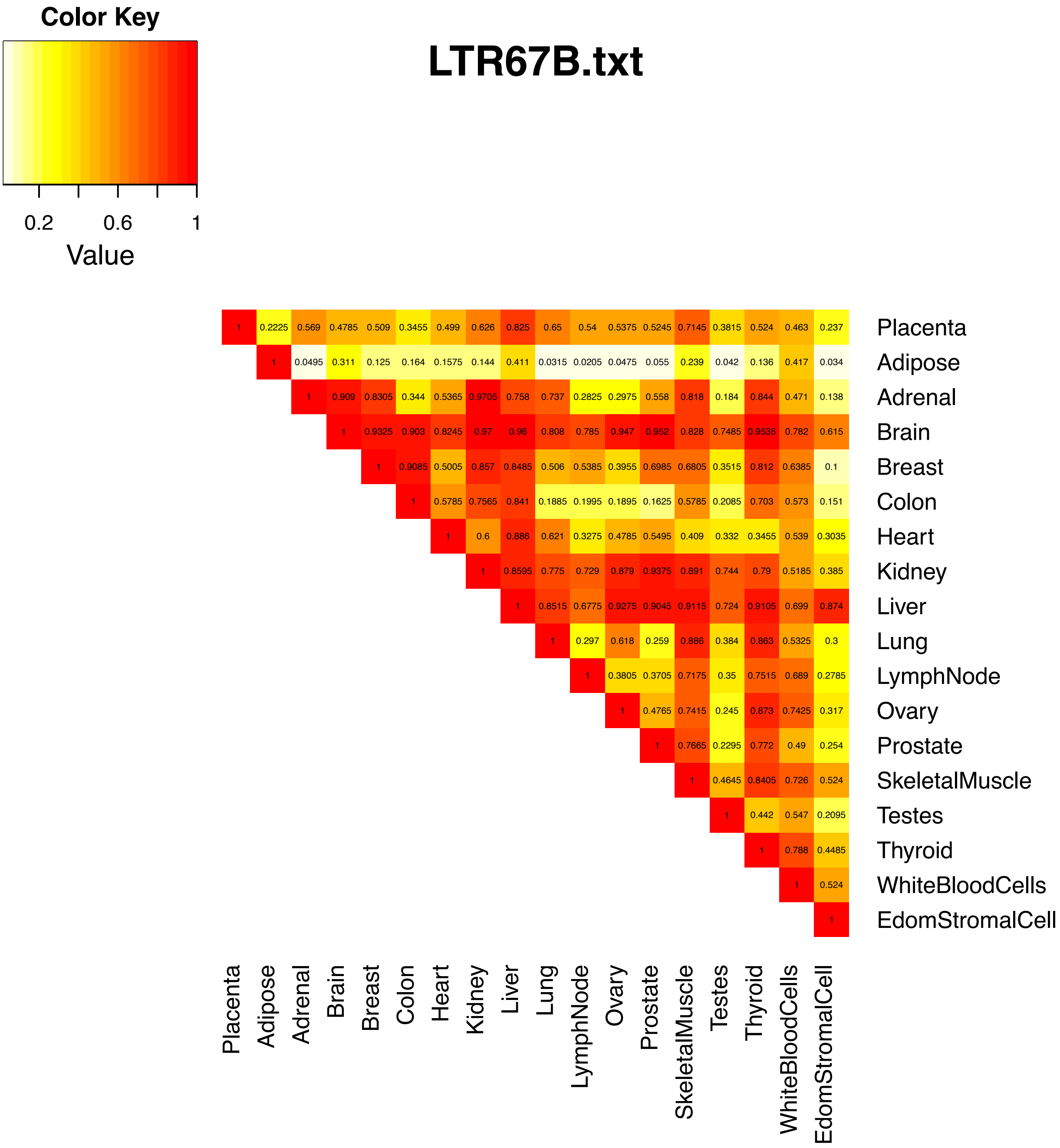
Color Key
0.2
0.6
1
Value
LTR67B.txt
Placenta 1 0.2225 0.569 0.4785 0.509 0.3455 0.499 0.626 0.825 0.65 0.54 0.5375 0.5245 0.7145 0.3815 0.524 0.463 0.237
Adipose 1 0.0495 0.311 0.125 0.164 0.1575 0.144 0.411 0.0315 0.0205 0.0475 0.055 0.239 0.042 0.136 0.417 0.034
Adrenal 1 0.909 0.8305 0.344 0.5365 0.9705 0.758 0.737 0.2825 0.2975 0.558 0.818 0.184 0.844 0.471 0.138
Brain 1 0.9325 0.903 0.8245 0.97 0.96 0.808 0.785 0.947 0.952 0.828 0.7485 0.9535 0.782 0.615
Breast 1 0.9085 0.5005 0.857 0.8485 0.506 0.5385 0.3955 0.6985 0.6805 0.3515 0.812 0.6385 0.1
Colon 1 0.5785 0.7565 0.841 0.1885 0.1995 0.1895 0.1625 0.5785 0.2085 0.703 0.573 0.151
Heart 1 0.6 0.886 0.621 0.3275 0.4785 0.5495 0.409 0.332 0.3455 0.539 0.3035
Kidney 1 0.8595 0.775 0.729 0.879 0.9375 0.891 0.744 0.79 0.5185 0.385
Liver 1 0.8515 0.6775 0.9275 0.9045 0.9115 0.724 0.9105 0.699 0.874
Lung 1 0.297 0.618 0.259 0.886 0.384 0.863 0.5325 0.3
LymphNode 1 0.3805 0.3705 0.7175 0.35 0.7515 0.689 0.2785
Ovary 1 0.4765 0.7415 0.245 0.873 0.7425 0.317
Prostate 1 0.7665 0.2295 0.772 0.49 0.254
SkeletalMuscle 1 0.4645 0.8405 0.726 0.524
Testes 1 0.442 0.547 0.2095
Thyroid 1 0.788 0.4485
WhiteBloodCells 1 0.524
EdomStromalCell 1
Placenta
Adipose
Adrenal
Brain
Breast
Colon
Heart
Kidney
Liver
Lung
LymphNode
Ovary
Prostate
SkeletalMuscle
Testes
Thyroid
WhiteBloodCells
EdomStromalCell

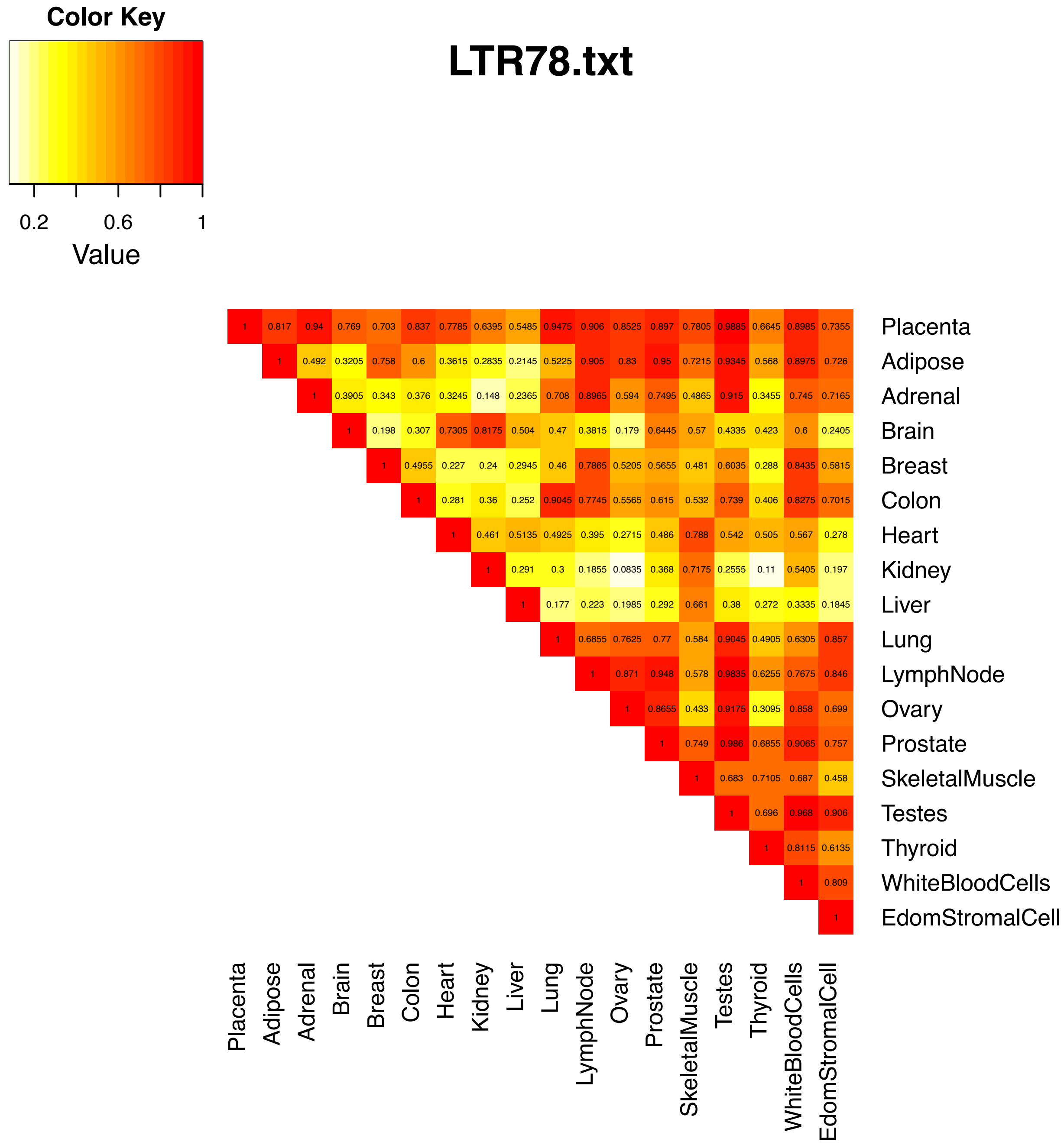
Color Key
0.2
0.6
1
Value
LTR78.txt
Placenta 1 0.817 0.94 0.769 0.703 0.837 0.7785 0.6395 0.5485 0.9475 0.906 0.8525 0.897 0.7805 0.9885 0.6645 0.8985 0.7355
Adipose 1 0.492 0.3205 0.758 0.6 0.3615 0.2835 0.2145 0.5225 0.905 0.83 0.95 0.7215 0.9345 0.568 0.8975 0.726
Adrenal 1 0.3905 0.343 0.376 0.3245 0.148 0.2365 0.708 0.8965 0.594 0.7495 0.4865 0.915 0.3455 0.745 0.7165
Brain 1 0.198 0.307 0.7305 0.8175 0.504 0.47 0.3815 0.179 0.6445 0.57 0.4335 0.423 0.6 0.2405
Breast 1 0.4955 0.227 0.24 0.2945 0.46 0.7865 0.5205 0.5655 0.481 0.6035 0.288 0.8435 0.5815
Colon 1 0.281 0.36 0.252 0.9045 0.7745 0.5565 0.615 0.532 0.739 0.406 0.8275 0.7015
Heart 1 0.461 0.5135 0.4925 0.395 0.2715 0.486 0.788 0.542 0.505 0.567 0.278
Kidney 1 0.291 0.3 0.1855 0.0835 0.368 0.7175 0.2555 0.11 0.5405 0.197
Liver 1 0.177 0.223 0.1985 0.292 0.661 0.38 0.272 0.3335 0.1845
Lung 1 0.6855 0.7625 0.77 0.584 0.9045 0.4905 0.6305 0.857
LymphNode 1 0.871 0.948 0.578 0.9835 0.6255 0.7675 0.846
Ovary 1 0.8655 0.433 0.9175 0.3095 0.858 0.699
Prostate 1 0.749 0.986 0.6855 0.9065 0.757
SkeletalMuscle 1 0.683 0.7105 0.687 0.458
Testes 1 0.696 0.968 0.906
Thyroid 1 0.8115 0.6135
WhiteBloodCells 1 0.809
EdomStromalCell 1
Placenta
Adipose
Adrenal
Brain
Breast
Colon
Heart
Kidney
Liver
Lung
LymphNode
Ovary
Prostate
SkeletalMuscle
Testes
Thyroid
WhiteBloodCells
EdomStromalCell

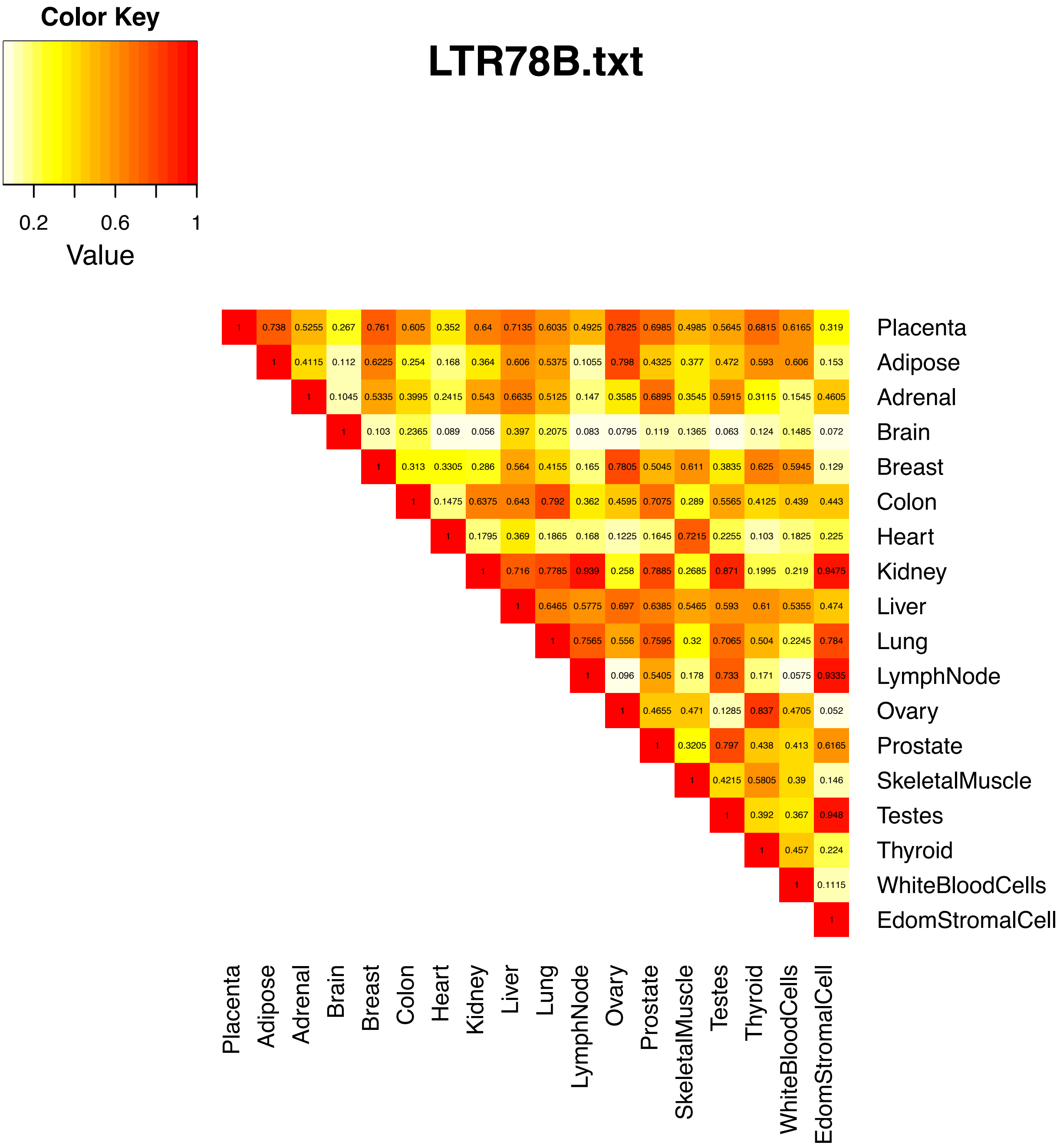
Color Key
0.2
0.6
1
Value
LTR78B.txt
Placenta Adipose Adrenal Brain Breast Colon Heart Kidney Liver Lung LymphNode Ovary Prostate SkeletalMuscle Testes Thyroid WhiteBloodCells EdomStromalCell
Placenta 1 0.738 0.5255 0.267 0.761 0.605 0.352 0.64 0.7135 0.6035 0.4925 0.7825 0.6985 0.4985 0.5645 0.6815 0.6165 0.319
Adipose 1 0.4115 0.112 0.6225 0.254 0.168 0.364 0.606 0.5375 0.1055 0.798 0.4325 0.377 0.472 0.593 0.606 0.153
Adrenal 1 0.1045 0.5335 0.3995 0.2415 0.543 0.6635 0.5125 0.147 0.3585 0.6895 0.3545 0.5915 0.3115 0.1545 0.4605
Brain 1 0.103 0.2365 0.089 0.056 0.397 0.2075 0.083 0.0795 0.119 0.1365 0.063 0.124 0.1485 0.072
Breast 1 0.313 0.3305 0.286 0.564 0.4155 0.165 0.7805 0.5045 0.611 0.3835 0.625 0.5945 0.129
Colon 1 0.1475 0.6375 0.643 0.792 0.362 0.4595 0.7075 0.289 0.5565 0.4125 0.439 0.443
Heart 1 0.1795 0.369 0.1865 0.168 0.1225 0.1645 0.7215 0.2255 0.103 0.1825 0.225
Kidney 1 0.716 0.7785 0.939 0.258 0.7885 0.2685 0.871 0.1995 0.219 0.9475
Liver 1 0.6465 0.5775 0.697 0.6385 0.5465 0.593 0.61 0.5355 0.474
Lung 1 0.7565 0.556 0.7595 0.32 0.7065 0.504 0.2245 0.784
LymphNode 1 0.096 0.5405 0.178 0.733 0.171 0.0575 0.9335
Ovary 1 0.4655 0.471 0.1285 0.837 0.4705 0.052
Prostate 1 0.3205 0.797 0.438 0.413 0.6165
SkeletalMuscle 1 0.4215 0.5805 0.39 0.146
Testes 1 0.392 0.367 0.948
Thyroid 1 0.457 0.224
WhiteBloodCells 1 0.1115
EdomStromalCell 1

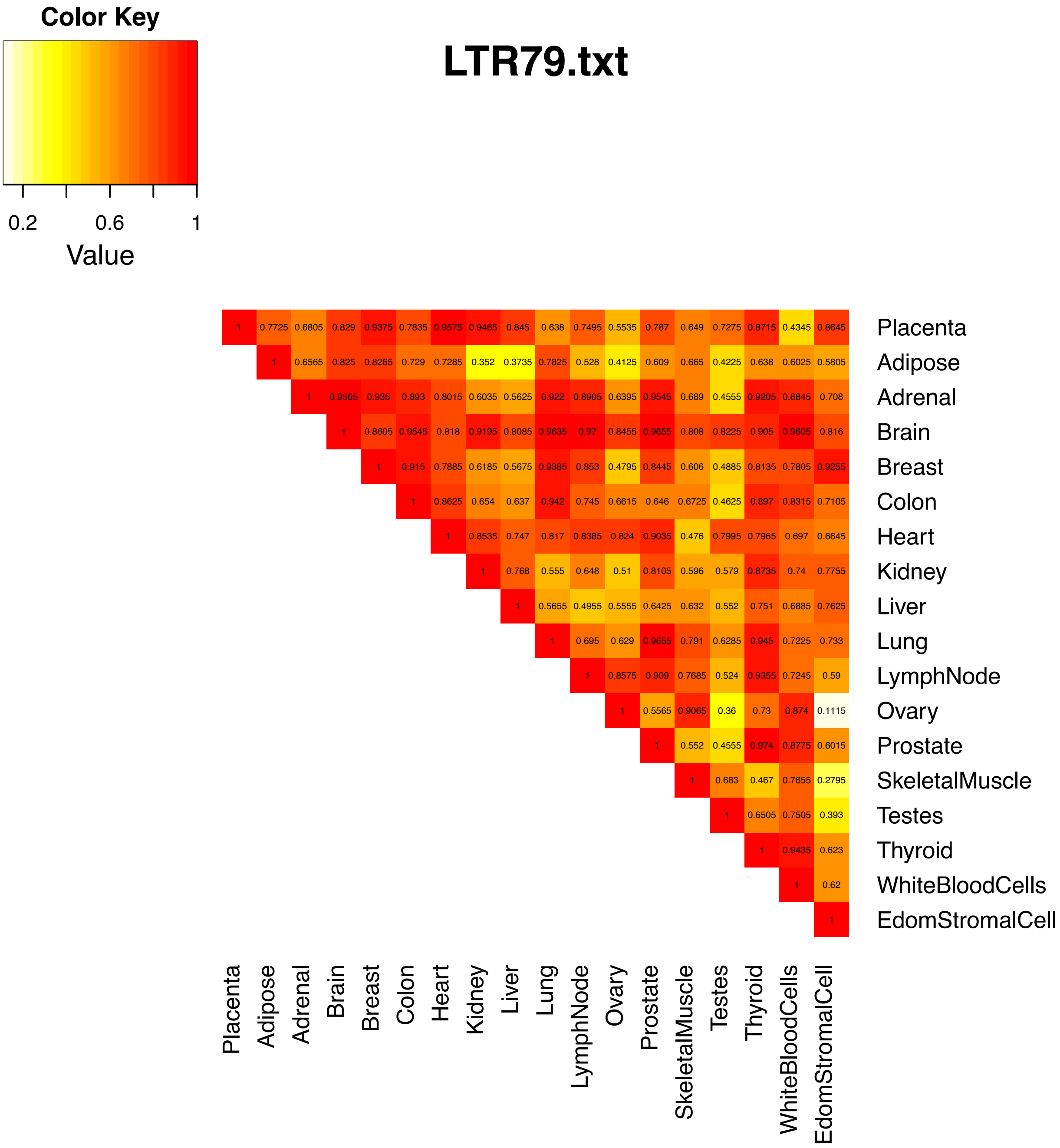

Color Key
0.2
0.6
1
Value
LTR79.txt
Placenta 1 0.7725 0.6805 0.829 0.9375 0.7835 0.9575 0.9465 0.845 0.638 0.7495 0.5535 0.787 0.649 0.7275 0.8715 0.4345 0.8645
Adipose 1 0.6565 0.825 0.8265 0.729 0.7285 0.352 0.3735 0.7825 0.528 0.4125 0.609 0.665 0.4225 0.638 0.6025 0.5805
Adrenal 1 0.9565 0.935 0.893 0.8015 0.6035 0.5625 0.922 0.8905 0.6395 0.9545 0.689 0.4555 0.9205 0.8845 0.708
Brain 1 0.8605 0.9545 0.818 0.9195 0.8085 0.9635 0.97 0.8455 0.9655 0.808 0.8225 0.905 0.9605 0.816
Breast 1 0.915 0.7885 0.6185 0.5675 0.9385 0.853 0.4795 0.8445 0.606 0.4885 0.8135 0.7805 0.9255
Colon 1 0.8625 0.654 0.637 0.942 0.745 0.6615 0.646 0.6725 0.4625 0.897 0.8315 0.7105
Heart 1 0.8535 0.747 0.817 0.8385 0.824 0.9035 0.476 0.7995 0.7965 0.697 0.6645
Kidney 1 0.768 0.555 0.648 0.51 0.8105 0.596 0.579 0.8735 0.74 0.7755
Liver 1 0.5655 0.4955 0.5555 0.6425 0.632 0.552 0.751 0.6885 0.7625
Lung 1 0.695 0.629 0.9655 0.791 0.6285 0.945 0.7225 0.733
LymphNode 1 0.8575 0.909 0.7685 0.524 0.9355 0.7245 0.59
Ovary 1 0.5565 0.9065 0.36 0.73 0.874 0.1115
Prostate 1 0.552 0.4555 0.974 0.8775 0.6015
SkeletalMuscle 1 0.683 0.467 0.7655 0.2795
Testes 1 0.6505 0.7505 0.393
Thyroid 1 0.9435 0.623
WhiteBloodCells 1 0.62
EdomStromalCell 1
Placenta
Adipose
Adrenal
Brain
Breast
Colon
Heart
Kidney
Liver
Lung
LymphNode
Ovary
Prostate
SkeletalMuscle
Testes
Thyroid
WhiteBloodCells
EdomStromalCell

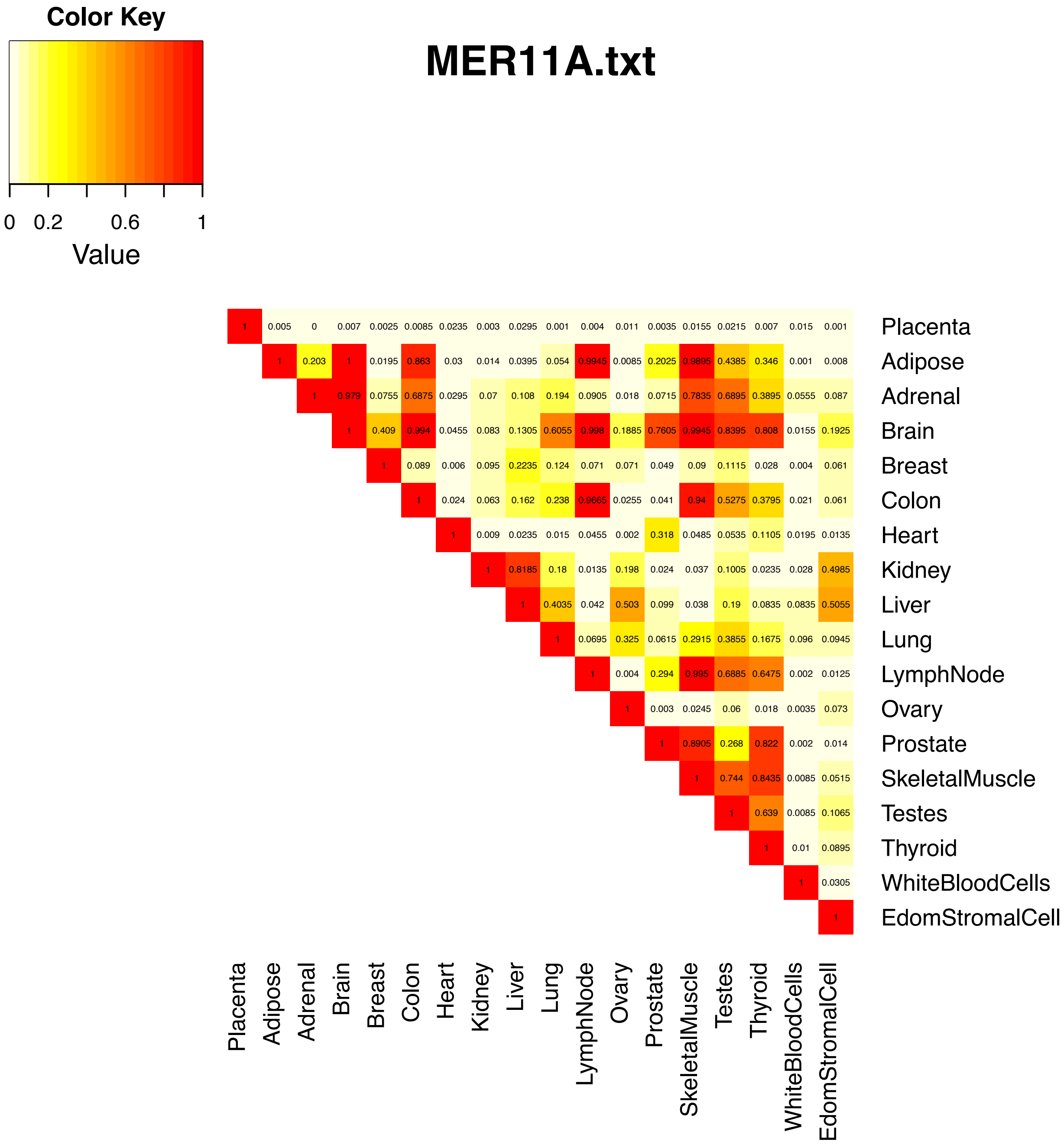

MER11A.txt
Color Key
0 0.2 0.6 1
Value
Placenta 1 0.005 0 0.007 0.0025 0.0085 0.0235 0.003 0.0295 0.001 0.004 0.011 0.0035 0.0155 0.0215 0.007 0.015 0.001
Adipose 1 0.203 1 0.0195 0.863 0.03 0.014 0.0395 0.054 0.9945 0.0085 0.2025 0.9895 0.4385 0.346 0.001 0.008
Adrenal 1 0.979 0.0755 0.6875 0.0295 0.07 0.108 0.194 0.0905 0.018 0.0715 0.7835 0.6895 0.3895 0.0555 0.087
Brain 1 0.409 0.994 0.0455 0.083 0.1305 0.6055 0.998 0.1885 0.7605 0.9945 0.8395 0.808 0.0155 0.1925
Breast 1 0.089 0.006 0.095 0.2235 0.124 0.071 0.071 0.049 0.09 0.1115 0.028 0.004 0.061
Colon 1 0.024 0.063 0.162 0.238 0.9665 0.0255 0.041 0.94 0.5275 0.3795 0.021 0.061
Heart 1 0.009 0.0235 0.015 0.0455 0.002 0.318 0.0485 0.0535 0.1105 0.0195 0.0135
Kidney 1 0.8185 0.18 0.0135 0.198 0.024 0.037 0.1005 0.0235 0.028 0.4985
Liver 1 0.4035 0.042 0.503 0.099 0.038 0.19 0.0835 0.0835 0.5055
Lung 1 0.0695 0.325 0.0615 0.2915 0.3855 0.1675 0.096 0.0945
LymphNode 1 0.004 0.294 0.995 0.6885 0.6475 0.002 0.0125
Ovary 1 0.003 0.0245 0.06 0.018 0.0035 0.073
Prostate 1 0.8905 0.268 0.822 0.002 0.014
SkeletalMuscle 1 0.744 0.8435 0.0085 0.0515
Testes 1 0.639 0.0085 0.1065
Thyroid 1 0.01 0.0895
WhiteBloodCells 1 0.0305
EdomStromalCell 1
Placenta Adipose Adrenal Brain Breast Colon Heart Kidney Liver Lung LymphNode Ovary Prostate SkeletalMuscle Testes Thyroid WhiteBloodCells EdomStromalCell

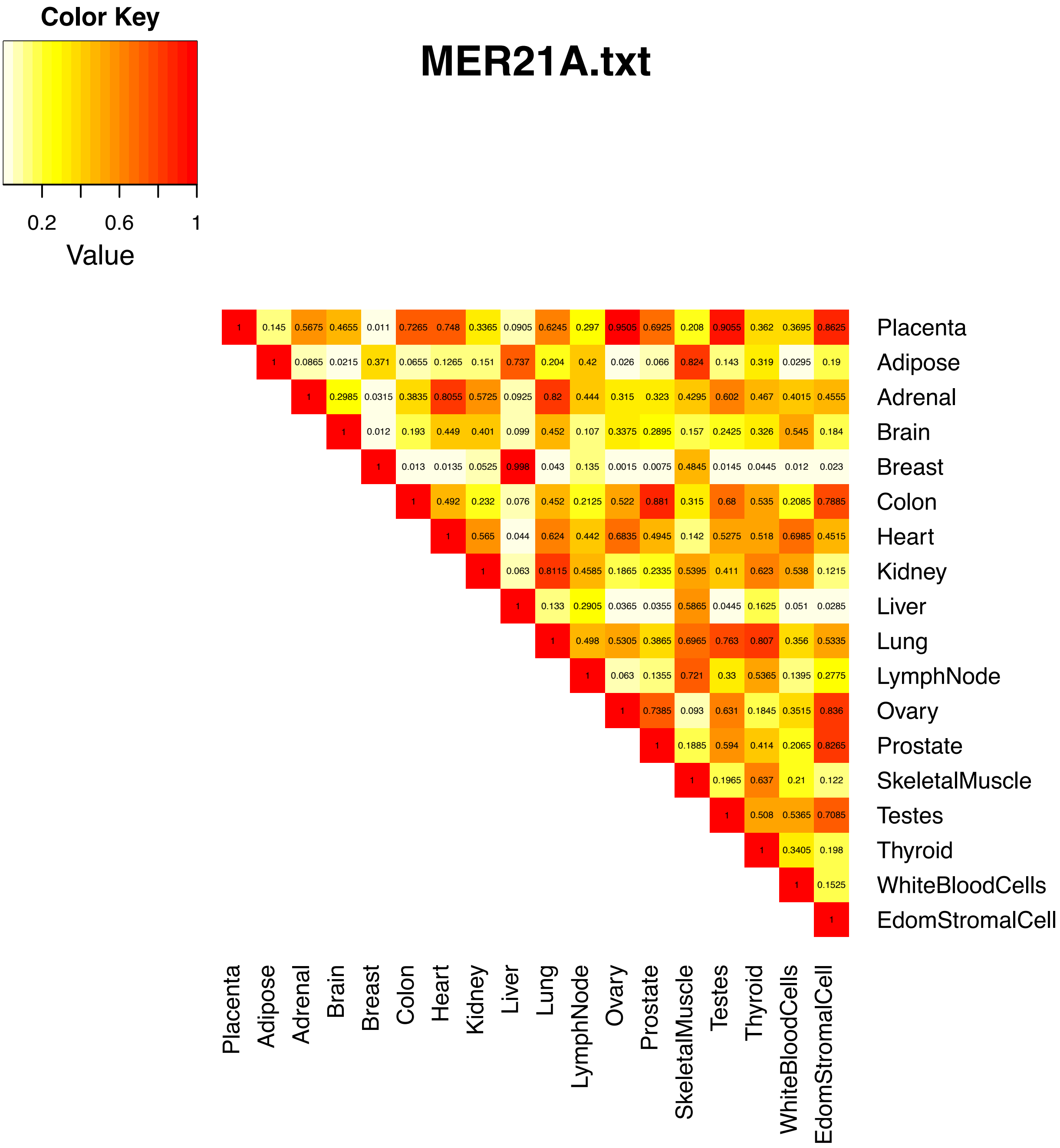
Color Key
0.2 0.6 1
Value
MER21A.txt
Placenta: 1 0.145 0.5675 0.4655 0.011 0.7265 0.748 0.3365 0.0905 0.6245 0.297 0.9505 0.6925 0.208 0.9055 0.362 0.3695 0.8625
Adipose: 1 0.0865 0.0215 0.371 0.0655 0.1265 0.151 0.737 0.204 0.42 0.026 0.066 0.824 0.143 0.319 0.0295 0.19
Adrenal: 1 0.2985 0.0315 0.3835 0.8055 0.5725 0.0925 0.82 0.444 0.315 0.323 0.4295 0.602 0.467 0.4015 0.4555
Brain: 1 0.012 0.193 0.449 0.401 0.099 0.452 0.107 0.3375 0.2895 0.157 0.2425 0.326 0.545 0.184
Breast: 1 0.013 0.0135 0.0525 0.998 0.043 0.135 0.0015 0.0075 0.4845 0.0145 0.0445 0.012 0.023
Colon: 1 0.492 0.232 0.076 0.452 0.2125 0.522 0.881 0.315 0.68 0.535 0.2085 0.7885
Heart: 1 0.565 0.044 0.624 0.442 0.6835 0.4945 0.142 0.5275 0.518 0.6985 0.4515
Kidney: 1 0.063 0.8115 0.4585 0.1865 0.2335 0.5395 0.411 0.623 0.538 0.1215
Liver: 1 0.133 0.2905 0.0365 0.0355 0.5865 0.0445 0.1625 0.051 0.0285
Lung: 1 0.498 0.5305 0.3865 0.6965 0.763 0.807 0.356 0.5335
LymphNode: 1 0.063 0.1355 0.721 0.33 0.5365 0.1395 0.2775
Ovary: 1 0.7385 0.093 0.631 0.1845 0.3515 0.836
Prostate: 1 0.1885 0.594 0.414 0.2065 0.8265
SkeletalMuscle: 1 0.1965 0.637 0.21 0.122
Testes: 1 0.508 0.5365 0.7085
Thyroid: 1 0.3405 0.198
WhiteBloodCells: 1 0.1525
EdomStromalCell: 1
Placenta Adipose Adrenal Brain Breast Colon Heart Kidney Liver Lung LymphNode Ovary Prostate SkeletalMuscle Testes Thyroid WhiteBloodCells EdomStromalCell

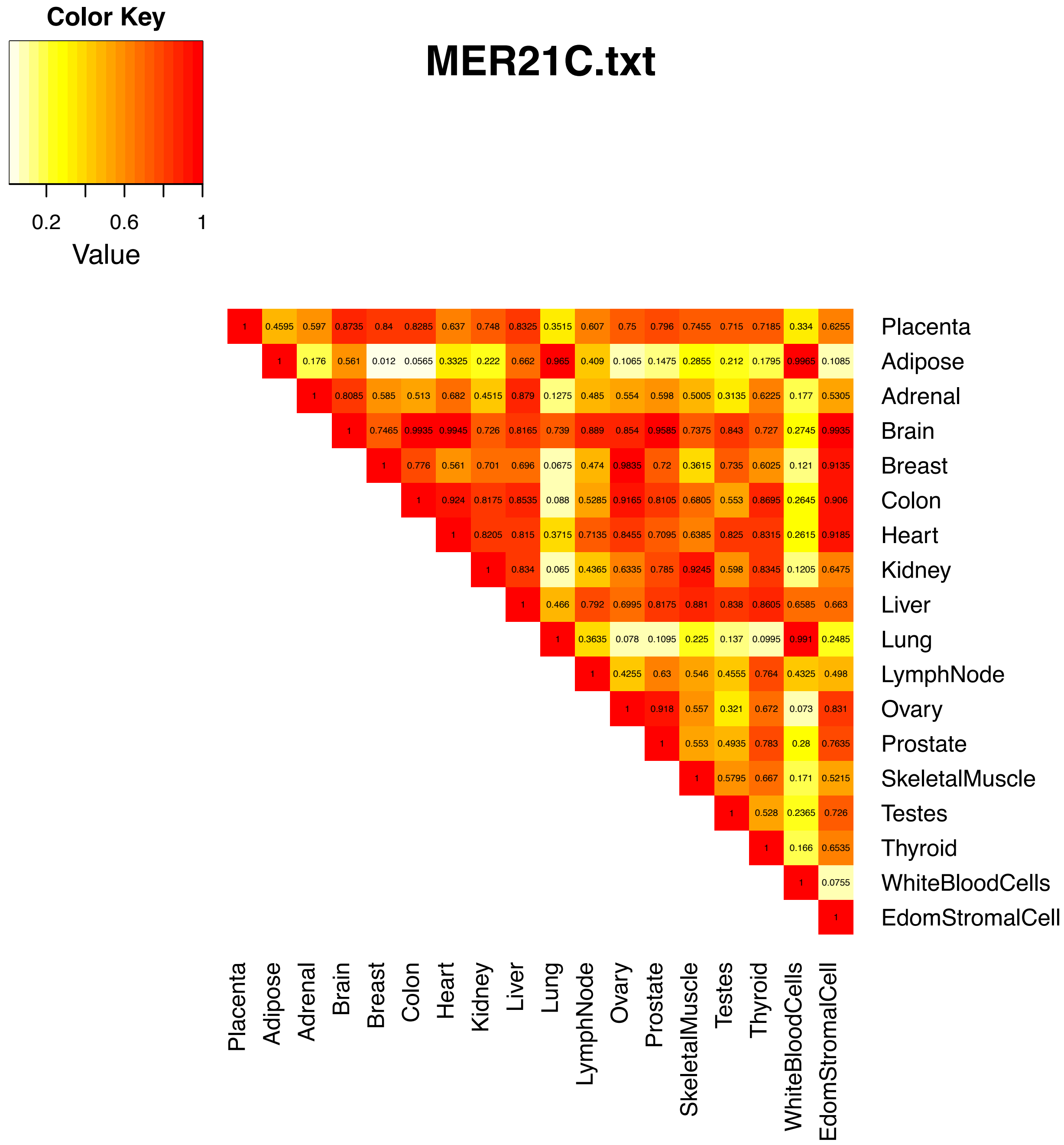

Color Key
0.2
0.6
1
Value
MER21C.txt
Placenta 1 0.4595 0.597 0.8735 0.84 0.8285 0.637 0.748 0.8325 0.3515 0.607 0.75 0.796 0.7455 0.715 0.7185 0.334 0.6255
Adipose 1 0.176 0.561 0.012 0.0565 0.3325 0.222 0.662 0.965 0.409 0.1065 0.1475 0.2855 0.212 0.1795 0.9965 0.1085
Adrenal 1 0.8085 0.585 0.513 0.682 0.4515 0.879 0.1275 0.485 0.554 0.598 0.5005 0.3135 0.6225 0.177 0.5305
Brain 1 0.7465 0.9935 0.9945 0.726 0.8165 0.739 0.889 0.854 0.9585 0.7375 0.843 0.727 0.2745 0.9935
Breast 1 0.776 0.561 0.701 0.696 0.0675 0.474 0.9835 0.72 0.3615 0.735 0.6025 0.121 0.9135
Colon 1 0.924 0.8175 0.8535 0.088 0.5285 0.9165 0.8105 0.6805 0.553 0.8695 0.2645 0.906
Heart 1 0.8205 0.815 0.3715 0.7135 0.8455 0.7095 0.6385 0.825 0.8315 0.2615 0.9185
Kidney 1 0.834 0.065 0.4365 0.6335 0.785 0.9245 0.598 0.8345 0.1205 0.6475
Liver 1 0.466 0.792 0.6995 0.8175 0.881 0.838 0.8605 0.6585 0.663
Lung 1 0.3635 0.078 0.1095 0.225 0.137 0.0995 0.991 0.2485
LymphNode 1 0.4255 0.63 0.546 0.4555 0.764 0.4325 0.498
Ovary 1 0.918 0.557 0.321 0.672 0.073 0.831
Prostate 1 0.553 0.4935 0.783 0.28 0.7635
SkeletalMuscle 1 0.5795 0.667 0.171 0.5215
Testes 1 0.528 0.2365 0.726
Thyroid 1 0.166 0.6535
WhiteBloodCells 1 0.0755
EdomStromalCell 1
Placenta
Adipose
Adrenal
Brain
Breast
Colon
Heart
Kidney
Liver
Lung
LymphNode
Ovary
Prostate
SkeletalMuscle
Testes
Thyroid
WhiteBloodCells
EdomStromalCell

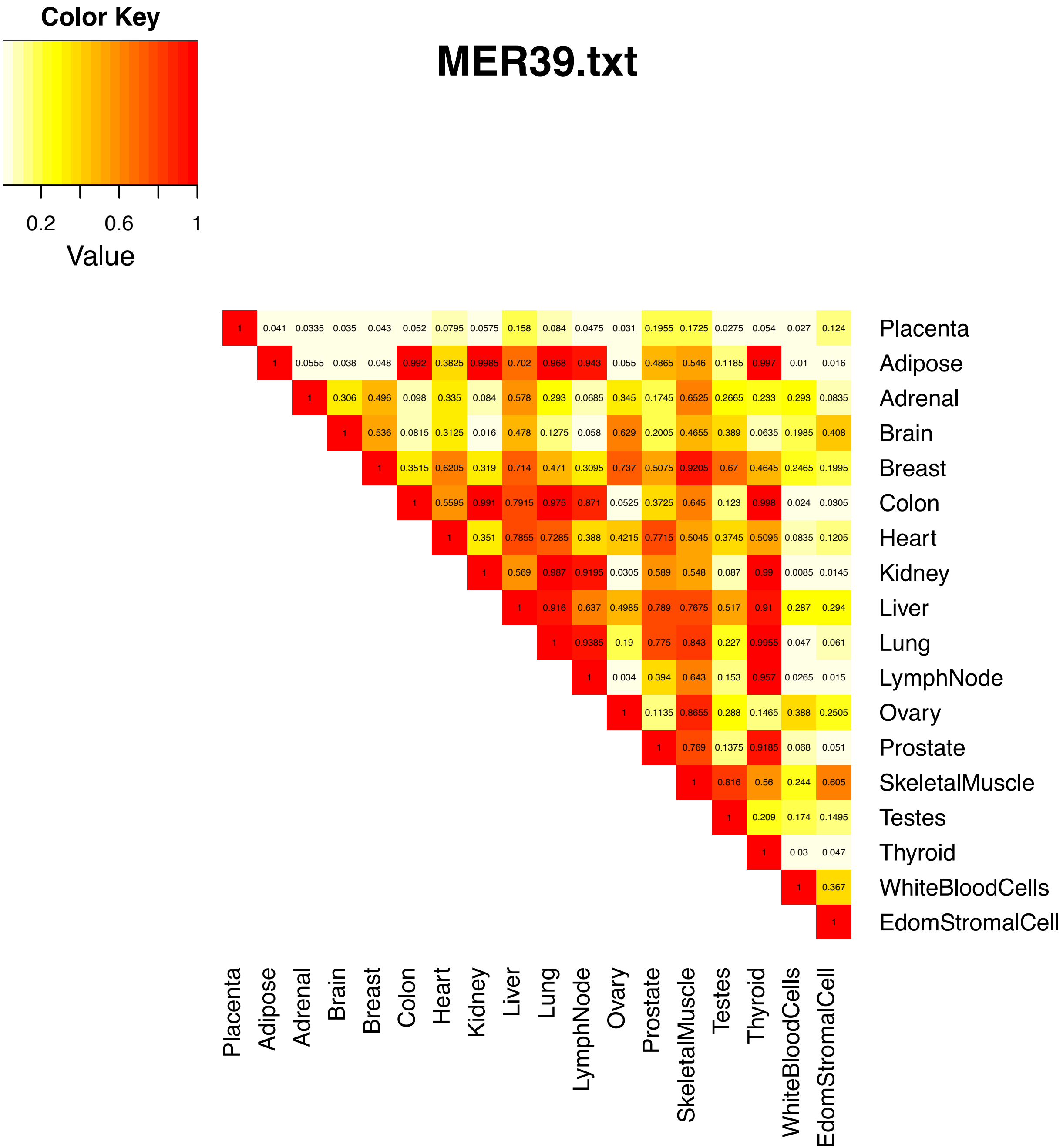
Color Key
0.2 0.6 1
Value
MER39.txt
Placenta 1 0.041 0.0335 0.035 0.043 0.052 0.0795 0.0575 0.158 0.084 0.0475 0.031 0.1955 0.1725 0.0275 0.054 0.027 0.124
Adipose 1 0.0555 0.038 0.048 0.992 0.3825 0.9985 0.702 0.968 0.943 0.055 0.4865 0.546 0.1185 0.997 0.01 0.016
Adrenal 1 0.306 0.496 0.098 0.335 0.084 0.578 0.293 0.0685 0.345 0.1745 0.6525 0.2665 0.233 0.293 0.0835
Brain 1 0.536 0.0815 0.3125 0.016 0.478 0.1275 0.058 0.629 0.2005 0.4655 0.389 0.0635 0.1985 0.408
Breast 1 0.3515 0.6205 0.319 0.714 0.471 0.3095 0.737 0.5075 0.9205 0.67 0.4645 0.2465 0.1995
Colon 1 0.5595 0.991 0.7915 0.975 0.871 0.0525 0.3725 0.645 0.123 0.998 0.024 0.0305
Heart 1 0.351 0.7855 0.7285 0.388 0.4215 0.7715 0.5045 0.3745 0.5095 0.0835 0.1205
Kidney 1 0.569 0.987 0.9195 0.0305 0.589 0.548 0.087 0.99 0.0085 0.0145
Liver 1 0.916 0.637 0.4985 0.789 0.7675 0.517 0.91 0.287 0.294
Lung 1 0.9385 0.19 0.775 0.843 0.227 0.9955 0.047 0.061
LymphNode 1 0.034 0.394 0.643 0.153 0.957 0.0265 0.015
Ovary 1 0.1135 0.8655 0.288 0.1465 0.388 0.2505
Prostate 1 0.769 0.1375 0.9185 0.068 0.051
SkeletalMuscle 1 0.816 0.56 0.244 0.605
Testes 1 0.209 0.174 0.1495
Thyroid 1 0.03 0.047
WhiteBloodCells 1 0.367
EdomStromalCell 1
Placenta Adipose Adrenal Brain Breast Colon Heart Kidney Liver Lung LymphNode Ovary Prostate SkeletalMuscle Testes Thyroid WhiteBloodCells EdomStromalCell

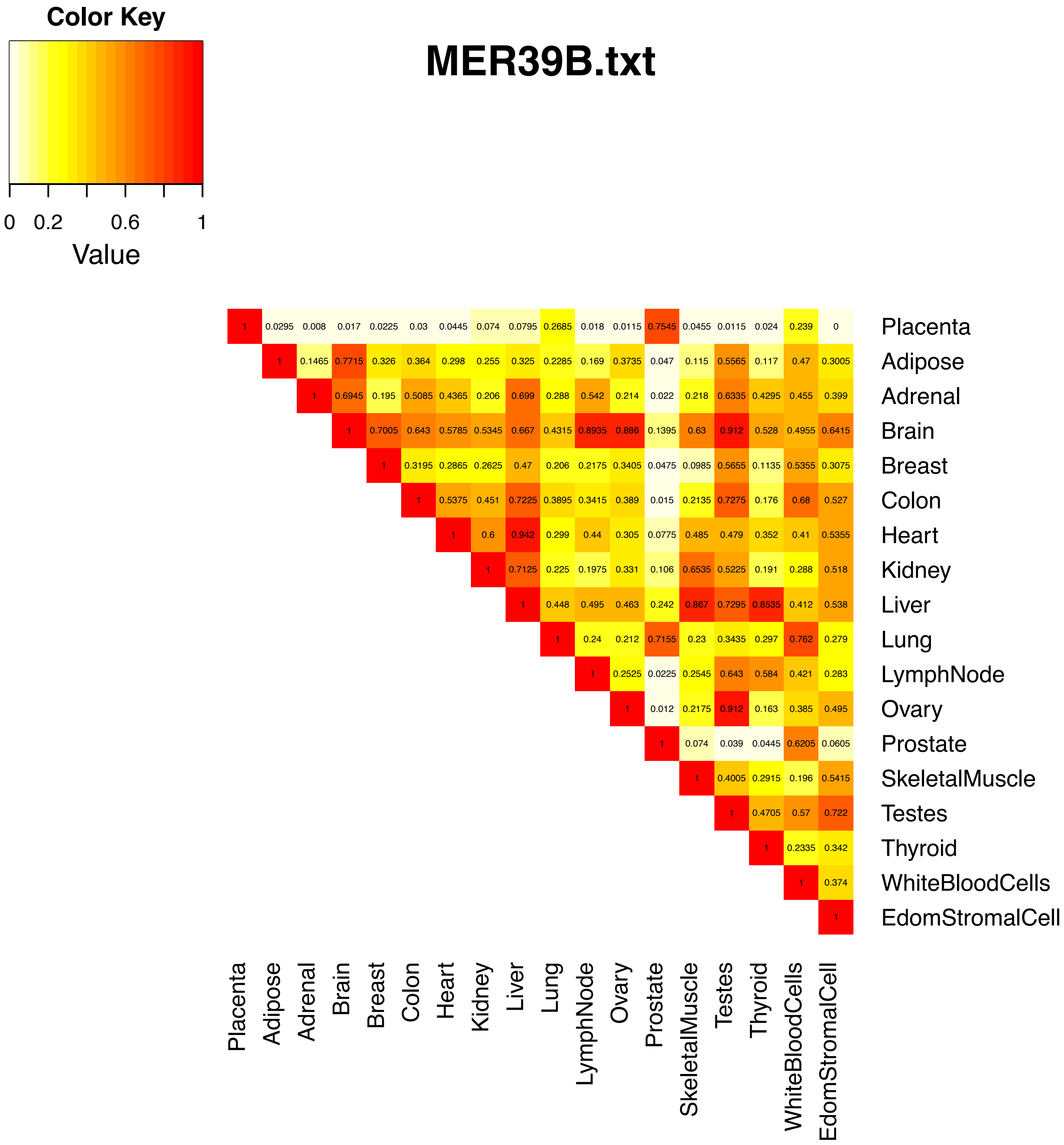

MER39B.txt
Color Key
0 0.2 0.6 1
Value
Placenta: 1 0.0295 0.008 0.017 0.0225 0.03 0.0445 0.074 0.0795 0.2685 0.018 0.0115 0.7545 0.0455 0.0115 0.024 0.239 0
Adipose: 1 0.1465 0.7715 0.326 0.364 0.298 0.255 0.325 0.2285 0.169 0.3735 0.047 0.115 0.5565 0.117 0.47 0.3005
Adrenal: 1 0.6945 0.195 0.5085 0.4365 0.206 0.699 0.288 0.542 0.214 0.022 0.218 0.6335 0.4295 0.455 0.399
Brain: 1 0.7005 0.643 0.5785 0.5345 0.667 0.4315 0.8935 0.886 0.1395 0.63 0.912 0.528 0.4955 0.6415
Breast: 1 0.3195 0.2865 0.2625 0.47 0.206 0.2175 0.3405 0.0475 0.0985 0.5655 0.1135 0.5355 0.3075
Colon: 1 0.5375 0.451 0.7225 0.3895 0.3415 0.389 0.015 0.2135 0.7275 0.176 0.68 0.527
Heart: 1 0.6 0.942 0.299 0.44 0.305 0.0775 0.485 0.479 0.352 0.41 0.5355
Kidney: 1 0.7125 0.225 0.1975 0.331 0.106 0.6535 0.5225 0.191 0.288 0.518
Liver: 1 0.448 0.495 0.463 0.242 0.867 0.7295 0.8535 0.412 0.538
Lung: 1 0.24 0.212 0.7155 0.23 0.3435 0.297 0.762 0.279
LymphNode: 1 0.2525 0.0225 0.2545 0.643 0.584 0.421 0.283
Ovary: 1 0.012 0.2175 0.912 0.163 0.385 0.495
Prostate: 1 0.074 0.039 0.0445 0.6205 0.0605
SkeletalMuscle: 1 0.4005 0.2915 0.196 0.5415
Testes: 1 0.4705 0.57 0.722
Thyroid: 1 0.2335 0.342
WhiteBloodCells: 1 0.374
EdomStromalCell: 1
Placenta Adipose Adrenal Brain Breast Colon Heart Kidney Liver Lung LymphNode Ovary Prostate SkeletalMuscle Testes Thyroid WhiteBloodCells EdomStromalCell

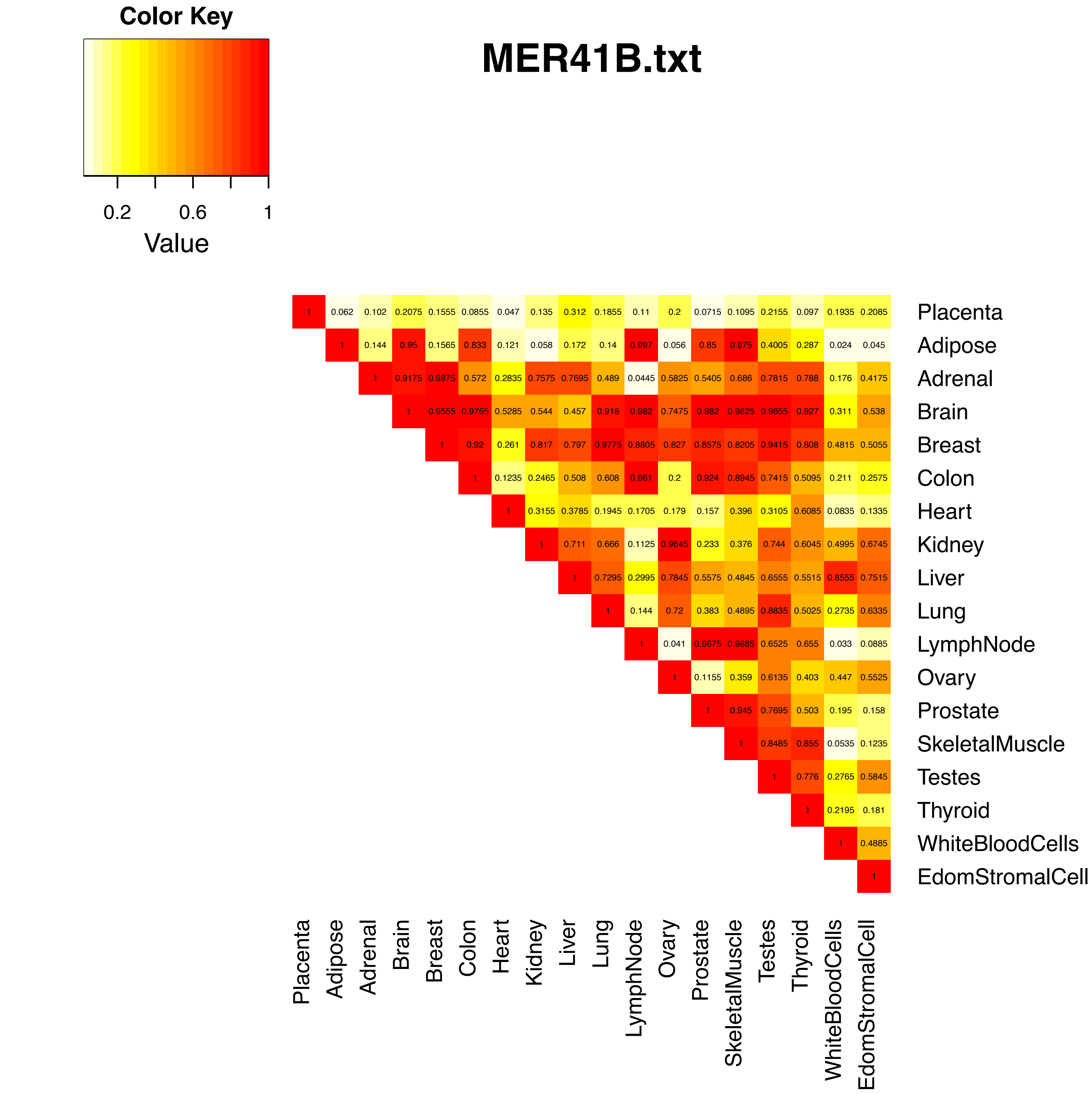
Color Key
0.2
0.6
1
Value
MER41B.txt
Placenta: 1 0.062 0.102 0.2075 0.1555 0.0855 0.047 0.135 0.312 0.1855 0.11 0.2 0.0715 0.1095 0.2155 0.097 0.1935 0.2085
Adipose: 1 0.144 0.95 0.1565 0.833 0.121 0.058 0.172 0.14 0.997 0.056 0.85 0.975 0.4005 0.287 0.024 0.045
Adrenal: 1 0.9175 0.9875 0.572 0.2835 0.7575 0.7695 0.489 0.0445 0.5825 0.5405 0.686 0.7815 0.788 0.176 0.4175
Brain: 1 0.9555 0.9765 0.5285 0.544 0.457 0.916 0.982 0.7475 0.982 0.9825 0.9655 0.927 0.311 0.538
Breast: 1 0.92 0.261 0.817 0.797 0.9775 0.8805 0.827 0.8575 0.8205 0.9415 0.808 0.4815 0.5055
Colon: 1 0.1235 0.2465 0.508 0.608 0.961 0.2 0.924 0.8945 0.7415 0.5095 0.211 0.2575
Heart: 1 0.3155 0.3785 0.1945 0.1705 0.179 0.157 0.396 0.3105 0.6085 0.0835 0.1335
Kidney: 1 0.711 0.666 0.1125 0.9645 0.233 0.376 0.744 0.6045 0.4995 0.6745
Liver: 1 0.7295 0.2995 0.7845 0.5575 0.4845 0.6555 0.5515 0.8555 0.7515
Lung: 1 0.144 0.72 0.383 0.4895 0.8835 0.5025 0.2735 0.6335
LymphNode: 1 0.041 0.9675 0.9885 0.6525 0.655 0.033 0.0885
Ovary: 1 0.1155 0.359 0.6135 0.403 0.447 0.5525
Prostate: 1 0.945 0.7695 0.503 0.195 0.158
SkeletalMuscle: 1 0.8485 0.855 0.0535 0.1235
Testes: 1 0.776 0.2765 0.5845
Thyroid: 1 0.2195 0.181
WhiteBloodCells: 1 0.4885
EdomStromalCell: 1
Placenta
Adipose
Adrenal
Brain
Breast
Colon
Heart
Kidney
Liver
Lung
LymphNode
Ovary
Prostate
SkeletalMuscle
Testes
Thyroid
WhiteBloodCells
EdomStromalCell

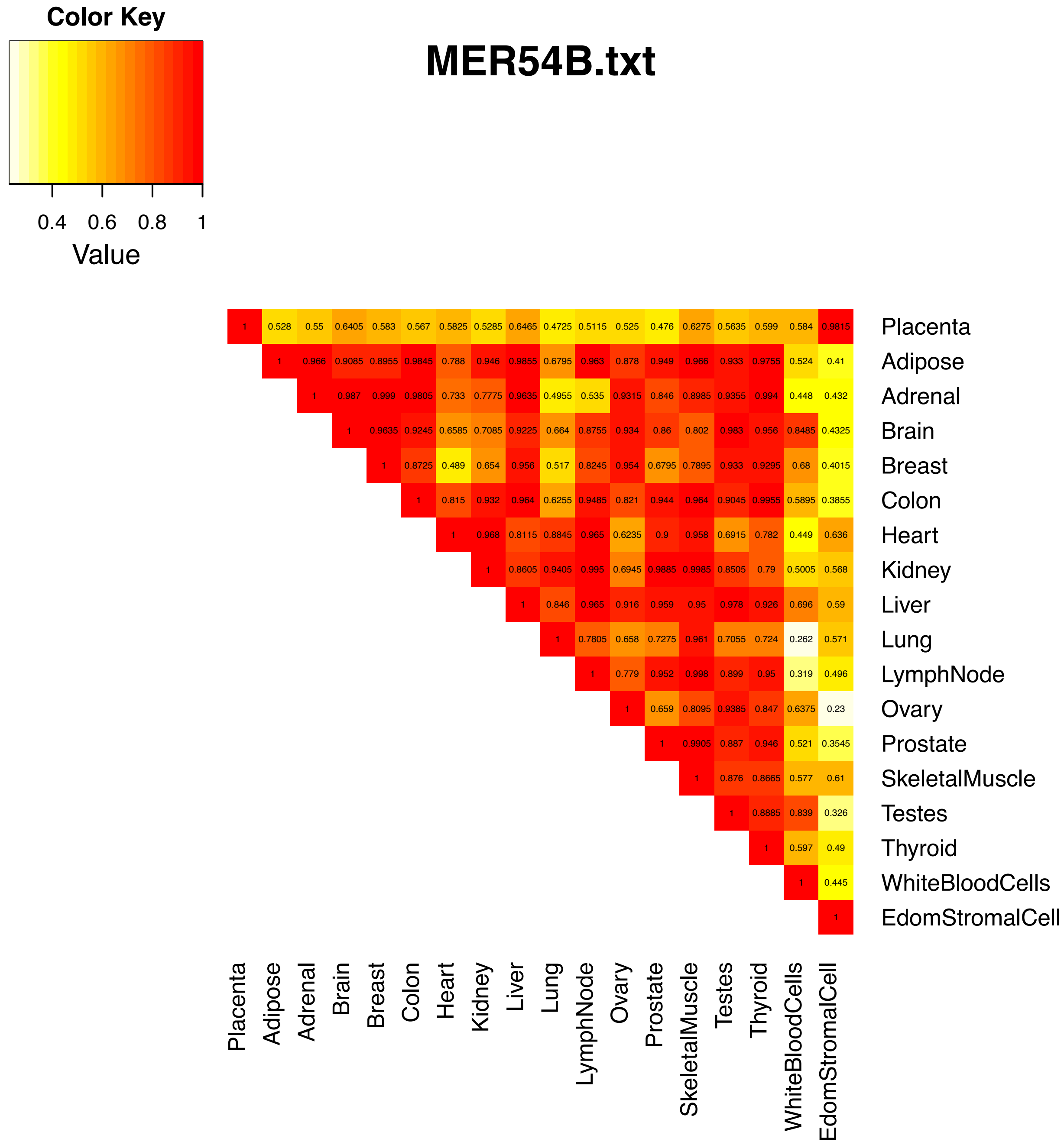

Color Key
0.4 0.6 0.8 1
Value
MER54B.txt
Placenta
Adipose
Adrenal
Brain
Breast
Colon
Heart
Kidney
Liver
Lung
LymphNode
Ovary
Prostate
SkeletalMuscle
Testes
Thyroid
WhiteBloodCells
EdomStromalCell
1 0.528 0.55 0.6405 0.583 0.567 0.5825 0.5285 0.6465 0.4725 0.5115 0.525 0.476 0.6275 0.5635 0.599 0.584 0.9815
1 0.966 0.9085 0.8955 0.9845 0.788 0.946 0.9855 0.6795 0.963 0.878 0.949 0.966 0.933 0.9755 0.524 0.41
1 0.987 0.999 0.9805 0.733 0.7775 0.9635 0.4955 0.535 0.9315 0.846 0.8985 0.9355 0.994 0.448 0.432
1 0.9635 0.9245 0.6585 0.7085 0.9225 0.664 0.8755 0.934 0.86 0.802 0.983 0.956 0.8485 0.4325
1 0.8725 0.489 0.654 0.956 0.517 0.8245 0.954 0.6795 0.7895 0.933 0.9295 0.68 0.4015
1 0.815 0.932 0.964 0.6255 0.9485 0.821 0.944 0.964 0.9045 0.9955 0.5895 0.3855
1 0.968 0.8115 0.8845 0.965 0.6235 0.9 0.958 0.6915 0.782 0.449 0.636
1 0.8605 0.9405 0.995 0.6945 0.9885 0.9985 0.8505 0.79 0.5005 0.568
1 0.846 0.965 0.916 0.959 0.95 0.978 0.926 0.696 0.59
1 0.7805 0.658 0.7275 0.961 0.7055 0.724 0.262 0.571
1 0.779 0.952 0.998 0.899 0.95 0.319 0.496
1 0.659 0.8095 0.9385 0.847 0.6375 0.23
1 0.9905 0.887 0.946 0.521 0.3545
1 0.876 0.8665 0.577 0.61
1 0.8885 0.839 0.326
1 0.597 0.49
1 0.445
1

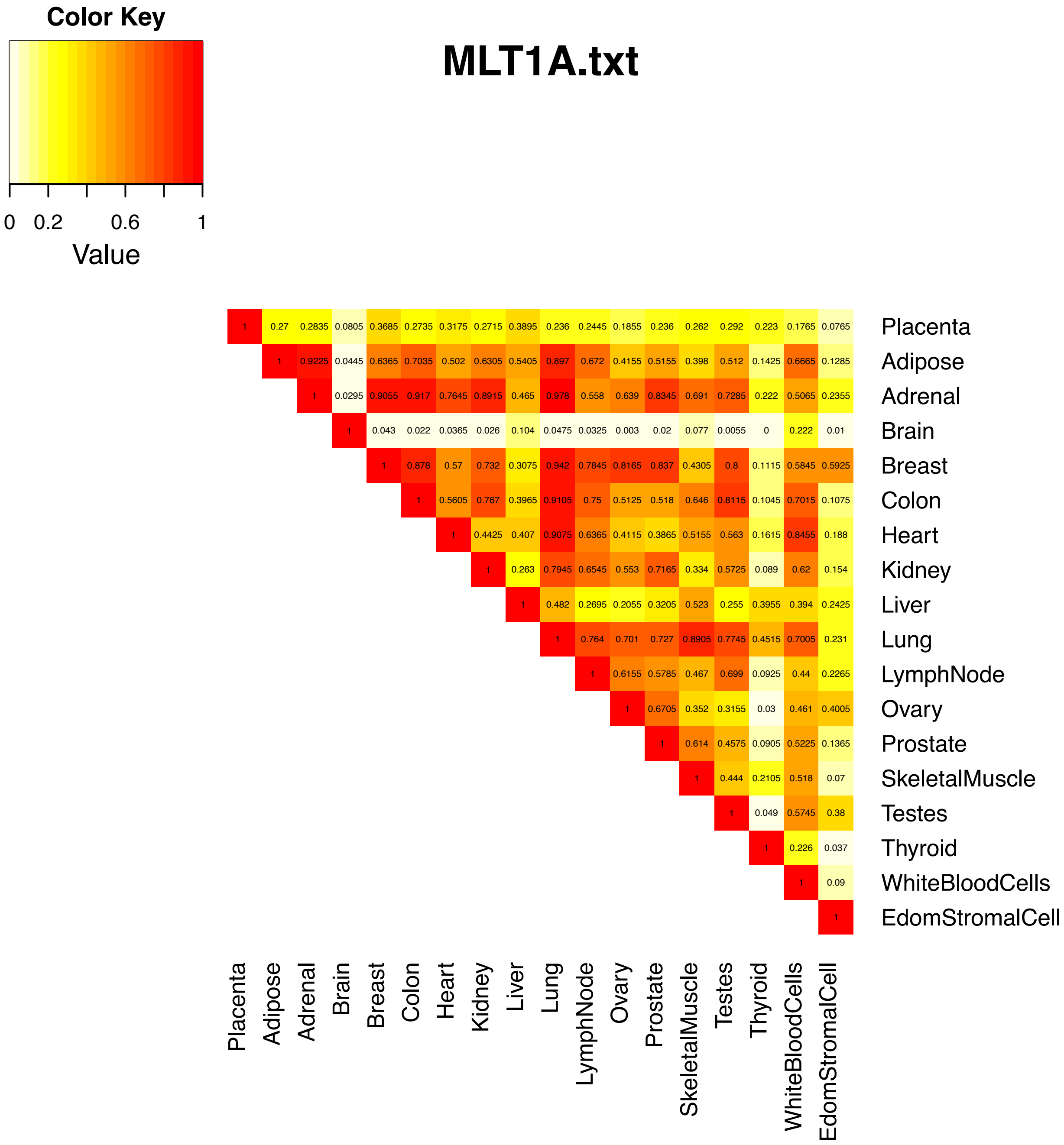
Color Key
0 0.2 0.6 1
Value
MLT1A.txt
1 0.27 0.2835 0.0805 0.3685 0.2735 0.3175 0.2715 0.3895 0.236 0.2445 0.1855 0.236 0.262 0.292 0.223 0.1765 0.0765 Placenta
1 0.9225 0.0445 0.6365 0.7035 0.502 0.6305 0.5405 0.897 0.672 0.4155 0.5155 0.398 0.512 0.1425 0.6665 0.1285 Adipose
1 0.0295 0.9055 0.917 0.7645 0.8915 0.465 0.978 0.558 0.639 0.8345 0.691 0.7285 0.222 0.5065 0.2355 Adrenal
1 0.043 0.022 0.0365 0.026 0.104 0.0475 0.0325 0.003 0.02 0.077 0.0055 0 0.222 0.01 Brain
1 0.878 0.57 0.732 0.3075 0.942 0.7845 0.8165 0.837 0.4305 0.8 0.1115 0.5845 0.5925 Breast
1 0.5605 0.767 0.3965 0.9105 0.75 0.5125 0.518 0.646 0.8115 0.1045 0.7015 0.1075 Colon
1 0.4425 0.407 0.9075 0.6365 0.4115 0.3865 0.5155 0.563 0.1615 0.8455 0.188 Heart
1 0.263 0.7945 0.6545 0.553 0.7165 0.334 0.5725 0.089 0.62 0.154 Kidney
1 0.482 0.2695 0.2055 0.3205 0.523 0.255 0.3955 0.394 0.2425 Liver
1 0.764 0.701 0.727 0.8905 0.7745 0.4515 0.7005 0.231 Lung
1 0.6155 0.5785 0.467 0.699 0.0925 0.44 0.2265 LymphNode
1 0.6705 0.352 0.3155 0.03 0.461 0.4005 Ovary
1 0.614 0.4575 0.0905 0.5225 0.1365 Prostate
1 0.444 0.2105 0.518 0.07 SkeletalMuscle
1 0.049 0.5745 0.38 Testes
1 0.226 0.037 Thyroid
1 0.09 WhiteBloodCells
1 EdomStromalCell
Placenta Adipose Adrenal Brain Breast Colon Heart Kidney Liver Lung LymphNode Ovary Prostate SkeletalMuscle Testes Thyroid WhiteBloodCells EdomStromalCell

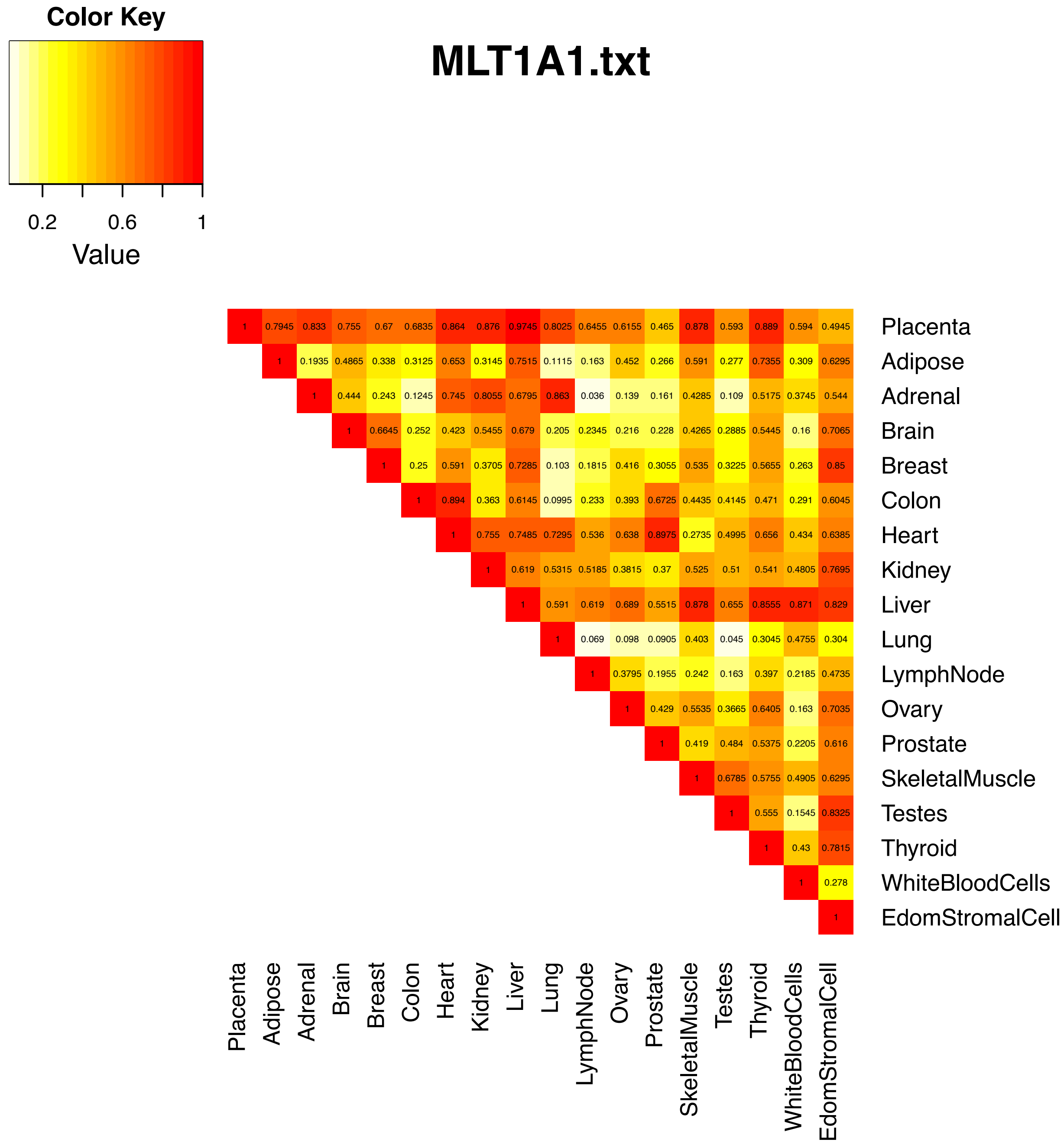
Color Key
0.2
0.6
1
Value
MLT1A1.txt
1 0.7945 0.833 0.755 0.67 0.6835 0.864 0.876 0.9745 0.8025 0.6455 0.6155 0.465 0.878 0.593 0.889 0.594 0.4945 Placenta
1 0.1935 0.4865 0.338 0.3125 0.653 0.3145 0.7515 0.1115 0.163 0.452 0.266 0.591 0.277 0.7355 0.309 0.6295 Adipose
1 0.444 0.243 0.1245 0.745 0.8055 0.6795 0.863 0.036 0.139 0.161 0.4285 0.109 0.5175 0.3745 0.544 Adrenal
1 0.6645 0.252 0.423 0.5455 0.679 0.205 0.2345 0.216 0.228 0.4265 0.2885 0.5445 0.16 0.7065 Brain
1 0.25 0.591 0.3705 0.7285 0.103 0.1815 0.416 0.3055 0.535 0.3225 0.5655 0.263 0.85 Breast
1 0.894 0.363 0.6145 0.0995 0.233 0.393 0.6725 0.4435 0.4145 0.471 0.291 0.6045 Colon
1 0.755 0.7485 0.7295 0.536 0.638 0.8975 0.2735 0.4995 0.656 0.434 0.6385 Heart
1 0.619 0.5315 0.5185 0.3815 0.37 0.525 0.51 0.541 0.4805 0.7695 Kidney
1 0.591 0.619 0.689 0.5515 0.878 0.655 0.8555 0.871 0.829 Liver
1 0.069 0.098 0.0905 0.403 0.045 0.3045 0.4755 0.304 Lung
1 0.3795 0.1955 0.242 0.163 0.397 0.2185 0.4735 LymphNode
1 0.429 0.5535 0.3665 0.6405 0.163 0.7035 Ovary
1 0.419 0.484 0.5375 0.2205 0.616 Prostate
1 0.6785 0.5755 0.4905 0.6295 SkeletalMuscle
1 0.555 0.1545 0.8325 Testes
1 0.43 0.7815 Thyroid
1 0.278 WhiteBloodCells
1 EdomStromalCell
Placenta
Adipose
Adrenal
Brain
Breast
Colon
Heart
Kidney
Liver
Lung
LymphNode
Ovary
Prostate
SkeletalMuscle
Testes
Thyroid
WhiteBloodCells
EdomStromalCell

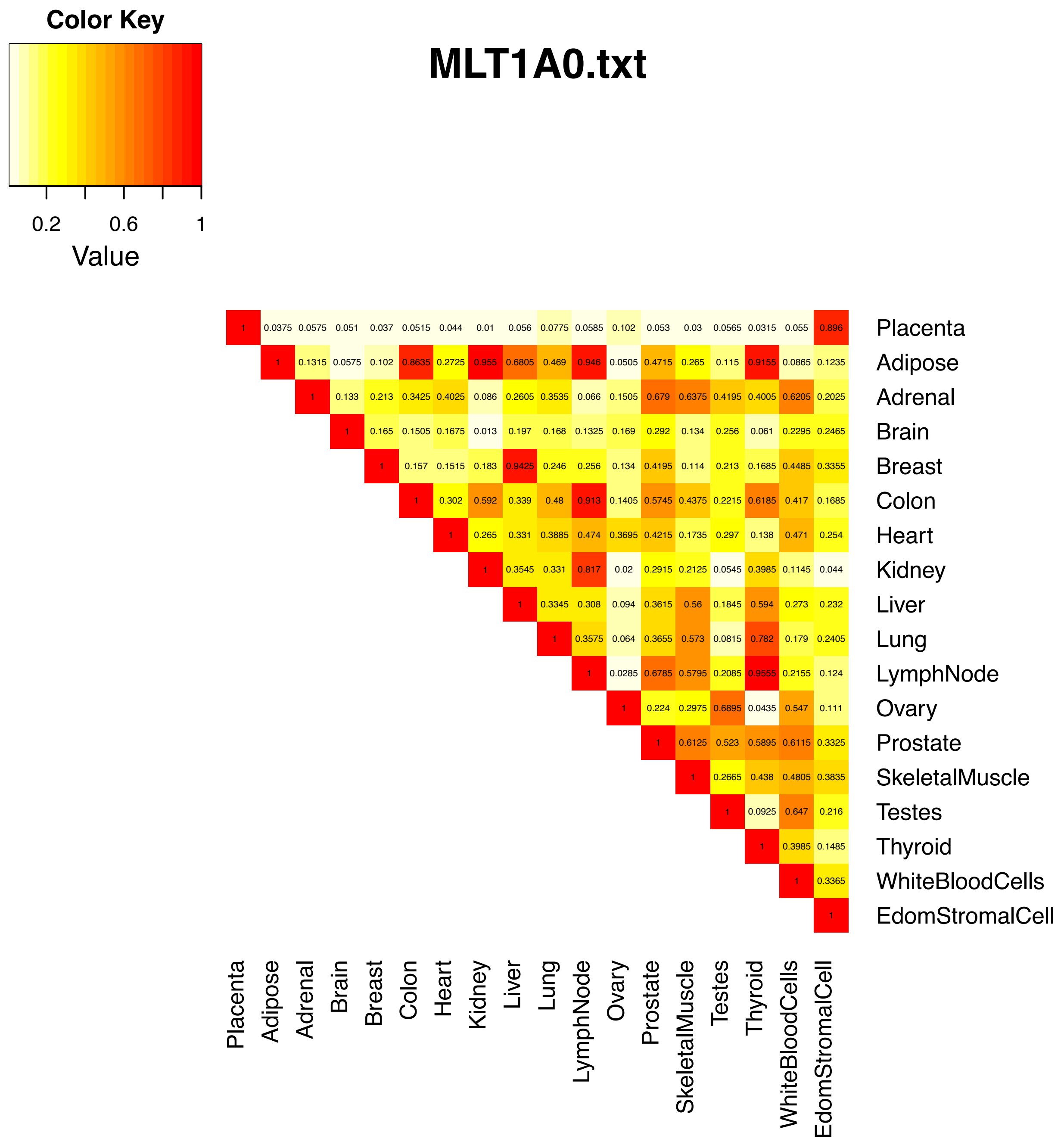
Color Key
0.2 0.6 1
Value
MLT1A0.txt
Placenta: 1 0.0375 0.0575 0.051 0.037 0.0515 0.044 0.01 0.056 0.0775 0.0585 0.102 0.053 0.03 0.0565 0.0315 0.055 0.896
Adipose: 1 0.1315 0.0575 0.102 0.8635 0.2725 0.955 0.6805 0.469 0.946 0.0505 0.4715 0.265 0.115 0.9155 0.0865 0.1235
Adrenal: 1 0.133 0.213 0.3425 0.4025 0.086 0.2605 0.3535 0.066 0.1505 0.679 0.6375 0.4195 0.4005 0.6205 0.2025
Brain: 1 0.165 0.1505 0.1675 0.013 0.197 0.168 0.1325 0.169 0.292 0.134 0.256 0.061 0.2295 0.2465
Breast: 1 0.157 0.1515 0.183 0.9425 0.246 0.256 0.134 0.4195 0.114 0.213 0.1685 0.4485 0.3355
Colon: 1 0.302 0.592 0.339 0.48 0.913 0.1405 0.5745 0.4375 0.2215 0.6185 0.417 0.1685
Heart: 1 0.265 0.331 0.3885 0.474 0.3695 0.4215 0.1735 0.297 0.138 0.471 0.254
Kidney: 1 0.3545 0.331 0.817 0.02 0.2915 0.2125 0.0545 0.3985 0.1145 0.044
Liver: 1 0.3345 0.308 0.094 0.3615 0.56 0.1845 0.594 0.273 0.232
Lung: 1 0.3575 0.064 0.3655 0.573 0.0815 0.782 0.179 0.2405
LymphNode: 1 0.0285 0.6785 0.5795 0.2085 0.9555 0.2155 0.124
Ovary: 1 0.224 0.2975 0.6895 0.0435 0.547 0.111
Prostate: 1 0.6125 0.523 0.5895 0.6115 0.3325
SkeletalMuscle: 1 0.2665 0.438 0.4805 0.3835
Testes: 1 0.0925 0.647 0.216
Thyroid: 1 0.3985 0.1485
WhiteBloodCells: 1 0.3365
EdomStromalCell: 1
Placenta Adipose Adrenal Brain Breast Colon Heart Kidney Liver Lung LymphNode Ovary Prostate SkeletalMuscle Testes Thyroid WhiteBloodCells EdomStromalCell

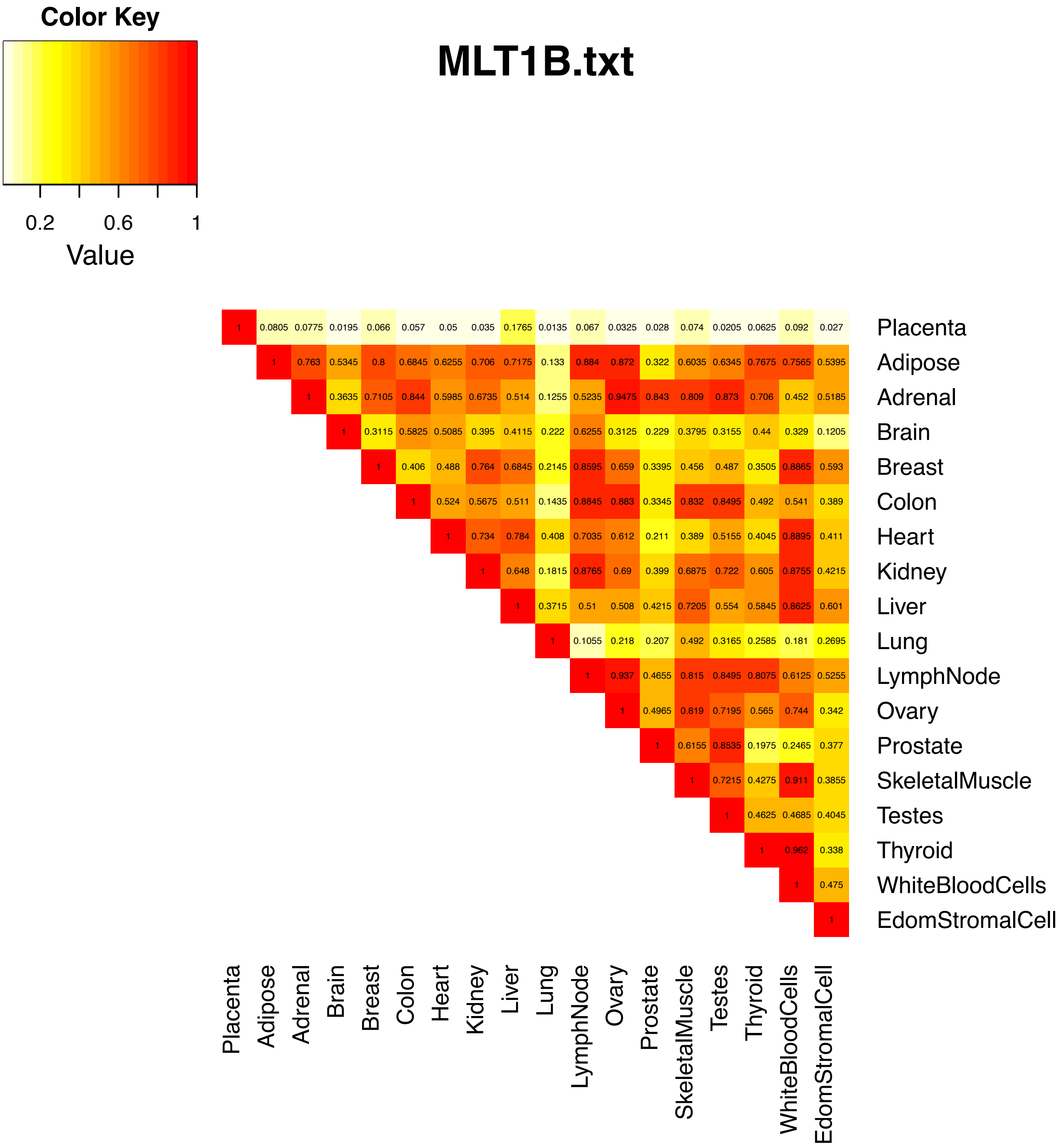

MLT1B.txt
Color Key
0.2
0.6
1
Value
Placenta 1 0.0805 0.0775 0.0195 0.066 0.057 0.05 0.035 0.1765 0.0135 0.067 0.0325 0.028 0.074 0.0205 0.0625 0.092 0.027
Adipose 1 0.763 0.5345 0.8 0.6845 0.6255 0.706 0.7175 0.133 0.884 0.872 0.322 0.6035 0.6345 0.7675 0.7565 0.5395
Adrenal 1 0.3635 0.7105 0.844 0.5985 0.6735 0.514 0.1255 0.5235 0.9475 0.843 0.809 0.873 0.706 0.452 0.5185
Brain 1 0.3115 0.5825 0.5085 0.395 0.4115 0.222 0.6255 0.3125 0.229 0.3795 0.3155 0.44 0.329 0.1205
Breast 1 0.406 0.488 0.764 0.6845 0.2145 0.8595 0.659 0.3395 0.456 0.487 0.3505 0.8865 0.593
Colon 1 0.524 0.5675 0.511 0.1435 0.8845 0.883 0.3345 0.832 0.8495 0.492 0.541 0.389
Heart 1 0.734 0.784 0.408 0.7035 0.612 0.211 0.389 0.5155 0.4045 0.8895 0.411
Kidney 1 0.648 0.1815 0.8765 0.69 0.399 0.6875 0.722 0.605 0.8755 0.4215
Liver 1 0.3715 0.51 0.508 0.4215 0.7205 0.554 0.5845 0.8625 0.601
Lung 1 0.1055 0.218 0.207 0.492 0.3165 0.2585 0.181 0.2695
LymphNode 1 0.937 0.4655 0.815 0.8495 0.8075 0.6125 0.5255
Ovary 1 0.4965 0.819 0.7195 0.565 0.744 0.342
Prostate 1 0.6155 0.8535 0.1975 0.2465 0.377
SkeletalMuscle 1 0.7215 0.4275 0.911 0.3855
Testes 1 0.4625 0.4685 0.4045
Thyroid 1 0.962 0.338
WhiteBloodCells 1 0.475
EdomStromalCell 1
Placenta
Adipose
Adrenal
Brain
Breast
Colon
Heart
Kidney
Liver
Lung
LymphNode
Ovary
Prostate
SkeletalMuscle
Testes
Thyroid
WhiteBloodCells
EdomStromalCell

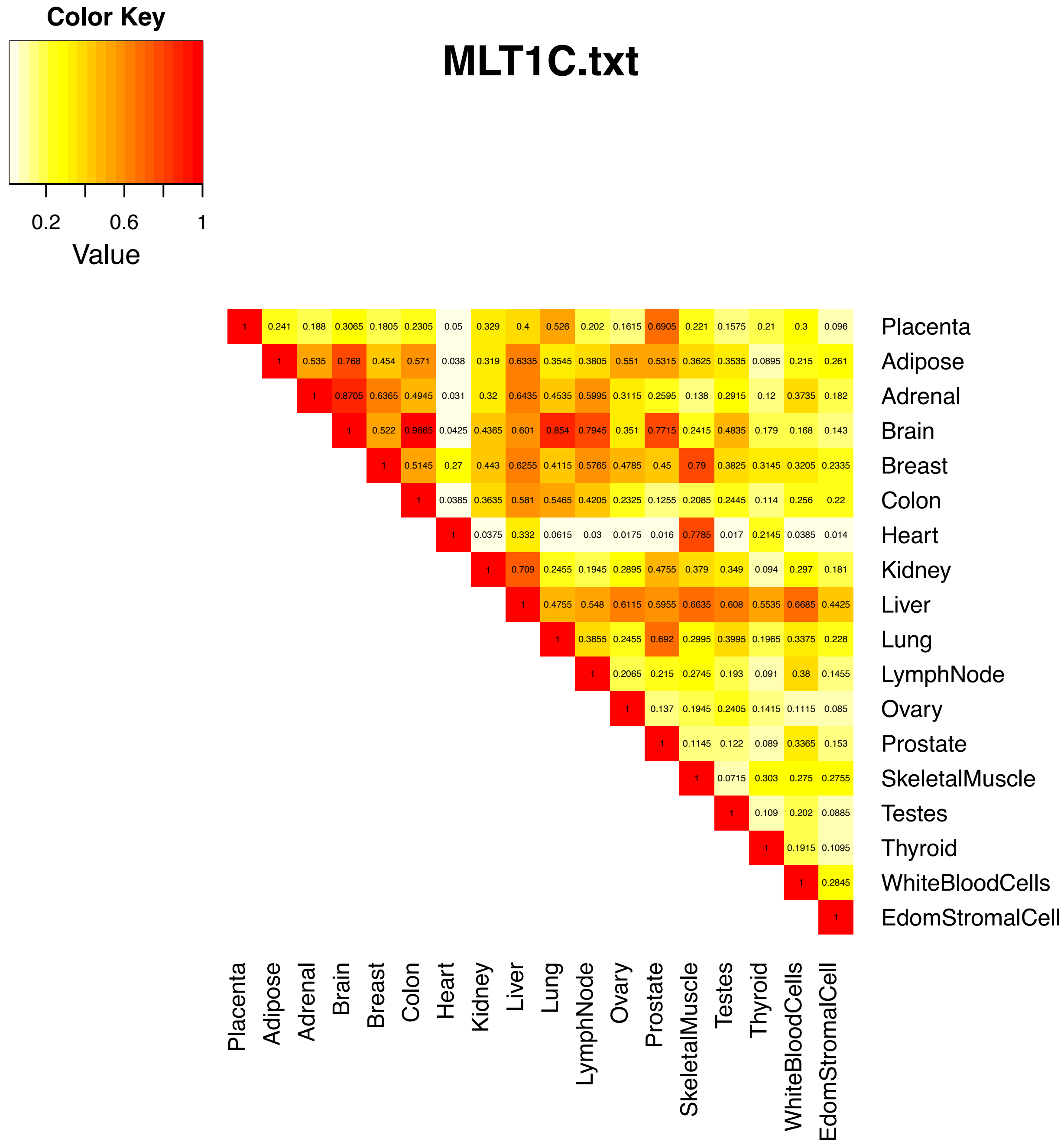

Color Key
0.2
0.6
1
Value
MLT1C.txt
Placenta: 1 0.241 0.188 0.3065 0.1805 0.2305 0.05 0.329 0.4 0.526 0.202 0.1615 0.6905 0.221 0.1575 0.21 0.3 0.096
Adipose: 1 0.535 0.768 0.454 0.571 0.038 0.319 0.6335 0.3545 0.3805 0.551 0.5315 0.3625 0.3535 0.0895 0.215 0.261
Adrenal: 1 0.8705 0.6365 0.4945 0.031 0.32 0.6435 0.4535 0.5995 0.3115 0.2595 0.138 0.2915 0.12 0.3735 0.182
Brain: 1 0.522 0.9665 0.0425 0.4365 0.601 0.854 0.7945 0.351 0.7715 0.2415 0.4835 0.179 0.168 0.143
Breast: 1 0.5145 0.27 0.443 0.6255 0.4115 0.5765 0.4785 0.45 0.79 0.3825 0.3145 0.3205 0.2335
Colon: 1 0.0385 0.3635 0.581 0.5465 0.4205 0.2325 0.1255 0.2085 0.2445 0.114 0.256 0.22
Heart: 1 0.0375 0.332 0.0615 0.03 0.0175 0.016 0.7785 0.017 0.2145 0.0385 0.014
Kidney: 1 0.709 0.2455 0.1945 0.2895 0.4755 0.379 0.349 0.094 0.297 0.181
Liver: 1 0.4755 0.548 0.6115 0.5955 0.6635 0.608 0.5535 0.6685 0.4425
Lung: 1 0.3855 0.2455 0.692 0.2995 0.3995 0.1965 0.3375 0.228
LymphNode: 1 0.2065 0.215 0.2745 0.193 0.091 0.38 0.1455
Ovary: 1 0.137 0.1945 0.2405 0.1415 0.1115 0.085
Prostate: 1 0.1145 0.122 0.089 0.3365 0.153
SkeletalMuscle: 1 0.0715 0.303 0.275 0.2755
Testes: 1 0.109 0.202 0.0885
Thyroid: 1 0.1915 0.1095
WhiteBloodCells: 1 0.2845
EdomStromalCell: 1
Placenta Adipose Adrenal Brain Breast Colon Heart Kidney Liver Lung LymphNode Ovary Prostate SkeletalMuscle Testes Thyroid WhiteBloodCells EdomStromalCell

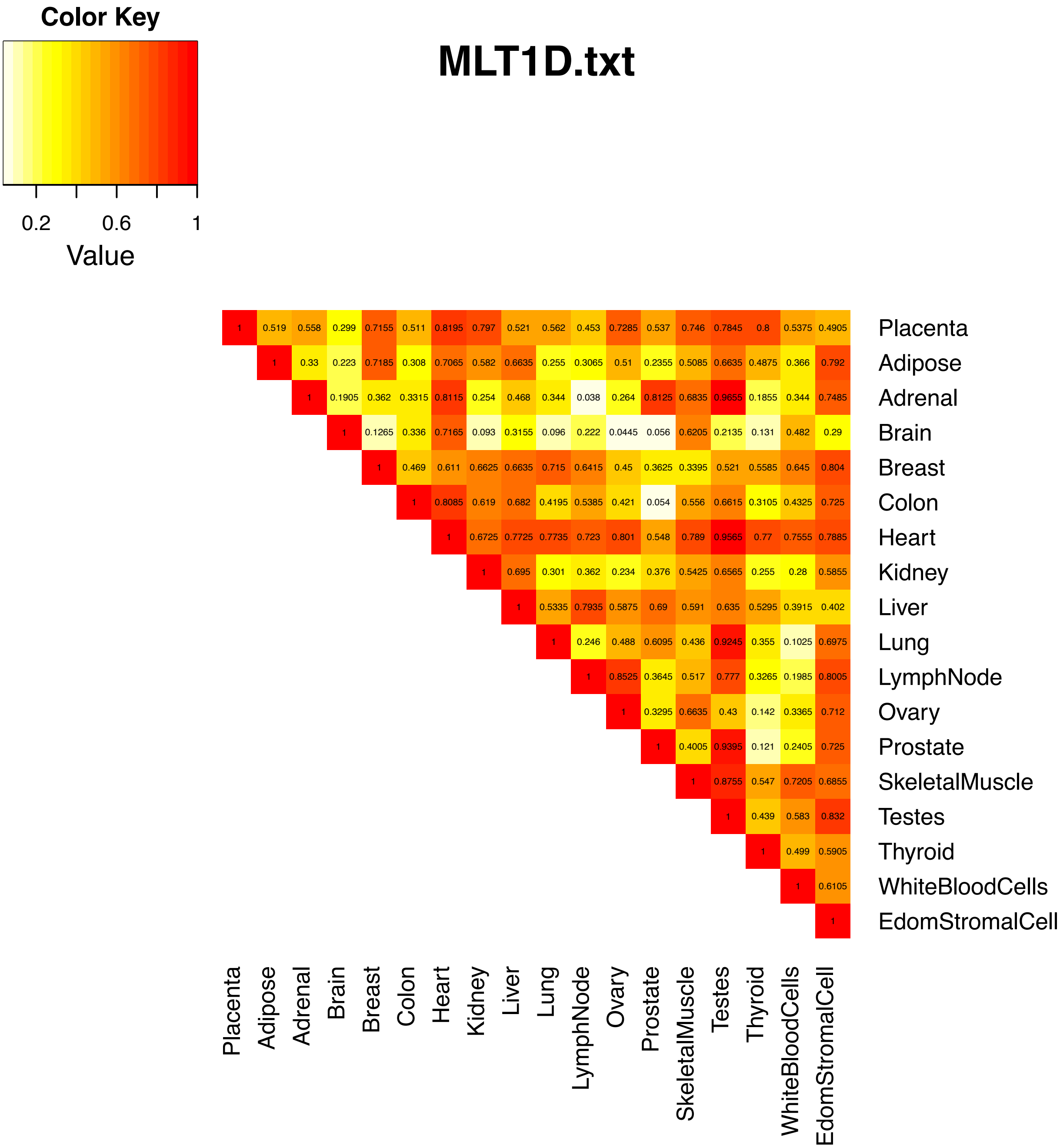
Color Key
0.2
0.6
1
Value
MLT1D.txt
Placenta 1 0.519 0.558 0.299 0.7155 0.511 0.8195 0.797 0.521 0.562 0.453 0.7285 0.537 0.746 0.7845 0.8 0.5375 0.4905
Adipose 1 0.33 0.223 0.7185 0.308 0.7065 0.582 0.6635 0.255 0.3065 0.51 0.2355 0.5085 0.6635 0.4875 0.366 0.792
Adrenal 1 0.1905 0.362 0.3315 0.8115 0.254 0.468 0.344 0.038 0.264 0.8125 0.6835 0.9655 0.1855 0.344 0.7485
Brain 1 0.1265 0.336 0.7165 0.093 0.3155 0.096 0.222 0.0445 0.056 0.6205 0.2135 0.131 0.482 0.29
Breast 1 0.469 0.611 0.6625 0.6635 0.715 0.6415 0.45 0.3625 0.3395 0.521 0.5585 0.645 0.804
Colon 1 0.8085 0.619 0.682 0.4195 0.5385 0.421 0.054 0.556 0.6615 0.3105 0.4325 0.725
Heart 1 0.6725 0.7725 0.7735 0.723 0.801 0.548 0.789 0.9565 0.77 0.7555 0.7885
Kidney 1 0.695 0.301 0.362 0.234 0.376 0.5425 0.6565 0.255 0.28 0.5855
Liver 1 0.5335 0.7935 0.5875 0.69 0.591 0.635 0.5295 0.3915 0.402
Lung 1 0.246 0.488 0.6095 0.436 0.9245 0.355 0.1025 0.6975
LymphNode 1 0.8525 0.3645 0.517 0.777 0.3265 0.1985 0.8005
Ovary 1 0.3295 0.6635 0.43 0.142 0.3365 0.712
Prostate 1 0.4005 0.9395 0.121 0.2405 0.725
SkeletalMuscle 1 0.8755 0.547 0.7205 0.6855
Testes 1 0.439 0.583 0.832
Thyroid 1 0.499 0.5905
WhiteBloodCells 1 0.6105
EdomStromalCell 1
Placenta Adipose Adrenal Brain Breast Colon Heart Kidney Liver Lung LymphNode Ovary Prostate SkeletalMuscle Testes Thyroid WhiteBloodCells EdomStromalCell

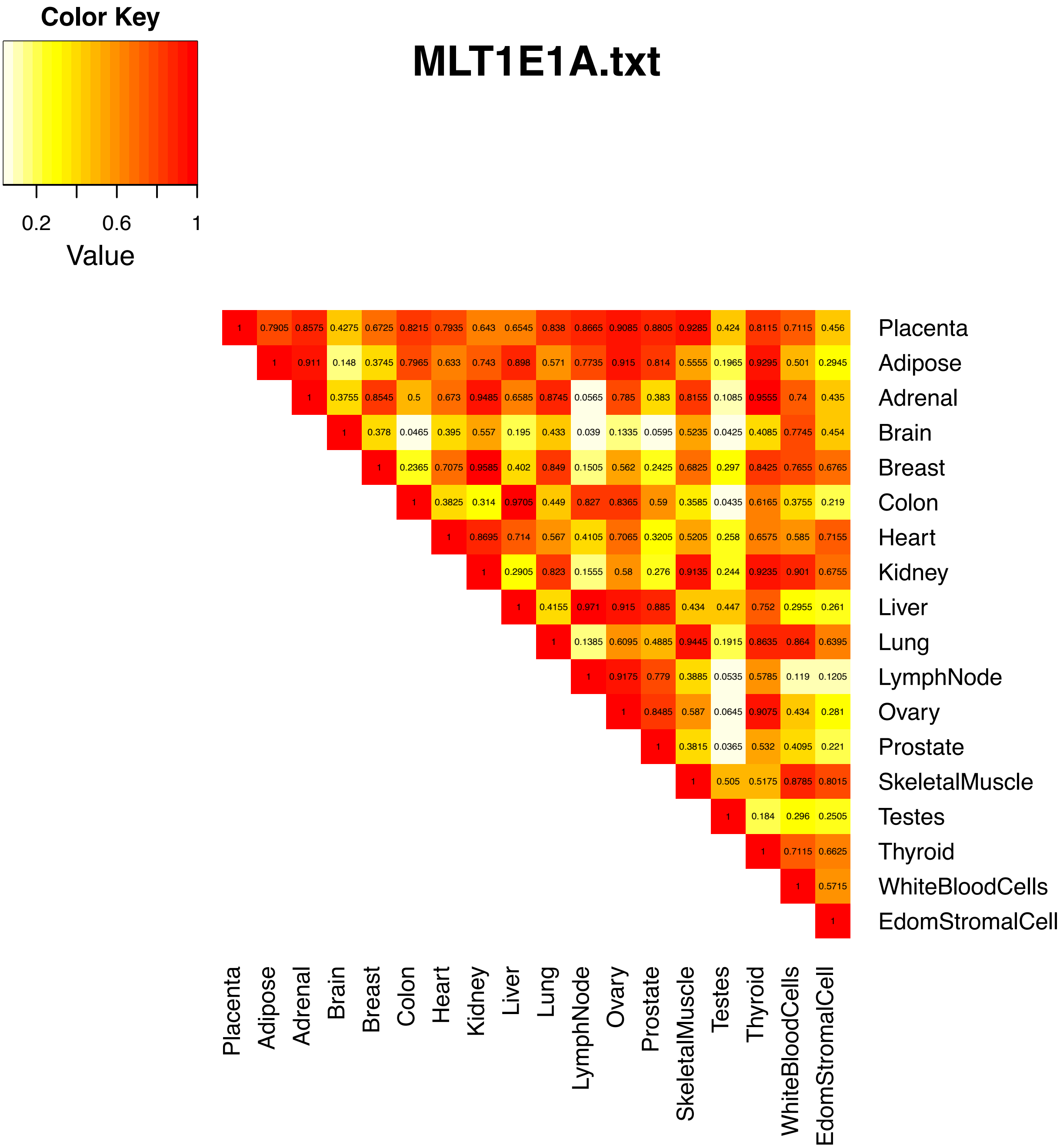
Color Key
0.2
0.6
1
Value
MLT1E1A.txt
Placenta 1 0.7905 0.8575 0.4275 0.6725 0.8215 0.7935 0.643 0.6545 0.838 0.8665 0.9085 0.8805 0.9285 0.424 0.8115 0.7115 0.456
Adipose 1 0.911 0.148 0.3745 0.7965 0.633 0.743 0.898 0.571 0.7735 0.915 0.814 0.5555 0.1965 0.9295 0.501 0.2945
Adrenal 1 0.3755 0.8545 0.5 0.673 0.9485 0.6585 0.8745 0.0565 0.785 0.383 0.8155 0.1085 0.9555 0.74 0.435
Brain 1 0.378 0.0465 0.395 0.557 0.195 0.433 0.039 0.1335 0.0595 0.5235 0.0425 0.4085 0.7745 0.454
Breast 1 0.2365 0.7075 0.9585 0.402 0.849 0.1505 0.562 0.2425 0.6825 0.297 0.8425 0.7655 0.6765
Colon 1 0.3825 0.314 0.9705 0.449 0.827 0.8365 0.59 0.3585 0.0435 0.6165 0.3755 0.219
Heart 1 0.8695 0.714 0.567 0.4105 0.7065 0.3205 0.5205 0.258 0.6575 0.585 0.7155
Kidney 1 0.2905 0.823 0.1555 0.58 0.276 0.9135 0.244 0.9235 0.901 0.6755
Liver 1 0.4155 0.971 0.915 0.885 0.434 0.447 0.752 0.2955 0.261
Lung 1 0.1385 0.6095 0.4885 0.9445 0.1915 0.8635 0.864 0.6395
LymphNode 1 0.9175 0.779 0.3885 0.0535 0.5785 0.119 0.1205
Ovary 1 0.8485 0.587 0.0645 0.9075 0.434 0.281
Prostate 1 0.3815 0.0365 0.532 0.4095 0.221
SkeletalMuscle 1 0.505 0.5175 0.8785 0.8015
Testes 1 0.184 0.296 0.2505
Thyroid 1 0.7115 0.6625
WhiteBloodCells 1 0.5715
EdomStromalCell 1
Placenta
Adipose
Adrenal
Brain
Breast
Colon
Heart
Kidney
Liver
Lung
LymphNode
Ovary
Prostate
SkeletalMuscle
Testes
Thyroid
WhiteBloodCells
EdomStromalCell

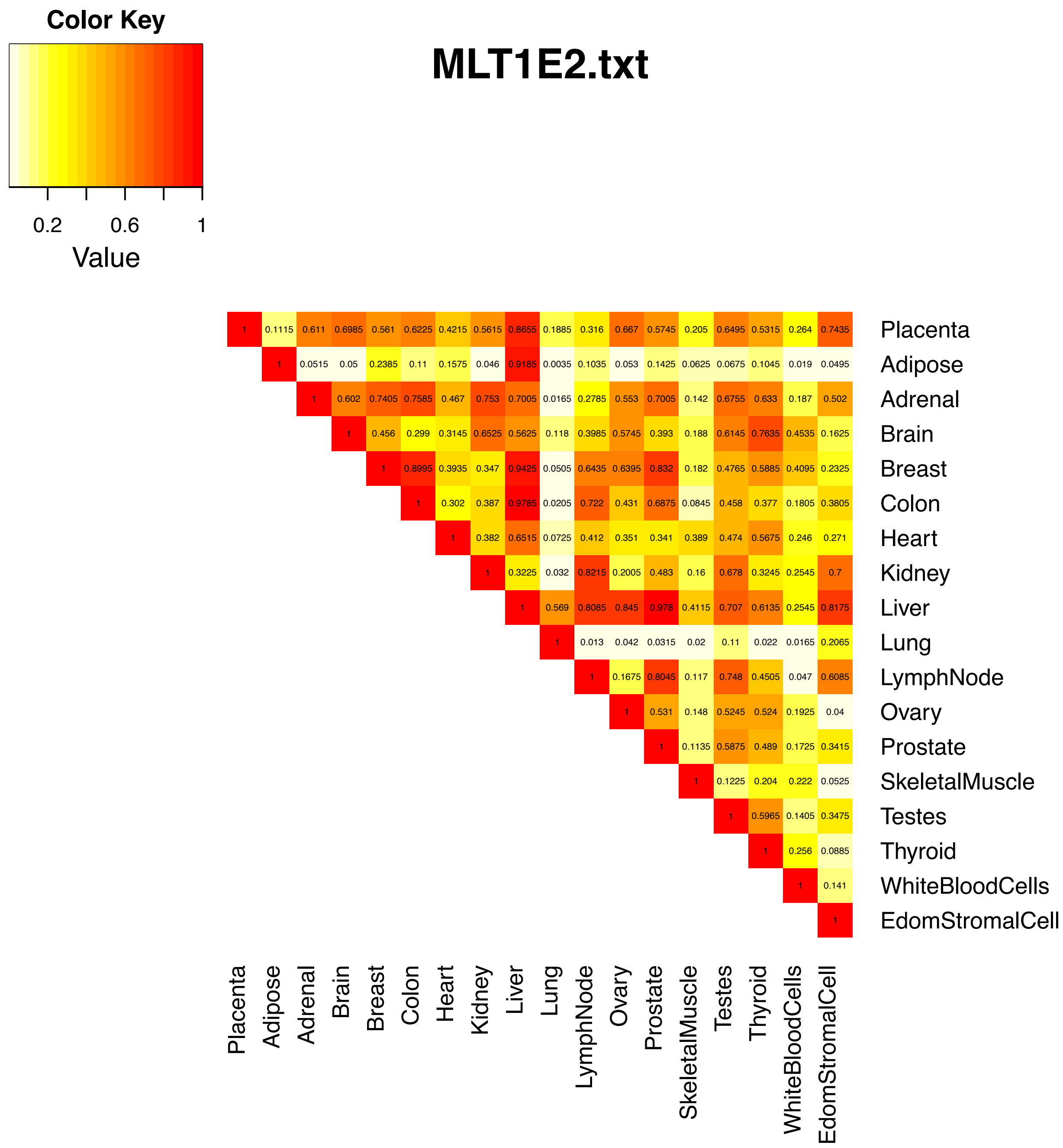

Color Key
0.2
0.6
1
Value
MLT1E2.txt
Placenta: 1 0.1115 0.611 0.6985 0.561 0.6225 0.4215 0.5615 0.8655 0.1885 0.316 0.667 0.5745 0.205 0.6495 0.5315 0.264 0.7435
Adipose: 1 0.0515 0.05 0.2385 0.11 0.1575 0.046 0.9185 0.0035 0.1035 0.053 0.1425 0.0625 0.0675 0.1045 0.019 0.0495
Adrenal: 1 0.602 0.7405 0.7585 0.467 0.753 0.7005 0.0165 0.2785 0.553 0.7005 0.142 0.6755 0.633 0.187 0.502
Brain: 1 0.456 0.299 0.3145 0.6525 0.5625 0.118 0.3985 0.5745 0.393 0.188 0.6145 0.7635 0.4535 0.1625
Breast: 1 0.8995 0.3935 0.347 0.9425 0.0505 0.6435 0.6395 0.832 0.182 0.4765 0.5885 0.4095 0.2325
Colon: 1 0.302 0.387 0.9785 0.0205 0.722 0.431 0.6875 0.0845 0.458 0.377 0.1805 0.3805
Heart: 1 0.382 0.6515 0.0725 0.412 0.351 0.341 0.389 0.474 0.5675 0.246 0.271
Kidney: 1 0.3225 0.032 0.8215 0.2005 0.483 0.16 0.678 0.3245 0.2545 0.7
Liver: 1 0.569 0.8085 0.845 0.978 0.4115 0.707 0.6135 0.2545 0.8175
Lung: 1 0.013 0.042 0.0315 0.02 0.11 0.022 0.0165 0.2065
LymphNode: 1 0.1675 0.8045 0.117 0.748 0.4505 0.047 0.6085
Ovary: 1 0.531 0.148 0.5245 0.524 0.1925 0.04
Prostate: 1 0.1135 0.5875 0.489 0.1725 0.3415
SkeletalMuscle: 1 0.1225 0.204 0.222 0.0525
Testes: 1 0.5965 0.1405 0.3475
Thyroid: 1 0.256 0.0885
WhiteBloodCells: 1 0.141
EdomStromalCell: 1
Placenta Adipose Adrenal Brain Breast Colon Heart Kidney Liver Lung LymphNode Ovary Prostate SkeletalMuscle Testes Thyroid WhiteBloodCells EdomStromalCell

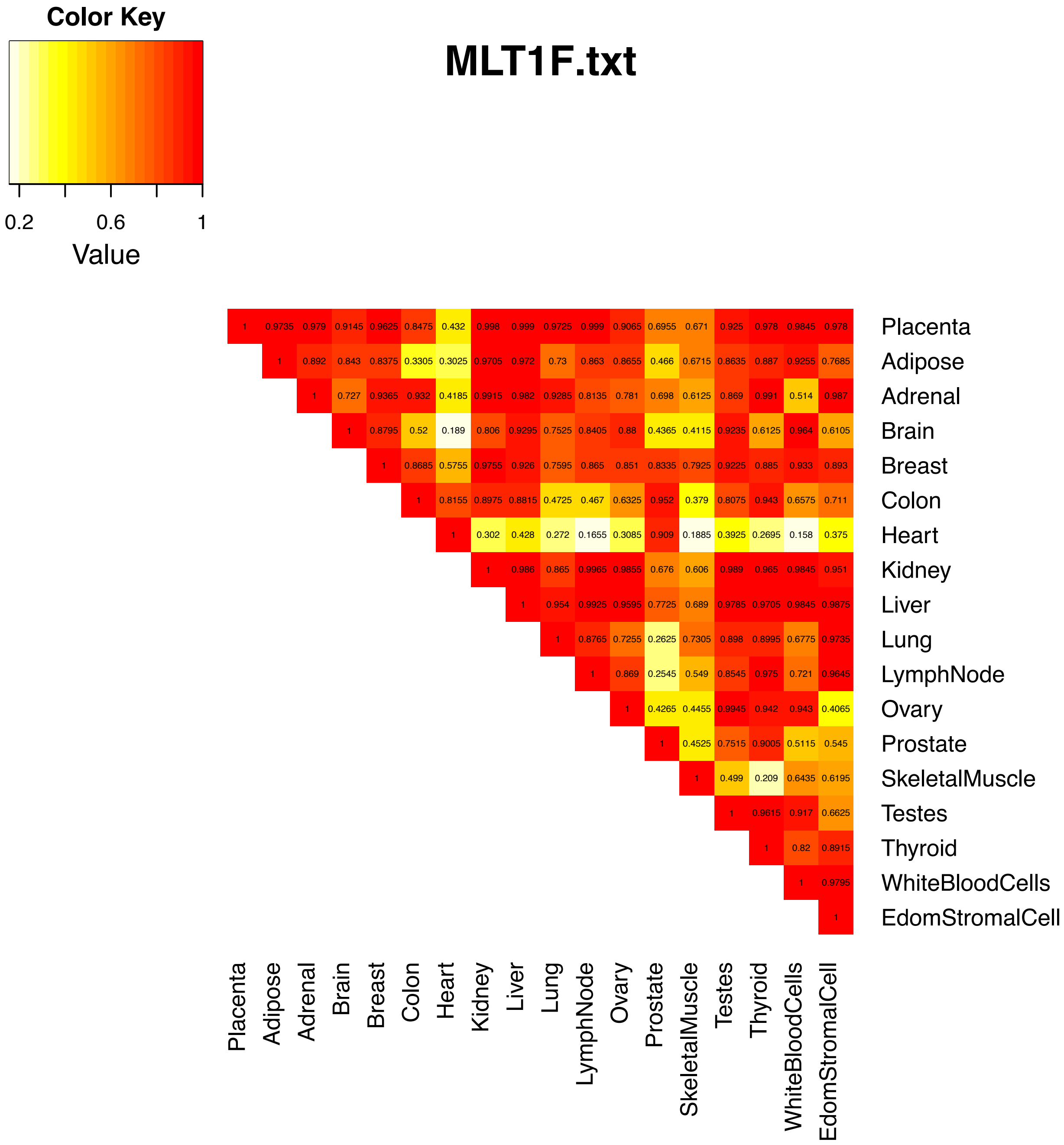

Color Key
0.2
0.6
1
Value
MLT1F.txt
Placenta: 1 0.9735 0.979 0.9145 0.9625 0.8475 0.432 0.998 0.999 0.9725 0.999 0.9065 0.6955 0.671 0.925 0.978 0.9845 0.978
Adipose: 1 0.892 0.843 0.8375 0.3305 0.3025 0.9705 0.972 0.73 0.863 0.8655 0.466 0.6715 0.8635 0.887 0.9255 0.7685
Adrenal: 1 0.727 0.9365 0.932 0.4185 0.9915 0.982 0.9285 0.8135 0.781 0.698 0.6125 0.869 0.991 0.514 0.987
Brain: 1 0.8795 0.52 0.189 0.806 0.9295 0.7525 0.8405 0.88 0.4365 0.4115 0.9235 0.6125 0.964 0.6105
Breast: 1 0.8685 0.5755 0.9755 0.926 0.7595 0.865 0.851 0.8335 0.7925 0.9225 0.885 0.933 0.893
Colon: 1 0.8155 0.8975 0.8815 0.4725 0.467 0.6325 0.952 0.379 0.8075 0.943 0.6575 0.711
Heart: 1 0.302 0.428 0.272 0.1655 0.3085 0.909 0.1885 0.3925 0.2695 0.158 0.375
Kidney: 1 0.986 0.865 0.9965 0.9855 0.676 0.606 0.989 0.965 0.9845 0.951
Liver: 1 0.954 0.9925 0.9595 0.7725 0.689 0.9785 0.9705 0.9845 0.9875
Lung: 1 0.8765 0.7255 0.2625 0.7305 0.898 0.8995 0.6775 0.9735
LymphNode: 1 0.869 0.2545 0.549 0.8545 0.975 0.721 0.9645
Ovary: 1 0.4265 0.4455 0.9945 0.942 0.943 0.4065
Prostate: 1 0.4525 0.7515 0.9005 0.5115 0.545
SkeletalMuscle: 1 0.499 0.209 0.6435 0.6195
Testes: 1 0.9615 0.917 0.6625
Thyroid: 1 0.82 0.8915
WhiteBloodCells: 1 0.9795
EdomStromalCell: 1
Placenta
Adipose
Adrenal
Brain
Breast
Colon
Heart
Kidney
Liver
Lung
LymphNode
Ovary
Prostate
SkeletalMuscle
Testes
Thyroid
WhiteBloodCells
EdomStromalCell

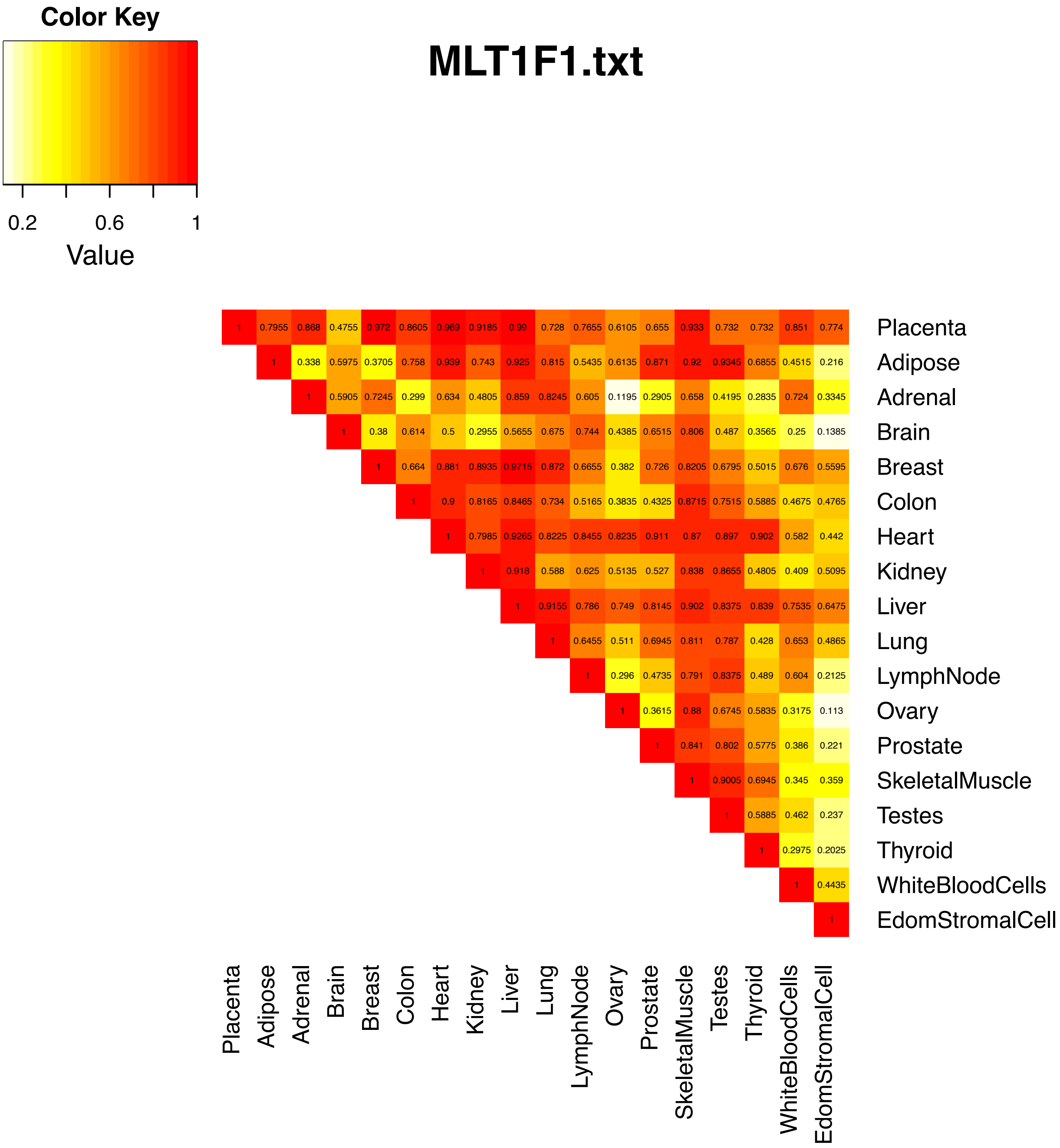
Color Key
0.2 0.6 1
Value
MLT1F1.txt
1 0.7955 0.868 0.4755 0.972 0.8605 0.969 0.9185 0.99 0.728 0.7655 0.6105 0.655 0.933 0.732 0.732 0.851 0.774 Placenta
1 0.338 0.5975 0.3705 0.758 0.939 0.743 0.925 0.815 0.5435 0.6135 0.871 0.92 0.9345 0.6855 0.4515 0.216 Adipose
1 0.5905 0.7245 0.299 0.634 0.4805 0.859 0.8245 0.605 0.1195 0.2905 0.658 0.4195 0.2835 0.724 0.3345 Adrenal
1 0.38 0.614 0.5 0.2955 0.5655 0.675 0.744 0.4385 0.6515 0.806 0.487 0.3565 0.25 0.1385 Brain
1 0.664 0.881 0.8935 0.9715 0.872 0.6655 0.382 0.726 0.8205 0.6795 0.5015 0.676 0.5595 Breast
1 0.9 0.8165 0.8465 0.734 0.5165 0.3835 0.4325 0.8715 0.7515 0.5885 0.4675 0.4765 Colon
1 0.7985 0.9265 0.8225 0.8455 0.8235 0.911 0.87 0.897 0.902 0.582 0.442 Heart
1 0.918 0.588 0.625 0.5135 0.527 0.838 0.8655 0.4805 0.409 0.5095 Kidney
1 0.9155 0.786 0.749 0.8145 0.902 0.8375 0.839 0.7535 0.6475 Liver
1 0.6455 0.511 0.6945 0.811 0.787 0.428 0.653 0.4865 Lung
1 0.296 0.4735 0.791 0.8375 0.489 0.604 0.2125 LymphNode
1 0.3615 0.88 0.6745 0.5835 0.3175 0.113 Ovary
1 0.841 0.802 0.5775 0.386 0.221 Prostate
1 0.9005 0.6945 0.345 0.359 SkeletalMuscle
1 0.5885 0.462 0.237 Testes
1 0.2975 0.2025 Thyroid
1 0.4435 WhiteBloodCells
1 EdomStromalCell
Placenta Adipose Adrenal Brain Breast Colon Heart Kidney Liver Lung LymphNode Ovary Prostate SkeletalMuscle Testes Thyroid WhiteBloodCells EdomStromalCell

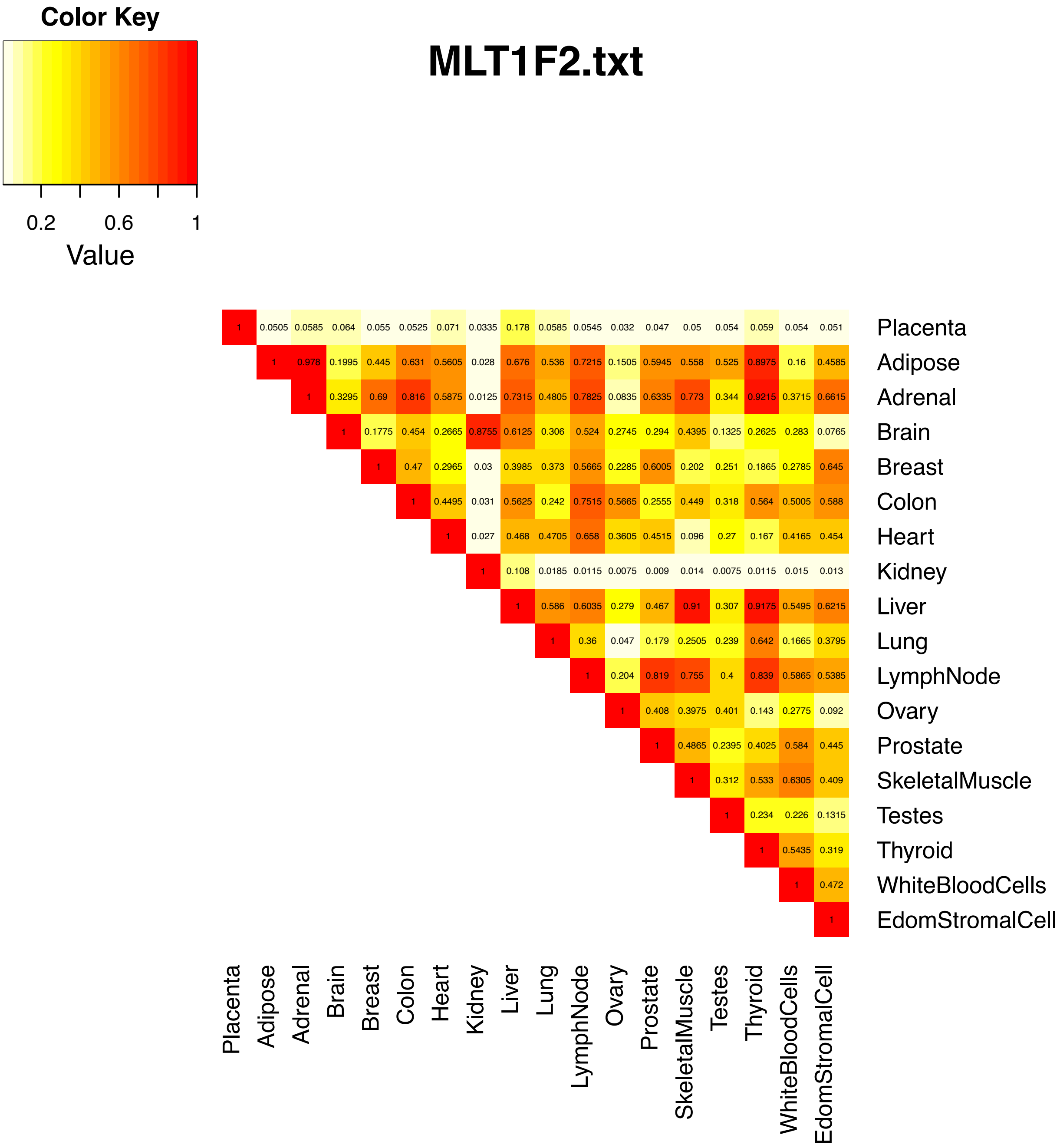

Color Key
0.2
0.6
1
Value
MLT1F2.txt
Placenta: 1 0.0505 0.0585 0.064 0.055 0.0525 0.071 0.0335 0.178 0.0585 0.0545 0.032 0.047 0.05 0.054 0.059 0.054 0.051
Adipose: 1 0.978 0.1995 0.445 0.631 0.5605 0.028 0.676 0.536 0.7215 0.1505 0.5945 0.558 0.525 0.8975 0.16 0.4585
Adrenal: 1 0.3295 0.69 0.816 0.5875 0.0125 0.7315 0.4805 0.7825 0.0835 0.6335 0.773 0.344 0.9215 0.3715 0.6615
Brain: 1 0.1775 0.454 0.2665 0.8755 0.6125 0.306 0.524 0.2745 0.294 0.4395 0.1325 0.2625 0.283 0.0765
Breast: 1 0.47 0.2965 0.03 0.3985 0.373 0.5665 0.2285 0.6005 0.202 0.251 0.1865 0.2785 0.645
Colon: 1 0.4495 0.031 0.5625 0.242 0.7515 0.5665 0.2555 0.449 0.318 0.564 0.5005 0.588
Heart: 1 0.027 0.468 0.4705 0.658 0.3605 0.4515 0.096 0.27 0.167 0.4165 0.454
Kidney: 1 0.108 0.0185 0.0115 0.0075 0.009 0.014 0.0075 0.0115 0.015 0.013
Liver: 1 0.586 0.6035 0.279 0.467 0.91 0.307 0.9175 0.5495 0.6215
Lung: 1 0.36 0.047 0.179 0.2505 0.239 0.642 0.1665 0.3795
LymphNode: 1 0.204 0.819 0.755 0.4 0.839 0.5865 0.5385
Ovary: 1 0.408 0.3975 0.401 0.143 0.2775 0.092
Prostate: 1 0.4865 0.2395 0.4025 0.584 0.445
SkeletalMuscle: 1 0.312 0.533 0.6305 0.409
Testes: 1 0.234 0.226 0.1315
Thyroid: 1 0.5435 0.319
WhiteBloodCells: 1 0.472
EdomStromalCell: 1
Placenta Adipose Adrenal Brain Breast Colon Heart Kidney Liver Lung LymphNode Ovary Prostate SkeletalMuscle Testes Thyroid WhiteBloodCells EdomStromalCell

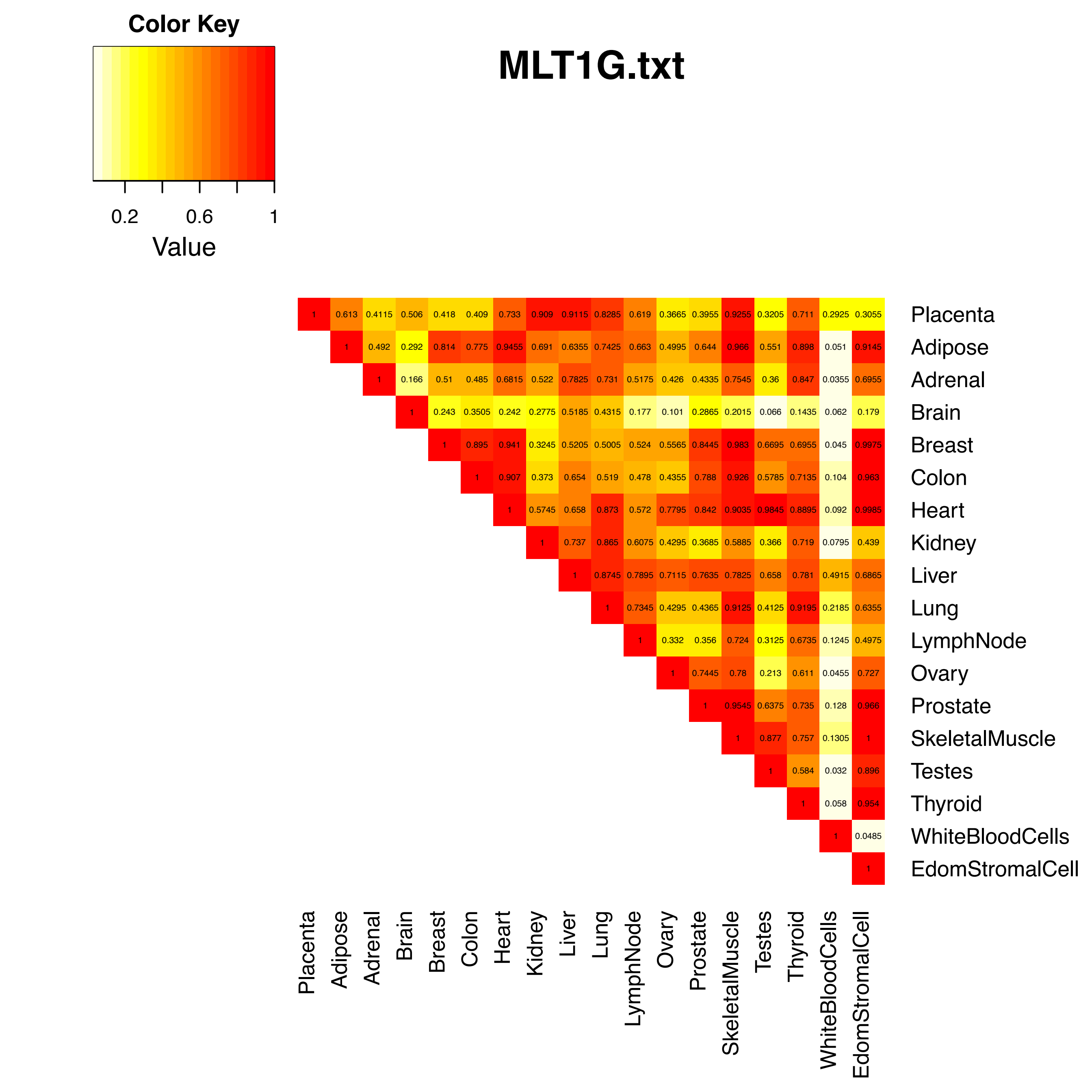
Color Key
0.2
0.6
1
Value
MLT1G.txt
Placenta 1 0.613 0.4115 0.506 0.418 0.409 0.733 0.909 0.9115 0.8285 0.619 0.3665 0.3955 0.9255 0.3205 0.711 0.2925 0.3055
Adipose 1 0.492 0.292 0.814 0.775 0.9455 0.691 0.6355 0.7425 0.663 0.4995 0.644 0.966 0.551 0.898 0.051 0.9145
Adrenal 1 0.166 0.51 0.485 0.6815 0.522 0.7825 0.731 0.5175 0.426 0.4335 0.7545 0.36 0.847 0.0355 0.6955
Brain 1 0.243 0.3505 0.242 0.2775 0.5185 0.4315 0.177 0.101 0.2865 0.2015 0.066 0.1435 0.062 0.179
Breast 1 0.895 0.941 0.3245 0.5205 0.5005 0.524 0.5565 0.8445 0.983 0.6695 0.6955 0.045 0.9975
Colon 1 0.907 0.373 0.654 0.519 0.478 0.4355 0.788 0.926 0.5785 0.7135 0.104 0.963
Heart 1 0.5745 0.658 0.873 0.572 0.7795 0.842 0.9035 0.9845 0.8895 0.092 0.9985
Kidney 1 0.737 0.865 0.6075 0.4295 0.3685 0.5885 0.366 0.719 0.0795 0.439
Liver 1 0.8745 0.7895 0.7115 0.7635 0.7825 0.658 0.781 0.4915 0.6865
Lung 1 0.7345 0.4295 0.4365 0.9125 0.4125 0.9195 0.2185 0.6355
LymphNode 1 0.332 0.356 0.724 0.3125 0.6735 0.1245 0.4975
Ovary 1 0.7445 0.78 0.213 0.611 0.0455 0.727
Prostate 1 0.9545 0.6375 0.735 0.128 0.966
SkeletalMuscle 1 0.877 0.757 0.1305 1
Testes 1 0.584 0.032 0.896
Thyroid 1 0.058 0.954
WhiteBloodCells 1 0.0485
EdomStromalCell 1
Placenta
Adipose
Adrenal
Brain
Breast
Colon
Heart
Kidney
Liver
Lung
LymphNode
Ovary
Prostate
SkeletalMuscle
Testes
Thyroid
WhiteBloodCells
EdomStromalCell

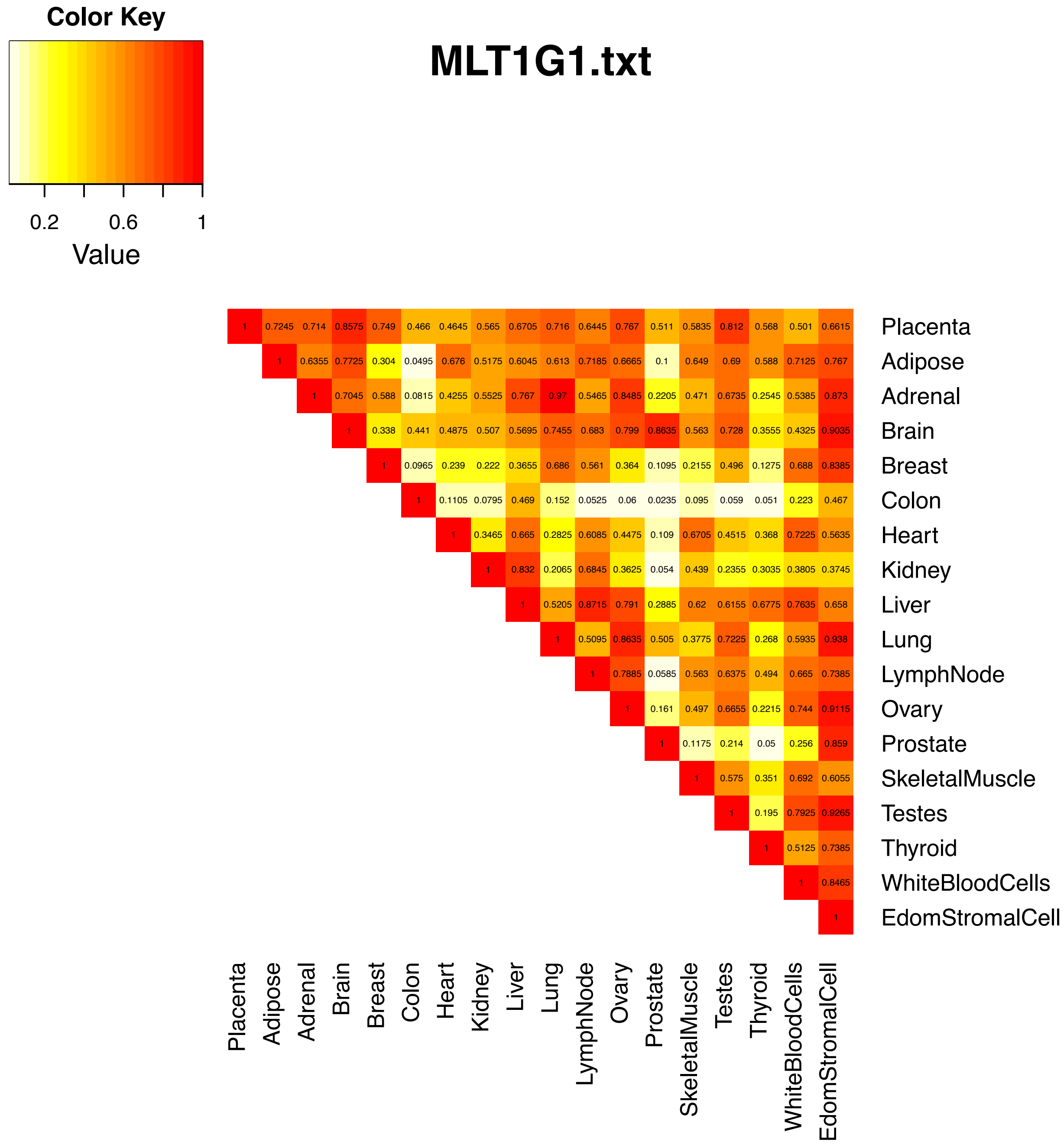

Color Key
0.2
0.6
1
Value
MLT1G1.txt
Placenta 1 0.7245 0.714 0.8575 0.749 0.466 0.4645 0.565 0.6705 0.716 0.6445 0.767 0.511 0.5835 0.812 0.568 0.501 0.6615
Adipose 1 0.6355 0.7725 0.304 0.0495 0.676 0.5175 0.6045 0.613 0.7185 0.6665 0.1 0.649 0.69 0.588 0.7125 0.767
Adrenal 1 0.7045 0.588 0.0815 0.4255 0.5525 0.767 0.97 0.5465 0.8485 0.2205 0.471 0.6735 0.2545 0.5385 0.873
Brain 1 0.338 0.441 0.4875 0.507 0.5695 0.7455 0.683 0.799 0.8635 0.563 0.728 0.3555 0.4325 0.9035
Breast 1 0.0965 0.239 0.222 0.3655 0.686 0.561 0.364 0.1095 0.2155 0.496 0.1275 0.688 0.8385
Colon 1 0.1105 0.0795 0.469 0.152 0.0525 0.06 0.0235 0.095 0.059 0.051 0.223 0.467
Heart 1 0.3465 0.665 0.2825 0.6085 0.4475 0.109 0.6705 0.4515 0.368 0.7225 0.5635
Kidney 1 0.832 0.2065 0.6845 0.3625 0.054 0.439 0.2355 0.3035 0.3805 0.3745
Liver 1 0.5205 0.8715 0.791 0.2885 0.62 0.6155 0.6775 0.7635 0.658
Lung 1 0.5095 0.8635 0.505 0.3775 0.7225 0.268 0.5935 0.938
LymphNode 1 0.7885 0.0585 0.563 0.6375 0.494 0.665 0.7385
Ovary 1 0.161 0.497 0.6655 0.2215 0.744 0.9115
Prostate 1 0.1175 0.214 0.05 0.256 0.859
SkeletalMuscle 1 0.575 0.351 0.692 0.6055
Testes 1 0.195 0.7925 0.9265
Thyroid 1 0.5125 0.7385
WhiteBloodCells 1 0.8465
EdomStromalCell 1
Placenta
Adipose
Adrenal
Brain
Breast
Colon
Heart
Kidney
Liver
Lung
LymphNode
Ovary
Prostate
SkeletalMuscle
Testes
Thyroid
WhiteBloodCells
EdomStromalCell

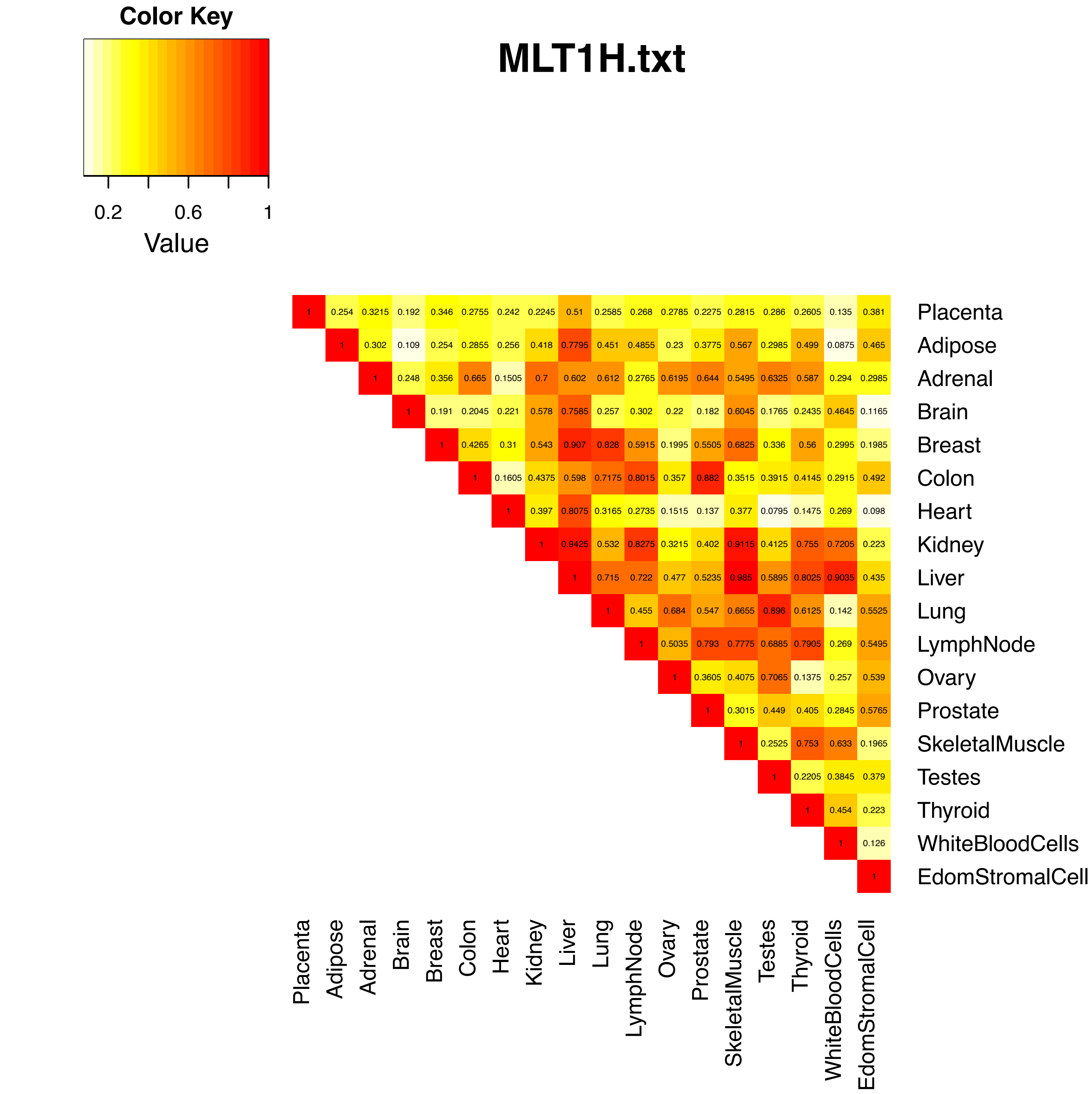
Color Key
0.2
0.6
1
Value
MLT1H.txt
Placenta
1 0.254 0.3215 0.192 0.346 0.2755 0.242 0.2245 0.51 0.2585 0.268 0.2785 0.2275 0.2815 0.286 0.2605 0.135 0.381
Adipose
1 0.302 0.109 0.254 0.2855 0.256 0.418 0.7795 0.451 0.4855 0.23 0.3775 0.567 0.2985 0.499 0.0875 0.465
Adrenal
1 0.248 0.356 0.665 0.1505 0.7 0.602 0.612 0.2765 0.6195 0.644 0.5495 0.6325 0.587 0.294 0.2985
Brain
1 0.191 0.2045 0.221 0.578 0.7585 0.257 0.302 0.22 0.182 0.6045 0.1765 0.2435 0.4645 0.1165
Breast
1 0.4265 0.31 0.543 0.907 0.828 0.5915 0.1995 0.5505 0.6825 0.336 0.56 0.2995 0.1985
Colon
1 0.1605 0.4375 0.598 0.7175 0.8015 0.357 0.882 0.3515 0.3915 0.4145 0.2915 0.492
Heart
1 0.397 0.8075 0.3165 0.2735 0.1515 0.137 0.377 0.0795 0.1475 0.269 0.098
Kidney
1 0.9425 0.532 0.8275 0.3215 0.402 0.9115 0.4125 0.755 0.7205 0.223
Liver
1 0.715 0.722 0.477 0.5235 0.985 0.5895 0.8025 0.9035 0.435
Lung
1 0.455 0.684 0.547 0.6655 0.896 0.6125 0.142 0.5525
LymphNode
1 0.5035 0.793 0.7775 0.6885 0.7905 0.269 0.5495
Ovary
1 0.3605 0.4075 0.7065 0.1375 0.257 0.539
Prostate
1 0.3015 0.449 0.405 0.2845 0.5765
SkeletalMuscle
1 0.2525 0.753 0.633 0.1965
Testes
1 0.2205 0.3845 0.379
Thyroid
1 0.454 0.223
WhiteBloodCells
1 0.126
EdomStromalCell
1
Placenta
Adipose
Adrenal
Brain
Breast
Colon
Heart
Kidney
Liver
Lung
LymphNode
Ovary
Prostate
SkeletalMuscle
Testes
Thyroid
WhiteBloodCells
EdomStromalCell

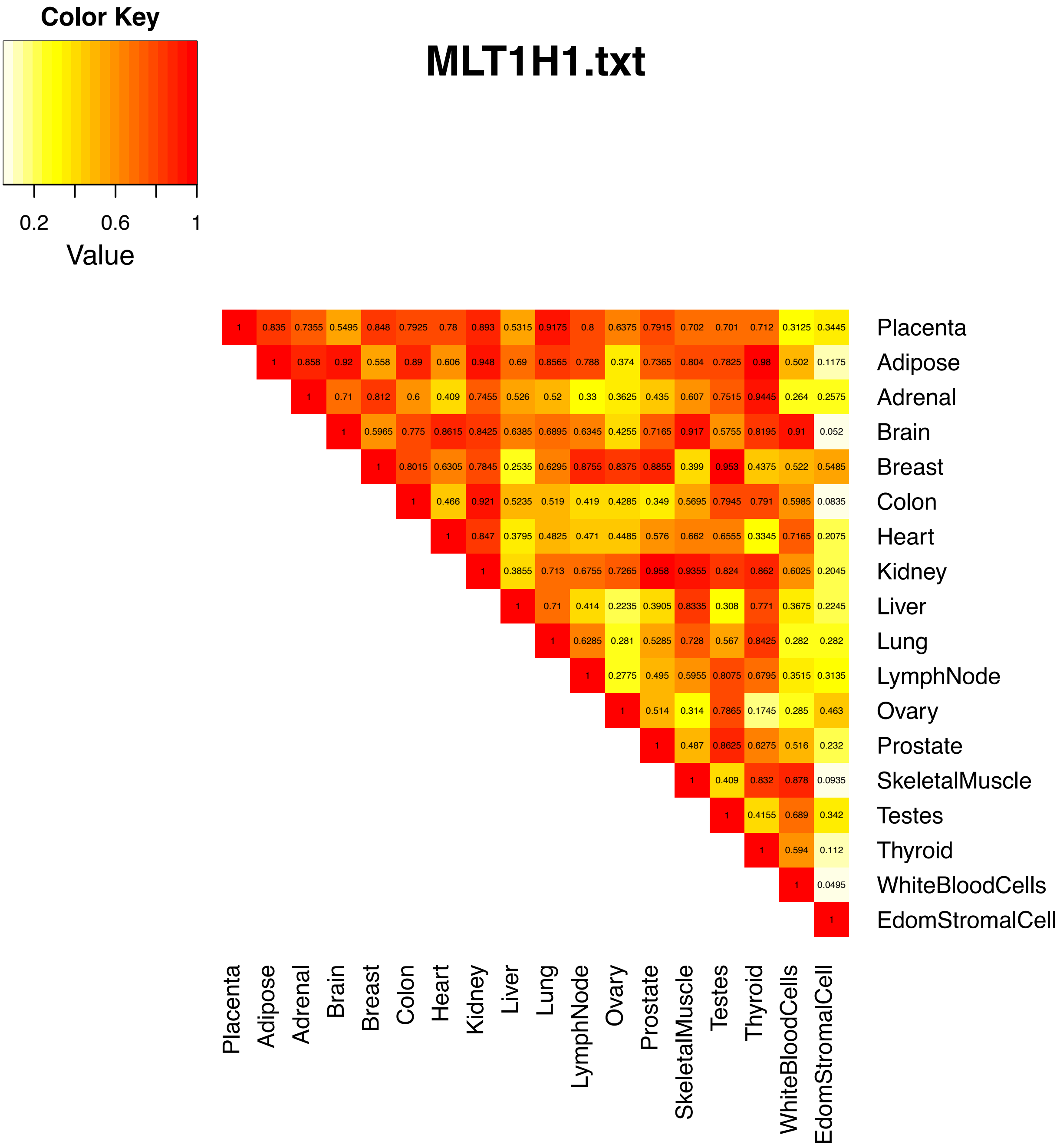
MLT1H1.txt
Color Key
0.2
0.6
1
Value
Placenta 1 0.835 0.7355 0.5495 0.848 0.7925 0.78 0.893 0.5315 0.9175 0.8 0.6375 0.7915 0.702 0.701 0.712 0.3125 0.3445
Adipose 1 0.858 0.92 0.558 0.89 0.606 0.948 0.69 0.8565 0.788 0.374 0.7365 0.804 0.7825 0.98 0.502 0.1175
Adrenal 1 0.71 0.812 0.6 0.409 0.7455 0.526 0.52 0.33 0.3625 0.435 0.607 0.7515 0.9445 0.264 0.2575
Brain 1 0.5965 0.775 0.8615 0.8425 0.6385 0.6895 0.6345 0.4255 0.7165 0.917 0.5755 0.8195 0.91 0.052
Breast 1 0.8015 0.6305 0.7845 0.2535 0.6295 0.8755 0.8375 0.8855 0.399 0.953 0.4375 0.522 0.5485
Colon 1 0.466 0.921 0.5235 0.519 0.419 0.4285 0.349 0.5695 0.7945 0.791 0.5985 0.0835
Heart 1 0.847 0.3795 0.4825 0.471 0.4485 0.576 0.662 0.6555 0.3345 0.7165 0.2075
Kidney 1 0.3855 0.713 0.6755 0.7265 0.958 0.9355 0.824 0.862 0.6025 0.2045
Liver 1 0.71 0.414 0.2235 0.3905 0.8335 0.308 0.771 0.3675 0.2245
Lung 1 0.6285 0.281 0.5285 0.728 0.567 0.8425 0.282 0.282
LymphNode 1 0.2775 0.495 0.5955 0.8075 0.6795 0.3515 0.3135
Ovary 1 0.514 0.314 0.7865 0.1745 0.285 0.463
Prostate 1 0.487 0.8625 0.6275 0.516 0.232
SkeletalMuscle 1 0.409 0.832 0.878 0.0935
Testes 1 0.4155 0.689 0.342
Thyroid 1 0.594 0.112
WhiteBloodCells 1 0.0495
EdomStromalCell 1
Placenta
Adipose
Adrenal
Brain
Breast
Colon
Heart
Kidney
Liver
Lung
LymphNode
Ovary
Prostate
SkeletalMuscle
Testes
Thyroid
WhiteBloodCells
EdomStromalCell

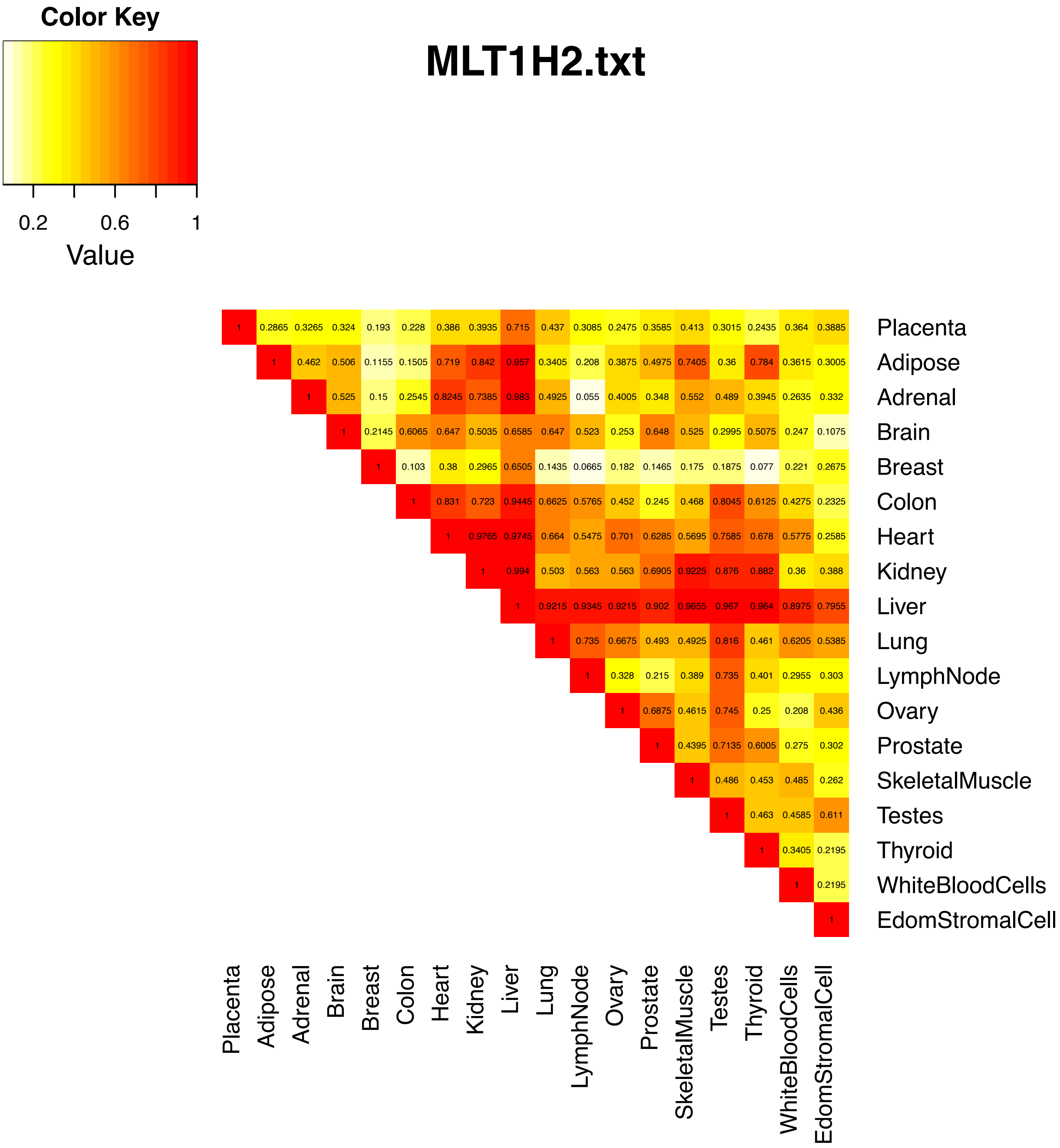
Color Key
0.2
0.6
1
Value
MLT1H2.txt
Placenta: 1 0.2865 0.3265 0.324 0.193 0.228 0.386 0.3935 0.715 0.437 0.3085 0.2475 0.3585 0.413 0.3015 0.2435 0.364 0.3885
Adipose: 1 0.462 0.506 0.1155 0.1505 0.719 0.842 0.957 0.3405 0.208 0.3875 0.4975 0.7405 0.36 0.784 0.3615 0.3005
Adrenal: 1 0.525 0.15 0.2545 0.8245 0.7385 0.983 0.4925 0.055 0.4005 0.348 0.552 0.489 0.3945 0.2635 0.332
Brain: 1 0.2145 0.6065 0.647 0.5035 0.6585 0.647 0.523 0.253 0.648 0.525 0.2995 0.5075 0.247 0.1075
Breast: 1 0.103 0.38 0.2965 0.6505 0.1435 0.0665 0.182 0.1465 0.175 0.1875 0.077 0.221 0.2675
Colon: 1 0.831 0.723 0.9445 0.6625 0.5765 0.452 0.245 0.468 0.8045 0.6125 0.4275 0.2325
Heart: 1 0.9765 0.9745 0.664 0.5475 0.701 0.6285 0.5695 0.7585 0.678 0.5775 0.2585
Kidney: 1 0.994 0.503 0.563 0.563 0.6905 0.9225 0.876 0.882 0.36 0.388
Liver: 1 0.9215 0.9345 0.9215 0.902 0.9655 0.967 0.964 0.8975 0.7955
Lung: 1 0.735 0.6675 0.493 0.4925 0.816 0.461 0.6205 0.5385
LymphNode: 1 0.328 0.215 0.389 0.735 0.401 0.2955 0.303
Ovary: 1 0.6875 0.4615 0.745 0.25 0.208 0.436
Prostate: 1 0.4395 0.7135 0.6005 0.275 0.302
SkeletalMuscle: 1 0.486 0.453 0.485 0.262
Testes: 1 0.463 0.4585 0.611
Thyroid: 1 0.3405 0.2195
WhiteBloodCells: 1 0.2195
EdomStromalCell: 1
Placenta
Adipose
Adrenal
Brain
Breast
Colon
Heart
Kidney
Liver
Lung
LymphNode
Ovary
Prostate
SkeletalMuscle
Testes
Thyroid
WhiteBloodCells
EdomStromalCell

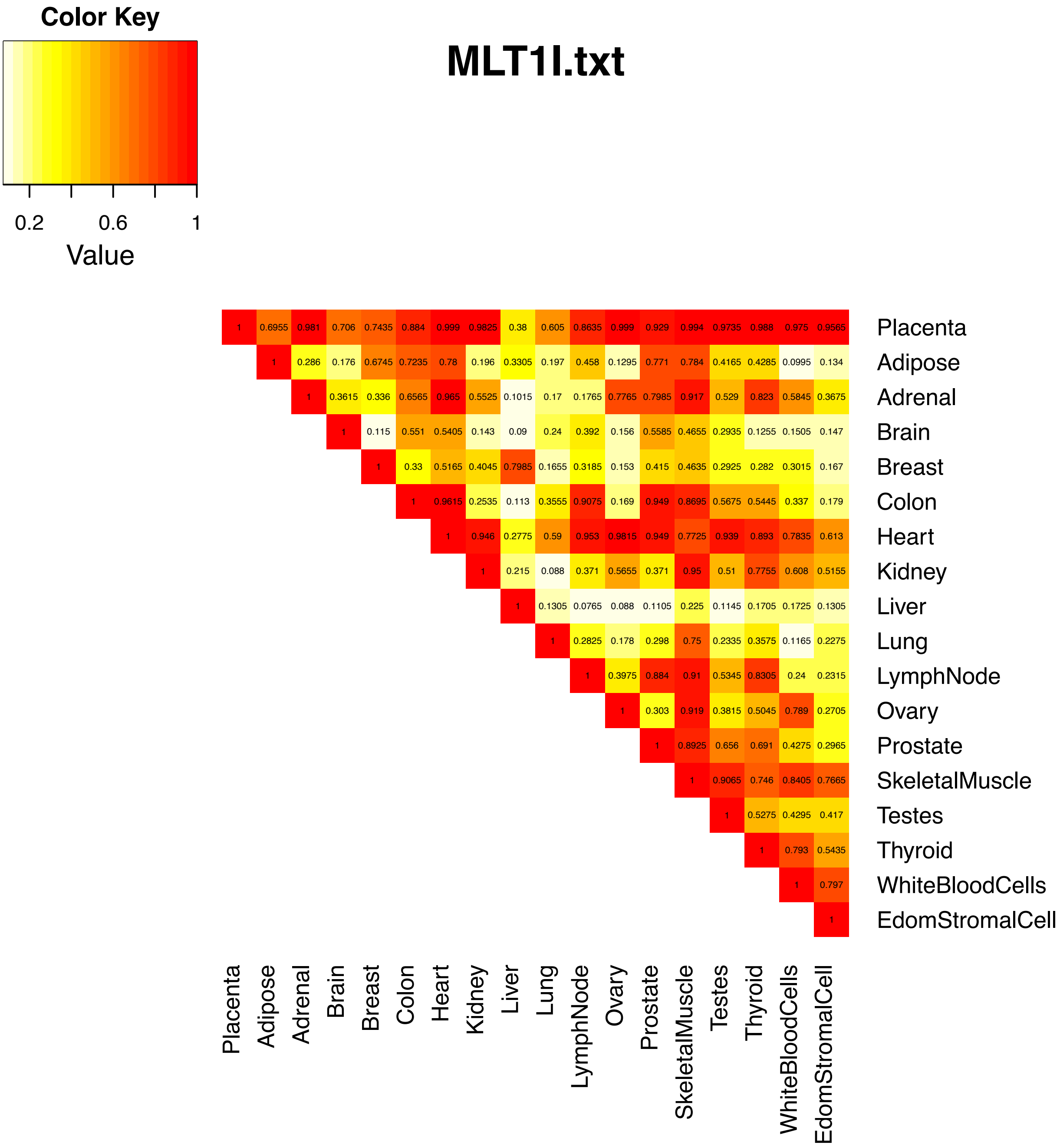
Color Key
0.2
0.6
1
Value
MLT1I.txt
Placenta: 1 0.6955 0.981 0.706 0.7435 0.884 0.999 0.9825 0.38 0.605 0.8635 0.999 0.929 0.994 0.9735 0.988 0.975 0.9565
Adipose: 1 0.286 0.176 0.6745 0.7235 0.78 0.196 0.3305 0.197 0.458 0.1295 0.771 0.784 0.4165 0.4285 0.0995 0.134
Adrenal: 1 0.3615 0.336 0.6565 0.965 0.5525 0.1015 0.17 0.1765 0.7765 0.7985 0.917 0.529 0.823 0.5845 0.3675
Brain: 1 0.115 0.551 0.5405 0.143 0.09 0.24 0.392 0.156 0.5585 0.4655 0.2935 0.1255 0.1505 0.147
Breast: 1 0.33 0.5165 0.4045 0.7985 0.1655 0.3185 0.153 0.415 0.4635 0.2925 0.282 0.3015 0.167
Colon: 1 0.9615 0.2535 0.113 0.3555 0.9075 0.169 0.949 0.8695 0.5675 0.5445 0.337 0.179
Heart: 1 0.946 0.2775 0.59 0.953 0.9815 0.949 0.7725 0.939 0.893 0.7835 0.613
Kidney: 1 0.215 0.088 0.371 0.5655 0.371 0.95 0.51 0.7755 0.608 0.5155
Liver: 1 0.1305 0.0765 0.088 0.1105 0.225 0.1145 0.1705 0.1725 0.1305
Lung: 1 0.2825 0.178 0.298 0.75 0.2335 0.3575 0.1165 0.2275
LymphNode: 1 0.3975 0.884 0.91 0.5345 0.8305 0.24 0.2315
Ovary: 1 0.303 0.919 0.3815 0.5045 0.789 0.2705
Prostate: 1 0.8925 0.656 0.691 0.4275 0.2965
SkeletalMuscle: 1 0.9065 0.746 0.8405 0.7665
Testes: 1 0.5275 0.4295 0.417
Thyroid: 1 0.793 0.5435
WhiteBloodCells: 1 0.797
EdomStromalCell: 1
Placenta Adipose Adrenal Brain Breast Colon Heart Kidney Liver Lung LymphNode Ovary Prostate SkeletalMuscle Testes Thyroid WhiteBloodCells EdomStromalCell

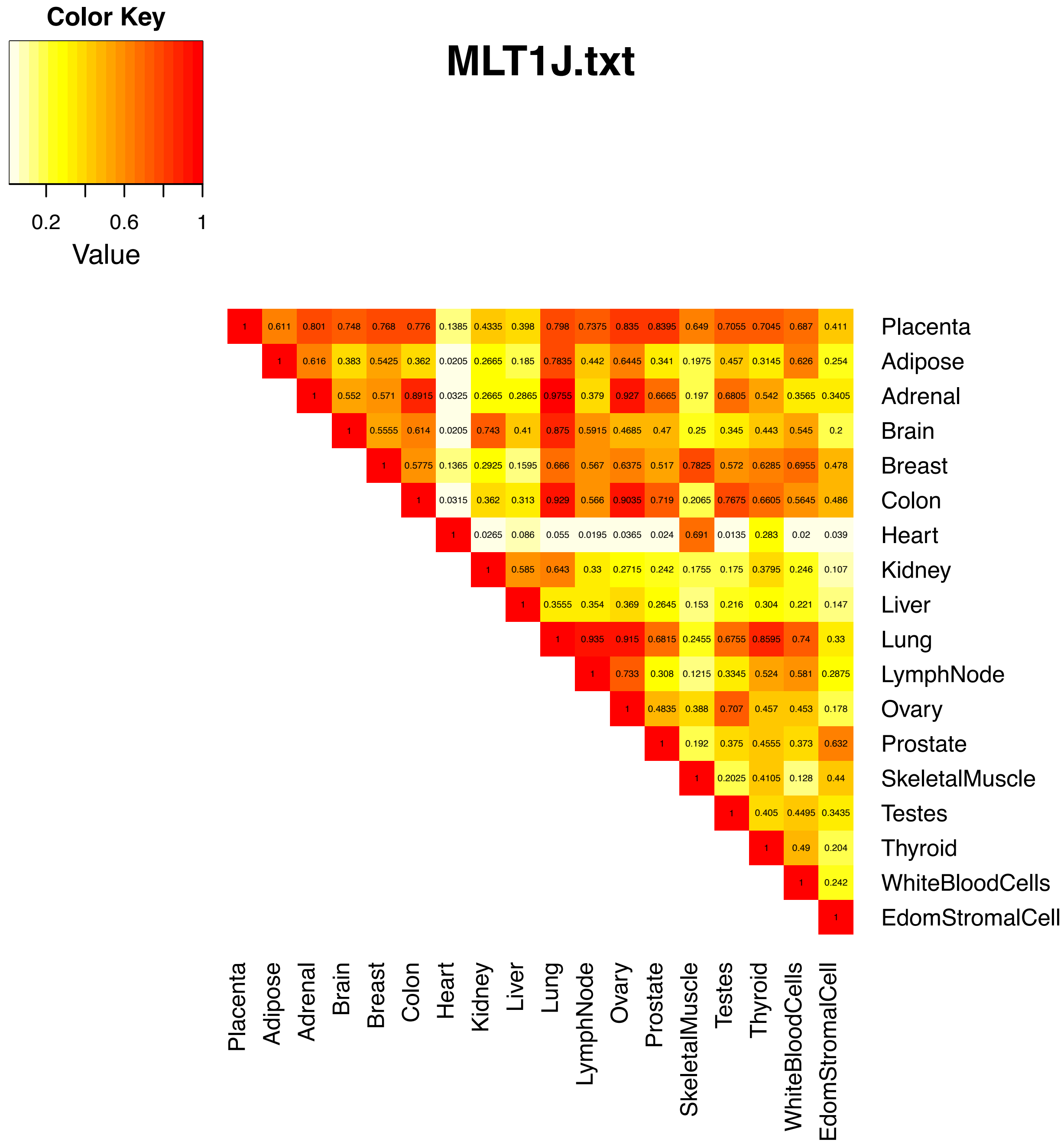
Color Key
0.2 0.6 1
Value
MLT1J.txt
Placenta 1 0.611 0.801 0.748 0.768 0.776 0.1385 0.4335 0.398 0.798 0.7375 0.835 0.8395 0.649 0.7055 0.7045 0.687 0.411
Adipose 1 0.616 0.383 0.5425 0.362 0.0205 0.2665 0.185 0.7835 0.442 0.6445 0.341 0.1975 0.457 0.3145 0.626 0.254
Adrenal 1 0.552 0.571 0.8915 0.0325 0.2665 0.2865 0.9755 0.379 0.927 0.6665 0.197 0.6805 0.542 0.3565 0.3405
Brain 1 0.5555 0.614 0.0205 0.743 0.41 0.875 0.5915 0.4685 0.47 0.25 0.345 0.443 0.545 0.2
Breast 1 0.5775 0.1365 0.2925 0.1595 0.666 0.567 0.6375 0.517 0.7825 0.572 0.6285 0.6955 0.478
Colon 1 0.0315 0.362 0.313 0.929 0.566 0.9035 0.719 0.2065 0.7675 0.6605 0.5645 0.486
Heart 1 0.0265 0.086 0.055 0.0195 0.0365 0.024 0.691 0.0135 0.283 0.02 0.039
Kidney 1 0.585 0.643 0.33 0.2715 0.242 0.1755 0.175 0.3795 0.246 0.107
Liver 1 0.3555 0.354 0.369 0.2645 0.153 0.216 0.304 0.221 0.147
Lung 1 0.935 0.915 0.6815 0.2455 0.6755 0.8595 0.74 0.33
LymphNode 1 0.733 0.308 0.1215 0.3345 0.524 0.581 0.2875
Ovary 1 0.4835 0.388 0.707 0.457 0.453 0.178
Prostate 1 0.192 0.375 0.4555 0.373 0.632
SkeletalMuscle 1 0.2025 0.4105 0.128 0.44
Testes 1 0.405 0.4495 0.3435
Thyroid 1 0.49 0.204
WhiteBloodCells 1 0.242
EdomStromalCell 1
Placenta Adipose Adrenal Brain Breast Colon Heart Kidney Liver Lung LymphNode Ovary Prostate SkeletalMuscle Testes Thyroid WhiteBloodCells EdomStromalCell

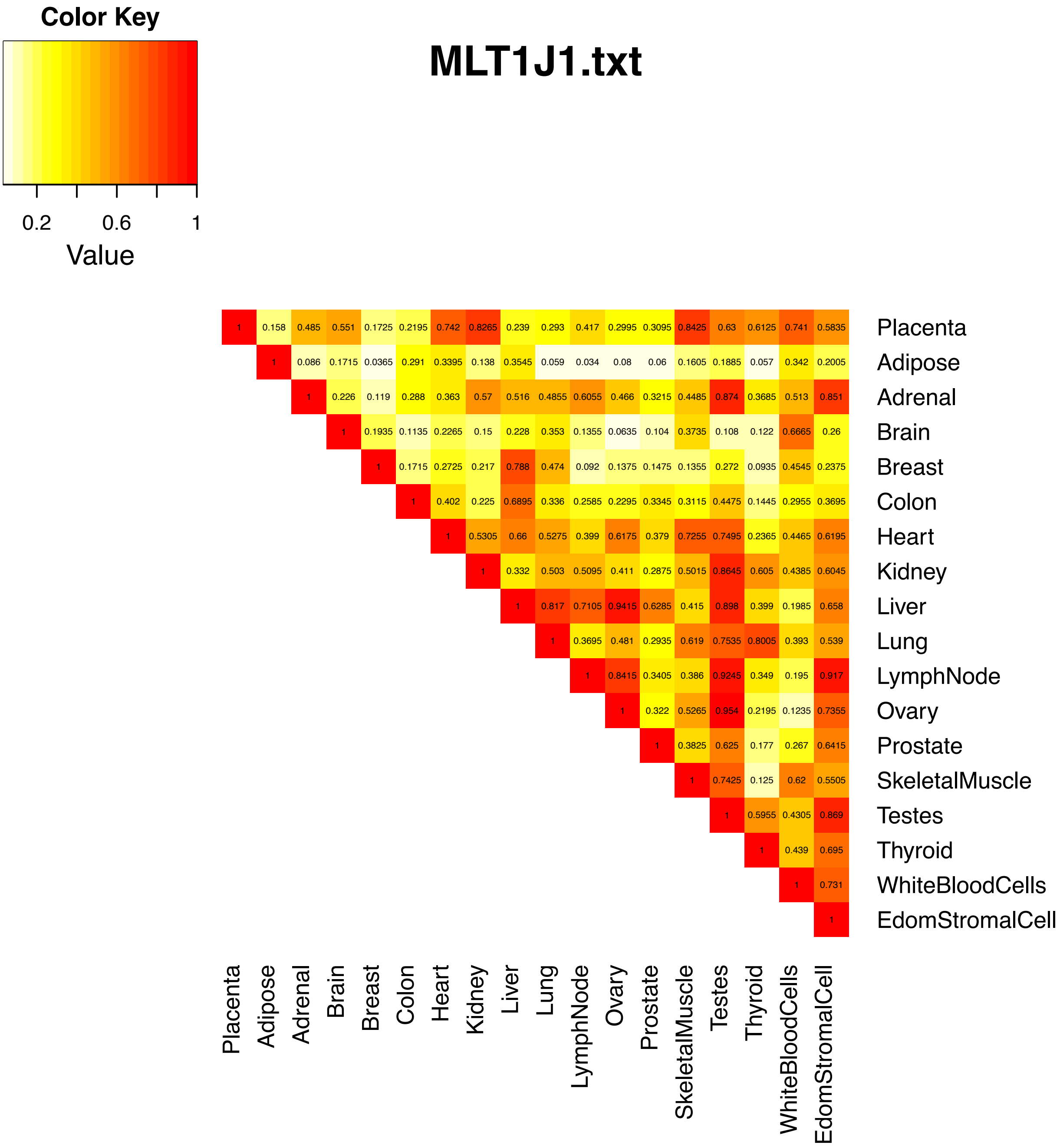

Color Key
0.2
0.6
1
Value
MLT1J1.txt
Placenta 1 0.158 0.485 0.551 0.1725 0.2195 0.742 0.8265 0.239 0.293 0.417 0.2995 0.3095 0.8425 0.63 0.6125 0.741 0.5835
Adipose 1 0.086 0.1715 0.0365 0.291 0.3395 0.138 0.3545 0.059 0.034 0.08 0.06 0.1605 0.1885 0.057 0.342 0.2005
Adrenal 1 0.226 0.119 0.288 0.363 0.57 0.516 0.4855 0.6055 0.466 0.3215 0.4485 0.874 0.3685 0.513 0.851
Brain 1 0.1935 0.1135 0.2265 0.15 0.228 0.353 0.1355 0.0635 0.104 0.3735 0.108 0.122 0.6665 0.26
Breast 1 0.1715 0.2725 0.217 0.788 0.474 0.092 0.1375 0.1475 0.1355 0.272 0.0935 0.4545 0.2375
Colon 1 0.402 0.225 0.6895 0.336 0.2585 0.2295 0.3345 0.3115 0.4475 0.1445 0.2955 0.3695
Heart 1 0.5305 0.66 0.5275 0.399 0.6175 0.379 0.7255 0.7495 0.2365 0.4465 0.6195
Kidney 1 0.332 0.503 0.5095 0.411 0.2875 0.5015 0.8645 0.605 0.4385 0.6045
Liver 1 0.817 0.7105 0.9415 0.6285 0.415 0.898 0.399 0.1985 0.658
Lung 1 0.3695 0.481 0.2935 0.619 0.7535 0.8005 0.393 0.539
LymphNode 1 0.8415 0.3405 0.386 0.9245 0.349 0.195 0.917
Ovary 1 0.322 0.5265 0.954 0.2195 0.1235 0.7355
Prostate 1 0.3825 0.625 0.177 0.267 0.6415
SkeletalMuscle 1 0.7425 0.125 0.62 0.5505
Testes 1 0.5955 0.4305 0.869
Thyroid 1 0.439 0.695
WhiteBloodCells 1 0.731
EdomStromalCell 1
Placenta
Adipose
Adrenal
Brain
Breast
Colon
Heart
Kidney
Liver
Lung
LymphNode
Ovary
Prostate
SkeletalMuscle
Testes
Thyroid
WhiteBloodCells
EdomStromalCell

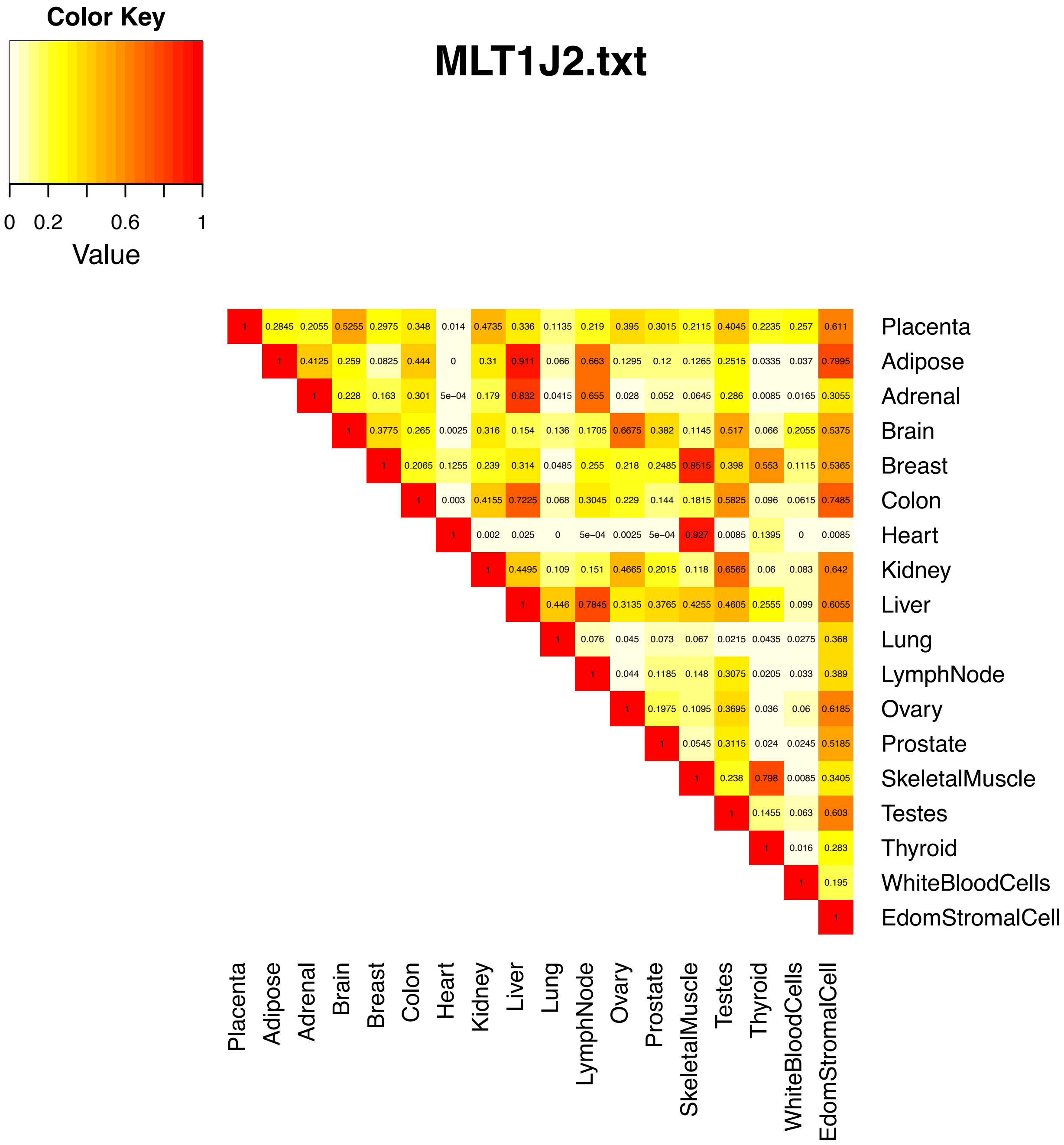

Color Key
0 0.2 0.6 1
Value
MLT1J2.txt
Placenta 1 0.2845 0.2055 0.5255 0.2975 0.348 0.014 0.4735 0.336 0.1135 0.219 0.395 0.3015 0.2115 0.4045 0.2235 0.257 0.611
Adipose 1 0.4125 0.259 0.0825 0.444 0 0.31 0.911 0.066 0.663 0.1295 0.12 0.1265 0.2515 0.0335 0.037 0.7995
Adrenal 1 0.228 0.163 0.301 5e−04 0.179 0.832 0.0415 0.655 0.028 0.052 0.0645 0.286 0.0085 0.0165 0.3055
Brain 1 0.3775 0.265 0.0025 0.316 0.154 0.136 0.1705 0.6675 0.382 0.1145 0.517 0.066 0.2055 0.5375
Breast 1 0.2065 0.1255 0.239 0.314 0.0485 0.255 0.218 0.2485 0.8515 0.398 0.553 0.1115 0.5365
Colon 1 0.003 0.4155 0.7225 0.068 0.3045 0.229 0.144 0.1815 0.5825 0.096 0.0615 0.7485
Heart 1 0.002 0.025 0 5e−04 0.0025 5e−04 0.927 0.0085 0.1395 0 0.0085
Kidney 1 0.4495 0.109 0.151 0.4665 0.2015 0.118 0.6565 0.06 0.083 0.642
Liver 1 0.446 0.7845 0.3135 0.3765 0.4255 0.4605 0.2555 0.099 0.6055
Lung 1 0.076 0.045 0.073 0.067 0.0215 0.0435 0.0275 0.368
LymphNode 1 0.044 0.1185 0.148 0.3075 0.0205 0.033 0.389
Ovary 1 0.1975 0.1095 0.3695 0.036 0.06 0.6185
Prostate 1 0.0545 0.3115 0.024 0.0245 0.5185
SkeletalMuscle 1 0.238 0.798 0.0085 0.3405
Testes 1 0.1455 0.063 0.603
Thyroid 1 0.016 0.283
WhiteBloodCells 1 0.195
EdomStromalCell 1
Placenta
Adipose
Adrenal
Brain
Breast
Colon
Heart
Kidney
Liver
Lung
LymphNode
Ovary
Prostate
SkeletalMuscle
Testes
Thyroid
WhiteBloodCells
EdomStromalCell

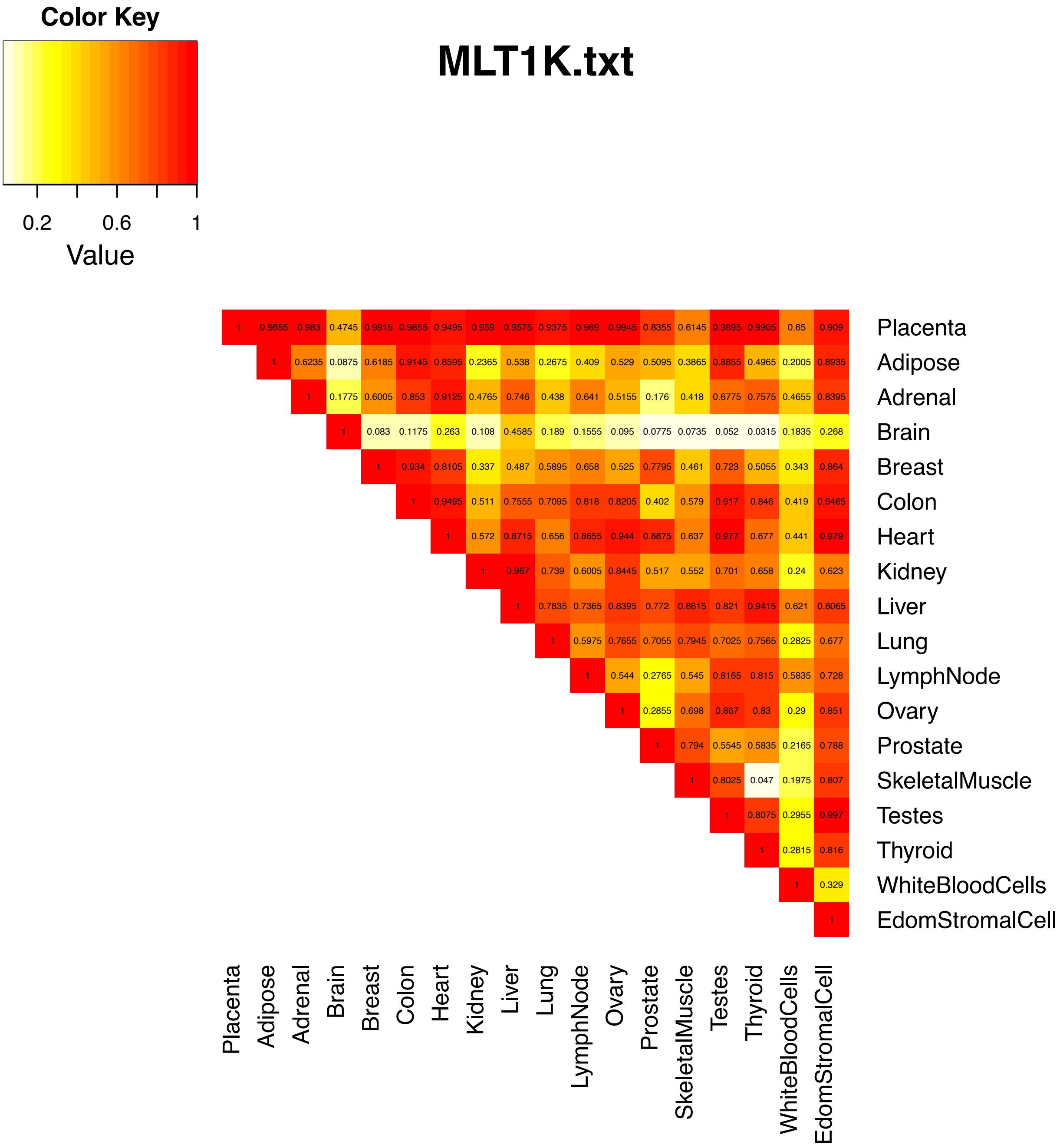
Color Key
0.2
0.6
1
Value
MLT1K.txt
Placenta 1 0.9655 0.983 0.4745 0.9915 0.9855 0.9495 0.959 0.9575 0.9375 0.969 0.9945 0.8355 0.6145 0.9895 0.9905 0.65 0.909
Adipose 1 0.6235 0.0875 0.6185 0.9145 0.8595 0.2365 0.538 0.2675 0.409 0.529 0.5095 0.3865 0.8855 0.4965 0.2005 0.8935
Adrenal 1 0.1775 0.6005 0.853 0.9125 0.4765 0.746 0.438 0.641 0.5155 0.176 0.418 0.6775 0.7575 0.4655 0.8395
Brain 1 0.083 0.1175 0.263 0.108 0.4585 0.189 0.1555 0.095 0.0775 0.0735 0.052 0.0315 0.1835 0.268
Breast 1 0.934 0.8105 0.337 0.487 0.5895 0.658 0.525 0.7795 0.461 0.723 0.5055 0.343 0.864
Colon 1 0.9495 0.511 0.7555 0.7095 0.818 0.8205 0.402 0.579 0.917 0.846 0.419 0.9465
Heart 1 0.572 0.8715 0.656 0.8655 0.944 0.8875 0.637 0.977 0.677 0.441 0.979
Kidney 1 0.967 0.739 0.6005 0.8445 0.517 0.552 0.701 0.658 0.24 0.623
Liver 1 0.7835 0.7365 0.8395 0.772 0.8615 0.821 0.9415 0.621 0.8065
Lung 1 0.5975 0.7655 0.7055 0.7945 0.7025 0.7565 0.2825 0.677
LymphNode 1 0.544 0.2765 0.545 0.8165 0.815 0.5835 0.728
Ovary 1 0.2855 0.698 0.867 0.83 0.29 0.851
Prostate 1 0.794 0.5545 0.5835 0.2165 0.788
SkeletalMuscle 1 0.8025 0.047 0.1975 0.807
Testes 1 0.8075 0.2955 0.997
Thyroid 1 0.2815 0.816
WhiteBloodCells 1 0.329
EdomStromalCell 1
Placenta
Adipose
Adrenal
Brain
Breast
Colon
Heart
Kidney
Liver
Lung
LymphNode
Ovary
Prostate
SkeletalMuscle
Testes
Thyroid
WhiteBloodCells
EdomStromalCell

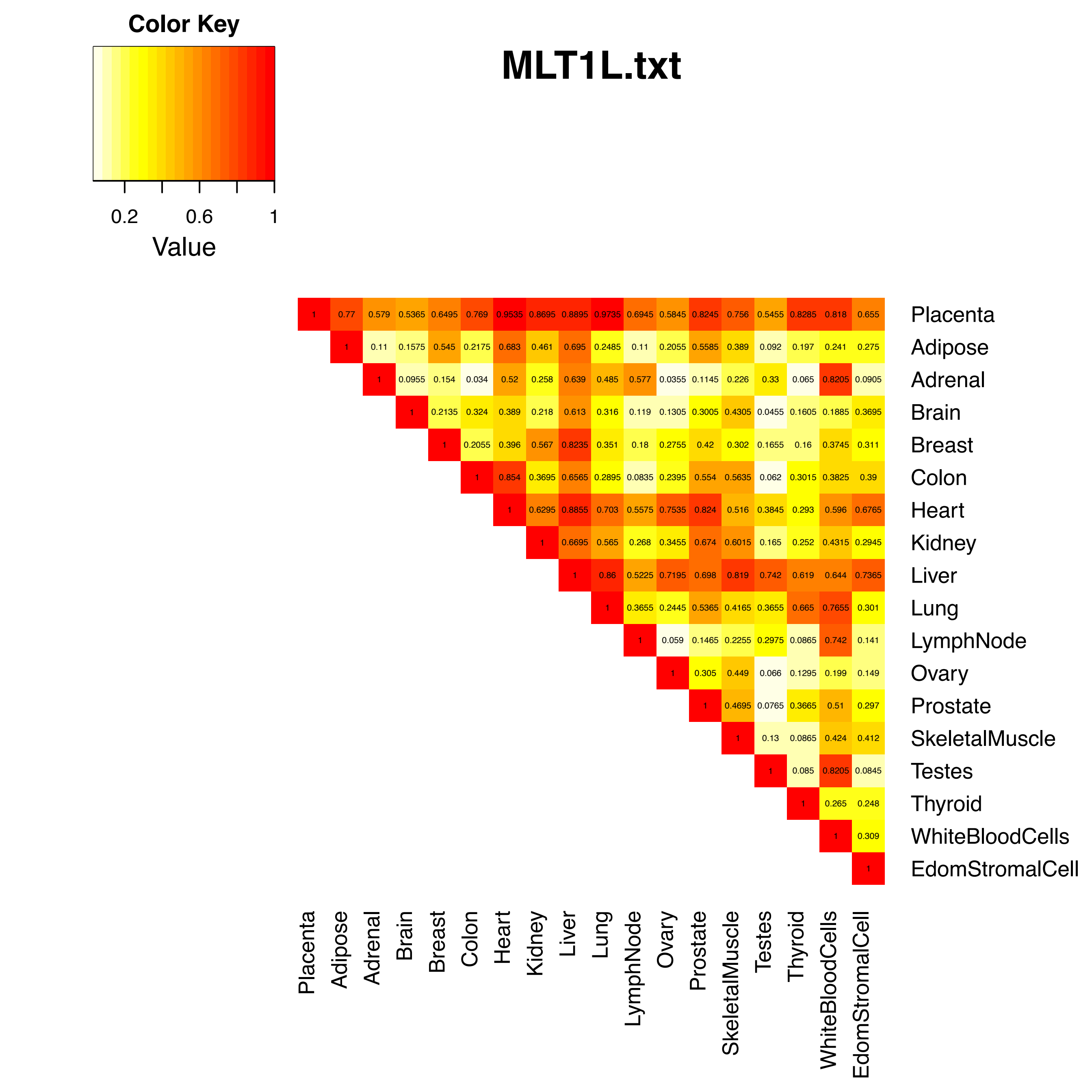
Color Key
0.2
0.6
1
Value
MLT1L.txt
Placenta 1 0.77 0.579 0.5365 0.6495 0.769 0.9535 0.8695 0.8895 0.9735 0.6945 0.5845 0.8245 0.756 0.5455 0.8285 0.818 0.655
Adipose 1 0.11 0.1575 0.545 0.2175 0.683 0.461 0.695 0.2485 0.11 0.2055 0.5585 0.389 0.092 0.197 0.241 0.275
Adrenal 1 0.0955 0.154 0.034 0.52 0.258 0.639 0.485 0.577 0.0355 0.1145 0.226 0.33 0.065 0.8205 0.0905
Brain 1 0.2135 0.324 0.389 0.218 0.613 0.316 0.119 0.1305 0.3005 0.4305 0.0455 0.1605 0.1885 0.3695
Breast 1 0.2055 0.396 0.567 0.8235 0.351 0.18 0.2755 0.42 0.302 0.1655 0.16 0.3745 0.311
Colon 1 0.854 0.3695 0.6565 0.2895 0.0835 0.2395 0.554 0.5635 0.062 0.3015 0.3825 0.39
Heart 1 0.6295 0.8855 0.703 0.5575 0.7535 0.824 0.516 0.3845 0.293 0.596 0.6765
Kidney 1 0.6695 0.565 0.268 0.3455 0.674 0.6015 0.165 0.252 0.4315 0.2945
Liver 1 0.86 0.5225 0.7195 0.698 0.819 0.742 0.619 0.644 0.7365
Lung 1 0.3655 0.2445 0.5365 0.4165 0.3655 0.665 0.7655 0.301
LymphNode 1 0.059 0.1465 0.2255 0.2975 0.0865 0.742 0.141
Ovary 1 0.305 0.449 0.066 0.1295 0.199 0.149
Prostate 1 0.4695 0.0765 0.3665 0.51 0.297
SkeletalMuscle 1 0.13 0.0865 0.424 0.412
Testes 1 0.085 0.8205 0.0845
Thyroid 1 0.265 0.248
WhiteBloodCells 1 0.309
EdomStromalCell 1
Placenta Adipose Adrenal Brain Breast Colon Heart Kidney Liver Lung LymphNode Ovary Prostate SkeletalMuscle Testes Thyroid WhiteBloodCells EdomStromalCell

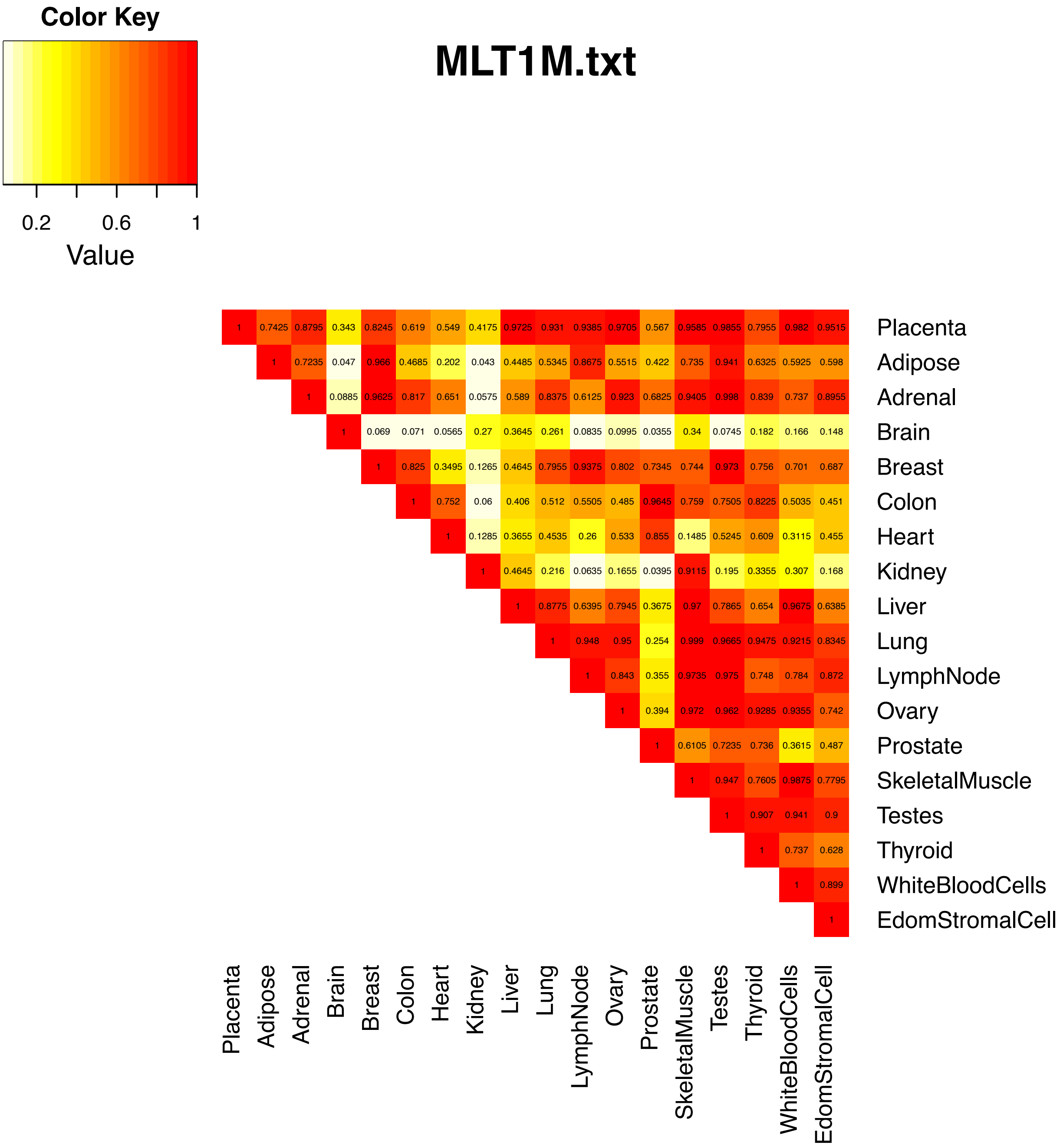
Color Key
0.2 0.6 1
Value
MLT1M.txt
Placenta: 1 0.7425 0.8795 0.343 0.8245 0.619 0.549 0.4175 0.9725 0.931 0.9385 0.9705 0.567 0.9585 0.9855 0.7955 0.982 0.9515
Adipose: 1 0.7235 0.047 0.966 0.4685 0.202 0.043 0.4485 0.5345 0.8675 0.5515 0.422 0.735 0.941 0.6325 0.5925 0.598
Adrenal: 1 0.0885 0.9625 0.817 0.651 0.0575 0.589 0.8375 0.6125 0.923 0.6825 0.9405 0.998 0.839 0.737 0.8955
Brain: 1 0.069 0.071 0.0565 0.27 0.3645 0.261 0.0835 0.0995 0.0355 0.34 0.0745 0.182 0.166 0.148
Breast: 1 0.825 0.3495 0.1265 0.4645 0.7955 0.9375 0.802 0.7345 0.744 0.973 0.756 0.701 0.687
Colon: 1 0.752 0.06 0.406 0.512 0.5505 0.485 0.9645 0.759 0.7505 0.8225 0.5035 0.451
Heart: 1 0.1285 0.3655 0.4535 0.26 0.533 0.855 0.1485 0.5245 0.609 0.3115 0.455
Kidney: 1 0.4645 0.216 0.0635 0.1655 0.0395 0.9115 0.195 0.3355 0.307 0.168
Liver: 1 0.8775 0.6395 0.7945 0.3675 0.97 0.7865 0.654 0.9675 0.6385
Lung: 1 0.948 0.95 0.254 0.999 0.9665 0.9475 0.9215 0.8345
LymphNode: 1 0.843 0.355 0.9735 0.975 0.748 0.784 0.872
Ovary: 1 0.394 0.972 0.962 0.9285 0.9355 0.742
Prostate: 1 0.6105 0.7235 0.736 0.3615 0.487
SkeletalMuscle: 1 0.947 0.7605 0.9875 0.7795
Testes: 1 0.907 0.941 0.9
Thyroid: 1 0.737 0.628
WhiteBloodCells: 1 0.899
EdomStromalCell: 1
Placenta Adipose Adrenal Brain Breast Colon Heart Kidney Liver Lung LymphNode Ovary Prostate SkeletalMuscle Testes Thyroid WhiteBloodCells EdomStromalCell

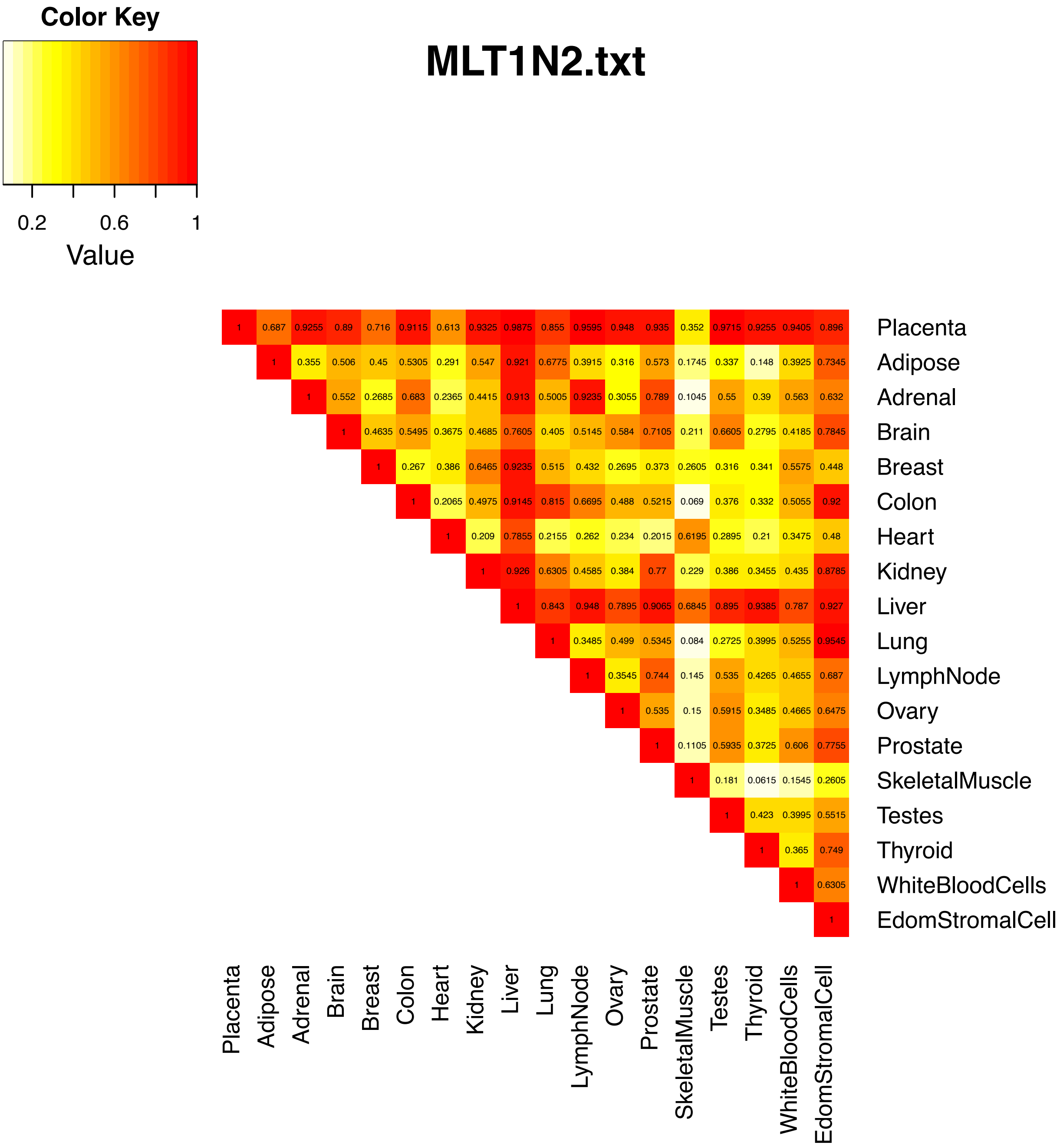
Color Key
0.2
0.6
1
Value
MLT1N2.txt
Placenta 1 0.687 0.9255 0.89 0.716 0.9115 0.613 0.9325 0.9875 0.855 0.9595 0.948 0.935 0.352 0.9715 0.9255 0.9405 0.896
Adipose 1 0.355 0.506 0.45 0.5305 0.291 0.547 0.921 0.6775 0.3915 0.316 0.573 0.1745 0.337 0.148 0.3925 0.7345
Adrenal 1 0.552 0.2685 0.683 0.2365 0.4415 0.913 0.5005 0.9235 0.3055 0.789 0.1045 0.55 0.39 0.563 0.632
Brain 1 0.4635 0.5495 0.3675 0.4685 0.7605 0.405 0.5145 0.584 0.7105 0.211 0.6605 0.2795 0.4185 0.7845
Breast 1 0.267 0.386 0.6465 0.9235 0.515 0.432 0.2695 0.373 0.2605 0.316 0.341 0.5575 0.448
Colon 1 0.2065 0.4975 0.9145 0.815 0.6695 0.488 0.5215 0.069 0.376 0.332 0.5055 0.92
Heart 1 0.209 0.7855 0.2155 0.262 0.234 0.2015 0.6195 0.2895 0.21 0.3475 0.48
Kidney 1 0.926 0.6305 0.4585 0.384 0.77 0.229 0.386 0.3455 0.435 0.8785
Liver 1 0.843 0.948 0.7895 0.9065 0.6845 0.895 0.9385 0.787 0.927
Lung 1 0.3485 0.499 0.5345 0.084 0.2725 0.3995 0.5255 0.9545
LymphNode 1 0.3545 0.744 0.145 0.535 0.4265 0.4655 0.687
Ovary 1 0.535 0.15 0.5915 0.3485 0.4665 0.6475
Prostate 1 0.1105 0.5935 0.3725 0.606 0.7755
SkeletalMuscle 1 0.181 0.0615 0.1545 0.2605
Testes 1 0.423 0.3995 0.5515
Thyroid 1 0.365 0.749
WhiteBloodCells 1 0.6305
EdomStromalCell 1
Placenta
Adipose
Adrenal
Brain
Breast
Colon
Heart
Kidney
Liver
Lung
LymphNode
Ovary
Prostate
SkeletalMuscle
Testes
Thyroid
WhiteBloodCells
EdomStromalCell

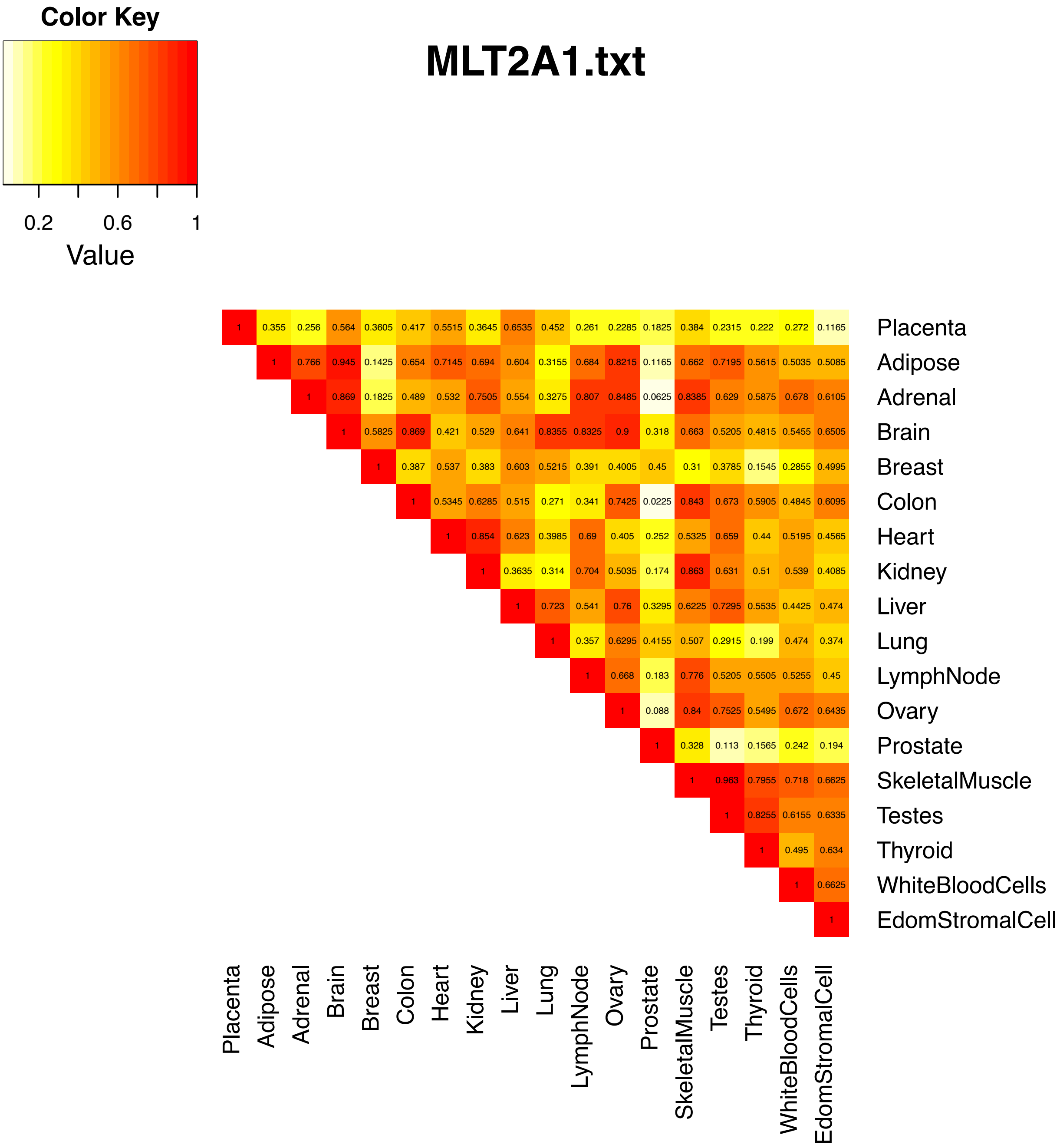

Color Key
0.2 0.6 1
Value
MLT2A1.txt
Placenta: 1 0.355 0.256 0.564 0.3605 0.417 0.5515 0.3645 0.6535 0.452 0.261 0.2285 0.1825 0.384 0.2315 0.222 0.272 0.1165
Adipose: 1 0.766 0.945 0.1425 0.654 0.7145 0.694 0.604 0.3155 0.684 0.8215 0.1165 0.662 0.7195 0.5615 0.5035 0.5085
Adrenal: 1 0.869 0.1825 0.489 0.532 0.7505 0.554 0.3275 0.807 0.8485 0.0625 0.8385 0.629 0.5875 0.678 0.6105
Brain: 1 0.5825 0.869 0.421 0.529 0.641 0.8355 0.8325 0.9 0.318 0.663 0.5205 0.4815 0.5455 0.6505
Breast: 1 0.387 0.537 0.383 0.603 0.5215 0.391 0.4005 0.45 0.31 0.3785 0.1545 0.2855 0.4995
Colon: 1 0.5345 0.6285 0.515 0.271 0.341 0.7425 0.0225 0.843 0.673 0.5905 0.4845 0.6095
Heart: 1 0.854 0.623 0.3985 0.69 0.405 0.252 0.5325 0.659 0.44 0.5195 0.4565
Kidney: 1 0.3635 0.314 0.704 0.5035 0.174 0.863 0.631 0.51 0.539 0.4085
Liver: 1 0.723 0.541 0.76 0.3295 0.6225 0.7295 0.5535 0.4425 0.474
Lung: 1 0.357 0.6295 0.4155 0.507 0.2915 0.199 0.474 0.374
LymphNode: 1 0.668 0.183 0.776 0.5205 0.5505 0.5255 0.45
Ovary: 1 0.088 0.84 0.7525 0.5495 0.672 0.6435
Prostate: 1 0.328 0.113 0.1565 0.242 0.194
SkeletalMuscle: 1 0.963 0.7955 0.718 0.6625
Testes: 1 0.8255 0.6155 0.6335
Thyroid: 1 0.495 0.634
WhiteBloodCells: 1 0.6625
EdomStromalCell: 1
Placenta Adipose Adrenal Brain Breast Colon Heart Kidney Liver Lung LymphNode Ovary Prostate SkeletalMuscle Testes Thyroid WhiteBloodCells EdomStromalCell

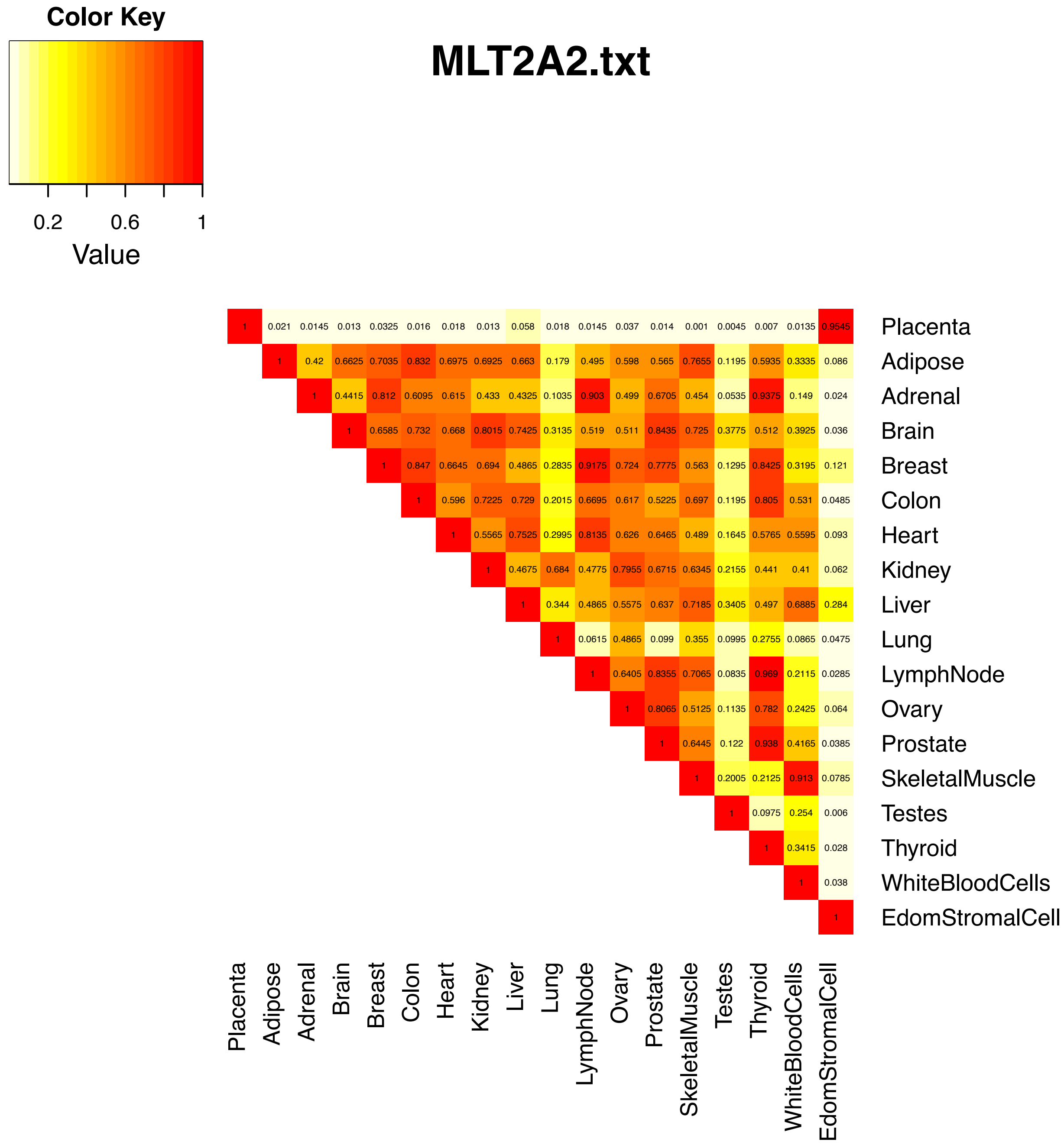
Color Key
0.2
0.6
1
Value
MLT2A2.txt
1 0.021 0.0145 0.013 0.0325 0.016 0.018 0.013 0.058 0.018 0.0145 0.037 0.014 0.001 0.0045 0.007 0.0135 0.9545 Placenta
1 0.42 0.6625 0.7035 0.832 0.6975 0.6925 0.663 0.179 0.495 0.598 0.565 0.7655 0.1195 0.5935 0.3335 0.086 Adipose
1 0.4415 0.812 0.6095 0.615 0.433 0.4325 0.1035 0.903 0.499 0.6705 0.454 0.0535 0.9375 0.149 0.024 Adrenal
1 0.6585 0.732 0.668 0.8015 0.7425 0.3135 0.519 0.511 0.8435 0.725 0.3775 0.512 0.3925 0.036 Brain
1 0.847 0.6645 0.694 0.4865 0.2835 0.9175 0.724 0.7775 0.563 0.1295 0.8425 0.3195 0.121 Breast
1 0.596 0.7225 0.729 0.2015 0.6695 0.617 0.5225 0.697 0.1195 0.805 0.531 0.0485 Colon
1 0.5565 0.7525 0.2995 0.8135 0.626 0.6465 0.489 0.1645 0.5765 0.5595 0.093 Heart
1 0.4675 0.684 0.4775 0.7955 0.6715 0.6345 0.2155 0.441 0.41 0.062 Kidney
1 0.344 0.4865 0.5575 0.637 0.7185 0.3405 0.497 0.6885 0.284 Liver
1 0.0615 0.4865 0.099 0.355 0.0995 0.2755 0.0865 0.0475 Lung
1 0.6405 0.8355 0.7065 0.0835 0.969 0.2115 0.0285 LymphNode
1 0.8065 0.5125 0.1135 0.782 0.2425 0.064 Ovary
1 0.6445 0.122 0.938 0.4165 0.0385 Prostate
1 0.2005 0.2125 0.913 0.0785 SkeletalMuscle
1 0.0975 0.254 0.006 Testes
1 0.3415 0.028 Thyroid
1 0.038 WhiteBloodCells
1 EdomStromalCell
Placenta
Adipose
Adrenal
Brain
Breast
Colon
Heart
Kidney
Liver
Lung
LymphNode
Ovary
Prostate
SkeletalMuscle
Testes
Thyroid
WhiteBloodCells
EdomStromalCell

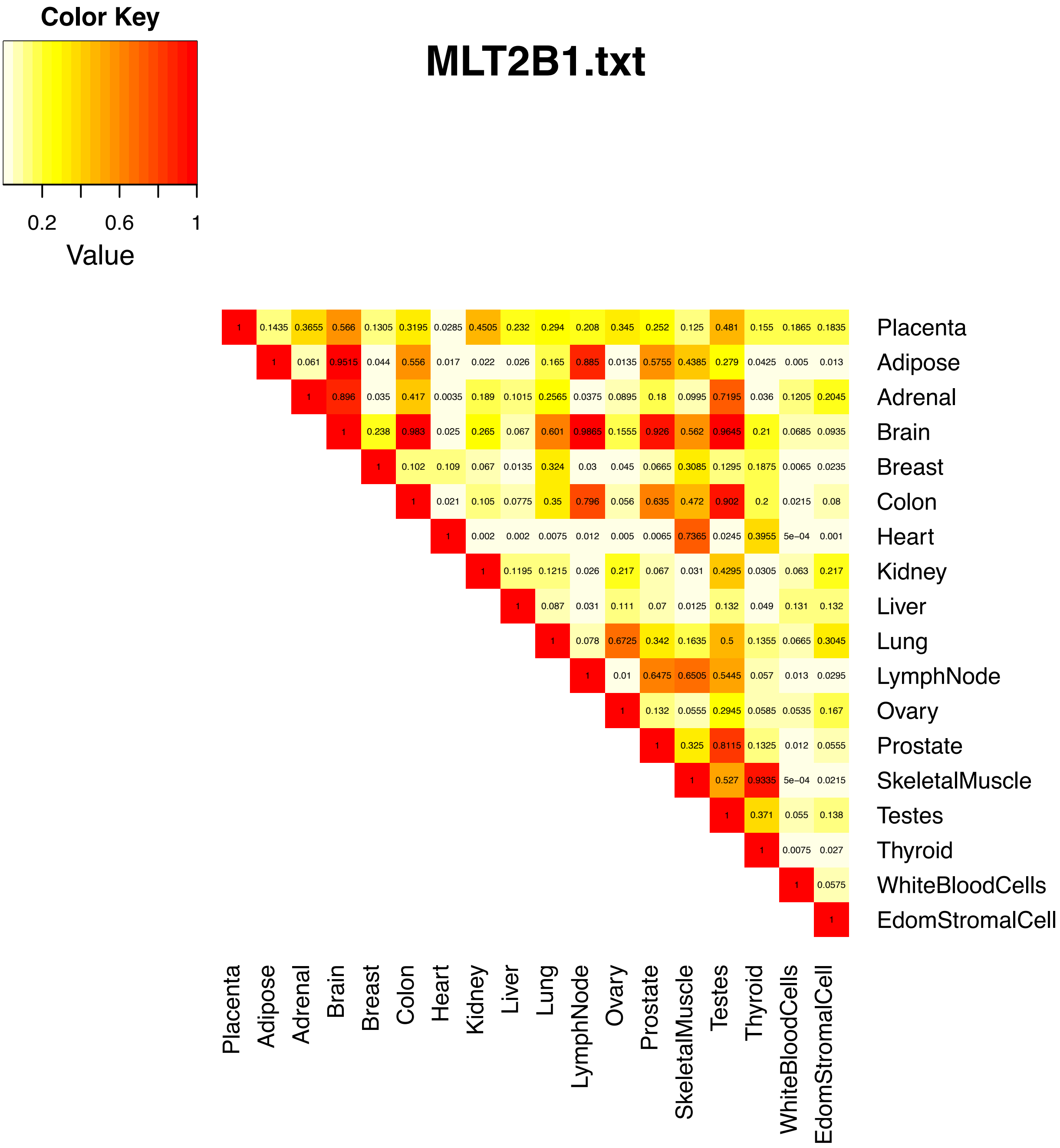
Color Key
0.2
0.6
1
Value
MLT2B1.txt
1 0.1435 0.3655 0.566 0.1305 0.3195 0.0285 0.4505 0.232 0.294 0.208 0.345 0.252 0.125 0.481 0.155 0.1865 0.1835 Placenta
1 0.061 0.9515 0.044 0.556 0.017 0.022 0.026 0.165 0.885 0.0135 0.5755 0.4385 0.279 0.0425 0.005 0.013 Adipose
1 0.896 0.035 0.417 0.0035 0.189 0.1015 0.2565 0.0375 0.0895 0.18 0.0995 0.7195 0.036 0.1205 0.2045 Adrenal
1 0.238 0.983 0.025 0.265 0.067 0.601 0.9865 0.1555 0.926 0.562 0.9645 0.21 0.0685 0.0935 Brain
1 0.102 0.109 0.067 0.0135 0.324 0.03 0.045 0.0665 0.3085 0.1295 0.1875 0.0065 0.0235 Breast
1 0.021 0.105 0.0775 0.35 0.796 0.056 0.635 0.472 0.902 0.2 0.0215 0.08 Colon
1 0.002 0.002 0.0075 0.012 0.005 0.0065 0.7365 0.0245 0.3955 5e−04 0.001 Heart
1 0.1195 0.1215 0.026 0.217 0.067 0.031 0.4295 0.0305 0.063 0.217 Kidney
1 0.087 0.031 0.111 0.07 0.0125 0.132 0.049 0.131 0.132 Liver
1 0.078 0.6725 0.342 0.1635 0.5 0.1355 0.0665 0.3045 Lung
1 0.01 0.6475 0.6505 0.5445 0.057 0.013 0.0295 LymphNode
1 0.132 0.0555 0.2945 0.0585 0.0535 0.167 Ovary
1 0.325 0.8115 0.1325 0.012 0.0555 Prostate
1 0.527 0.9335 5e−04 0.0215 SkeletalMuscle
1 0.371 0.055 0.138 Testes
1 0.0075 0.027 Thyroid
1 0.0575 WhiteBloodCells
1 EdomStromalCell
Placenta Adipose Adrenal Brain Breast Colon Heart Kidney Liver Lung LymphNode Ovary Prostate SkeletalMuscle Testes Thyroid WhiteBloodCells EdomStromalCell

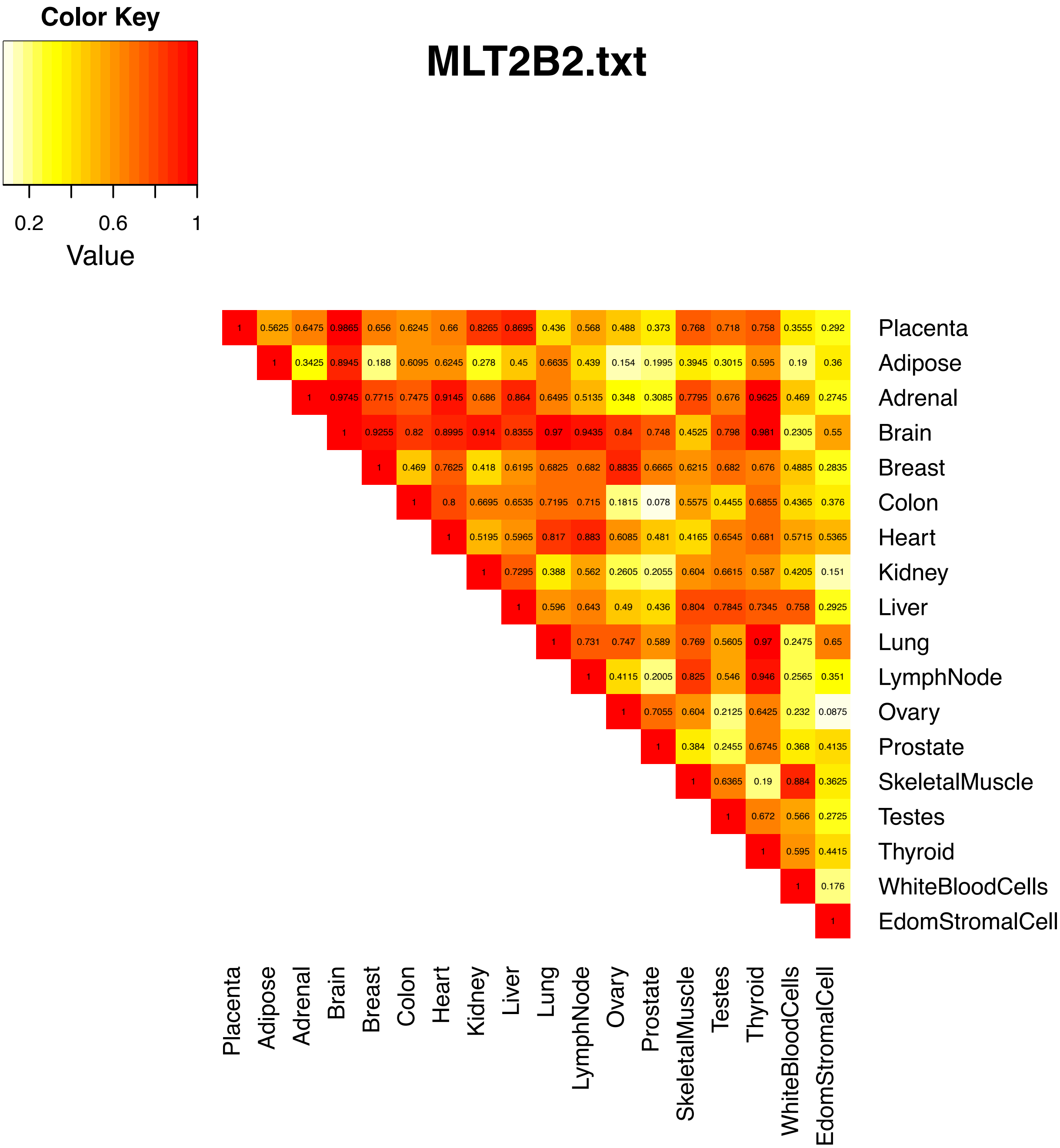
Color Key
0.2 0.6 1
Value
MLT2B2.txt
Placenta: 1 0.5625 0.6475 0.9865 0.656 0.6245 0.66 0.8265 0.8695 0.436 0.568 0.488 0.373 0.768 0.718 0.758 0.3555 0.292
Adipose: 1 0.3425 0.8945 0.188 0.6095 0.6245 0.278 0.45 0.6635 0.439 0.154 0.1995 0.3945 0.3015 0.595 0.19 0.36
Adrenal: 1 0.9745 0.7715 0.7475 0.9145 0.686 0.864 0.6495 0.5135 0.348 0.3085 0.7795 0.676 0.9625 0.469 0.2745
Brain: 1 0.9255 0.82 0.8995 0.914 0.8355 0.97 0.9435 0.84 0.748 0.4525 0.798 0.981 0.2305 0.55
Breast: 1 0.469 0.7625 0.418 0.6195 0.6825 0.682 0.8835 0.6665 0.6215 0.682 0.676 0.4885 0.2835
Colon: 1 0.8 0.6695 0.6535 0.7195 0.715 0.1815 0.078 0.5575 0.4455 0.6855 0.4365 0.376
Heart: 1 0.5195 0.5965 0.817 0.883 0.6085 0.481 0.4165 0.6545 0.681 0.5715 0.5365
Kidney: 1 0.7295 0.388 0.562 0.2605 0.2055 0.604 0.6615 0.587 0.4205 0.151
Liver: 1 0.596 0.643 0.49 0.436 0.804 0.7845 0.7345 0.758 0.2925
Lung: 1 0.731 0.747 0.589 0.769 0.5605 0.97 0.2475 0.65
LymphNode: 1 0.4115 0.2005 0.825 0.546 0.946 0.2565 0.351
Ovary: 1 0.7055 0.604 0.2125 0.6425 0.232 0.0875
Prostate: 1 0.384 0.2455 0.6745 0.368 0.4135
SkeletalMuscle: 1 0.6365 0.19 0.884 0.3625
Testes: 1 0.672 0.566 0.2725
Thyroid: 1 0.595 0.4415
WhiteBloodCells: 1 0.176
EdomStromalCell: 1
Placenta Adipose Adrenal Brain Breast Colon Heart Kidney Liver Lung LymphNode Ovary Prostate SkeletalMuscle Testes Thyroid WhiteBloodCells EdomStromalCell

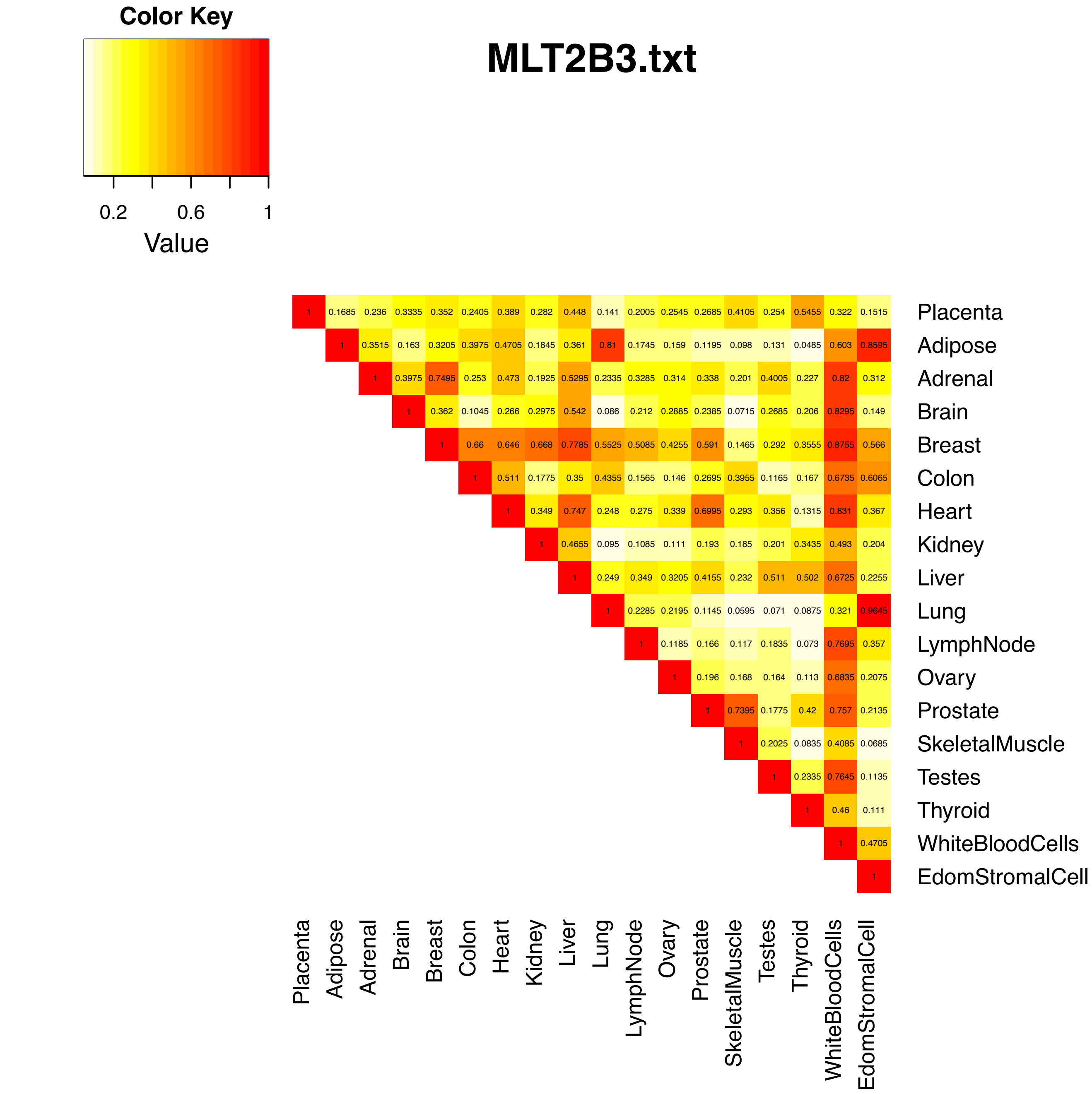
MLT2B3.txt
Color Key
0.2
0.6
1
Value
Placenta 1 0.1685 0.236 0.3335 0.352 0.2405 0.389 0.282 0.448 0.141 0.2005 0.2545 0.2685 0.4105 0.254 0.5455 0.322 0.1515
Adipose 1 0.3515 0.163 0.3205 0.3975 0.4705 0.1845 0.361 0.81 0.1745 0.159 0.1195 0.098 0.131 0.0485 0.603 0.8595
Adrenal 1 0.3975 0.7495 0.253 0.473 0.1925 0.5295 0.2335 0.3285 0.314 0.338 0.201 0.4005 0.227 0.82 0.312
Brain 1 0.362 0.1045 0.266 0.2975 0.542 0.086 0.212 0.2885 0.2385 0.0715 0.2685 0.206 0.8295 0.149
Breast 1 0.66 0.646 0.668 0.7785 0.5525 0.5085 0.4255 0.591 0.1465 0.292 0.3555 0.8755 0.566
Colon 1 0.511 0.1775 0.35 0.4355 0.1565 0.146 0.2695 0.3955 0.1165 0.167 0.6735 0.6065
Heart 1 0.349 0.747 0.248 0.275 0.339 0.6995 0.293 0.356 0.1315 0.831 0.367
Kidney 1 0.4655 0.095 0.1085 0.111 0.193 0.185 0.201 0.3435 0.493 0.204
Liver 1 0.249 0.349 0.3205 0.4155 0.232 0.511 0.502 0.6725 0.2255
Lung 1 0.2285 0.2195 0.1145 0.0595 0.071 0.0875 0.321 0.9645
LymphNode 1 0.1185 0.166 0.117 0.1835 0.073 0.7695 0.357
Ovary 1 0.196 0.168 0.164 0.113 0.6835 0.2075
Prostate 1 0.7395 0.1775 0.42 0.757 0.2135
SkeletalMuscle 1 0.2025 0.0835 0.4085 0.0685
Testes 1 0.2335 0.7645 0.1135
Thyroid 1 0.46 0.111
WhiteBloodCells 1 0.4705
EdomStromalCell 1
Placenta
Adipose
Adrenal
Brain
Breast
Colon
Heart
Kidney
Liver
Lung
LymphNode
Ovary
Prostate
SkeletalMuscle
Testes
Thyroid
WhiteBloodCells
EdomStromalCell

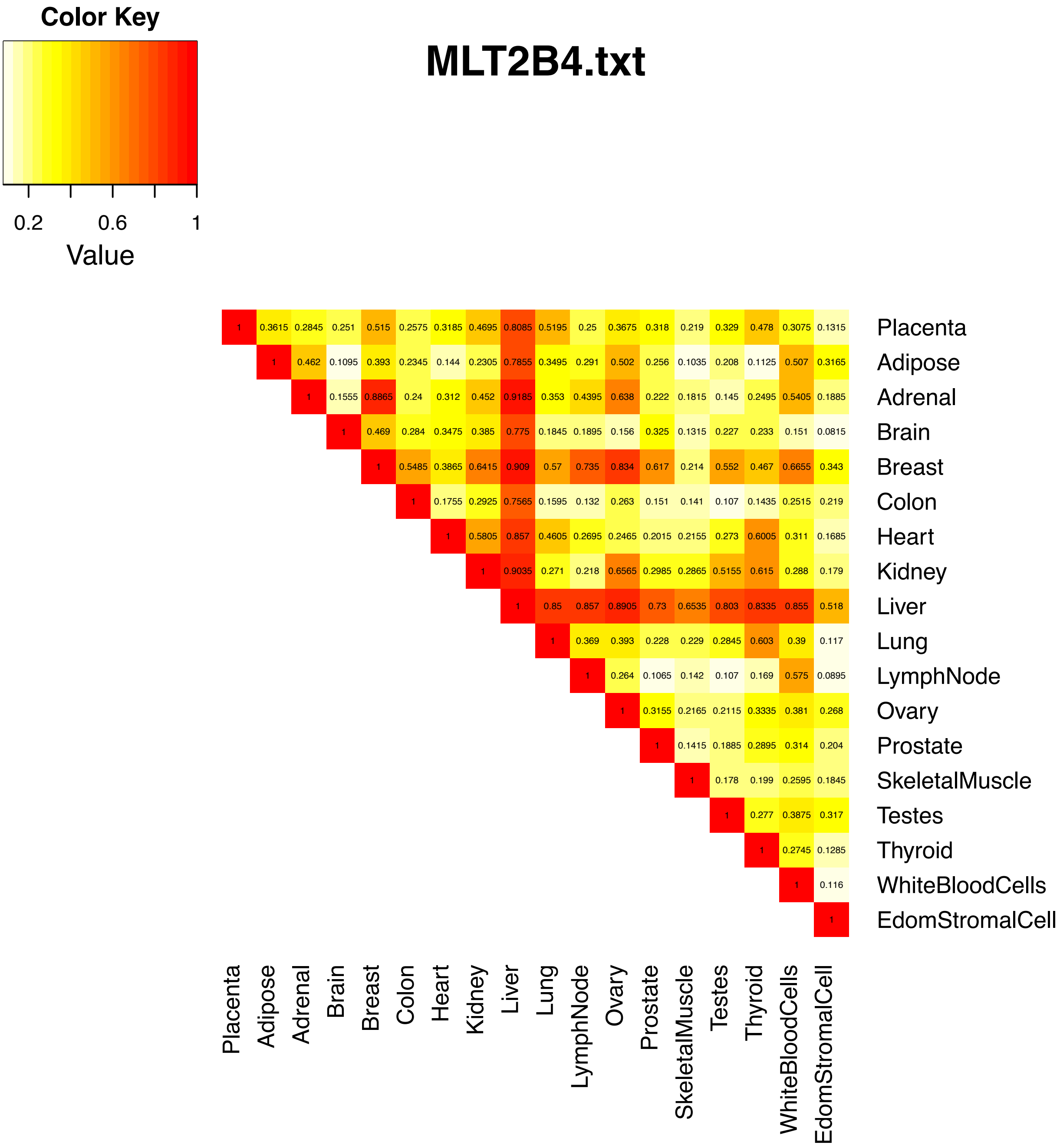
Color Key
0.2 0.6 1
Value
MLT2B4.txt
Placenta 1 0.3615 0.2845 0.251 0.515 0.2575 0.3185 0.4695 0.8085 0.5195 0.25 0.3675 0.318 0.219 0.329 0.478 0.3075 0.1315
Adipose 1 0.462 0.1095 0.393 0.2345 0.144 0.2305 0.7855 0.3495 0.291 0.502 0.256 0.1035 0.208 0.1125 0.507 0.3165
Adrenal 1 0.1555 0.8865 0.24 0.312 0.452 0.9185 0.353 0.4395 0.638 0.222 0.1815 0.145 0.2495 0.5405 0.1885
Brain 1 0.469 0.284 0.3475 0.385 0.775 0.1845 0.1895 0.156 0.325 0.1315 0.227 0.233 0.151 0.0815
Breast 1 0.5485 0.3865 0.6415 0.909 0.57 0.735 0.834 0.617 0.214 0.552 0.467 0.6655 0.343
Colon 1 0.1755 0.2925 0.7565 0.1595 0.132 0.263 0.151 0.141 0.107 0.1435 0.2515 0.219
Heart 1 0.5805 0.857 0.4605 0.2695 0.2465 0.2015 0.2155 0.273 0.6005 0.311 0.1685
Kidney 1 0.9035 0.271 0.218 0.6565 0.2985 0.2865 0.5155 0.615 0.288 0.179
Liver 1 0.85 0.857 0.8905 0.73 0.6535 0.803 0.8335 0.855 0.518
Lung 1 0.369 0.393 0.228 0.229 0.2845 0.603 0.39 0.117
LymphNode 1 0.264 0.1065 0.142 0.107 0.169 0.575 0.0895
Ovary 1 0.3155 0.2165 0.2115 0.3335 0.381 0.268
Prostate 1 0.1415 0.1885 0.2895 0.314 0.204
SkeletalMuscle 1 0.178 0.199 0.2595 0.1845
Testes 1 0.277 0.3875 0.317
Thyroid 1 0.2745 0.1285
WhiteBloodCells 1 0.116
EdomStromalCell 1
Placenta Adipose Adrenal Brain Breast Colon Heart Kidney Liver Lung LymphNode Ovary Prostate SkeletalMuscle Testes Thyroid WhiteBloodCells EdomStromalCell

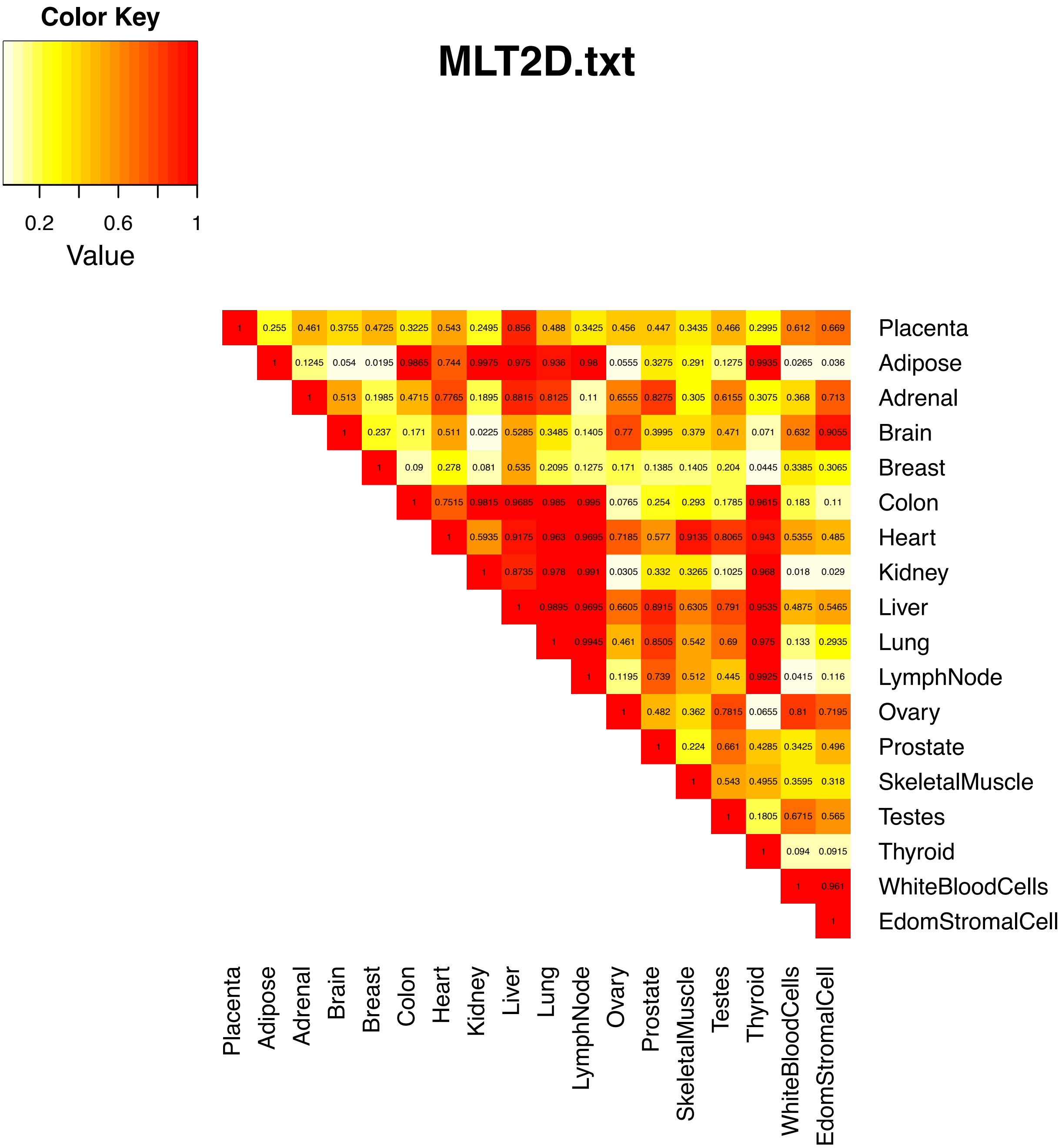
Color Key
0.2 0.6 1
Value
MLT2D.txt
1 0.255 0.461 0.3755 0.4725 0.3225 0.543 0.2495 0.856 0.488 0.3425 0.456 0.447 0.3435 0.466 0.2995 0.612 0.669 Placenta
1 0.1245 0.054 0.0195 0.9865 0.744 0.9975 0.975 0.936 0.98 0.0555 0.3275 0.291 0.1275 0.9935 0.0265 0.036 Adipose
1 0.513 0.1985 0.4715 0.7765 0.1895 0.8815 0.8125 0.11 0.6555 0.8275 0.305 0.6155 0.3075 0.368 0.713 Adrenal
1 0.237 0.171 0.511 0.0225 0.5285 0.3485 0.1405 0.77 0.3995 0.379 0.471 0.071 0.632 0.9055 Brain
1 0.09 0.278 0.081 0.535 0.2095 0.1275 0.171 0.1385 0.1405 0.204 0.0445 0.3385 0.3065 Breast
1 0.7515 0.9815 0.9685 0.985 0.995 0.0765 0.254 0.293 0.1785 0.9615 0.183 0.11 Colon
1 0.5935 0.9175 0.963 0.9695 0.7185 0.577 0.9135 0.8065 0.943 0.5355 0.485 Heart
1 0.8735 0.978 0.991 0.0305 0.332 0.3265 0.1025 0.968 0.018 0.029 Kidney
1 0.9895 0.9695 0.6605 0.8915 0.6305 0.791 0.9535 0.4875 0.5465 Liver
1 0.9945 0.461 0.8505 0.542 0.69 0.975 0.133 0.2935 Lung
1 0.1195 0.739 0.512 0.445 0.9925 0.0415 0.116 LymphNode
1 0.482 0.362 0.7815 0.0655 0.81 0.7195 Ovary
1 0.224 0.661 0.4285 0.3425 0.496 Prostate
1 0.543 0.4955 0.3595 0.318 SkeletalMuscle
1 0.1805 0.6715 0.565 Testes
1 0.094 0.0915 Thyroid
1 0.961 WhiteBloodCells
1 EdomStromalCell
Placenta Adipose Adrenal Brain Breast Colon Heart Kidney Liver Lung LymphNode Ovary Prostate SkeletalMuscle Testes Thyroid WhiteBloodCells EdomStromalCell

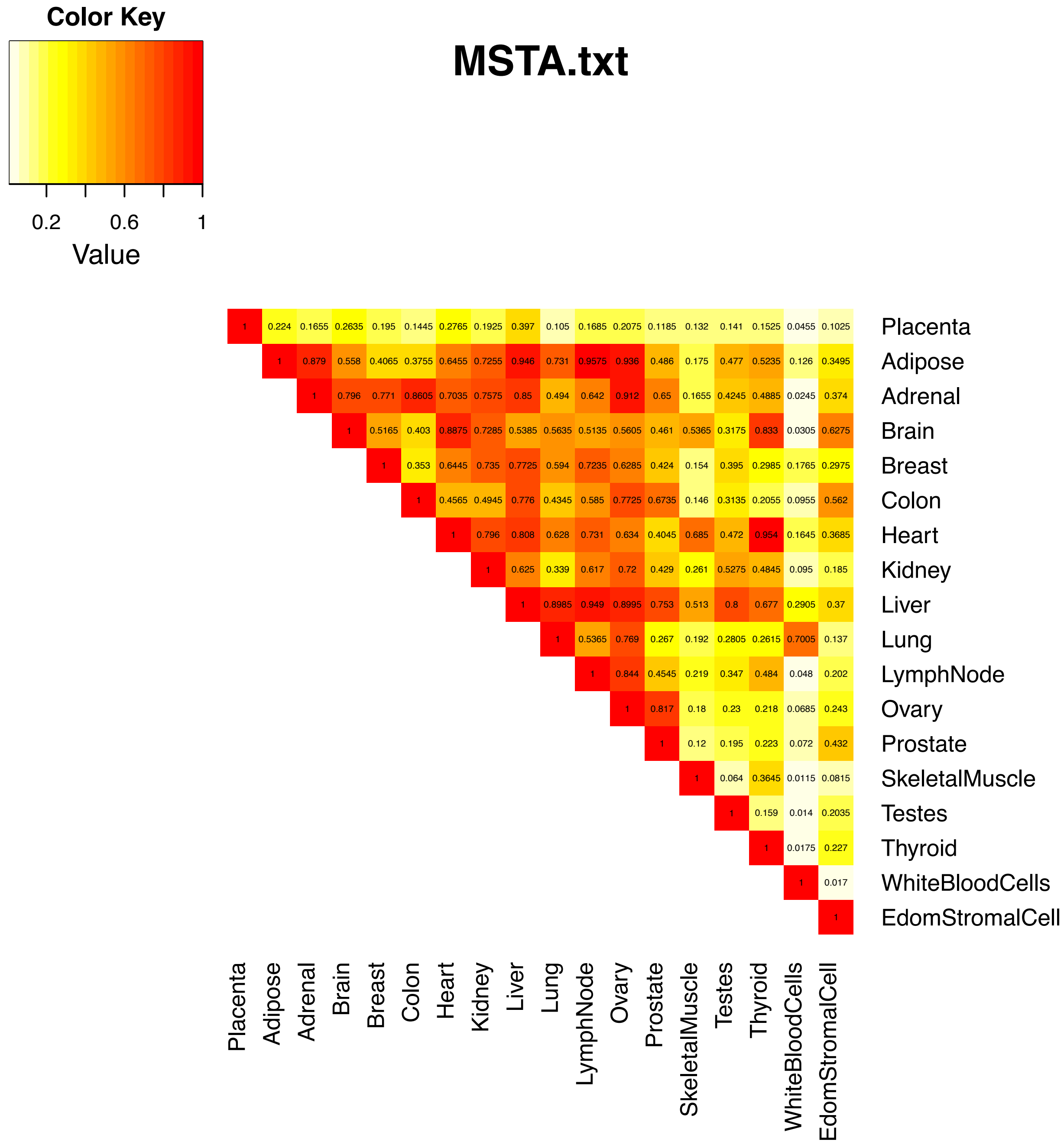
Color Key
0.2
0.6
1
Value
MSTA.txt
Placenta 1 0.224 0.1655 0.2635 0.195 0.1445 0.2765 0.1925 0.397 0.105 0.1685 0.2075 0.1185 0.132 0.141 0.1525 0.0455 0.1025
Adipose 1 0.879 0.558 0.4065 0.3755 0.6455 0.7255 0.946 0.731 0.9575 0.936 0.486 0.175 0.477 0.5235 0.126 0.3495
Adrenal 1 0.796 0.771 0.8605 0.7035 0.7575 0.85 0.494 0.642 0.912 0.65 0.1655 0.4245 0.4885 0.0245 0.374
Brain 1 0.5165 0.403 0.8875 0.7285 0.5385 0.5635 0.5135 0.5605 0.461 0.5365 0.3175 0.833 0.0305 0.6275
Breast 1 0.353 0.6445 0.735 0.7725 0.594 0.7235 0.6285 0.424 0.154 0.395 0.2985 0.1765 0.2975
Colon 1 0.4565 0.4945 0.776 0.4345 0.585 0.7725 0.6735 0.146 0.3135 0.2055 0.0955 0.562
Heart 1 0.796 0.808 0.628 0.731 0.634 0.4045 0.685 0.472 0.954 0.1645 0.3685
Kidney 1 0.625 0.339 0.617 0.72 0.429 0.261 0.5275 0.4845 0.095 0.185
Liver 1 0.8985 0.949 0.8995 0.753 0.513 0.8 0.677 0.2905 0.37
Lung 1 0.5365 0.769 0.267 0.192 0.2805 0.2615 0.7005 0.137
LymphNode 1 0.844 0.4545 0.219 0.347 0.484 0.048 0.202
Ovary 1 0.817 0.18 0.23 0.218 0.0685 0.243
Prostate 1 0.12 0.195 0.223 0.072 0.432
SkeletalMuscle 1 0.064 0.3645 0.0115 0.0815
Testes 1 0.159 0.014 0.2035
Thyroid 1 0.0175 0.227
WhiteBloodCells 1 0.017
EdomStromalCell 1
Placenta
Adipose
Adrenal
Brain
Breast
Colon
Heart
Kidney
Liver
Lung
LymphNode
Ovary
Prostate
SkeletalMuscle
Testes
Thyroid
WhiteBloodCells
EdomStromalCell

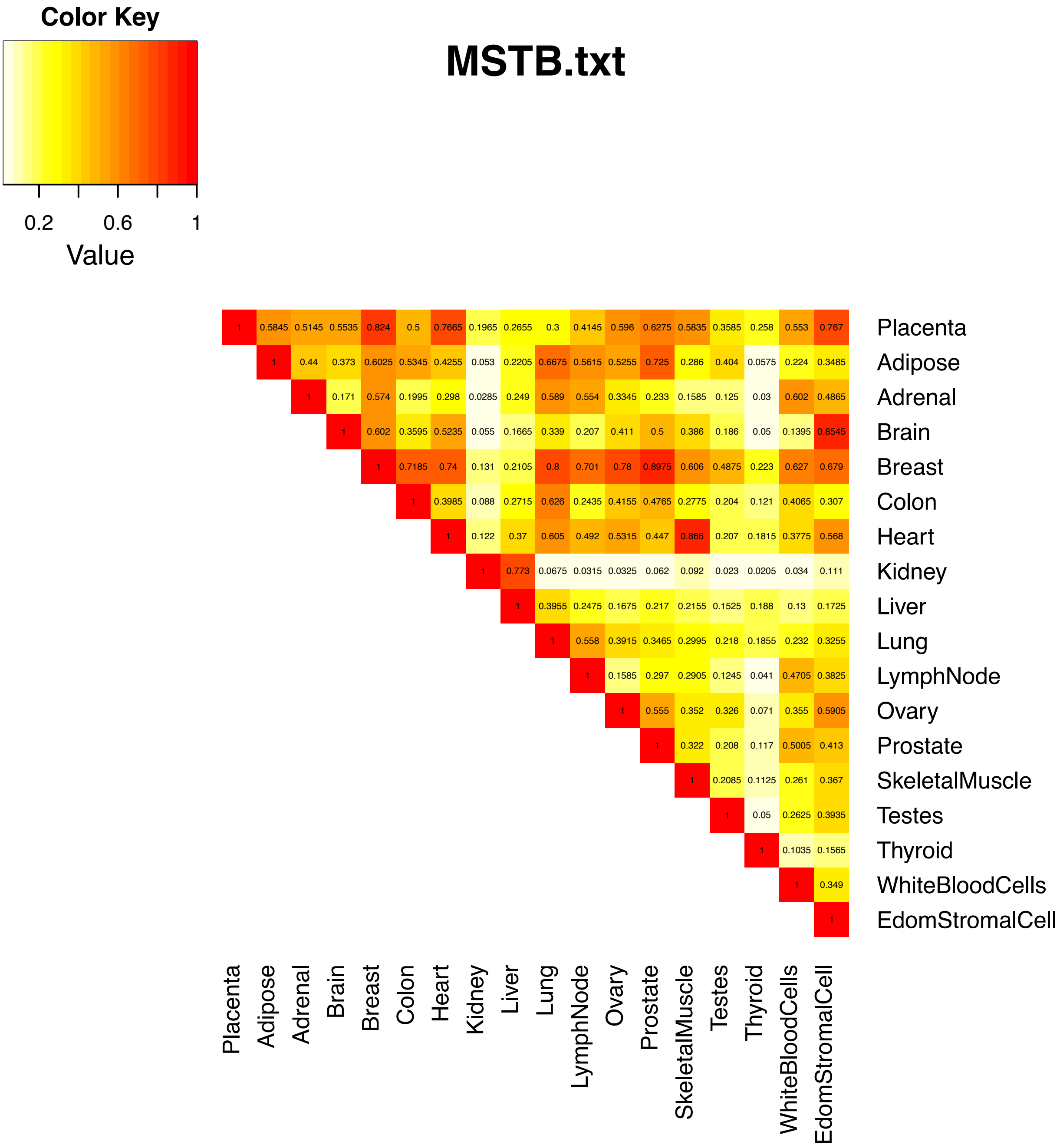
Color Key
0.2 0.6 1
Value
MSTB.txt
Placenta 1 0.5845 0.5145 0.5535 0.824 0.5 0.7665 0.1965 0.2655 0.3 0.4145 0.596 0.6275 0.5835 0.3585 0.258 0.553 0.767
Adipose 1 0.44 0.373 0.6025 0.5345 0.4255 0.053 0.2205 0.6675 0.5615 0.5255 0.725 0.286 0.404 0.0575 0.224 0.3485
Adrenal 1 0.171 0.574 0.1995 0.298 0.0285 0.249 0.589 0.554 0.3345 0.233 0.1585 0.125 0.03 0.602 0.4865
Brain 1 0.602 0.3595 0.5235 0.055 0.1665 0.339 0.207 0.411 0.5 0.386 0.186 0.05 0.1395 0.8545
Breast 1 0.7185 0.74 0.131 0.2105 0.8 0.701 0.78 0.8975 0.606 0.4875 0.223 0.627 0.679
Colon 1 0.3985 0.088 0.2715 0.626 0.2435 0.4155 0.4765 0.2775 0.204 0.121 0.4065 0.307
Heart 1 0.122 0.37 0.605 0.492 0.5315 0.447 0.866 0.207 0.1815 0.3775 0.568
Kidney 1 0.773 0.0675 0.0315 0.0325 0.062 0.092 0.023 0.0205 0.034 0.111
Liver 1 0.3955 0.2475 0.1675 0.217 0.2155 0.1525 0.188 0.13 0.1725
Lung 1 0.558 0.3915 0.3465 0.2995 0.218 0.1855 0.232 0.3255
LymphNode 1 0.1585 0.297 0.2905 0.1245 0.041 0.4705 0.3825
Ovary 1 0.555 0.352 0.326 0.071 0.355 0.5905
Prostate 1 0.322 0.208 0.117 0.5005 0.413
SkeletalMuscle 1 0.2085 0.1125 0.261 0.367
Testes 1 0.05 0.2625 0.3935
Thyroid 1 0.1035 0.1565
WhiteBloodCells 1 0.349
EdomStromalCell 1
Placenta Adipose Adrenal Brain Breast Colon Heart Kidney Liver Lung LymphNode Ovary Prostate SkeletalMuscle Testes Thyroid WhiteBloodCells EdomStromalCell

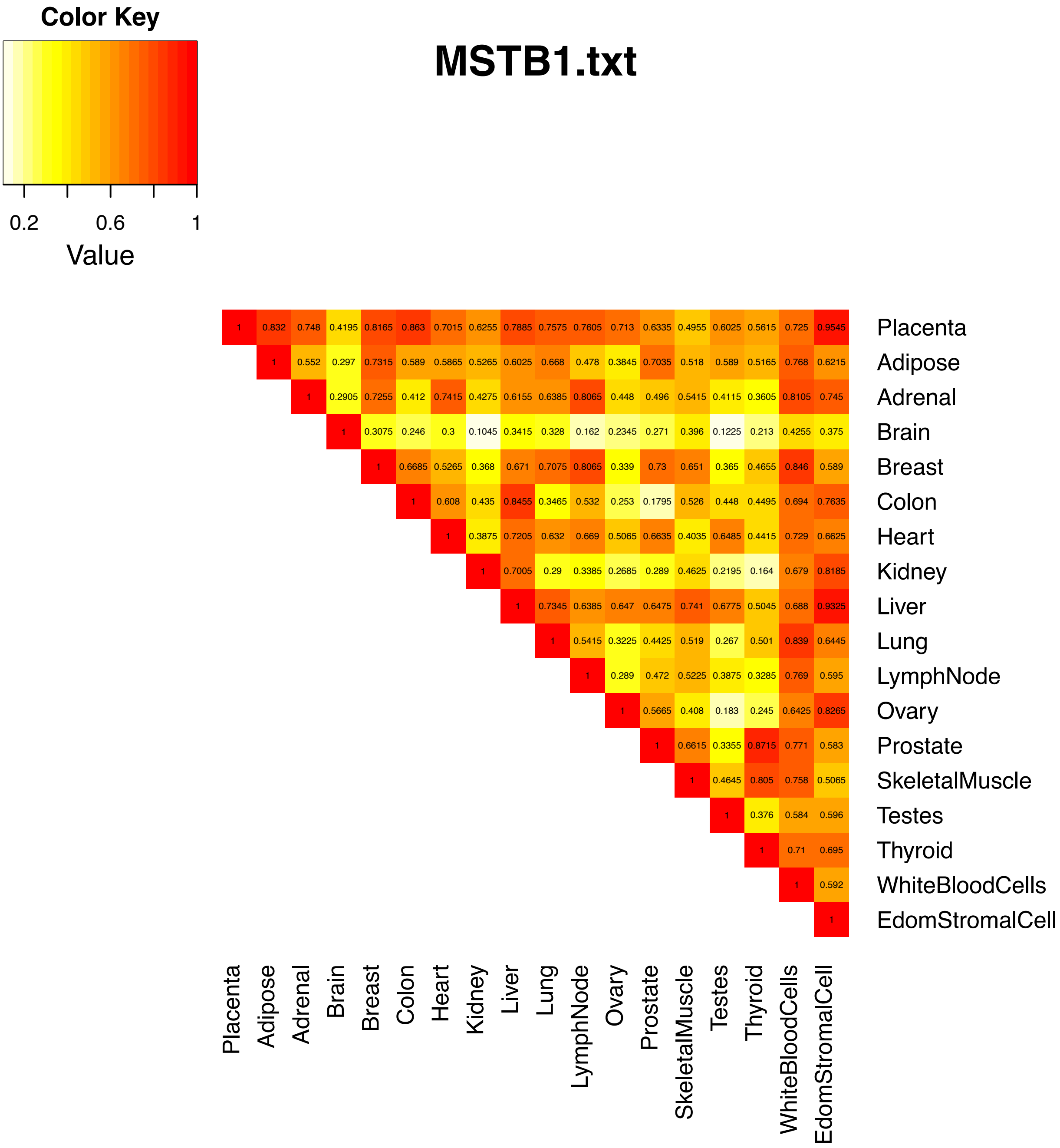

Color Key
0.2
0.6
1
Value
MSTB1.txt
Placenta 1 0.832 0.748 0.4195 0.8165 0.863 0.7015 0.6255 0.7885 0.7575 0.7605 0.713 0.6335 0.4955 0.6025 0.5615 0.725 0.9545
Adipose 1 0.552 0.297 0.7315 0.589 0.5865 0.5265 0.6025 0.668 0.478 0.3845 0.7035 0.518 0.589 0.5165 0.768 0.6215
Adrenal 1 0.2905 0.7255 0.412 0.7415 0.4275 0.6155 0.6385 0.8065 0.448 0.496 0.5415 0.4115 0.3605 0.8105 0.745
Brain 1 0.3075 0.246 0.3 0.1045 0.3415 0.328 0.162 0.2345 0.271 0.396 0.1225 0.213 0.4255 0.375
Breast 1 0.6685 0.5265 0.368 0.671 0.7075 0.8065 0.339 0.73 0.651 0.365 0.4655 0.846 0.589
Colon 1 0.608 0.435 0.8455 0.3465 0.532 0.253 0.1795 0.526 0.448 0.4495 0.694 0.7635
Heart 1 0.3875 0.7205 0.632 0.669 0.5065 0.6635 0.4035 0.6485 0.4415 0.729 0.6625
Kidney 1 0.7005 0.29 0.3385 0.2685 0.289 0.4625 0.2195 0.164 0.679 0.8185
Liver 1 0.7345 0.6385 0.647 0.6475 0.741 0.6775 0.5045 0.688 0.9325
Lung 1 0.5415 0.3225 0.4425 0.519 0.267 0.501 0.839 0.6445
LymphNode 1 0.289 0.472 0.5225 0.3875 0.3285 0.769 0.595
Ovary 1 0.5665 0.408 0.183 0.245 0.6425 0.8265
Prostate 1 0.6615 0.3355 0.8715 0.771 0.583
SkeletalMuscle 1 0.4645 0.805 0.758 0.5065
Testes 1 0.376 0.584 0.596
Thyroid 1 0.71 0.695
WhiteBloodCells 1 0.592
EdomStromalCell 1
Placenta
Adipose
Adrenal
Brain
Breast
Colon
Heart
Kidney
Liver
Lung
LymphNode
Ovary
Prostate
SkeletalMuscle
Testes
Thyroid
WhiteBloodCells
EdomStromalCell

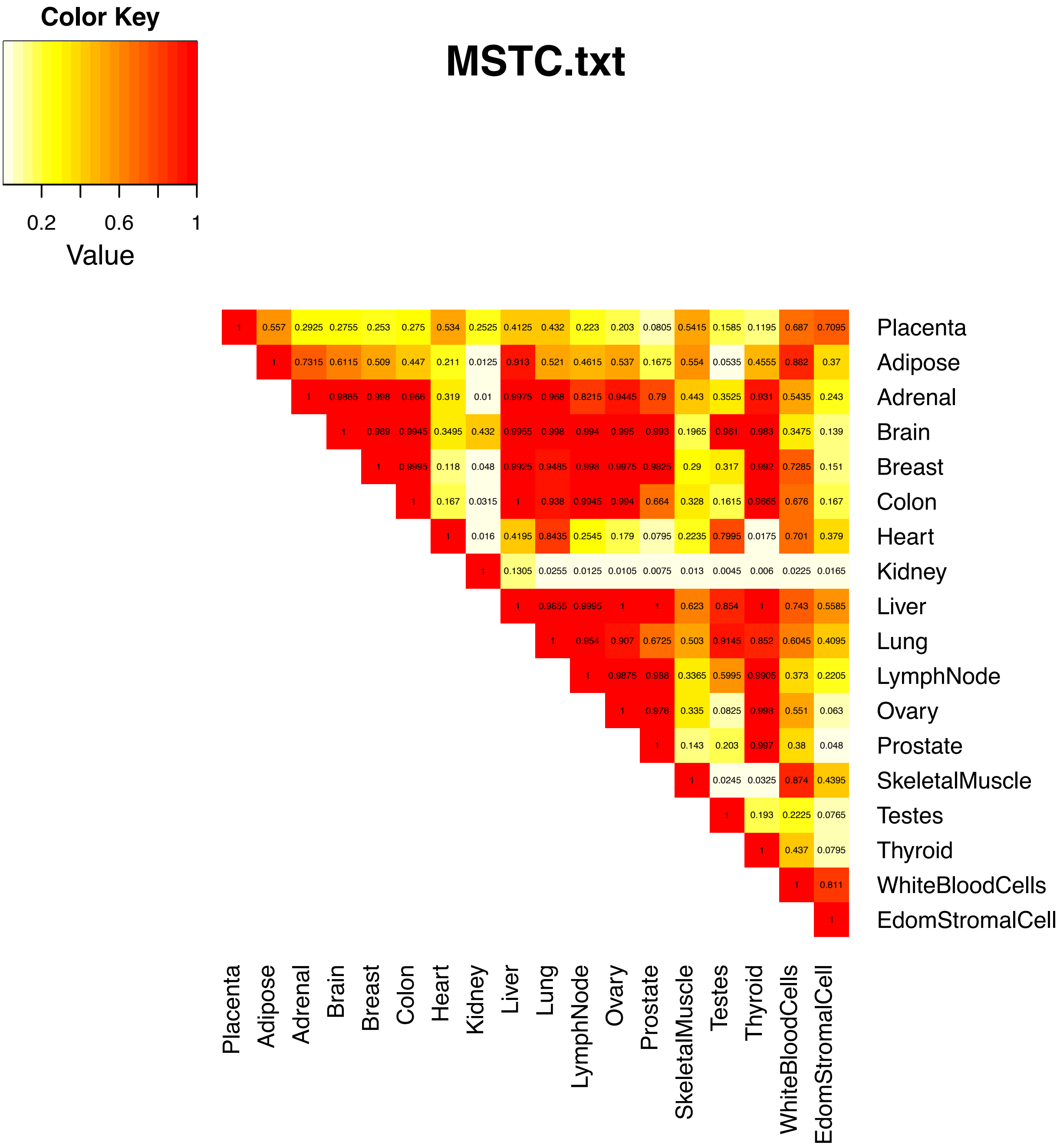
Color Key
0.2 0.6 1
Value
MSTC.txt
Placenta: 1 0.557 0.2925 0.2755 0.253 0.275 0.534 0.2525 0.4125 0.432 0.223 0.203 0.0805 0.5415 0.1585 0.1195 0.687 0.7095
Adipose: 1 0.7315 0.6115 0.509 0.447 0.211 0.0125 0.913 0.521 0.4615 0.537 0.1675 0.554 0.0535 0.4555 0.882 0.37
Adrenal: 1 0.9885 0.998 0.966 0.319 0.01 0.9975 0.968 0.8215 0.9445 0.79 0.443 0.3525 0.931 0.5435 0.243
Brain: 1 0.989 0.9945 0.3495 0.432 0.9955 0.998 0.994 0.995 0.993 0.1965 0.961 0.983 0.3475 0.139
Breast: 1 0.9995 0.118 0.048 0.9925 0.9485 0.998 0.9975 0.9825 0.29 0.317 0.992 0.7285 0.151
Colon: 1 0.167 0.0315 1 0.938 0.9945 0.994 0.664 0.328 0.1615 0.9665 0.676 0.167
Heart: 1 0.016 0.4195 0.8435 0.2545 0.179 0.0795 0.2235 0.7995 0.0175 0.701 0.379
Kidney: 1 0.1305 0.0255 0.0125 0.0105 0.0075 0.013 0.0045 0.006 0.0225 0.0165
Liver: 1 0.9855 0.9995 1 1 0.623 0.854 1 0.743 0.5585
Lung: 1 0.954 0.907 0.6725 0.503 0.9145 0.852 0.6045 0.4095
LymphNode: 1 0.9875 0.988 0.3365 0.5995 0.9905 0.373 0.2205
Ovary: 1 0.976 0.335 0.0825 0.998 0.551 0.063
Prostate: 1 0.143 0.203 0.997 0.38 0.048
SkeletalMuscle: 1 0.0245 0.0325 0.874 0.4395
Testes: 1 0.193 0.2225 0.0765
Thyroid: 1 0.437 0.0795
WhiteBloodCells: 1 0.811
EdomStromalCell: 1
Placenta Adipose Adrenal Brain Breast Colon Heart Kidney Liver Lung LymphNode Ovary Prostate SkeletalMuscle Testes Thyroid WhiteBloodCells EdomStromalCell

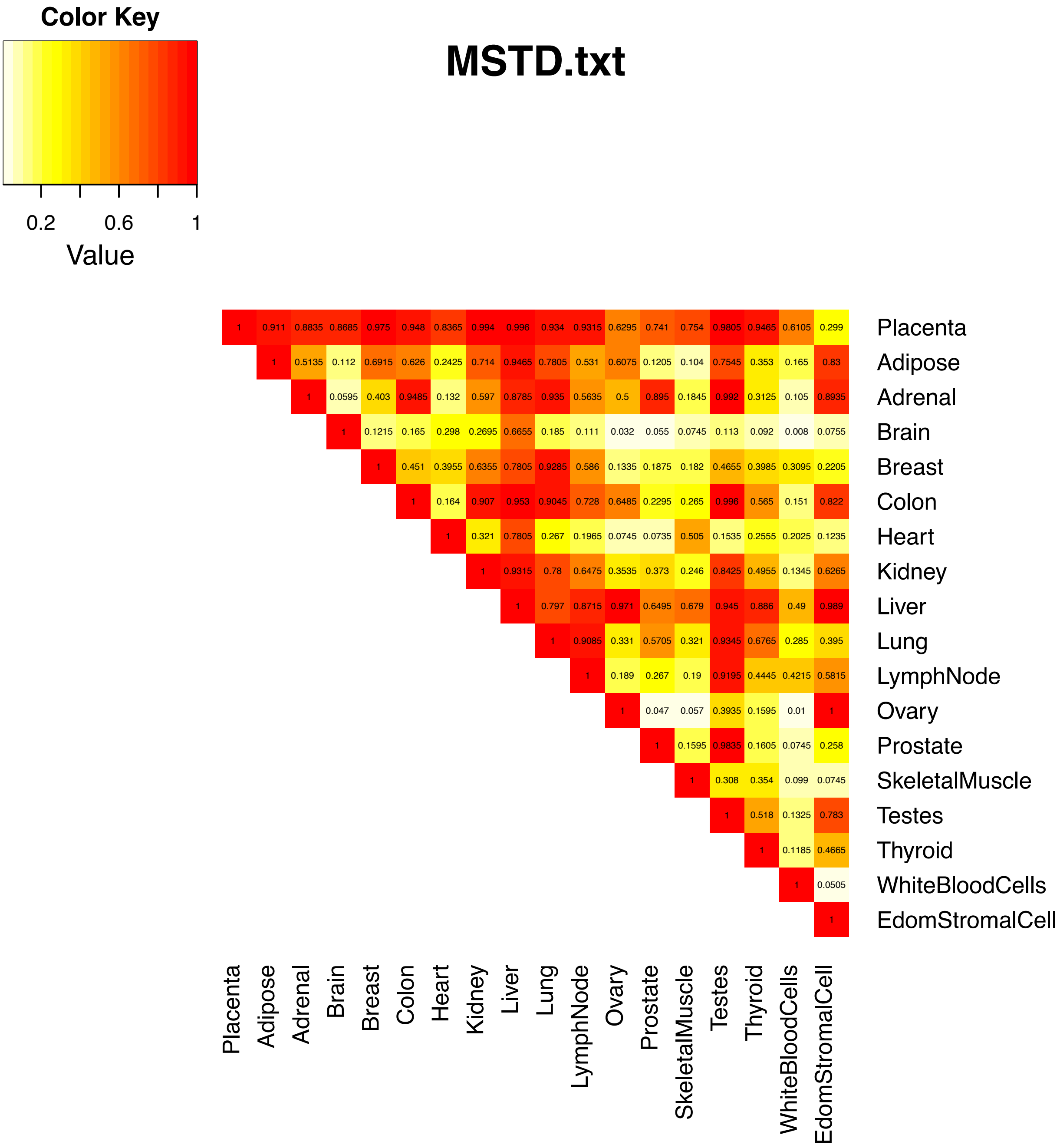
Color Key
0.2
0.6
1
Value
MSTD.txt
Placenta
Adipose
Adrenal
Brain
Breast
Colon
Heart
Kidney
Liver
Lung
LymphNode
Ovary
Prostate
SkeletalMuscle
Testes
Thyroid
WhiteBloodCells
EdomStromalCell
1 0.911 0.8835 0.8685 0.975 0.948 0.8365 0.994 0.996 0.934 0.9315 0.6295 0.741 0.754 0.9805 0.9465 0.6105 0.299
1 0.5135 0.112 0.6915 0.626 0.2425 0.714 0.9465 0.7805 0.531 0.6075 0.1205 0.104 0.7545 0.353 0.165 0.83
1 0.0595 0.403 0.9485 0.132 0.597 0.8785 0.935 0.5635 0.5 0.895 0.1845 0.992 0.3125 0.105 0.8935
1 0.1215 0.165 0.298 0.2695 0.6655 0.185 0.111 0.032 0.055 0.0745 0.113 0.092 0.008 0.0755
1 0.451 0.3955 0.6355 0.7805 0.9285 0.586 0.1335 0.1875 0.182 0.4655 0.3985 0.3095 0.2205
1 0.164 0.907 0.953 0.9045 0.728 0.6485 0.2295 0.265 0.996 0.565 0.151 0.822
1 0.321 0.7805 0.267 0.1965 0.0745 0.0735 0.505 0.1535 0.2555 0.2025 0.1235
1 0.9315 0.78 0.6475 0.3535 0.373 0.246 0.8425 0.4955 0.1345 0.6265
1 0.797 0.8715 0.971 0.6495 0.679 0.945 0.886 0.49 0.989
1 0.9085 0.331 0.5705 0.321 0.9345 0.6765 0.285 0.395
1 0.189 0.267 0.19 0.9195 0.4445 0.4215 0.5815
1 0.047 0.057 0.3935 0.1595 0.01 1
1 0.1595 0.9835 0.1605 0.0745 0.258
1 0.308 0.354 0.099 0.0745
1 0.518 0.1325 0.783
1 0.1185 0.4665
1 0.0505
1

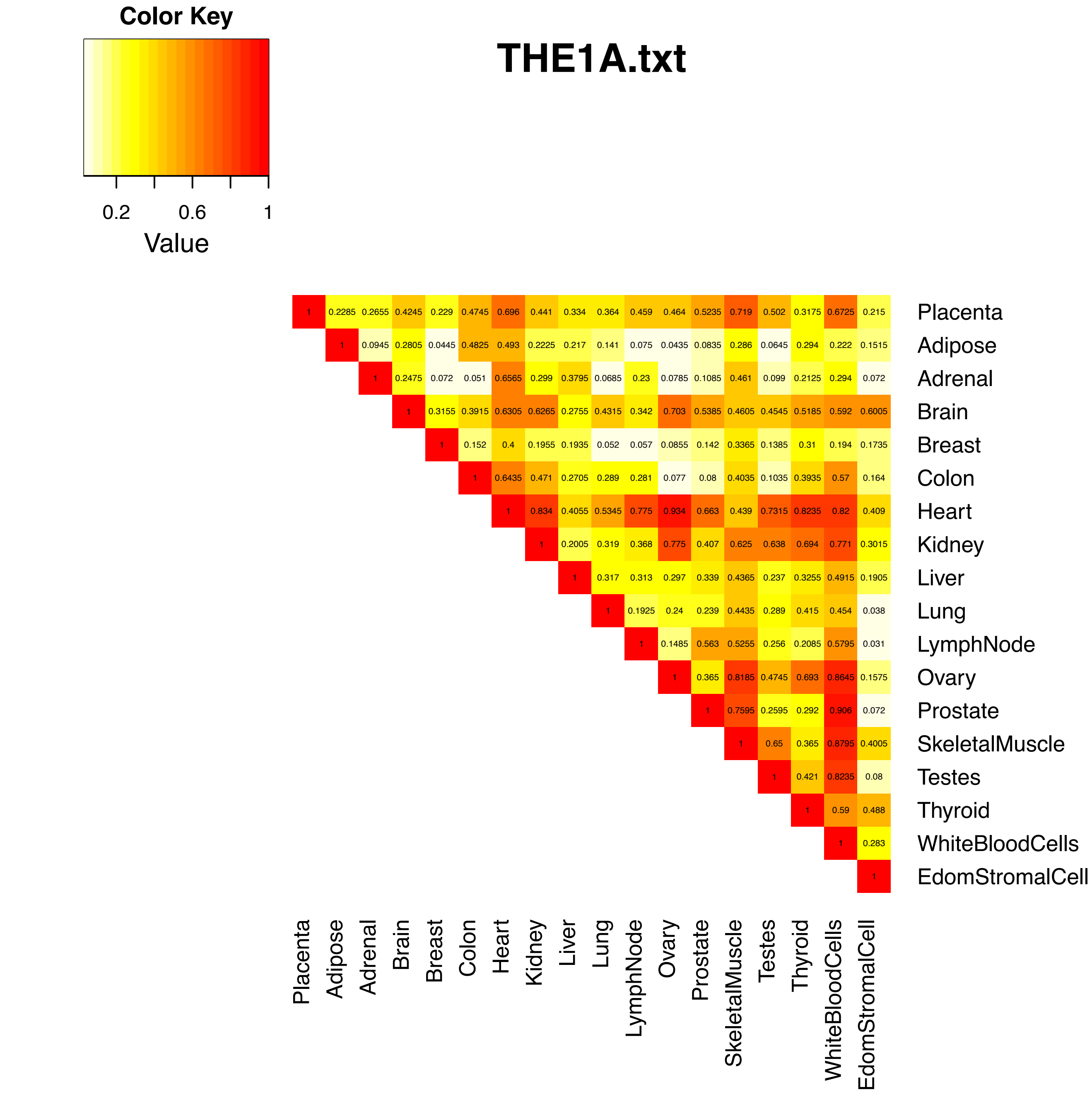

Color Key
0.2
0.6
1
Value
THE1A.txt
Placenta 1 0.2285 0.2655 0.4245 0.229 0.4745 0.696 0.441 0.334 0.364 0.459 0.464 0.5235 0.719 0.502 0.3175 0.6725 0.215
Adipose 1 0.0945 0.2805 0.0445 0.4825 0.493 0.2225 0.217 0.141 0.075 0.0435 0.0835 0.286 0.0645 0.294 0.222 0.1515
Adrenal 1 0.2475 0.072 0.051 0.6565 0.299 0.3795 0.0685 0.23 0.0785 0.1085 0.461 0.099 0.2125 0.294 0.072
Brain 1 0.3155 0.3915 0.6305 0.6265 0.2755 0.4315 0.342 0.703 0.5385 0.4605 0.4545 0.5185 0.592 0.6005
Breast 1 0.152 0.4 0.1955 0.1935 0.052 0.057 0.0855 0.142 0.3365 0.1385 0.31 0.194 0.1735
Colon 1 0.6435 0.471 0.2705 0.289 0.281 0.077 0.08 0.4035 0.1035 0.3935 0.57 0.164
Heart 1 0.834 0.4055 0.5345 0.775 0.934 0.663 0.439 0.7315 0.8235 0.82 0.409
Kidney 1 0.2005 0.319 0.368 0.775 0.407 0.625 0.638 0.694 0.771 0.3015
Liver 1 0.317 0.313 0.297 0.339 0.4365 0.237 0.3255 0.4915 0.1905
Lung 1 0.1925 0.24 0.239 0.4435 0.289 0.415 0.454 0.038
LymphNode 1 0.1485 0.563 0.5255 0.256 0.2085 0.5795 0.031
Ovary 1 0.365 0.8185 0.4745 0.693 0.8645 0.1575
Prostate 1 0.7595 0.2595 0.292 0.906 0.072
SkeletalMuscle 1 0.65 0.365 0.8795 0.4005
Testes 1 0.421 0.8235 0.08
Thyroid 1 0.59 0.488
WhiteBloodCells 1 0.283
EdomStromalCell 1
Placenta
Adipose
Adrenal
Brain
Breast
Colon
Heart
Kidney
Liver
Lung
LymphNode
Ovary
Prostate
SkeletalMuscle
Testes
Thyroid
WhiteBloodCells
EdomStromalCell

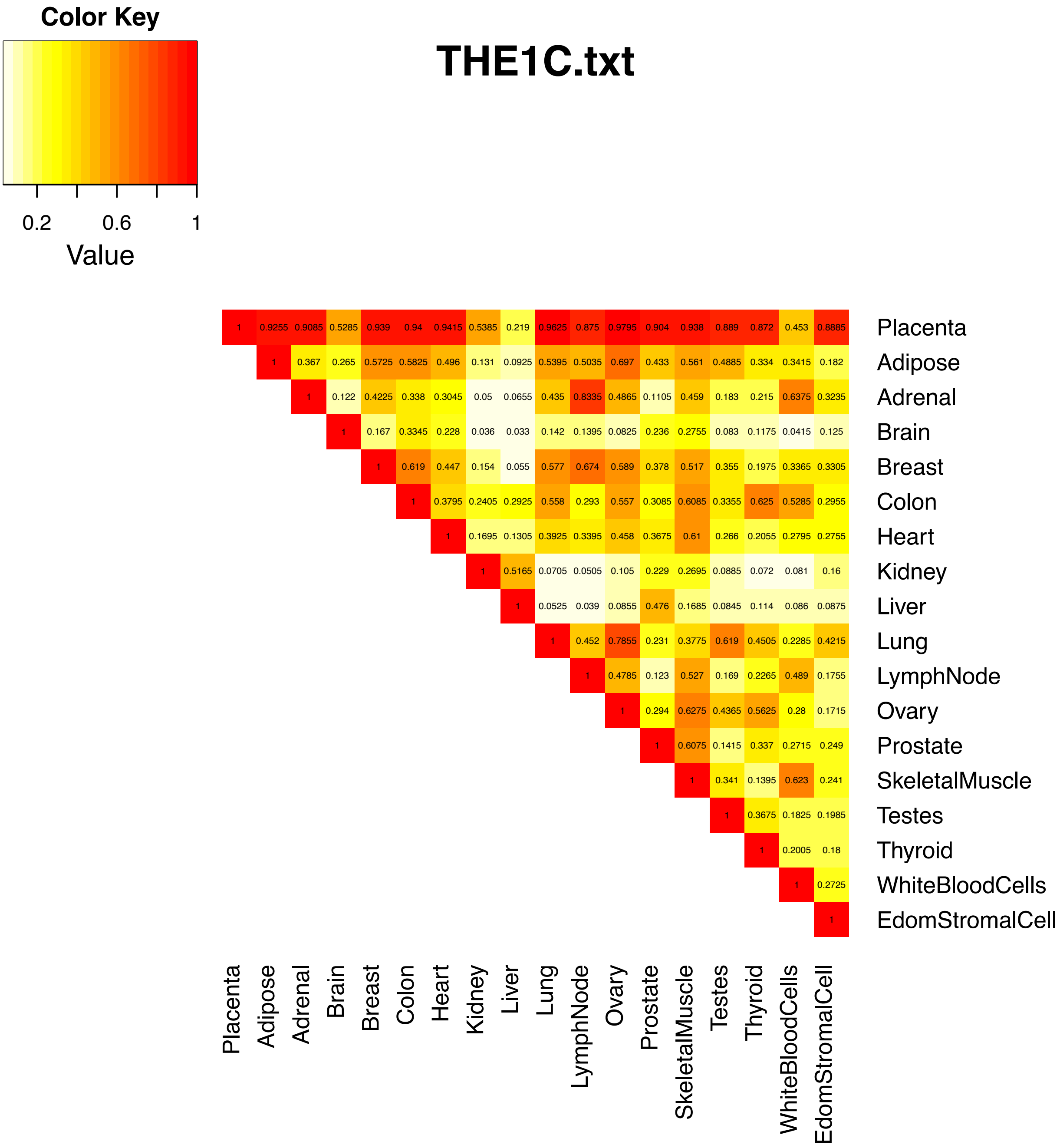
Color Key
0.2
0.6
1
Value
THE1C.txt
Placenta 1 0.9255 0.9085 0.5285 0.939 0.94 0.9415 0.5385 0.219 0.9625 0.875 0.9795 0.904 0.938 0.889 0.872 0.453 0.8885
Adipose 1 0.367 0.265 0.5725 0.5825 0.496 0.131 0.0925 0.5395 0.5035 0.697 0.433 0.561 0.4885 0.334 0.3415 0.182
Adrenal 1 0.122 0.4225 0.338 0.3045 0.05 0.0655 0.435 0.8335 0.4865 0.1105 0.459 0.183 0.215 0.6375 0.3235
Brain 1 0.167 0.3345 0.228 0.036 0.033 0.142 0.1395 0.0825 0.236 0.2755 0.083 0.1175 0.0415 0.125
Breast 1 0.619 0.447 0.154 0.055 0.577 0.674 0.589 0.378 0.517 0.355 0.1975 0.3365 0.3305
Colon 1 0.3795 0.2405 0.2925 0.558 0.293 0.557 0.3085 0.6085 0.3355 0.625 0.5285 0.2955
Heart 1 0.1695 0.1305 0.3925 0.3395 0.458 0.3675 0.61 0.266 0.2055 0.2795 0.2755
Kidney 1 0.5165 0.0705 0.0505 0.105 0.229 0.2695 0.0885 0.072 0.081 0.16
Liver 1 0.0525 0.039 0.0855 0.476 0.1685 0.0845 0.114 0.086 0.0875
Lung 1 0.452 0.7855 0.231 0.3775 0.619 0.4505 0.2285 0.4215
LymphNode 1 0.4785 0.123 0.527 0.169 0.2265 0.489 0.1755
Ovary 1 0.294 0.6275 0.4365 0.5625 0.28 0.1715
Prostate 1 0.6075 0.1415 0.337 0.2715 0.249
SkeletalMuscle 1 0.341 0.1395 0.623 0.241
Testes 1 0.3675 0.1825 0.1985
Thyroid 1 0.2005 0.18
WhiteBloodCells 1 0.2725
EdomStromalCell 1
Placenta Adipose Adrenal Brain Breast Colon Heart Kidney Liver Lung LymphNode Ovary Prostate SkeletalMuscle Testes Thyroid WhiteBloodCells EdomStromalCell

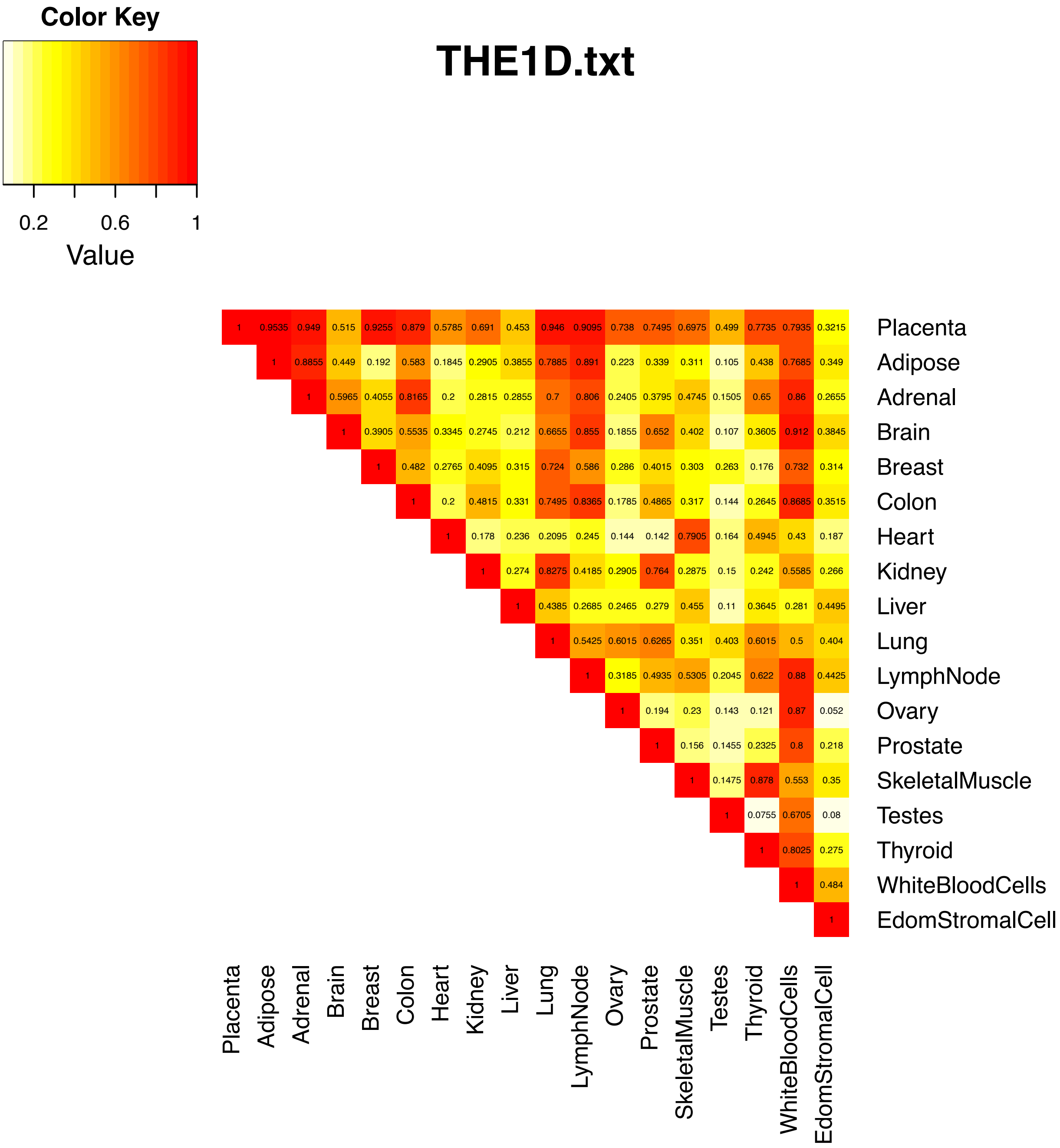
Color Key
0.2 0.6 1
Value
THE1D.txt
Placenta 1 0.9535 0.949 0.515 0.9255 0.879 0.5785 0.691 0.453 0.946 0.9095 0.738 0.7495 0.6975 0.499 0.7735 0.7935 0.3215
Adipose 1 0.8855 0.449 0.192 0.583 0.1845 0.2905 0.3855 0.7885 0.891 0.223 0.339 0.311 0.105 0.438 0.7685 0.349
Adrenal 1 0.5965 0.4055 0.8165 0.2 0.2815 0.2855 0.7 0.806 0.2405 0.3795 0.4745 0.1505 0.65 0.86 0.2655
Brain 1 0.3905 0.5535 0.3345 0.2745 0.212 0.6655 0.855 0.1855 0.652 0.402 0.107 0.3605 0.912 0.3845
Breast 1 0.482 0.2765 0.4095 0.315 0.724 0.586 0.286 0.4015 0.303 0.263 0.176 0.732 0.314
Colon 1 0.2 0.4815 0.331 0.7495 0.8365 0.1785 0.4865 0.317 0.144 0.2645 0.8685 0.3515
Heart 1 0.178 0.236 0.2095 0.245 0.144 0.142 0.7905 0.164 0.4945 0.43 0.187
Kidney 1 0.274 0.8275 0.4185 0.2905 0.764 0.2875 0.15 0.242 0.5585 0.266
Liver 1 0.4385 0.2685 0.2465 0.279 0.455 0.11 0.3645 0.281 0.4495
Lung 1 0.5425 0.6015 0.6265 0.351 0.403 0.6015 0.5 0.404
LymphNode 1 0.3185 0.4935 0.5305 0.2045 0.622 0.88 0.4425
Ovary 1 0.194 0.23 0.143 0.121 0.87 0.052
Prostate 1 0.156 0.1455 0.2325 0.8 0.218
SkeletalMuscle 1 0.1475 0.878 0.553 0.35
Testes 1 0.0755 0.6705 0.08
Thyroid 1 0.8025 0.275
WhiteBloodCells 1 0.484
EdomStromalCell 1
Placenta Adipose Adrenal Brain Breast Colon Heart Kidney Liver Lung LymphNode Ovary Prostate SkeletalMuscle Testes Thyroid WhiteBloodCells EdomStromalCell
