# Supplementary material for: Detecting Endogenous Retrovirus-Driven Tissue-Specific Gene Transcription
Source: Genome Biol Evol. 2015 Mar 11;7(4):1082–97. doi: 10.1093/gbe/evv049 (PMC4419796; doi:10.1093/gbe/evv049)
Supplement: Supplementary Data [file supp_evv049_S4_TEPlots_50kb.pdf]

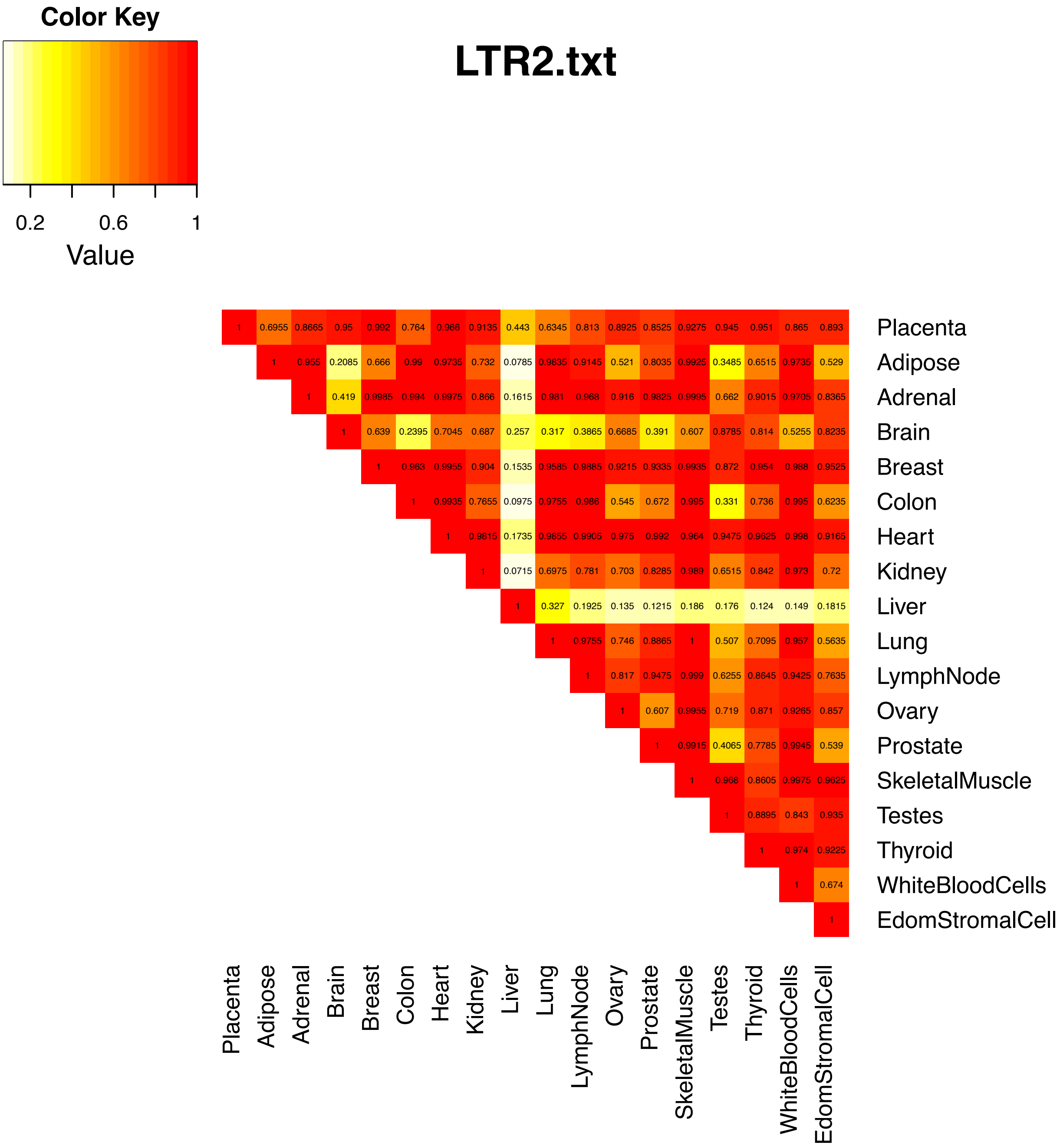
Color Key
0.2
0.6
1
Value
LTR2.txt
Placenta
Adipose
Adrenal
Brain
Breast
Colon
Heart
Kidney
Liver
Lung
LymphNode
Ovary
Prostate
SkeletalMuscle
Testes
Thyroid
WhiteBloodCells
EdomStromalCell
1 0.6955 0.8665 0.95 0.992 0.764 0.966 0.9135 0.443 0.6345 0.813 0.8925 0.8525 0.9275 0.945 0.951 0.865 0.893
1 0.955 0.2085 0.666 0.99 0.9735 0.732 0.0785 0.9835 0.9145 0.521 0.8035 0.9925 0.3485 0.6515 0.9735 0.529
1 0.419 0.9985 0.994 0.9975 0.866 0.1615 0.981 0.968 0.916 0.9825 0.9995 0.662 0.9015 0.9705 0.8365
1 0.639 0.2395 0.7045 0.687 0.257 0.317 0.3865 0.6685 0.391 0.607 0.8785 0.814 0.5255 0.8235
1 0.963 0.9955 0.904 0.1535 0.9585 0.9885 0.9215 0.9335 0.9935 0.872 0.954 0.988 0.9525
1 0.9935 0.7655 0.0975 0.9755 0.986 0.545 0.672 0.995 0.331 0.736 0.995 0.6235
1 0.9815 0.1735 0.9855 0.9905 0.975 0.992 0.964 0.9475 0.9625 0.998 0.9165
1 0.0715 0.6975 0.781 0.703 0.8285 0.989 0.6515 0.842 0.973 0.72
1 0.327 0.1925 0.135 0.1215 0.186 0.176 0.124 0.149 0.1815
1 0.9755 0.746 0.8865 1 0.507 0.7095 0.957 0.5635
1 0.817 0.9475 0.999 0.6255 0.8645 0.9425 0.7635
1 0.607 0.9955 0.719 0.871 0.9265 0.857
1 0.9915 0.4065 0.7785 0.9945 0.539
1 0.968 0.8605 0.9975 0.9625
1 0.8895 0.843 0.935
1 0.974 0.9225
1 0.674
1

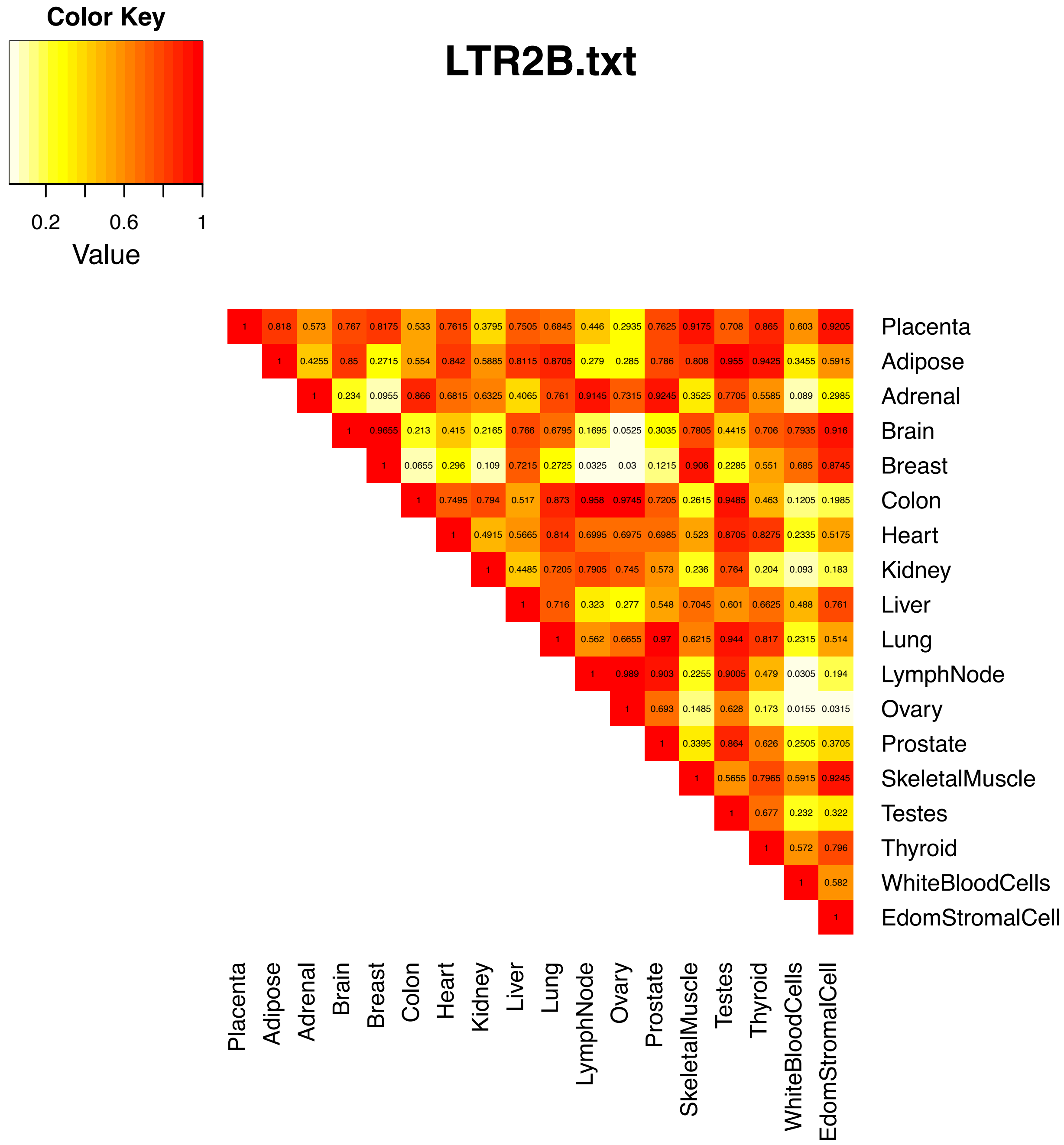
LTR2B.txt
Color Key
0.2 0.6 1
Value
Placenta
Adipose
Adrenal
Brain
Breast
Colon
Heart
Kidney
Liver
Lung
LymphNode
Ovary
Prostate
SkeletalMuscle
Testes
Thyroid
WhiteBloodCells
EdomStromalCell
1 0.818 0.573 0.767 0.8175 0.533 0.7615 0.3795 0.7505 0.6845 0.446 0.2935 0.7625 0.9175 0.708 0.865 0.603 0.9205
1 0.4255 0.85 0.2715 0.554 0.842 0.5885 0.8115 0.8705 0.279 0.285 0.786 0.808 0.955 0.9425 0.3455 0.5915
1 0.234 0.0955 0.866 0.6815 0.6325 0.4065 0.761 0.9145 0.7315 0.9245 0.3525 0.7705 0.5585 0.089 0.2985
1 0.9655 0.213 0.415 0.2165 0.766 0.6795 0.1695 0.0525 0.3035 0.7805 0.4415 0.706 0.7935 0.916
1 0.0655 0.296 0.109 0.7215 0.2725 0.0325 0.03 0.1215 0.906 0.2285 0.551 0.685 0.8745
1 0.7495 0.794 0.517 0.873 0.958 0.9745 0.7205 0.2615 0.9485 0.463 0.1205 0.1985
1 0.4915 0.5665 0.814 0.6995 0.6975 0.6985 0.523 0.8705 0.8275 0.2335 0.5175
1 0.4485 0.7205 0.7905 0.745 0.573 0.236 0.764 0.204 0.093 0.183
1 0.716 0.323 0.277 0.548 0.7045 0.601 0.6625 0.488 0.761
1 0.562 0.6655 0.97 0.6215 0.944 0.817 0.2315 0.514
1 0.989 0.903 0.2255 0.9005 0.479 0.0305 0.194
1 0.693 0.1485 0.628 0.173 0.0155 0.0315
1 0.3395 0.864 0.626 0.2505 0.3705
1 0.5655 0.7965 0.5915 0.9245
1 0.677 0.232 0.322
1 0.572 0.796
1 0.582
1

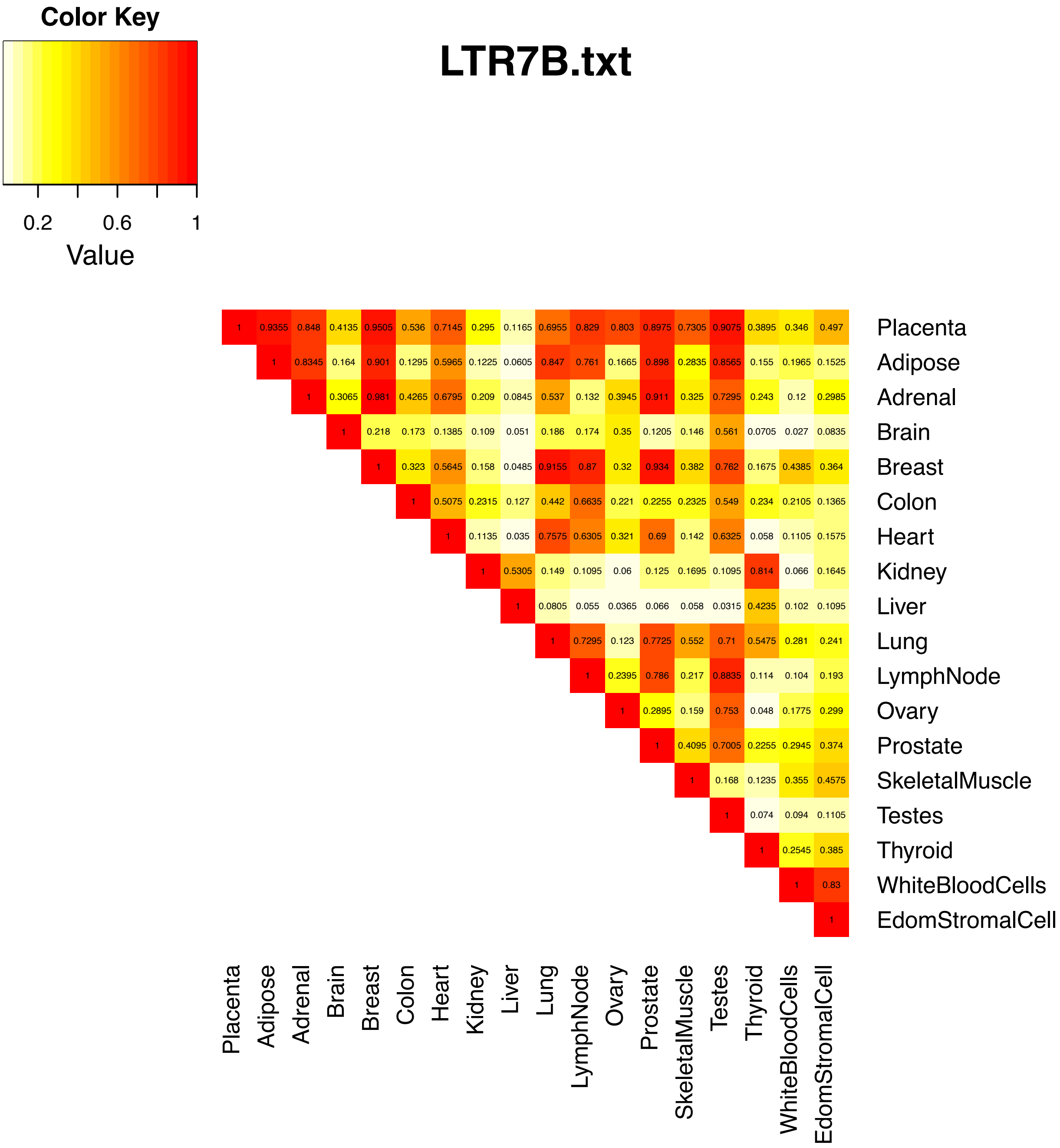
Color Key
0.2 0.6 1
Value
LTR7B.txt
Placenta 1 0.9355 0.848 0.4135 0.9505 0.536 0.7145 0.295 0.1165 0.6955 0.829 0.803 0.8975 0.7305 0.9075 0.3895 0.346 0.497
Adipose 1 0.8345 0.164 0.901 0.1295 0.5965 0.1225 0.0605 0.847 0.761 0.1665 0.898 0.2835 0.8565 0.155 0.1965 0.1525
Adrenal 1 0.3065 0.981 0.4265 0.6795 0.209 0.0845 0.537 0.132 0.3945 0.911 0.325 0.7295 0.243 0.12 0.2985
Brain 1 0.218 0.173 0.1385 0.109 0.051 0.186 0.174 0.35 0.1205 0.146 0.561 0.0705 0.027 0.0835
Breast 1 0.323 0.5645 0.158 0.0485 0.9155 0.87 0.32 0.934 0.382 0.762 0.1675 0.4385 0.364
Colon 1 0.5075 0.2315 0.127 0.442 0.6635 0.221 0.2255 0.2325 0.549 0.234 0.2105 0.1365
Heart 1 0.1135 0.035 0.7575 0.6305 0.321 0.69 0.142 0.6325 0.058 0.1105 0.1575
Kidney 1 0.5305 0.149 0.1095 0.06 0.125 0.1695 0.1095 0.814 0.066 0.1645
Liver 1 0.0805 0.055 0.0365 0.066 0.058 0.0315 0.4235 0.102 0.1095
Lung 1 0.7295 0.123 0.7725 0.552 0.71 0.5475 0.281 0.241
LymphNode 1 0.2395 0.786 0.217 0.8835 0.114 0.104 0.193
Ovary 1 0.2895 0.159 0.753 0.048 0.1775 0.299
Prostate 1 0.4095 0.7005 0.2255 0.2945 0.374
SkeletalMuscle 1 0.168 0.1235 0.355 0.4575
Testes 1 0.074 0.094 0.1105
Thyroid 1 0.2545 0.385
WhiteBloodCells 1 0.83
EdomStromalCell 1
Placenta Adipose Adrenal Brain Breast Colon Heart Kidney Liver Lung LymphNode Ovary Prostate SkeletalMuscle Testes Thyroid WhiteBloodCells EdomStromalCell

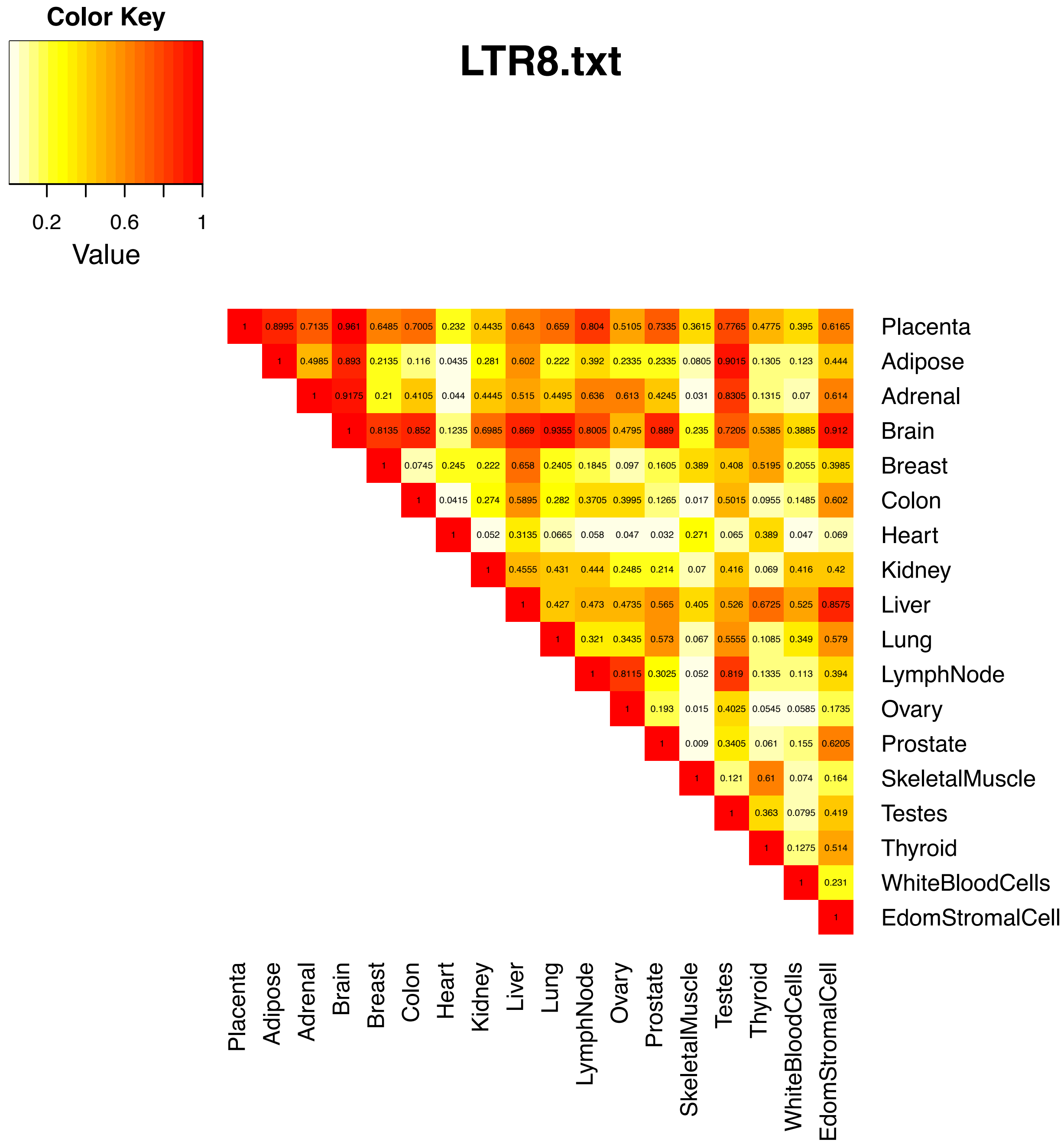
Color Key
0.2 0.6 1
Value
LTR8.txt
Placenta: 1 0.8995 0.7135 0.961 0.6485 0.7005 0.232 0.4435 0.643 0.659 0.804 0.5105 0.7335 0.3615 0.7765 0.4775 0.395 0.6165
Adipose: 1 0.4985 0.893 0.2135 0.116 0.0435 0.281 0.602 0.222 0.392 0.2335 0.2335 0.0805 0.9015 0.1305 0.123 0.444
Adrenal: 1 0.9175 0.21 0.4105 0.044 0.4445 0.515 0.4495 0.636 0.613 0.4245 0.031 0.8305 0.1315 0.07 0.614
Brain: 1 0.8135 0.852 0.1235 0.6985 0.869 0.9355 0.8005 0.4795 0.889 0.235 0.7205 0.5385 0.3885 0.912
Breast: 1 0.0745 0.245 0.222 0.658 0.2405 0.1845 0.097 0.1605 0.389 0.408 0.5195 0.2055 0.3985
Colon: 1 0.0415 0.274 0.5895 0.282 0.3705 0.3995 0.1265 0.017 0.5015 0.0955 0.1485 0.602
Heart: 1 0.052 0.3135 0.0665 0.058 0.047 0.032 0.271 0.065 0.389 0.047 0.069
Kidney: 1 0.4555 0.431 0.444 0.2485 0.214 0.07 0.416 0.069 0.416 0.42
Liver: 1 0.427 0.473 0.4735 0.565 0.405 0.526 0.6725 0.525 0.8575
Lung: 1 0.321 0.3435 0.573 0.067 0.5555 0.1085 0.349 0.579
LymphNode: 1 0.8115 0.3025 0.052 0.819 0.1335 0.113 0.394
Ovary: 1 0.193 0.015 0.4025 0.0545 0.0585 0.1735
Prostate: 1 0.009 0.3405 0.061 0.155 0.6205
SkeletalMuscle: 1 0.121 0.61 0.074 0.164
Testes: 1 0.363 0.0795 0.419
Thyroid: 1 0.1275 0.514
WhiteBloodCells: 1 0.231
EdomStromalCell: 1
Placenta Adipose Adrenal Brain Breast Colon Heart Kidney Liver Lung LymphNode Ovary Prostate SkeletalMuscle Testes Thyroid WhiteBloodCells EdomStromalCell

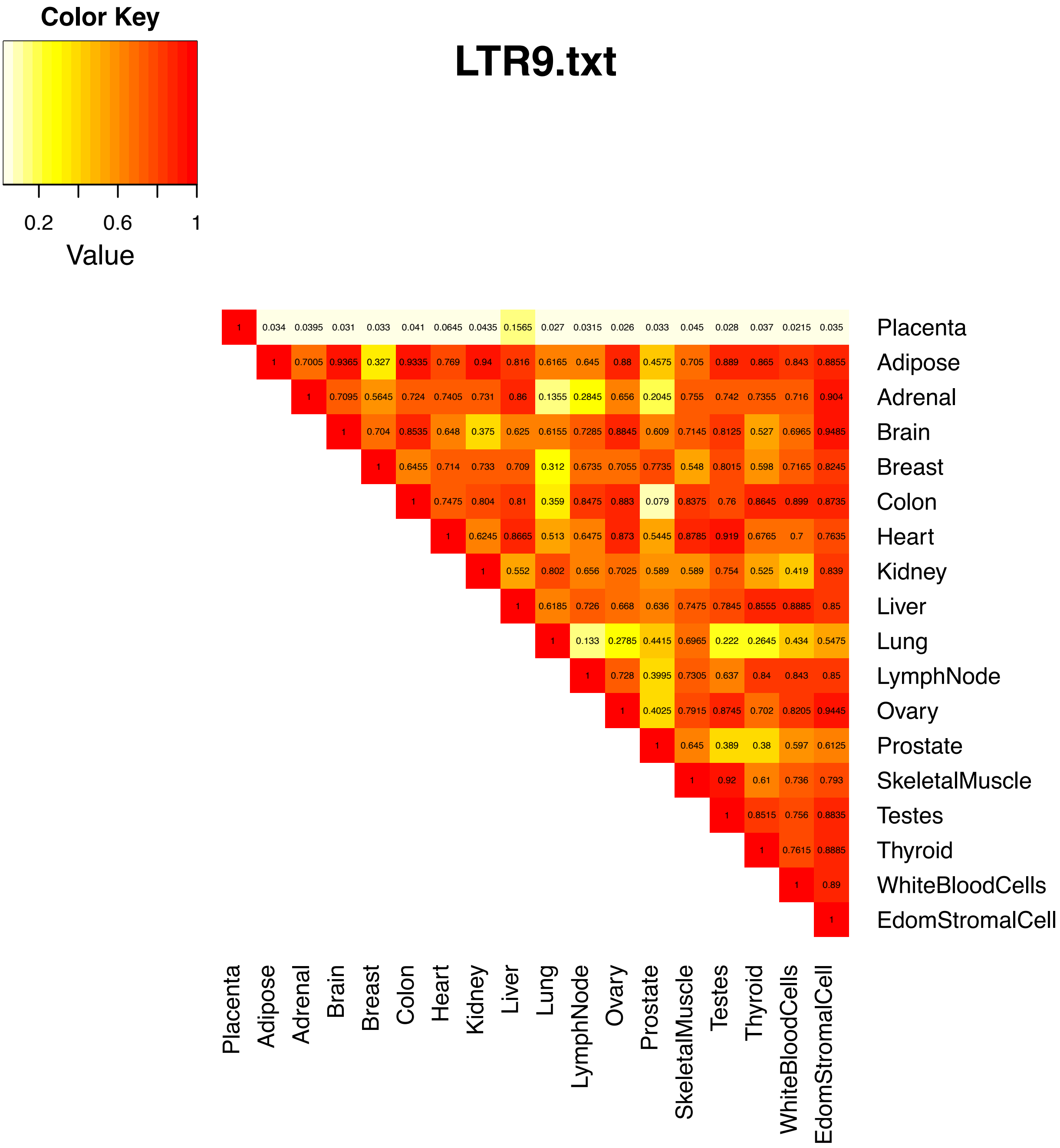
Color Key
0.2
0.6
1
Value
LTR9.txt
Placenta
Adipose
Adrenal
Brain
Breast
Colon
Heart
Kidney
Liver
Lung
LymphNode
Ovary
Prostate
SkeletalMuscle
Testes
Thyroid
WhiteBloodCells
EdomStromalCell
1 0.034 0.0395 0.031 0.033 0.041 0.0645 0.0435 0.1565 0.027 0.0315 0.026 0.033 0.045 0.028 0.037 0.0215 0.035
1 0.7005 0.9365 0.327 0.9335 0.769 0.94 0.816 0.6165 0.645 0.88 0.4575 0.705 0.889 0.865 0.843 0.8855
1 0.7095 0.5645 0.724 0.7405 0.731 0.86 0.1355 0.2845 0.656 0.2045 0.755 0.742 0.7355 0.716 0.904
1 0.704 0.8535 0.648 0.375 0.625 0.6155 0.7285 0.8845 0.609 0.7145 0.8125 0.527 0.6965 0.9485
1 0.6455 0.714 0.733 0.709 0.312 0.6735 0.7055 0.7735 0.548 0.8015 0.598 0.7165 0.8245
1 0.7475 0.804 0.81 0.359 0.8475 0.883 0.079 0.8375 0.76 0.8645 0.899 0.8735
1 0.6245 0.8665 0.513 0.6475 0.873 0.5445 0.8785 0.919 0.6765 0.7 0.7635
1 0.552 0.802 0.656 0.7025 0.589 0.589 0.754 0.525 0.419 0.839
1 0.6185 0.726 0.668 0.636 0.7475 0.7845 0.8555 0.8885 0.85
1 0.133 0.2785 0.4415 0.6965 0.222 0.2645 0.434 0.5475
1 0.728 0.3995 0.7305 0.637 0.84 0.843 0.85
1 0.4025 0.7915 0.8745 0.702 0.8205 0.9445
1 0.645 0.389 0.38 0.597 0.6125
1 0.92 0.61 0.736 0.793
1 0.8515 0.756 0.8835
1 0.7615 0.8885
1 0.89
1
Placenta
Adipose
Adrenal
Brain
Breast
Colon
Heart
Kidney
Liver
Lung
LymphNode
Ovary
Prostate
SkeletalMuscle
Testes
Thyroid
WhiteBloodCells
EdomStromalCell

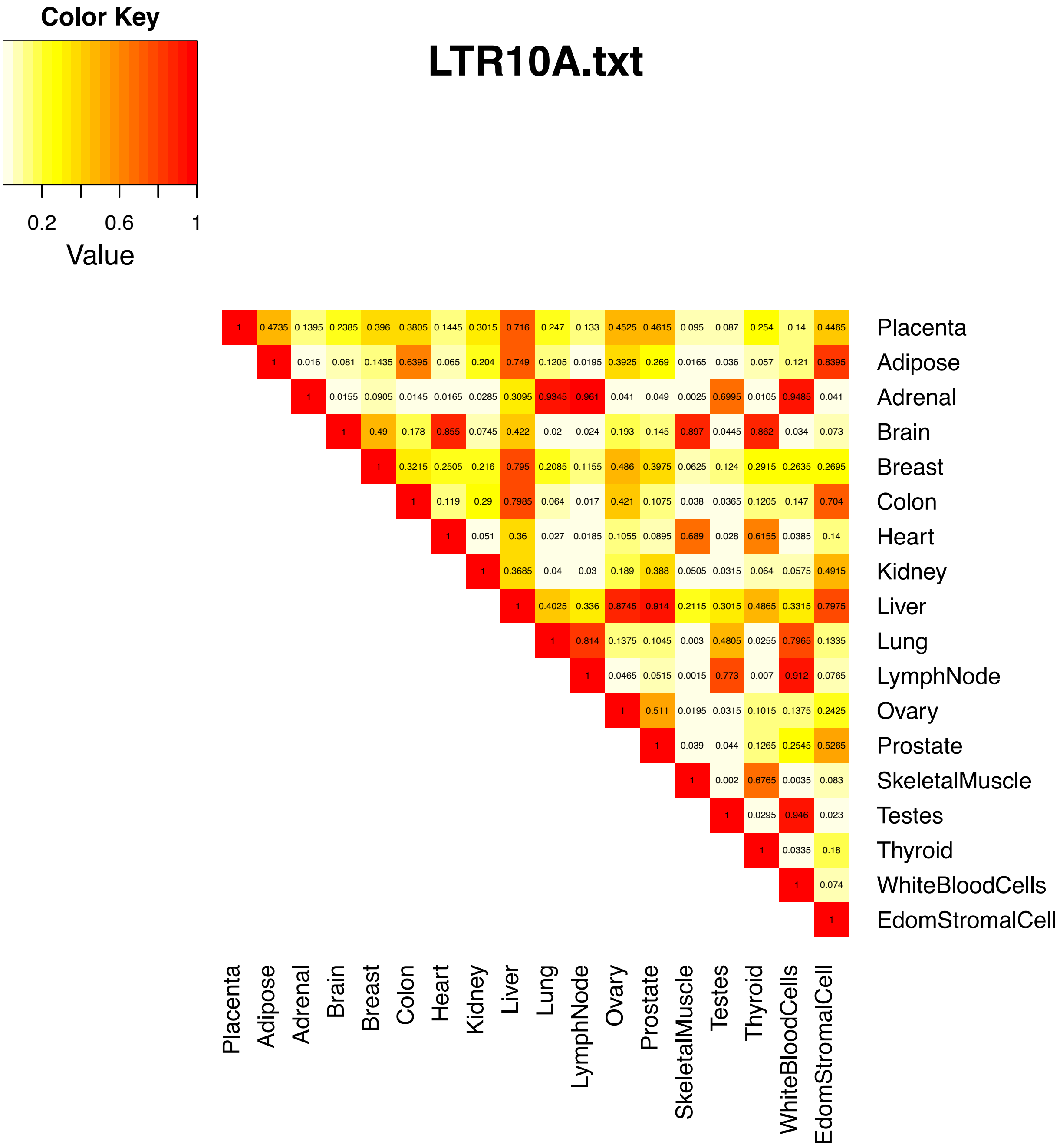
Color Key
0.2 0.6 1
Value
LTR10A.txt
Placenta
Adipose
Adrenal
Brain
Breast
Colon
Heart
Kidney
Liver
Lung
LymphNode
Ovary
Prostate
SkeletalMuscle
Testes
Thyroid
WhiteBloodCells
EdomStromalCell
1 0.4735 0.1395 0.2385 0.396 0.3805 0.1445 0.3015 0.716 0.247 0.133 0.4525 0.4615 0.095 0.087 0.254 0.14 0.4465
1 0.016 0.081 0.1435 0.6395 0.065 0.204 0.749 0.1205 0.0195 0.3925 0.269 0.0165 0.036 0.057 0.121 0.8395
1 0.0155 0.0905 0.0145 0.0165 0.0285 0.3095 0.9345 0.961 0.041 0.049 0.0025 0.6995 0.0105 0.9485 0.041
1 0.49 0.178 0.855 0.0745 0.422 0.02 0.024 0.193 0.145 0.897 0.0445 0.862 0.034 0.073
1 0.3215 0.2505 0.216 0.795 0.2085 0.1155 0.486 0.3975 0.0625 0.124 0.2915 0.2635 0.2695
1 0.119 0.29 0.7985 0.064 0.017 0.421 0.1075 0.038 0.0365 0.1205 0.147 0.704
1 0.051 0.36 0.027 0.0185 0.1055 0.0895 0.689 0.028 0.6155 0.0385 0.14
1 0.3685 0.04 0.03 0.189 0.388 0.0505 0.0315 0.064 0.0575 0.4915
1 0.4025 0.336 0.8745 0.914 0.2115 0.3015 0.4865 0.3315 0.7975
1 0.814 0.1375 0.1045 0.003 0.4805 0.0255 0.7965 0.1335
1 0.0465 0.0515 0.0015 0.773 0.007 0.912 0.0765
1 0.511 0.0195 0.0315 0.1015 0.1375 0.2425
1 0.039 0.044 0.1265 0.2545 0.5265
1 0.002 0.6765 0.0035 0.083
1 0.0295 0.946 0.023
1 0.0335 0.18
1 0.074
1

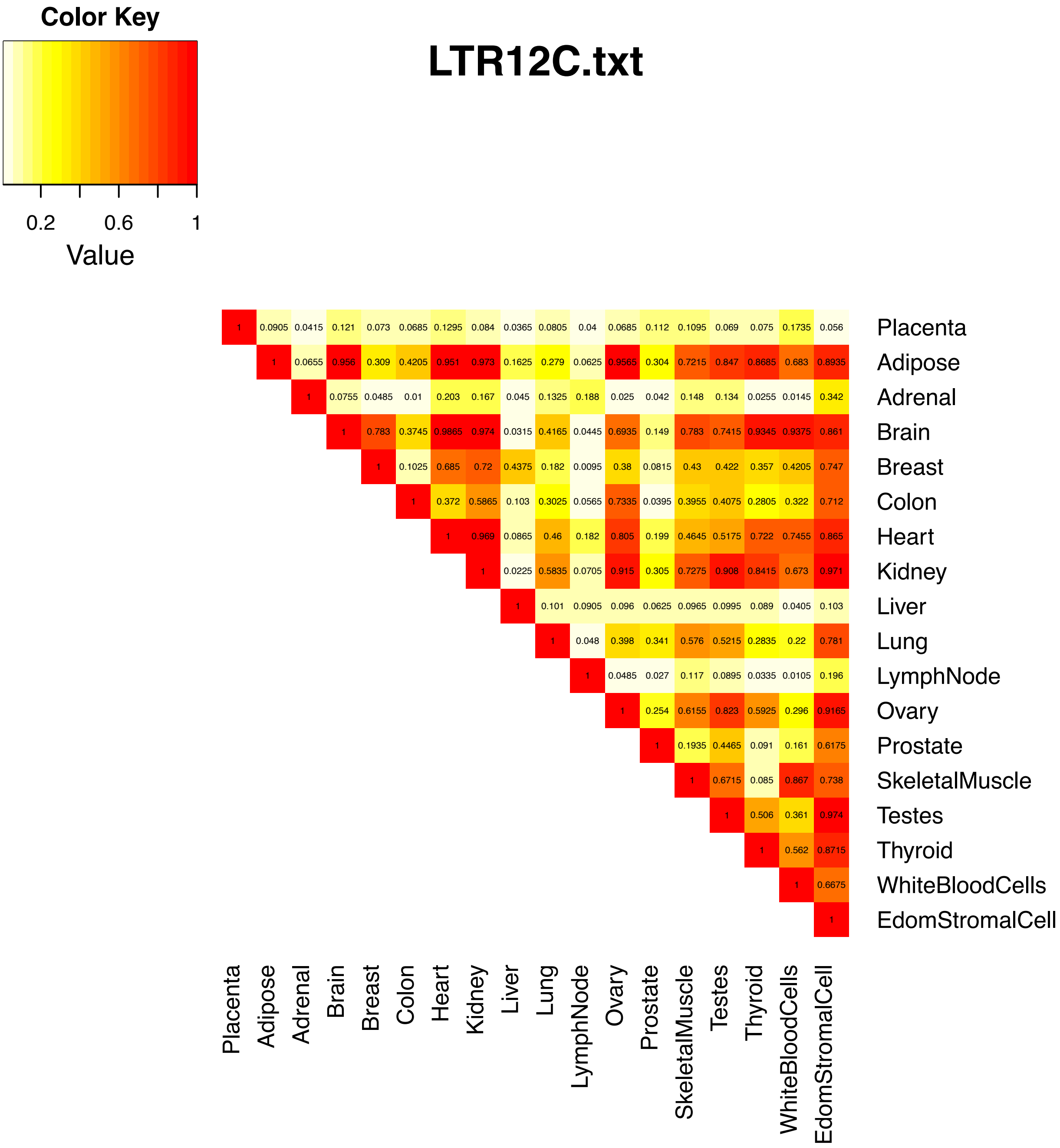
Color Key
0.2 0.6 1
Value
LTR12C.txt
Placenta: 1 0.0905 0.0415 0.121 0.073 0.0685 0.1295 0.084 0.0365 0.0805 0.04 0.0685 0.112 0.1095 0.069 0.075 0.1735 0.056
Adipose: 1 0.0655 0.956 0.309 0.4205 0.951 0.973 0.1625 0.279 0.0625 0.9565 0.304 0.7215 0.847 0.8685 0.683 0.8935
Adrenal: 1 0.0755 0.0485 0.01 0.203 0.167 0.045 0.1325 0.188 0.025 0.042 0.148 0.134 0.0255 0.0145 0.342
Brain: 1 0.783 0.3745 0.9865 0.974 0.0315 0.4165 0.0445 0.6935 0.149 0.783 0.7415 0.9345 0.9375 0.861
Breast: 1 0.1025 0.685 0.72 0.4375 0.182 0.0095 0.38 0.0815 0.43 0.422 0.357 0.4205 0.747
Colon: 1 0.372 0.5865 0.103 0.3025 0.0565 0.7335 0.0395 0.3955 0.4075 0.2805 0.322 0.712
Heart: 1 0.969 0.0865 0.46 0.182 0.805 0.199 0.4645 0.5175 0.722 0.7455 0.865
Kidney: 1 0.0225 0.5835 0.0705 0.915 0.305 0.7275 0.908 0.8415 0.673 0.971
Liver: 1 0.101 0.0905 0.096 0.0625 0.0965 0.0995 0.089 0.0405 0.103
Lung: 1 0.048 0.398 0.341 0.576 0.5215 0.2835 0.22 0.781
LymphNode: 1 0.0485 0.027 0.117 0.0895 0.0335 0.0105 0.196
Ovary: 1 0.254 0.6155 0.823 0.5925 0.296 0.9165
Prostate: 1 0.1935 0.4465 0.091 0.161 0.6175
SkeletalMuscle: 1 0.6715 0.085 0.867 0.738
Testes: 1 0.506 0.361 0.974
Thyroid: 1 0.562 0.8715
WhiteBloodCells: 1 0.6675
EdomStromalCell: 1
Placenta Adipose Adrenal Brain Breast Colon Heart Kidney Liver Lung LymphNode Ovary Prostate SkeletalMuscle Testes Thyroid WhiteBloodCells EdomStromalCell

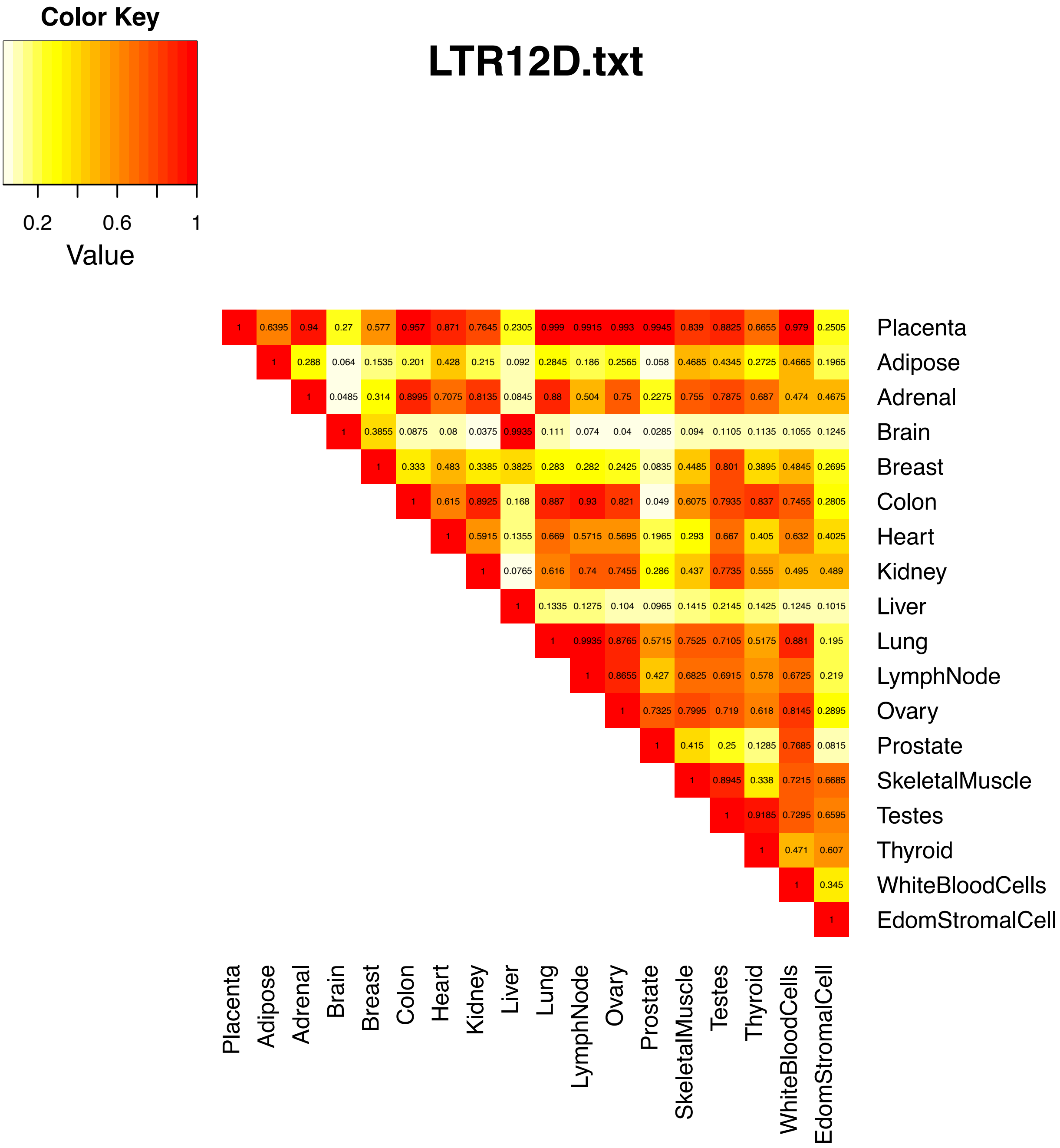
Color Key
0.2 0.6 1
Value
LTR12D.txt
Placenta: 1 0.6395 0.94 0.27 0.577 0.957 0.871 0.7645 0.2305 0.999 0.9915 0.993 0.9945 0.839 0.8825 0.6655 0.979 0.2505
Adipose: 1 0.288 0.064 0.1535 0.201 0.428 0.215 0.092 0.2845 0.186 0.2565 0.058 0.4685 0.4345 0.2725 0.4665 0.1965
Adrenal: 1 0.0485 0.314 0.8995 0.7075 0.8135 0.0845 0.88 0.504 0.75 0.2275 0.755 0.7875 0.687 0.474 0.4675
Brain: 1 0.3855 0.0875 0.08 0.0375 0.9935 0.111 0.074 0.04 0.0285 0.094 0.1105 0.1135 0.1055 0.1245
Breast: 1 0.333 0.483 0.3385 0.3825 0.283 0.282 0.2425 0.0835 0.4485 0.801 0.3895 0.4845 0.2695
Colon: 1 0.615 0.8925 0.168 0.887 0.93 0.821 0.049 0.6075 0.7935 0.837 0.7455 0.2805
Heart: 1 0.5915 0.1355 0.669 0.5715 0.5695 0.1965 0.293 0.667 0.405 0.632 0.4025
Kidney: 1 0.0765 0.616 0.74 0.7455 0.286 0.437 0.7735 0.555 0.495 0.489
Liver: 1 0.1335 0.1275 0.104 0.0965 0.1415 0.2145 0.1425 0.1245 0.1015
Lung: 1 0.9935 0.8765 0.5715 0.7525 0.7105 0.5175 0.881 0.195
LymphNode: 1 0.8655 0.427 0.6825 0.6915 0.578 0.6725 0.219
Ovary: 1 0.7325 0.7995 0.719 0.618 0.8145 0.2895
Prostate: 1 0.415 0.25 0.1285 0.7685 0.0815
SkeletalMuscle: 1 0.8945 0.338 0.7215 0.6685
Testes: 1 0.9185 0.7295 0.6595
Thyroid: 1 0.471 0.607
WhiteBloodCells: 1 0.345
EdomStromalCell: 1
Placenta Adipose Adrenal Brain Breast Colon Heart Kidney Liver Lung LymphNode Ovary Prostate SkeletalMuscle Testes Thyroid WhiteBloodCells EdomStromalCell

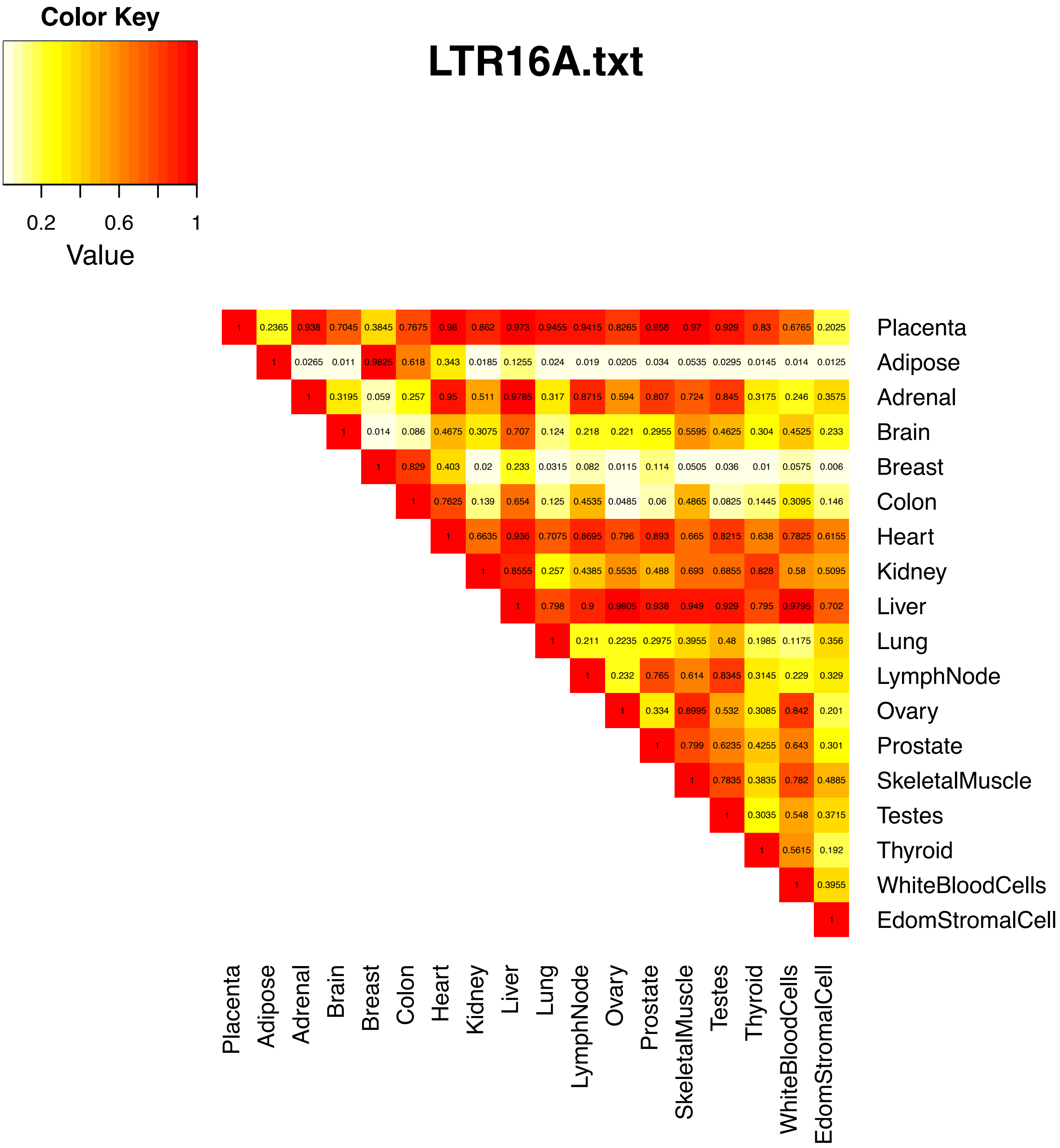

Color Key
0.2
0.6
1
Value
LTR16A.txt
Placenta: 1 0.2365 0.938 0.7045 0.3845 0.7675 0.98 0.862 0.973 0.9455 0.9415 0.8265 0.958 0.97 0.929 0.83 0.6765 0.2025
Adipose: 1 0.0265 0.011 0.9825 0.618 0.343 0.0185 0.1255 0.024 0.019 0.0205 0.034 0.0535 0.0295 0.0145 0.014 0.0125
Adrenal: 1 0.3195 0.059 0.257 0.95 0.511 0.9785 0.317 0.8715 0.594 0.807 0.724 0.845 0.3175 0.246 0.3575
Brain: 1 0.014 0.086 0.4675 0.3075 0.707 0.124 0.218 0.221 0.2955 0.5595 0.4625 0.304 0.4525 0.233
Breast: 1 0.829 0.403 0.02 0.233 0.0315 0.082 0.0115 0.114 0.0505 0.036 0.01 0.0575 0.006
Colon: 1 0.7625 0.139 0.654 0.125 0.4535 0.0485 0.06 0.4865 0.0825 0.1445 0.3095 0.146
Heart: 1 0.6635 0.936 0.7075 0.8695 0.796 0.893 0.665 0.8215 0.638 0.7825 0.6155
Kidney: 1 0.8555 0.257 0.4385 0.5535 0.488 0.693 0.6855 0.828 0.58 0.5095
Liver: 1 0.798 0.9 0.9805 0.938 0.949 0.929 0.795 0.9795 0.702
Lung: 1 0.211 0.2235 0.2975 0.3955 0.48 0.1985 0.1175 0.356
LymphNode: 1 0.232 0.765 0.614 0.8345 0.3145 0.229 0.329
Ovary: 1 0.334 0.8995 0.532 0.3085 0.842 0.201
Prostate: 1 0.799 0.6235 0.4255 0.643 0.301
SkeletalMuscle: 1 0.7835 0.3835 0.782 0.4885
Testes: 1 0.3035 0.548 0.3715
Thyroid: 1 0.5615 0.192
WhiteBloodCells: 1 0.3955
EdomStromalCell: 1
Placenta
Adipose
Adrenal
Brain
Breast
Colon
Heart
Kidney
Liver
Lung
LymphNode
Ovary
Prostate
SkeletalMuscle
Testes
Thyroid
WhiteBloodCells
EdomStromalCell

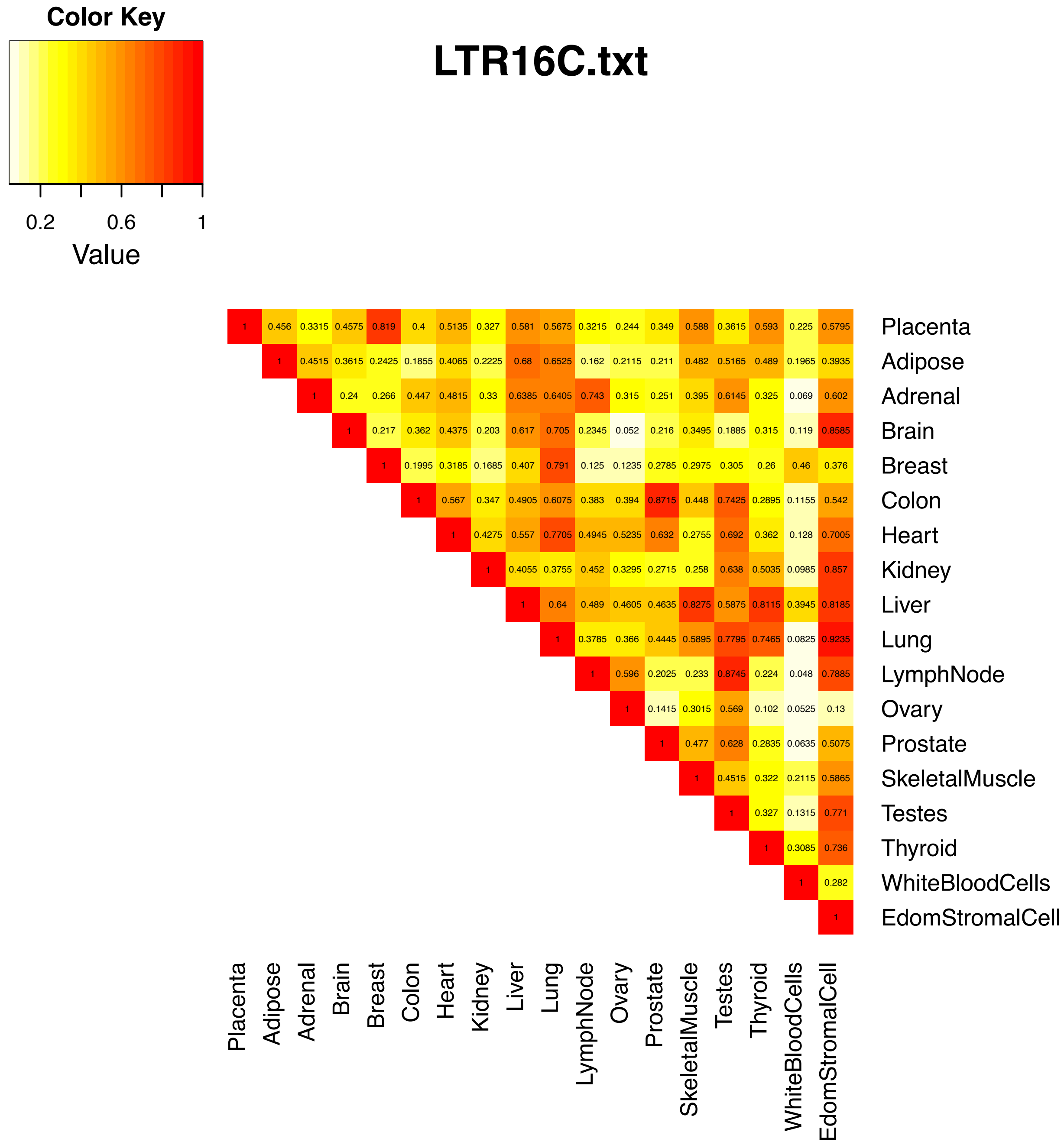
Color Key
0.2
0.6
1
Value
LTR16C.txt
Placenta
Adipose
Adrenal
Brain
Breast
Colon
Heart
Kidney
Liver
Lung
LymphNode
Ovary
Prostate
SkeletalMuscle
Testes
Thyroid
WhiteBloodCells
EdomStromalCell
1 0.456 0.3315 0.4575 0.819 0.4 0.5135 0.327 0.581 0.5675 0.3215 0.244 0.349 0.588 0.3615 0.593 0.225 0.5795
1 0.4515 0.3615 0.2425 0.1855 0.4065 0.2225 0.68 0.6525 0.162 0.2115 0.211 0.482 0.5165 0.489 0.1965 0.3935
1 0.24 0.266 0.447 0.4815 0.33 0.6385 0.6405 0.743 0.315 0.251 0.395 0.6145 0.325 0.069 0.602
1 0.217 0.362 0.4375 0.203 0.617 0.705 0.2345 0.052 0.216 0.3495 0.1885 0.315 0.119 0.8585
1 0.1995 0.3185 0.1685 0.407 0.791 0.125 0.1235 0.2785 0.2975 0.305 0.26 0.46 0.376
1 0.567 0.347 0.4905 0.6075 0.383 0.394 0.8715 0.448 0.7425 0.2895 0.1155 0.542
1 0.4275 0.557 0.7705 0.4945 0.5235 0.632 0.2755 0.692 0.362 0.128 0.7005
1 0.4055 0.3755 0.452 0.3295 0.2715 0.258 0.638 0.5035 0.0985 0.857
1 0.64 0.489 0.4605 0.4635 0.8275 0.5875 0.8115 0.3945 0.8185
1 0.3785 0.366 0.4445 0.5895 0.7795 0.7465 0.0825 0.9235
1 0.596 0.2025 0.233 0.8745 0.224 0.048 0.7885
1 0.1415 0.3015 0.569 0.102 0.0525 0.13
1 0.477 0.628 0.2835 0.0635 0.5075
1 0.4515 0.322 0.2115 0.5865
1 0.327 0.1315 0.771
1 0.3085 0.736
1 0.282
1
Placenta
Adipose
Adrenal
Brain
Breast
Colon
Heart
Kidney
Liver
Lung
LymphNode
Ovary
Prostate
SkeletalMuscle
Testes
Thyroid
WhiteBloodCells
EdomStromalCell

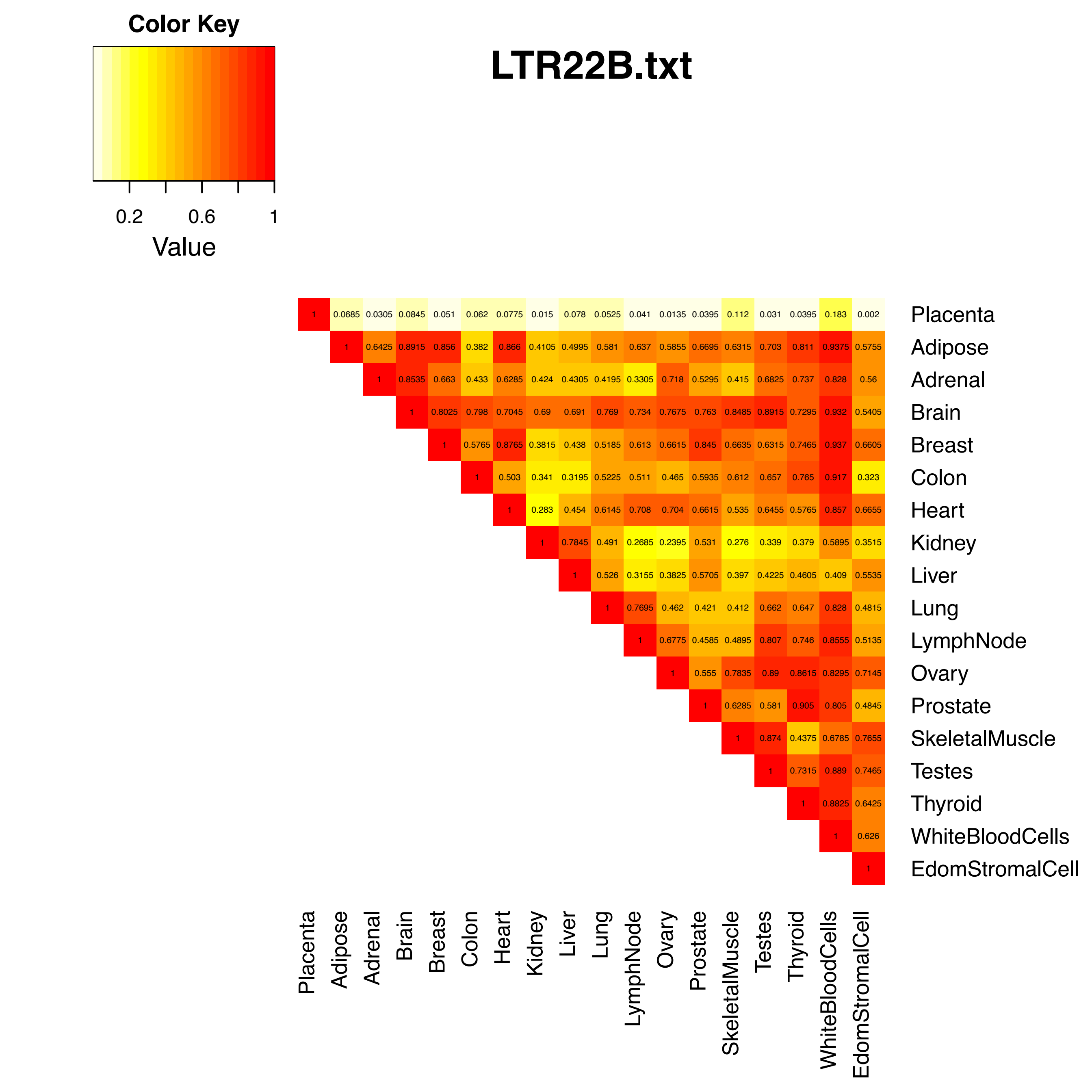
Color Key
0.2
0.6
1
Value
LTR22B.txt
1 0.0685 0.0305 0.0845 0.051 0.062 0.0775 0.015 0.078 0.0525 0.041 0.0135 0.0395 0.112 0.031 0.0395 0.183 0.002 Placenta
1 0.6425 0.8915 0.856 0.382 0.866 0.4105 0.4995 0.581 0.637 0.5855 0.6695 0.6315 0.703 0.811 0.9375 0.5755 Adipose
1 0.8535 0.663 0.433 0.6285 0.424 0.4305 0.4195 0.3305 0.718 0.5295 0.415 0.6825 0.737 0.828 0.56 Adrenal
1 0.8025 0.798 0.7045 0.69 0.691 0.769 0.734 0.7675 0.763 0.8485 0.8915 0.7295 0.932 0.5405 Brain
1 0.5765 0.8765 0.3815 0.438 0.5185 0.613 0.6615 0.845 0.6635 0.6315 0.7465 0.937 0.6605 Breast
1 0.503 0.341 0.3195 0.5225 0.511 0.465 0.5935 0.612 0.657 0.765 0.917 0.323 Colon
1 0.283 0.454 0.6145 0.708 0.704 0.6615 0.535 0.6455 0.5765 0.857 0.6655 Heart
1 0.7845 0.491 0.2685 0.2395 0.531 0.276 0.339 0.379 0.5895 0.3515 Kidney
1 0.526 0.3155 0.3825 0.5705 0.397 0.4225 0.4605 0.409 0.5535 Liver
1 0.7695 0.462 0.421 0.412 0.662 0.647 0.828 0.4815 Lung
1 0.6775 0.4585 0.4895 0.807 0.746 0.8555 0.5135 LymphNode
1 0.555 0.7835 0.89 0.8615 0.8295 0.7145 Ovary
1 0.6285 0.581 0.905 0.805 0.4845 Prostate
1 0.874 0.4375 0.6785 0.7655 SkeletalMuscle
1 0.7315 0.889 0.7465 Testes
1 0.8825 0.6425 Thyroid
1 0.626 WhiteBloodCells
1 EdomStromalCell
Placenta
Adipose
Adrenal
Brain
Breast
Colon
Heart
Kidney
Liver
Lung
LymphNode
Ovary
Prostate
SkeletalMuscle
Testes
Thyroid
WhiteBloodCells
EdomStromalCell

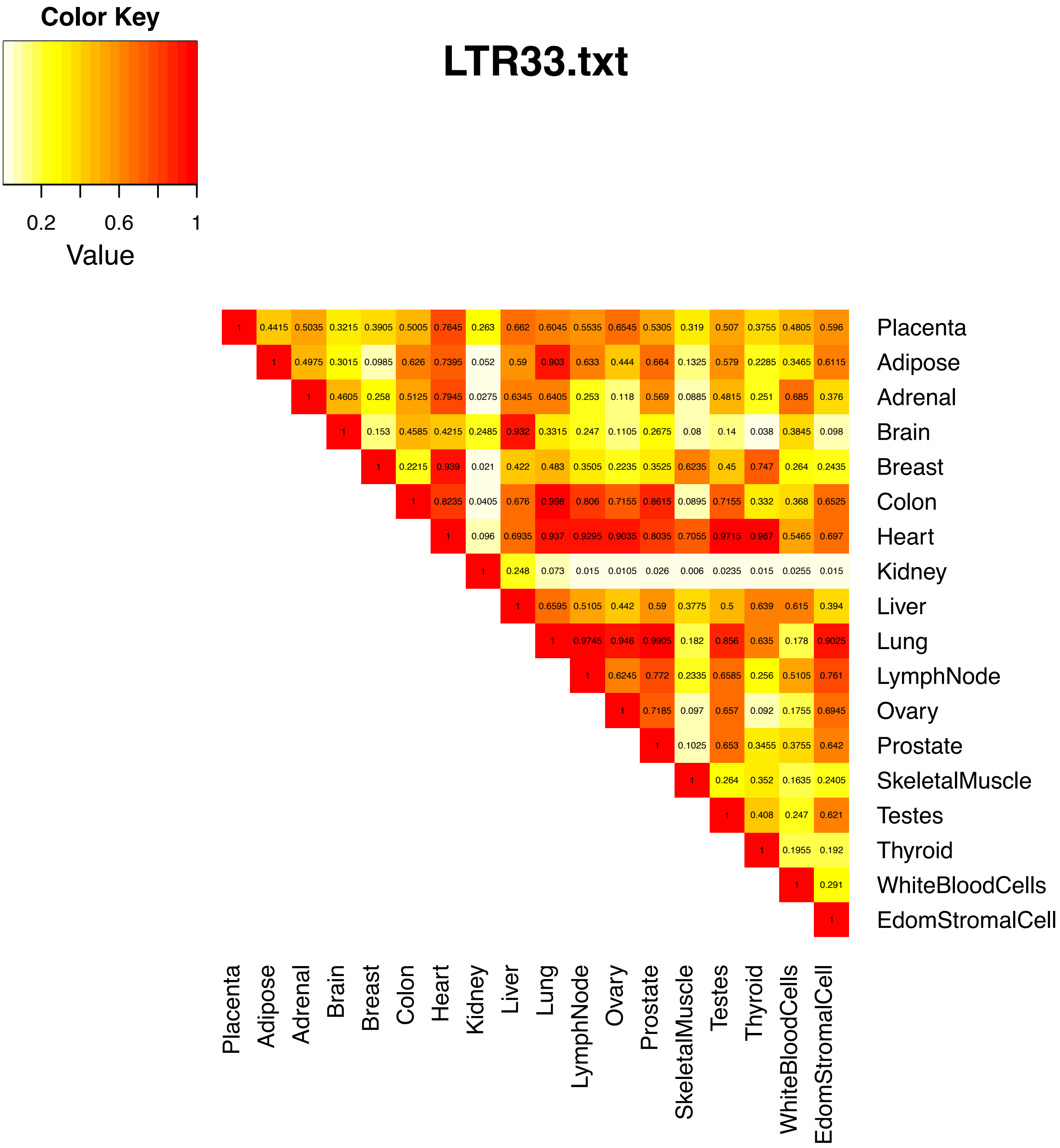
Color Key
0.2 0.6 1
Value
LTR33.txt
Placenta
Adipose
Adrenal
Brain
Breast
Colon
Heart
Kidney
Liver
Lung
LymphNode
Ovary
Prostate
SkeletalMuscle
Testes
Thyroid
WhiteBloodCells
EdomStromalCell
1 0.4415 0.5035 0.3215 0.3905 0.5005 0.7645 0.263 0.662 0.6045 0.5535 0.6545 0.5305 0.319 0.507 0.3755 0.4805 0.596
1 0.4975 0.3015 0.0985 0.626 0.7395 0.052 0.59 0.903 0.633 0.444 0.664 0.1325 0.579 0.2285 0.3465 0.6115
1 0.4605 0.258 0.5125 0.7945 0.0275 0.6345 0.6405 0.253 0.118 0.569 0.0885 0.4815 0.251 0.685 0.376
1 0.153 0.4585 0.4215 0.2485 0.932 0.3315 0.247 0.1105 0.2675 0.08 0.14 0.038 0.3845 0.098
1 0.2215 0.939 0.021 0.422 0.483 0.3505 0.2235 0.3525 0.6235 0.45 0.747 0.264 0.2435
1 0.8235 0.0405 0.676 0.998 0.806 0.7155 0.8615 0.0895 0.7155 0.332 0.368 0.6525
1 0.096 0.6935 0.937 0.9295 0.9035 0.8035 0.7055 0.9715 0.967 0.5465 0.697
1 0.248 0.073 0.015 0.0105 0.026 0.006 0.0235 0.015 0.0255 0.015
1 0.6595 0.5105 0.442 0.59 0.3775 0.5 0.639 0.615 0.394
1 0.9745 0.946 0.9905 0.182 0.856 0.635 0.178 0.9025
1 0.6245 0.772 0.2335 0.6585 0.256 0.5105 0.761
1 0.7185 0.097 0.657 0.092 0.1755 0.6945
1 0.1025 0.653 0.3455 0.3755 0.642
1 0.264 0.352 0.1635 0.2405
1 0.408 0.247 0.621
1 0.1955 0.192
1 0.291
1
Placenta
Adipose
Adrenal
Brain
Breast
Colon
Heart
Kidney
Liver
Lung
LymphNode
Ovary
Prostate
SkeletalMuscle
Testes
Thyroid
WhiteBloodCells
EdomStromalCell

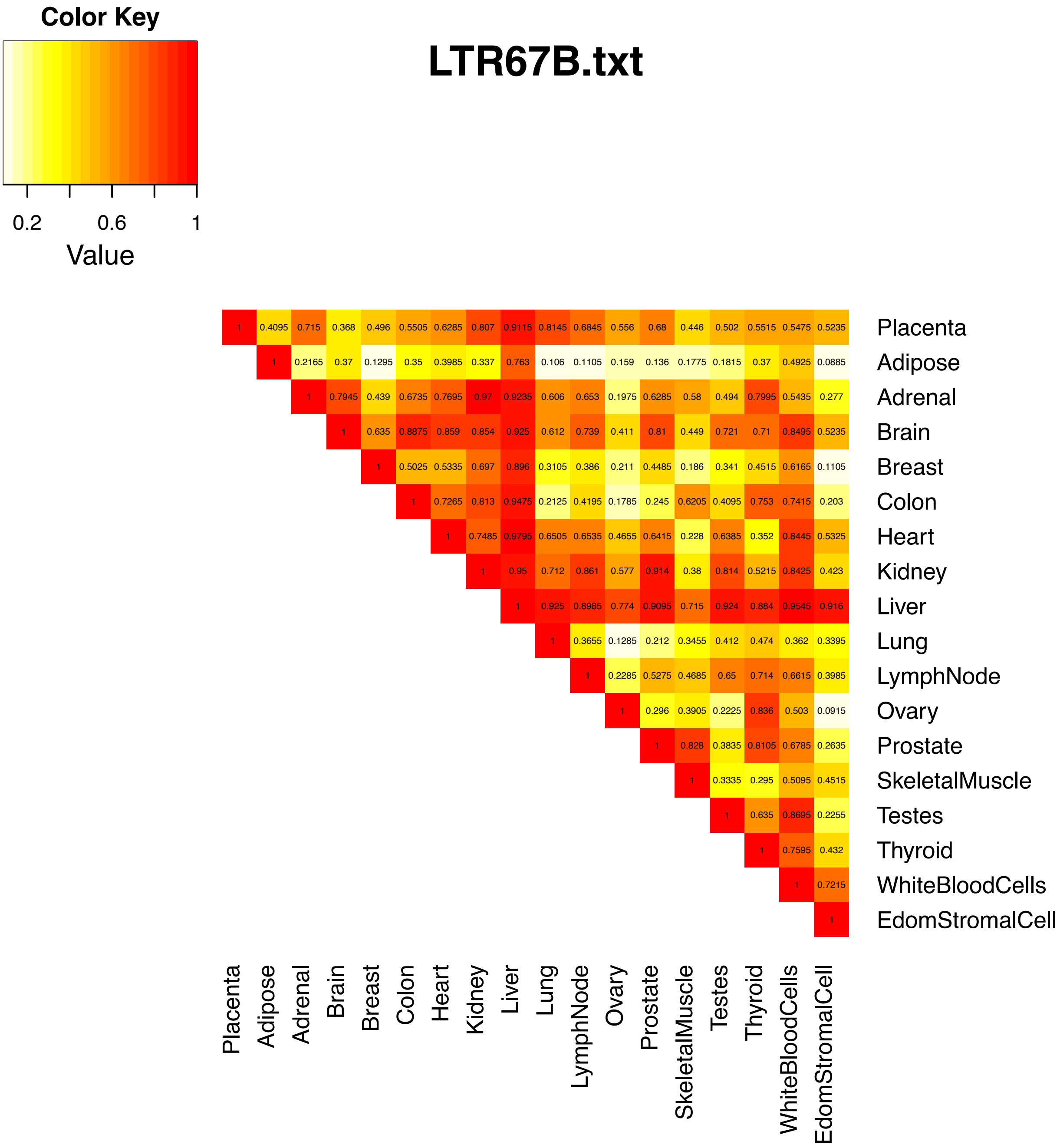
Color Key
0.2
0.6
1
Value
LTR67B.txt
Placenta 1 0.4095 0.715 0.368 0.496 0.5505 0.6285 0.807 0.9115 0.8145 0.6845 0.556 0.68 0.446 0.502 0.5515 0.5475 0.5235
Adipose 1 0.2165 0.37 0.1295 0.35 0.3985 0.337 0.763 0.106 0.1105 0.159 0.136 0.1775 0.1815 0.37 0.4925 0.0885
Adrenal 1 0.7945 0.439 0.6735 0.7695 0.97 0.9235 0.606 0.653 0.1975 0.6285 0.58 0.494 0.7995 0.5435 0.277
Brain 1 0.635 0.8875 0.859 0.854 0.925 0.612 0.739 0.411 0.81 0.449 0.721 0.71 0.8495 0.5235
Breast 1 0.5025 0.5335 0.697 0.896 0.3105 0.386 0.211 0.4485 0.186 0.341 0.4515 0.6165 0.1105
Colon 1 0.7265 0.813 0.9475 0.2125 0.4195 0.1785 0.245 0.6205 0.4095 0.753 0.7415 0.203
Heart 1 0.7485 0.9795 0.6505 0.6535 0.4655 0.6415 0.228 0.6385 0.352 0.8445 0.5325
Kidney 1 0.95 0.712 0.861 0.577 0.914 0.38 0.814 0.5215 0.8425 0.423
Liver 1 0.925 0.8985 0.774 0.9095 0.715 0.924 0.884 0.9545 0.916
Lung 1 0.3655 0.1285 0.212 0.3455 0.412 0.474 0.362 0.3395
LymphNode 1 0.2285 0.5275 0.4685 0.65 0.714 0.6615 0.3985
Ovary 1 0.296 0.3905 0.2225 0.836 0.503 0.0915
Prostate 1 0.828 0.3835 0.8105 0.6785 0.2635
SkeletalMuscle 1 0.3335 0.295 0.5095 0.4515
Testes 1 0.635 0.8695 0.2255
Thyroid 1 0.7595 0.432
WhiteBloodCells 1 0.7215
EdomStromalCell 1
Placenta Adipose Adrenal Brain Breast Colon Heart Kidney Liver Lung LymphNode Ovary Prostate SkeletalMuscle Testes Thyroid WhiteBloodCells EdomStromalCell

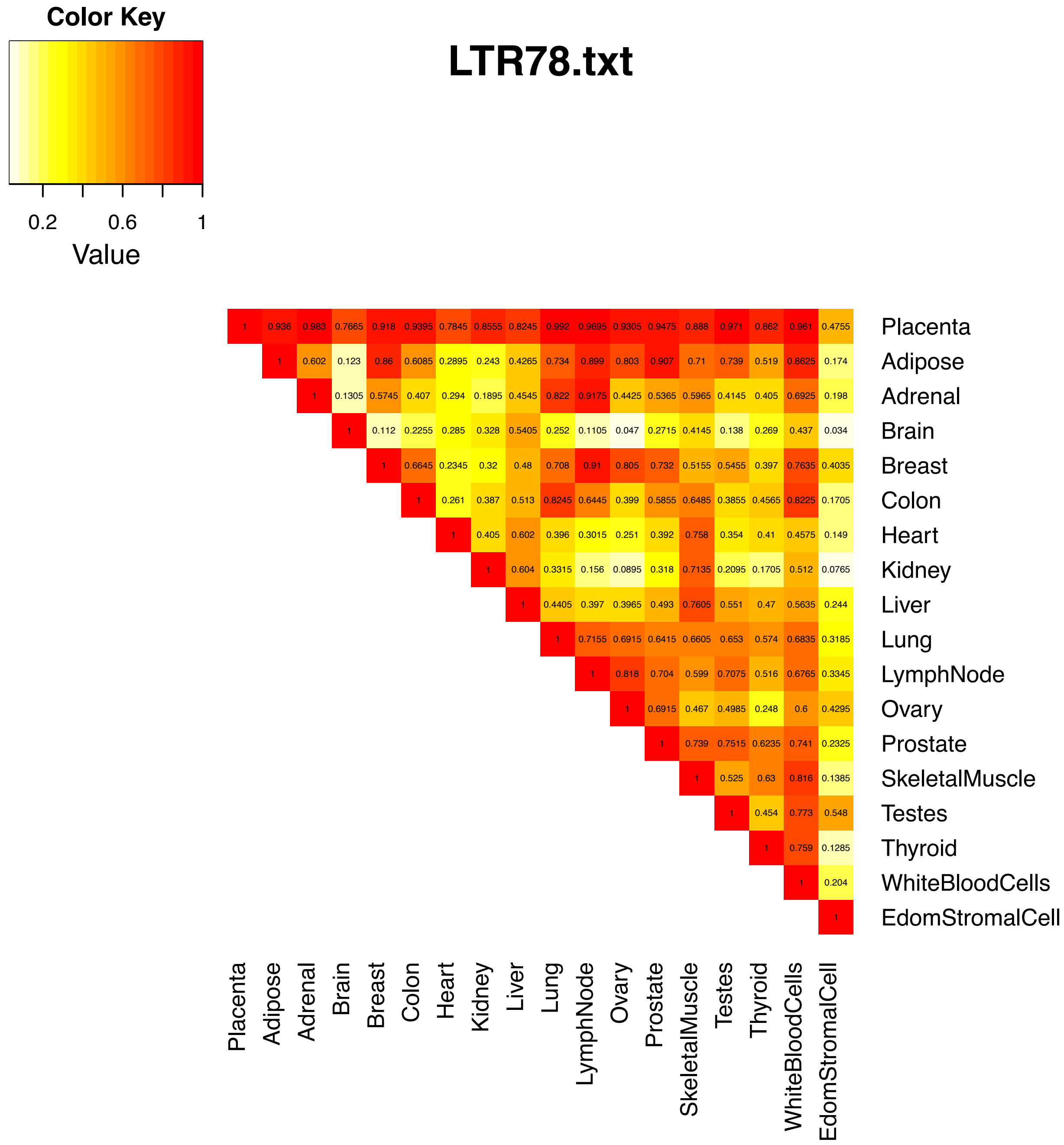
LTR78.txt
Color Key
0.2
0.6
1
Value
Placenta: 1 0.936 0.983 0.7665 0.918 0.9395 0.7845 0.8555 0.8245 0.992 0.9695 0.9305 0.9475 0.888 0.971 0.862 0.961 0.4755
Adipose: 1 0.602 0.123 0.86 0.6085 0.2895 0.243 0.4265 0.734 0.899 0.803 0.907 0.71 0.739 0.519 0.8625 0.174
Adrenal: 1 0.1305 0.5745 0.407 0.294 0.1895 0.4545 0.822 0.9175 0.4425 0.5365 0.5965 0.4145 0.405 0.6925 0.198
Brain: 1 0.112 0.2255 0.285 0.328 0.5405 0.252 0.1105 0.047 0.2715 0.4145 0.138 0.269 0.437 0.034
Breast: 1 0.6645 0.2345 0.32 0.48 0.708 0.91 0.805 0.732 0.5155 0.5455 0.397 0.7635 0.4035
Colon: 1 0.261 0.387 0.513 0.8245 0.6445 0.399 0.5855 0.6485 0.3855 0.4565 0.8225 0.1705
Heart: 1 0.405 0.602 0.396 0.3015 0.251 0.392 0.758 0.354 0.41 0.4575 0.149
Kidney: 1 0.604 0.3315 0.156 0.0895 0.318 0.7135 0.2095 0.1705 0.512 0.0765
Liver: 1 0.4405 0.397 0.3965 0.493 0.7605 0.551 0.47 0.5635 0.244
Lung: 1 0.7155 0.6915 0.6415 0.6605 0.653 0.574 0.6835 0.3185
LymphNode: 1 0.818 0.704 0.599 0.7075 0.516 0.6765 0.3345
Ovary: 1 0.6915 0.467 0.4985 0.248 0.6 0.4295
Prostate: 1 0.739 0.7515 0.6235 0.741 0.2325
SkeletalMuscle: 1 0.525 0.63 0.816 0.1385
Testes: 1 0.454 0.773 0.548
Thyroid: 1 0.759 0.1285
WhiteBloodCells: 1 0.204
EdomStromalCell: 1
Placenta
Adipose
Adrenal
Brain
Breast
Colon
Heart
Kidney
Liver
Lung
LymphNode
Ovary
Prostate
SkeletalMuscle
Testes
Thyroid
WhiteBloodCells
EdomStromalCell

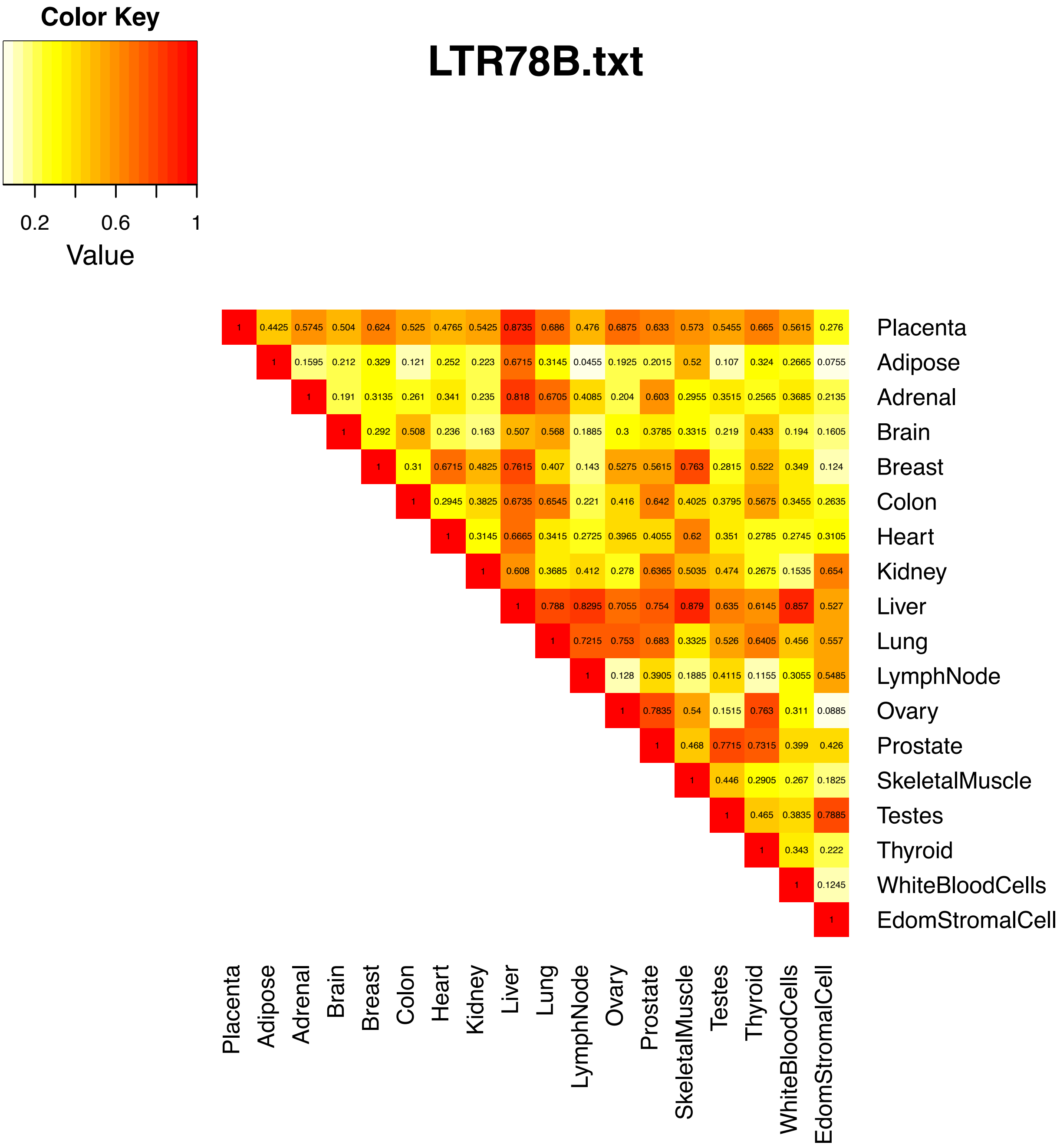
Color Key
0.2
0.6
1
Value
LTR78B.txt
Placenta 1 0.4425 0.5745 0.504 0.624 0.525 0.4765 0.5425 0.8735 0.686 0.476 0.6875 0.633 0.573 0.5455 0.665 0.5615 0.276
Adipose 1 0.1595 0.212 0.329 0.121 0.252 0.223 0.6715 0.3145 0.0455 0.1925 0.2015 0.52 0.107 0.324 0.2665 0.0755
Adrenal 1 0.191 0.3135 0.261 0.341 0.235 0.818 0.6705 0.4085 0.204 0.603 0.2955 0.3515 0.2565 0.3685 0.2135
Brain 1 0.292 0.508 0.236 0.163 0.507 0.568 0.1885 0.3 0.3785 0.3315 0.219 0.433 0.194 0.1605
Breast 1 0.31 0.6715 0.4825 0.7615 0.407 0.143 0.5275 0.5615 0.763 0.2815 0.522 0.349 0.124
Colon 1 0.2945 0.3825 0.6735 0.6545 0.221 0.416 0.642 0.4025 0.3795 0.5675 0.3455 0.2635
Heart 1 0.3145 0.6665 0.3415 0.2725 0.3965 0.4055 0.62 0.351 0.2785 0.2745 0.3105
Kidney 1 0.608 0.3685 0.412 0.278 0.6365 0.5035 0.474 0.2675 0.1535 0.654
Liver 1 0.788 0.8295 0.7055 0.754 0.879 0.635 0.6145 0.857 0.527
Lung 1 0.7215 0.753 0.683 0.3325 0.526 0.6405 0.456 0.557
LymphNode 1 0.128 0.3905 0.1885 0.4115 0.1155 0.3055 0.5485
Ovary 1 0.7835 0.54 0.1515 0.763 0.311 0.0885
Prostate 1 0.468 0.7715 0.7315 0.399 0.426
SkeletalMuscle 1 0.446 0.2905 0.267 0.1825
Testes 1 0.465 0.3835 0.7885
Thyroid 1 0.343 0.222
WhiteBloodCells 1 0.1245
EdomStromalCell 1
Placenta Adipose Adrenal Brain Breast Colon Heart Kidney Liver Lung LymphNode Ovary Prostate SkeletalMuscle Testes Thyroid WhiteBloodCells EdomStromalCell

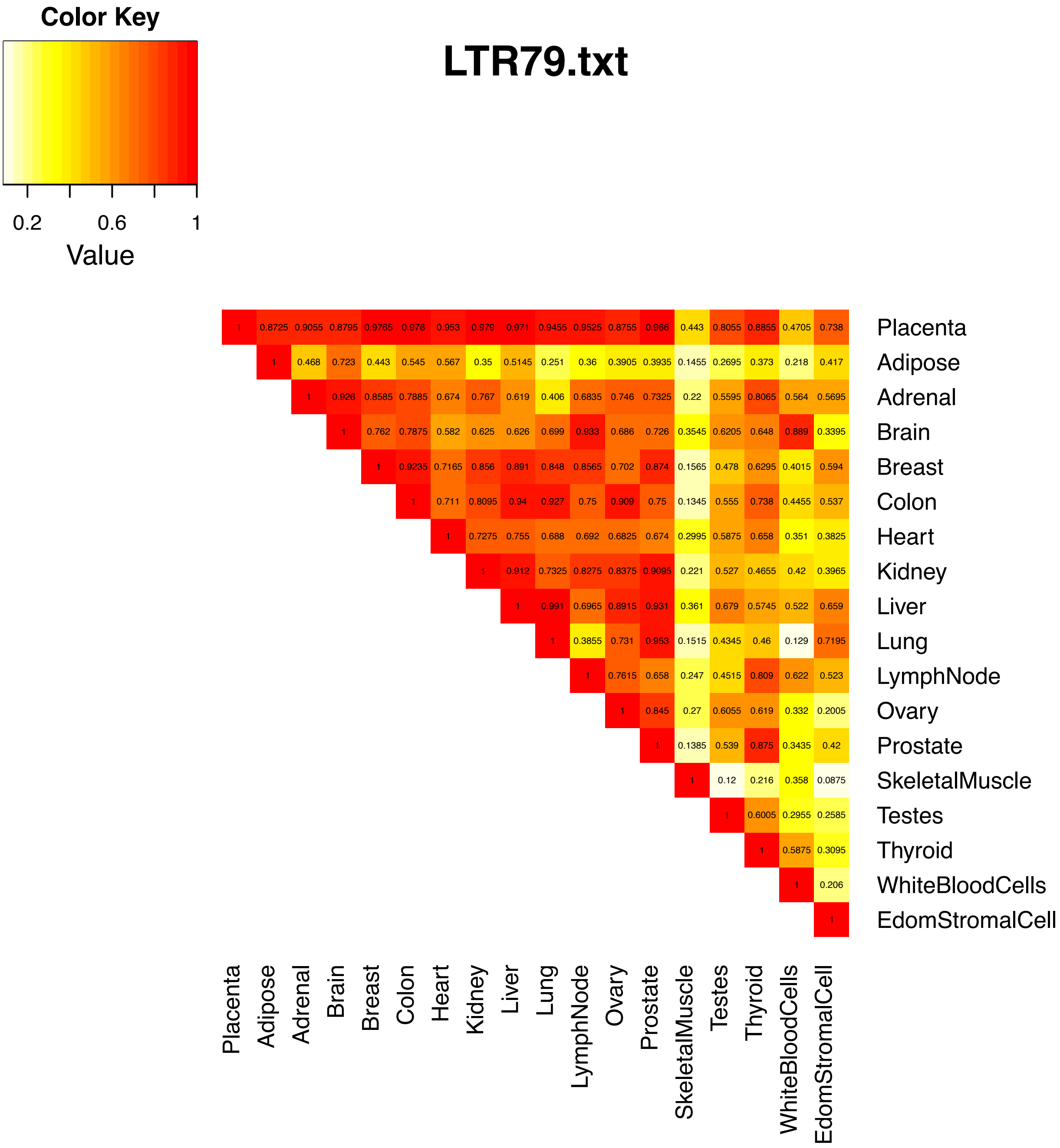

Color Key
0.2
0.6
1
Value
LTR79.txt
1 0.8725 0.9055 0.8795 0.9765 0.976 0.953 0.979 0.971 0.9455 0.9525 0.8755 0.966 0.443 0.8055 0.8855 0.4705 0.738 Placenta
1 0.468 0.723 0.443 0.545 0.567 0.35 0.5145 0.251 0.36 0.3905 0.3935 0.1455 0.2695 0.373 0.218 0.417 Adipose
1 0.926 0.8585 0.7885 0.674 0.767 0.619 0.406 0.6835 0.746 0.7325 0.22 0.5595 0.8065 0.564 0.5695 Adrenal
1 0.762 0.7875 0.582 0.625 0.626 0.699 0.933 0.686 0.726 0.3545 0.6205 0.648 0.889 0.3395 Brain
1 0.9235 0.7165 0.856 0.891 0.848 0.8565 0.702 0.874 0.1565 0.478 0.6295 0.4015 0.594 Breast
1 0.711 0.8095 0.94 0.927 0.75 0.909 0.75 0.1345 0.555 0.738 0.4455 0.537 Colon
1 0.7275 0.755 0.688 0.692 0.6825 0.674 0.2995 0.5875 0.658 0.351 0.3825 Heart
1 0.912 0.7325 0.8275 0.8375 0.9095 0.221 0.527 0.4655 0.42 0.3965 Kidney
1 0.991 0.6965 0.8915 0.931 0.361 0.679 0.5745 0.522 0.659 Liver
1 0.3855 0.731 0.953 0.1515 0.4345 0.46 0.129 0.7195 Lung
1 0.7615 0.658 0.247 0.4515 0.809 0.622 0.523 LymphNode
1 0.845 0.27 0.6055 0.619 0.332 0.2005 Ovary
1 0.1385 0.539 0.875 0.3435 0.42 Prostate
1 0.12 0.216 0.358 0.0875 SkeletalMuscle
1 0.6005 0.2955 0.2585 Testes
1 0.5875 0.3095 Thyroid
1 0.206 WhiteBloodCells
1 EdomStromalCell
Placenta
Adipose
Adrenal
Brain
Breast
Colon
Heart
Kidney
Liver
Lung
LymphNode
Ovary
Prostate
SkeletalMuscle
Testes
Thyroid
WhiteBloodCells
EdomStromalCell

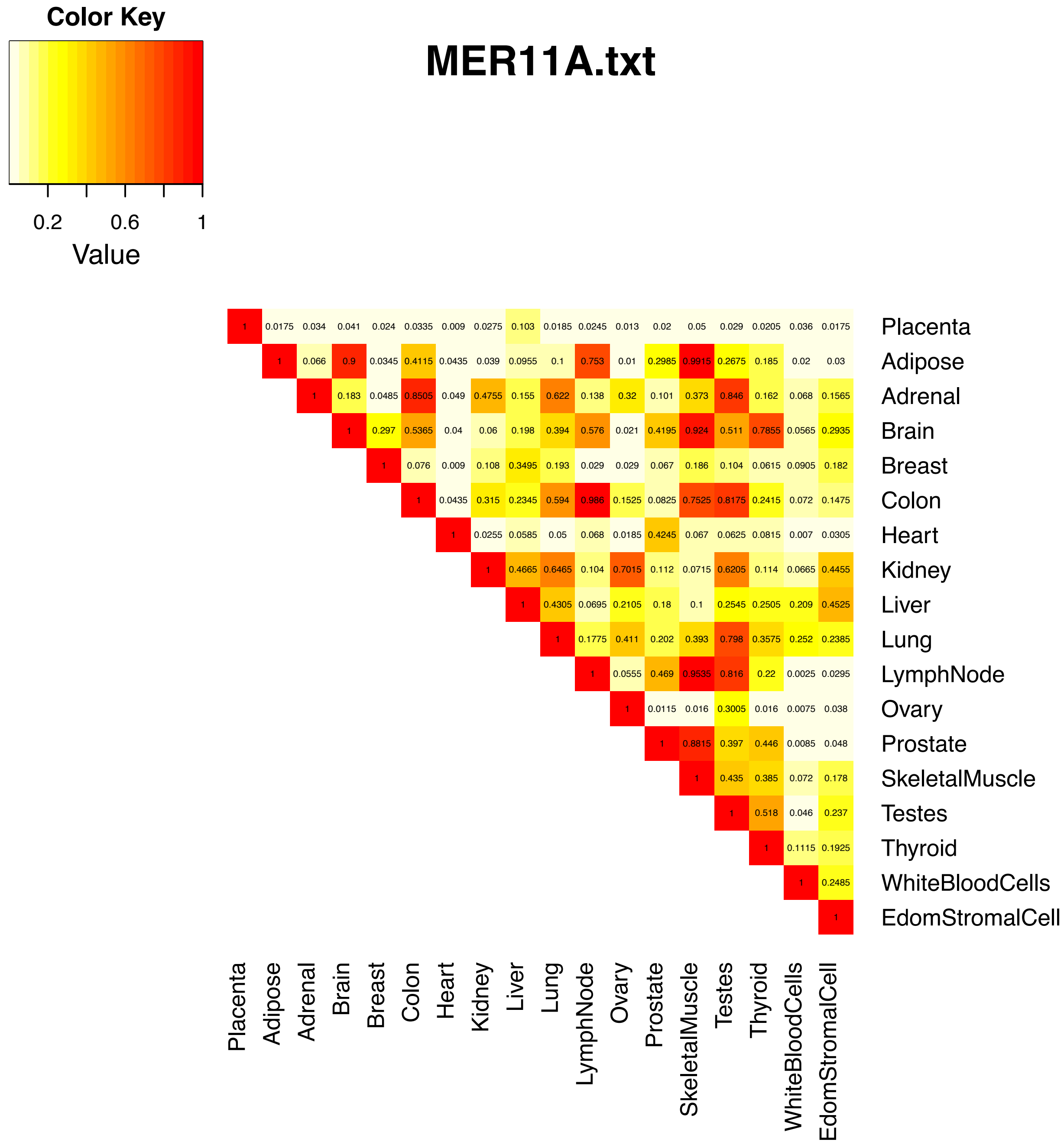
MER11A.txt
Color Key
0.2
0.6
1
Value
1 0.0175 0.034 0.041 0.024 0.0335 0.009 0.0275 0.103 0.0185 0.0245 0.013 0.02 0.05 0.029 0.0205 0.036 0.0175 Placenta
1 0.066 0.9 0.0345 0.4115 0.0435 0.039 0.0955 0.1 0.753 0.01 0.2985 0.9915 0.2675 0.185 0.02 0.03 Adipose
1 0.183 0.0485 0.8505 0.049 0.4755 0.155 0.622 0.138 0.32 0.101 0.373 0.846 0.162 0.068 0.1565 Adrenal
1 0.297 0.5365 0.04 0.06 0.198 0.394 0.576 0.021 0.4195 0.924 0.511 0.7855 0.0565 0.2935 Brain
1 0.076 0.009 0.108 0.3495 0.193 0.029 0.029 0.067 0.186 0.104 0.0615 0.0905 0.182 Breast
1 0.0435 0.315 0.2345 0.594 0.986 0.1525 0.0825 0.7525 0.8175 0.2415 0.072 0.1475 Colon
1 0.0255 0.0585 0.05 0.068 0.0185 0.4245 0.067 0.0625 0.0815 0.007 0.0305 Heart
1 0.4665 0.6465 0.104 0.7015 0.112 0.0715 0.6205 0.114 0.0665 0.4455 Kidney
1 0.4305 0.0695 0.2105 0.18 0.1 0.2545 0.2505 0.209 0.4525 Liver
1 0.1775 0.411 0.202 0.393 0.798 0.3575 0.252 0.2385 Lung
1 0.0555 0.469 0.9535 0.816 0.22 0.0025 0.0295 LymphNode
1 0.0115 0.016 0.3005 0.016 0.0075 0.038 Ovary
1 0.8815 0.397 0.446 0.0085 0.048 Prostate
1 0.435 0.385 0.072 0.178 SkeletalMuscle
1 0.518 0.046 0.237 Testes
1 0.1115 0.1925 Thyroid
1 0.2485 WhiteBloodCells
1 EdomStromalCell
Placenta
Adipose
Adrenal
Brain
Breast
Colon
Heart
Kidney
Liver
Lung
LymphNode
Ovary
Prostate
SkeletalMuscle
Testes
Thyroid
WhiteBloodCells
EdomStromalCell

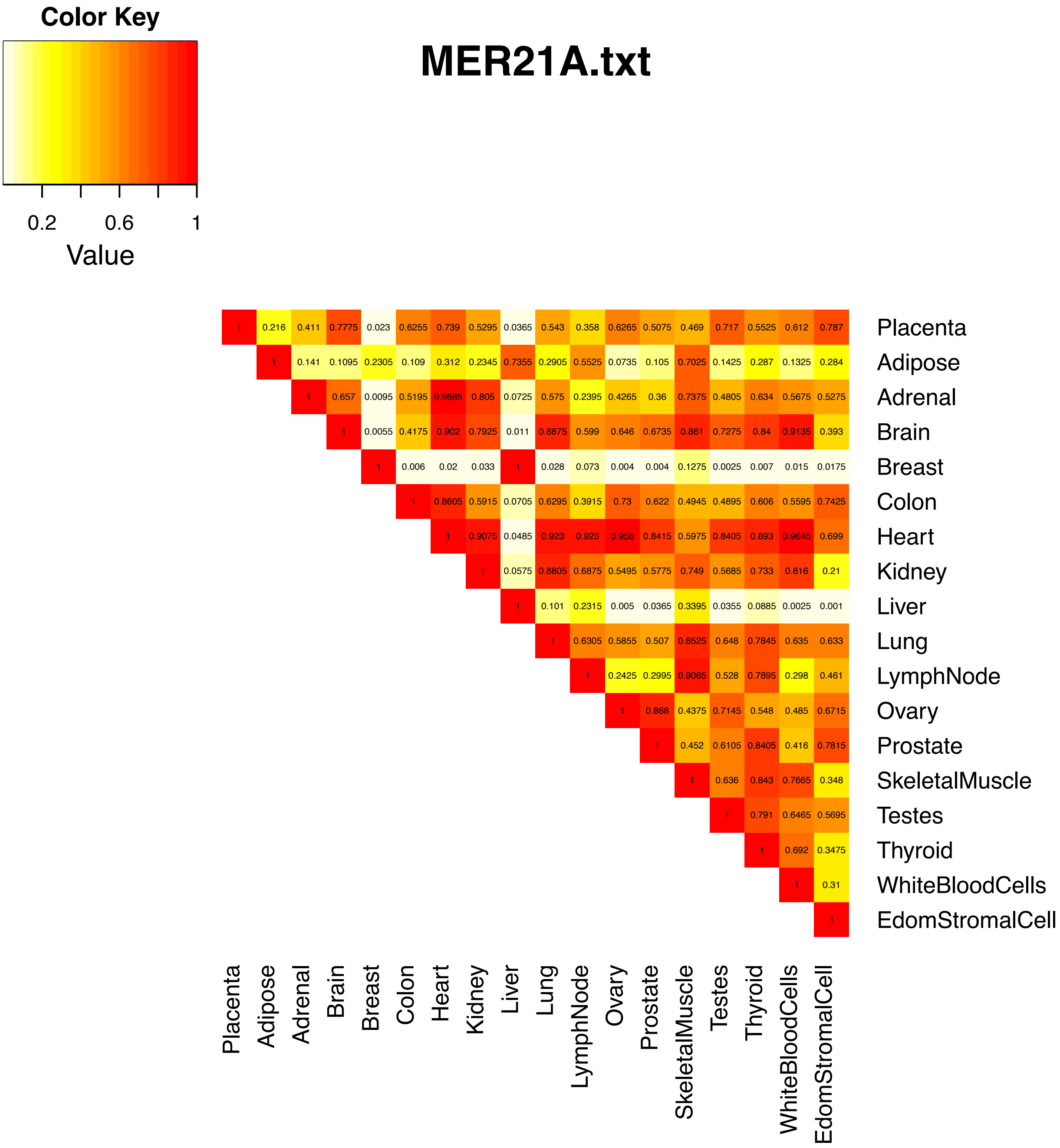
Color Key
0.2 0.6 1
Value
MER21A.txt
Placenta 1 0.216 0.411 0.7775 0.023 0.6255 0.739 0.5295 0.0365 0.543 0.358 0.6265 0.5075 0.469 0.717 0.5525 0.612 0.787
Adipose 1 0.141 0.1095 0.2305 0.109 0.312 0.2345 0.7355 0.2905 0.5525 0.0735 0.105 0.7025 0.1425 0.287 0.1325 0.284
Adrenal 1 0.657 0.0095 0.5195 0.9685 0.805 0.0725 0.575 0.2395 0.4265 0.36 0.7375 0.4805 0.634 0.5675 0.5275
Brain 1 0.0055 0.4175 0.902 0.7925 0.011 0.8875 0.599 0.646 0.6735 0.861 0.7275 0.84 0.9135 0.393
Breast 1 0.006 0.02 0.033 1 0.028 0.073 0.004 0.004 0.1275 0.0025 0.007 0.015 0.0175
Colon 1 0.8605 0.5915 0.0705 0.6295 0.3915 0.73 0.622 0.4945 0.4895 0.606 0.5595 0.7425
Heart 1 0.9075 0.0485 0.923 0.923 0.956 0.8415 0.5975 0.8405 0.893 0.9645 0.699
Kidney 1 0.0575 0.8805 0.6875 0.5495 0.5775 0.749 0.5685 0.733 0.816 0.21
Liver 1 0.101 0.2315 0.005 0.0365 0.3395 0.0355 0.0885 0.0025 0.001
Lung 1 0.6305 0.5855 0.507 0.8525 0.648 0.7845 0.635 0.633
LymphNode 1 0.2425 0.2995 0.9065 0.528 0.7895 0.298 0.461
Ovary 1 0.868 0.4375 0.7145 0.548 0.485 0.6715
Prostate 1 0.452 0.6105 0.8405 0.416 0.7815
SkeletalMuscle 1 0.636 0.843 0.7665 0.348
Testes 1 0.791 0.6465 0.5695
Thyroid 1 0.692 0.3475
WhiteBloodCells 1 0.31
EdomStromalCell 1
Placenta Adipose Adrenal Brain Breast Colon Heart Kidney Liver Lung LymphNode Ovary Prostate SkeletalMuscle Testes Thyroid WhiteBloodCells EdomStromalCell

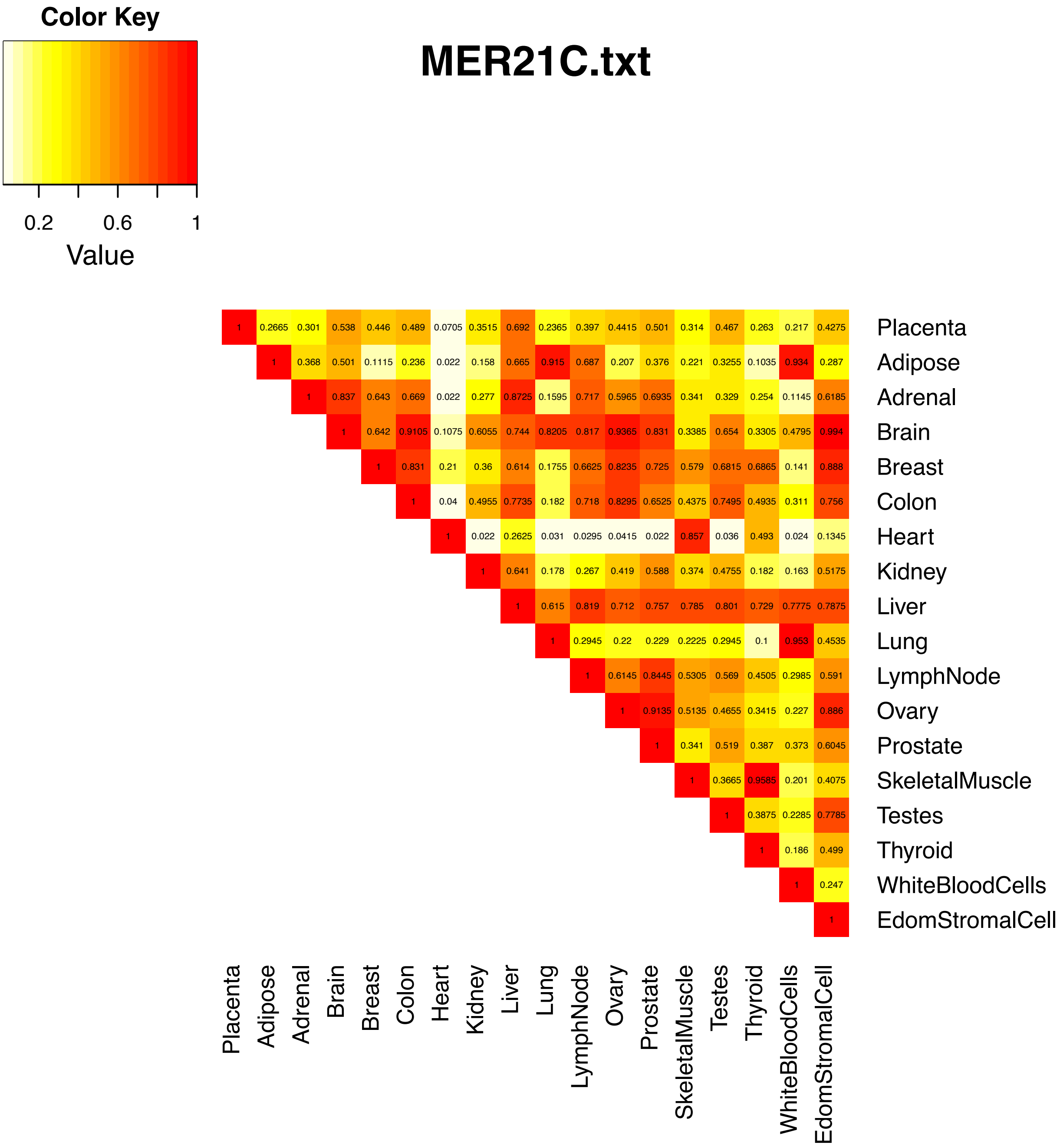
MER21C.txt
Color Key
0.2
0.6
1
Value
Placenta 1 0.2665 0.301 0.538 0.446 0.489 0.0705 0.3515 0.692 0.2365 0.397 0.4415 0.501 0.314 0.467 0.263 0.217 0.4275
Adipose 1 0.368 0.501 0.1115 0.236 0.022 0.158 0.665 0.915 0.687 0.207 0.376 0.221 0.3255 0.1035 0.934 0.287
Adrenal 1 0.837 0.643 0.669 0.022 0.277 0.8725 0.1595 0.717 0.5965 0.6935 0.341 0.329 0.254 0.1145 0.6185
Brain 1 0.642 0.9105 0.1075 0.6055 0.744 0.8205 0.817 0.9365 0.831 0.3385 0.654 0.3305 0.4795 0.994
Breast 1 0.831 0.21 0.36 0.614 0.1755 0.6625 0.8235 0.725 0.579 0.6815 0.6865 0.141 0.888
Colon 1 0.04 0.4955 0.7735 0.182 0.718 0.8295 0.6525 0.4375 0.7495 0.4935 0.311 0.756
Heart 1 0.022 0.2625 0.031 0.0295 0.0415 0.022 0.857 0.036 0.493 0.024 0.1345
Kidney 1 0.641 0.178 0.267 0.419 0.588 0.374 0.4755 0.182 0.163 0.5175
Liver 1 0.615 0.819 0.712 0.757 0.785 0.801 0.729 0.7775 0.7875
Lung 1 0.2945 0.22 0.229 0.2225 0.2945 0.1 0.953 0.4535
LymphNode 1 0.6145 0.8445 0.5305 0.569 0.4505 0.2985 0.591
Ovary 1 0.9135 0.5135 0.4655 0.3415 0.227 0.886
Prostate 1 0.341 0.519 0.387 0.373 0.6045
SkeletalMuscle 1 0.3665 0.9585 0.201 0.4075
Testes 1 0.3875 0.2285 0.7785
Thyroid 1 0.186 0.499
WhiteBloodCells 1 0.247
EdomStromalCell 1
Placenta
Adipose
Adrenal
Brain
Breast
Colon
Heart
Kidney
Liver
Lung
LymphNode
Ovary
Prostate
SkeletalMuscle
Testes
Thyroid
WhiteBloodCells
EdomStromalCell

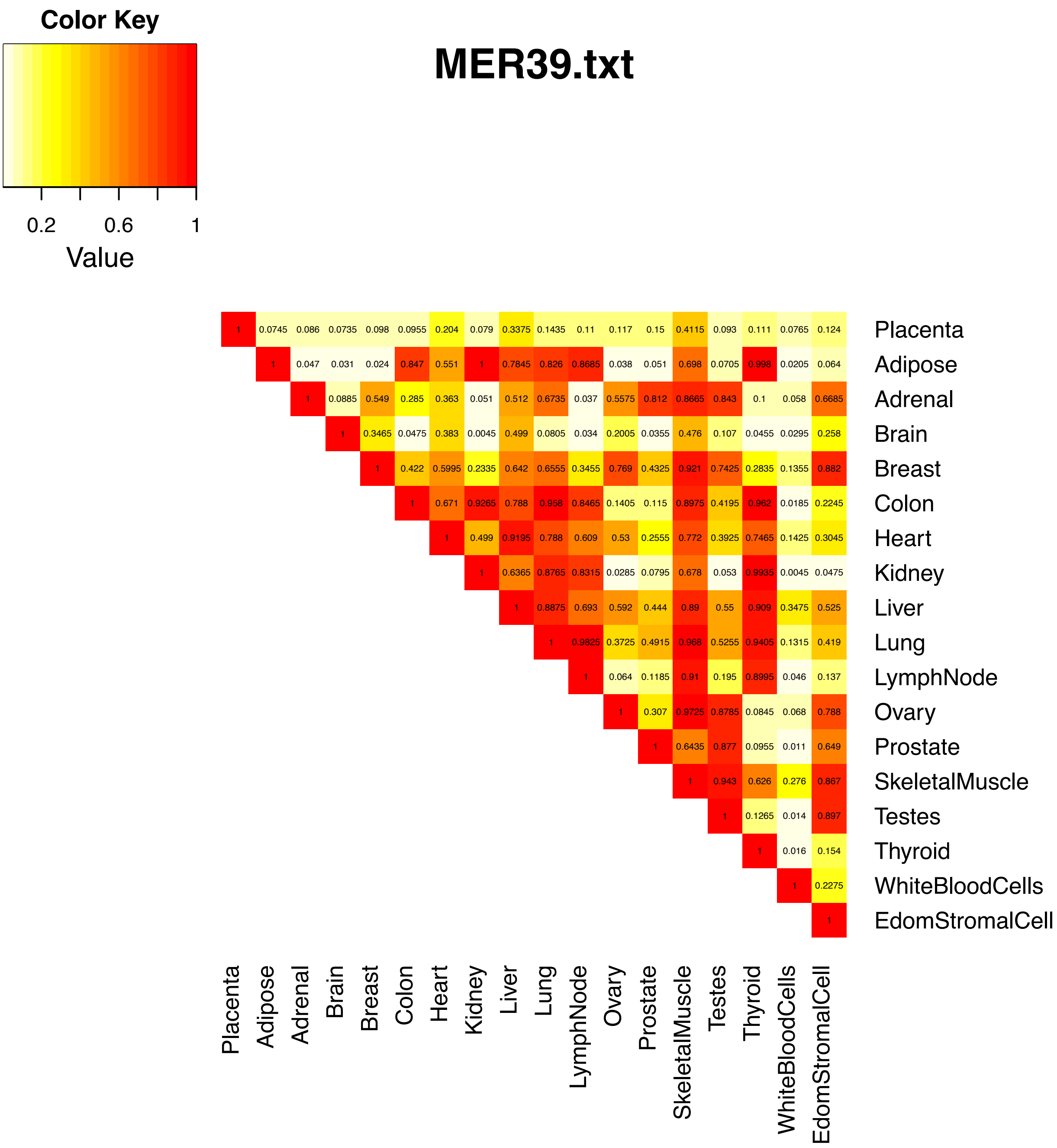
Color Key
0.2
0.6
1
Value
MER39.txt
Placenta
Adipose
Adrenal
Brain
Breast
Colon
Heart
Kidney
Liver
Lung
LymphNode
Ovary
Prostate
SkeletalMuscle
Testes
Thyroid
WhiteBloodCells
EdomStromalCell

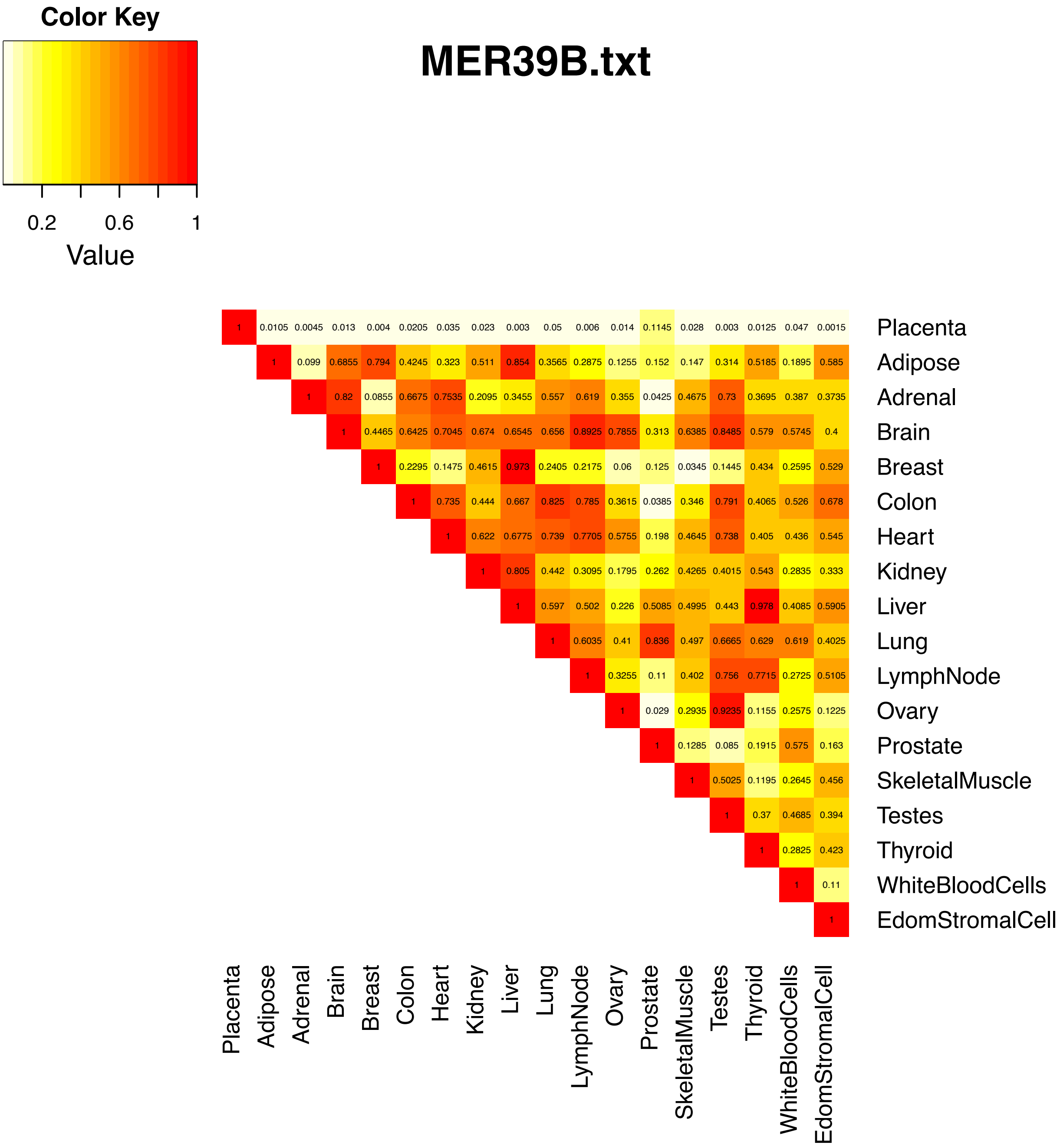
Color Key
0.2
0.6
1
Value
MER39B.txt
Placenta: 1 0.0105 0.0045 0.013 0.004 0.0205 0.035 0.023 0.003 0.05 0.006 0.014 0.1145 0.028 0.003 0.0125 0.047 0.0015
Adipose: 1 0.099 0.6855 0.794 0.4245 0.323 0.511 0.854 0.3565 0.2875 0.1255 0.152 0.147 0.314 0.5185 0.1895 0.585
Adrenal: 1 0.82 0.0855 0.6675 0.7535 0.2095 0.3455 0.557 0.619 0.355 0.0425 0.4675 0.73 0.3695 0.387 0.3735
Brain: 1 0.4465 0.6425 0.7045 0.674 0.6545 0.656 0.8925 0.7855 0.313 0.6385 0.8485 0.579 0.5745 0.4
Breast: 1 0.2295 0.1475 0.4615 0.973 0.2405 0.2175 0.06 0.125 0.0345 0.1445 0.434 0.2595 0.529
Colon: 1 0.735 0.444 0.667 0.825 0.785 0.3615 0.0385 0.346 0.791 0.4065 0.526 0.678
Heart: 1 0.622 0.6775 0.739 0.7705 0.5755 0.198 0.4645 0.738 0.405 0.436 0.545
Kidney: 1 0.805 0.442 0.3095 0.1795 0.262 0.4265 0.4015 0.543 0.2835 0.333
Liver: 1 0.597 0.502 0.226 0.5085 0.4995 0.443 0.978 0.4085 0.5905
Lung: 1 0.6035 0.41 0.836 0.497 0.6665 0.629 0.619 0.4025
LymphNode: 1 0.3255 0.11 0.402 0.756 0.7715 0.2725 0.5105
Ovary: 1 0.029 0.2935 0.9235 0.1155 0.2575 0.1225
Prostate: 1 0.1285 0.085 0.1915 0.575 0.163
SkeletalMuscle: 1 0.5025 0.1195 0.2645 0.456
Testes: 1 0.37 0.4685 0.394
Thyroid: 1 0.2825 0.423
WhiteBloodCells: 1 0.11
EdomStromalCell: 1
Placenta Adipose Adrenal Brain Breast Colon Heart Kidney Liver Lung LymphNode Ovary Prostate SkeletalMuscle Testes Thyroid WhiteBloodCells EdomStromalCell

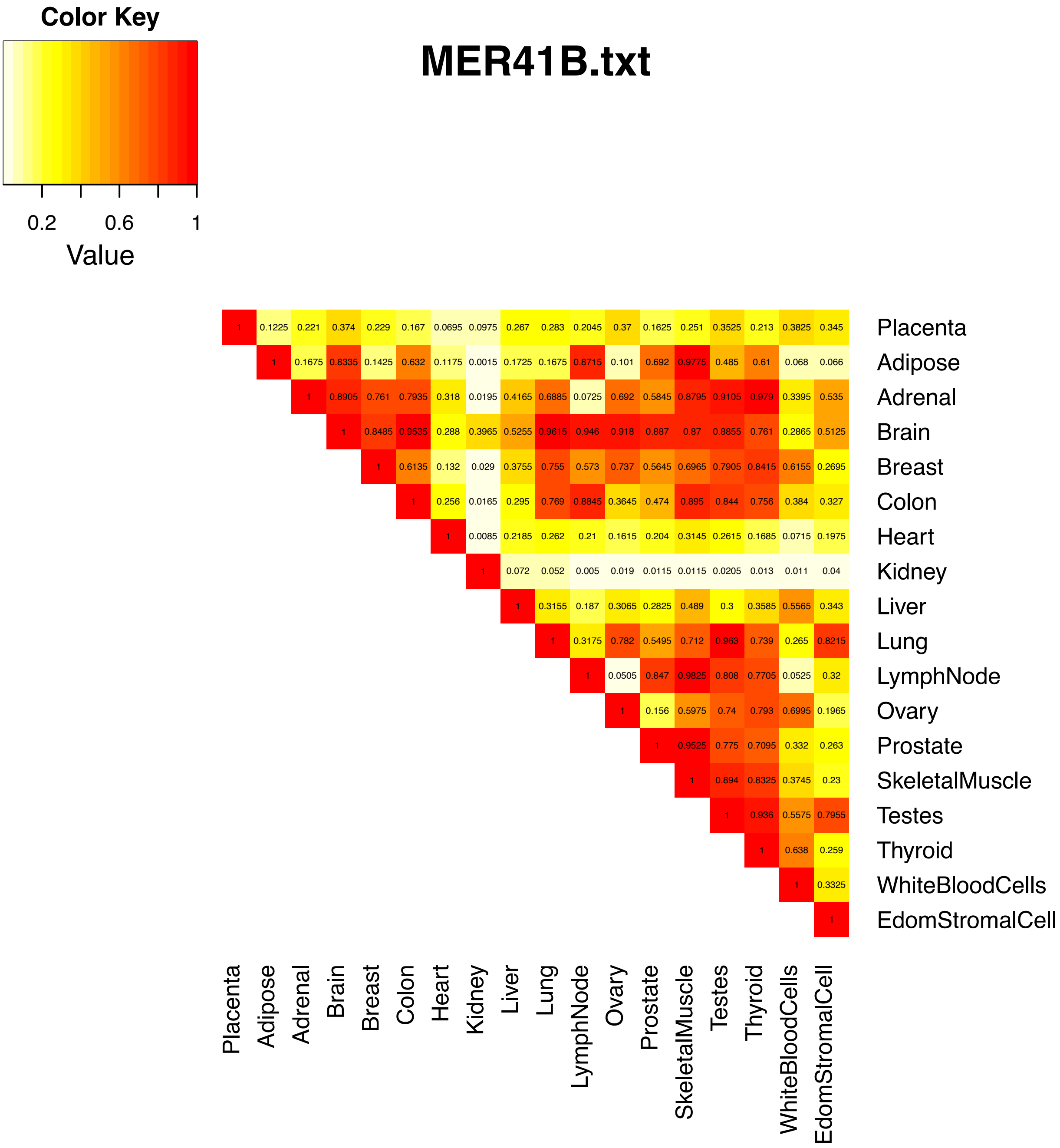
MER41B.txt
Color Key
0.2 0.6 1
Value
Placenta: 1 0.1225 0.221 0.374 0.229 0.167 0.0695 0.0975 0.267 0.283 0.2045 0.37 0.1625 0.251 0.3525 0.213 0.3825 0.345
Adipose: 1 0.1675 0.8335 0.1425 0.632 0.1175 0.0015 0.1725 0.1675 0.8715 0.101 0.692 0.9775 0.485 0.61 0.068 0.066
Adrenal: 1 0.8905 0.761 0.7935 0.318 0.0195 0.4165 0.6885 0.0725 0.692 0.5845 0.8795 0.9105 0.979 0.3395 0.535
Brain: 1 0.8485 0.9535 0.288 0.3965 0.5255 0.9615 0.946 0.918 0.887 0.87 0.8855 0.761 0.2865 0.5125
Breast: 1 0.6135 0.132 0.029 0.3755 0.755 0.573 0.737 0.5645 0.6965 0.7905 0.8415 0.6155 0.2695
Colon: 1 0.256 0.0165 0.295 0.769 0.8845 0.3645 0.474 0.895 0.844 0.756 0.384 0.327
Heart: 1 0.0085 0.2185 0.262 0.21 0.1615 0.204 0.3145 0.2615 0.1685 0.0715 0.1975
Kidney: 1 0.072 0.052 0.005 0.019 0.0115 0.0115 0.0205 0.013 0.011 0.04
Liver: 1 0.3155 0.187 0.3065 0.2825 0.489 0.3 0.3585 0.5565 0.343
Lung: 1 0.3175 0.782 0.5495 0.712 0.963 0.739 0.265 0.8215
LymphNode: 1 0.0505 0.847 0.9825 0.808 0.7705 0.0525 0.32
Ovary: 1 0.156 0.5975 0.74 0.793 0.6995 0.1965
Prostate: 1 0.9525 0.775 0.7095 0.332 0.263
SkeletalMuscle: 1 0.894 0.8325 0.3745 0.23
Testes: 1 0.936 0.5575 0.7955
Thyroid: 1 0.638 0.259
WhiteBloodCells: 1 0.3325
EdomStromalCell: 1
Placenta Adipose Adrenal Brain Breast Colon Heart Kidney Liver Lung LymphNode Ovary Prostate SkeletalMuscle Testes Thyroid WhiteBloodCells EdomStromalCell

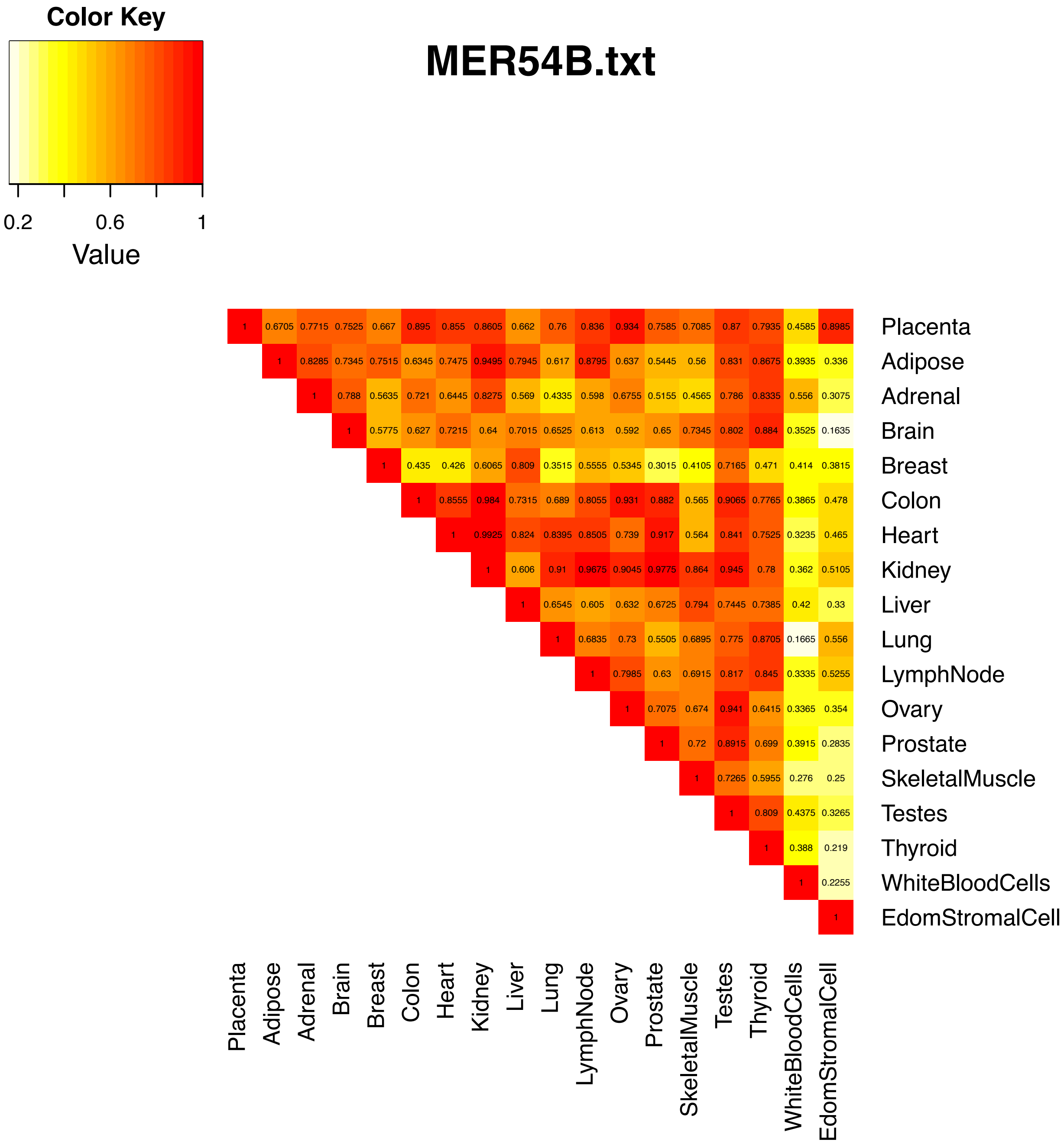
Color Key
0.2
0.6
1
Value
MER54B.txt
Placenta: 1 0.6705 0.7715 0.7525 0.667 0.895 0.855 0.8605 0.662 0.76 0.836 0.934 0.7585 0.7085 0.87 0.7935 0.4585 0.8985
Adipose: 1 0.8285 0.7345 0.7515 0.6345 0.7475 0.9495 0.7945 0.617 0.8795 0.637 0.5445 0.56 0.831 0.8675 0.3935 0.336
Adrenal: 1 0.788 0.5635 0.721 0.6445 0.8275 0.569 0.4335 0.598 0.6755 0.5155 0.4565 0.786 0.8335 0.556 0.3075
Brain: 1 0.5775 0.627 0.7215 0.64 0.7015 0.6525 0.613 0.592 0.65 0.7345 0.802 0.884 0.3525 0.1635
Breast: 1 0.435 0.426 0.6065 0.809 0.3515 0.5555 0.5345 0.3015 0.4105 0.7165 0.471 0.414 0.3815
Colon: 1 0.8555 0.984 0.7315 0.689 0.8055 0.931 0.882 0.565 0.9065 0.7765 0.3865 0.478
Heart: 1 0.9925 0.824 0.8395 0.8505 0.739 0.917 0.564 0.841 0.7525 0.3235 0.465
Kidney: 1 0.606 0.91 0.9675 0.9045 0.9775 0.864 0.945 0.78 0.362 0.5105
Liver: 1 0.6545 0.605 0.632 0.6725 0.794 0.7445 0.7385 0.42 0.33
Lung: 1 0.6835 0.73 0.5505 0.6895 0.775 0.8705 0.1665 0.556
LymphNode: 1 0.7985 0.63 0.6915 0.817 0.845 0.3335 0.5255
Ovary: 1 0.7075 0.674 0.941 0.6415 0.3365 0.354
Prostate: 1 0.72 0.8915 0.699 0.3915 0.2835
SkeletalMuscle: 1 0.7265 0.5955 0.276 0.25
Testes: 1 0.809 0.4375 0.3265
Thyroid: 1 0.388 0.219
WhiteBloodCells: 1 0.2255
EdomStromalCell: 1
Placenta Adipose Adrenal Brain Breast Colon Heart Kidney Liver Lung LymphNode Ovary Prostate SkeletalMuscle Testes Thyroid WhiteBloodCells EdomStromalCell

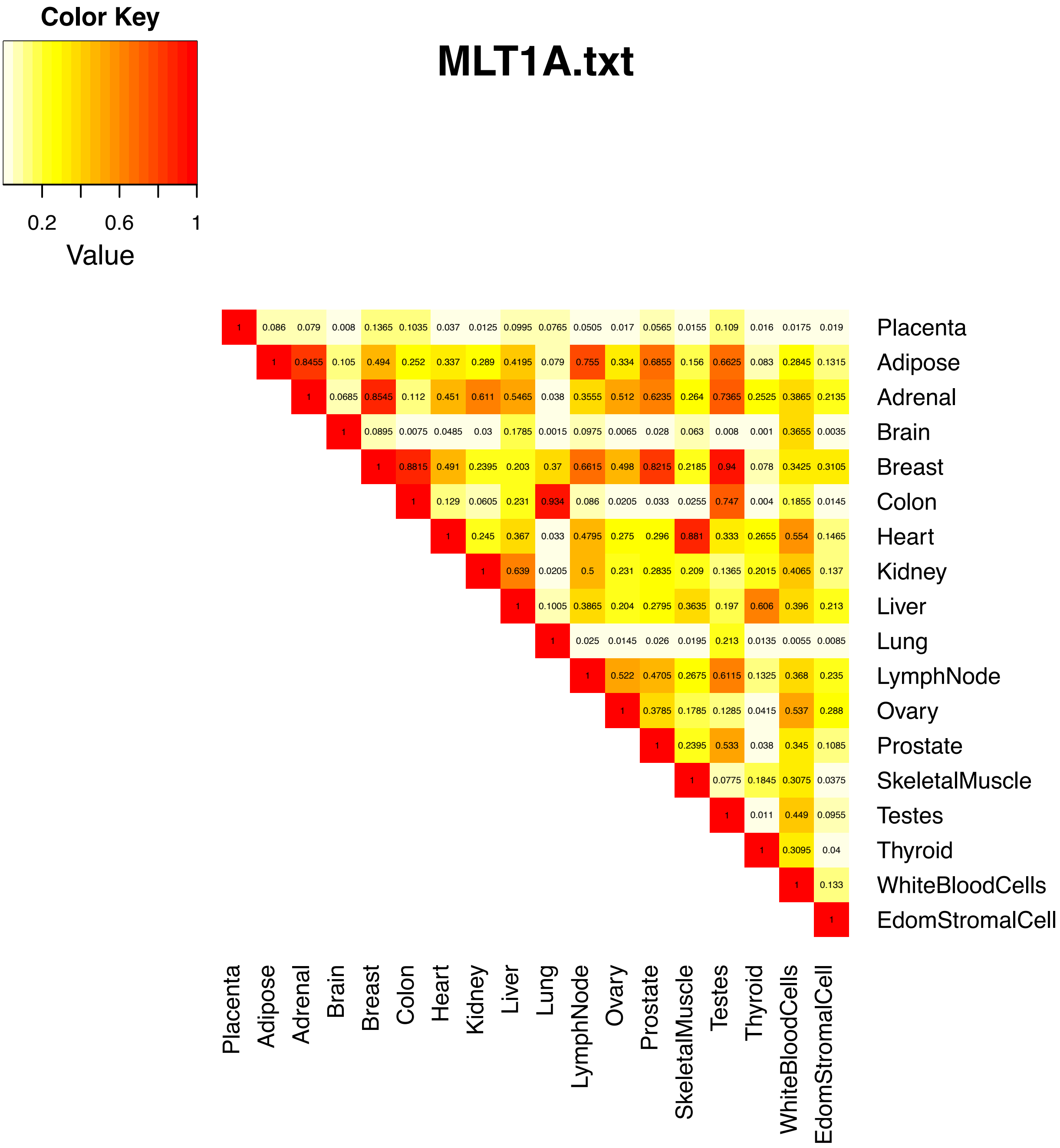
Color Key
0.2
0.6
1
Value
MLT1A.txt
Placenta: 1 0.086 0.079 0.008 0.1365 0.1035 0.037 0.0125 0.0995 0.0765 0.0505 0.017 0.0565 0.0155 0.109 0.016 0.0175 0.019
Adipose: 1 0.8455 0.105 0.494 0.252 0.337 0.289 0.4195 0.079 0.755 0.334 0.6855 0.156 0.6625 0.083 0.2845 0.1315
Adrenal: 1 0.0685 0.8545 0.112 0.451 0.611 0.5465 0.038 0.3555 0.512 0.6235 0.264 0.7365 0.2525 0.3865 0.2135
Brain: 1 0.0895 0.0075 0.0485 0.03 0.1785 0.0015 0.0975 0.0065 0.028 0.063 0.008 0.001 0.3655 0.0035
Breast: 1 0.8815 0.491 0.2395 0.203 0.37 0.6615 0.498 0.8215 0.2185 0.94 0.078 0.3425 0.3105
Colon: 1 0.129 0.0605 0.231 0.934 0.086 0.0205 0.033 0.0255 0.747 0.004 0.1855 0.0145
Heart: 1 0.245 0.367 0.033 0.4795 0.275 0.296 0.881 0.333 0.2655 0.554 0.1465
Kidney: 1 0.639 0.0205 0.5 0.231 0.2835 0.209 0.1365 0.2015 0.4065 0.137
Liver: 1 0.1005 0.3865 0.204 0.2795 0.3635 0.197 0.606 0.396 0.213
Lung: 1 0.025 0.0145 0.026 0.0195 0.213 0.0135 0.0055 0.0085
LymphNode: 1 0.522 0.4705 0.2675 0.6115 0.1325 0.368 0.235
Ovary: 1 0.3785 0.1785 0.1285 0.0415 0.537 0.288
Prostate: 1 0.2395 0.533 0.038 0.345 0.1085
SkeletalMuscle: 1 0.0775 0.1845 0.3075 0.0375
Testes: 1 0.011 0.449 0.0955
Thyroid: 1 0.3095 0.04
WhiteBloodCells: 1 0.133
EdomStromalCell: 1
Placenta Adipose Adrenal Brain Breast Colon Heart Kidney Liver Lung LymphNode Ovary Prostate SkeletalMuscle Testes Thyroid WhiteBloodCells EdomStromalCell

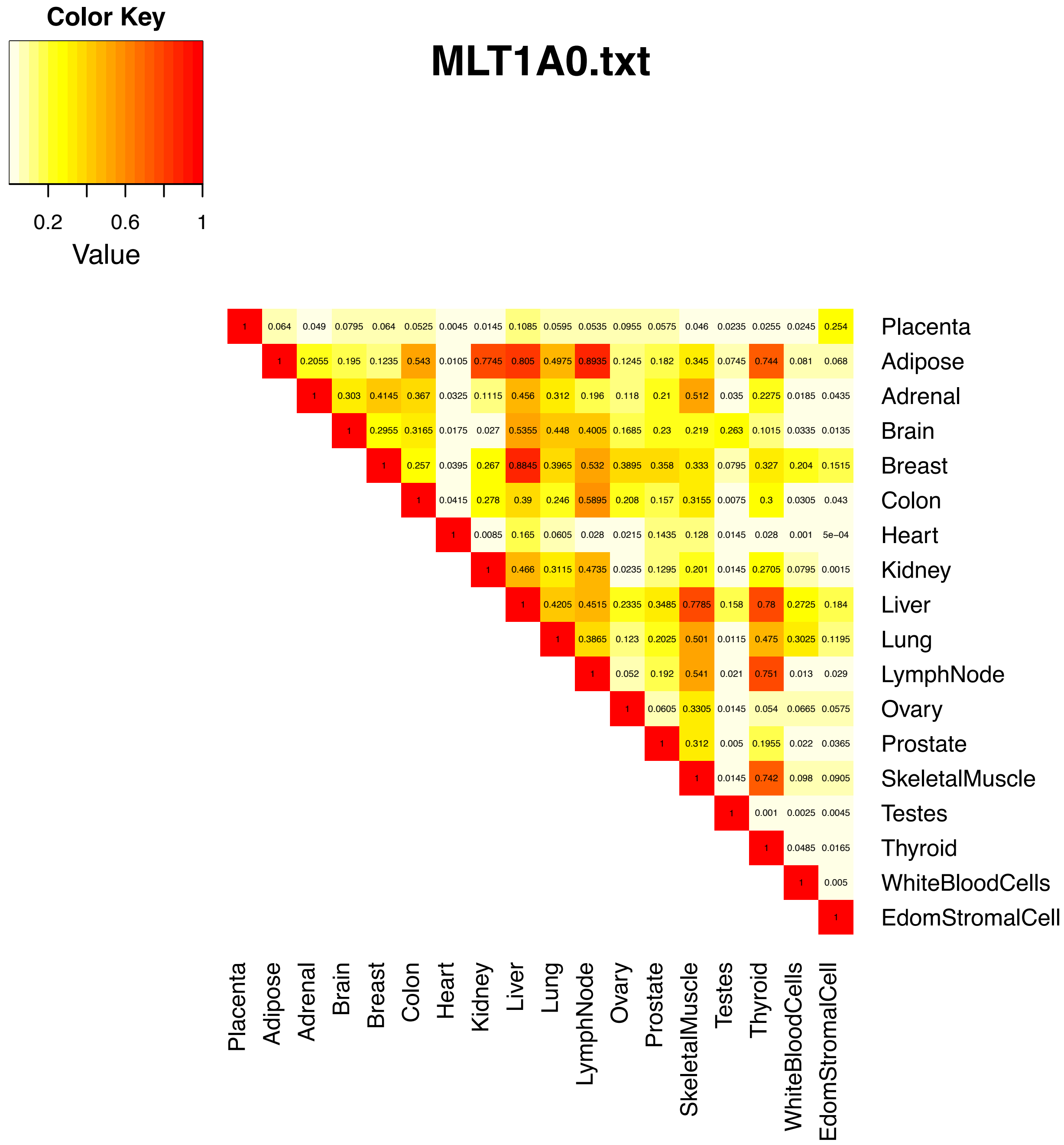
Color Key
0.2
0.6
1
Value
MLT1A0.txt
Placenta
Adipose
Adrenal
Brain
Breast
Colon
Heart
Kidney
Liver
Lung
LymphNode
Ovary
Prostate
SkeletalMuscle
Testes
Thyroid
WhiteBloodCells
EdomStromalCell
1 0.064 0.049 0.0795 0.064 0.0525 0.0045 0.0145 0.1085 0.0595 0.0535 0.0955 0.0575 0.046 0.0235 0.0255 0.0245 0.254
1 0.2055 0.195 0.1235 0.543 0.0105 0.7745 0.805 0.4975 0.8935 0.1245 0.182 0.345 0.0745 0.744 0.081 0.068
1 0.303 0.4145 0.367 0.0325 0.1115 0.456 0.312 0.196 0.118 0.21 0.512 0.035 0.2275 0.0185 0.0435
1 0.2955 0.3165 0.0175 0.027 0.5355 0.448 0.4005 0.1685 0.23 0.219 0.263 0.1015 0.0335 0.0135
1 0.257 0.0395 0.267 0.8845 0.3965 0.532 0.3895 0.358 0.333 0.0795 0.327 0.204 0.1515
1 0.0415 0.278 0.39 0.246 0.5895 0.208 0.157 0.3155 0.0075 0.3 0.0305 0.043
1 0.0085 0.165 0.0605 0.028 0.0215 0.1435 0.128 0.0145 0.028 0.001 5e−04
1 0.466 0.3115 0.4735 0.0235 0.1295 0.201 0.0145 0.2705 0.0795 0.0015
1 0.4205 0.4515 0.2335 0.3485 0.7785 0.158 0.78 0.2725 0.184
1 0.3865 0.123 0.2025 0.501 0.0115 0.475 0.3025 0.1195
1 0.052 0.192 0.541 0.021 0.751 0.013 0.029
1 0.0605 0.3305 0.0145 0.054 0.0665 0.0575
1 0.312 0.005 0.1955 0.022 0.0365
1 0.0145 0.742 0.098 0.0905
1 0.001 0.0025 0.0045
1 0.0485 0.0165
1 0.005
1
Placenta
Adipose
Adrenal
Brain
Breast
Colon
Heart
Kidney
Liver
Lung
LymphNode
Ovary
Prostate
SkeletalMuscle
Testes
Thyroid
WhiteBloodCells
EdomStromalCell

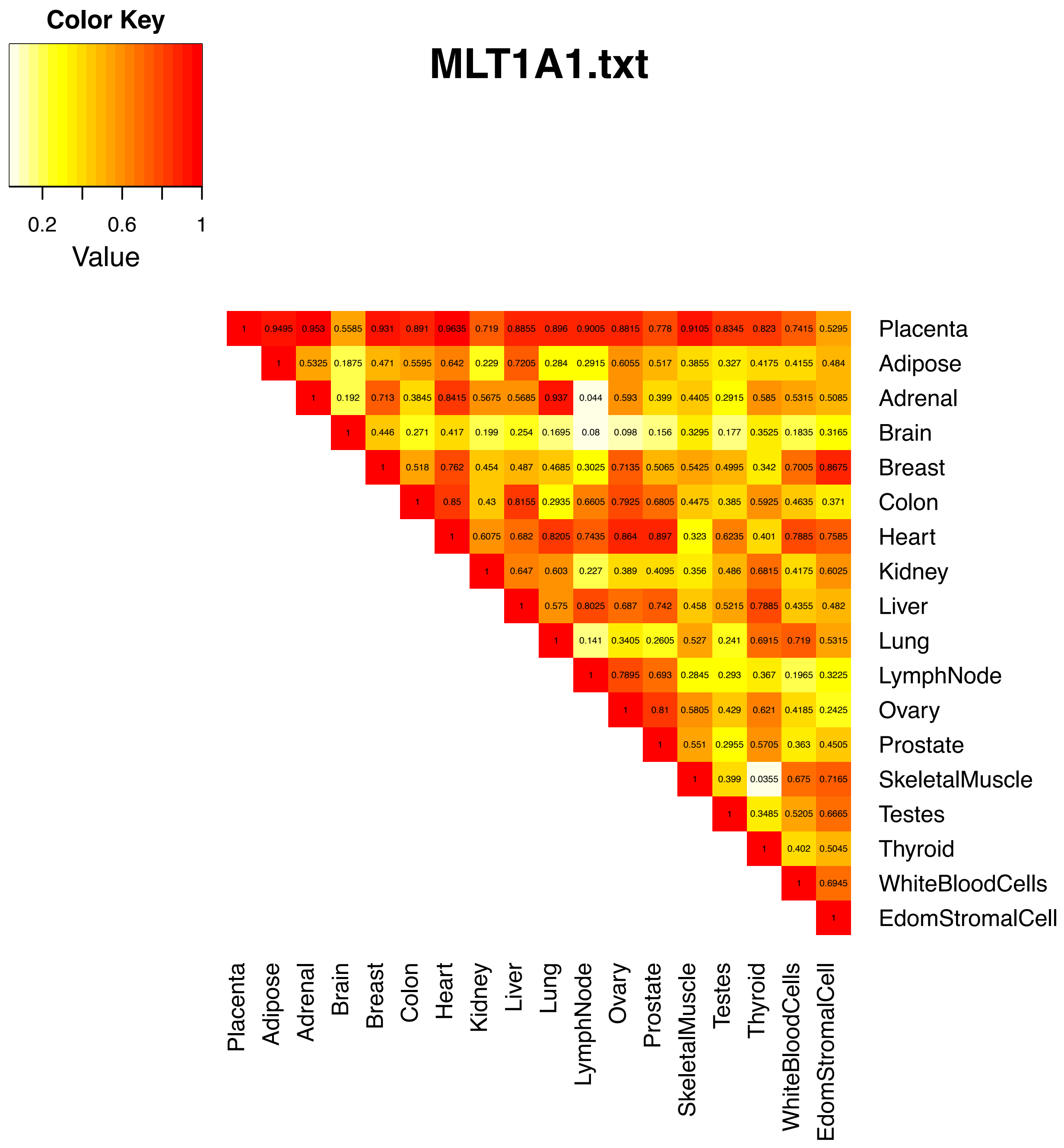

Color Key
0.2
0.6
1
Value
MLT1A1.txt
Placenta 1 0.9495 0.953 0.5585 0.931 0.891 0.9635 0.719 0.8855 0.896 0.9005 0.8815 0.778 0.9105 0.8345 0.823 0.7415 0.5295
Adipose 1 0.5325 0.1875 0.471 0.5595 0.642 0.229 0.7205 0.284 0.2915 0.6055 0.517 0.3855 0.327 0.4175 0.4155 0.484
Adrenal 1 0.192 0.713 0.3845 0.8415 0.5675 0.5685 0.937 0.044 0.593 0.399 0.4405 0.2915 0.585 0.5315 0.5085
Brain 1 0.446 0.271 0.417 0.199 0.254 0.1695 0.08 0.098 0.156 0.3295 0.177 0.3525 0.1835 0.3165
Breast 1 0.518 0.762 0.454 0.487 0.4685 0.3025 0.7135 0.5065 0.5425 0.4995 0.342 0.7005 0.8675
Colon 1 0.85 0.43 0.8155 0.2935 0.6605 0.7925 0.6805 0.4475 0.385 0.5925 0.4635 0.371
Heart 1 0.6075 0.682 0.8205 0.7435 0.864 0.897 0.323 0.6235 0.401 0.7885 0.7585
Kidney 1 0.647 0.603 0.227 0.389 0.4095 0.356 0.486 0.6815 0.4175 0.6025
Liver 1 0.575 0.8025 0.687 0.742 0.458 0.5215 0.7885 0.4355 0.482
Lung 1 0.141 0.3405 0.2605 0.527 0.241 0.6915 0.719 0.5315
LymphNode 1 0.7895 0.693 0.2845 0.293 0.367 0.1965 0.3225
Ovary 1 0.81 0.5805 0.429 0.621 0.4185 0.2425
Prostate 1 0.551 0.2955 0.5705 0.363 0.4505
SkeletalMuscle 1 0.399 0.0355 0.675 0.7165
Testes 1 0.3485 0.5205 0.6665
Thyroid 1 0.402 0.5045
WhiteBloodCells 1 0.6945
EdomStromalCell 1
Placenta
Adipose
Adrenal
Brain
Breast
Colon
Heart
Kidney
Liver
Lung
LymphNode
Ovary
Prostate
SkeletalMuscle
Testes
Thyroid
WhiteBloodCells
EdomStromalCell

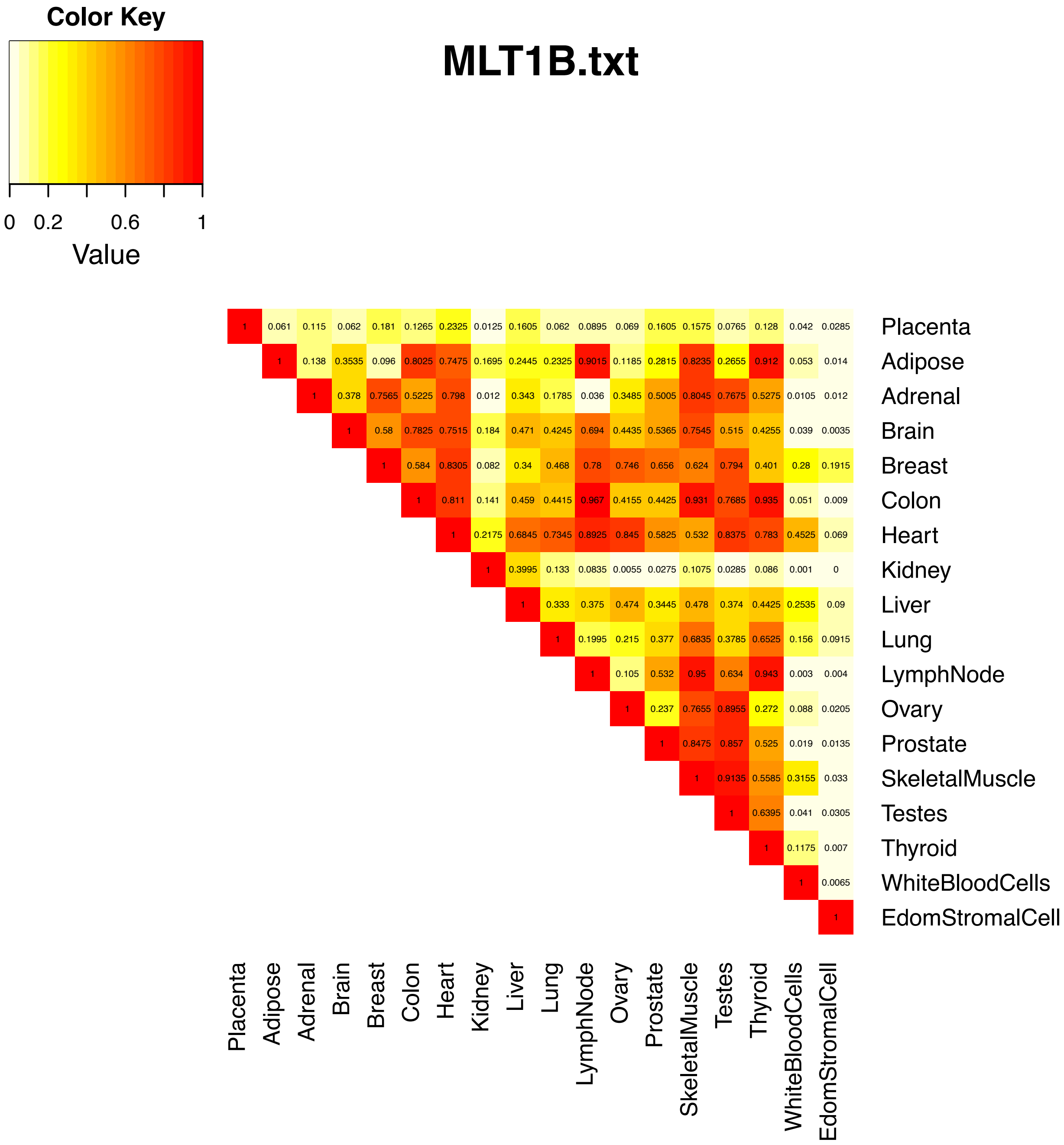
Color Key
0 0.2 0.6 1
Value
MLT1B.txt
Placenta 1 0.061 0.115 0.062 0.181 0.1265 0.2325 0.0125 0.1605 0.062 0.0895 0.069 0.1605 0.1575 0.0765 0.128 0.042 0.0285
Adipose 1 0.138 0.3535 0.096 0.8025 0.7475 0.1695 0.2445 0.2325 0.9015 0.1185 0.2815 0.8235 0.2655 0.912 0.053 0.014
Adrenal 1 0.378 0.7565 0.5225 0.798 0.012 0.343 0.1785 0.036 0.3485 0.5005 0.8045 0.7675 0.5275 0.0105 0.012
Brain 1 0.58 0.7825 0.7515 0.184 0.471 0.4245 0.694 0.4435 0.5365 0.7545 0.515 0.4255 0.039 0.0035
Breast 1 0.584 0.8305 0.082 0.34 0.468 0.78 0.746 0.656 0.624 0.794 0.401 0.28 0.1915
Colon 1 0.811 0.141 0.459 0.4415 0.967 0.4155 0.4425 0.931 0.7685 0.935 0.051 0.009
Heart 1 0.2175 0.6845 0.7345 0.8925 0.845 0.5825 0.532 0.8375 0.783 0.4525 0.069
Kidney 1 0.3995 0.133 0.0835 0.0055 0.0275 0.1075 0.0285 0.086 0.001 0
Liver 1 0.333 0.375 0.474 0.3445 0.478 0.374 0.4425 0.2535 0.09
Lung 1 0.1995 0.215 0.377 0.6835 0.3785 0.6525 0.156 0.0915
LymphNode 1 0.105 0.532 0.95 0.634 0.943 0.003 0.004
Ovary 1 0.237 0.7655 0.8955 0.272 0.088 0.0205
Prostate 1 0.8475 0.857 0.525 0.019 0.0135
SkeletalMuscle 1 0.9135 0.5585 0.3155 0.033
Testes 1 0.6395 0.041 0.0305
Thyroid 1 0.1175 0.007
WhiteBloodCells 1 0.0065
EdomStromalCell 1
Placenta Adipose Adrenal Brain Breast Colon Heart Kidney Liver Lung LymphNode Ovary Prostate SkeletalMuscle Testes Thyroid WhiteBloodCells EdomStromalCell

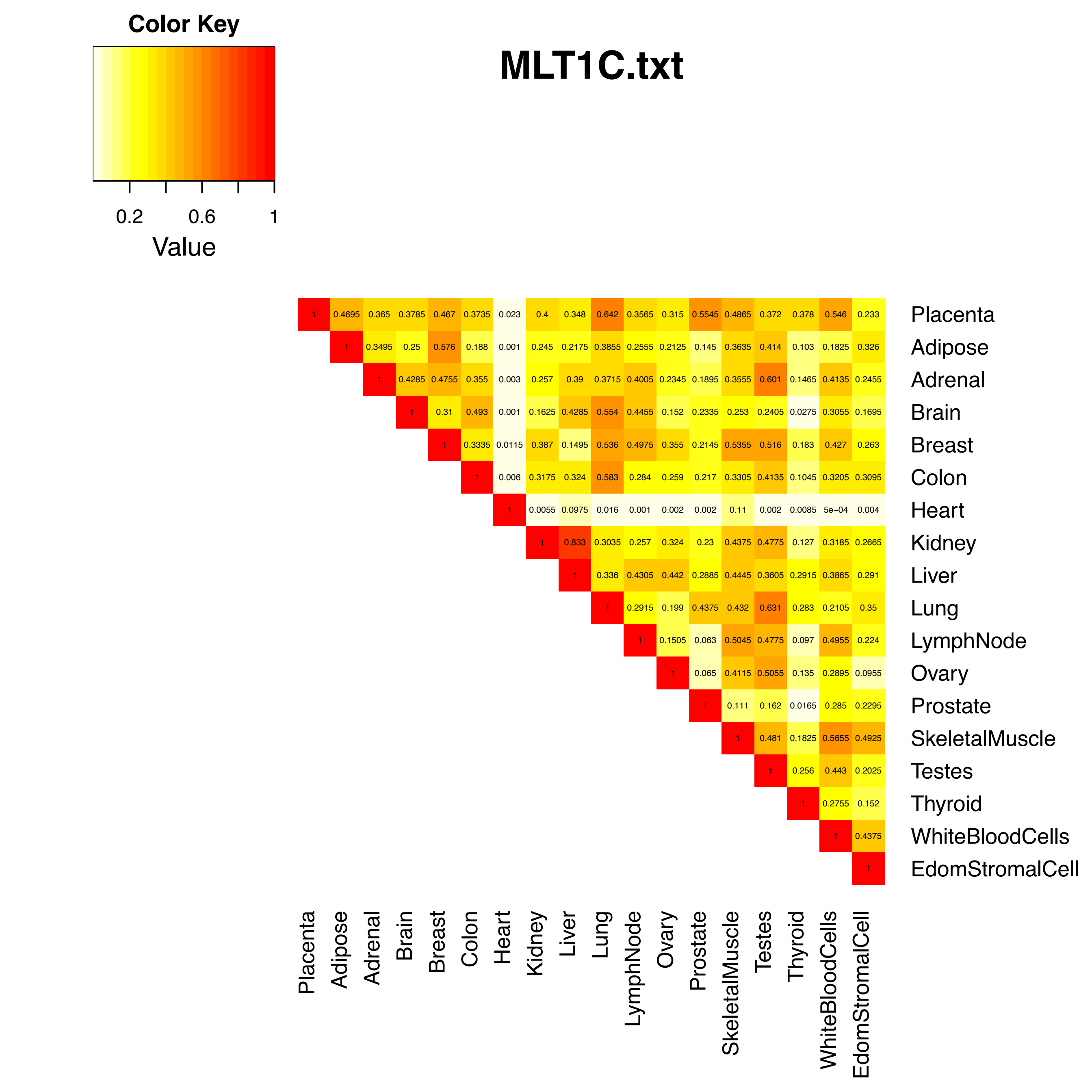
Color Key
0.2
0.6
1
Value
MLT1C.txt
Placenta 1 0.4695 0.365 0.3785 0.467 0.3735 0.023 0.4 0.348 0.642 0.3565 0.315 0.5545 0.4865 0.372 0.378 0.546 0.233
Adipose 1 0.3495 0.25 0.576 0.188 0.001 0.245 0.2175 0.3855 0.2555 0.2125 0.145 0.3635 0.414 0.103 0.1825 0.326
Adrenal 1 0.4285 0.4755 0.355 0.003 0.257 0.39 0.3715 0.4005 0.2345 0.1895 0.3555 0.601 0.1465 0.4135 0.2455
Brain 1 0.31 0.493 0.001 0.1625 0.4285 0.554 0.4455 0.152 0.2335 0.253 0.2405 0.0275 0.3055 0.1695
Breast 1 0.3335 0.0115 0.387 0.1495 0.536 0.4975 0.355 0.2145 0.5355 0.516 0.183 0.427 0.263
Colon 1 0.006 0.3175 0.324 0.583 0.284 0.259 0.217 0.3305 0.4135 0.1045 0.3205 0.3095
Heart 1 0.0055 0.0975 0.016 0.001 0.002 0.002 0.11 0.002 0.0085 5e−04 0.004
Kidney 1 0.833 0.3035 0.257 0.324 0.23 0.4375 0.4775 0.127 0.3185 0.2665
Liver 1 0.336 0.4305 0.442 0.2885 0.4445 0.3605 0.2915 0.3865 0.291
Lung 1 0.2915 0.199 0.4375 0.432 0.631 0.283 0.2105 0.35
LymphNode 1 0.1505 0.063 0.5045 0.4775 0.097 0.4955 0.224
Ovary 1 0.065 0.4115 0.5055 0.135 0.2895 0.0955
Prostate 1 0.111 0.162 0.0165 0.285 0.2295
SkeletalMuscle 1 0.481 0.1825 0.5655 0.4925
Testes 1 0.256 0.443 0.2025
Thyroid 1 0.2755 0.152
WhiteBloodCells 1 0.4375
EdomStromalCell 1
Placenta Adipose Adrenal Brain Breast Colon Heart Kidney Liver Lung LymphNode Ovary Prostate SkeletalMuscle Testes Thyroid WhiteBloodCells EdomStromalCell

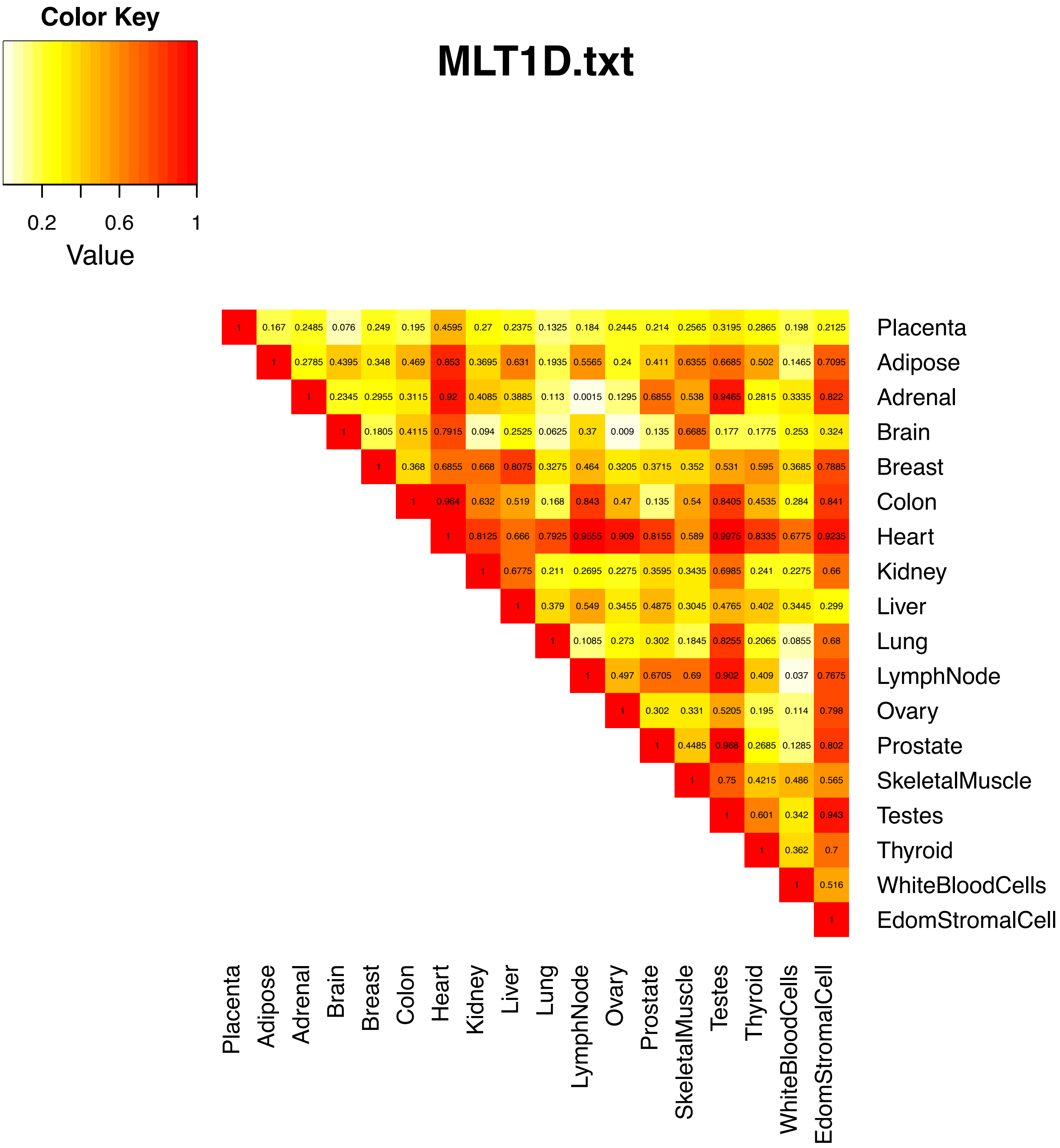
Color Key
0.2
0.6
1
Value
MLT1D.txt
Placenta 1 0.167 0.2485 0.076 0.249 0.195 0.4595 0.27 0.2375 0.1325 0.184 0.2445 0.214 0.2565 0.3195 0.2865 0.198 0.2125
Adipose 1 0.2785 0.4395 0.348 0.469 0.853 0.3695 0.631 0.1935 0.5565 0.24 0.411 0.6355 0.6685 0.502 0.1465 0.7095
Adrenal 1 0.2345 0.2955 0.3115 0.92 0.4085 0.3885 0.113 0.0015 0.1295 0.6855 0.538 0.9465 0.2815 0.3335 0.822
Brain 1 0.1805 0.4115 0.7915 0.094 0.2525 0.0625 0.37 0.009 0.135 0.6685 0.177 0.1775 0.253 0.324
Breast 1 0.368 0.6855 0.668 0.8075 0.3275 0.464 0.3205 0.3715 0.352 0.531 0.595 0.3685 0.7885
Colon 1 0.964 0.632 0.519 0.168 0.843 0.47 0.135 0.54 0.8405 0.4535 0.284 0.841
Heart 1 0.8125 0.666 0.7925 0.9555 0.909 0.8155 0.589 0.9975 0.8335 0.6775 0.9235
Kidney 1 0.6775 0.211 0.2695 0.2275 0.3595 0.3435 0.6985 0.241 0.2275 0.66
Liver 1 0.379 0.549 0.3455 0.4875 0.3045 0.4765 0.402 0.3445 0.299
Lung 1 0.1085 0.273 0.302 0.1845 0.8255 0.2065 0.0855 0.68
LymphNode 1 0.497 0.6705 0.69 0.902 0.409 0.037 0.7675
Ovary 1 0.302 0.331 0.5205 0.195 0.114 0.798
Prostate 1 0.4485 0.968 0.2685 0.1285 0.802
SkeletalMuscle 1 0.75 0.4215 0.486 0.565
Testes 1 0.601 0.342 0.943
Thyroid 1 0.362 0.7
WhiteBloodCells 1 0.516
EdomStromalCell 1
Placenta
Adipose
Adrenal
Brain
Breast
Colon
Heart
Kidney
Liver
Lung
LymphNode
Ovary
Prostate
SkeletalMuscle
Testes
Thyroid
WhiteBloodCells
EdomStromalCell

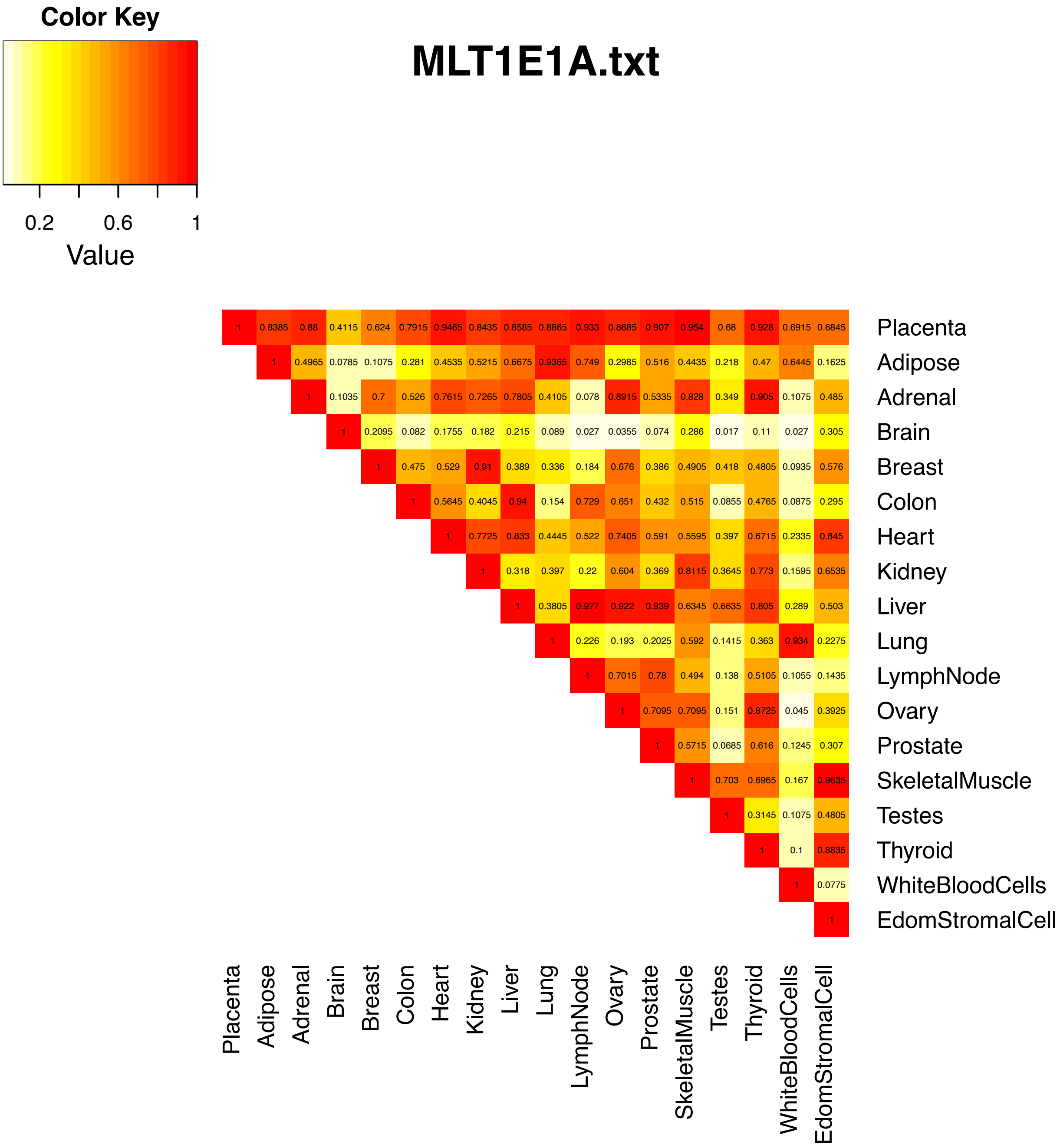
Color Key
0.2
0.6
1
Value
MLT1E1A.txt
Placenta 1 0.8385 0.88 0.4115 0.624 0.7915 0.9465 0.8435 0.8585 0.8865 0.933 0.8685 0.907 0.954 0.68 0.928 0.6915 0.6845
Adipose 1 0.4965 0.0785 0.1075 0.281 0.4535 0.5215 0.6675 0.9365 0.749 0.2985 0.516 0.4435 0.218 0.47 0.6445 0.1625
Adrenal 1 0.1035 0.7 0.526 0.7615 0.7265 0.7805 0.4105 0.078 0.8915 0.5335 0.828 0.349 0.905 0.1075 0.485
Brain 1 0.2095 0.082 0.1755 0.182 0.215 0.089 0.027 0.0355 0.074 0.286 0.017 0.11 0.027 0.305
Breast 1 0.475 0.529 0.91 0.389 0.336 0.184 0.676 0.386 0.4905 0.418 0.4805 0.0935 0.576
Colon 1 0.5645 0.4045 0.94 0.154 0.729 0.651 0.432 0.515 0.0855 0.4765 0.0875 0.295
Heart 1 0.7725 0.833 0.4445 0.522 0.7405 0.591 0.5595 0.397 0.6715 0.2335 0.845
Kidney 1 0.318 0.397 0.22 0.604 0.369 0.8115 0.3645 0.773 0.1595 0.6535
Liver 1 0.3805 0.977 0.922 0.939 0.6345 0.6635 0.805 0.289 0.503
Lung 1 0.226 0.193 0.2025 0.592 0.1415 0.363 0.934 0.2275
LymphNode 1 0.7015 0.78 0.494 0.138 0.5105 0.1055 0.1435
Ovary 1 0.7095 0.7095 0.151 0.8725 0.045 0.3925
Prostate 1 0.5715 0.0685 0.616 0.1245 0.307
SkeletalMuscle 1 0.703 0.6965 0.167 0.9635
Testes 1 0.3145 0.1075 0.4805
Thyroid 1 0.1 0.8835
WhiteBloodCells 1 0.0775
EdomStromalCell 1
Placenta
Adipose
Adrenal
Brain
Breast
Colon
Heart
Kidney
Liver
Lung
LymphNode
Ovary
Prostate
SkeletalMuscle
Testes
Thyroid
WhiteBloodCells
EdomStromalCell

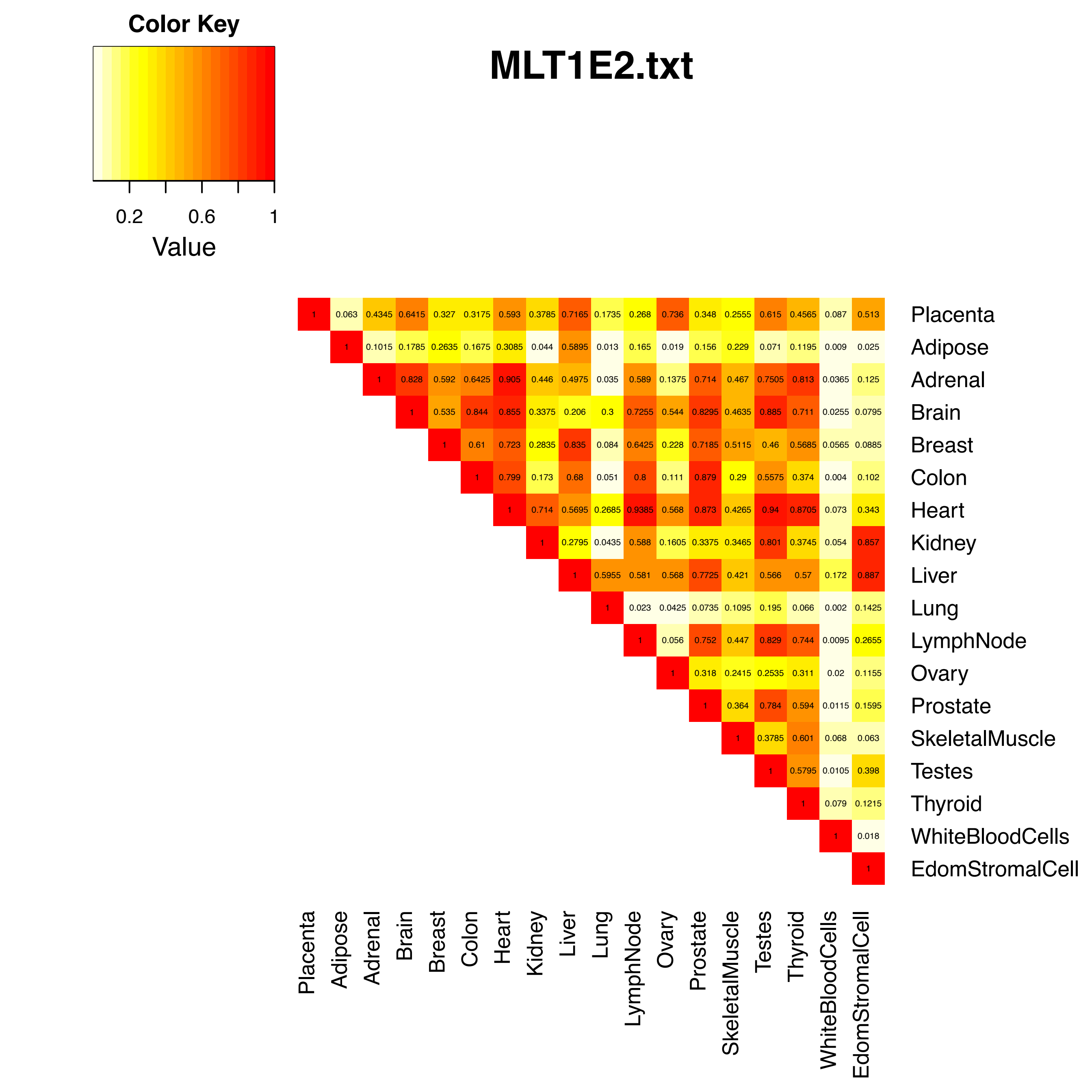
Color Key
0.2
0.6
1
Value
MLT1E2.txt
Placenta: 1 0.063 0.4345 0.6415 0.327 0.3175 0.593 0.3785 0.7165 0.1735 0.268 0.736 0.348 0.2555 0.615 0.4565 0.087 0.513
Adipose: 1 0.1015 0.1785 0.2635 0.1675 0.3085 0.044 0.5895 0.013 0.165 0.019 0.156 0.229 0.071 0.1195 0.009 0.025
Adrenal: 1 0.828 0.592 0.6425 0.905 0.446 0.4975 0.035 0.589 0.1375 0.714 0.467 0.7505 0.813 0.0365 0.125
Brain: 1 0.535 0.844 0.855 0.3375 0.206 0.3 0.7255 0.544 0.8295 0.4635 0.885 0.711 0.0255 0.0795
Breast: 1 0.61 0.723 0.2835 0.835 0.084 0.6425 0.228 0.7185 0.5115 0.46 0.5685 0.0565 0.0885
Colon: 1 0.799 0.173 0.68 0.051 0.8 0.111 0.879 0.29 0.5575 0.374 0.004 0.102
Heart: 1 0.714 0.5695 0.2685 0.9385 0.568 0.873 0.4265 0.94 0.8705 0.073 0.343
Kidney: 1 0.2795 0.0435 0.588 0.1605 0.3375 0.3465 0.801 0.3745 0.054 0.857
Liver: 1 0.5955 0.581 0.568 0.7725 0.421 0.566 0.57 0.172 0.887
Lung: 1 0.023 0.0425 0.0735 0.1095 0.195 0.066 0.002 0.1425
LymphNode: 1 0.056 0.752 0.447 0.829 0.744 0.0095 0.2655
Ovary: 1 0.318 0.2415 0.2535 0.311 0.02 0.1155
Prostate: 1 0.364 0.784 0.594 0.0115 0.1595
SkeletalMuscle: 1 0.3785 0.601 0.068 0.063
Testes: 1 0.5795 0.0105 0.398
Thyroid: 1 0.079 0.1215
WhiteBloodCells: 1 0.018
EdomStromalCell: 1
Placenta
Adipose
Adrenal
Brain
Breast
Colon
Heart
Kidney
Liver
Lung
LymphNode
Ovary
Prostate
SkeletalMuscle
Testes
Thyroid
WhiteBloodCells
EdomStromalCell

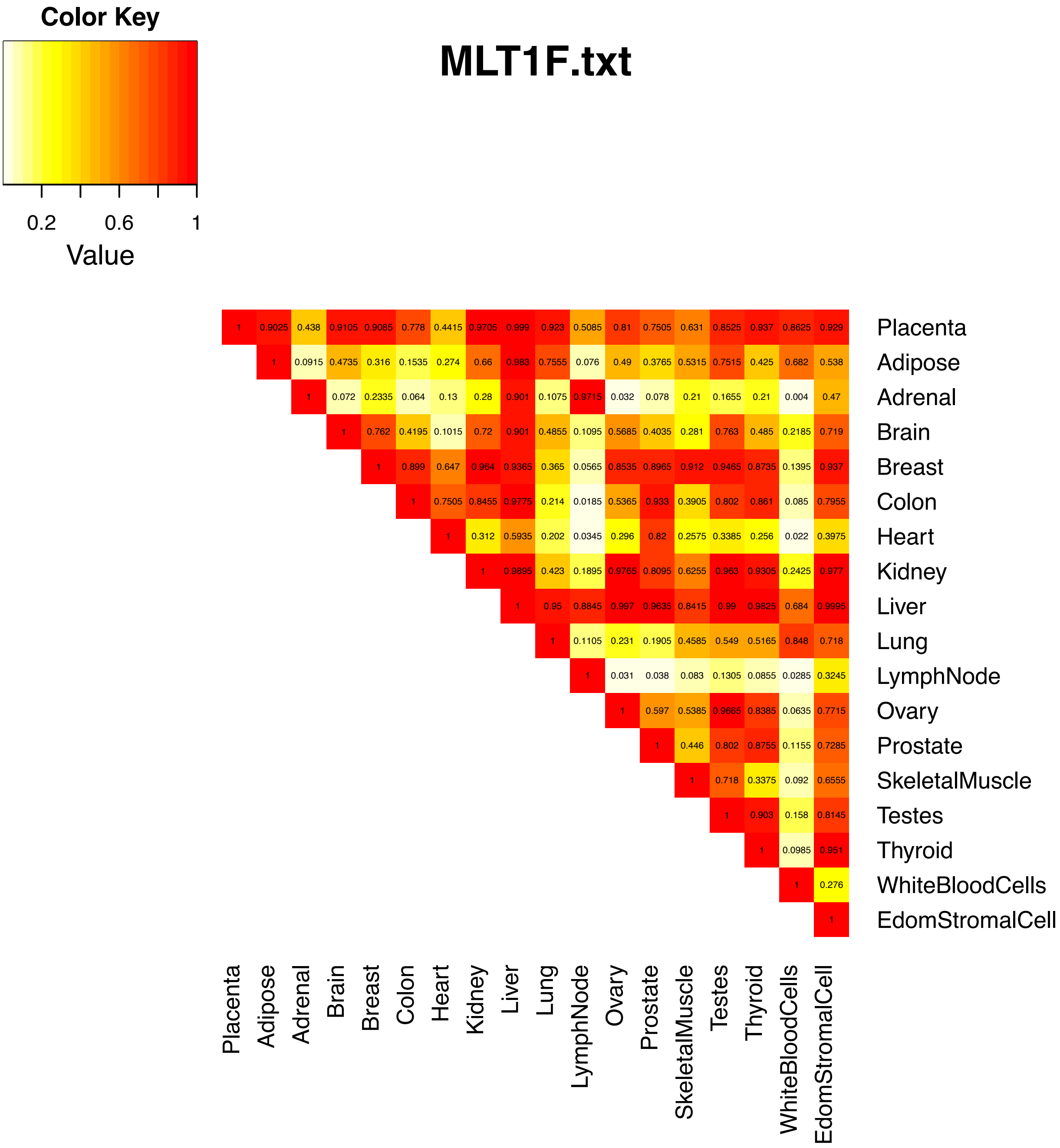
Color Key
0.2
0.6
1
Value
MLT1F.txt
Placenta: 1 0.9025 0.438 0.9105 0.9085 0.778 0.4415 0.9705 0.999 0.923 0.5085 0.81 0.7505 0.631 0.8525 0.937 0.8625 0.929
Adipose: 1 0.0915 0.4735 0.316 0.1535 0.274 0.66 0.983 0.7555 0.076 0.49 0.3765 0.5315 0.7515 0.425 0.682 0.538
Adrenal: 1 0.072 0.2335 0.064 0.13 0.28 0.901 0.1075 0.9715 0.032 0.078 0.21 0.1655 0.21 0.004 0.47
Brain: 1 0.762 0.4195 0.1015 0.72 0.901 0.4855 0.1095 0.5685 0.4035 0.281 0.763 0.485 0.2185 0.719
Breast: 1 0.899 0.647 0.964 0.9365 0.365 0.0565 0.8535 0.8965 0.912 0.9465 0.8735 0.1395 0.937
Colon: 1 0.7505 0.8455 0.9775 0.214 0.0185 0.5365 0.933 0.3905 0.802 0.861 0.085 0.7955
Heart: 1 0.312 0.5935 0.202 0.0345 0.296 0.82 0.2575 0.3385 0.256 0.022 0.3975
Kidney: 1 0.9895 0.423 0.1895 0.9765 0.8095 0.6255 0.963 0.9305 0.2425 0.977
Liver: 1 0.95 0.8845 0.997 0.9635 0.8415 0.99 0.9825 0.684 0.9995
Lung: 1 0.1105 0.231 0.1905 0.4585 0.549 0.5165 0.848 0.718
LymphNode: 1 0.031 0.038 0.083 0.1305 0.0855 0.0285 0.3245
Ovary: 1 0.597 0.5385 0.9665 0.8385 0.0635 0.7715
Prostate: 1 0.446 0.802 0.8755 0.1155 0.7285
SkeletalMuscle: 1 0.718 0.3375 0.092 0.6555
Testes: 1 0.903 0.158 0.8145
Thyroid: 1 0.0985 0.951
WhiteBloodCells: 1 0.276
EdomStromalCell: 1
Placenta Adipose Adrenal Brain Breast Colon Heart Kidney Liver Lung LymphNode Ovary Prostate SkeletalMuscle Testes Thyroid WhiteBloodCells EdomStromalCell

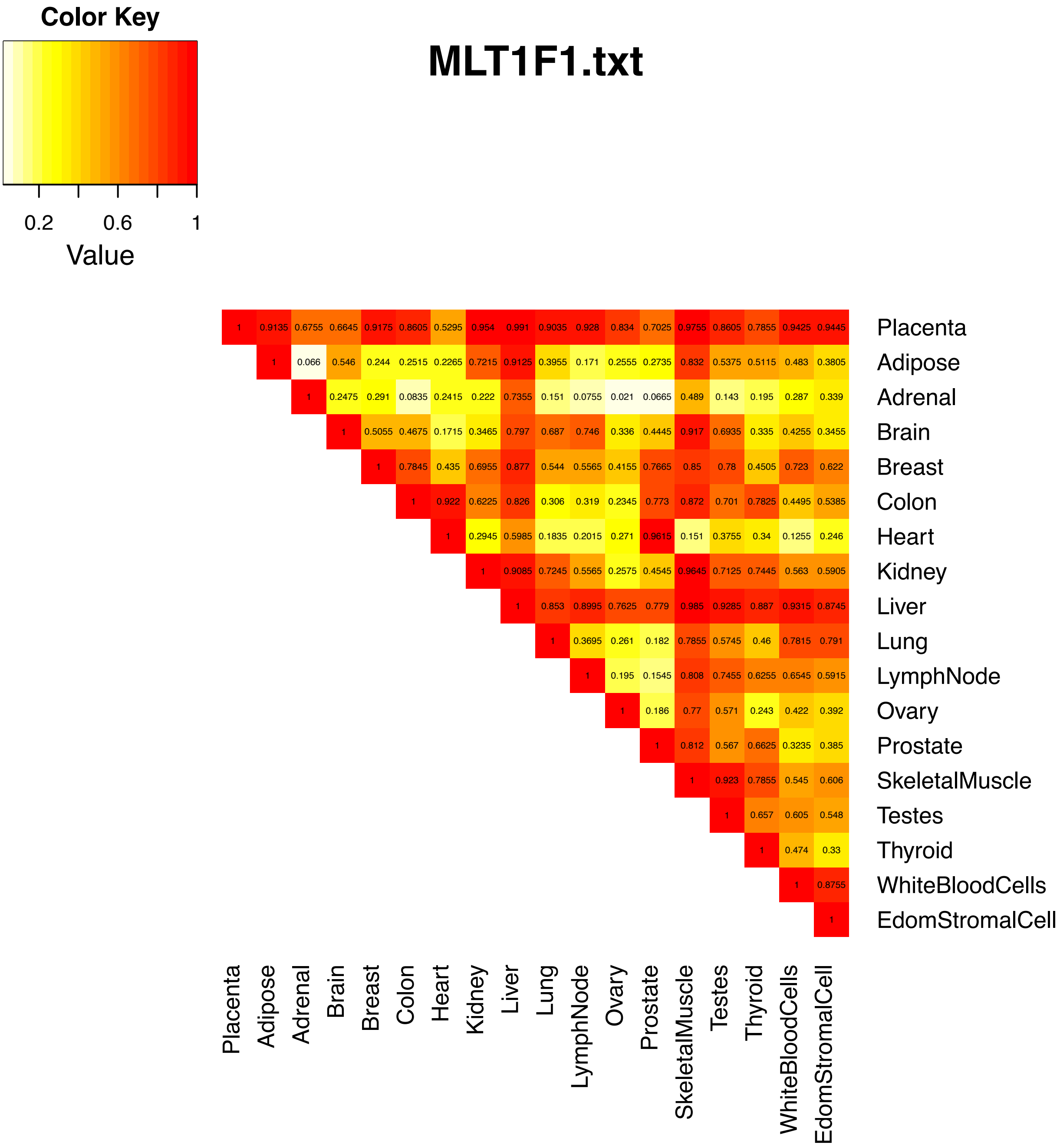
Color Key
0.2
0.6
1
Value
MLT1F1.txt
Placenta
Adipose
Adrenal
Brain
Breast
Colon
Heart
Kidney
Liver
Lung
LymphNode
Ovary
Prostate
SkeletalMuscle
Testes
Thyroid
WhiteBloodCells
EdomStromalCell
1 0.9135 0.6755 0.6645 0.9175 0.8605 0.5295 0.954 0.991 0.9035 0.928 0.834 0.7025 0.9755 0.8605 0.7855 0.9425 0.9445
1 0.066 0.546 0.244 0.2515 0.2265 0.7215 0.9125 0.3955 0.171 0.2555 0.2735 0.832 0.5375 0.5115 0.483 0.3805
1 0.2475 0.291 0.0835 0.2415 0.222 0.7355 0.151 0.0755 0.021 0.0665 0.489 0.143 0.195 0.287 0.339
1 0.5055 0.4675 0.1715 0.3465 0.797 0.687 0.746 0.336 0.4445 0.917 0.6935 0.335 0.4255 0.3455
1 0.7845 0.435 0.6955 0.877 0.544 0.5565 0.4155 0.7665 0.85 0.78 0.4505 0.723 0.622
1 0.922 0.6225 0.826 0.306 0.319 0.2345 0.773 0.872 0.701 0.7825 0.4495 0.5385
1 0.2945 0.5985 0.1835 0.2015 0.271 0.9615 0.151 0.3755 0.34 0.1255 0.246
1 0.9085 0.7245 0.5565 0.2575 0.4545 0.9645 0.7125 0.7445 0.563 0.5905
1 0.853 0.8995 0.7625 0.779 0.985 0.9285 0.887 0.9315 0.8745
1 0.3695 0.261 0.182 0.7855 0.5745 0.46 0.7815 0.791
1 0.195 0.1545 0.808 0.7455 0.6255 0.6545 0.5915
1 0.186 0.77 0.571 0.243 0.422 0.392
1 0.812 0.567 0.6625 0.3235 0.385
1 0.923 0.7855 0.545 0.606
1 0.657 0.605 0.548
1 0.474 0.33
1 0.8755
1

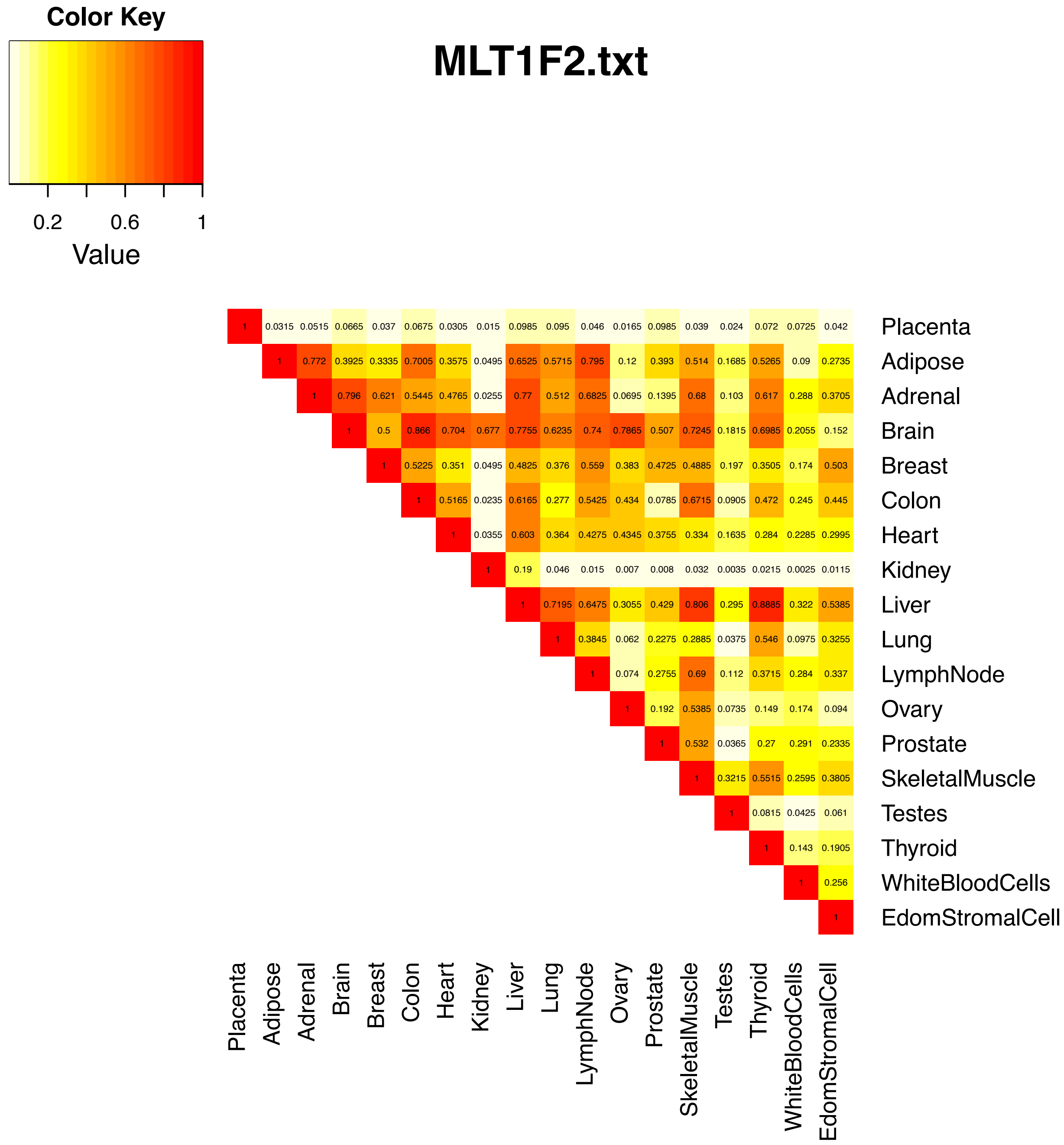

Color Key
0.2 0.6 1
Value
MLT1F2.txt
Placenta 1 0.0315 0.0515 0.0665 0.037 0.0675 0.0305 0.015 0.0985 0.095 0.046 0.0165 0.0985 0.039 0.024 0.072 0.0725 0.042
Adipose 1 0.772 0.3925 0.3335 0.7005 0.3575 0.0495 0.6525 0.5715 0.795 0.12 0.393 0.514 0.1685 0.5265 0.09 0.2735
Adrenal 1 0.796 0.621 0.5445 0.4765 0.0255 0.77 0.512 0.6825 0.0695 0.1395 0.68 0.103 0.617 0.288 0.3705
Brain 1 0.5 0.866 0.704 0.677 0.7755 0.6235 0.74 0.7865 0.507 0.7245 0.1815 0.6985 0.2055 0.152
Breast 1 0.5225 0.351 0.0495 0.4825 0.376 0.559 0.383 0.4725 0.4885 0.197 0.3505 0.174 0.503
Colon 1 0.5165 0.0235 0.6165 0.277 0.5425 0.434 0.0785 0.6715 0.0905 0.472 0.245 0.445
Heart 1 0.0355 0.603 0.364 0.4275 0.4345 0.3755 0.334 0.1635 0.284 0.2285 0.2995
Kidney 1 0.19 0.046 0.015 0.007 0.008 0.032 0.0035 0.0215 0.0025 0.0115
Liver 1 0.7195 0.6475 0.3055 0.429 0.806 0.295 0.8885 0.322 0.5385
Lung 1 0.3845 0.062 0.2275 0.2885 0.0375 0.546 0.0975 0.3255
LymphNode 1 0.074 0.2755 0.69 0.112 0.3715 0.284 0.337
Ovary 1 0.192 0.5385 0.0735 0.149 0.174 0.094
Prostate 1 0.532 0.0365 0.27 0.291 0.2335
SkeletalMuscle 1 0.3215 0.5515 0.2595 0.3805
Testes 1 0.0815 0.0425 0.061
Thyroid 1 0.143 0.1905
WhiteBloodCells 1 0.256
EdomStromalCell 1
Placenta Adipose Adrenal Brain Breast Colon Heart Kidney Liver Lung LymphNode Ovary Prostate SkeletalMuscle Testes Thyroid WhiteBloodCells EdomStromalCell

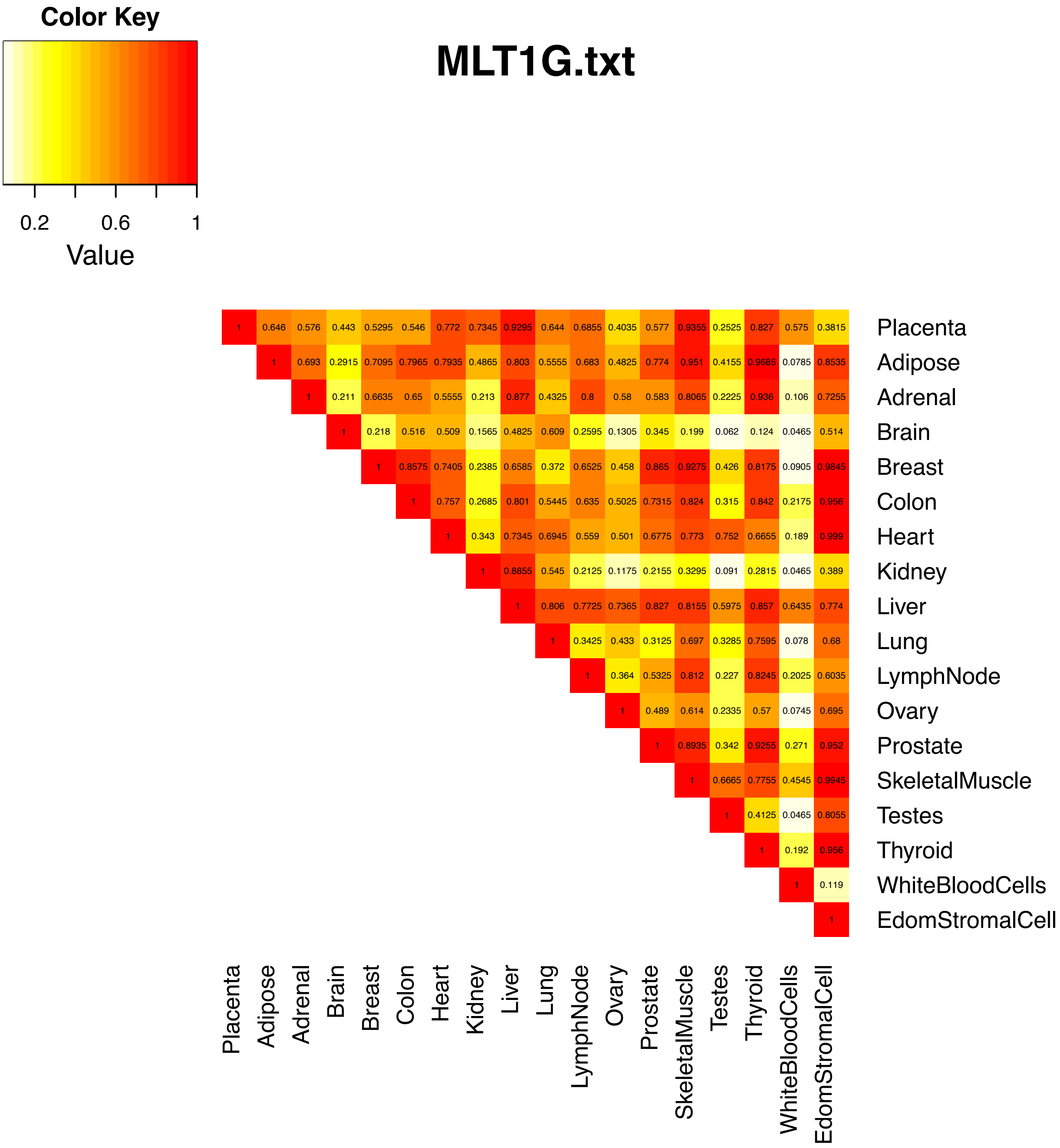
Color Key
0.2
0.6
1
Value
MLT1G.txt
Placenta: 1 0.646 0.576 0.443 0.5295 0.546 0.772 0.7345 0.9295 0.644 0.6855 0.4035 0.577 0.9355 0.2525 0.827 0.575 0.3815
Adipose: 1 0.693 0.2915 0.7095 0.7965 0.7935 0.4865 0.803 0.5555 0.683 0.4825 0.774 0.951 0.4155 0.9685 0.0785 0.8535
Adrenal: 1 0.211 0.6635 0.65 0.5555 0.213 0.877 0.4325 0.8 0.58 0.583 0.8065 0.2225 0.936 0.106 0.7255
Brain: 1 0.218 0.516 0.509 0.1565 0.4825 0.609 0.2595 0.1305 0.345 0.199 0.062 0.124 0.0465 0.514
Breast: 1 0.8575 0.7405 0.2385 0.6585 0.372 0.6525 0.458 0.865 0.9275 0.426 0.8175 0.0905 0.9845
Colon: 1 0.757 0.2685 0.801 0.5445 0.635 0.5025 0.7315 0.824 0.315 0.842 0.2175 0.956
Heart: 1 0.343 0.7345 0.6945 0.559 0.501 0.6775 0.773 0.752 0.6655 0.189 0.999
Kidney: 1 0.8855 0.545 0.2125 0.1175 0.2155 0.3295 0.091 0.2815 0.0465 0.389
Liver: 1 0.806 0.7725 0.7365 0.827 0.8155 0.5975 0.857 0.6435 0.774
Lung: 1 0.3425 0.433 0.3125 0.697 0.3285 0.7595 0.078 0.68
LymphNode: 1 0.364 0.5325 0.812 0.227 0.8245 0.2025 0.6035
Ovary: 1 0.489 0.614 0.2335 0.57 0.0745 0.695
Prostate: 1 0.8935 0.342 0.9255 0.271 0.952
SkeletalMuscle: 1 0.6665 0.7755 0.4545 0.9945
Testes: 1 0.4125 0.0465 0.8055
Thyroid: 1 0.192 0.956
WhiteBloodCells: 1 0.119
EdomStromalCell: 1
Placenta Adipose Adrenal Brain Breast Colon Heart Kidney Liver Lung LymphNode Ovary Prostate SkeletalMuscle Testes Thyroid WhiteBloodCells EdomStromalCell

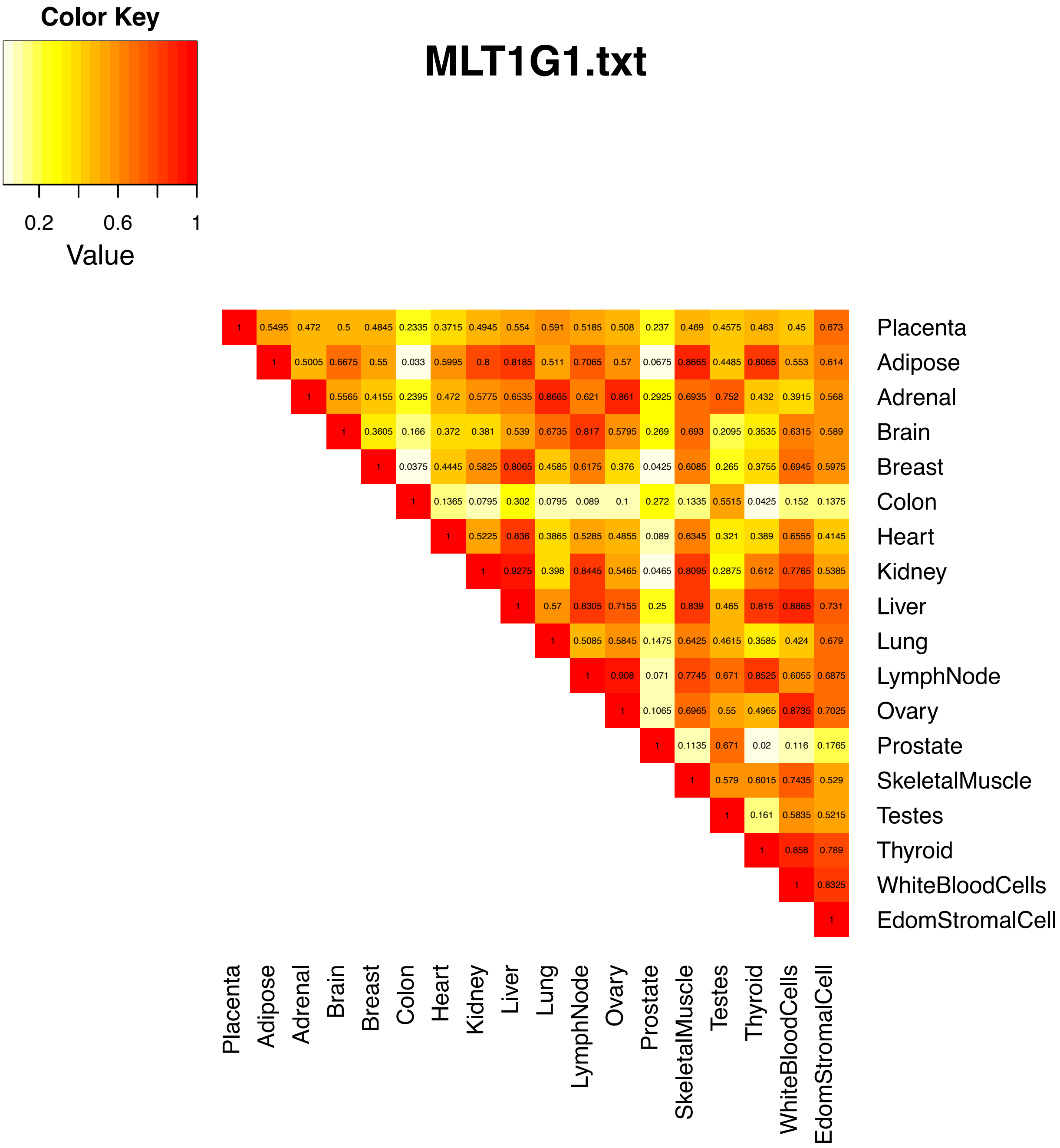
Color Key
0.2 0.6 1
Value
MLT1G1.txt
Placenta 1 0.5495 0.472 0.5 0.4845 0.2335 0.3715 0.4945 0.554 0.591 0.5185 0.508 0.237 0.469 0.4575 0.463 0.45 0.673
Adipose 1 0.5005 0.6675 0.55 0.033 0.5995 0.8 0.8185 0.511 0.7065 0.57 0.0675 0.8665 0.4485 0.8065 0.553 0.614
Adrenal 1 0.5565 0.4155 0.2395 0.472 0.5775 0.6535 0.8665 0.621 0.861 0.2925 0.6935 0.752 0.432 0.3915 0.568
Brain 1 0.3605 0.166 0.372 0.381 0.539 0.6735 0.817 0.5795 0.269 0.693 0.2095 0.3535 0.6315 0.589
Breast 1 0.0375 0.4445 0.5825 0.8065 0.4585 0.6175 0.376 0.0425 0.6085 0.265 0.3755 0.6945 0.5975
Colon 1 0.1365 0.0795 0.302 0.0795 0.089 0.1 0.272 0.1335 0.5515 0.0425 0.152 0.1375
Heart 1 0.5225 0.836 0.3865 0.5285 0.4855 0.089 0.6345 0.321 0.389 0.6555 0.4145
Kidney 1 0.9275 0.398 0.8445 0.5465 0.0465 0.8095 0.2875 0.612 0.7765 0.5385
Liver 1 0.57 0.8305 0.7155 0.25 0.839 0.465 0.815 0.8865 0.731
Lung 1 0.5085 0.5845 0.1475 0.6425 0.4615 0.3585 0.424 0.679
LymphNode 1 0.908 0.071 0.7745 0.671 0.8525 0.6055 0.6875
Ovary 1 0.1065 0.6965 0.55 0.4965 0.8735 0.7025
Prostate 1 0.1135 0.671 0.02 0.116 0.1765
SkeletalMuscle 1 0.579 0.6015 0.7435 0.529
Testes 1 0.161 0.5835 0.5215
Thyroid 1 0.858 0.789
WhiteBloodCells 1 0.8325
EdomStromalCell 1
Placenta Adipose Adrenal Brain Breast Colon Heart Kidney Liver Lung LymphNode Ovary Prostate SkeletalMuscle Testes Thyroid WhiteBloodCells EdomStromalCell

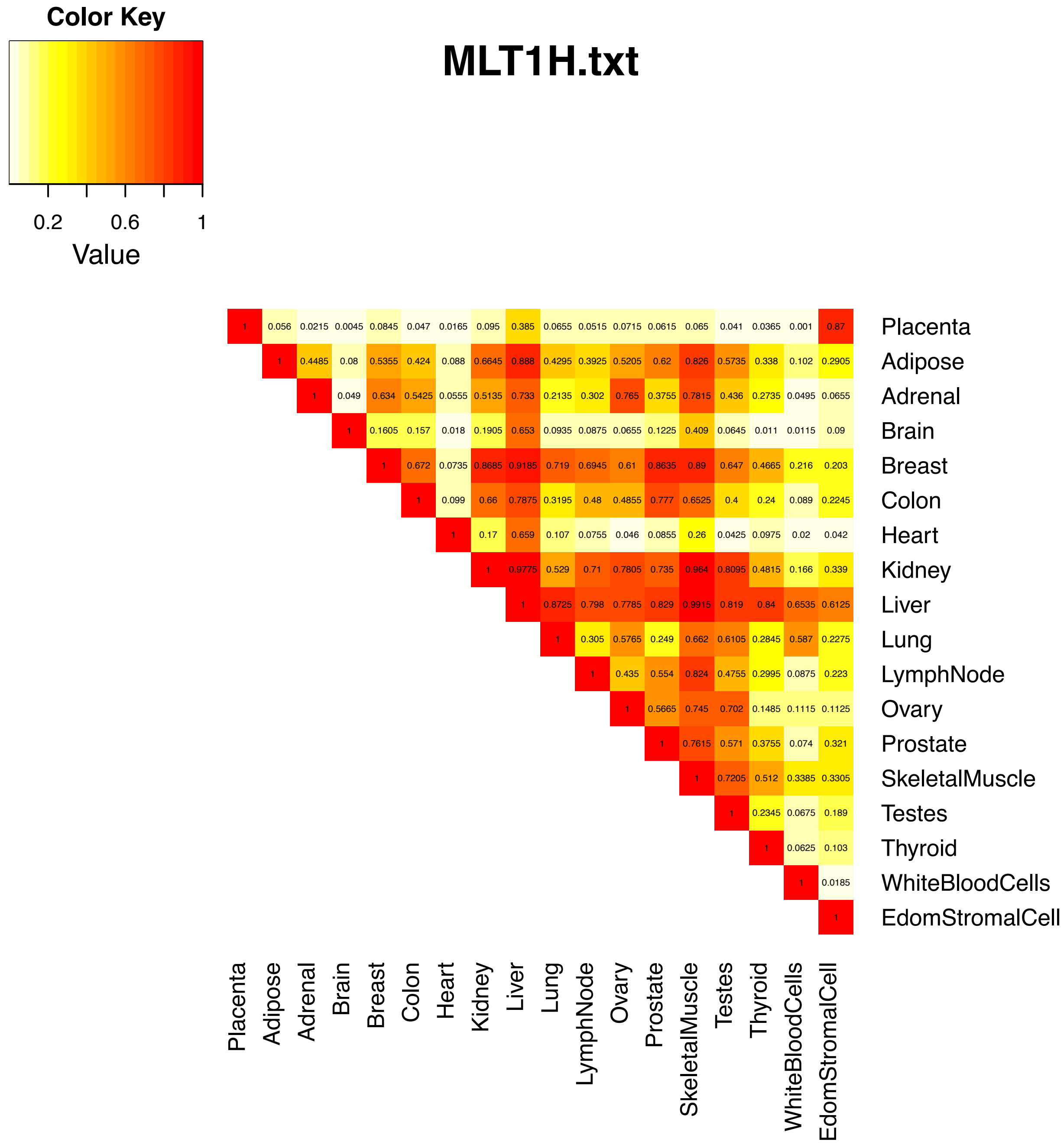
Color Key
0.2
0.6
1
Value
MLT1H.txt
Placenta: 1 0.056 0.0215 0.0045 0.0845 0.047 0.0165 0.095 0.385 0.0655 0.0515 0.0715 0.0615 0.065 0.041 0.0365 0.001 0.87
Adipose: 1 0.4485 0.08 0.5355 0.424 0.088 0.6645 0.888 0.4295 0.3925 0.5205 0.62 0.826 0.5735 0.338 0.102 0.2905
Adrenal: 1 0.049 0.634 0.5425 0.0555 0.5135 0.733 0.2135 0.302 0.765 0.3755 0.7815 0.436 0.2735 0.0495 0.0655
Brain: 1 0.1605 0.157 0.018 0.1905 0.653 0.0935 0.0875 0.0655 0.1225 0.409 0.0645 0.011 0.0115 0.09
Breast: 1 0.672 0.0735 0.8685 0.9185 0.719 0.6945 0.61 0.8635 0.89 0.647 0.4665 0.216 0.203
Colon: 1 0.099 0.66 0.7875 0.3195 0.48 0.4855 0.777 0.6525 0.4 0.24 0.089 0.2245
Heart: 1 0.17 0.659 0.107 0.0755 0.046 0.0855 0.26 0.0425 0.0975 0.02 0.042
Kidney: 1 0.9775 0.529 0.71 0.7805 0.735 0.964 0.8095 0.4815 0.166 0.339
Liver: 1 0.8725 0.798 0.7785 0.829 0.9915 0.819 0.84 0.6535 0.6125
Lung: 1 0.305 0.5765 0.249 0.662 0.6105 0.2845 0.587 0.2275
LymphNode: 1 0.435 0.554 0.824 0.4755 0.2995 0.0875 0.223
Ovary: 1 0.5665 0.745 0.702 0.1485 0.1115 0.1125
Prostate: 1 0.7615 0.571 0.3755 0.074 0.321
SkeletalMuscle: 1 0.7205 0.512 0.3385 0.3305
Testes: 1 0.2345 0.0675 0.189
Thyroid: 1 0.0625 0.103
WhiteBloodCells: 1 0.0185
EdomStromalCell: 1
Placenta Adipose Adrenal Brain Breast Colon Heart Kidney Liver Lung LymphNode Ovary Prostate SkeletalMuscle Testes Thyroid WhiteBloodCells EdomStromalCell

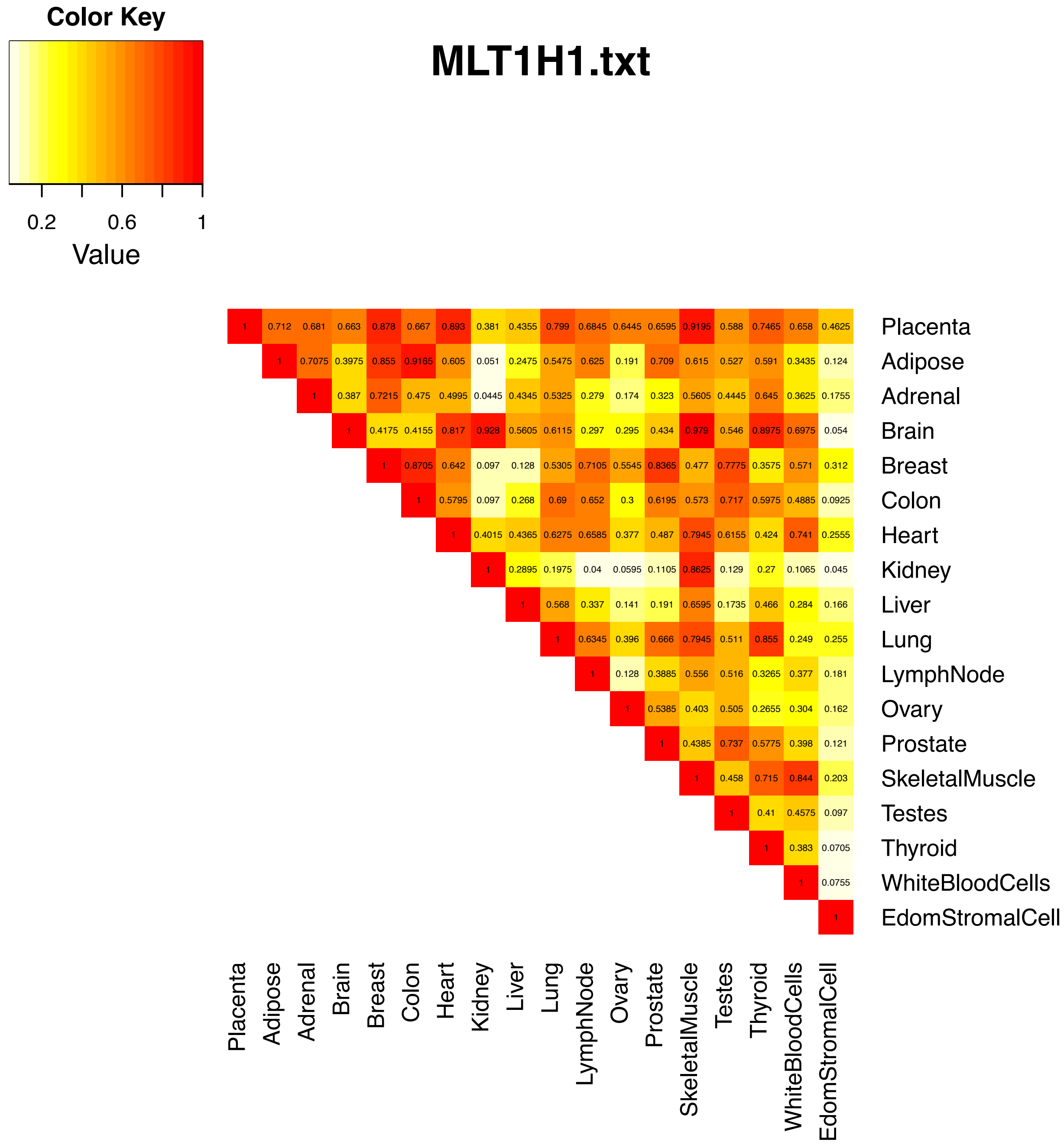

Color Key
0.2
0.6
1
Value
MLT1H1.txt
Placenta: 1 0.712 0.681 0.663 0.878 0.667 0.893 0.381 0.4355 0.799 0.6845 0.6445 0.6595 0.9195 0.588 0.7465 0.658 0.4625
Adipose: 1 0.7075 0.3975 0.855 0.9165 0.605 0.051 0.2475 0.5475 0.625 0.191 0.709 0.615 0.527 0.591 0.3435 0.124
Adrenal: 1 0.387 0.7215 0.475 0.4995 0.0445 0.4345 0.5325 0.279 0.174 0.323 0.5605 0.4445 0.645 0.3625 0.1755
Brain: 1 0.4175 0.4155 0.817 0.928 0.5605 0.6115 0.297 0.295 0.434 0.979 0.546 0.8975 0.6975 0.054
Breast: 1 0.8705 0.642 0.097 0.128 0.5305 0.7105 0.5545 0.8365 0.477 0.7775 0.3575 0.571 0.312
Colon: 1 0.5795 0.097 0.268 0.69 0.652 0.3 0.6195 0.573 0.717 0.5975 0.4885 0.0925
Heart: 1 0.4015 0.4365 0.6275 0.6585 0.377 0.487 0.7945 0.6155 0.424 0.741 0.2555
Kidney: 1 0.2895 0.1975 0.04 0.0595 0.1105 0.8625 0.129 0.27 0.1065 0.045
Liver: 1 0.568 0.337 0.141 0.191 0.6595 0.1735 0.466 0.284 0.166
Lung: 1 0.6345 0.396 0.666 0.7945 0.511 0.855 0.249 0.255
LymphNode: 1 0.128 0.3885 0.556 0.516 0.3265 0.377 0.181
Ovary: 1 0.5385 0.403 0.505 0.2655 0.304 0.162
Prostate: 1 0.4385 0.737 0.5775 0.398 0.121
SkeletalMuscle: 1 0.458 0.715 0.844 0.203
Testes: 1 0.41 0.4575 0.097
Thyroid: 1 0.383 0.0705
WhiteBloodCells: 1 0.0755
EdomStromalCell: 1
Placenta
Adipose
Adrenal
Brain
Breast
Colon
Heart
Kidney
Liver
Lung
LymphNode
Ovary
Prostate
SkeletalMuscle
Testes
Thyroid
WhiteBloodCells
EdomStromalCell

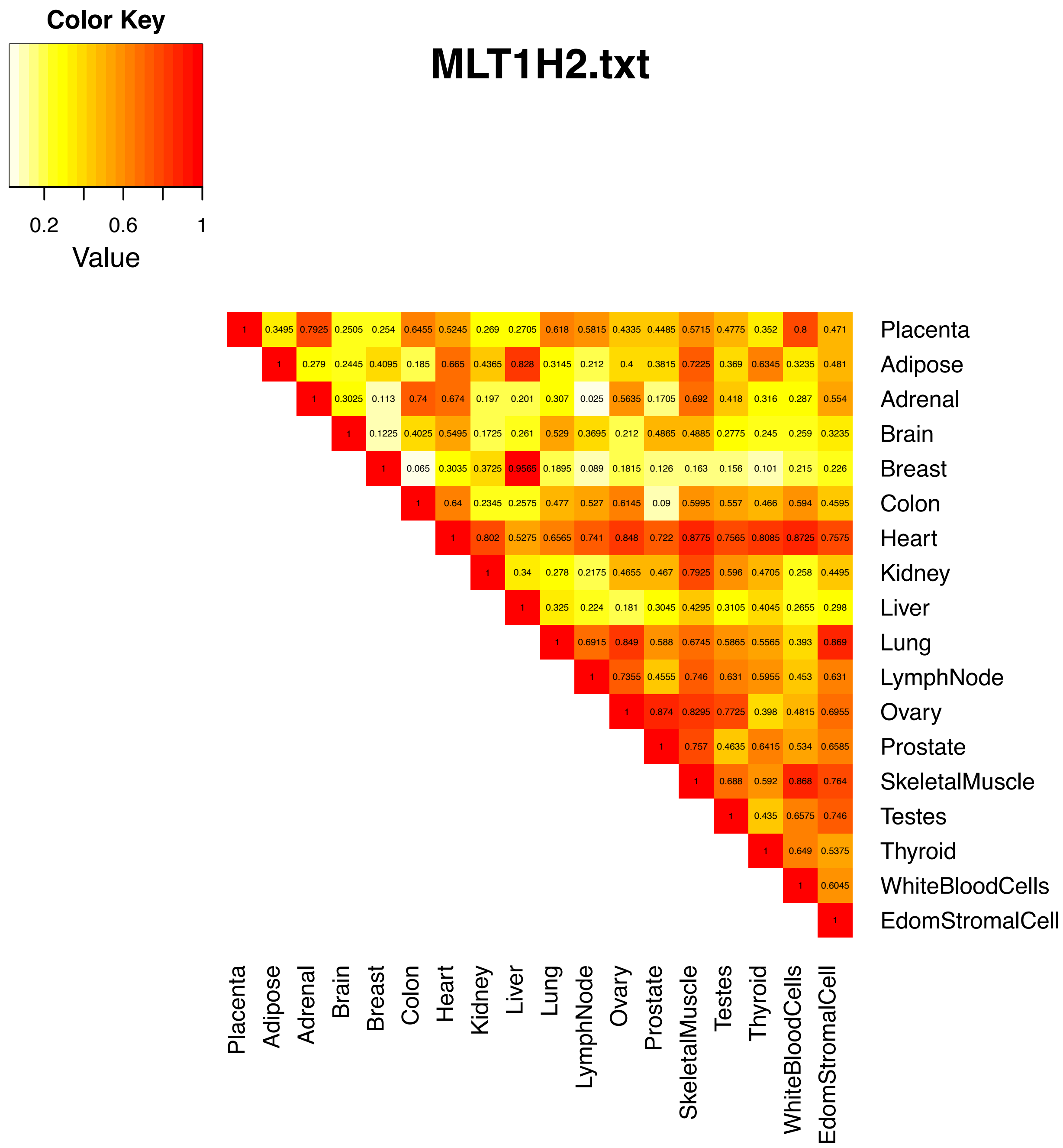
Color Key
0.2
0.6
1
Value
MLT1H2.txt
Placenta: 1 0.3495 0.7925 0.2505 0.254 0.6455 0.5245 0.269 0.2705 0.618 0.5815 0.4335 0.4485 0.5715 0.4775 0.352 0.8 0.471
Adipose: 1 0.279 0.2445 0.4095 0.185 0.665 0.4365 0.828 0.3145 0.212 0.4 0.3815 0.7225 0.369 0.6345 0.3235 0.481
Adrenal: 1 0.3025 0.113 0.74 0.674 0.197 0.201 0.307 0.025 0.5635 0.1705 0.692 0.418 0.316 0.287 0.554
Brain: 1 0.1225 0.4025 0.5495 0.1725 0.261 0.529 0.3695 0.212 0.4865 0.4885 0.2775 0.245 0.259 0.3235
Breast: 1 0.065 0.3035 0.3725 0.9565 0.1895 0.089 0.1815 0.126 0.163 0.156 0.101 0.215 0.226
Colon: 1 0.64 0.2345 0.2575 0.477 0.527 0.6145 0.09 0.5995 0.557 0.466 0.594 0.4595
Heart: 1 0.802 0.5275 0.6565 0.741 0.848 0.722 0.8775 0.7565 0.8085 0.8725 0.7575
Kidney: 1 0.34 0.278 0.2175 0.4655 0.467 0.7925 0.596 0.4705 0.258 0.4495
Liver: 1 0.325 0.224 0.181 0.3045 0.4295 0.3105 0.4045 0.2655 0.298
Lung: 1 0.6915 0.849 0.588 0.6745 0.5865 0.5565 0.393 0.869
LymphNode: 1 0.7355 0.4555 0.746 0.631 0.5955 0.453 0.631
Ovary: 1 0.874 0.8295 0.7725 0.398 0.4815 0.6955
Prostate: 1 0.757 0.4635 0.6415 0.534 0.6585
SkeletalMuscle: 1 0.688 0.592 0.868 0.764
Testes: 1 0.435 0.6575 0.746
Thyroid: 1 0.649 0.5375
WhiteBloodCells: 1 0.6045
EdomStromalCell: 1
Placenta
Adipose
Adrenal
Brain
Breast
Colon
Heart
Kidney
Liver
Lung
LymphNode
Ovary
Prostate
SkeletalMuscle
Testes
Thyroid
WhiteBloodCells
EdomStromalCell

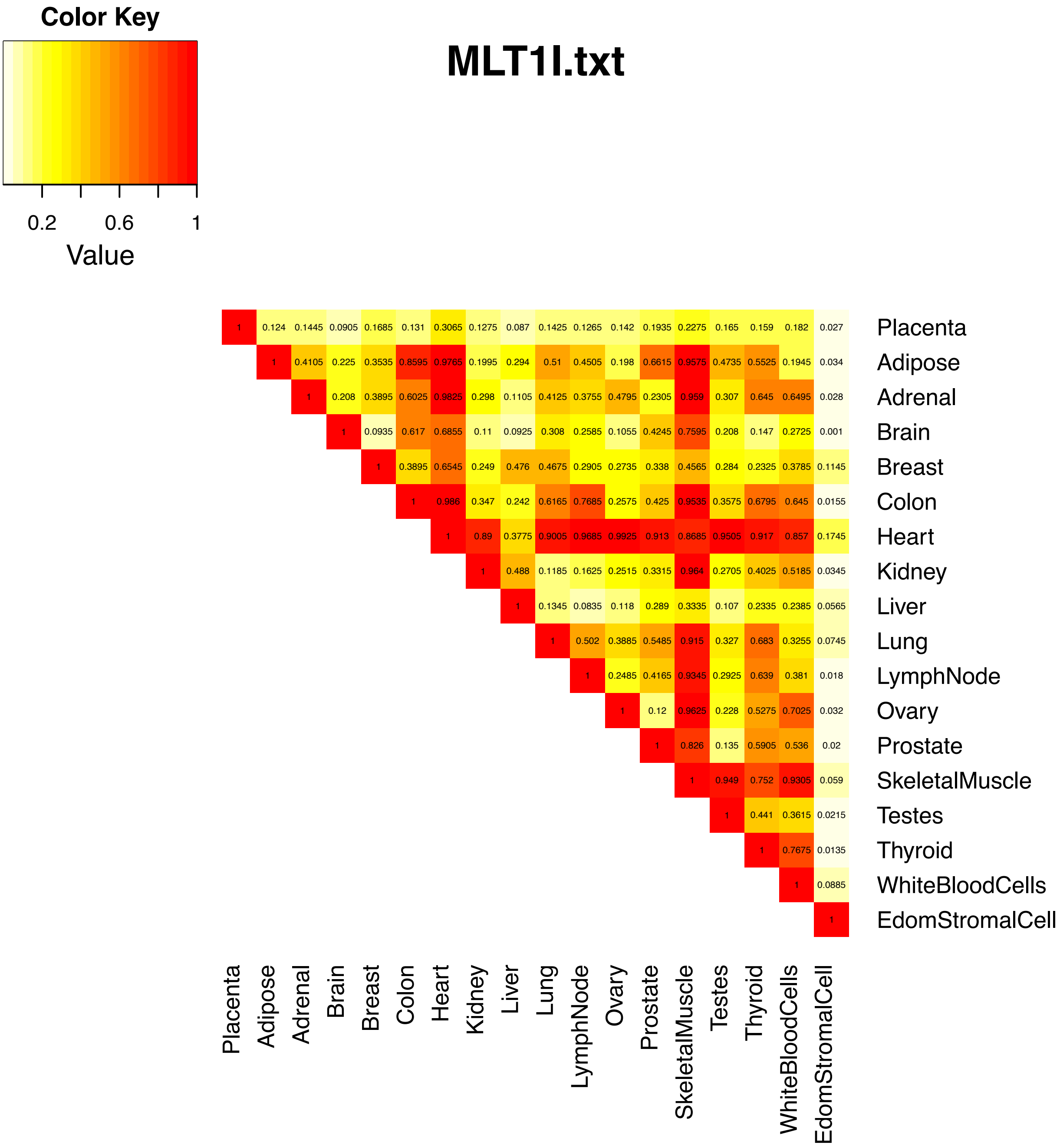
Color Key
0.2
0.6
1
Value
MLT1I.txt
Placenta 1 0.124 0.1445 0.0905 0.1685 0.131 0.3065 0.1275 0.087 0.1425 0.1265 0.142 0.1935 0.2275 0.165 0.159 0.182 0.027
Adipose 1 0.4105 0.225 0.3535 0.8595 0.9765 0.1995 0.294 0.51 0.4505 0.198 0.6615 0.9575 0.4735 0.5525 0.1945 0.034
Adrenal 1 0.208 0.3895 0.6025 0.9825 0.298 0.1105 0.4125 0.3755 0.4795 0.2305 0.959 0.307 0.645 0.6495 0.028
Brain 1 0.0935 0.617 0.6855 0.11 0.0925 0.308 0.2585 0.1055 0.4245 0.7595 0.208 0.147 0.2725 0.001
Breast 1 0.3895 0.6545 0.249 0.476 0.4675 0.2905 0.2735 0.338 0.4565 0.284 0.2325 0.3785 0.1145
Colon 1 0.986 0.347 0.242 0.6165 0.7685 0.2575 0.425 0.9535 0.3575 0.6795 0.645 0.0155
Heart 1 0.89 0.3775 0.9005 0.9685 0.9925 0.913 0.8685 0.9505 0.917 0.857 0.1745
Kidney 1 0.488 0.1185 0.1625 0.2515 0.3315 0.964 0.2705 0.4025 0.5185 0.0345
Liver 1 0.1345 0.0835 0.118 0.289 0.3335 0.107 0.2335 0.2385 0.0565
Lung 1 0.502 0.3885 0.5485 0.915 0.327 0.683 0.3255 0.0745
LymphNode 1 0.2485 0.4165 0.9345 0.2925 0.639 0.381 0.018
Ovary 1 0.12 0.9625 0.228 0.5275 0.7025 0.032
Prostate 1 0.826 0.135 0.5905 0.536 0.02
SkeletalMuscle 1 0.949 0.752 0.9305 0.059
Testes 1 0.441 0.3615 0.0215
Thyroid 1 0.7675 0.0135
WhiteBloodCells 1 0.0885
EdomStromalCell 1
Placenta
Adipose
Adrenal
Brain
Breast
Colon
Heart
Kidney
Liver
Lung
LymphNode
Ovary
Prostate
SkeletalMuscle
Testes
Thyroid
WhiteBloodCells
EdomStromalCell

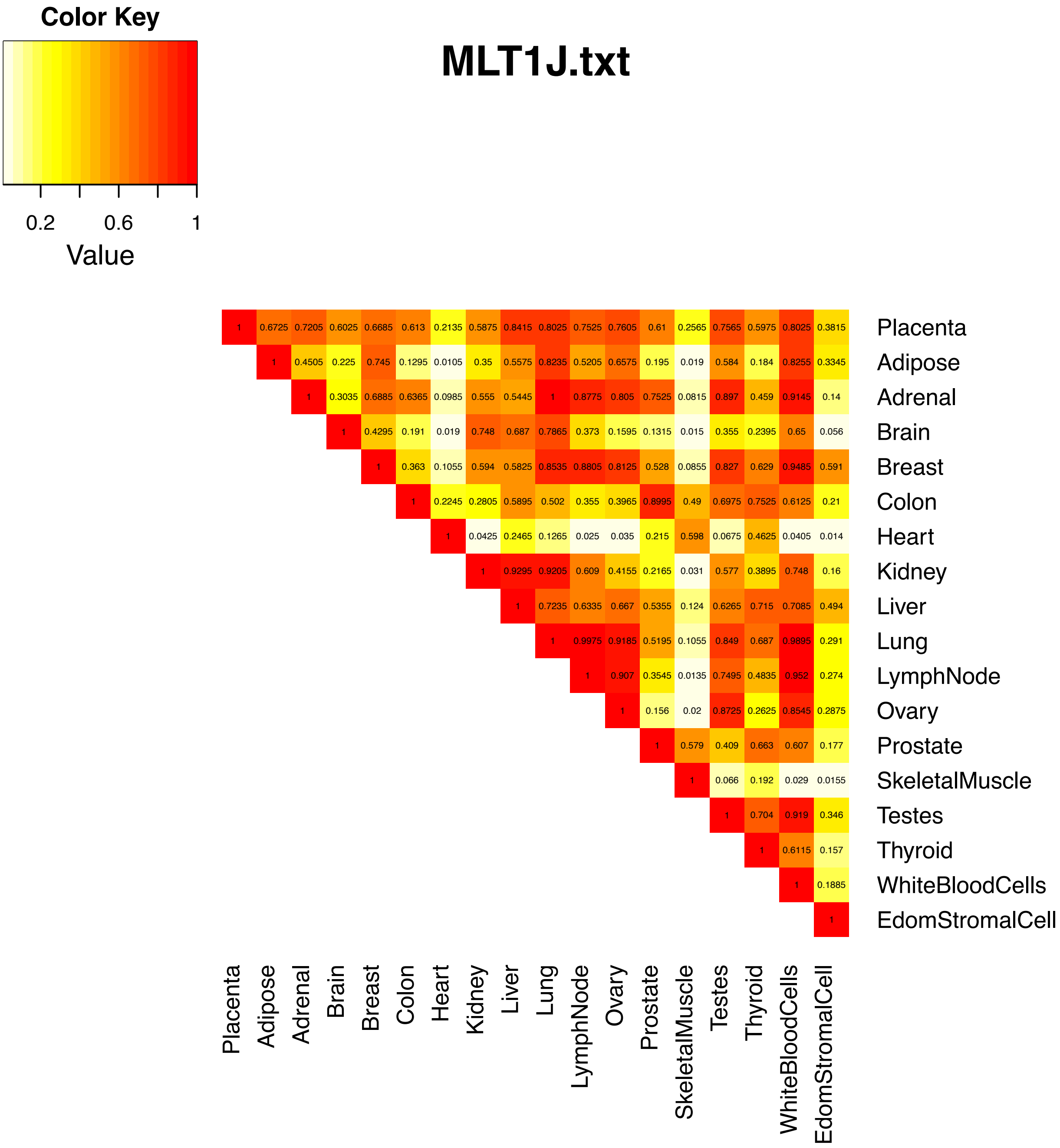
Color Key
0.2
0.6
1
Value
MLT1J.txt
Placenta: 1 0.6725 0.7205 0.6025 0.6685 0.613 0.2135 0.5875 0.8415 0.8025 0.7525 0.7605 0.61 0.2565 0.7565 0.5975 0.8025 0.3815
Adipose: 1 0.4505 0.225 0.745 0.1295 0.0105 0.35 0.5575 0.8235 0.5205 0.6575 0.195 0.019 0.584 0.184 0.8255 0.3345
Adrenal: 1 0.3035 0.6885 0.6365 0.0985 0.555 0.5445 1 0.8775 0.805 0.7525 0.0815 0.897 0.459 0.9145 0.14
Brain: 1 0.4295 0.191 0.019 0.748 0.687 0.7865 0.373 0.1595 0.1315 0.015 0.355 0.2395 0.65 0.056
Breast: 1 0.363 0.1055 0.594 0.5825 0.8535 0.8805 0.8125 0.528 0.0855 0.827 0.629 0.9485 0.591
Colon: 1 0.2245 0.2805 0.5895 0.502 0.355 0.3965 0.8995 0.49 0.6975 0.7525 0.6125 0.21
Heart: 1 0.0425 0.2465 0.1265 0.025 0.035 0.215 0.598 0.0675 0.4625 0.0405 0.014
Kidney: 1 0.9295 0.9205 0.609 0.4155 0.2165 0.031 0.577 0.3895 0.748 0.16
Liver: 1 0.7235 0.6335 0.667 0.5355 0.124 0.6265 0.715 0.7085 0.494
Lung: 1 0.9975 0.9185 0.5195 0.1055 0.849 0.687 0.9895 0.291
LymphNode: 1 0.907 0.3545 0.0135 0.7495 0.4835 0.952 0.274
Ovary: 1 0.156 0.02 0.8725 0.2625 0.8545 0.2875
Prostate: 1 0.579 0.409 0.663 0.607 0.177
SkeletalMuscle: 1 0.066 0.192 0.029 0.0155
Testes: 1 0.704 0.919 0.346
Thyroid: 1 0.6115 0.157
WhiteBloodCells: 1 0.1885
EdomStromalCell: 1
Placenta Adipose Adrenal Brain Breast Colon Heart Kidney Liver Lung LymphNode Ovary Prostate SkeletalMuscle Testes Thyroid WhiteBloodCells EdomStromalCell

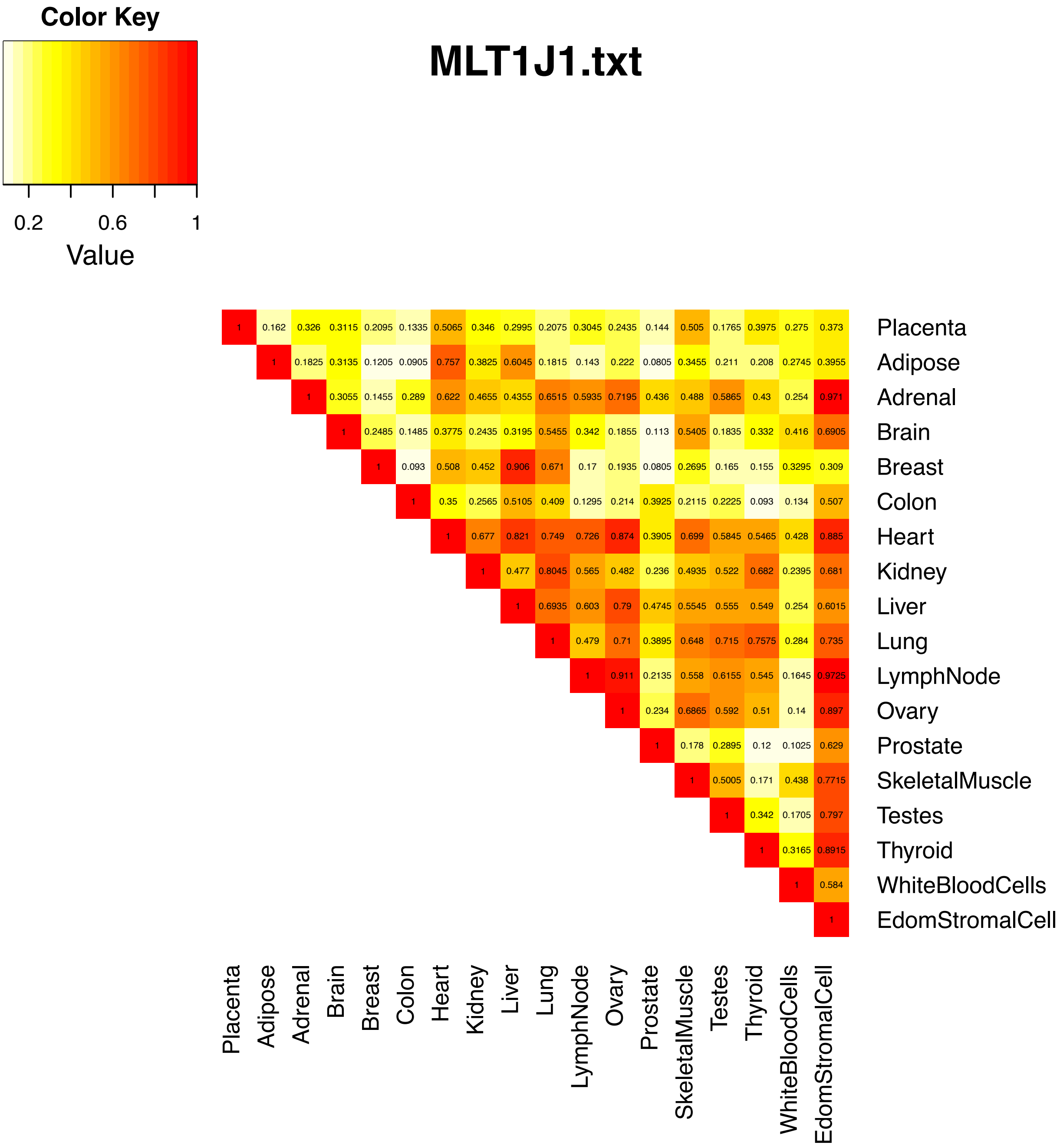
Color Key
0.2
0.6
1
Value
MLT1J1.txt
Placenta: 1 0.162 0.326 0.3115 0.2095 0.1335 0.5065 0.346 0.2995 0.2075 0.3045 0.2435 0.144 0.505 0.1765 0.3975 0.275 0.373
Adipose: 1 0.1825 0.3135 0.1205 0.0905 0.757 0.3825 0.6045 0.1815 0.143 0.222 0.0805 0.3455 0.211 0.208 0.2745 0.3955
Adrenal: 1 0.3055 0.1455 0.289 0.622 0.4655 0.4355 0.6515 0.5935 0.7195 0.436 0.488 0.5865 0.43 0.254 0.971
Brain: 1 0.2485 0.1485 0.3775 0.2435 0.3195 0.5455 0.342 0.1855 0.113 0.5405 0.1835 0.332 0.416 0.6905
Breast: 1 0.093 0.508 0.452 0.906 0.671 0.17 0.1935 0.0805 0.2695 0.165 0.155 0.3295 0.309
Colon: 1 0.35 0.2565 0.5105 0.409 0.1295 0.214 0.3925 0.2115 0.2225 0.093 0.134 0.507
Heart: 1 0.677 0.821 0.749 0.726 0.874 0.3905 0.699 0.5845 0.5465 0.428 0.885
Kidney: 1 0.477 0.8045 0.565 0.482 0.236 0.4935 0.522 0.682 0.2395 0.681
Liver: 1 0.6935 0.603 0.79 0.4745 0.5545 0.555 0.549 0.254 0.6015
Lung: 1 0.479 0.71 0.3895 0.648 0.715 0.7575 0.284 0.735
LymphNode: 1 0.911 0.2135 0.558 0.6155 0.545 0.1645 0.9725
Ovary: 1 0.234 0.6865 0.592 0.51 0.14 0.897
Prostate: 1 0.178 0.2895 0.12 0.1025 0.629
SkeletalMuscle: 1 0.5005 0.171 0.438 0.7715
Testes: 1 0.342 0.1705 0.797
Thyroid: 1 0.3165 0.8915
WhiteBloodCells: 1 0.584
EdomStromalCell: 1
Placenta Adipose Adrenal Brain Breast Colon Heart Kidney Liver Lung LymphNode Ovary Prostate SkeletalMuscle Testes Thyroid WhiteBloodCells EdomStromalCell

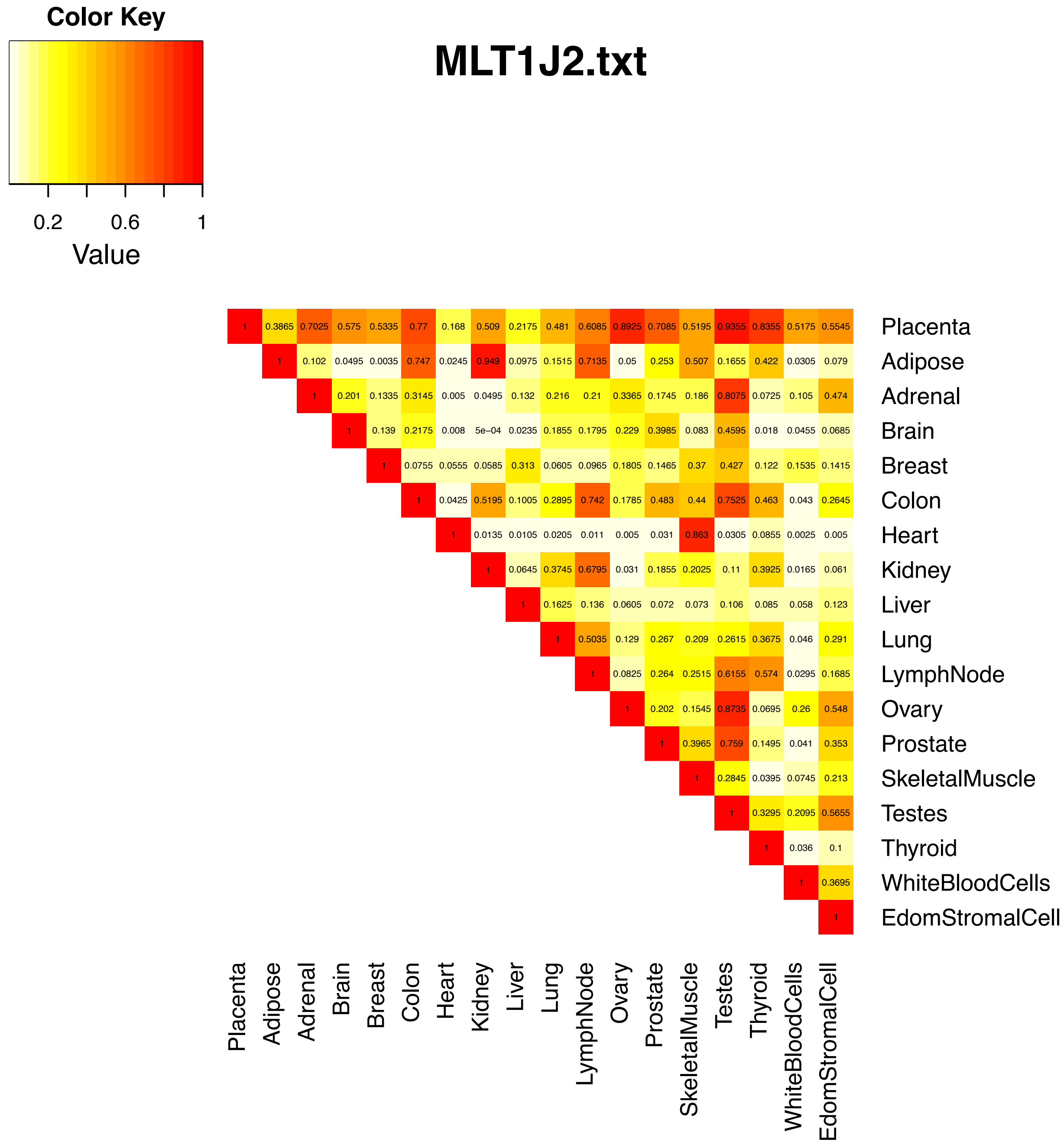
Color Key
0.2 0.6 1
Value
MLT1J2.txt
Placenta 1 0.3865 0.7025 0.575 0.5335 0.77 0.168 0.509 0.2175 0.481 0.6085 0.8925 0.7085 0.5195 0.9355 0.8355 0.5175 0.5545
Adipose 1 0.102 0.0495 0.0035 0.747 0.0245 0.949 0.0975 0.1515 0.7135 0.05 0.253 0.507 0.1655 0.422 0.0305 0.079
Adrenal 1 0.201 0.1335 0.3145 0.005 0.0495 0.132 0.216 0.21 0.3365 0.1745 0.186 0.8075 0.0725 0.105 0.474
Brain 1 0.139 0.2175 0.008 5e−04 0.0235 0.1855 0.1795 0.229 0.3985 0.083 0.4595 0.018 0.0455 0.0685
Breast 1 0.0755 0.0555 0.0585 0.313 0.0605 0.0965 0.1805 0.1465 0.37 0.427 0.122 0.1535 0.1415
Colon 1 0.0425 0.5195 0.1005 0.2895 0.742 0.1785 0.483 0.44 0.7525 0.463 0.043 0.2645
Heart 1 0.0135 0.0105 0.0205 0.011 0.005 0.031 0.863 0.0305 0.0855 0.0025 0.005
Kidney 1 0.0645 0.3745 0.6795 0.031 0.1855 0.2025 0.11 0.3925 0.0165 0.061
Liver 1 0.1625 0.136 0.0605 0.072 0.073 0.106 0.085 0.058 0.123
Lung 1 0.5035 0.129 0.267 0.209 0.2615 0.3675 0.046 0.291
LymphNode 1 0.0825 0.264 0.2515 0.6155 0.574 0.0295 0.1685
Ovary 1 0.202 0.1545 0.8735 0.0695 0.26 0.548
Prostate 1 0.3965 0.759 0.1495 0.041 0.353
SkeletalMuscle 1 0.2845 0.0395 0.0745 0.213
Testes 1 0.3295 0.2095 0.5655
Thyroid 1 0.036 0.1
WhiteBloodCells 1 0.3695
EdomStromalCell 1
Placenta Adipose Adrenal Brain Breast Colon Heart Kidney Liver Lung LymphNode Ovary Prostate SkeletalMuscle Testes Thyroid WhiteBloodCells EdomStromalCell

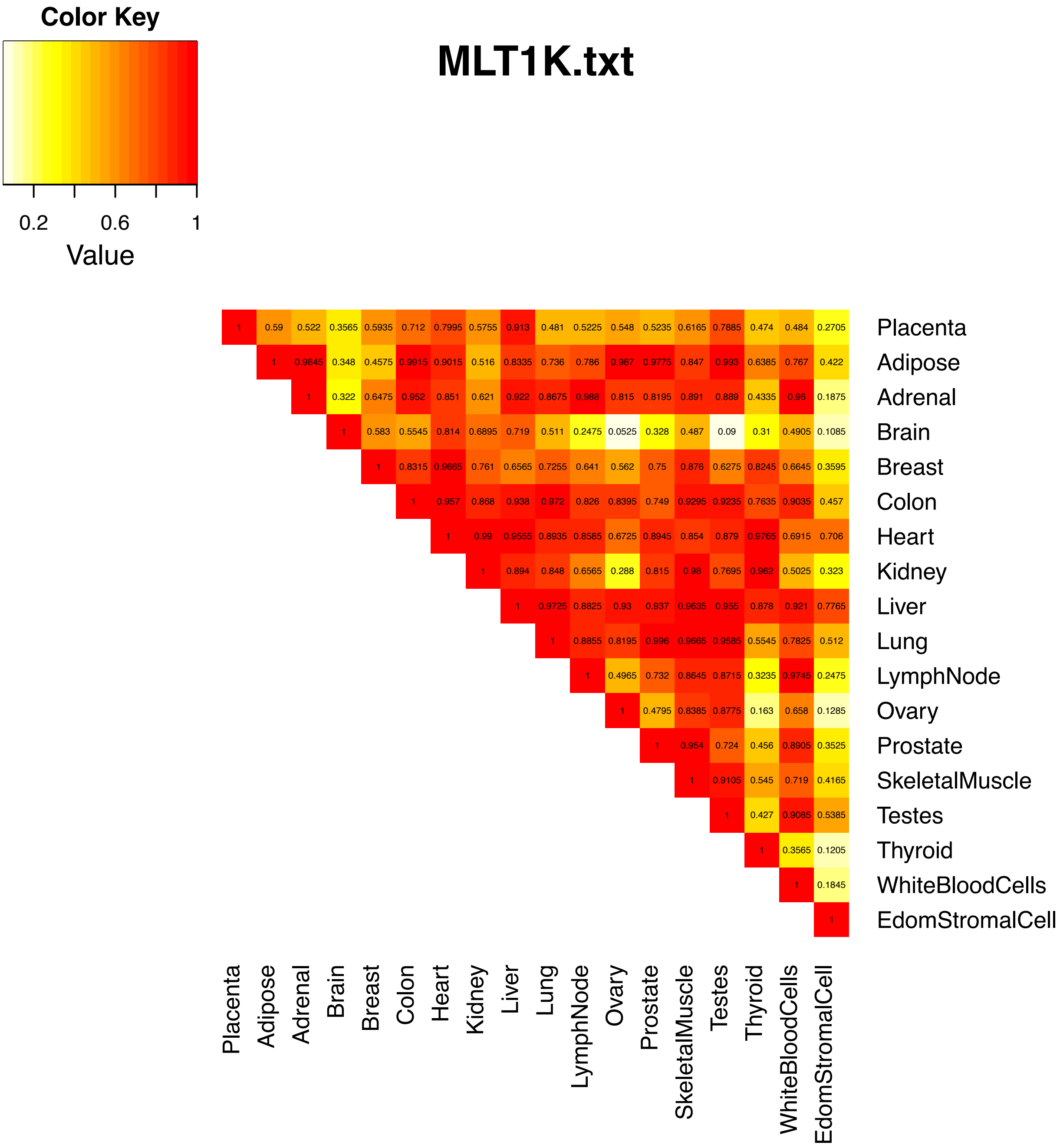
Color Key
0.2
0.6
1
Value
MLT1K.txt
Placenta 1 0.59 0.522 0.3565 0.5935 0.712 0.7995 0.5755 0.913 0.481 0.5225 0.548 0.5235 0.6165 0.7885 0.474 0.484 0.2705
Adipose 1 0.9845 0.348 0.4575 0.9915 0.9015 0.516 0.8335 0.736 0.786 0.987 0.9775 0.847 0.993 0.6385 0.767 0.422
Adrenal 1 0.322 0.6475 0.952 0.851 0.621 0.922 0.8675 0.988 0.815 0.8195 0.891 0.889 0.4335 0.96 0.1875
Brain 1 0.583 0.5545 0.814 0.6895 0.719 0.511 0.2475 0.0525 0.328 0.487 0.09 0.31 0.4905 0.1085
Breast 1 0.8315 0.9665 0.761 0.6565 0.7255 0.641 0.562 0.75 0.876 0.6275 0.8245 0.6645 0.3595
Colon 1 0.957 0.868 0.938 0.972 0.826 0.8395 0.749 0.9295 0.9235 0.7635 0.9035 0.457
Heart 1 0.99 0.9555 0.8935 0.8585 0.6725 0.8945 0.854 0.879 0.9765 0.6915 0.706
Kidney 1 0.894 0.848 0.6565 0.288 0.815 0.98 0.7695 0.962 0.5025 0.323
Liver 1 0.9725 0.8825 0.93 0.937 0.9635 0.955 0.878 0.921 0.7765
Lung 1 0.8855 0.8195 0.996 0.9665 0.9585 0.5545 0.7825 0.512
LymphNode 1 0.4965 0.732 0.8645 0.8715 0.3235 0.9745 0.2475
Ovary 1 0.4795 0.8385 0.8775 0.163 0.658 0.1285
Prostate 1 0.954 0.724 0.456 0.8905 0.3525
SkeletalMuscle 1 0.9105 0.545 0.719 0.4165
Testes 1 0.427 0.9085 0.5385
Thyroid 1 0.3565 0.1205
WhiteBloodCells 1 0.1845
EdomStromalCell 1
Placenta
Adipose
Adrenal
Brain
Breast
Colon
Heart
Kidney
Liver
Lung
LymphNode
Ovary
Prostate
SkeletalMuscle
Testes
Thyroid
WhiteBloodCells
EdomStromalCell

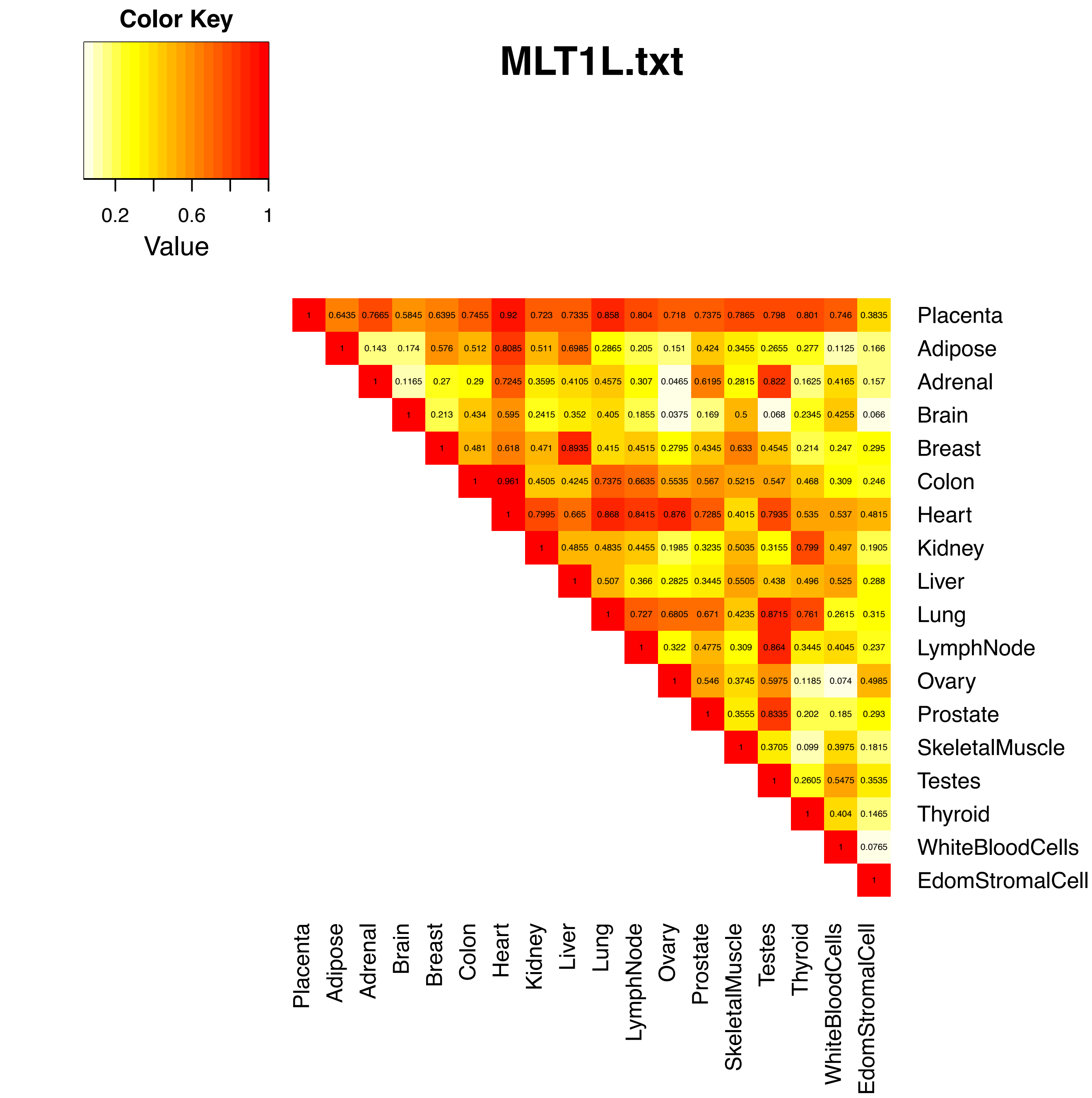
Color Key
0.2
0.6
1
Value
MLT1L.txt
Placenta
Adipose
Adrenal
Brain
Breast
Colon
Heart
Kidney
Liver
Lung
LymphNode
Ovary
Prostate
SkeletalMuscle
Testes
Thyroid
WhiteBloodCells
EdomStromalCell
1 0.6435 0.7665 0.5845 0.6395 0.7455 0.92 0.723 0.7335 0.858 0.804 0.718 0.7375 0.7865 0.798 0.801 0.746 0.3835
1 0.143 0.174 0.576 0.512 0.8085 0.511 0.6985 0.2865 0.205 0.151 0.424 0.3455 0.2655 0.277 0.1125 0.166
1 0.1165 0.27 0.29 0.7245 0.3595 0.4105 0.4575 0.307 0.0465 0.6195 0.2815 0.822 0.1625 0.4165 0.157
1 0.213 0.434 0.595 0.2415 0.352 0.405 0.1855 0.0375 0.169 0.5 0.068 0.2345 0.4255 0.066
1 0.481 0.618 0.471 0.8935 0.415 0.4515 0.2795 0.4345 0.633 0.4545 0.214 0.247 0.295
1 0.961 0.4505 0.4245 0.7375 0.6635 0.5535 0.567 0.5215 0.547 0.468 0.309 0.246
1 0.7995 0.665 0.868 0.8415 0.876 0.7285 0.4015 0.7935 0.535 0.537 0.4815
1 0.4855 0.4835 0.4455 0.1985 0.3235 0.5035 0.3155 0.799 0.497 0.1905
1 0.507 0.366 0.2825 0.3445 0.5505 0.438 0.496 0.525 0.288
1 0.727 0.6805 0.671 0.4235 0.8715 0.761 0.2615 0.315
1 0.322 0.4775 0.309 0.864 0.3445 0.4045 0.237
1 0.546 0.3745 0.5975 0.1185 0.074 0.4985
1 0.3555 0.8335 0.202 0.185 0.293
1 0.3705 0.099 0.3975 0.1815
1 0.2605 0.5475 0.3535
1 0.404 0.1465
1 0.0765
1

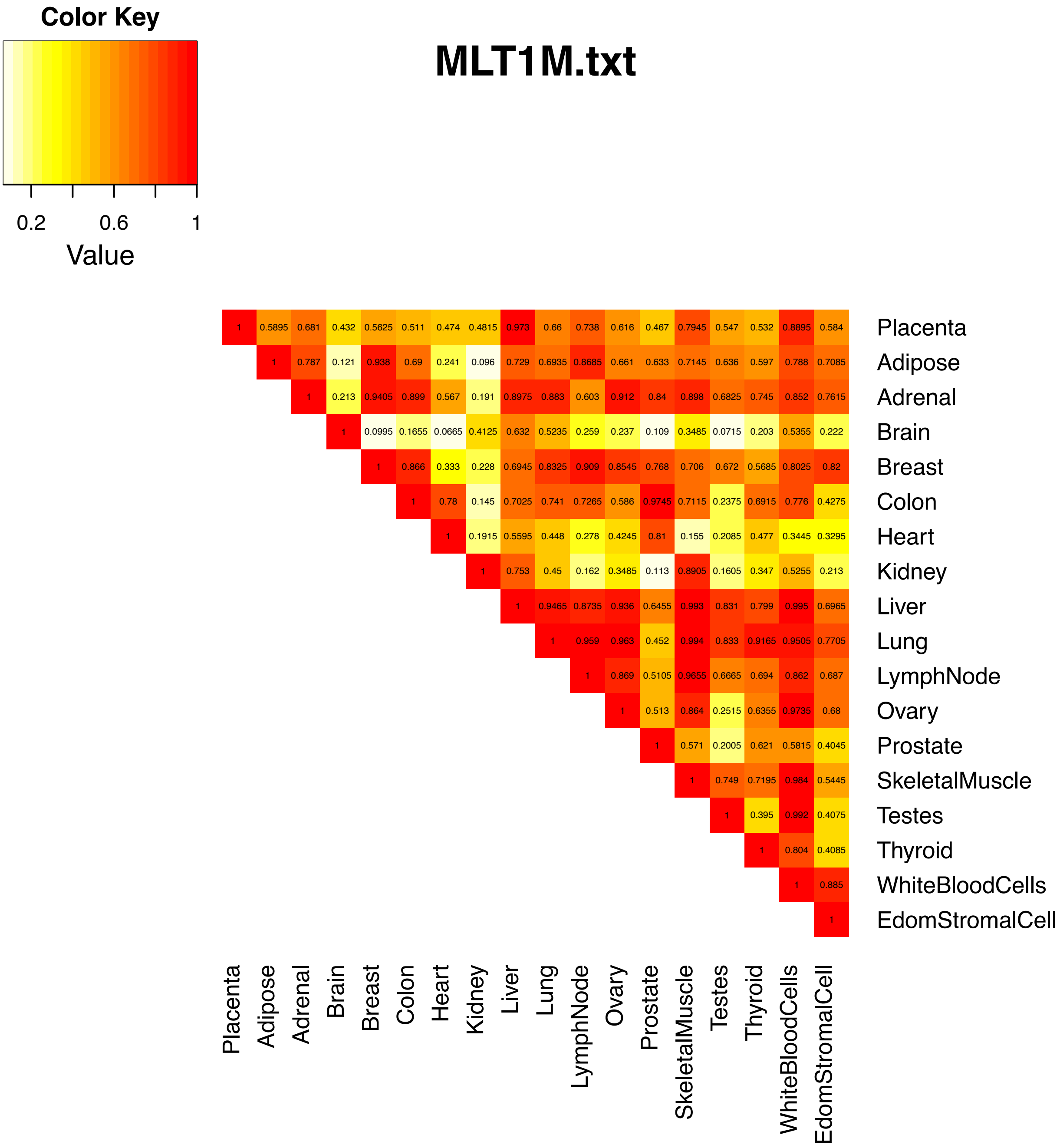
Color Key
0.2
0.6
1
Value
MLT1M.txt
Placenta 1 0.5895 0.681 0.432 0.5625 0.511 0.474 0.4815 0.973 0.66 0.738 0.616 0.467 0.7945 0.547 0.532 0.8895 0.584
Adipose 1 0.787 0.121 0.938 0.69 0.241 0.096 0.729 0.6935 0.8685 0.661 0.633 0.7145 0.636 0.597 0.788 0.7085
Adrenal 1 0.213 0.9405 0.899 0.567 0.191 0.8975 0.883 0.603 0.912 0.84 0.898 0.6825 0.745 0.852 0.7615
Brain 1 0.0995 0.1655 0.0665 0.4125 0.632 0.5235 0.259 0.237 0.109 0.3485 0.0715 0.203 0.5355 0.222
Breast 1 0.866 0.333 0.228 0.6945 0.8325 0.909 0.8545 0.768 0.706 0.672 0.5685 0.8025 0.82
Colon 1 0.78 0.145 0.7025 0.741 0.7265 0.586 0.9745 0.7115 0.2375 0.6915 0.776 0.4275
Heart 1 0.1915 0.5595 0.448 0.278 0.4245 0.81 0.155 0.2085 0.477 0.3445 0.3295
Kidney 1 0.753 0.45 0.162 0.3485 0.113 0.8905 0.1605 0.347 0.5255 0.213
Liver 1 0.9465 0.8735 0.936 0.6455 0.993 0.831 0.799 0.995 0.6965
Lung 1 0.959 0.963 0.452 0.994 0.833 0.9165 0.9505 0.7705
LymphNode 1 0.869 0.5105 0.9655 0.6665 0.694 0.862 0.687
Ovary 1 0.513 0.864 0.2515 0.6355 0.9735 0.68
Prostate 1 0.571 0.2005 0.621 0.5815 0.4045
SkeletalMuscle 1 0.749 0.7195 0.984 0.5445
Testes 1 0.395 0.992 0.4075
Thyroid 1 0.804 0.4085
WhiteBloodCells 1 0.885
EdomStromalCell 1
Placenta
Adipose
Adrenal
Brain
Breast
Colon
Heart
Kidney
Liver
Lung
LymphNode
Ovary
Prostate
SkeletalMuscle
Testes
Thyroid
WhiteBloodCells
EdomStromalCell

MLT1N2.txt

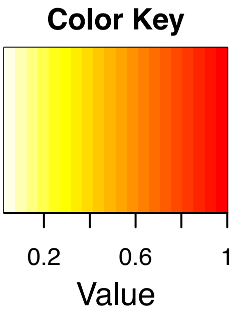

	Placenta	Adipose	Adrenal	Brain	Breast	Colon	Heart	Kidney	Liver	Lung	LymphNode	Ovary	Prostate	SkeletalMuscle	Testes	Thyroid	WhiteBloodCells	EdomStromalCell
Placenta	1	0.832	0.8925	0.942	0.876	0.8805	0.4745	0.904	0.8975	0.7695	0.939	0.9635	0.8345	0.471	0.9725	0.8585	0.972	0.951
Adipose		1	0.745	0.8085	0.7195	0.587	0.237	0.334	0.518	0.448	0.787	0.892	0.4985	0.223	0.7745	0.356	0.813	0.9435
Adrenal			1	0.8695	0.6415	0.911	0.356	0.4125	0.601	0.325	0.899	0.785	0.6615	0.1775	0.8785	0.701	0.8795	0.9185
Brain				1	0.608	0.814	0.358	0.6175	0.689	0.697	0.818	0.553	0.715	0.527	0.918	0.6005	0.919	0.817
Breast					1	0.5415	0.3075	0.4335	0.4695	0.492	0.7605	0.754	0.552	0.258	0.6505	0.361	0.8735	0.7615
Colon						1	0.5865	0.561	0.605	0.508	0.7295	0.7425	0.6255	0.182	0.815	0.7375	0.786	0.811
Heart							1	0.1515	0.4815	0.1165	0.1615	0.246	0.561	0.0905	0.3295	0.1815	0.2665	0.401
Kidney								1	0.7925	0.743	0.4015	0.3205	0.6595	0.372	0.6175	0.5475	0.4985	0.714
Liver									1	0.6725	0.5825	0.4285	0.5015	0.5415	0.6755	0.7615	0.681	0.694
Lung										1	0.3585	0.5615	0.604	0.481	0.5685	0.413	0.608	0.8605
LymphNode											1	0.821	0.4655	0.303	0.843	0.607	0.8175	0.9295
Ovary												1	0.5835	0.2235	0.909	0.3565	0.839	0.799
Prostate													1	0.4005	0.7565	0.622	0.676	0.566
SkeletalMuscle														1	0.2945	0.0265	0.397	0.3815
Testes															1	0.7705	0.829	0.7055
Thyroid																1	0.5785	0.734
WhiteBloodCells																	1	0.937
EdomStromalCell																		1

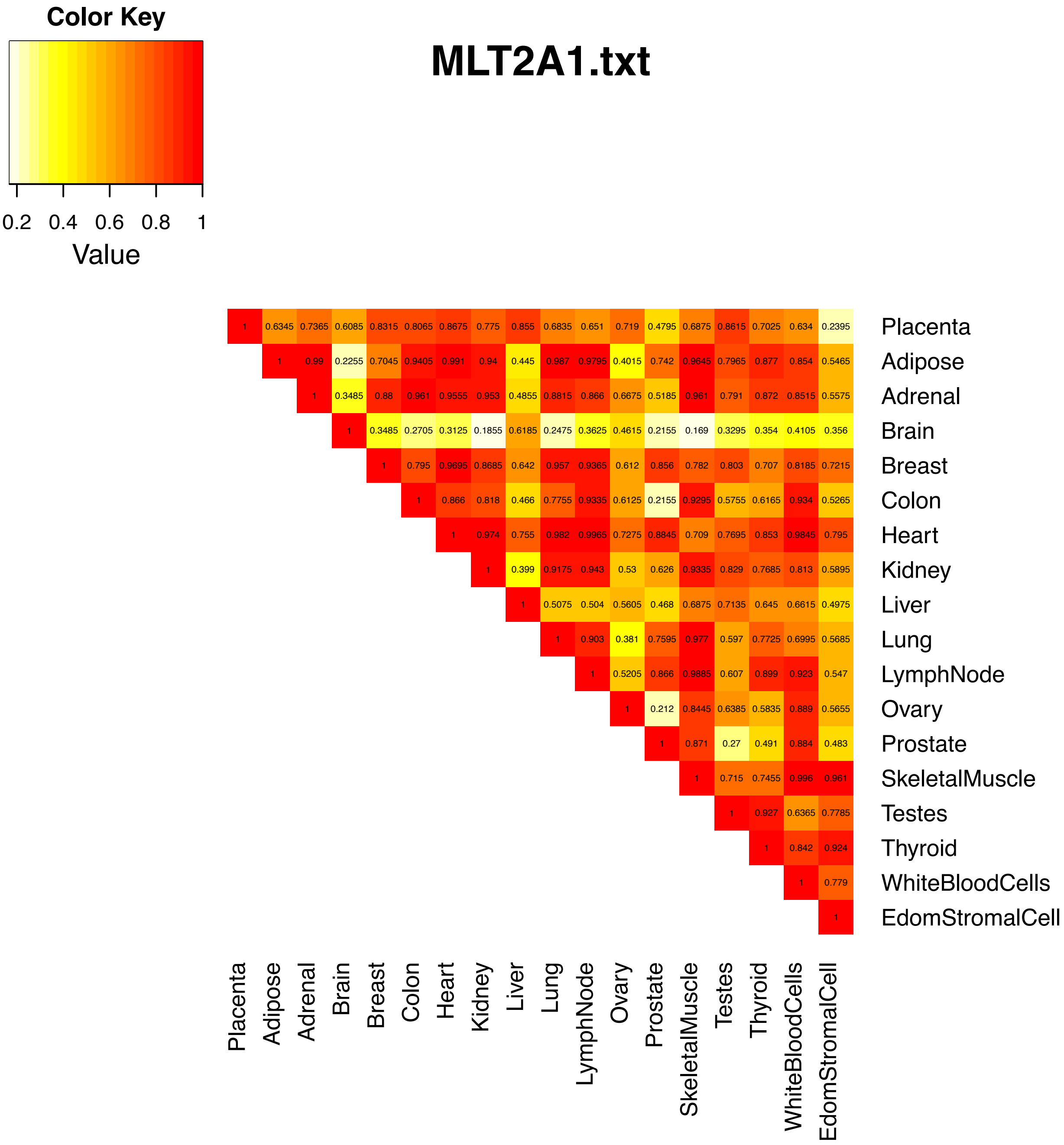
Color Key
0.2 0.4 0.6 0.8 1
Value
MLT2A1.txt
Placenta 1 0.6345 0.7365 0.6085 0.8315 0.8065 0.8675 0.775 0.855 0.6835 0.651 0.719 0.4795 0.6875 0.8615 0.7025 0.634 0.2395
Adipose 1 0.99 0.2255 0.7045 0.9405 0.991 0.94 0.445 0.987 0.9795 0.4015 0.742 0.9645 0.7965 0.877 0.854 0.5465
Adrenal 1 0.3485 0.88 0.961 0.9555 0.953 0.4855 0.8815 0.866 0.6675 0.5185 0.961 0.791 0.872 0.8515 0.5575
Brain 1 0.3485 0.2705 0.3125 0.1855 0.6185 0.2475 0.3625 0.4615 0.2155 0.169 0.3295 0.354 0.4105 0.356
Breast 1 0.795 0.9695 0.8685 0.642 0.957 0.9365 0.612 0.856 0.782 0.803 0.707 0.8185 0.7215
Colon 1 0.866 0.818 0.466 0.7755 0.9335 0.6125 0.2155 0.9295 0.5755 0.6165 0.934 0.5265
Heart 1 0.974 0.755 0.982 0.9965 0.7275 0.8845 0.709 0.7695 0.853 0.9845 0.795
Kidney 1 0.399 0.9175 0.943 0.53 0.626 0.9335 0.829 0.7685 0.813 0.5895
Liver 1 0.5075 0.504 0.5605 0.468 0.6875 0.7135 0.645 0.6615 0.4975
Lung 1 0.903 0.381 0.7595 0.977 0.597 0.7725 0.6995 0.5685
LymphNode 1 0.5205 0.866 0.9885 0.607 0.899 0.923 0.547
Ovary 1 0.212 0.8445 0.6385 0.5835 0.889 0.5655
Prostate 1 0.871 0.27 0.491 0.884 0.483
SkeletalMuscle 1 0.715 0.7455 0.996 0.961
Testes 1 0.927 0.6365 0.7785
Thyroid 1 0.842 0.924
WhiteBloodCells 1 0.779
EdomStromalCell 1
Placenta Adipose Adrenal Brain Breast Colon Heart Kidney Liver Lung LymphNode Ovary Prostate SkeletalMuscle Testes Thyroid WhiteBloodCells EdomStromalCell

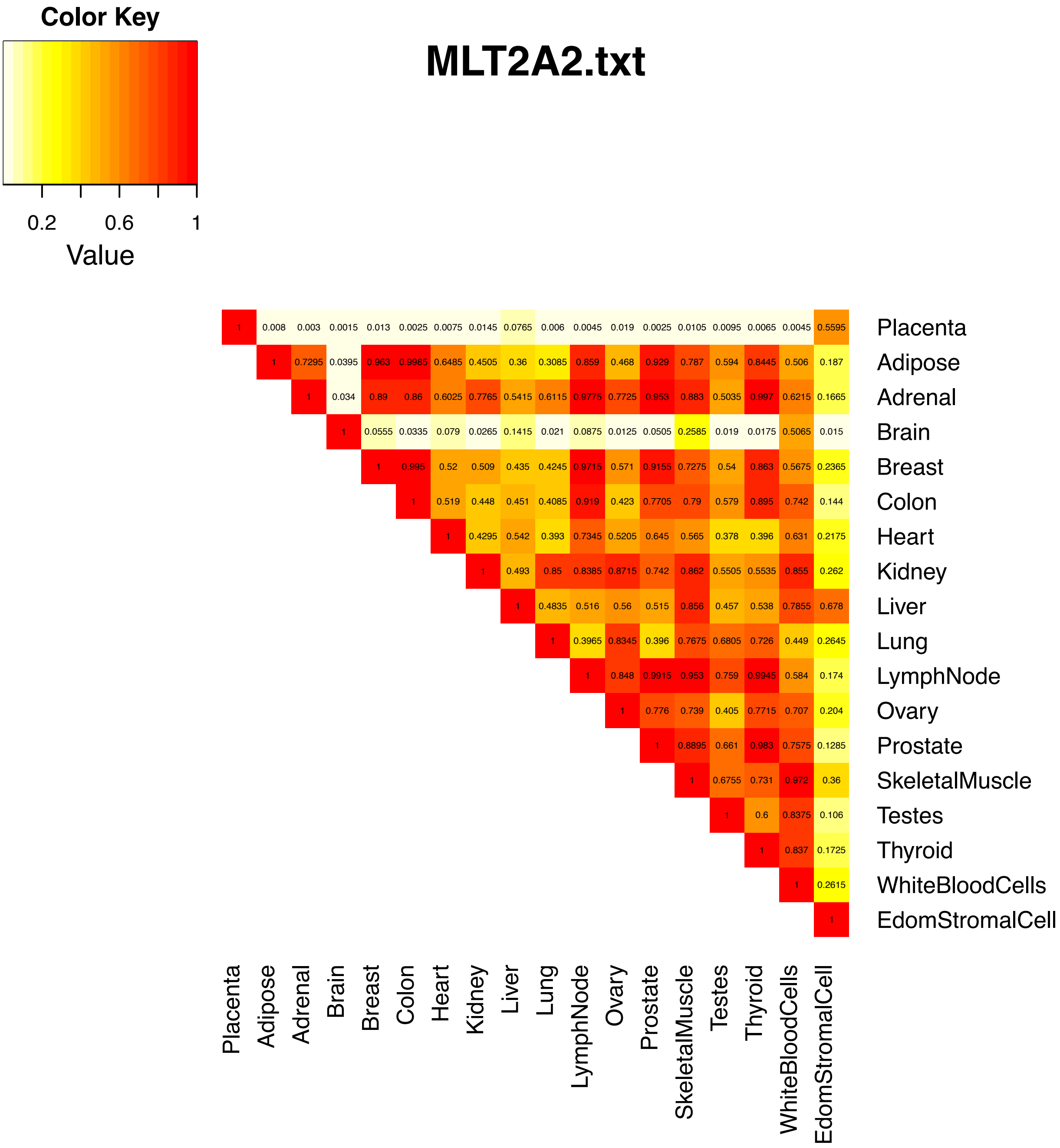
Color Key
0.2
0.6
1
Value
MLT2A2.txt
Placenta 1 0.008 0.003 0.0015 0.013 0.0025 0.0075 0.0145 0.0765 0.006 0.0045 0.019 0.0025 0.0105 0.0095 0.0065 0.0045 0.5595
Adipose 1 0.7295 0.0395 0.963 0.9985 0.6485 0.4505 0.36 0.3085 0.859 0.468 0.929 0.787 0.594 0.8445 0.506 0.187
Adrenal 1 0.034 0.89 0.86 0.6025 0.7765 0.5415 0.6115 0.9775 0.7725 0.953 0.883 0.5035 0.997 0.6215 0.1665
Brain 1 0.0555 0.0335 0.079 0.0265 0.1415 0.021 0.0875 0.0125 0.0505 0.2585 0.019 0.0175 0.5065 0.015
Breast 1 0.995 0.52 0.509 0.435 0.4245 0.9715 0.571 0.9155 0.7275 0.54 0.863 0.5675 0.2365
Colon 1 0.519 0.448 0.451 0.4085 0.919 0.423 0.7705 0.79 0.579 0.895 0.742 0.144
Heart 1 0.4295 0.542 0.393 0.7345 0.5205 0.645 0.565 0.378 0.396 0.631 0.2175
Kidney 1 0.493 0.85 0.8385 0.8715 0.742 0.862 0.5505 0.5535 0.855 0.262
Liver 1 0.4835 0.516 0.56 0.515 0.856 0.457 0.538 0.7855 0.678
Lung 1 0.3965 0.8345 0.396 0.7675 0.6805 0.726 0.449 0.2645
LymphNode 1 0.848 0.9915 0.953 0.759 0.9945 0.584 0.174
Ovary 1 0.776 0.739 0.405 0.7715 0.707 0.204
Prostate 1 0.8895 0.661 0.983 0.7575 0.1285
SkeletalMuscle 1 0.6755 0.731 0.972 0.36
Testes 1 0.6 0.8375 0.106
Thyroid 1 0.837 0.1725
WhiteBloodCells 1 0.2615
EdomStromalCell 1
Placenta
Adipose
Adrenal
Brain
Breast
Colon
Heart
Kidney
Liver
Lung
LymphNode
Ovary
Prostate
SkeletalMuscle
Testes
Thyroid
WhiteBloodCells
EdomStromalCell

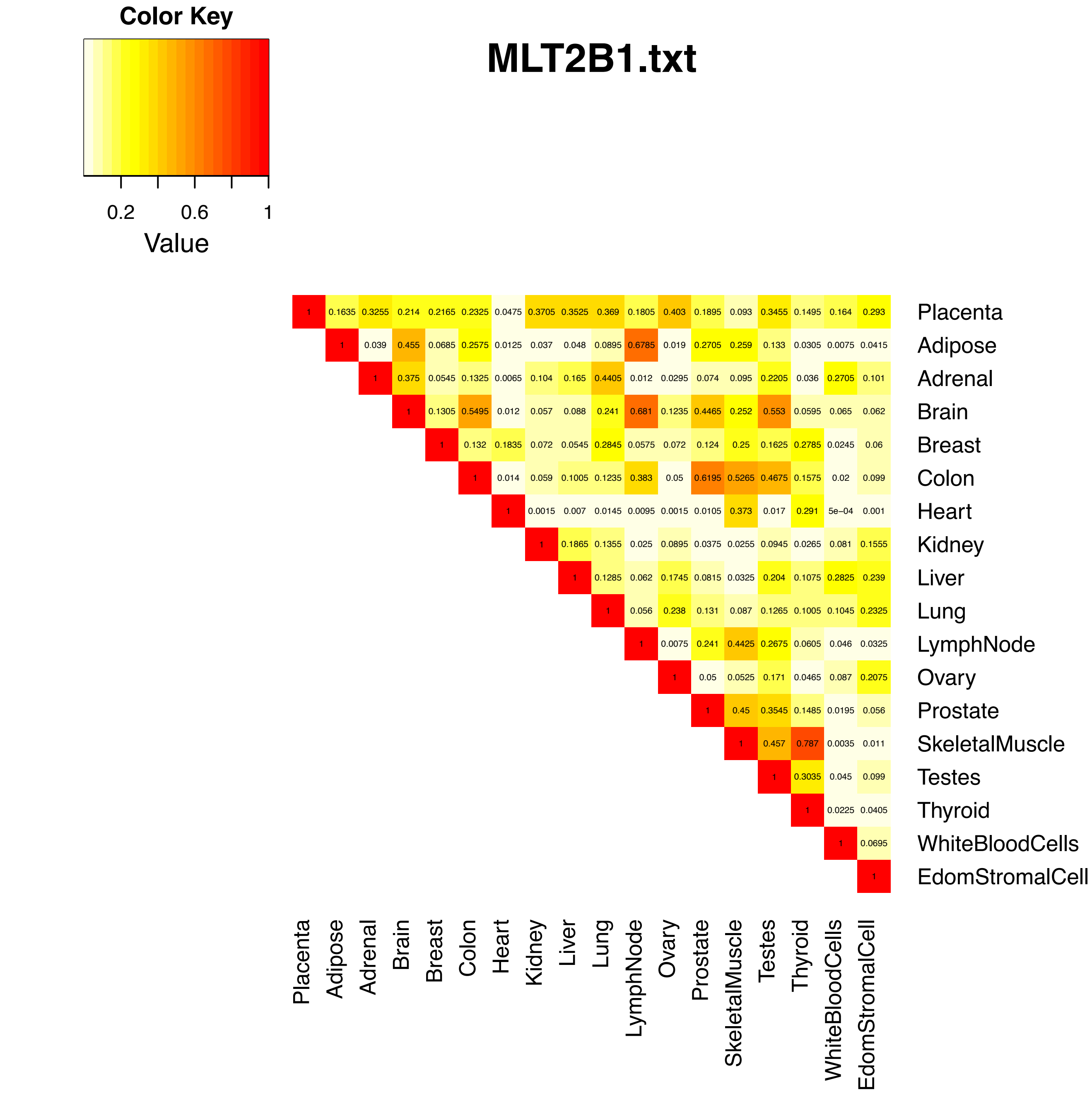
Color Key
0.2 0.6 1
Value
MLT2B1.txt
Placenta 1 0.1635 0.3255 0.214 0.2165 0.2325 0.0475 0.3705 0.3525 0.369 0.1805 0.403 0.1895 0.093 0.3455 0.1495 0.164 0.293
Adipose 1 0.039 0.455 0.0685 0.2575 0.0125 0.037 0.048 0.0895 0.6785 0.019 0.2705 0.259 0.133 0.0305 0.0075 0.0415
Adrenal 1 0.375 0.0545 0.1325 0.0065 0.104 0.165 0.4405 0.012 0.0295 0.074 0.095 0.2205 0.036 0.2705 0.101
Brain 1 0.1305 0.5495 0.012 0.057 0.088 0.241 0.681 0.1235 0.4465 0.252 0.553 0.0595 0.065 0.062
Breast 1 0.132 0.1835 0.072 0.0545 0.2845 0.0575 0.072 0.124 0.25 0.1625 0.2785 0.0245 0.06
Colon 1 0.014 0.059 0.1005 0.1235 0.383 0.05 0.6195 0.5265 0.4675 0.1575 0.02 0.099
Heart 1 0.0015 0.007 0.0145 0.0095 0.0015 0.0105 0.373 0.017 0.291 5e−04 0.001
Kidney 1 0.1865 0.1355 0.025 0.0895 0.0375 0.0255 0.0945 0.0265 0.081 0.1555
Liver 1 0.1285 0.062 0.1745 0.0815 0.0325 0.204 0.1075 0.2825 0.239
Lung 1 0.056 0.238 0.131 0.087 0.1265 0.1005 0.1045 0.2325
LymphNode 1 0.0075 0.241 0.4425 0.2675 0.0605 0.046 0.0325
Ovary 1 0.05 0.0525 0.171 0.0465 0.087 0.2075
Prostate 1 0.45 0.3545 0.1485 0.0195 0.056
SkeletalMuscle 1 0.457 0.787 0.0035 0.011
Testes 1 0.3035 0.045 0.099
Thyroid 1 0.0225 0.0405
WhiteBloodCells 1 0.0695
EdomStromalCell 1
Placenta Adipose Adrenal Brain Breast Colon Heart Kidney Liver Lung LymphNode Ovary Prostate SkeletalMuscle Testes Thyroid WhiteBloodCells EdomStromalCell

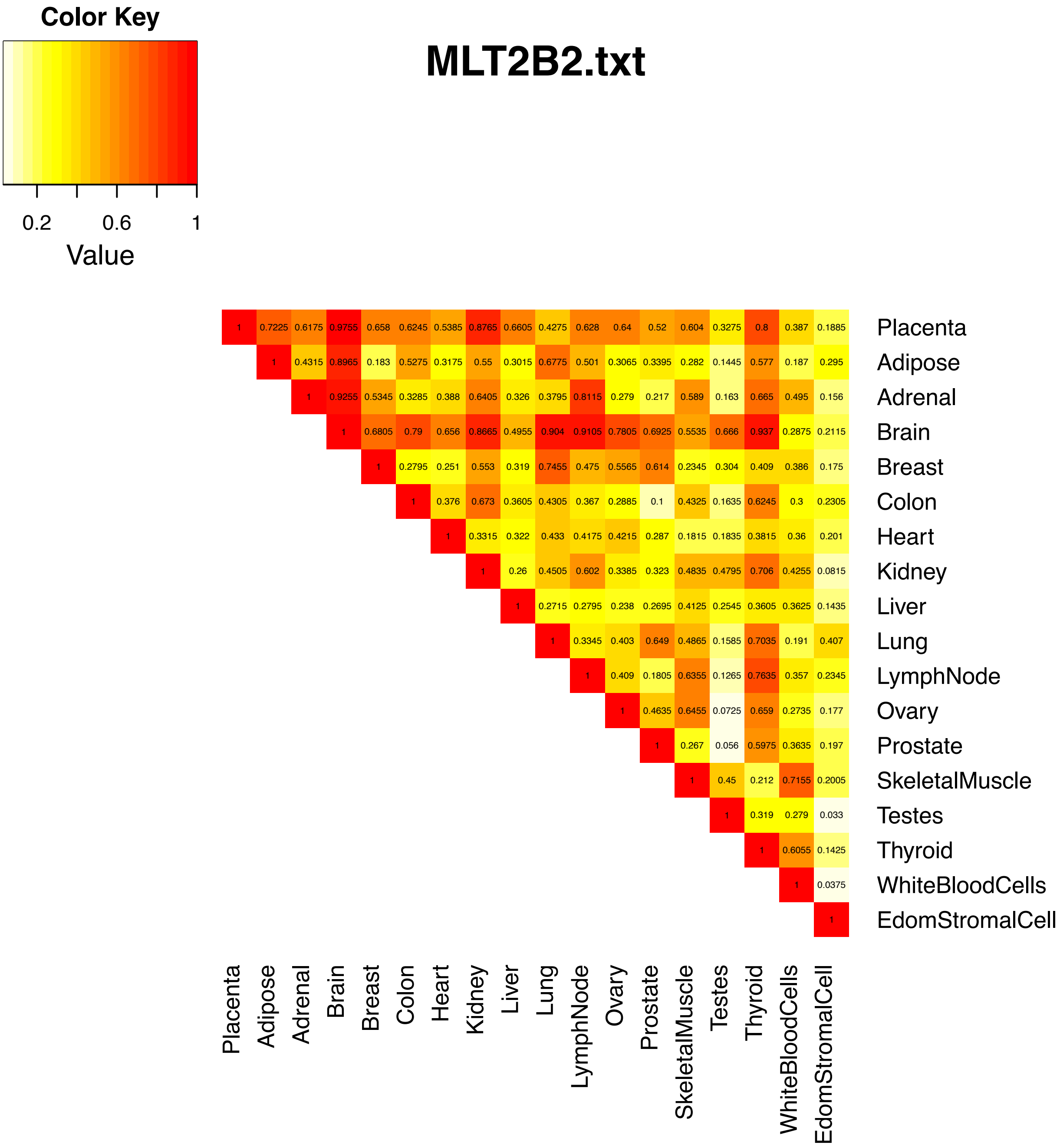
Color Key
0.2 0.6 1
Value
MLT2B2.txt
Placenta 1 0.7225 0.6175 0.9755 0.658 0.6245 0.5385 0.8765 0.6605 0.4275 0.628 0.64 0.52 0.604 0.3275 0.8 0.387 0.1885
Adipose 1 0.4315 0.8965 0.183 0.5275 0.3175 0.55 0.3015 0.6775 0.501 0.3065 0.3395 0.282 0.1445 0.577 0.187 0.295
Adrenal 1 0.9255 0.5345 0.3285 0.388 0.6405 0.326 0.3795 0.8115 0.279 0.217 0.589 0.163 0.665 0.495 0.156
Brain 1 0.6805 0.79 0.656 0.8665 0.4955 0.904 0.9105 0.7805 0.6925 0.5535 0.666 0.937 0.2875 0.2115
Breast 1 0.2795 0.251 0.553 0.319 0.7455 0.475 0.5565 0.614 0.2345 0.304 0.409 0.386 0.175
Colon 1 0.376 0.673 0.3605 0.4305 0.367 0.2885 0.1 0.4325 0.1635 0.6245 0.3 0.2305
Heart 1 0.3315 0.322 0.433 0.4175 0.4215 0.287 0.1815 0.1835 0.3815 0.36 0.201
Kidney 1 0.26 0.4505 0.602 0.3385 0.323 0.4835 0.4795 0.706 0.4255 0.0815
Liver 1 0.2715 0.2795 0.238 0.2695 0.4125 0.2545 0.3605 0.3625 0.1435
Lung 1 0.3345 0.403 0.649 0.4865 0.1585 0.7035 0.191 0.407
LymphNode 1 0.409 0.1805 0.6355 0.1265 0.7635 0.357 0.2345
Ovary 1 0.4635 0.6455 0.0725 0.659 0.2735 0.177
Prostate 1 0.267 0.056 0.5975 0.3635 0.197
SkeletalMuscle 1 0.45 0.212 0.7155 0.2005
Testes 1 0.319 0.279 0.033
Thyroid 1 0.6055 0.1425
WhiteBloodCells 1 0.0375
EdomStromalCell 1
Placenta Adipose Adrenal Brain Breast Colon Heart Kidney Liver Lung LymphNode Ovary Prostate SkeletalMuscle Testes Thyroid WhiteBloodCells EdomStromalCell

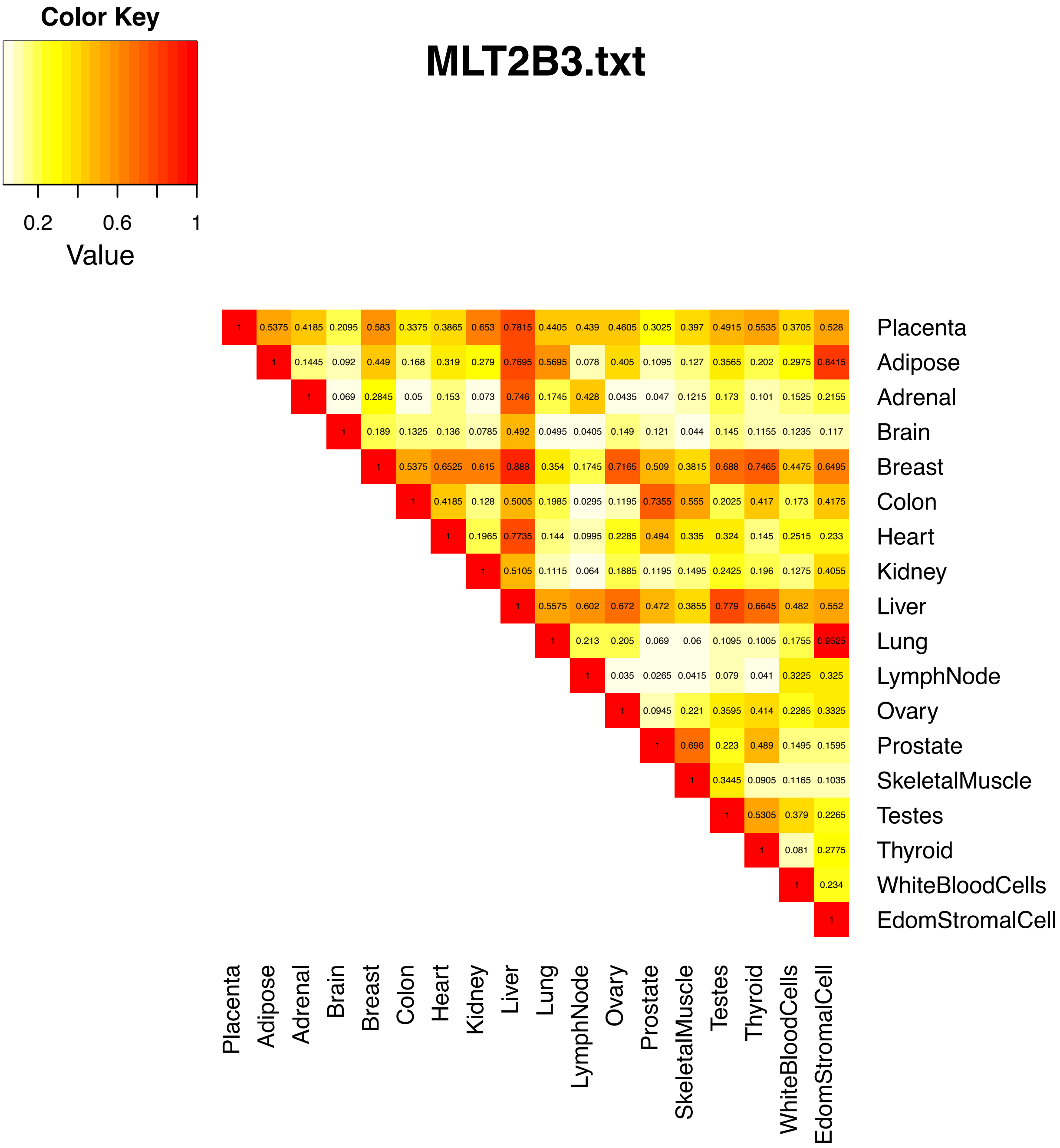
MLT2B3.txt
Color Key
0.2 0.6 1
Value
Placenta: 1 0.5375 0.4185 0.2095 0.583 0.3375 0.3865 0.653 0.7815 0.4405 0.439 0.4605 0.3025 0.397 0.4915 0.5535 0.3705 0.528
Adipose: 1 0.1445 0.092 0.449 0.168 0.319 0.279 0.7695 0.5695 0.078 0.405 0.1095 0.127 0.3565 0.202 0.2975 0.8415
Adrenal: 1 0.069 0.2845 0.05 0.153 0.073 0.746 0.1745 0.428 0.0435 0.047 0.1215 0.173 0.101 0.1525 0.2155
Brain: 1 0.189 0.1325 0.136 0.0785 0.492 0.0495 0.0405 0.149 0.121 0.044 0.145 0.1155 0.1235 0.117
Breast: 1 0.5375 0.6525 0.615 0.888 0.354 0.1745 0.7165 0.509 0.3815 0.688 0.7465 0.4475 0.6495
Colon: 1 0.4185 0.128 0.5005 0.1985 0.0295 0.1195 0.7355 0.555 0.2025 0.417 0.173 0.4175
Heart: 1 0.1965 0.7735 0.144 0.0995 0.2285 0.494 0.335 0.324 0.145 0.2515 0.233
Kidney: 1 0.5105 0.1115 0.064 0.1885 0.1195 0.1495 0.2425 0.196 0.1275 0.4055
Liver: 1 0.5575 0.602 0.672 0.472 0.3855 0.779 0.6645 0.482 0.552
Lung: 1 0.213 0.205 0.069 0.06 0.1095 0.1005 0.1755 0.9525
LymphNode: 1 0.035 0.0265 0.0415 0.079 0.041 0.3225 0.325
Ovary: 1 0.0945 0.221 0.3595 0.414 0.2285 0.3325
Prostate: 1 0.696 0.223 0.489 0.1495 0.1595
SkeletalMuscle: 1 0.3445 0.0905 0.1165 0.1035
Testes: 1 0.5305 0.379 0.2265
Thyroid: 1 0.081 0.2775
WhiteBloodCells: 1 0.234
EdomStromalCell: 1
Placenta Adipose Adrenal Brain Breast Colon Heart Kidney Liver Lung LymphNode Ovary Prostate SkeletalMuscle Testes Thyroid WhiteBloodCells EdomStromalCell

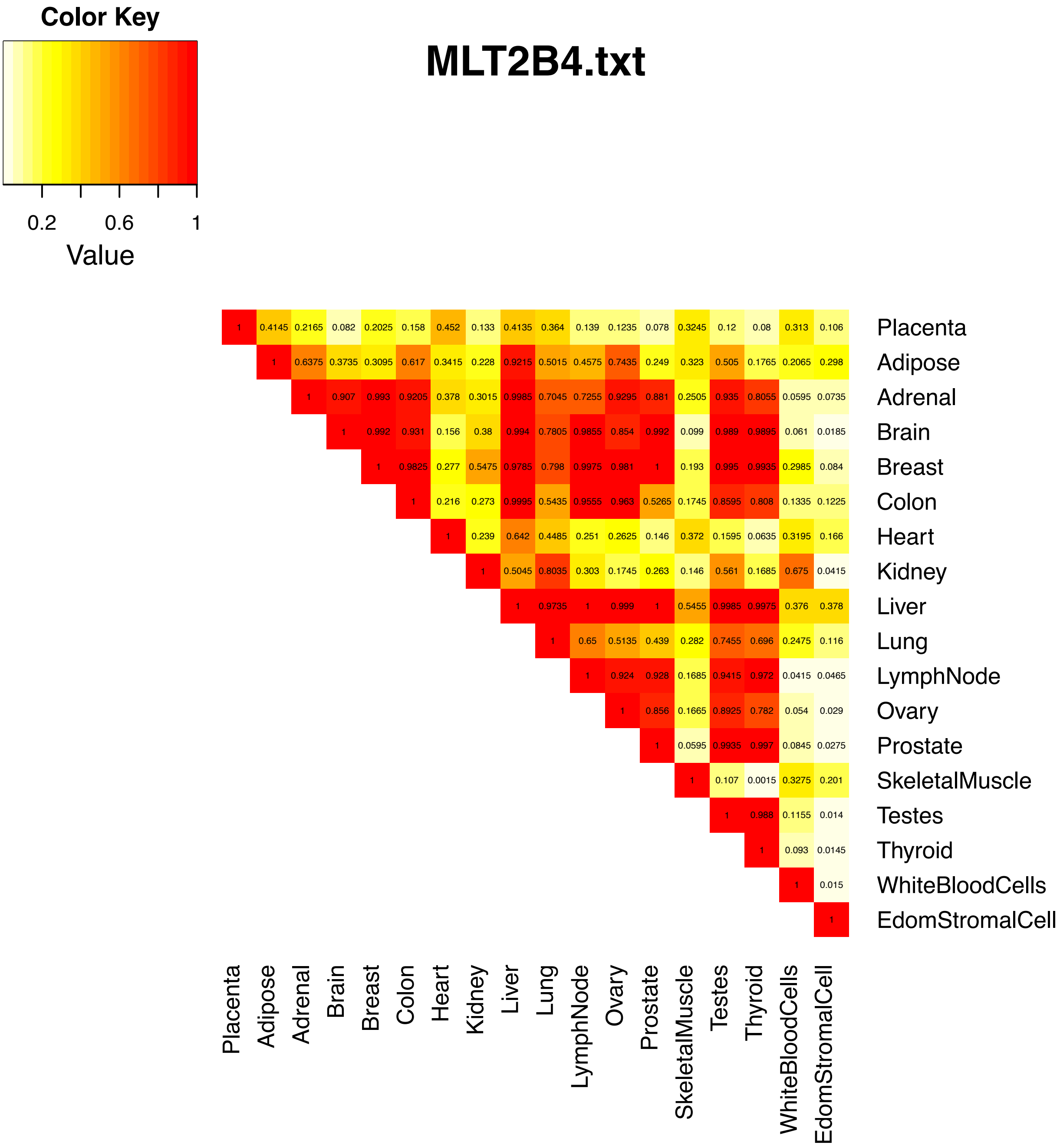
Color Key
0.2
0.6
1
Value
MLT2B4.txt
Placenta
Adipose
Adrenal
Brain
Breast
Colon
Heart
Kidney
Liver
Lung
LymphNode
Ovary
Prostate
SkeletalMuscle
Testes
Thyroid
WhiteBloodCells
EdomStromalCell

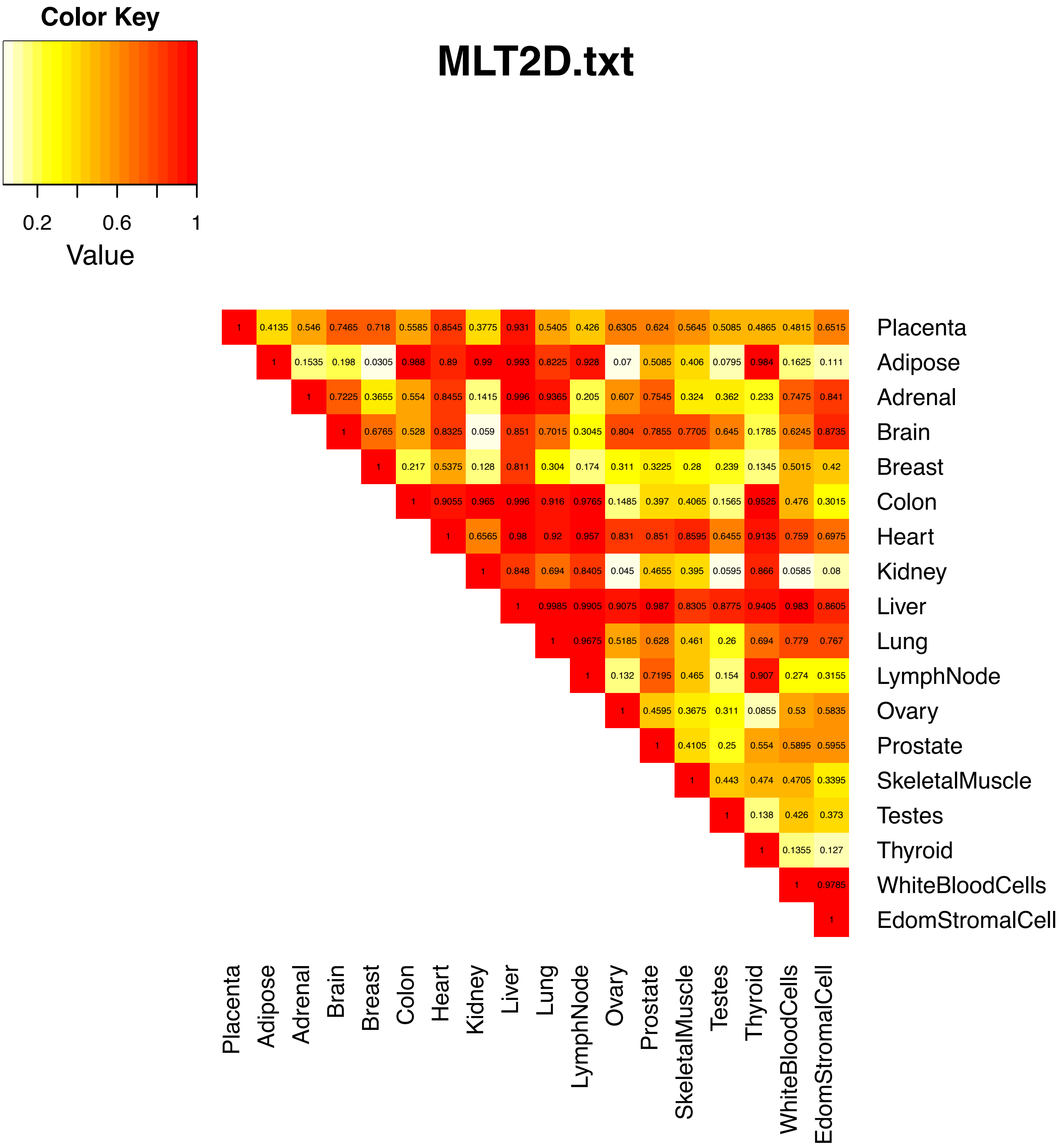
Color Key
0.2 0.6 1
Value
MLT2D.txt
Placenta: 1 0.4135 0.546 0.7465 0.718 0.5585 0.8545 0.3775 0.931 0.5405 0.426 0.6305 0.624 0.5645 0.5085 0.4865 0.4815 0.6515
Adipose: 1 0.1535 0.198 0.0305 0.988 0.89 0.99 0.993 0.8225 0.928 0.07 0.5085 0.406 0.0795 0.984 0.1625 0.111
Adrenal: 1 0.7225 0.3655 0.554 0.8455 0.1415 0.996 0.9365 0.205 0.607 0.7545 0.324 0.362 0.233 0.7475 0.841
Brain: 1 0.6765 0.528 0.8325 0.059 0.851 0.7015 0.3045 0.804 0.7855 0.7705 0.645 0.1785 0.6245 0.8735
Breast: 1 0.217 0.5375 0.128 0.811 0.304 0.174 0.311 0.3225 0.28 0.239 0.1345 0.5015 0.42
Colon: 1 0.9055 0.965 0.996 0.916 0.9765 0.1485 0.397 0.4065 0.1565 0.9525 0.476 0.3015
Heart: 1 0.6565 0.98 0.92 0.957 0.831 0.851 0.8595 0.6455 0.9135 0.759 0.6975
Kidney: 1 0.848 0.694 0.8405 0.045 0.4655 0.395 0.0595 0.866 0.0585 0.08
Liver: 1 0.9985 0.9905 0.9075 0.987 0.8305 0.8775 0.9405 0.983 0.8605
Lung: 1 0.9675 0.5185 0.628 0.461 0.26 0.694 0.779 0.767
LymphNode: 1 0.132 0.7195 0.465 0.154 0.907 0.274 0.3155
Ovary: 1 0.4595 0.3675 0.311 0.0855 0.53 0.5835
Prostate: 1 0.4105 0.25 0.554 0.5895 0.5955
SkeletalMuscle: 1 0.443 0.474 0.4705 0.3395
Testes: 1 0.138 0.426 0.373
Thyroid: 1 0.1355 0.127
WhiteBloodCells: 1 0.9785
EdomStromalCell: 1
Placenta Adipose Adrenal Brain Breast Colon Heart Kidney Liver Lung LymphNode Ovary Prostate SkeletalMuscle Testes Thyroid WhiteBloodCells EdomStromalCell

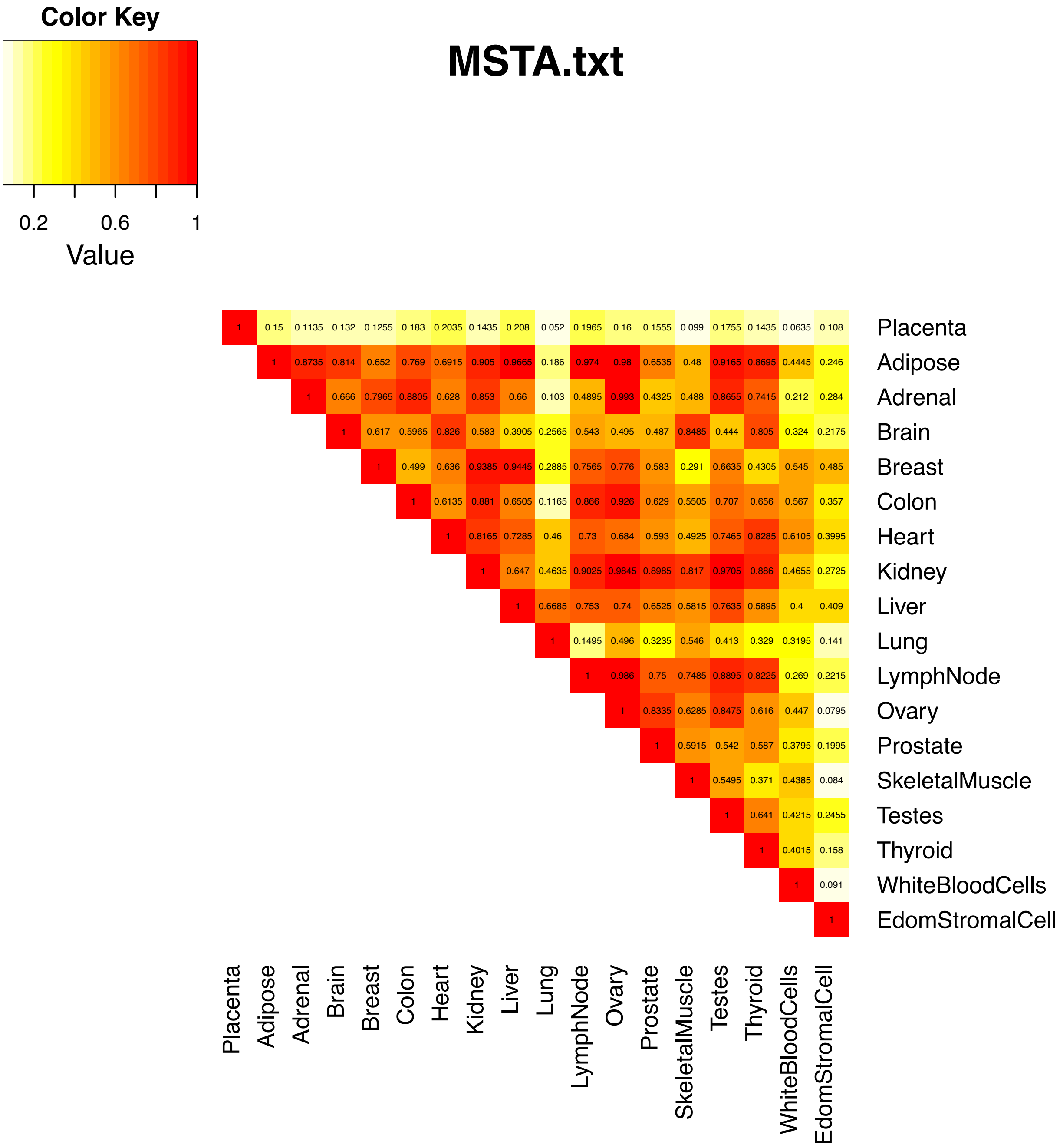

Color Key
0.2
0.6
1
Value
MSTA.txt
Placenta: 1 0.15 0.1135 0.132 0.1255 0.183 0.2035 0.1435 0.208 0.052 0.1965 0.16 0.1555 0.099 0.1755 0.1435 0.0635 0.108
Adipose: 1 0.8735 0.814 0.652 0.769 0.6915 0.905 0.9665 0.186 0.974 0.98 0.6535 0.48 0.9165 0.8695 0.4445 0.246
Adrenal: 1 0.666 0.7965 0.8805 0.628 0.853 0.66 0.103 0.4895 0.993 0.4325 0.488 0.8655 0.7415 0.212 0.284
Brain: 1 0.617 0.5965 0.826 0.583 0.3905 0.2565 0.543 0.495 0.487 0.8485 0.444 0.805 0.324 0.2175
Breast: 1 0.499 0.636 0.9385 0.9445 0.2885 0.7565 0.776 0.583 0.291 0.6635 0.4305 0.545 0.485
Colon: 1 0.6135 0.881 0.6505 0.1165 0.866 0.926 0.629 0.5505 0.707 0.656 0.567 0.357
Heart: 1 0.8165 0.7285 0.46 0.73 0.684 0.593 0.4925 0.7465 0.8285 0.6105 0.3995
Kidney: 1 0.647 0.4635 0.9025 0.9845 0.8985 0.817 0.9705 0.886 0.4655 0.2725
Liver: 1 0.6685 0.753 0.74 0.6525 0.5815 0.7635 0.5895 0.4 0.409
Lung: 1 0.1495 0.496 0.3235 0.546 0.413 0.329 0.3195 0.141
LymphNode: 1 0.986 0.75 0.7485 0.8895 0.8225 0.269 0.2215
Ovary: 1 0.8335 0.6285 0.8475 0.616 0.447 0.0795
Prostate: 1 0.5915 0.542 0.587 0.3795 0.1995
SkeletalMuscle: 1 0.5495 0.371 0.4385 0.084
Testes: 1 0.641 0.4215 0.2455
Thyroid: 1 0.4015 0.158
WhiteBloodCells: 1 0.091
EdomStromalCell: 1
Placenta Adipose Adrenal Brain Breast Colon Heart Kidney Liver Lung LymphNode Ovary Prostate SkeletalMuscle Testes Thyroid WhiteBloodCells EdomStromalCell

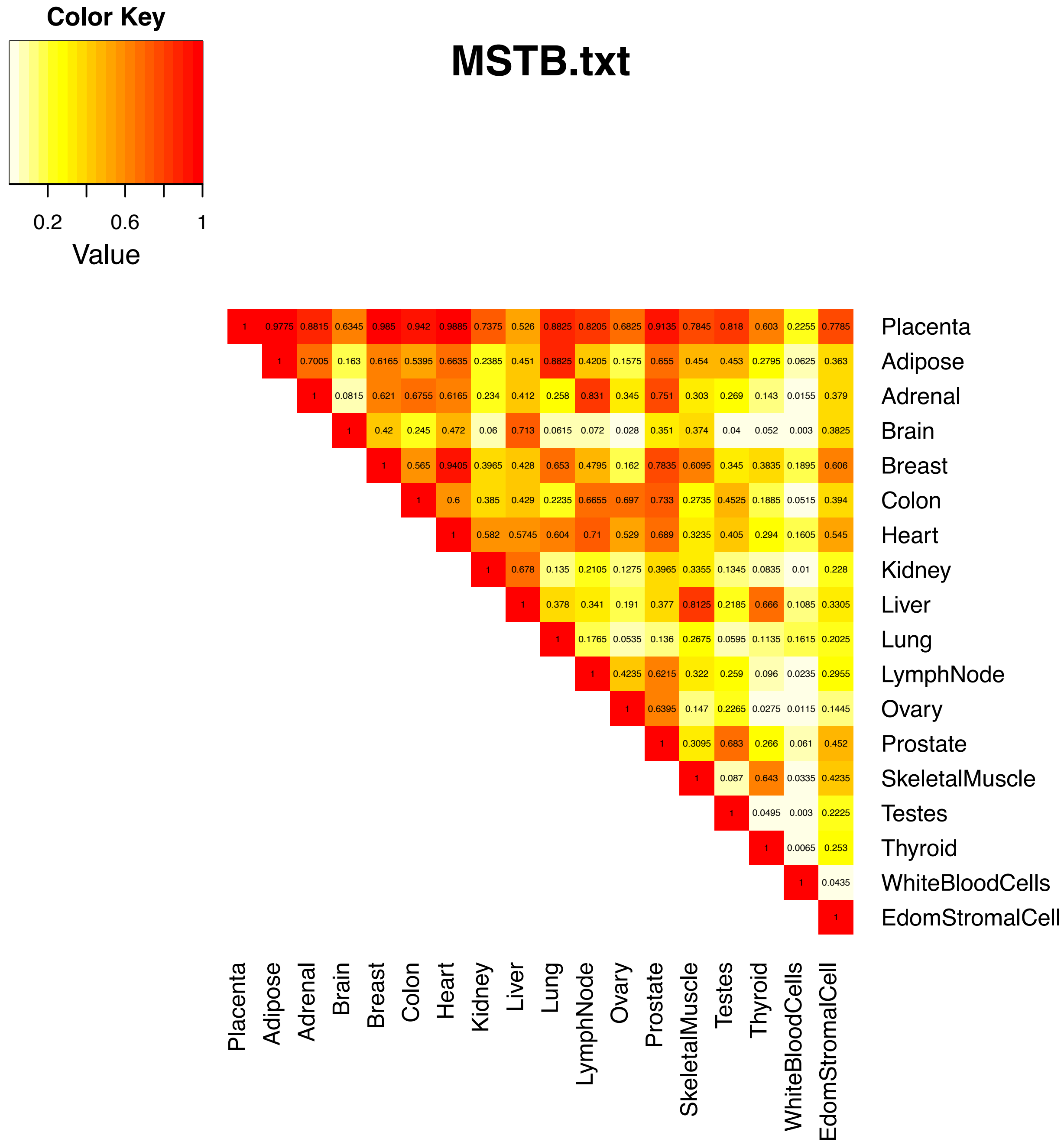
Color Key
0.2 0.6 1
Value
MSTB.txt
Placenta 1 0.9775 0.8815 0.6345 0.985 0.942 0.9885 0.7375 0.526 0.8825 0.8205 0.6825 0.9135 0.7845 0.818 0.603 0.2255 0.7785
Adipose 1 0.7005 0.163 0.6165 0.5395 0.6635 0.2385 0.451 0.8825 0.4205 0.1575 0.655 0.454 0.453 0.2795 0.0625 0.363
Adrenal 1 0.0815 0.621 0.6755 0.6165 0.234 0.412 0.258 0.831 0.345 0.751 0.303 0.269 0.143 0.0155 0.379
Brain 1 0.42 0.245 0.472 0.06 0.713 0.0615 0.072 0.028 0.351 0.374 0.04 0.052 0.003 0.3825
Breast 1 0.565 0.9405 0.3965 0.428 0.653 0.4795 0.162 0.7835 0.6095 0.345 0.3835 0.1895 0.606
Colon 1 0.6 0.385 0.429 0.2235 0.6655 0.697 0.733 0.2735 0.4525 0.1885 0.0515 0.394
Heart 1 0.582 0.5745 0.604 0.71 0.529 0.689 0.3235 0.405 0.294 0.1605 0.545
Kidney 1 0.678 0.135 0.2105 0.1275 0.3965 0.3355 0.1345 0.0835 0.01 0.228
Liver 1 0.378 0.341 0.191 0.377 0.8125 0.2185 0.666 0.1085 0.3305
Lung 1 0.1765 0.0535 0.136 0.2675 0.0595 0.1135 0.1615 0.2025
LymphNode 1 0.4235 0.6215 0.322 0.259 0.096 0.0235 0.2955
Ovary 1 0.6395 0.147 0.2265 0.0275 0.0115 0.1445
Prostate 1 0.3095 0.683 0.266 0.061 0.452
SkeletalMuscle 1 0.087 0.643 0.0335 0.4235
Testes 1 0.0495 0.003 0.2225
Thyroid 1 0.0065 0.253
WhiteBloodCells 1 0.0435
EdomStromalCell 1
Placenta
Adipose
Adrenal
Brain
Breast
Colon
Heart
Kidney
Liver
Lung
LymphNode
Ovary
Prostate
SkeletalMuscle
Testes
Thyroid
WhiteBloodCells
EdomStromalCell

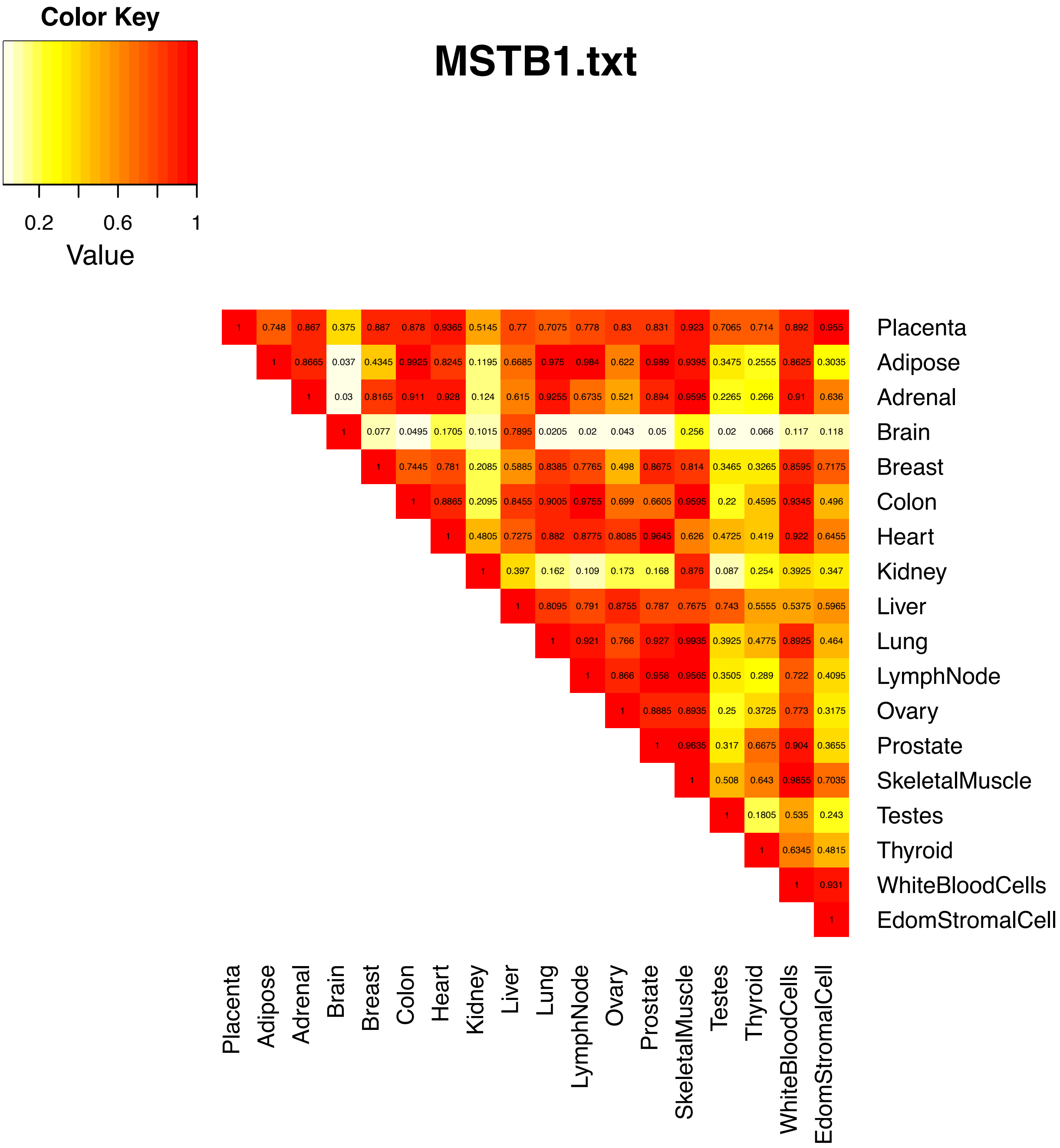
Color Key
0.2
0.6
1
Value
MSTB1.txt
Placenta: 1 0.748 0.867 0.375 0.887 0.878 0.9365 0.5145 0.77 0.7075 0.778 0.83 0.831 0.923 0.7065 0.714 0.892 0.955
Adipose: 1 0.8665 0.037 0.4345 0.9925 0.8245 0.1195 0.6685 0.975 0.984 0.622 0.989 0.9395 0.3475 0.2555 0.8625 0.3035
Adrenal: 1 0.03 0.8165 0.911 0.928 0.124 0.615 0.9255 0.6735 0.521 0.894 0.9595 0.2265 0.266 0.91 0.636
Brain: 1 0.077 0.0495 0.1705 0.1015 0.7895 0.0205 0.02 0.043 0.05 0.256 0.02 0.066 0.117 0.118
Breast: 1 0.7445 0.781 0.2085 0.5885 0.8385 0.7765 0.498 0.8675 0.814 0.3465 0.3265 0.8595 0.7175
Colon: 1 0.8865 0.2095 0.8455 0.9005 0.9755 0.699 0.6605 0.9595 0.22 0.4595 0.9345 0.496
Heart: 1 0.4805 0.7275 0.882 0.8775 0.8085 0.9645 0.626 0.4725 0.419 0.922 0.6455
Kidney: 1 0.397 0.162 0.109 0.173 0.168 0.876 0.087 0.254 0.3925 0.347
Liver: 1 0.8095 0.791 0.8755 0.787 0.7675 0.743 0.5555 0.5375 0.5965
Lung: 1 0.921 0.766 0.927 0.9935 0.3925 0.4775 0.8925 0.464
LymphNode: 1 0.866 0.958 0.9565 0.3505 0.289 0.722 0.4095
Ovary: 1 0.8885 0.8935 0.25 0.3725 0.773 0.3175
Prostate: 1 0.9635 0.317 0.6675 0.904 0.3655
SkeletalMuscle: 1 0.508 0.643 0.9855 0.7035
Testes: 1 0.1805 0.535 0.243
Thyroid: 1 0.6345 0.4815
WhiteBloodCells: 1 0.931
EdomStromalCell: 1
Placenta Adipose Adrenal Brain Breast Colon Heart Kidney Liver Lung LymphNode Ovary Prostate SkeletalMuscle Testes Thyroid WhiteBloodCells EdomStromalCell

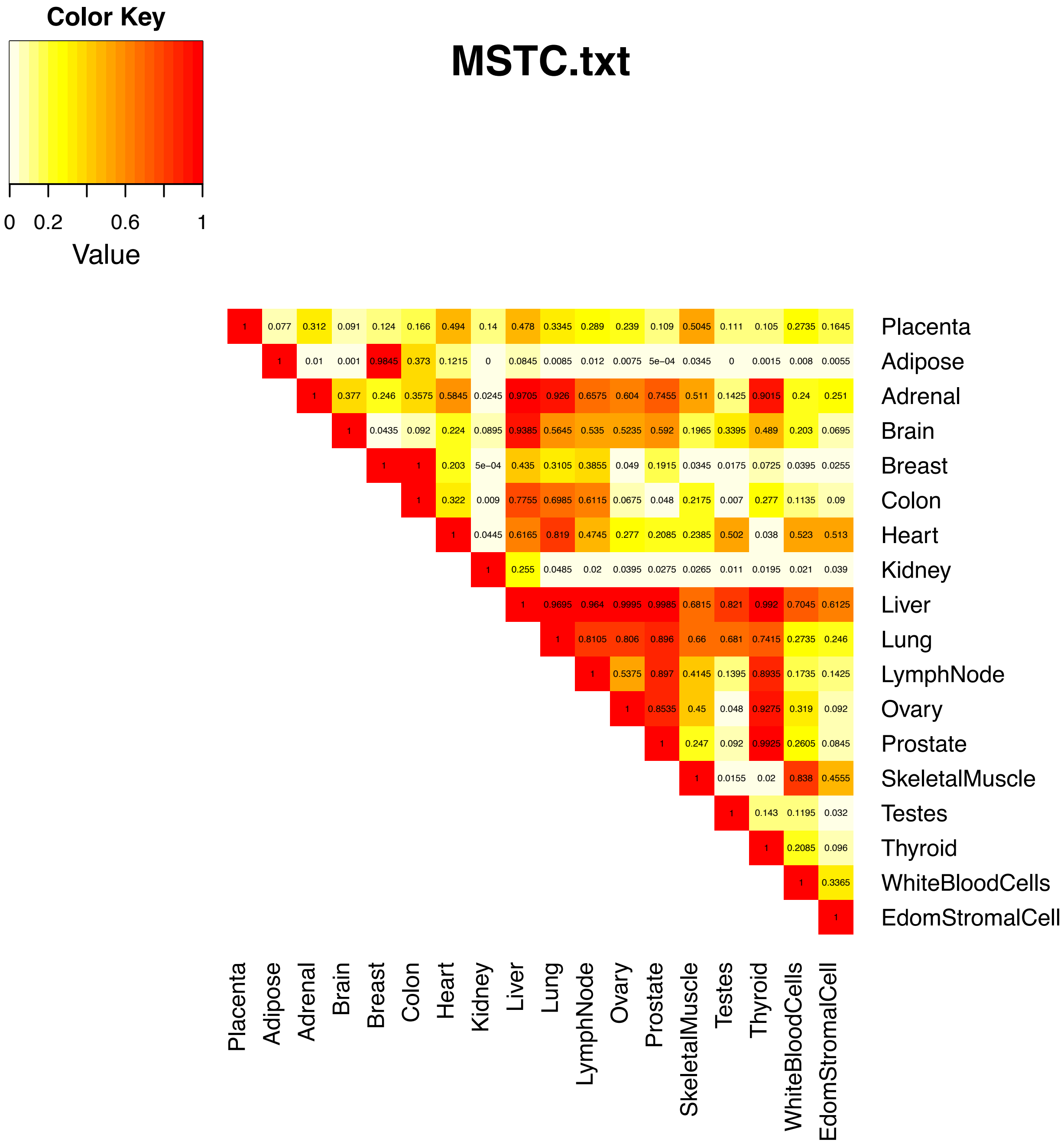
Color Key
0
0.2
0.6
1
Value
MSTC.txt
Placenta: 1 0.077 0.312 0.091 0.124 0.166 0.494 0.14 0.478 0.3345 0.289 0.239 0.109 0.5045 0.111 0.105 0.2735 0.1645
Adipose: 1 0.01 0.001 0.9845 0.373 0.1215 0 0.0845 0.0085 0.012 0.0075 5e−04 0.0345 0 0.0015 0.008 0.0055
Adrenal: 1 0.377 0.246 0.3575 0.5845 0.0245 0.9705 0.926 0.6575 0.604 0.7455 0.511 0.1425 0.9015 0.24 0.251
Brain: 1 0.0435 0.092 0.224 0.0895 0.9385 0.5645 0.535 0.5235 0.592 0.1965 0.3395 0.489 0.203 0.0695
Breast: 1 1 0.203 5e−04 0.435 0.3105 0.3855 0.049 0.1915 0.0345 0.0175 0.0725 0.0395 0.0255
Colon: 1 0.322 0.009 0.7755 0.6985 0.6115 0.0675 0.048 0.2175 0.007 0.277 0.1135 0.09
Heart: 1 0.0445 0.6165 0.819 0.4745 0.277 0.2085 0.2385 0.502 0.038 0.523 0.513
Kidney: 1 0.255 0.0485 0.02 0.0395 0.0275 0.0265 0.011 0.0195 0.021 0.039
Liver: 1 0.9695 0.964 0.9995 0.9985 0.6815 0.821 0.992 0.7045 0.6125
Lung: 1 0.8105 0.806 0.896 0.66 0.681 0.7415 0.2735 0.246
LymphNode: 1 0.5375 0.897 0.4145 0.1395 0.8935 0.1735 0.1425
Ovary: 1 0.8535 0.45 0.048 0.9275 0.319 0.092
Prostate: 1 0.247 0.092 0.9925 0.2605 0.0845
SkeletalMuscle: 1 0.0155 0.02 0.838 0.4555
Testes: 1 0.143 0.1195 0.032
Thyroid: 1 0.2085 0.096
WhiteBloodCells: 1 0.3365
EdomStromalCell: 1
Placenta
Adipose
Adrenal
Brain
Breast
Colon
Heart
Kidney
Liver
Lung
LymphNode
Ovary
Prostate
SkeletalMuscle
Testes
Thyroid
WhiteBloodCells
EdomStromalCell

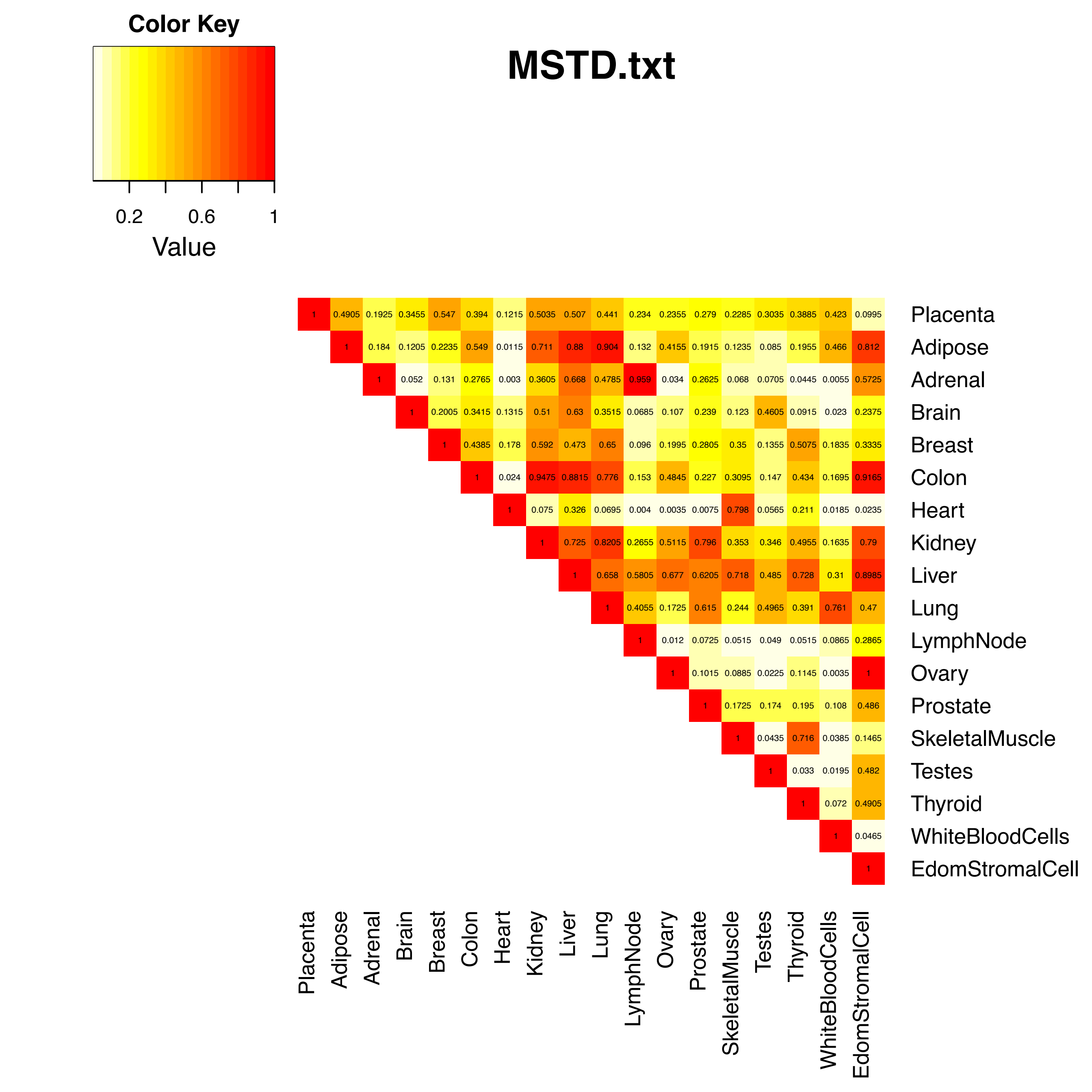
Color Key
0.2
0.6
1
Value
MSTD.txt
Placenta: 1 0.4905 0.1925 0.3455 0.547 0.394 0.1215 0.5035 0.507 0.441 0.234 0.2355 0.279 0.2285 0.3035 0.3885 0.423 0.0995
Adipose: 1 0.184 0.1205 0.2235 0.549 0.0115 0.711 0.88 0.904 0.132 0.4155 0.1915 0.1235 0.085 0.1955 0.466 0.812
Adrenal: 1 0.052 0.131 0.2765 0.003 0.3605 0.668 0.4785 0.959 0.034 0.2625 0.068 0.0705 0.0445 0.0055 0.5725
Brain: 1 0.2005 0.3415 0.1315 0.51 0.63 0.3515 0.0685 0.107 0.239 0.123 0.4605 0.0915 0.023 0.2375
Breast: 1 0.4385 0.178 0.592 0.473 0.65 0.096 0.1995 0.2805 0.35 0.1355 0.5075 0.1835 0.3335
Colon: 1 0.024 0.9475 0.8815 0.776 0.153 0.4845 0.227 0.3095 0.147 0.434 0.1695 0.9165
Heart: 1 0.075 0.326 0.0695 0.004 0.0035 0.0075 0.798 0.0565 0.211 0.0185 0.0235
Kidney: 1 0.725 0.8205 0.2655 0.5115 0.796 0.353 0.346 0.4955 0.1635 0.79
Liver: 1 0.658 0.5805 0.677 0.6205 0.718 0.485 0.728 0.31 0.8985
Lung: 1 0.4055 0.1725 0.615 0.244 0.4965 0.391 0.761 0.47
LymphNode: 1 0.012 0.0725 0.0515 0.049 0.0515 0.0865 0.2865
Ovary: 1 0.1015 0.0885 0.0225 0.1145 0.0035 1
Prostate: 1 0.1725 0.174 0.195 0.108 0.486
SkeletalMuscle: 1 0.0435 0.716 0.0385 0.1465
Testes: 1 0.033 0.0195 0.482
Thyroid: 1 0.072 0.4905
WhiteBloodCells: 1 0.0465
EdomStromalCell: 1
Placenta
Adipose
Adrenal
Brain
Breast
Colon
Heart
Kidney
Liver
Lung
LymphNode
Ovary
Prostate
SkeletalMuscle
Testes
Thyroid
WhiteBloodCells
EdomStromalCell

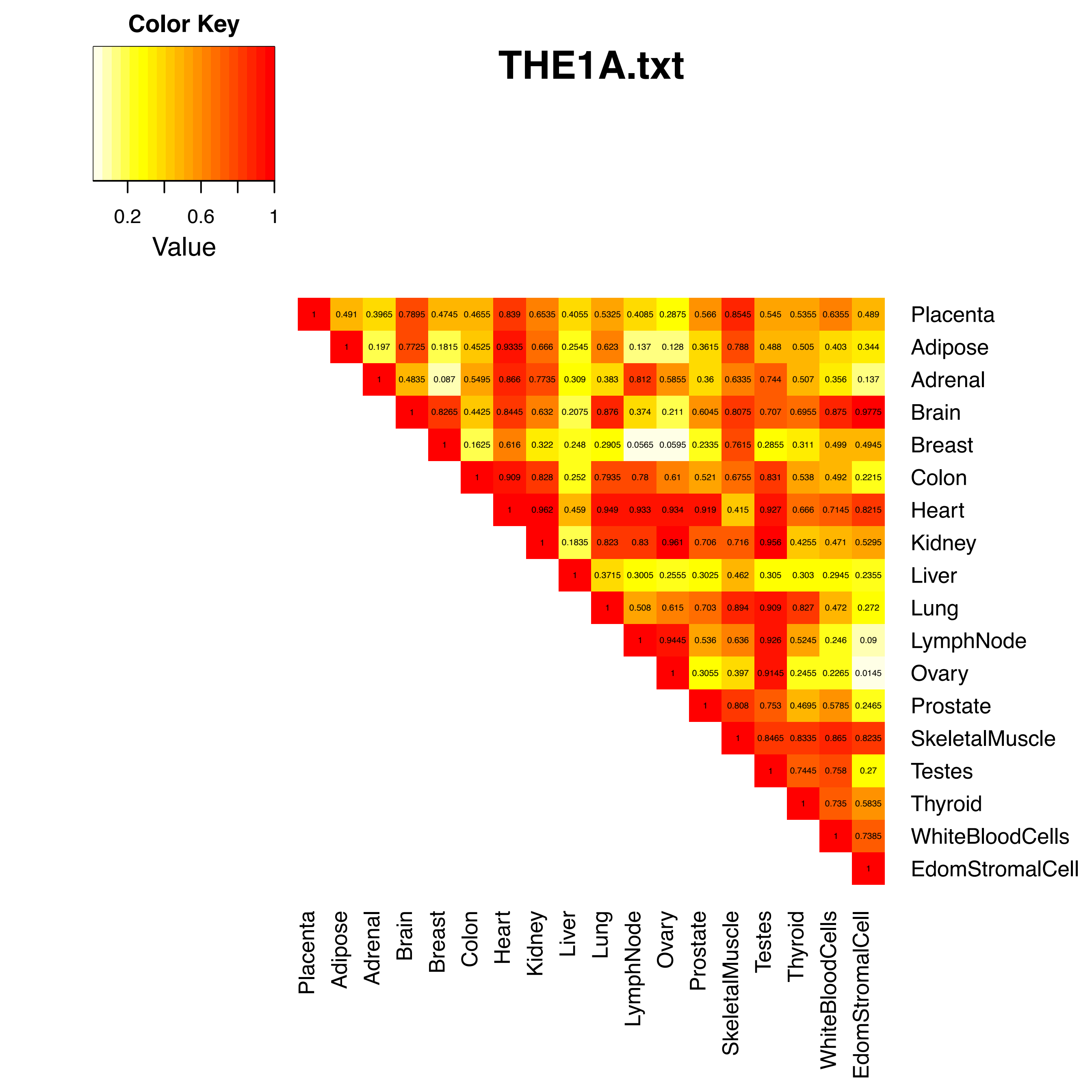
Color Key
0.2 0.6 1
Value
THE1A.txt
Placenta 1 0.491 0.3965 0.7895 0.4745 0.4655 0.839 0.6535 0.4055 0.5325 0.4085 0.2875 0.566 0.8545 0.545 0.5355 0.6355 0.489
Adipose 1 0.197 0.7725 0.1815 0.4525 0.9335 0.666 0.2545 0.623 0.137 0.128 0.3615 0.788 0.488 0.505 0.403 0.344
Adrenal 1 0.4835 0.087 0.5495 0.866 0.7735 0.309 0.383 0.812 0.5855 0.36 0.6335 0.744 0.507 0.356 0.137
Brain 1 0.8265 0.4425 0.8445 0.632 0.2075 0.876 0.374 0.211 0.6045 0.8075 0.707 0.6955 0.875 0.9775
Breast 1 0.1625 0.616 0.322 0.248 0.2905 0.0565 0.0595 0.2335 0.7615 0.2855 0.311 0.499 0.4945
Colon 1 0.909 0.828 0.252 0.7935 0.78 0.61 0.521 0.6755 0.831 0.538 0.492 0.2215
Heart 1 0.962 0.459 0.949 0.933 0.934 0.919 0.415 0.927 0.666 0.7145 0.8215
Kidney 1 0.1835 0.823 0.83 0.961 0.706 0.716 0.956 0.4255 0.471 0.5295
Liver 1 0.3715 0.3005 0.2555 0.3025 0.462 0.305 0.303 0.2945 0.2355
Lung 1 0.508 0.615 0.703 0.894 0.909 0.827 0.472 0.272
LymphNode 1 0.9445 0.536 0.636 0.926 0.5245 0.246 0.09
Ovary 1 0.3055 0.397 0.9145 0.2455 0.2265 0.0145
Prostate 1 0.808 0.753 0.4695 0.5785 0.2465
SkeletalMuscle 1 0.8465 0.8335 0.865 0.8235
Testes 1 0.7445 0.758 0.27
Thyroid 1 0.735 0.5835
WhiteBloodCells 1 0.7385
EdomStromalCell 1
Placenta Adipose Adrenal Brain Breast Colon Heart Kidney Liver Lung LymphNode Ovary Prostate SkeletalMuscle Testes Thyroid WhiteBloodCells EdomStromalCell

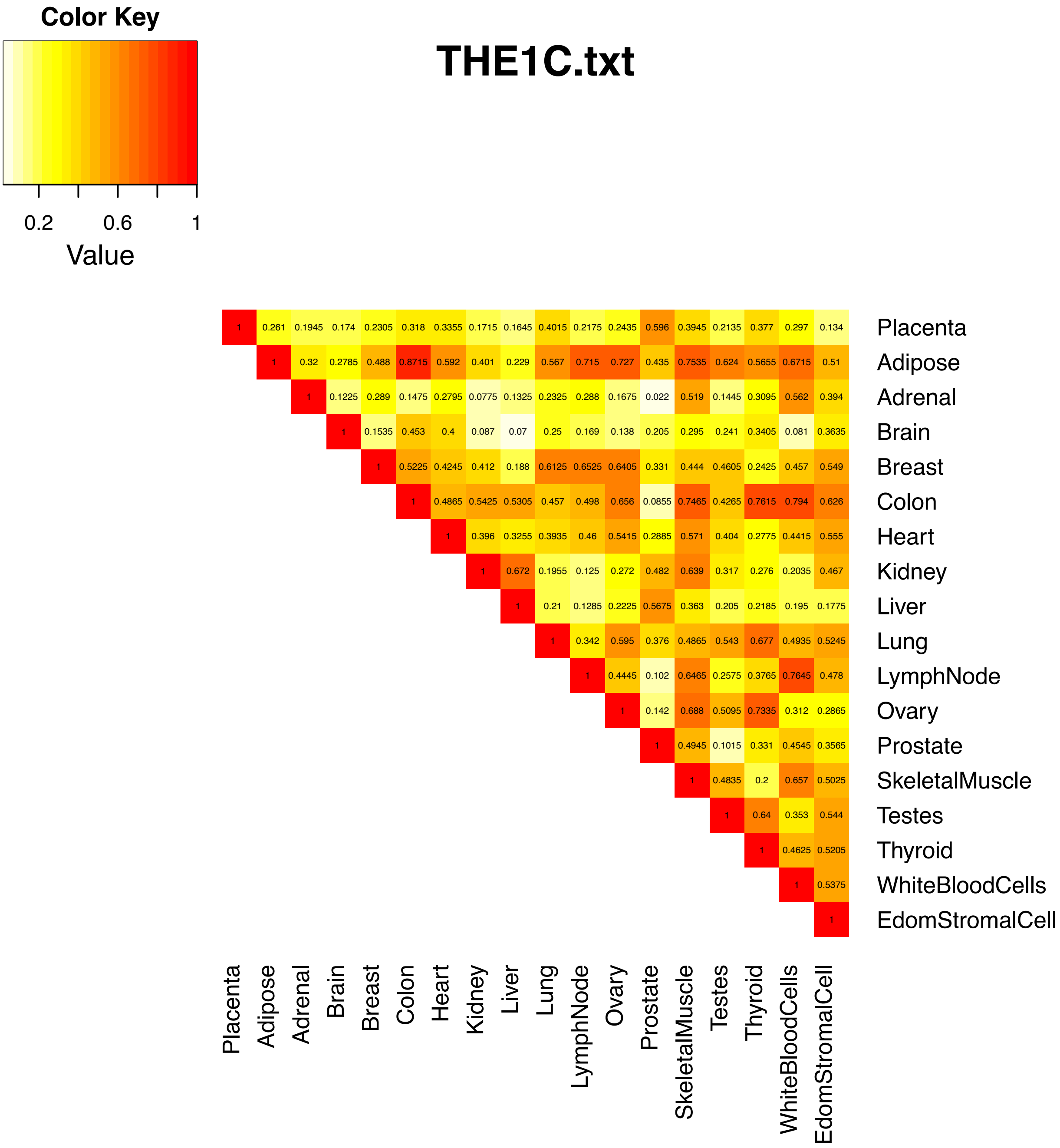
Color Key
0.2
0.6
1
Value
THE1C.txt
Placenta 1 0.261 0.1945 0.174 0.2305 0.318 0.3355 0.1715 0.1645 0.4015 0.2175 0.2435 0.596 0.3945 0.2135 0.377 0.297 0.134
Adipose 1 0.32 0.2785 0.488 0.8715 0.592 0.401 0.229 0.567 0.715 0.727 0.435 0.7535 0.624 0.5655 0.6715 0.51
Adrenal 1 0.1225 0.289 0.1475 0.2795 0.0775 0.1325 0.2325 0.288 0.1675 0.022 0.519 0.1445 0.3095 0.562 0.394
Brain 1 0.1535 0.453 0.4 0.087 0.07 0.25 0.169 0.138 0.205 0.295 0.241 0.3405 0.081 0.3635
Breast 1 0.5225 0.4245 0.412 0.188 0.6125 0.6525 0.6405 0.331 0.444 0.4605 0.2425 0.457 0.549
Colon 1 0.4865 0.5425 0.5305 0.457 0.498 0.656 0.0855 0.7465 0.4265 0.7615 0.794 0.626
Heart 1 0.396 0.3255 0.3935 0.46 0.5415 0.2885 0.571 0.404 0.2775 0.4415 0.555
Kidney 1 0.672 0.1955 0.125 0.272 0.482 0.639 0.317 0.276 0.2035 0.467
Liver 1 0.21 0.1285 0.2225 0.5675 0.363 0.205 0.2185 0.195 0.1775
Lung 1 0.342 0.595 0.376 0.4865 0.543 0.677 0.4935 0.5245
LymphNode 1 0.4445 0.102 0.6465 0.2575 0.3765 0.7645 0.478
Ovary 1 0.142 0.688 0.5095 0.7335 0.312 0.2865
Prostate 1 0.4945 0.1015 0.331 0.4545 0.3565
SkeletalMuscle 1 0.4835 0.2 0.657 0.5025
Testes 1 0.64 0.353 0.544
Thyroid 1 0.4625 0.5205
WhiteBloodCells 1 0.5375
EdomStromalCell 1
Placenta
Adipose
Adrenal
Brain
Breast
Colon
Heart
Kidney
Liver
Lung
LymphNode
Ovary
Prostate
SkeletalMuscle
Testes
Thyroid
WhiteBloodCells
EdomStromalCell

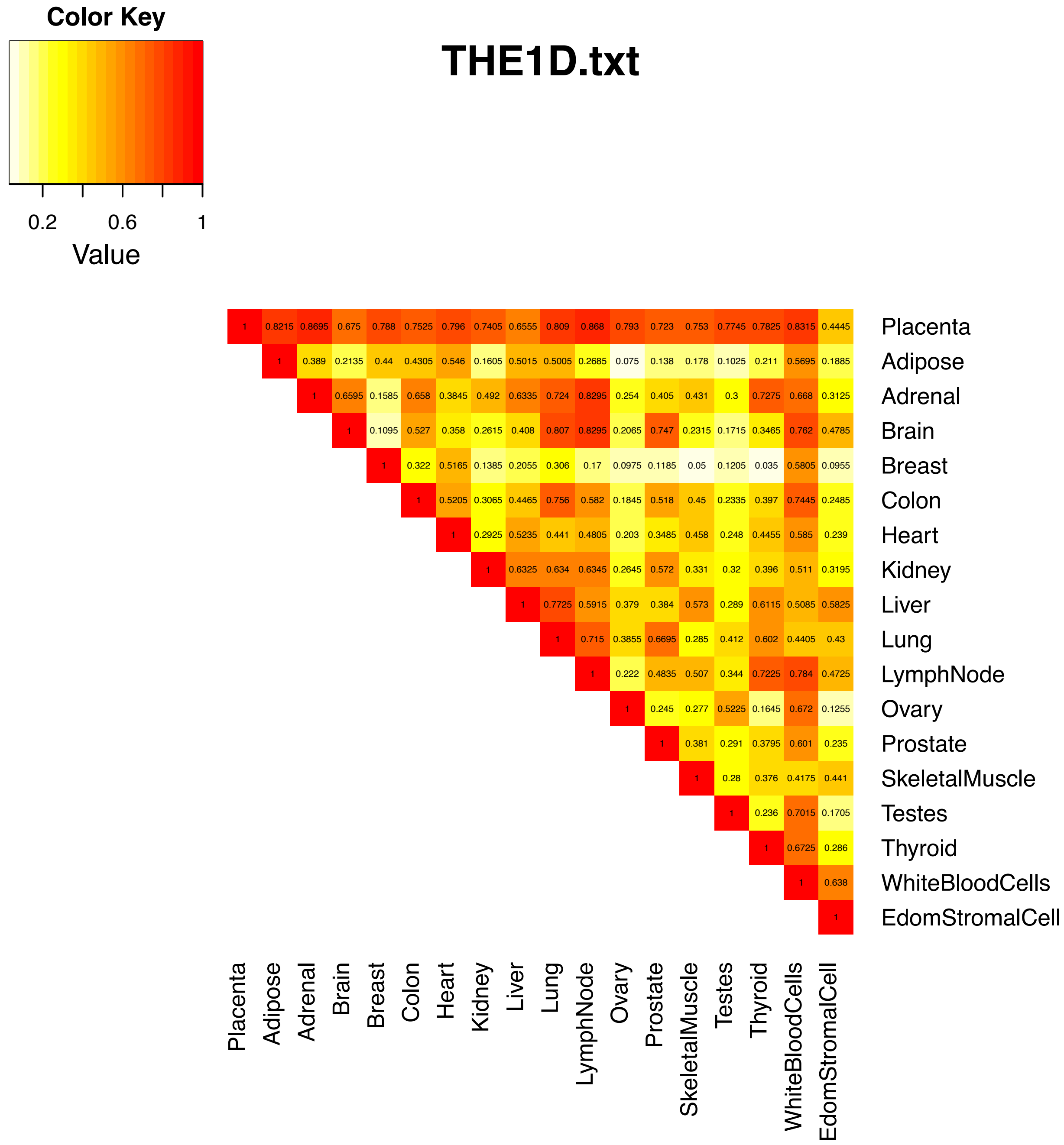
Color Key
0.2
0.6
1
Value
THE1D.txt
Placenta 1 0.8215 0.8695 0.675 0.788 0.7525 0.796 0.7405 0.6555 0.809 0.868 0.793 0.723 0.753 0.7745 0.7825 0.8315 0.4445
Adipose 1 0.389 0.2135 0.44 0.4305 0.546 0.1605 0.5015 0.5005 0.2685 0.075 0.138 0.178 0.1025 0.211 0.5695 0.1885
Adrenal 1 0.6595 0.1585 0.658 0.3845 0.492 0.6335 0.724 0.8295 0.254 0.405 0.431 0.3 0.7275 0.668 0.3125
Brain 1 0.1095 0.527 0.358 0.2615 0.408 0.807 0.8295 0.2065 0.747 0.2315 0.1715 0.3465 0.762 0.4785
Breast 1 0.322 0.5165 0.1385 0.2055 0.306 0.17 0.0975 0.1185 0.05 0.1205 0.035 0.5805 0.0955
Colon 1 0.5205 0.3065 0.4465 0.756 0.582 0.1845 0.518 0.45 0.2335 0.397 0.7445 0.2485
Heart 1 0.2925 0.5235 0.441 0.4805 0.203 0.3485 0.458 0.248 0.4455 0.585 0.239
Kidney 1 0.6325 0.634 0.6345 0.2645 0.572 0.331 0.32 0.396 0.511 0.3195
Liver 1 0.7725 0.5915 0.379 0.384 0.573 0.289 0.6115 0.5085 0.5825
Lung 1 0.715 0.3855 0.6695 0.285 0.412 0.602 0.4405 0.43
LymphNode 1 0.222 0.4835 0.507 0.344 0.7225 0.784 0.4725
Ovary 1 0.245 0.277 0.5225 0.1645 0.672 0.1255
Prostate 1 0.381 0.291 0.3795 0.601 0.235
SkeletalMuscle 1 0.28 0.376 0.4175 0.441
Testes 1 0.236 0.7015 0.1705
Thyroid 1 0.6725 0.286
WhiteBloodCells 1 0.638
EdomStromalCell 1
Placenta
Adipose
Adrenal
Brain
Breast
Colon
Heart
Kidney
Liver
Lung
LymphNode
Ovary
Prostate
SkeletalMuscle
Testes
Thyroid
WhiteBloodCells
EdomStromalCell
